# Marker genes that are less conserved in their sequences are useful for predicting genome-wide similarity levels between closely related prokaryotic strains

**DOI:** 10.1186/s40168-016-0162-5

**Published:** 2016-05-03

**Authors:** Yemin Lan, Gail Rosen, Ruth Hershberg

**Affiliations:** School of Biomedical Engineering, Science and Health Systems, Drexel University, 3141 Chestnut Street, Philadelphia, PA 19104 USA; Ecological and Evolutionary Signal-processing and Informatics Laboratory, Electrical & Computer Engineering Department, Drexel University, 3141 Chestnut Street, Philadelphia, PA 19104 USA; Rachel & Menachem Mendelovitch Evolutionary Processes of Mutation & Natural Selection Research Laboratory, Department of Genetics and Developmental Biology, the Ruth and Bruce Rappaport Faculty of Medicine, Technion-Israel Institute of Technology, 31096 Haifa, Israel

**Keywords:** Less-conserved genes, Lineage-specific, Marker genes, Genome-wide similarity

## Abstract

**Background:**

The 16s rRNA gene is so far the most widely used marker for taxonomical classification and separation of prokaryotes. Since it is universally conserved among prokaryotes, it is possible to use this gene to classify a broad range of prokaryotic organisms. At the same time, it has often been noted that the 16s rRNA gene is too conserved to separate between prokaryotes at finer taxonomic levels.

**Results:**

In this paper, we examine how well levels of similarity of 16s rRNA and 73 additional universal or nearly universal marker genes correlate with genome-wide levels of gene sequence similarity. We demonstrate that the percent identity of 16s rRNA predicts genome-wide levels of similarity very well for distantly related prokaryotes, but not for closely related ones. In closely related prokaryotes, we find that there are many other marker genes for which levels of similarity are much more predictive of genome-wide levels of gene sequence similarity. Finally, we show that the identities of the markers that are most useful for predicting genome-wide levels of similarity within closely related prokaryotic lineages vary greatly between lineages. However, the most useful markers are always those that are least conserved in their sequences within each lineage.

**Conclusions:**

Our results show that by choosing markers that are less conserved in their sequences within a lineage of interest, it is possible to better predict genome-wide gene sequence similarity between closely related prokaryotes than is possible using the 16s rRNA gene. We point readers towards a database we have created (POGO-DB) that can be used to easily establish which markers show lowest levels of sequence conservation within different prokaryotic lineages.

**Electronic supplementary material:**

The online version of this article (doi:10.1186/s40168-016-0162-5) contains supplementary material, which is available to authorized users.

## Background

One key aim of microbiome studies is to characterize the genomic diversity of prokaryotic species present within environments of interest. However, addressing the genomic diversity can be highly challenging for several reasons. These reasons include technical limitations, such as short read length of high-throughput sequencing, as well as biological challenges that result from prevalent horizontal gene transfer and variation in rates of evolution between different genomes, and within genomes between different genes [1].

In studies where resolving the taxonomical composition of a microbiome is the main goal, it is common practice to amplify and sequence a marker gene and infer the microbiome composition based on its sequence. In most such studies, the 16s rRNA gene serves as the marker of choice [2–5]. The 16s rRNA gene is universally present in prokaryotes and is rarely affected by horizontal gene transfer (HGT) [6, 7]. The 16s rRNA gene contains several hyper-variable regions, allowing for the distinction of different prokaryotes [8]. At the same time, outside of the hyper-variable regions, the sequence of 16s rRNA is relatively very highly conserved across prokaryotes. This allows for the design of universal primers that can be used to amplify 16s rRNA from a very large fraction of prokaryotes. These special characteristics of the 16s rRNA gene make it a useful marker for taxonomical classification and separation, giving rise to various 16s identification tools such as the Ribosomal Database Project [9], Greengenes [10], and SILVA [11].

While 16s rRNA has been demonstrated to be highly useful for taxonomical classification and separation in a great number of microbiome studies, its limitations have also been noted [12, 13]. Despite its hyper-variable regions, the sequence of 16s rRNA tends to be relatively conserved. This makes this gene very useful for studying levels of variation between distantly related prokaryotes. However, it may make 16s rRNA less useful for distinguishing between closely related prokaryotes. In a recent attempt to evaluate species-level identification using the 16s rRNA gene, it was shown that the trade-off between precision and recall could not be well balanced [14]. More recently, oligotyping has been introduced as a method to detect closely related lineages using the 16s rRNA gene [15]. While the method provides finer taxonomic breakdown of the sequenced microbiomes, it provides little information to resolve the relationship between taxa. It was also shown that the 16s rRNA gene has variable explanatory power between prokaryotic lineages, explaining as little as 28 % of the variance among *Enterobacteria* genomes and as much as 70 % of the variance among *Bacteroidetes* [16]. Additionally, some studies suggest that, contrary to previous assumptions, the 16s rRNA gene can be horizontally transferred [17]. Finally, the 16s rRNA gene has varying number of copies in approximately 80 % of all fully sequenced prokaryotic genomes, which may greatly skew estimates of the prokaryotic abundances in a community.

To overcome these limitations, the use of alternative marker genes has been suggested. In several studies, an essential housekeeping gene, such as *rpoB*, *amoA*, *pmoA*, *nirS*, *nirK*, *nosZ*, and *pufM*, was used to determine taxonomical relationships for lineages of interest [12, 18–22]. For example, the *recA* gene was used to provide unambiguous identification of *Lactobacillus* strains [21]. The *rpoB* gene was used to estimate biodiversity in a soil sample [12] and reconstruct taxonomical relationships among strains belonging to the *Halobacteriales* lineage [19]. The *rpoB* gene was also shown to be more useful than 16s rRNA at discriminating closely related organisms [20]. The chaperonin-60 universal target was shown to be a particularly useful marker as it was demonstrated to predict similar bacterial genome relatedness to whole genome sequence alignments over a broad range of taxa [22]. More recently, taxonomical inference dependent on whole genome sequencing and combined use of a variety of markers has been repeatedly proposed, where the definition of markers ranged from housekeeping genes to representative homologous gene groups and exclusive genetic episodes [23–26]. In these studies, housekeeping genes have been suggested to be useful for discriminating lineages, as they are a major component of the core genes for a lineage [25] and are thought to be subject to less environmental pressure than other genes [27]. Wu et al. identified 31 housekeeping genes from 100 genomes that can be used to classify and separate prokaryotes [23]. These markers have been used to speed up the taxonomic classification based on metagenomic shotgun data [24]. Ciccarelli et al. have used a concatenation of 31 marker genes to reconstruct the tree of life for 191 species with their whole genome sequenced at the time [28]. Another comprehensive study has surveyed 32 protein-coding genes that are widely distributed among bacterial genomes and demonstrated the usefulness of single-gene alignments in predicting genome relatedness in specific lineages [29].

In the meantime, whole genome sequencing (WGS) has become more feasible and more prevalent in microbiome studies [30–32]. In comparison with the sequencing of a single marker gene, WGS uncovers both the microbial composition and functional composition of the microbiomes of interest. Hence, various tools are developed over the past few years to facilitate analysis of WGS data, such as WGSQuikr [33] and MetaPhlAn [34]. In addition to taking advantage of the full microbiome sequenced from WGS, some approaches resort to a small subset of widely conserved marker genes mined from WGS data, such as PhyloSift [35], AMPHORA2 [36], MetaPhyler [24], EMIRGE [37], and PhylOTU [38]. Consequently, in various scenarios, many widely conserved genes other than the 16s rRNA gene have proven valuable towards better assessment of interspecies relationships [19–21, 24, 26, 39]. While WGS has become cheaper and more popular in microbiome studies, use of a single marker gene similar to that of the 16s rRNA gene still has its advantage in effectiveness and efficiency. Despite the well-understood benefits of utilizing marker genes for classifying and separating prokaryotes, a major challenge remains in determining which marker genes are most useful for different lineages and various scenarios. Therefore, a systematic analysis of the utility of different marker genes and the development of criteria for choosing the correct marker genes should yet prove beneficial.

Here, we conducted genome-wide comparisons of levels of gene similarity of ~2000 fully sequenced genomes. For each genome pair compared, we also estimated levels of similarity of the 16s rRNA gene as well as of 73 additional marker genes that are each present within at least 90 % of all prokaryotes. We show that levels of similarity of 16s rRNA are very good predictors of genome-wide levels of similarity for distantly related prokaryotes. At the same time, many other marker genes are much more useful than 16s rRNA for predicting genome-wide levels of similarity for more closely related prokaryotes. The identity of the most useful markers varies between prokaryotic lineages. However, within each lineage, the markers that are most useful for predicting genome-wide levels of similarity are those markers that have the lowest levels of sequence conservation within that lineage. Our results indicate that it should be possible to obtain far better separation of closely related strains in lineage-specific prokaryotic studies by using markers that are less conserved in their sequences.

## Methods

### Extracting whole genome and 16s rRNA gene sequences

The complete genomes of 2013 prokaryotic strains were downloaded from the NCBI database (in July, 2012). Genes annotated as “16s rRNA gene” were extracted from each strain. A total of 1897 genomes were retained with 16s rRNA genes of legitimate length from 1000- to 1800-bp nucleotides [8].

### Identification of universal or nearly universal marker genes

Single-copy and universally distributed genes in the COG database [40] were considered as potential markers (Additional file 1: Table S1). Copies of these genes in each genome were recognized from BLAST reciprocal best hits, using the copies of these genes in *Escherichia coli K12 W3110* (NCBI Genome unique ID 161931) as references. Each of the 73 potential markers was found to be present in more than 90 % of the prokaryotic genomes. A total of 1204 genomes harbor all marker genes.

### Calculating marker gene percent identity

For each pair of genomes, all 16s rRNA gene copies were pairwise-aligned using the Needleman-Wunsch algorithm [41]. The maximal 16s rRNA identity was recorded for each genome pair. Likewise, the percent identity of each marker gene between a pair of genomes was obtained by aligning the nucleotide sequences using the pairwise Needleman-Wunsch alignment algorithm.

### Calculating average AAI

The average amino acid identity (AAI) was computed for all genome pairs whose maximal 16s rRNA gene identity was at least 80 %. Towards this end, protein-coding sequences in each genome were obtained from the NCBI Genome database [42]. Homologs between a pair of genomes were identified by reciprocal BLAST comparisons [43] and re-aligned using the Smith-Waterman algorithm [44]. To be defined as homologs, proteins had to be aligned over at least 70 % of the shorter sequence and the AAI had to be above 30 % [45]. The average AAI was computed using all identified homologs between two genomes, if they had at least 200 homologs (out of 717,861 pairwise comparisons, 2556 were discarded because less than 200 homologs were identified).

### Average ranking of marker genes

Focusing only on genomes that contain all 74 marker genes (including the 16s rRNA gene), we calculated for each genome pair the relative rank of each marker gene by their percent identities within that genome pair. The marker gene that was least similar between the two genomes (i.e., had the lowest percent identity) received a rank of 74, and the marker gene that was most similar (i.e., had the highest percent identity) received a rank of 1. The average ranking of a gene within a certain lineage was then calculated based on its ranking for each pair of genomes from that lineage. At the end of this process, lower ranks (closer to 1) correspond to more conserved genes.

### Generating trees based on average AAIs and based on marker gene percent identities

Trees were generated using the Unweighted Pair Group Method with Arithmetic Mean (UPGMA) algorithm, where the pairwise distance matrix values were one minus the AAIs or one minus the percent identities of a specific marker gene. Split distance (equivalent to the Robinson-Foulds distance) between trees was calculated using TOPD/FMTS software [46].

## Results and discussion

### The 16s rRNA gene is an ambiguous marker for inferring extent of similarity of closely related prokaryotes

The percent identity of the 16s rRNA gene is considered to be indicative of the taxonomical relationship between prokaryotes. For example, sequences with a percent identity of 97 % or higher are considered by many as coming from the same species [47] and a percent identity of 94 % or higher is thought to indicate that the sampled prokaryotes belong to the same genus [16]. However, as is discovered often and especially at finer taxonomic levels, relying on the 16s rRNA alone may give faulty inferences [12, 13].

**Fig. 1:**
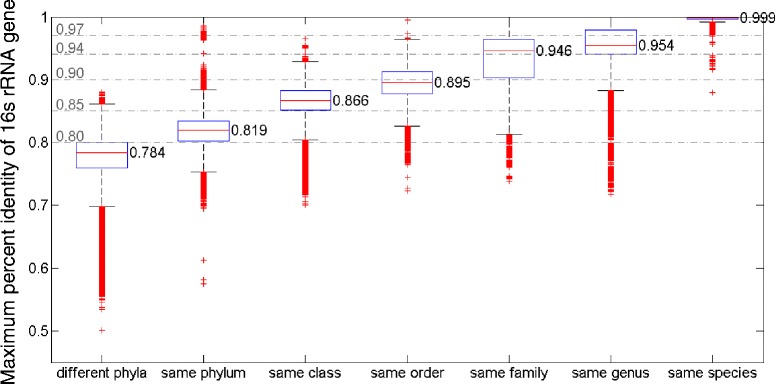
Box and whisker plot of 16s rRNA percent identities between genomes of the same taxa (species/genus/etc.). The *whiskers* represent boundaries within 1.5 interquartile range of the lower and upper quartiles. Although the 16s rRNA genes are more similar at finer taxonomic levels, there are still large overlaps between taxonomic classification levels and outliers within each level. Therefore, the 16s rRNA gene is sometimes an ambiguous marker in taxonomic classification

To systematically address this issue, we examined the relationship between the percent identity of the 16s rRNA gene and taxonomical relationship, according to NCBI classifications (Fig. 1). As expected, a general trend holds that higher 16s rRNA identity is indicative of closer taxonomic relationships. However, the inference of the level of taxonomic relatedness based on 16s percent identities is difficult, as the range of 16s identity overlaps between different taxonomic relationships. For example, sequences from the same species can be less than 94 % identical while sequences from different classes can be 97 % identical. It is of note though that due to missing taxa, inconsistent taxonomy, and the presence of numerous informal names, the fact that we use NCBI taxonomy may also contribute to the observed noisiness.

To overcome the limitations of the NCBI taxonomic classifications, we used an alternative approach for estimating the evolutionary distance between different prokaryotes. Namely, we calculated the average AAI between genomes using all homologous protein-coding genes they shared. Calculating AAI is computationally expensive and possible only for fully sequenced genomes. However, we assume that it is more accurate to estimate the evolutionary distance between two genomes based on all homologs, rather than based on a single locus such as the 16s rRNA gene. AAI likely does not provide a perfect estimation of relatedness, as it can also be somewhat affected by HGT. However, because it combines information across a very large number of loci, AAI likely provides us with the most reliable available estimation of the extent of relatedness of two genomes.

**Fig. 2:**
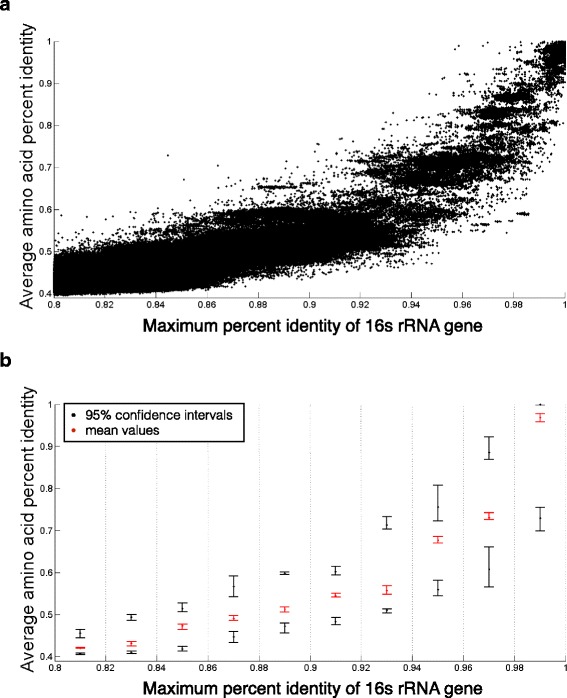
The 16s rRNA percent identity compared with the average AAI for all pairs of genomes (**a**) and with random samplings (**b**). The latter shows the 95 % confidence interval of AAI for each 2 % range of 16s rRNA percent identity, based on 20 random samplings of 100 genome pairs to assure that no genome was considered twice. While there is a general trend that higher 16s rRNA similarity indicates higher AAI, the range of AAI becomes broader for prokaryotes with more similar 16s rRNA sequences. For example, pairs of prokaryotes with 98 % 16s rRNA identity can have an AAI of anywhere between 50 and 100 %. The range of average AAI values for each bin is plotted in *red*, with the 95 % confidence interval upper and lower boundaries plotted in *black*

We compared every two genomes with a 16s identity higher than 80 %, and a total of 717,861 pairs of genomes were analyzed. In general, as expected, the percent identity of the 16s rRNA gene correlates well with AAI (rho = 0.7487 and *p* value ≪0.0001, according to a Spearman test, Fig. 2a). However, as the 16s rRNA genes get more similar, the range of AAI becomes broader (Fig. 2a). In other words, the percent identity of the 16s rRNA gene becomes more ambiguous at predicting AAI when genomes are more closely related.

The analysis described above was carried out by comparing all genomes to each other in a pairwise manner. Due to this, Fig. 2a might be greatly affected by individual genome outliers. For example, it is possible that one genome has mutated abnormally faster than other genomes of its species but retained the almost identical 16s rRNA gene. This may result in many data points with high 16s identity but low AAI, while the cause is merely one outlier genome. To reduce the impact of abnormal data points caused by individual outlier genomes, we performed 20 random samplings of 100 genome pairs and calculated the 95 % confidence intervals for AAI. In these samplings, a genome could only be selected once. This was done for each 16s rRNA gene identity level with an interval of 2 %. The result is shown in Fig. 2b, where the range of confidence interval increases as genomes become more similar. For example, genome pairs with 80–82 % 16s rRNA identity are 95 % likely to have an AAI between 40 and 46 %. In comparison, genome pairs with a 98–100 % 16s rRNA identity can have an AAI anywhere between 70 and 100 %. This again shows that 16s rRNA gene percent identities become more ambiguous predictors of AAI as genome pairs become more similar.

### When considering closely related prokaryotes, the percent identity of many marker genes better predicts AAI than the percent identity of 16s rRNA

The ambiguity of 16s rRNA in predicting genome-wide levels of similarity (as measured by AAI), especially for closely related prokaryotes, led to the question of whether other housekeeping genes might be more useful for this purpose. To find a potentially better marker gene, we started with the genes that were common to all genomes in the COG database and trimmed them down to 73 that were present in over 90 % of fully sequenced prokaryote genomes (“Methods” section, Additional file 1: Figure S1).

**Fig. 3:**
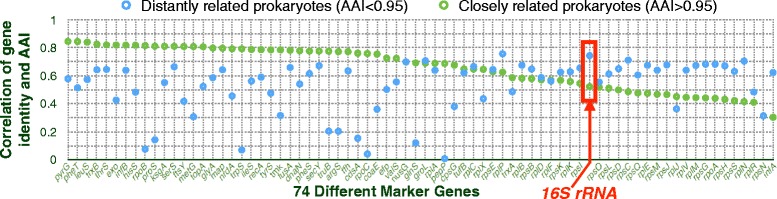
Spearman’s correlation between each marker gene and the average AAI for distantly related prokaryotes whose AAIs are lower than 95 % and closely related prokaryotes whose AAIs are greater than 95 %. Genes are ordered by decreasing correlation coefficient for closely related prokaryotes

Figure 3 shows the Spearman correlation between the percent identity of each marker gene and AAI, for genome pairs with AAI values lower or greater than 95 % (see Additional file 1: Figure S2 for Spearman’s correlation between each marker gene and AAI for all genomes altogether). For the more distantly related genomes with AAIs lower than 95 %, the 16s rRNA percent identity correlates very well with AAI. In fact, there is only one other marker gene (*rplP*) that yields a slightly better correlation than 16s rRNA for these genome pairs. However, when it comes to closely related genomes, with AAIs over 95 %, most markers outperform 16s rRNA (Fig. 3). Among the markers whose percent identities correlate better with AAI, for genomes with AAI over 95 %, is *rplP*. This means that *rplP* provides better correlations with AAI for both closely and distantly related genomes. It is interesting to note that *rpoB* and *recA* are among the many marker genes that outperform 16s rRNA for closely related genomes*.* This likely explains why these markers were previously shown to be more useful for classifying strains within specific lineages [19–21]. It is also worth noting the *groL* gene (often referred to as *groEL* or cpn60 (see Additional file 1: Table S2 for alternative names of surveyed marker genes)). Fitting with previous suggestions that *groL* may be a particularly well-performing marker [48, 49], we found that the percent identity of *groL* correlates equally well with AAI for both closely and distantly related prokaryotes (Fig. 3).

The better correlation with AAI observed for the percent identities of various marker genes suggests that some of them should be more useful than the 16s rRNA gene in classifying and separating strains within closely related lineages. While we cannot know the true species trees of any bacterial lineage, we again relied on an assumption that the best trees available to us will be generated by considering levels of similarity between all shared orthologs or, in other words, by considering AAI. We therefore reconstructed predicted species trees of three bacterial lineages, using AAI or each of the 74 marker genes including 16s rRNA. The three bacterial lineages for which we reconstructed such trees were *Escherichia*/*Shigella* (Additional file 1: Figure S3), *Streptococcus* (Additional file 1: Figure S4), and *Bacillus* (Additional file 1: Figure S5). The trees reconstructed based on each of the marker genes (including the 16s rRNA gene) were compared by how well they resemble the tree reconstructed based on AAI (Additional file 1: Table S3).

**Fig. 4:**
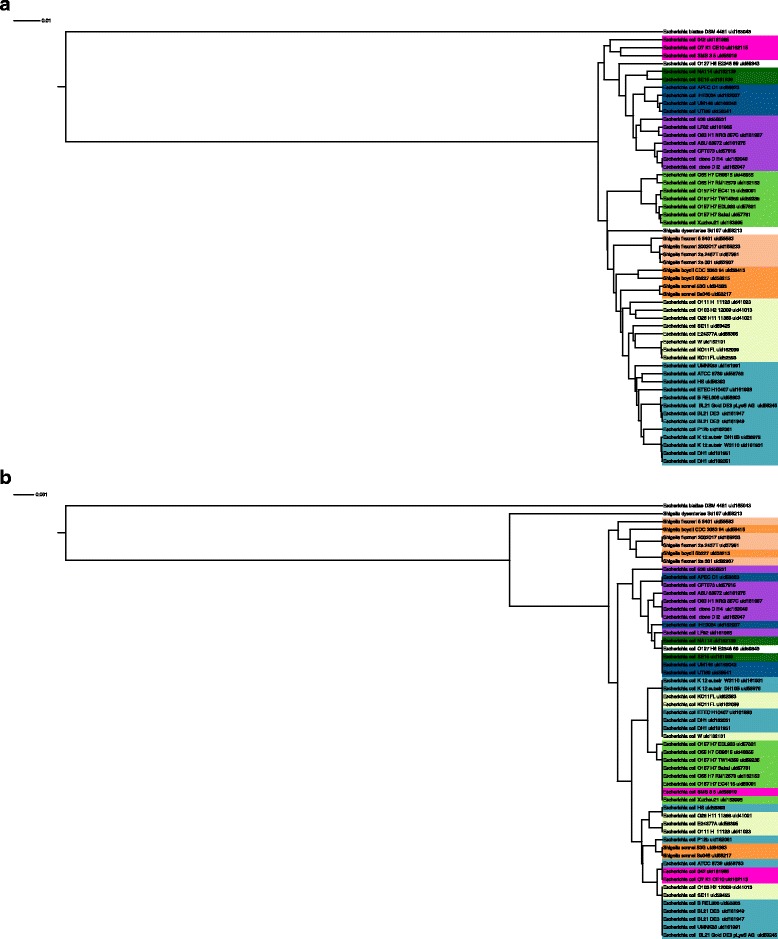
Trees of the *Escherichia*/*Shigella* lineage generated **a** based on AAI and **b** based on 16s rRNA percent identity. The latter fails to separate some major divisions of this lineage and poorly reconstitutes the genome-wise similarity relationship

Manual examination of the AAI-generated trees and of trees generated using 16s rRNA results in a number of examples in which the tree generated using 16s rRNA does not provide accurate separation between strains. For example, the *Escherichia*/*Shigella* tree generated based on AAI (Fig. 4a) well separates the *E. coli O157*:*H7* strains and *E. coli O55*:*H7* strains, which were mixed in the tree generated from 16s rRNA identities (Fig. 4b). In addition, the 16s tree failed to show an immediate common ancestor for *Shigella dysenteriae* and *Escherichia coli O157*:*H7* [50], while this is clearly seen in the AAI tree. In contrast, the trees generated based on several marker genes were able to capture these separations (Additional file 1: Figure S3). Similar results can also be seen for the *Streptococcus* and *Bacillus* lineages (Additional file 1: Figures S4 and S5).

To quantify the usefulness of the marker genes in reconstructing similar relationships between strains to those observed using genome-wide information, we computed the split distance between each marker gene tree and the AAI tree (Additional file 1: Table S3, “Methods” section). For all three lineages, many marker genes outperformed the 16s rRNA gene. In fact, out of 74 marker genes (including 16s rRNA), the 16s rRNA gene was only the 48th, 64th, and 31st best-performing gene in reconstructing the AAI tree of *Escherichia*/*Shigella*, *Bacillus*, and *Streptococcus*, respectively. The top 10 marker genes that best reconstructed each lineage are listed in Table 1. The genes that were most useful for reconstruction of the AAI-based tree vary between lineages, suggesting that marker genes need to be individually selected for each specific lineage of interest.

**Table 1:** The identity of the top 10 best performing markers differs between the three examined lineages

*Escherichia*/*Shigella*	*Streptococcus*	*Bacillus*
*argS*	*nrdA*	*cdsA*
*glnS*	*serS*	*glyA*
*ileS*	*dnaN*	*hisS*
*valS*	*cpsG*	*leuS*
*infB*	*fusA*	*pheT*
*fusA*	*proS*	*tmk*
*topA*	*pyrG*	*infB*
*Ffh*	*tyrS*	*ksgA*
*proS*	*ftsY*	*pheS*
*Tmk*	*glnS*	*topA*

### Marker genes that are least conserved in their sequences within a lineage can be used to generate trees most similar to that inferred using AAI within that lineage

**Fig. 5:**
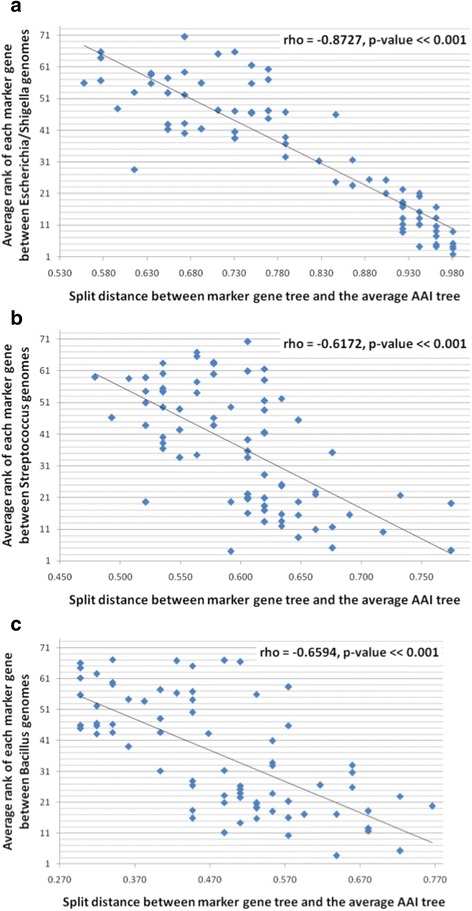
Trees generated using less sequence-conserved marker genes better reconstitute the tree generated using AAI, for three lineages: **a** *Escherichia*/*Shigella*, **b** *Streptococcus*, and **c** *Bacillus*. The percent identity rank of each gene represents its relative sequence conservation within the lineage. The split distance represents the resemblance between the tree generated using each marker gene and the tree generated using AAI. Spearman correlation coefficients and *p* values are shown, and a *linear trend line* is drawn that minimizes the squared error

The above finding that different marker genes seem to be most useful for reconstructing the AAI-based tree of different bacterial lineages raises the question of how to choose the correct marker genes for examining a lineage of interest. We hypothesized that genes that are less conserved in their sequences within a lineage should be more capable of capturing the evolutionary differences between its members and therefore should allow for the reconstruction of better trees. To test this hypothesis, we ranked the different marker genes according to their percent identities within the three examined lineages (“Methods” section). Genes with higher ranks (closer to 74) have lower percent identities (are less conserved in their sequences). At the same time, genes with lower ranks (closer to 1) have higher levels of sequence conservation. As is shown in Fig. 5, the rank of genes strongly correlates with their AAI-based tree reconstruction performance, for the three lineages examined (*p* values ≪0.001 according to the Spearman correlation test). This means that marker genes that are the least conserved in their sequences within each lineage can be used to better reconstruct the AAI-based tree for the given lineage.

**Fig. 6:**
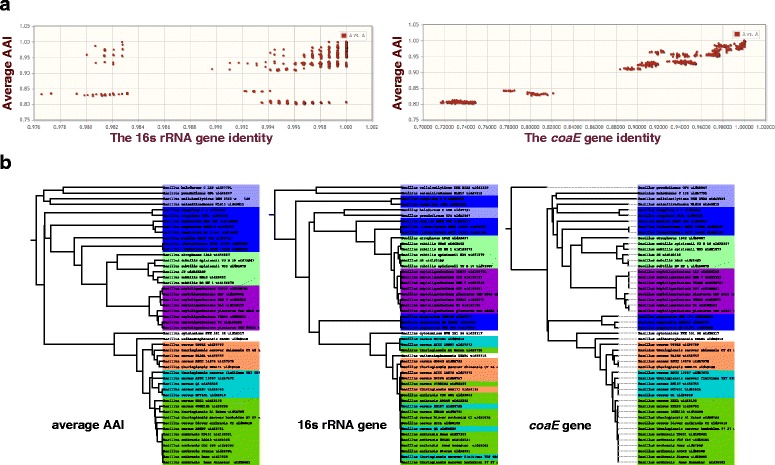
Comparison of the 16s rRNA gene and the *coaE* gene within the *Bacillus* lineage, for **a** the gene identity of 16s rRNA or *coaE* vs. the average AAI and **b** tree reconstructed from 16s rRNA or *coaE* gene in comparison with the tree reconstructed from AAI. As the least sequence-conserved marker gene within the *Bacillus* lineage, *coaE* correlates well with the AAI in percent identities and is able to reconstruct the tree resembling that reconstructed from AAI

An example of this trend can be seen when considering the marker gene with the lowest levels of sequence conservation for the *Bacillus* lineage, *coaE.* The correlation observed between AAI and the percent identity of *coaE* is much better than that observed between AAI and the percent identity of the 16s rRNA gene (Fig. 6a). Furthermore, *coaE* reconstructs a tree that is much more similar to the one obtained using AAI (Fig. 6b). Specifically, when we used the 16s rRNA gene to reconstruct the *Bacillus* tree, we could not distinguish between closely related genomes. However, when we used *coaE*, such closely related genomes could be clustered as they were in the AAI-derived tree (Fig. 6b).

It was previously suggested that *Bacillus anthracis* (the causative agent of anthrax), *Bacillus cereus*, and *Bacillus thuringiensis* are genetically one species in spite of their widely different phenotypes and pathogenic effects. In agreement with previous studies into *Bacillus* [51], the trees we reconstruct for *Bacillus* using AAI or *coaE* both show that *Bacillus* has two major clades, with *B. anthracis* forming a monophyletic branch in one of the clades (Fig. 6b). In contrast, the 16s rRNA reconstructed tree does not capture the two-clade division and also does not capture the monophyletic branch of *B. anthracis*. This is noteworthy because it demonstrates that by using marker genes, individually chosen to best separate particular lineages of interest, we may in some cases be able to separate pathogens (e.g., anthrax) from less pathogenic members of their lineage.

Combined, our results suggest that the relative levels of sequence conservation of marker genes provide a useful metric for the selection of the best markers to use to infer degrees of genome-wide similarity within specific lineages of interest.

## Conclusions

The 16s rRNA gene was used in many microbiome studies in which it was demonstrated that fluctuations in microbiome structure correlate with ailments such as obesity [52, 53], caries and periodontal disease [54, 55], gastrointestinal disease [56, 57], and urinary tract infections [58]. At the same time, as we show here and others have shown before us, 16s rRNA is not very useful when it comes to inferring the degree of genome-wide similarity of closely related prokaryotic strains. Understanding whether and how fluctuations in specific species abundances contribute to the observed correlations may thus require separation of strains at higher resolution than possible using 16s rRNA. For example, in microbiome studies that aim to separate certain members from a lineage such as the pathogenic ones, or revisit a classification scheme, a marker that can better distinguish between closely related genomes may be required.

Here, we present an analysis quantifying the extent to which 16s rRNA similarity predicts genome-wide similarity between both closely and distantly related prokaryotes. We showed that 16s rRNA is one of the best marker genes for inferring genome-wide gene sequence similarity (as estimated using AAI) for distantly related genomes. At the same time, many marker genes can be more useful than 16s rRNA, when it comes to estimating genome-wide levels of gene sequence similarity, for closely related prokaryotes. Finally, we show that the marker genes that are least conserved in their sequences within a lineage of interest are the most useful in inferring trees that resemble those constructed based on genome-wide similarity information (as measured using AAI).

We show that less sequence-conserved markers are less useful than 16s rRNA and other more sequence-conserved markers for inferring genome-wide levels of gene sequence similarity between distantly related prokaryotes. At the same time, less sequence-conserved markers are more useful for inferring similarity of closely related prokaryotes. Why is this the case? Less conserved genes are ones that have, per definition, acquired more changes in their sequences. These changes can include indels and multiple hits at the same exact sites, which will make alignments and subsequent quantification of levels of similarity less reliable at larger evolutionary distances. For such distantly related genomes, levels of similarity between more sequence-conserved markers, such as 16s rRNA, will result in better inference of genome-wide levels of similarity. At the same time, the fact that less sequence-conserved markers accumulate more substitutions gives them more power to detect differences between closely related strains. This makes them more useful for predicting genome-wide similarity at short distances.

Prokaryotic genomes tend to undergo substantial horizontal gene transfer (HGT). Such HGT can greatly dilute any signal pertaining to the relationship between strains and lead to false inferences. This is true no matter what method of inference is used, be it 16s rRNA-based, based on other markers, or even based on whole microbiome or whole genome data (such as AAI). While whole genome (AAI)-based classification can therefore also be quite noisy, it nevertheless provides us with the best possible inference of the extent of similarity between genomes, since they utilize data from all available loci, rather than from a specific gene alone. Using data from entire genomes is not, however, always feasible, especially not in microbiome studies in which most bacteria cannot be cultured. Shotgun metagenome studies allow one to combine information from large number of loci, but in a much more complex way, since rarely will multiple loci be sequenced from exactly the same strain of prokaryote. Additionally, such studies are often beyond the budget of many researchers. This is why 16s rRNA has become so useful for microbiome studies, and this is also why other marker genes may be useful. However, a concern was raised that markers other than 16s rRNA may be less useful, because they may undergo more HGT than 16s rRNA [28]. Here we show that even under the potential impact of HGT, some less sequence-conserved markers outperform 16s rRNA, in predicting genome-wide levels of gene similarity (as estimated using genome-wide AAI data), for closely related bacterial lineages. This in turn may suggest that as a group, these markers do not undergo more HGT than 16s rRNA within the lineages in which they outperform 16s rRNA.

We find that markers that provide the best resolution, for a specific lineage, are those that are least conserved in their sequences within that lineage. Therefore, less sequence-conserved markers can be chosen for specific lineages of interest that would provide more accurate reconstruction of the diversity in ecosystems and allow for higher resolution microbiome studies. In order to facilitate easy marker choice, we have built the POGO database (Database of Pairwise-comparisons Of Genomes and conserved Orthologous genes) [59]. POGO-DB allows users to rank markers according to their relative levels of sequence conservation within a lineage of interest and also allows users to compare the percent identity of different markers to AAI for the same lineage. Therefore, users can use POGO-DB to verify whether the 16s rRNA gene has good correlation to the AAI within their lineages of interest or to select the best alternative markers for the lineages in which they are interested, in an informed manner.

An advantage of using 16s rRNA has been that its conservation is such that universal primers can be designed to amplify its sequence across all prokaryotes. This is likely not possible for most other marker genes and especially not for the ones that are least conserved in their sequences. However, as we demonstrate, the less sequence-conserved marker genes are most useful for inferring finer grained strain differences for particular lineages of interest. Therefore, in order to utilize these markers, where they are most useful, it is only necessary to design primers that can recognize areas of the marker genes’ sequences that are conserved within a lineage of interest. To demonstrate the feasibility of designing such primers, we designed 10 primer sets for the 10 marker genes that are least conserved in their sequences in the *Escherichia*/*Shigella* lineage (Additional file 1: Table S4). These primer sets were designed to allow for the amplification of these 10 markers across all sequenced *Escherichia* and *Shigella* strains. For 7 of the 10 markers, the designed primers enable the amplification of over 70 % of the full gene length. In order to assist researchers in designing lineage-specific primers for markers of interest, we have added a feature to POGO-DB that allows users to download the sequences of a marker gene from a list of specified genomes. These sequences can then be aligned to identify regions that are conserved within the lineage of interest and that can be used to design primers that will amplify the gene within that lineage.

### Availability of data and materials

The data set supporting the results of this article is available in the POGO database, http://pogo.ece.drexel.edu.

## Additional file

Additional supplementary information may be found in the online version of this article. Additional file 1: Figure S1. The number of genomes in which each marker gene is identified. Out of the 79 potential marker genes, 73 are present in at least 90 % of the genomes. **Figure S2.** Spearman’s correlation between each marker gene and the average AAI for all complete genomes. Genes are ordered in the same way as in Fig. 3. **Figure S3.** Trees generated based on AAI and on percent identities of each marker gene (including 16s rRNA), for the *Escherichia*/*Shigella* clade. **Figure S4.** Trees generated based on AAI and on percent identities of each marker gene (including 16s rRNA), for the *Streptococcus* clade. **Figure S5.** Trees generated based on AAI and on percent identities of each marker gene (including 16s rRNA), for the *Bacillus* clade. **Table S1.** List of 79 potential marker genes surveyed, out of which 73 were found to be present in at least 90 % of the genomes. **Table S2.** Alternative names of 79 potential marker genes surveyed. **Table S3.** Split distances between UPGMA tree generated using AAI and that generated using the percent identities of each marker gene, shown in correspondence with the average percent identity ranks of the marker genes. **Table S4.** Designed primers for each of the 10 genes that were least conserved in their sequences in the *Escherichia*/*Shigella* lineage. (ZIP 3355 kb)

## Abbreviations

AAI: amino acid identity
HGT: horizontal gene transfer
UPGMA: Unweighted Pair Group Method with Arithmetic Mean
