# Supplementary material for: Marker genes that are less conserved in their sequences are useful for predicting genome-wide similarity levels between closely related prokaryotic strains
Source: Microbiome. 2016 May 3;4:18. doi: 10.1186/s40168-016-0162-5 (PMC4853863; doi:10.1186/s40168-016-0162-5)
Supplement: Additional file 1: Figure S1. — The number of genomes in which each marker gene is identified. Out of the 79 potential marker genes, 73 are present in at least 90 % of the genomes. Figure S2. Spearman’s correlation between each marker gene and the average AAI for all complete genomes. Genes are ordered in the same way as in Fig. 3. Figure S3. Trees generated based on AAI and on percent identities of each marker gene (including 16s rRNA), for the Escherichia/Shigella clade. Figure S4. Trees generated based on AAI and on percent identities of each marker gene (including 16s rRNA), for the Streptococcus clade. Figure S5. Trees generated based on AAI and on percent identities of each marker gene (including 16s rRNA), for the Bacillus clade. Table S1. List of 79 potential marker genes surveyed, out of which 73 were found to be present in at least 90 % of the genomes. Table S2. Alternative names of 79 potential marker genes surveyed. Table S3. Split distances between UPGMA tree generated using AAI and that generated using the percent identities of each marker gene, shown in correspondence with the average percent identity ranks of the marker genes. Table S4. Designed primers for each of the 10 genes that were least conserved in their sequences in the Escherichia/Shigella lineage. (ZIP 3355 kb) [file 40168_2016_162_MOESM1_ESM.zip › FigureS3.pdf]

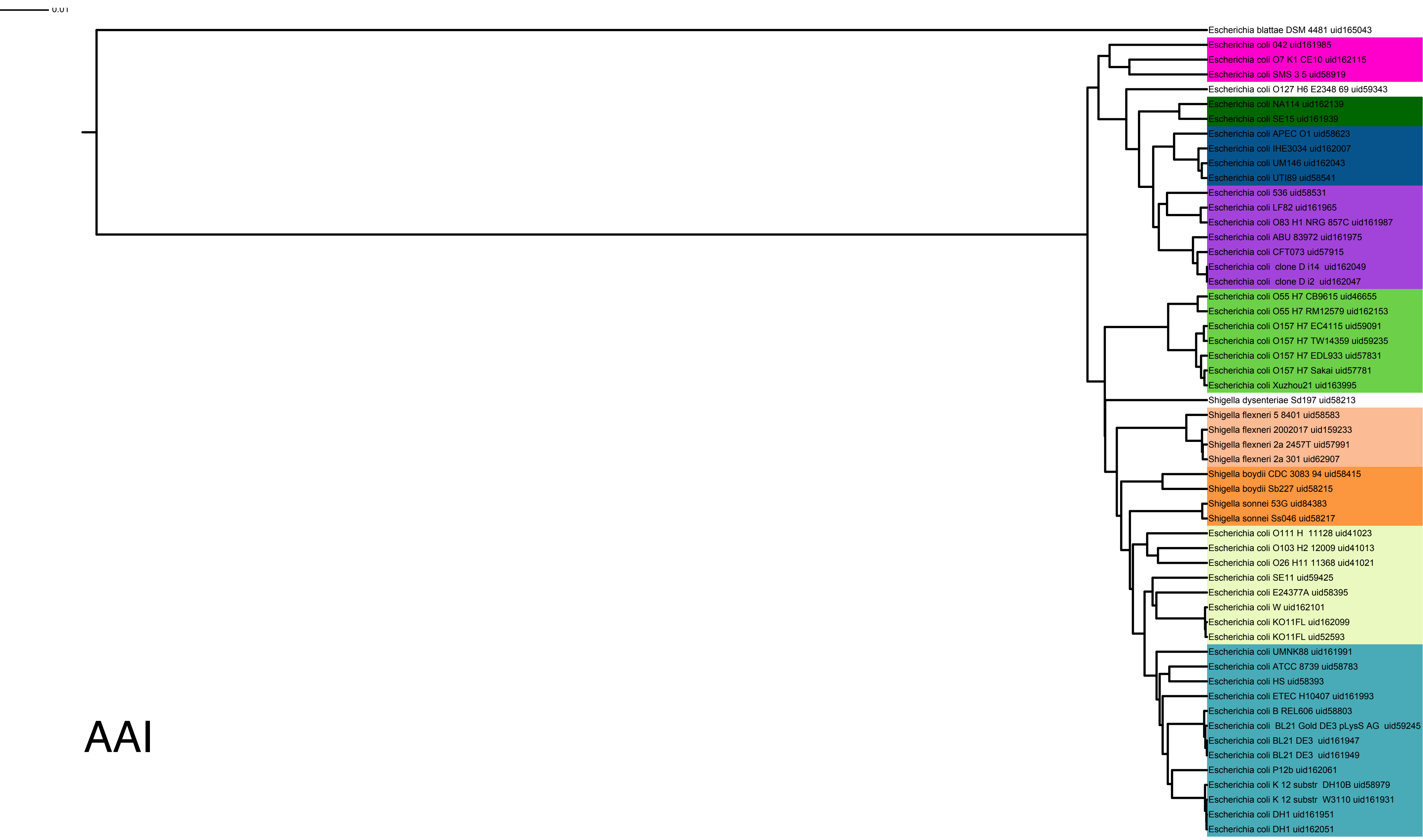
0.01
Escherichia blattae DSM 4481 uid165043
Escherichia coli 042 uid161985
Escherichia coli O7 K1 CE10 uid162115
Escherichia coli SMS 3 5 uid58919
Escherichia coli O127 H6 E2348 69 uid59343
Escherichia coli NA114 uid162139
Escherichia coli SE15 uid161939
Escherichia coli APEC O1 uid58623
Escherichia coli IHE3034 uid162007
Escherichia coli UM146 uid162043
Escherichia coli UTI89 uid58541
Escherichia coli 536 uid58531
Escherichia coli LF82 uid161965
Escherichia coli O83 H1 NRG 857C uid161987
Escherichia coli ABU 83972 uid161975
Escherichia coli CFT073 uid57915
Escherichia coli clone D i14 uid162049
Escherichia coli clone D i2 uid162047
Escherichia coli O55 H7 CB9615 uid46655
Escherichia coli O55 H7 RM12579 uid162153
Escherichia coli O157 H7 EC4115 uid59091
Escherichia coli O157 H7 TW14359 uid59235
Escherichia coli O157 H7 EDL933 uid57831
Escherichia coli O157 H7 Sakai uid57781
Escherichia coli Xuzhou21 uid163995
Shigella dysenteriae Sd197 uid58213
Shigella flexneri 5 8401 uid58583
Shigella flexneri 2002017 uid159233
Shigella flexneri 2a 2457T uid57991
Shigella flexneri 2a 301 uid62907
Shigella boydii CDC 3083 94 uid58415
Shigella boydii Sb227 uid58215
Shigella sonnei 53G uid84383
Shigella sonnei Ss046 uid58217
Escherichia coli O111 H 11128 uid41023
Escherichia coli O103 H2 12009 uid41013
Escherichia coli O26 H11 11368 uid41021
Escherichia coli SE11 uid59425
Escherichia coli E24377A uid58395
Escherichia coli W uid162101
Escherichia coli KO11FL uid162099
Escherichia coli KO11FL uid52593
Escherichia coli UMNK88 uid161991
Escherichia coli ATCC 8739 uid58783
Escherichia coli HS uid58393
Escherichia coli ETEC H10407 uid161993
Escherichia coli B REL606 uid58803
Escherichia coli BL21 Gold DE3 pLysS AG uid59245
Escherichia coli BL21 DE3 uid161947
Escherichia coli BL21 DE3 uid161949
Escherichia coli P12b uid162061
Escherichia coli K 12 substr DH10B uid58979
Escherichia coli K 12 substr W3110 uid161931
Escherichia coli DH1 uid161951
Escherichia coli DH1 uid162051
AAI

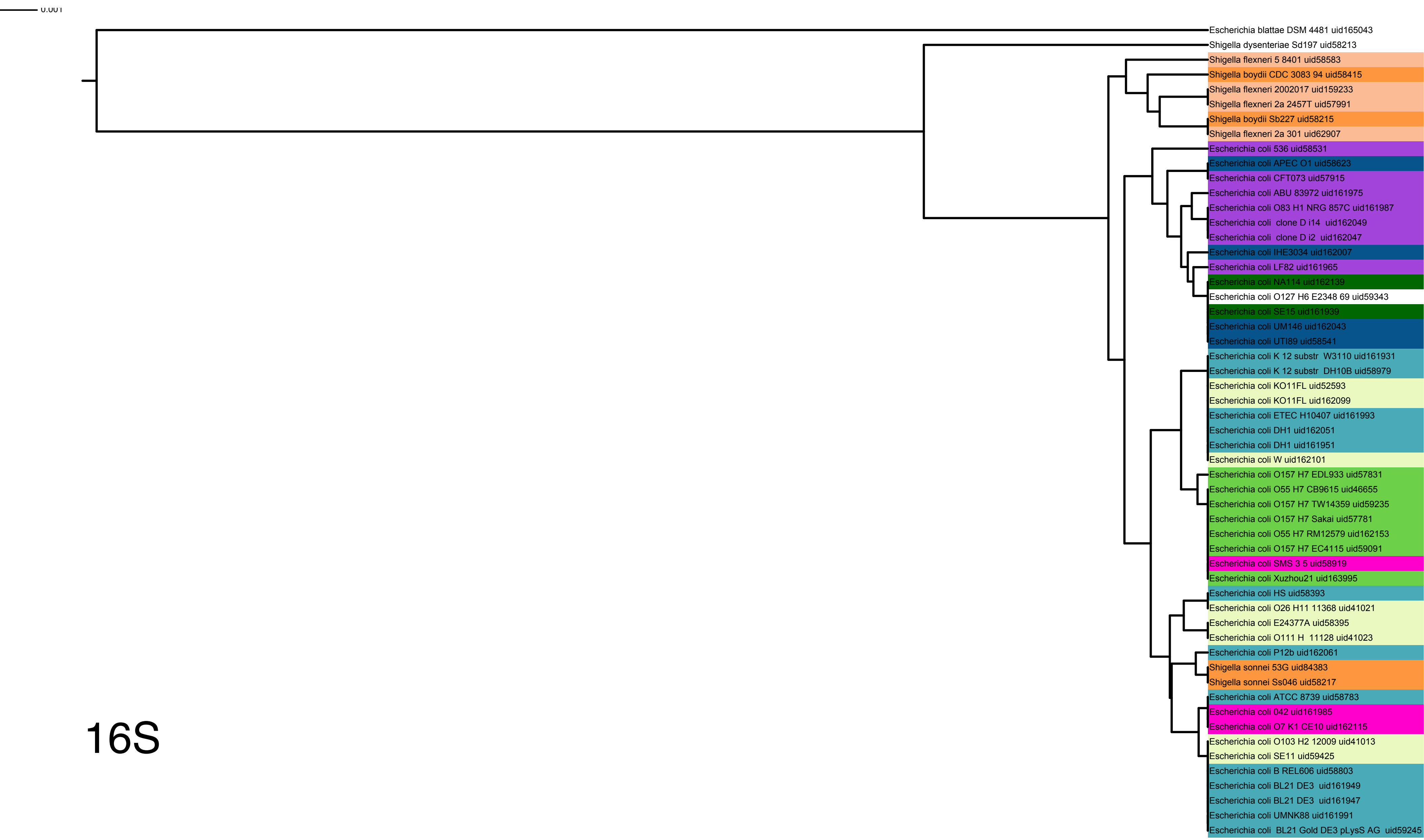
0.001
Escherichia blattae DSM 4481 uid165043
Shigella dysenteriae Sd197 uid58213
Shigella flexneri 5 8401 uid58583
Shigella boydii CDC 3083 94 uid58415
Shigella flexneri 2002017 uid159233
Shigella flexneri 2a 2457T uid57991
Shigella boydii Sb227 uid58215
Shigella flexneri 2a 301 uid62907
Escherichia coli 536 uid58531
Escherichia coli APEC O1 uid58623
Escherichia coli CFT073 uid57915
Escherichia coli ABU 83972 uid161975
Escherichia coli O83 H1 NRG 857C uid161987
Escherichia coli clone D i14 uid162049
Escherichia coli clone D i2 uid162047
Escherichia coli IHE3034 uid162007
Escherichia coli LF82 uid161965
Escherichia coli NA114 uid162139
Escherichia coli O127 H6 E2348 69 uid59343
Escherichia coli SE15 uid161939
Escherichia coli UM146 uid162043
Escherichia coli UTI89 uid58541
Escherichia coli K 12 substr W3110 uid161931
Escherichia coli K 12 substr DH10B uid58979
Escherichia coli KO11FL uid52593
Escherichia coli KO11FL uid162099
Escherichia coli ETEC H10407 uid161993
Escherichia coli DH1 uid162051
Escherichia coli DH1 uid161951
Escherichia coli W uid162101
Escherichia coli O157 H7 EDL933 uid57831
Escherichia coli O55 H7 CB9615 uid46655
Escherichia coli O157 H7 TW14359 uid59235
Escherichia coli O157 H7 Sakai uid57781
Escherichia coli O55 H7 RM12579 uid162153
Escherichia coli O157 H7 EC4115 uid59091
Escherichia coli SMS 3 5 uid58919
Escherichia coli Xuzhou21 uid163995
Escherichia coli HS uid58393
Escherichia coli O26 H11 11368 uid41021
Escherichia coli E24377A uid58395
Escherichia coli O111 H 11128 uid41023
Escherichia coli P12b uid162061
Shigella sonnei 53G uid84383
Shigella sonnei Ss046 uid58217
Escherichia coli ATCC 8739 uid58783
Escherichia coli 042 uid161985
Escherichia coli O7 K1 CE10 uid162115
Escherichia coli O103 H2 12009 uid41013
Escherichia coli SE11 uid59425
Escherichia coli B REL606 uid58803
Escherichia coli BL21 DE3 uid161949
Escherichia coli BL21 DE3 uid161947
Escherichia coli UMNK88 uid161991
Escherichia coli BL21 Gold DE3 pLysS AG uid59245
16S

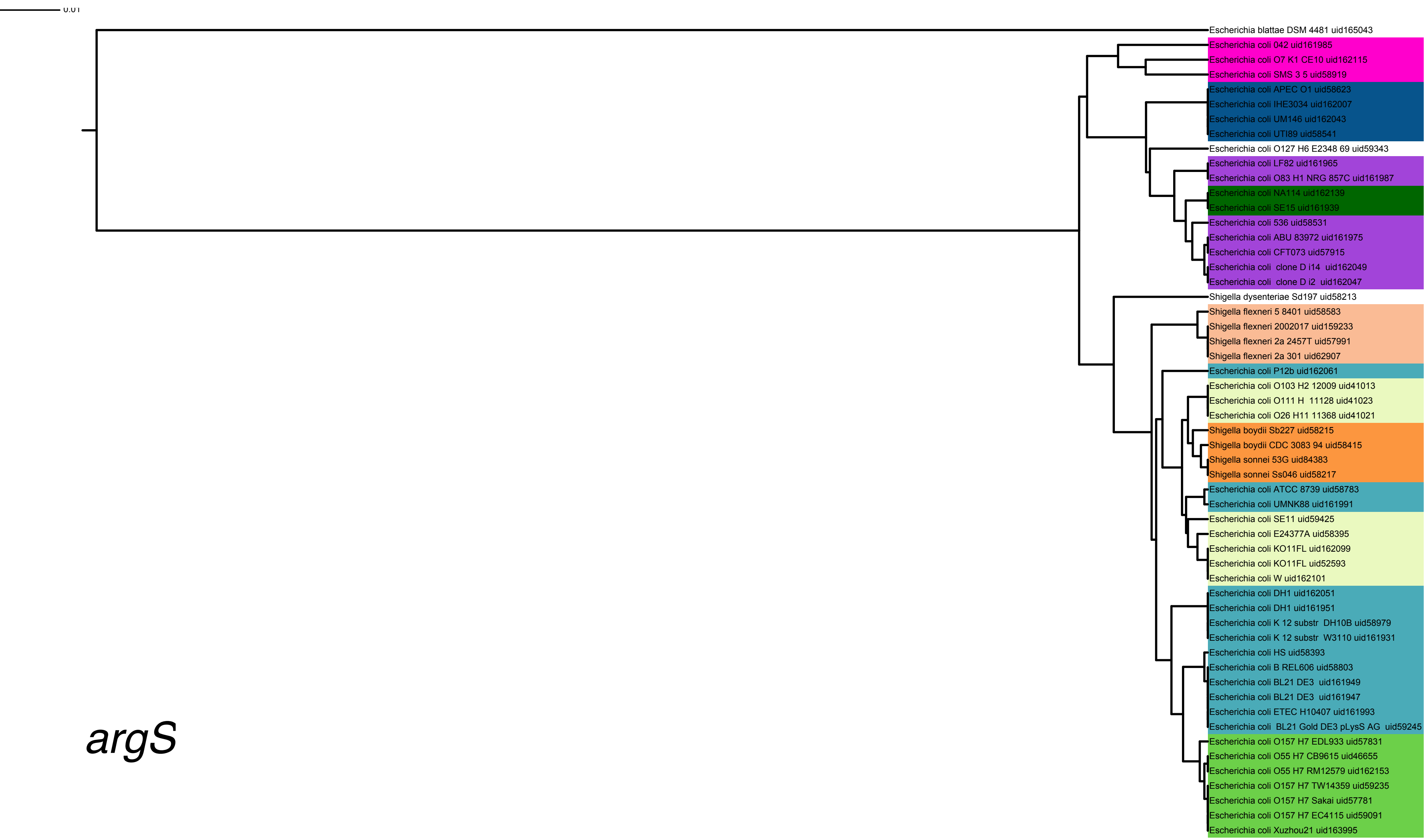
0.01
Escherichia blattae DSM 4481 uid165043
Escherichia coli 042 uid161985
Escherichia coli O7 K1 CE10 uid162115
Escherichia coli SMS 3 5 uid58919
Escherichia coli APEC O1 uid58623
Escherichia coli IHE3034 uid162007
Escherichia coli UM146 uid162043
Escherichia coli UTI89 uid58541
Escherichia coli O127 H6 E2348 69 uid59343
Escherichia coli LF82 uid161965
Escherichia coli O83 H1 NRG 857C uid161987
Escherichia coli NA114 uid162139
Escherichia coli SE15 uid161939
Escherichia coli 536 uid58531
Escherichia coli ABU 83972 uid161975
Escherichia coli CFT073 uid57915
Escherichia coli clone D i14 uid162049
Escherichia coli clone D i2 uid162047
Shigella dysenteriae Sd197 uid58213
Shigella flexneri 5 8401 uid58583
Shigella flexneri 2002017 uid159233
Shigella flexneri 2a 2457T uid57991
Shigella flexneri 2a 301 uid62907
Escherichia coli P12b uid162061
Escherichia coli O103 H2 12009 uid41013
Escherichia coli O111 H 11128 uid41023
Escherichia coli O26 H11 11368 uid41021
Shigella boydii Sb227 uid58215
Shigella boydii CDC 3083 94 uid58415
Shigella sonnei 53G uid84383
Shigella sonnei Ss046 uid58217
Escherichia coli ATCC 8739 uid58783
Escherichia coli UMNK88 uid161991
Escherichia coli SE11 uid59425
Escherichia coli E24377A uid58395
Escherichia coli KO11FL uid162099
Escherichia coli KO11FL uid52593
Escherichia coli W uid162101
Escherichia coli DH1 uid162051
Escherichia coli DH1 uid161951
Escherichia coli K 12 substr DH10B uid58979
Escherichia coli K 12 substr W3110 uid161931
Escherichia coli HS uid58393
Escherichia coli B REL606 uid58803
Escherichia coli BL21 DE3 uid161949
Escherichia coli BL21 DE3 uid161947
Escherichia coli ETEC H10407 uid161993
Escherichia coli BL21 Gold DE3 pLysS AG uid59245
Escherichia coli O157 H7 EDL933 uid57831
Escherichia coli O55 H7 CB9615 uid46655
Escherichia coli O55 H7 RM12579 uid162153
Escherichia coli O157 H7 TW14359 uid59235
Escherichia coli O157 H7 Sakai uid57781
Escherichia coli O157 H7 EC4115 uid59091
Escherichia coli Xuzhou21 uid163995

argS

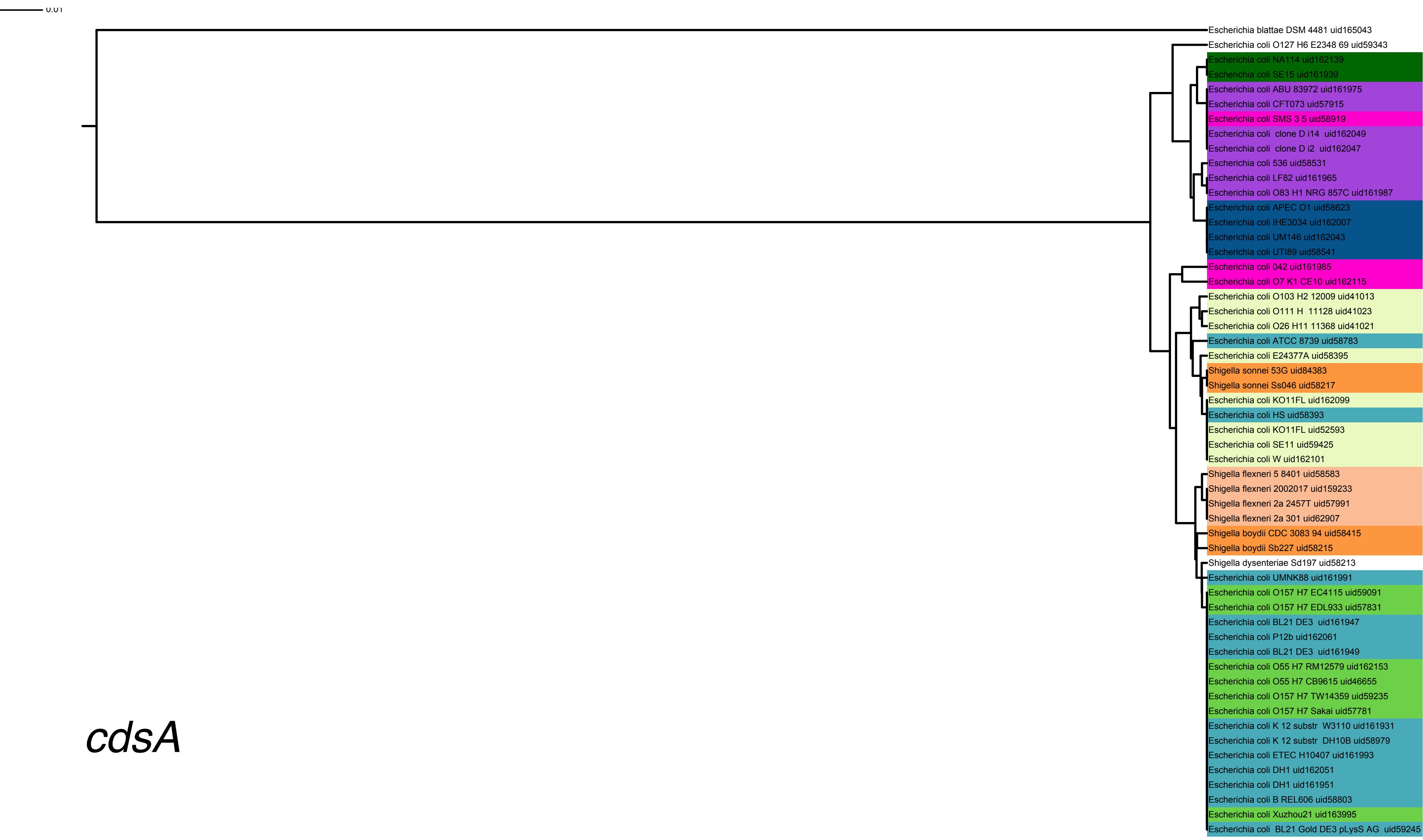

0.01
Escherichia blattae DSM 4481 uid165043
Escherichia coli O127 H6 E2348 69 uid59343
Escherichia coli NA114 uid162139
Escherichia coli SE15 uid161939
Escherichia coli ABU 83972 uid161975
Escherichia coli CFT073 uid57915
Escherichia coli SMS 3 5 uid58919
Escherichia coli clone D i14 uid162049
Escherichia coli clone D i2 uid162047
Escherichia coli 536 uid58531
Escherichia coli LF82 uid161965
Escherichia coli O83 H1 NRG 857C uid161987
Escherichia coli APEC O1 uid58623
Escherichia coli IHE3034 uid162007
Escherichia coli UM146 uid162043
Escherichia coli UTI89 uid58541
Escherichia coli 042 uid161985
Escherichia coli O7 K1 CE10 uid162115
Escherichia coli O103 H2 12009 uid41013
Escherichia coli O111 H 11128 uid41023
Escherichia coli O26 H11 11368 uid41021
Escherichia coli ATCC 8739 uid58783
Escherichia coli E24377A uid58395
Shigella sonnei 53G uid84383
Shigella sonnei Ss046 uid58217
Escherichia coli KO11FL uid162099
Escherichia coli HS uid58393
Escherichia coli KO11FL uid52593
Escherichia coli SE11 uid59425
Escherichia coli W uid162101
Shigella flexneri 5 8401 uid58583
Shigella flexneri 2002017 uid159233
Shigella flexneri 2a 2457T uid57991
Shigella flexneri 2a 301 uid62907
Shigella boydii CDC 3083 94 uid58415
Shigella boydii Sb227 uid58215
Shigella dysenteriae Sd197 uid58213
Escherichia coli UMNK88 uid161991
Escherichia coli O157 H7 EC4115 uid59091
Escherichia coli O157 H7 EDL933 uid57831
Escherichia coli BL21 DE3 uid161947
Escherichia coli P12b uid162061
Escherichia coli BL21 DE3 uid161949
Escherichia coli O55 H7 RM12579 uid162153
Escherichia coli O55 H7 CB9615 uid46655
Escherichia coli O157 H7 TW14359 uid59235
Escherichia coli O157 H7 Sakai uid57781
Escherichia coli K 12 substr W3110 uid161931
Escherichia coli K 12 substr DH10B uid58979
Escherichia coli ETEC H10407 uid161993
Escherichia coli DH1 uid162051
Escherichia coli DH1 uid161951
Escherichia coli B REL606 uid58803
Escherichia coli Xuzhou21 uid163995
Escherichia coli BL21 Gold DE3 pLysS AG uid59245
cdsA

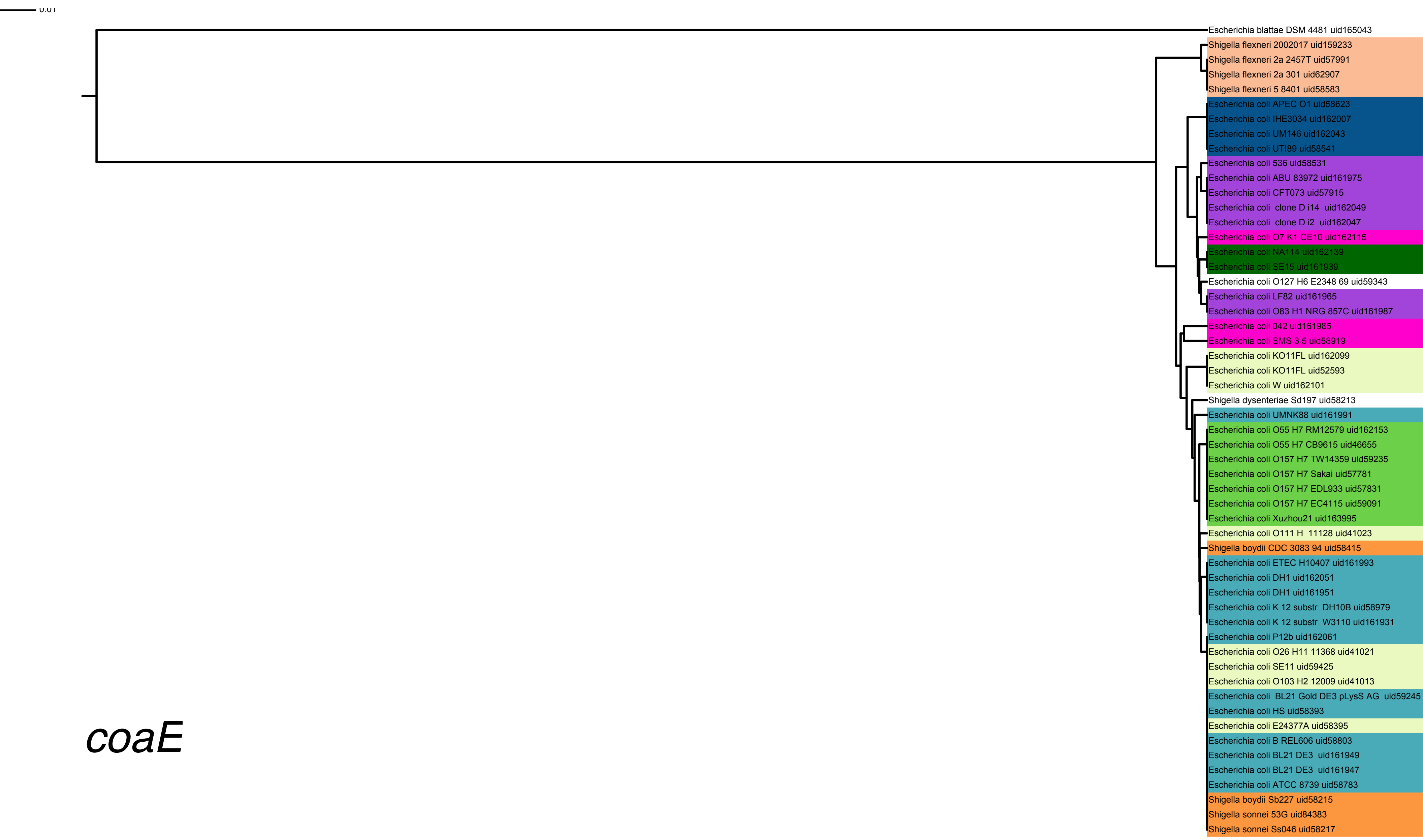
0.01
Escherichia blattae DSM 4481 uid165043
Shigella flexneri 2002017 uid159233
Shigella flexneri 2a 2457T uid57991
Shigella flexneri 2a 301 uid62907
Shigella flexneri 5 8401 uid58583
Escherichia coli APEC O1 uid58623
Escherichia coli IHE3034 uid162007
Escherichia coli UM146 uid162043
Escherichia coli UTI89 uid58541
Escherichia coli 536 uid58531
Escherichia coli ABU 83972 uid161975
Escherichia coli CFT073 uid57915
Escherichia coli clone D i14 uid162049
Escherichia coli clone D i2 uid162047
Escherichia coli O7 K1 CE10 uid162115
Escherichia coli NA114 uid162139
Escherichia coli SE15 uid161939
Escherichia coli O127 H6 E2348 69 uid59343
Escherichia coli LF82 uid161965
Escherichia coli O83 H1 NRG 857C uid161987
Escherichia coli 042 uid161985
Escherichia coli SMS 3 5 uid58919
Escherichia coli KO11FL uid162099
Escherichia coli KO11FL uid52593
Escherichia coli W uid162101
Shigella dysenteriae Sd197 uid58213
Escherichia coli UMNK88 uid161991
Escherichia coli O55 H7 RM12579 uid162153
Escherichia coli O55 H7 CB9615 uid46655
Escherichia coli O157 H7 TW14359 uid59235
Escherichia coli O157 H7 Sakai uid57781
Escherichia coli O157 H7 EDL933 uid57831
Escherichia coli O157 H7 EC4115 uid59091
Escherichia coli Xuzhou21 uid163995
Escherichia coli O111 H 11128 uid41023
Shigella boydii CDC 3083 94 uid58415
Escherichia coli ETEC H10407 uid161993
Escherichia coli DH1 uid162051
Escherichia coli DH1 uid161951
Escherichia coli K 12 substr DH10B uid58979
Escherichia coli K 12 substr W3110 uid161931
Escherichia coli P12b uid162061
Escherichia coli O26 H11 11368 uid41021
Escherichia coli SE11 uid59425
Escherichia coli O103 H2 12009 uid41013
Escherichia coli BL21 Gold DE3 pLysS AG uid59245
Escherichia coli HS uid58393
Escherichia coli E24377A uid58395
Escherichia coli B REL606 uid58803
Escherichia coli BL21 DE3 uid161949
Escherichia coli BL21 DE3 uid161947
Escherichia coli ATCC 8739 uid58783
Shigella boydii Sb227 uid58215
Shigella sonnei 53G uid84383
Shigella sonnei Ss046 uid58217
coaE

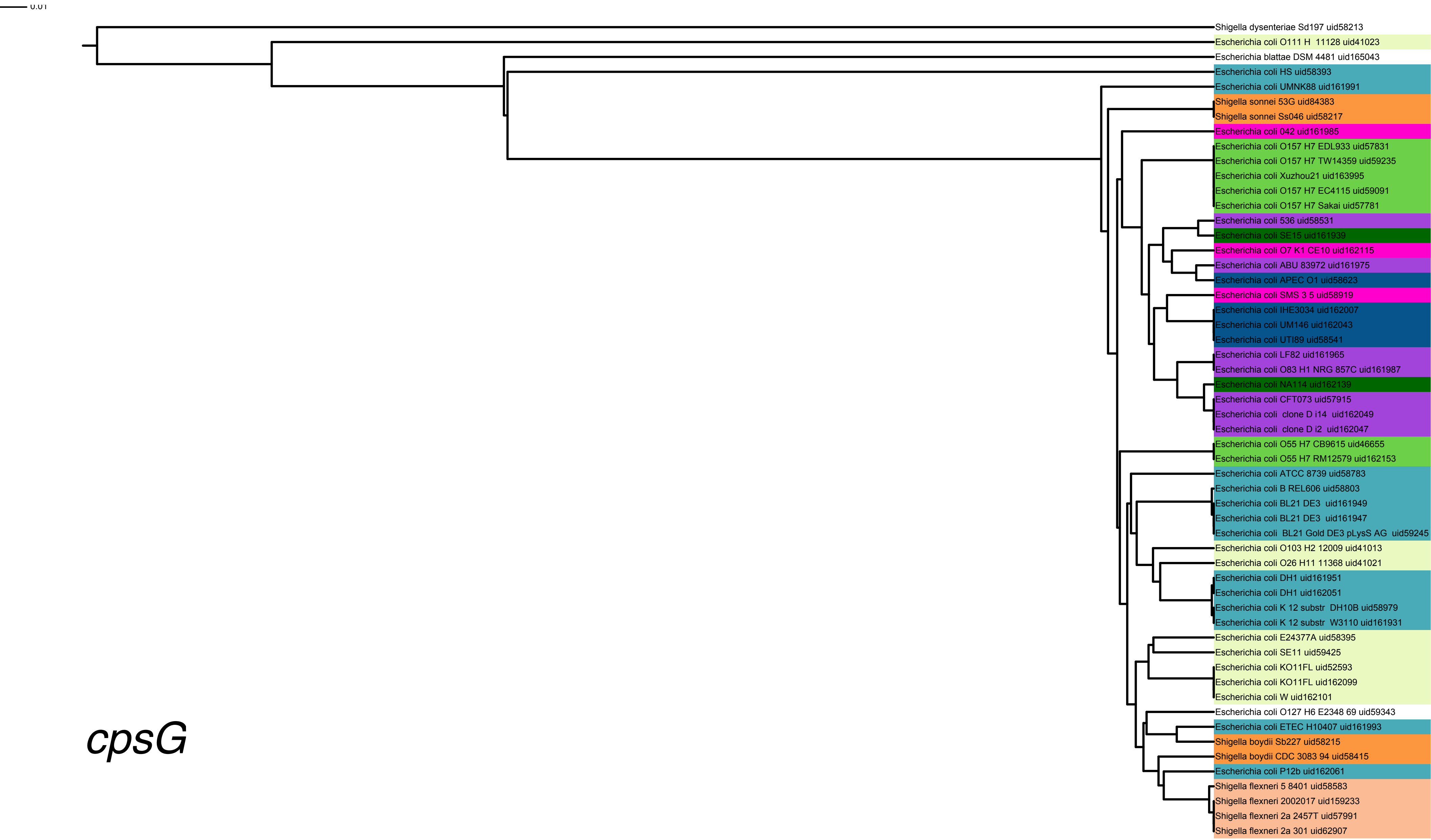
0.01
Shigella dysenteriae Sd197 uid58213
Escherichia coli O111 H 11128 uid41023
Escherichia blattae DSM 4481 uid165043
Escherichia coli HS uid58393
Escherichia coli UMNK88 uid161991
Shigella sonnei 53G uid84383
Shigella sonnei Ss046 uid58217
Escherichia coli 042 uid161985
Escherichia coli O157 H7 EDL933 uid57831
Escherichia coli O157 H7 TW14359 uid59235
Escherichia coli Xuzhou21 uid163995
Escherichia coli O157 H7 EC4115 uid59091
Escherichia coli O157 H7 Sakai uid57781
Escherichia coli 536 uid58531
Escherichia coli SE15 uid161939
Escherichia coli O7 K1 CE10 uid162115
Escherichia coli ABU 83972 uid161975
Escherichia coli APEC O1 uid58623
Escherichia coli SMS 3 5 uid58919
Escherichia coli IHE3034 uid162007
Escherichia coli UM146 uid162043
Escherichia coli UTI89 uid58541
Escherichia coli LF82 uid161965
Escherichia coli O83 H1 NRG 857C uid161987
Escherichia coli NA114 uid162139
Escherichia coli CFT073 uid57915
Escherichia coli clone D i14 uid162049
Escherichia coli clone D i2 uid162047
Escherichia coli O55 H7 CB9615 uid46655
Escherichia coli O55 H7 RM12579 uid162153
Escherichia coli ATCC 8739 uid58783
Escherichia coli B REL606 uid58803
Escherichia coli BL21 DE3 uid161949
Escherichia coli BL21 DE3 uid161947
Escherichia coli BL21 Gold DE3 pLysS AG uid59245
Escherichia coli O103 H2 12009 uid41013
Escherichia coli O26 H11 11368 uid41021
Escherichia coli DH1 uid161951
Escherichia coli DH1 uid162051
Escherichia coli K 12 substr DH10B uid58979
Escherichia coli K 12 substr W3110 uid161931
Escherichia coli E24377A uid58395
Escherichia coli SE11 uid59425
Escherichia coli KO11FL uid52593
Escherichia coli KO11FL uid162099
Escherichia coli W uid162101
Escherichia coli O127 H6 E2348 69 uid59343
Escherichia coli ETEC H10407 uid161993
Shigella boydii Sb227 uid58215
Shigella boydii CDC 3083 94 uid58415
Escherichia coli P12b uid162061
Shigella flexneri 5 8401 uid58583
Shigella flexneri 2002017 uid159233
Shigella flexneri 2a 2457T uid57991
Shigella flexneri 2a 301 uid62907
cpsG

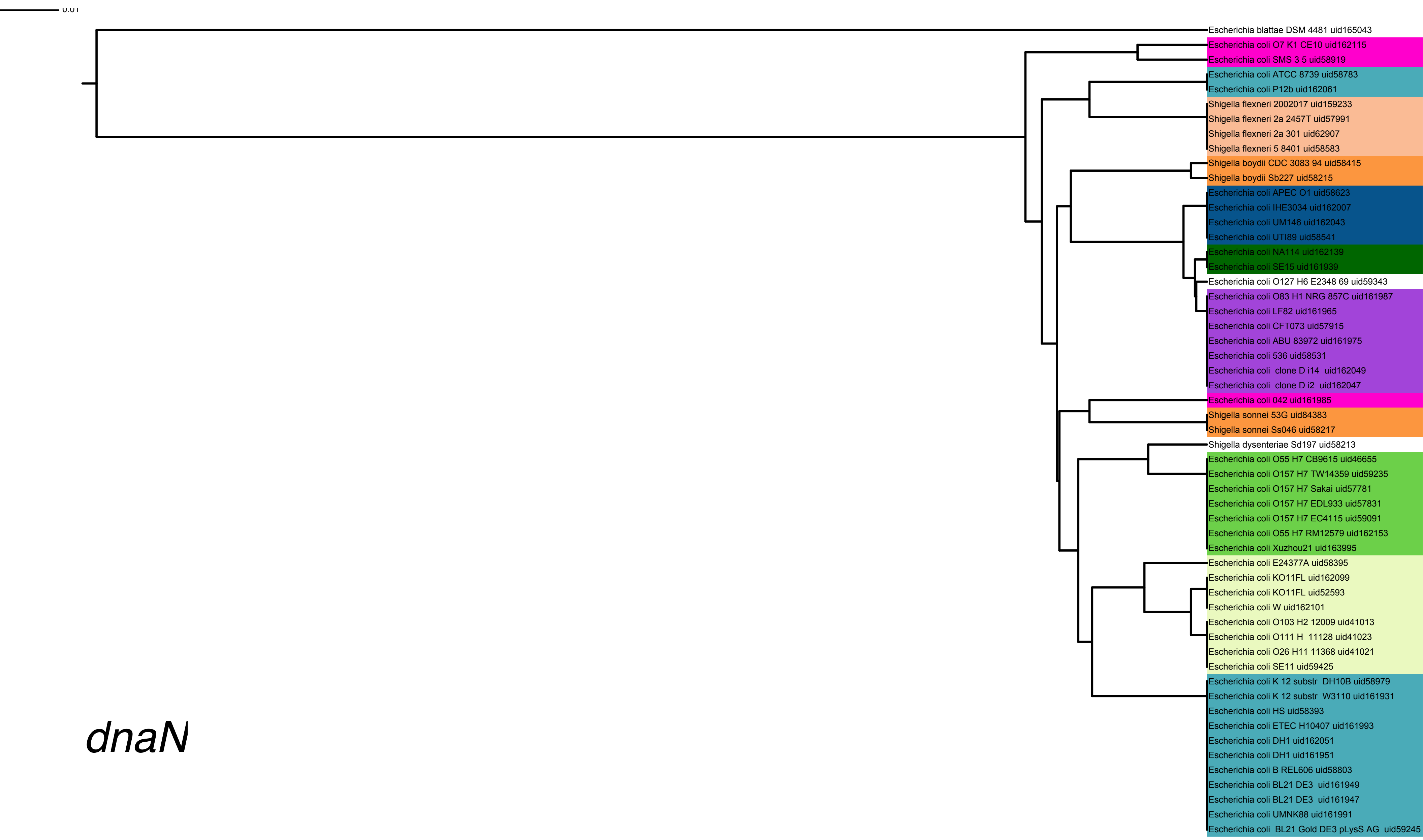
0.01
Escherichia blattae DSM 4481 uid165043
Escherichia coli O7 K1 CE10 uid162115
Escherichia coli SMS 3 5 uid58919
Escherichia coli ATCC 8739 uid58783
Escherichia coli P12b uid162061
Shigella flexneri 2002017 uid159233
Shigella flexneri 2a 2457T uid57991
Shigella flexneri 2a 301 uid62907
Shigella flexneri 5 8401 uid58583
Shigella boydii CDC 3083 94 uid58415
Shigella boydii Sb227 uid58215
Escherichia coli APEC O1 uid58623
Escherichia coli IHE3034 uid162007
Escherichia coli UM146 uid162043
Escherichia coli UTI89 uid58541
Escherichia coli NA114 uid162139
Escherichia coli SE15 uid161939
Escherichia coli O127 H6 E2348 69 uid59343
Escherichia coli O83 H1 NRG 857C uid161987
Escherichia coli LF82 uid161965
Escherichia coli CFT073 uid57915
Escherichia coli ABU 83972 uid161975
Escherichia coli 536 uid58531
Escherichia coli clone D i14 uid162049
Escherichia coli clone D i2 uid162047
Escherichia coli 042 uid161985
Shigella sonnei 53G uid84383
Shigella sonnei Ss046 uid58217
Shigella dysenteriae Sd197 uid58213
Escherichia coli O55 H7 CB9615 uid46655
Escherichia coli O157 H7 TW14359 uid59235
Escherichia coli O157 H7 Sakai uid57781
Escherichia coli O157 H7 EDL933 uid57831
Escherichia coli O157 H7 EC4115 uid59091
Escherichia coli O55 H7 RM12579 uid162153
Escherichia coli Xuzhou21 uid163995
Escherichia coli E24377A uid58395
Escherichia coli KO11FL uid162099
Escherichia coli KO11FL uid52593
Escherichia coli W uid162101
Escherichia coli O103 H2 12009 uid41013
Escherichia coli O111 H 11128 uid41023
Escherichia coli O26 H11 11368 uid41021
Escherichia coli SE11 uid59425
Escherichia coli K 12 substr DH10B uid58979
Escherichia coli K 12 substr W3110 uid161931
Escherichia coli HS uid58393
Escherichia coli ETEC H10407 uid161993
Escherichia coli DH1 uid162051
Escherichia coli DH1 uid161951
Escherichia coli B REL606 uid58803
Escherichia coli BL21 DE3 uid161949
Escherichia coli BL21 DE3 uid161947
Escherichia coli UMNK88 uid161991
Escherichia coli BL21 Gold DE3 pLysS AG uid59245
dnaN

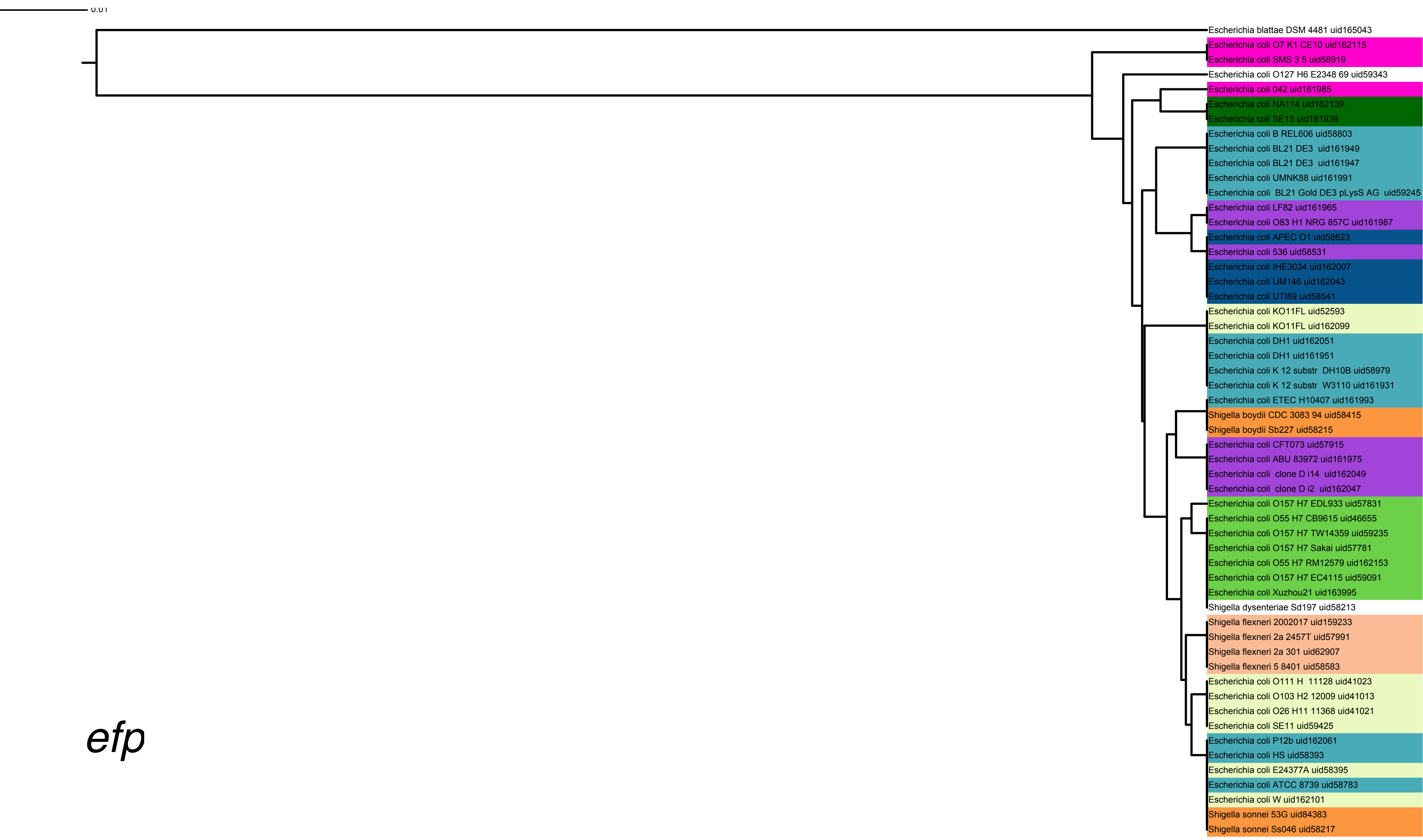
0.01
Escherichia blattae DSM 4481 uid165043
Escherichia coli O7 K1 CE10 uid162115
Escherichia coli SMS 3 5 uid58919
Escherichia coli O127 H6 E2348 69 uid59343
Escherichia coli 042 uid161985
Escherichia coli NA114 uid162139
Escherichia coli SE15 uid161939
Escherichia coli B REL606 uid58803
Escherichia coli BL21 DE3 uid161949
Escherichia coli BL21 DE3 uid161947
Escherichia coli UMNK88 uid161991
Escherichia coli BL21 Gold DE3 pLysS AG uid59245
Escherichia coli LF82 uid161965
Escherichia coli O83 H1 NRG 857C uid161987
Escherichia coli APEC O1 uid58623
Escherichia coli 536 uid58531
Escherichia coli IHE3034 uid162007
Escherichia coli UM146 uid162043
Escherichia coli UTI89 uid58541
Escherichia coli KO11FL uid52593
Escherichia coli KO11FL uid162099
Escherichia coli DH1 uid162051
Escherichia coli DH1 uid161951
Escherichia coli K 12 substr DH10B uid58979
Escherichia coli K 12 substr W3110 uid161931
Escherichia coli ETEC H10407 uid161993
Shigella boydii CDC 3083 94 uid58415
Shigella boydii Sb227 uid58215
Escherichia coli CFT073 uid57915
Escherichia coli ABU 83972 uid161975
Escherichia coli clone D i14 uid162049
Escherichia coli clone D i2 uid162047
Escherichia coli O157 H7 EDL933 uid57831
Escherichia coli O55 H7 CB9615 uid46655
Escherichia coli O157 H7 TW14359 uid59235
Escherichia coli O157 H7 Sakai uid57781
Escherichia coli O55 H7 RM12579 uid162153
Escherichia coli O157 H7 EC4115 uid59091
Escherichia coli Xuzhou21 uid163995
Shigella dysenteriae Sd197 uid58213
Shigella flexneri 2002017 uid159233
Shigella flexneri 2a 2457T uid57991
Shigella flexneri 2a 301 uid62907
Shigella flexneri 5 8401 uid58583
Escherichia coli O111 H 11128 uid41023
Escherichia coli O103 H2 12009 uid41013
Escherichia coli O26 H11 11368 uid41021
Escherichia coli SE11 uid59425
Escherichia coli P12b uid162061
Escherichia coli HS uid58393
Escherichia coli E24377A uid58395
Escherichia coli ATCC 8739 uid58783
Escherichia coli W uid162101
Shigella sonnei 53G uid84383
Shigella sonnei Ss046 uid58217
efp

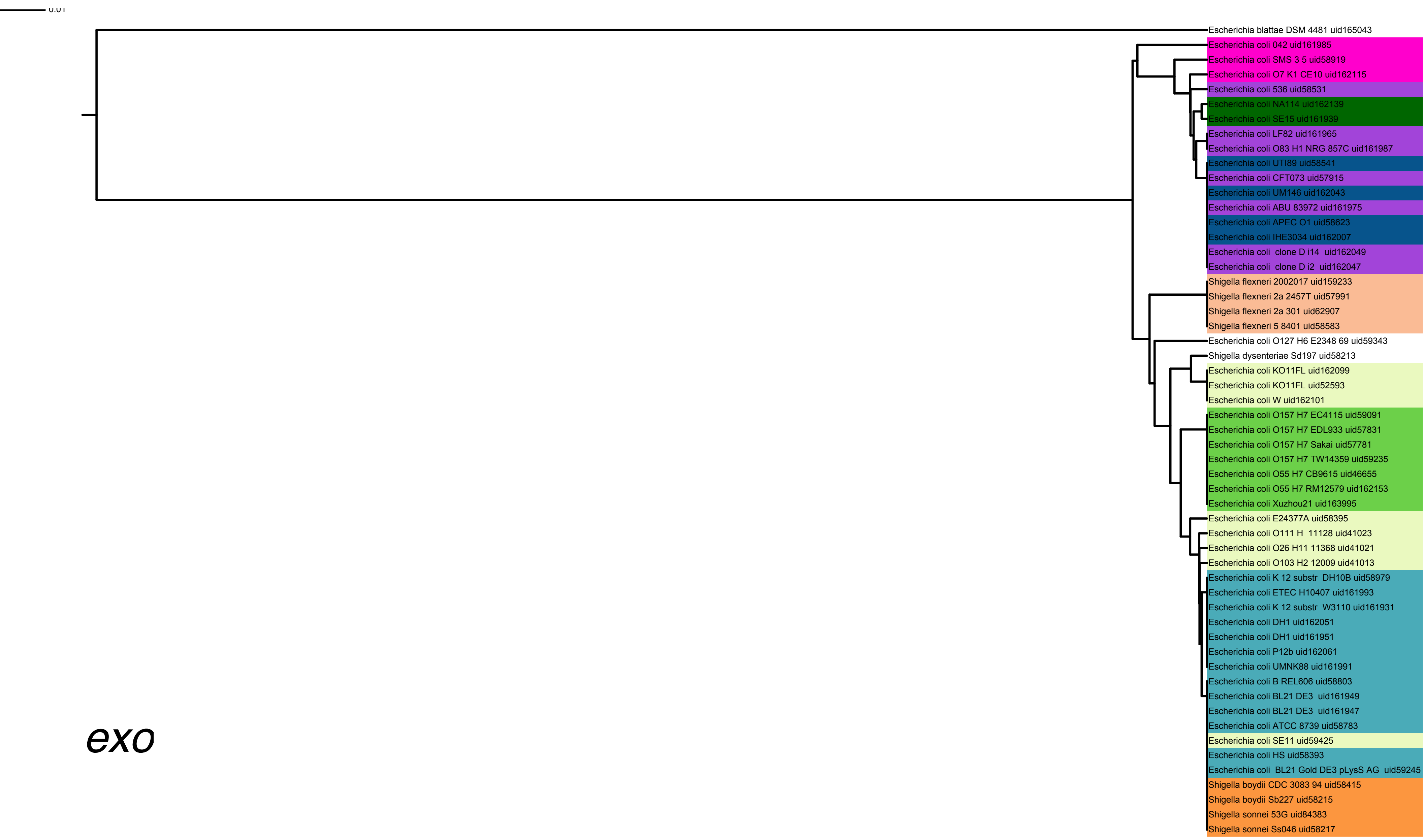
0.01
Escherichia blattae DSM 4481 uid165043
Escherichia coli 042 uid161985
Escherichia coli SMS 3 5 uid58919
Escherichia coli O7 K1 CE10 uid162115
Escherichia coli 536 uid58531
Escherichia coli NA114 uid162139
Escherichia coli SE15 uid161939
Escherichia coli LF82 uid161965
Escherichia coli O83 H1 NRG 857C uid161987
Escherichia coli UTI89 uid58541
Escherichia coli CFT073 uid57915
Escherichia coli UM146 uid162043
Escherichia coli ABU 83972 uid161975
Escherichia coli APEC O1 uid58623
Escherichia coli IHE3034 uid162007
Escherichia coli clone D i14 uid162049
Escherichia coli clone D i2 uid162047
Shigella flexneri 2002017 uid159233
Shigella flexneri 2a 2457T uid57991
Shigella flexneri 2a 301 uid62907
Shigella flexneri 5 8401 uid58583
Escherichia coli O127 H6 E2348 69 uid59343
Shigella dysenteriae Sd197 uid58213
Escherichia coli KO11FL uid162099
Escherichia coli KO11FL uid52593
Escherichia coli W uid162101
Escherichia coli O157 H7 EC4115 uid59091
Escherichia coli O157 H7 EDL933 uid57831
Escherichia coli O157 H7 Sakai uid57781
Escherichia coli O157 H7 TW14359 uid59235
Escherichia coli O55 H7 CB9615 uid46655
Escherichia coli O55 H7 RM12579 uid162153
Escherichia coli Xuzhou21 uid163995
Escherichia coli E24377A uid58395
Escherichia coli O111 H 11128 uid41023
Escherichia coli O26 H11 11368 uid41021
Escherichia coli O103 H2 12009 uid41013
Escherichia coli K 12 substr DH10B uid58979
Escherichia coli ETEC H10407 uid161993
Escherichia coli K 12 substr W3110 uid161931
Escherichia coli DH1 uid162051
Escherichia coli DH1 uid161951
Escherichia coli P12b uid162061
Escherichia coli UMNK88 uid161991
Escherichia coli B REL606 uid58803
Escherichia coli BL21 DE3 uid161949
Escherichia coli BL21 DE3 uid161947
Escherichia coli ATCC 8739 uid58783
Escherichia coli SE11 uid59425
Escherichia coli HS uid58393
Escherichia coli BL21 Gold DE3 pLysS AG uid59245
Shigella boydii CDC 3083 94 uid58415
Shigella boydii Sb227 uid58215
Shigella sonnei 53G uid84383
Shigella sonnei Ss046 uid58217
exo

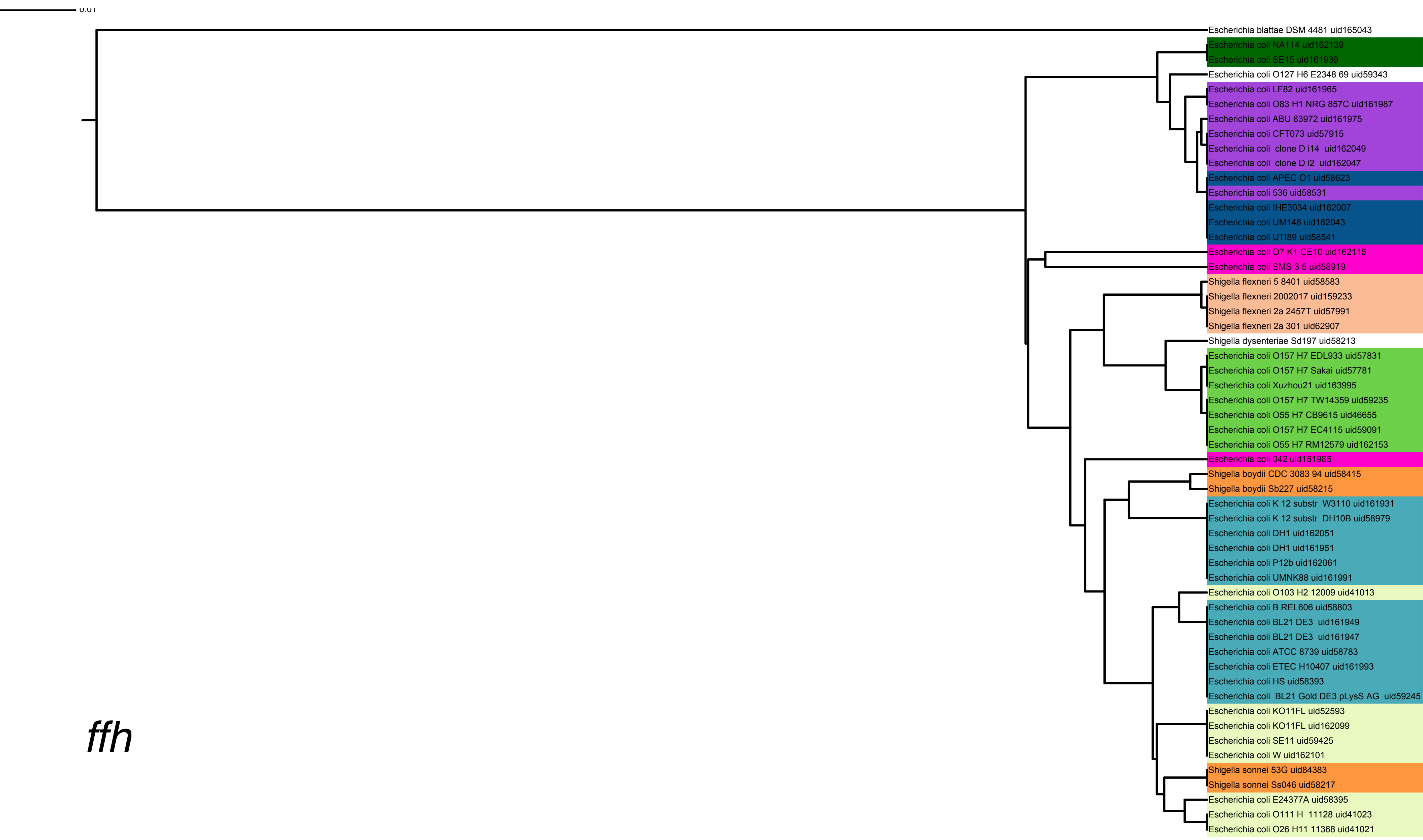
0.01
Escherichia blattae DSM 4481 uid165043
Escherichia coli NA114 uid162139
Escherichia coli SE15 uid161939
Escherichia coli O127 H6 E2348 69 uid59343
Escherichia coli LF82 uid161965
Escherichia coli O83 H1 NRG 857C uid161987
Escherichia coli ABU 83972 uid161975
Escherichia coli CFT073 uid57915
Escherichia coli clone D i14 uid162049
Escherichia coli clone D i2 uid162047
Escherichia coli APEC O1 uid58623
Escherichia coli 536 uid58531
Escherichia coli IHE3034 uid162007
Escherichia coli UM146 uid162043
Escherichia coli UTI89 uid58541
Escherichia coli O7 K1 CE10 uid162115
Escherichia coli SMS 3 5 uid58919
Shigella flexneri 5 8401 uid58583
Shigella flexneri 2002017 uid159233
Shigella flexneri 2a 2457T uid57991
Shigella flexneri 2a 301 uid62907
Shigella dysenteriae Sd197 uid58213
Escherichia coli O157 H7 EDL933 uid57831
Escherichia coli O157 H7 Sakai uid57781
Escherichia coli Xuzhou21 uid163995
Escherichia coli O157 H7 TW14359 uid59235
Escherichia coli O55 H7 CB9615 uid46655
Escherichia coli O157 H7 EC4115 uid59091
Escherichia coli O55 H7 RM12579 uid162153
Escherichia coli 042 uid161985
Shigella boydii CDC 3083 94 uid58415
Shigella boydii Sb227 uid58215
Escherichia coli K 12 substr W3110 uid161931
Escherichia coli K 12 substr DH10B uid58979
Escherichia coli DH1 uid162051
Escherichia coli DH1 uid161951
Escherichia coli P12b uid162061
Escherichia coli UMNK88 uid161991
Escherichia coli O103 H2 12009 uid41013
Escherichia coli B REL606 uid58803
Escherichia coli BL21 DE3 uid161949
Escherichia coli BL21 DE3 uid161947
Escherichia coli ATCC 8739 uid58783
Escherichia coli ETEC H10407 uid161993
Escherichia coli HS uid58393
Escherichia coli BL21 Gold DE3 pLysS AG uid59245
Escherichia coli KO11FL uid52593
Escherichia coli KO11FL uid162099
Escherichia coli SE11 uid59425
Escherichia coli W uid162101
Shigella sonnei 53G uid84383
Shigella sonnei Ss046 uid58217
Escherichia coli E24377A uid58395
Escherichia coli O111 H 11128 uid41023
Escherichia coli O26 H11 11368 uid41021
ffh

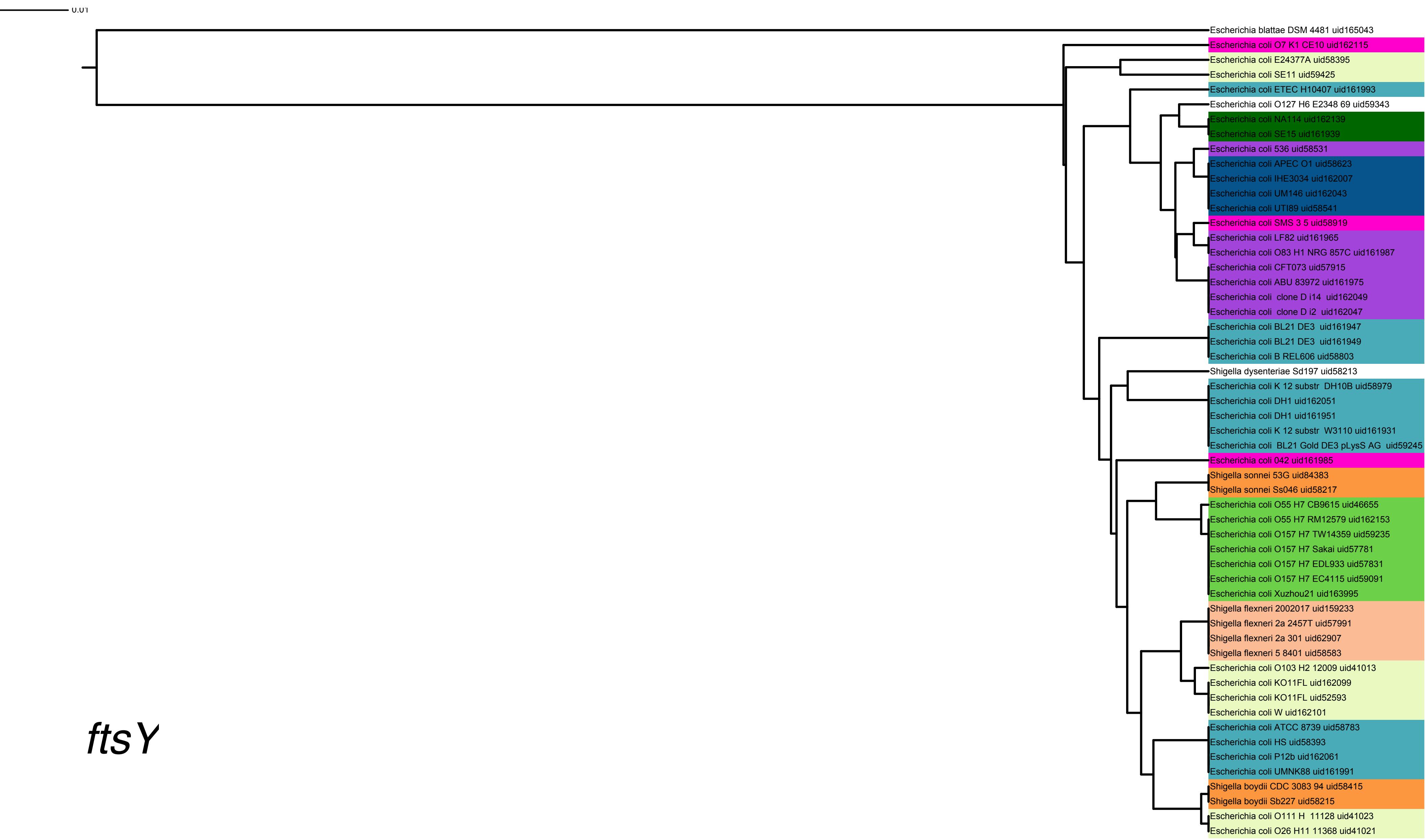
0.01
Escherichia blattae DSM 4481 uid165043
Escherichia coli O7 K1 CE10 uid162115
Escherichia coli E24377A uid58395
Escherichia coli SE11 uid59425
Escherichia coli ETEC H10407 uid161993
Escherichia coli O127 H6 E2348 69 uid59343
Escherichia coli NA114 uid162139
Escherichia coli SE15 uid161939
Escherichia coli 536 uid58531
Escherichia coli APEC O1 uid58623
Escherichia coli IHE3034 uid162007
Escherichia coli UM146 uid162043
Escherichia coli UTI89 uid58541
Escherichia coli SMS 3 5 uid58919
Escherichia coli LF82 uid161965
Escherichia coli O83 H1 NRG 857C uid161987
Escherichia coli CFT073 uid57915
Escherichia coli ABU 83972 uid161975
Escherichia coli clone D i14 uid162049
Escherichia coli clone D i2 uid162047
Escherichia coli BL21 DE3 uid161947
Escherichia coli BL21 DE3 uid161949
Escherichia coli B REL606 uid58803
Shigella dysenteriae Sd197 uid58213
Escherichia coli K 12 substr DH10B uid58979
Escherichia coli DH1 uid162051
Escherichia coli DH1 uid161951
Escherichia coli K 12 substr W3110 uid161931
Escherichia coli BL21 Gold DE3 pLysS AG uid59245
Escherichia coli 042 uid161985
Shigella sonnei 53G uid84383
Shigella sonnei Ss046 uid58217
Escherichia coli O55 H7 CB9615 uid46655
Escherichia coli O55 H7 RM12579 uid162153
Escherichia coli O157 H7 TW14359 uid59235
Escherichia coli O157 H7 Sakai uid57781
Escherichia coli O157 H7 EDL933 uid57831
Escherichia coli O157 H7 EC4115 uid59091
Escherichia coli Xuzhou21 uid163995
Shigella flexneri 2002017 uid159233
Shigella flexneri 2a 2457T uid57991
Shigella flexneri 2a 301 uid62907
Shigella flexneri 5 8401 uid58583
Escherichia coli O103 H2 12009 uid41013
Escherichia coli KO11FL uid162099
Escherichia coli KO11FL uid52593
Escherichia coli W uid162101
Escherichia coli ATCC 8739 uid58783
Escherichia coli HS uid58393
Escherichia coli P12b uid162061
Escherichia coli UMNK88 uid161991
Shigella boydii CDC 3083 94 uid58415
Shigella boydii Sb227 uid58215
Escherichia coli O111 H 11128 uid41023
Escherichia coli O26 H11 11368 uid41021
ftsY

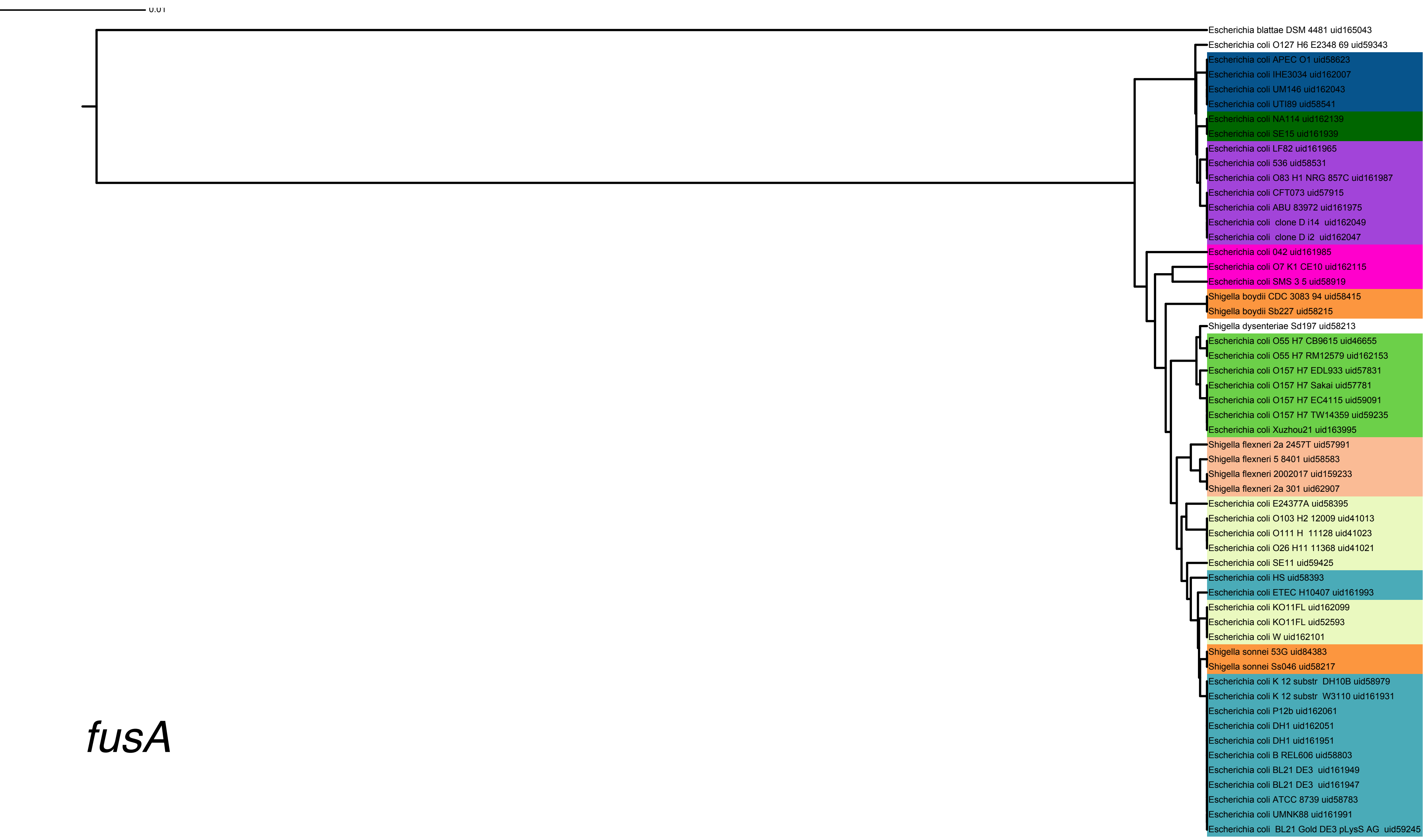
0.01
Escherichia blattae DSM 4481 uid165043
Escherichia coli O127 H6 E2348 69 uid59343
Escherichia coli APEC O1 uid58623
Escherichia coli IHE3034 uid162007
Escherichia coli UM146 uid162043
Escherichia coli UTI89 uid58541
Escherichia coli NA114 uid162139
Escherichia coli SE15 uid161939
Escherichia coli LF82 uid161965
Escherichia coli 536 uid58531
Escherichia coli O83 H1 NRG 857C uid161987
Escherichia coli CFT073 uid57915
Escherichia coli ABU 83972 uid161975
Escherichia coli clone D i14 uid162049
Escherichia coli clone D i2 uid162047
Escherichia coli 042 uid161985
Escherichia coli O7 K1 CE10 uid162115
Escherichia coli SMS 3 5 uid58919
Shigella boydii CDC 3083 94 uid58415
Shigella boydii Sb227 uid58215
Shigella dysenteriae Sd197 uid58213
Escherichia coli O55 H7 CB9615 uid46655
Escherichia coli O55 H7 RM12579 uid162153
Escherichia coli O157 H7 EDL933 uid57831
Escherichia coli O157 H7 Sakai uid57781
Escherichia coli O157 H7 EC4115 uid59091
Escherichia coli O157 H7 TW14359 uid59235
Escherichia coli Xuzhou21 uid163995
Shigella flexneri 2a 2457T uid57991
Shigella flexneri 5 8401 uid58583
Shigella flexneri 2002017 uid159233
Shigella flexneri 2a 301 uid62907
Escherichia coli E24377A uid58395
Escherichia coli O103 H2 12009 uid41013
Escherichia coli O111 H 11128 uid41023
Escherichia coli O26 H11 11368 uid41021
Escherichia coli SE11 uid59425
Escherichia coli HS uid58393
Escherichia coli ETEC H10407 uid161993
Escherichia coli KO11FL uid162099
Escherichia coli KO11FL uid52593
Escherichia coli W uid162101
Shigella sonnei 53G uid84383
Shigella sonnei Ss046 uid58217
Escherichia coli K 12 substr DH10B uid58979
Escherichia coli K 12 substr W3110 uid161931
Escherichia coli P12b uid162061
Escherichia coli DH1 uid162051
Escherichia coli DH1 uid161951
Escherichia coli B REL606 uid58803
Escherichia coli BL21 DE3 uid161949
Escherichia coli BL21 DE3 uid161947
Escherichia coli ATCC 8739 uid58783
Escherichia coli UMNK88 uid161991
Escherichia coli BL21 Gold DE3 pLysS AG uid59245
fusA

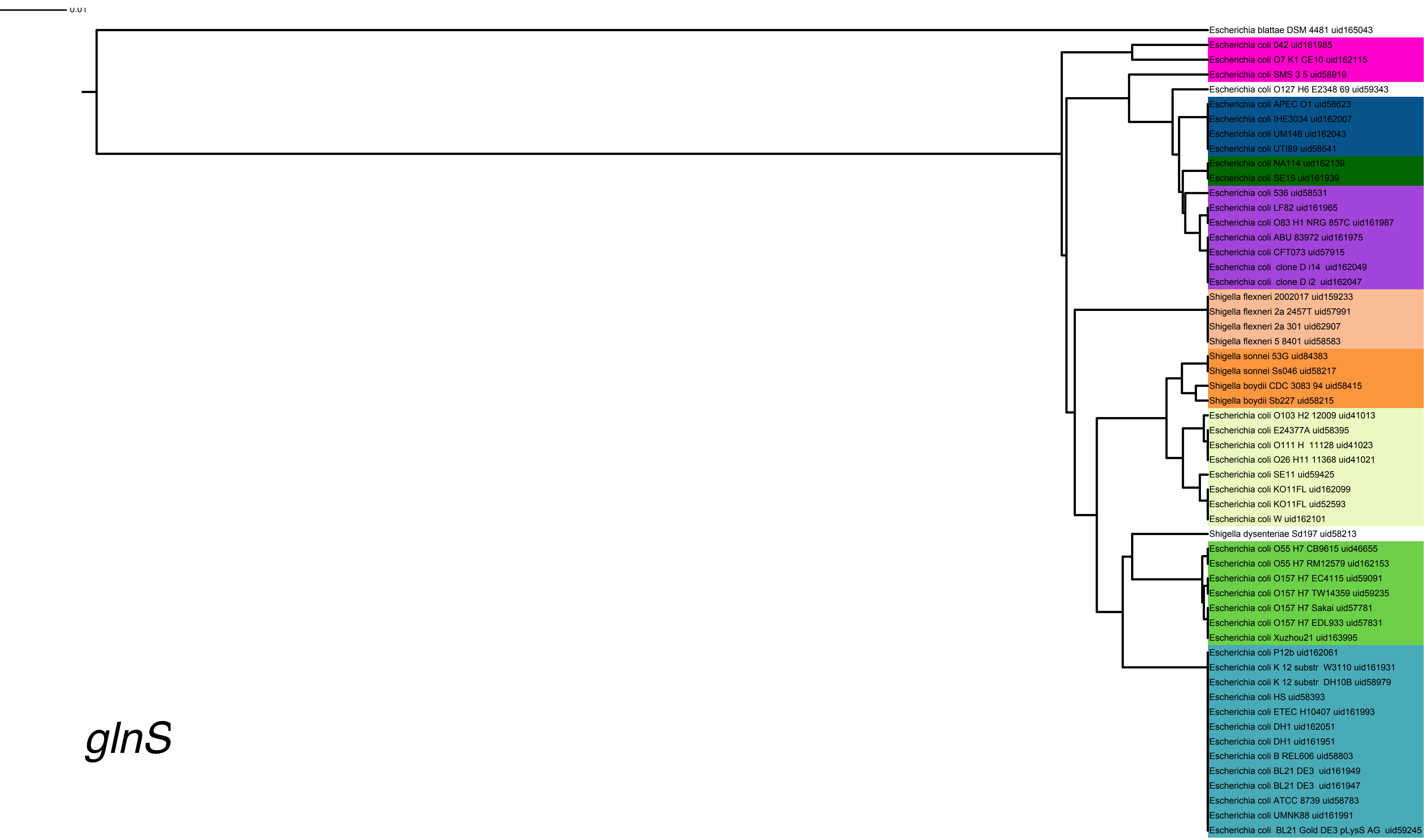
0.01
Escherichia blattae DSM 4481 uid165043
Escherichia coli 042 uid161985
Escherichia coli O7 K1 CE10 uid162115
Escherichia coli SMS 3 5 uid58919
Escherichia coli O127 H6 E2348 69 uid59343
Escherichia coli APEC O1 uid58623
Escherichia coli IHE3034 uid162007
Escherichia coli UM146 uid162043
Escherichia coli UTI89 uid58541
Escherichia coli NA114 uid162139
Escherichia coli SE15 uid161939
Escherichia coli 536 uid58531
Escherichia coli LF82 uid161965
Escherichia coli O83 H1 NRG 857C uid161987
Escherichia coli ABU 83972 uid161975
Escherichia coli CFT073 uid57915
Escherichia coli clone D i14 uid162049
Escherichia coli clone D i2 uid162047
Shigella flexneri 2002017 uid159233
Shigella flexneri 2a 2457T uid57991
Shigella flexneri 2a 301 uid62907
Shigella flexneri 5 8401 uid58583
Shigella sonnei 53G uid84383
Shigella sonnei Ss046 uid58217
Shigella boydii CDC 3083 94 uid58415
Shigella boydii Sb227 uid58215
Escherichia coli O103 H2 12009 uid41013
Escherichia coli E24377A uid58395
Escherichia coli O111 H 11128 uid41023
Escherichia coli O26 H11 11368 uid41021
Escherichia coli SE11 uid59425
Escherichia coli KO11FL uid162099
Escherichia coli KO11FL uid52593
Escherichia coli W uid162101
Shigella dysenteriae Sd197 uid58213
Escherichia coli O55 H7 CB9615 uid46655
Escherichia coli O55 H7 RM12579 uid162153
Escherichia coli O157 H7 EC4115 uid59091
Escherichia coli O157 H7 TW14359 uid59235
Escherichia coli O157 H7 Sakai uid57781
Escherichia coli O157 H7 EDL933 uid57831
Escherichia coli Xuzhou21 uid163995
Escherichia coli P12b uid162061
Escherichia coli K 12 substr W3110 uid161931
Escherichia coli K 12 substr DH10B uid58979
Escherichia coli HS uid58393
Escherichia coli ETEC H10407 uid161993
Escherichia coli DH1 uid162051
Escherichia coli DH1 uid161951
Escherichia coli B REL606 uid58803
Escherichia coli BL21 DE3 uid161949
Escherichia coli BL21 DE3 uid161947
Escherichia coli ATCC 8739 uid58783
Escherichia coli UMNK88 uid161991
Escherichia coli BL21 Gold DE3 pLysS AG uid59245

glnS

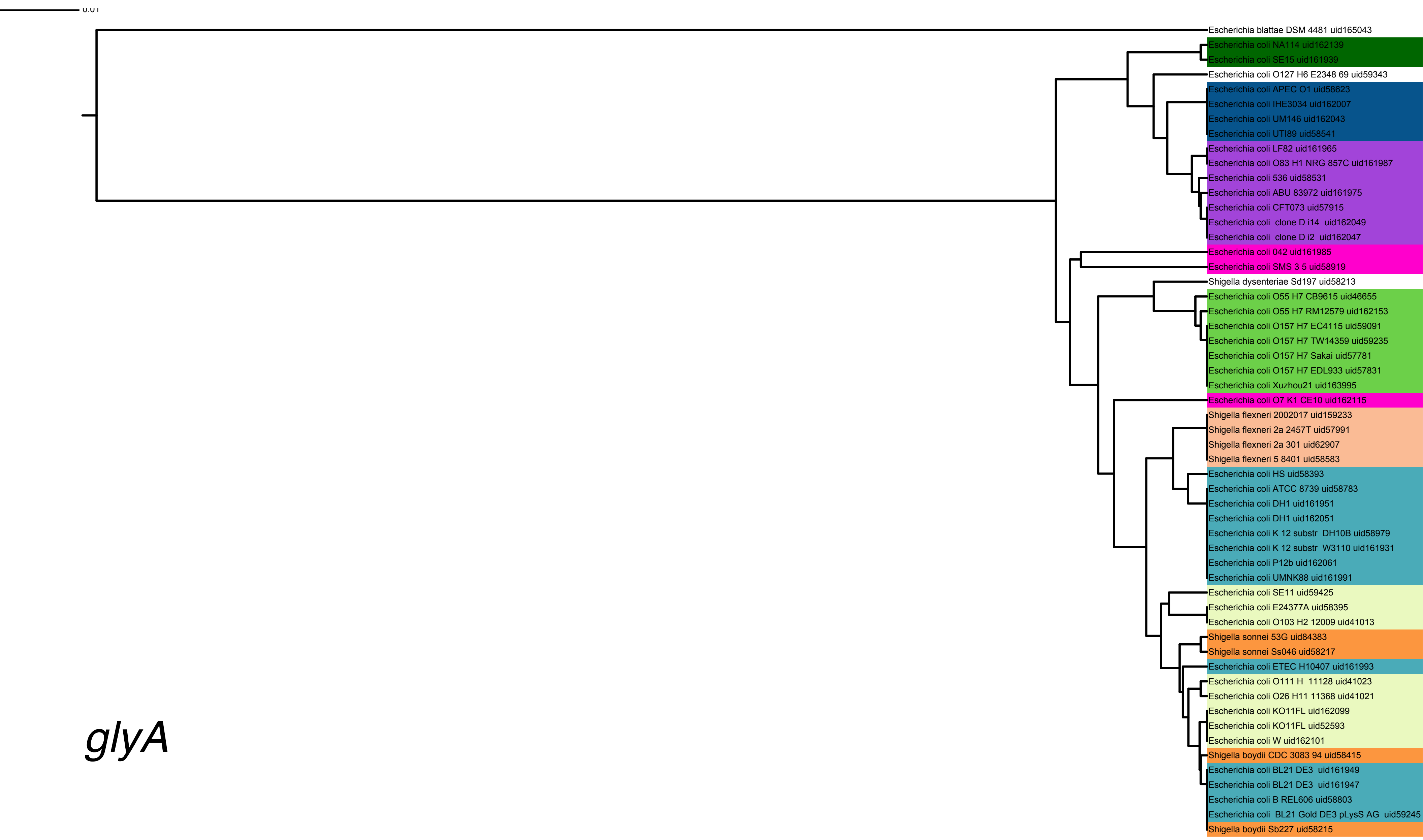
0.01
Escherichia blattae DSM 4481 uid165043
Escherichia coli NA114 uid162139
Escherichia coli SE15 uid161939
Escherichia coli O127 H6 E2348 69 uid59343
Escherichia coli APEC O1 uid58623
Escherichia coli IHE3034 uid162007
Escherichia coli UM146 uid162043
Escherichia coli UTI89 uid58541
Escherichia coli LF82 uid161965
Escherichia coli O83 H1 NRG 857C uid161987
Escherichia coli 536 uid58531
Escherichia coli ABU 83972 uid161975
Escherichia coli CFT073 uid57915
Escherichia coli clone D i14 uid162049
Escherichia coli clone D i2 uid162047
Escherichia coli 042 uid161985
Escherichia coli SMS 3 5 uid58919
Shigella dysenteriae Sd197 uid58213
Escherichia coli O55 H7 CB9615 uid46655
Escherichia coli O55 H7 RM12579 uid162153
Escherichia coli O157 H7 EC4115 uid59091
Escherichia coli O157 H7 TW14359 uid59235
Escherichia coli O157 H7 Sakai uid57781
Escherichia coli O157 H7 EDL933 uid57831
Escherichia coli Xuzhou21 uid163995
Escherichia coli O7 K1 CE10 uid162115
Shigella flexneri 2002017 uid159233
Shigella flexneri 2a 2457T uid57991
Shigella flexneri 2a 301 uid62907
Shigella flexneri 5 8401 uid58583
Escherichia coli HS uid58393
Escherichia coli ATCC 8739 uid58783
Escherichia coli DH1 uid161951
Escherichia coli DH1 uid162051
Escherichia coli K 12 substr DH10B uid58979
Escherichia coli K 12 substr W3110 uid161931
Escherichia coli P12b uid162061
Escherichia coli UMNK88 uid161991
Escherichia coli SE11 uid59425
Escherichia coli E24377A uid58395
Escherichia coli O103 H2 12009 uid41013
Shigella sonnei 53G uid84383
Shigella sonnei Ss046 uid58217
Escherichia coli ETEC H10407 uid161993
Escherichia coli O111 H 11128 uid41023
Escherichia coli O26 H11 11368 uid41021
Escherichia coli KO11FL uid162099
Escherichia coli KO11FL uid52593
Escherichia coli W uid162101
Shigella boydii CDC 3083 94 uid58415
Escherichia coli BL21 DE3 uid161949
Escherichia coli BL21 DE3 uid161947
Escherichia coli B REL606 uid58803
Escherichia coli BL21 Gold DE3 pLysS AG uid59245
Shigella boydii Sb227 uid58215
glyA

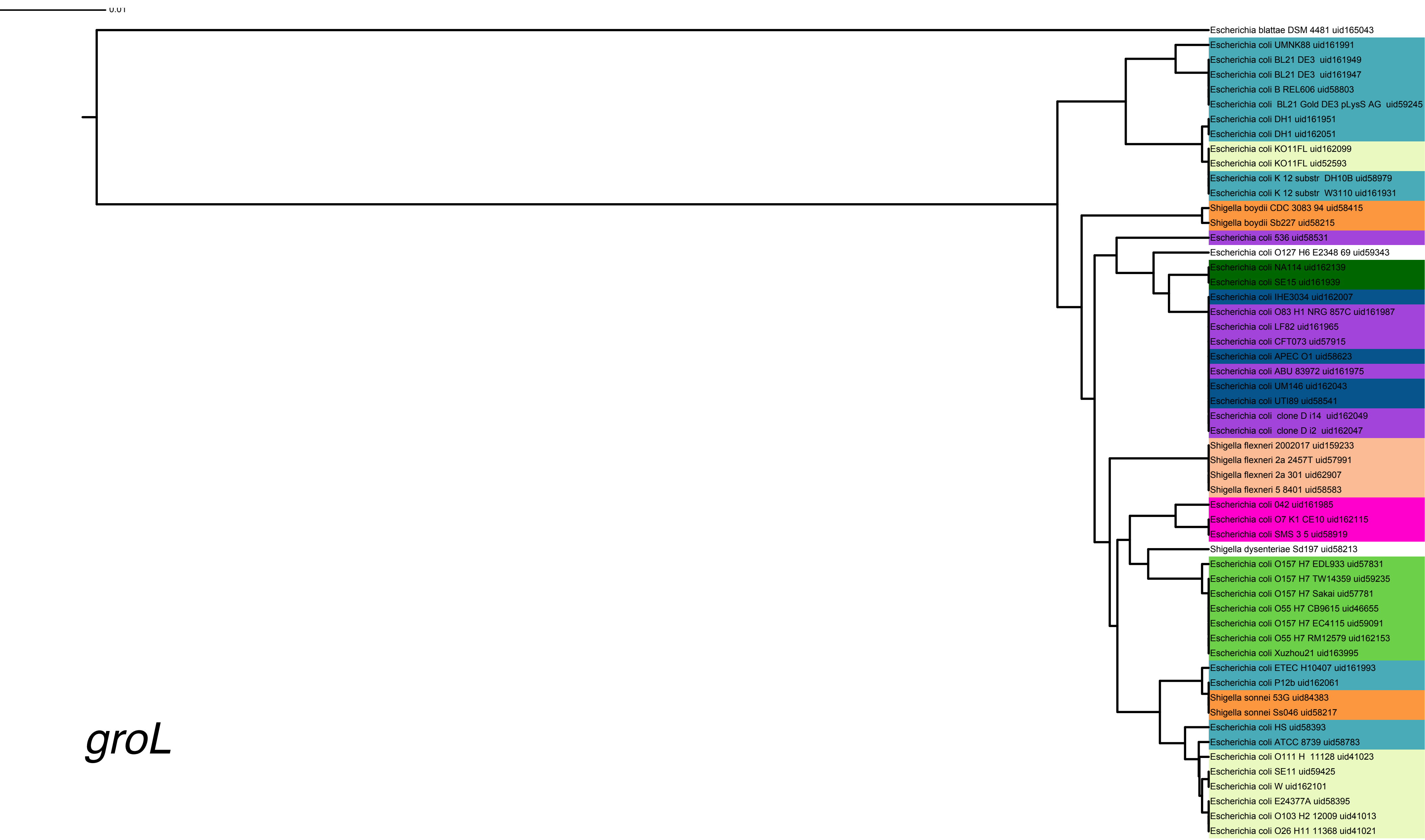
0.01
Escherichia blattae DSM 4481 uid165043
Escherichia coli UMNK88 uid161991
Escherichia coli BL21 DE3 uid161949
Escherichia coli BL21 DE3 uid161947
Escherichia coli B REL606 uid58803
Escherichia coli BL21 Gold DE3 pLysS AG uid59245
Escherichia coli DH1 uid161951
Escherichia coli DH1 uid162051
Escherichia coli KO11FL uid162099
Escherichia coli KO11FL uid52593
Escherichia coli K 12 substr DH10B uid58979
Escherichia coli K 12 substr W3110 uid161931
Shigella boydii CDC 3083 94 uid58415
Shigella boydii Sb227 uid58215
Escherichia coli 536 uid58531
Escherichia coli O127 H6 E2348 69 uid59343
Escherichia coli NA114 uid162139
Escherichia coli SE15 uid161939
Escherichia coli IHE3034 uid162007
Escherichia coli O83 H1 NRG 857C uid161987
Escherichia coli LF82 uid161965
Escherichia coli CFT073 uid57915
Escherichia coli APEC O1 uid58623
Escherichia coli ABU 83972 uid161975
Escherichia coli UM146 uid162043
Escherichia coli UTI89 uid58541
Escherichia coli clone D i14 uid162049
Escherichia coli clone D i2 uid162047
Shigella flexneri 2002017 uid159233
Shigella flexneri 2a 2457T uid57991
Shigella flexneri 2a 301 uid62907
Shigella flexneri 5 8401 uid58583
Escherichia coli 042 uid161985
Escherichia coli O7 K1 CE10 uid162115
Escherichia coli SMS 3 5 uid58919
Shigella dysenteriae Sd197 uid58213
Escherichia coli O157 H7 EDL933 uid57831
Escherichia coli O157 H7 TW14359 uid59235
Escherichia coli O157 H7 Sakai uid57781
Escherichia coli O55 H7 CB9615 uid46655
Escherichia coli O157 H7 EC4115 uid59091
Escherichia coli O55 H7 RM12579 uid162153
Escherichia coli Xuzhou21 uid163995
Escherichia coli ETEC H10407 uid161993
Escherichia coli P12b uid162061
Shigella sonnei 53G uid84383
Shigella sonnei Ss046 uid58217
Escherichia coli HS uid58393
Escherichia coli ATCC 8739 uid58783
Escherichia coli O111 H 11128 uid41023
Escherichia coli SE11 uid59425
Escherichia coli W uid162101
Escherichia coli E24377A uid58395
Escherichia coli O103 H2 12009 uid41013
Escherichia coli O26 H11 11368 uid41021
groL

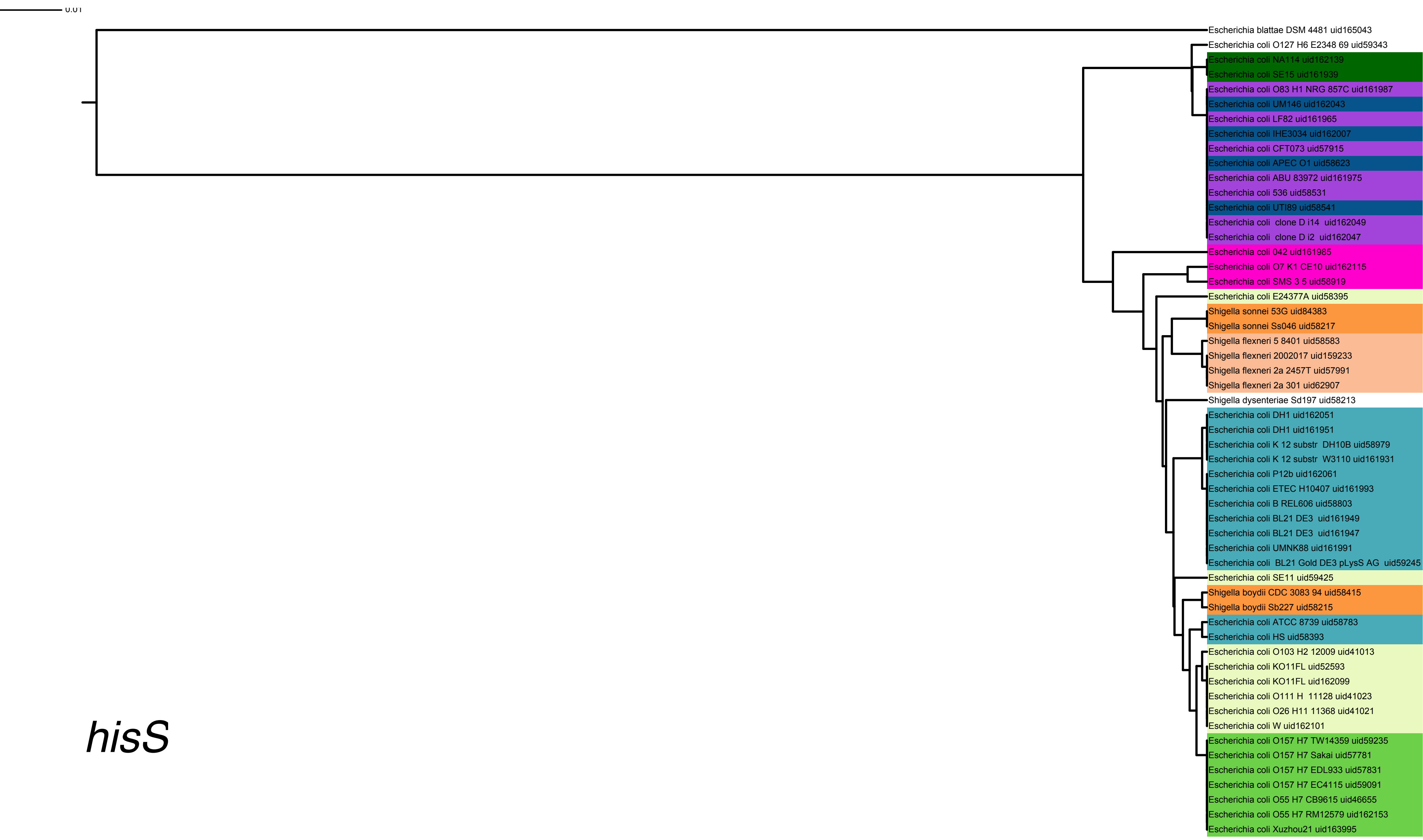

0.01
Escherichia blattae DSM 4481 uid165043
Escherichia coli O127 H6 E2348 69 uid59343
Escherichia coli NA114 uid162139
Escherichia coli SE15 uid161939
Escherichia coli O83 H1 NRG 857C uid161987
Escherichia coli UM146 uid162043
Escherichia coli LF82 uid161965
Escherichia coli IHE3034 uid162007
Escherichia coli CFT073 uid57915
Escherichia coli APEC O1 uid58623
Escherichia coli ABU 83972 uid161975
Escherichia coli 536 uid58531
Escherichia coli UTI89 uid58541
Escherichia coli clone D i14 uid162049
Escherichia coli clone D i2 uid162047
Escherichia coli 042 uid161985
Escherichia coli O7 K1 CE10 uid162115
Escherichia coli SMS 3 5 uid58919
Escherichia coli E24377A uid58395
Shigella sonnei 53G uid84383
Shigella sonnei Ss046 uid58217
Shigella flexneri 5 8401 uid58583
Shigella flexneri 2002017 uid159233
Shigella flexneri 2a 2457T uid57991
Shigella flexneri 2a 301 uid62907
Shigella dysenteriae Sd197 uid58213
Escherichia coli DH1 uid162051
Escherichia coli DH1 uid161951
Escherichia coli K 12 substr DH10B uid58979
Escherichia coli K 12 substr W3110 uid161931
Escherichia coli P12b uid162061
Escherichia coli ETEC H10407 uid161993
Escherichia coli B REL606 uid58803
Escherichia coli BL21 DE3 uid161949
Escherichia coli BL21 DE3 uid161947
Escherichia coli UMNK88 uid161991
Escherichia coli BL21 Gold DE3 pLysS AG uid59245
Escherichia coli SE11 uid59425
Shigella boydii CDC 3083 94 uid58415
Shigella boydii Sb227 uid58215
Escherichia coli ATCC 8739 uid58783
Escherichia coli HS uid58393
Escherichia coli O103 H2 12009 uid41013
Escherichia coli KO11FL uid52593
Escherichia coli KO11FL uid162099
Escherichia coli O111 H 11128 uid41023
Escherichia coli O26 H11 11368 uid41021
Escherichia coli W uid162101
Escherichia coli O157 H7 TW14359 uid59235
Escherichia coli O157 H7 Sakai uid57781
Escherichia coli O157 H7 EDL933 uid57831
Escherichia coli O157 H7 EC4115 uid59091
Escherichia coli O55 H7 CB9615 uid46655
Escherichia coli O55 H7 RM12579 uid162153
Escherichia coli Xuzhou21 uid163995
hisS

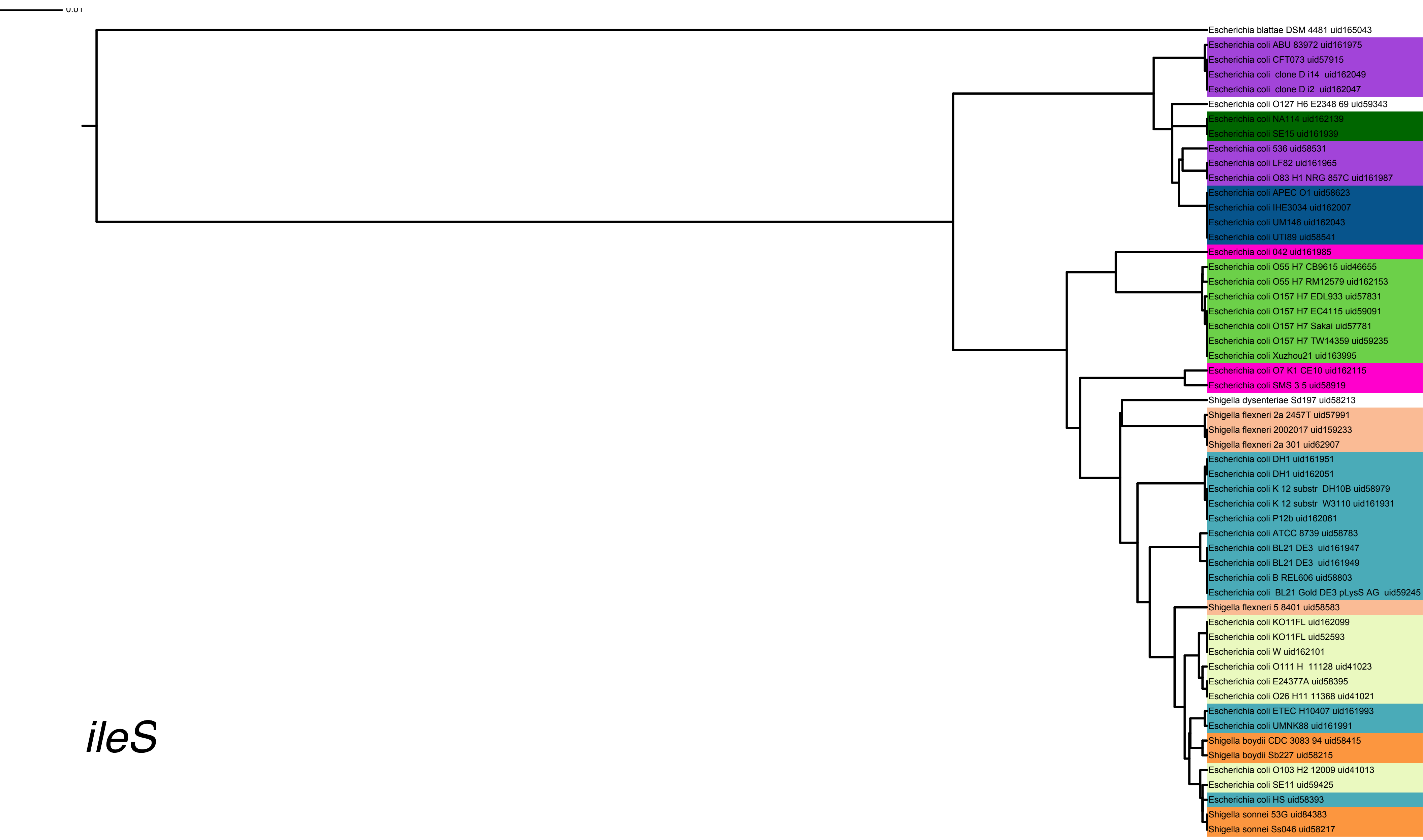

0.01
Escherichia blattae DSM 4481 uid165043
Escherichia coli ABU 83972 uid161975
Escherichia coli CFT073 uid57915
Escherichia coli clone D i14 uid162049
Escherichia coli clone D i2 uid162047
Escherichia coli O127 H6 E2348 69 uid59343
Escherichia coli NA114 uid162139
Escherichia coli SE15 uid161939
Escherichia coli 536 uid58531
Escherichia coli LF82 uid161965
Escherichia coli O83 H1 NRG 857C uid161987
Escherichia coli APEC O1 uid58623
Escherichia coli IHE3034 uid162007
Escherichia coli UM146 uid162043
Escherichia coli UTI89 uid58541
Escherichia coli 042 uid161985
Escherichia coli O55 H7 CB9615 uid46655
Escherichia coli O55 H7 RM12579 uid162153
Escherichia coli O157 H7 EDL933 uid57831
Escherichia coli O157 H7 EC4115 uid59091
Escherichia coli O157 H7 Sakai uid57781
Escherichia coli O157 H7 TW14359 uid59235
Escherichia coli Xuzhou21 uid163995
Escherichia coli O7 K1 CE10 uid162115
Escherichia coli SMS 3 5 uid58919
Shigella dysenteriae Sd197 uid58213
Shigella flexneri 2a 2457T uid57991
Shigella flexneri 2002017 uid159233
Shigella flexneri 2a 301 uid62907
Escherichia coli DH1 uid161951
Escherichia coli DH1 uid162051
Escherichia coli K 12 substr DH10B uid58979
Escherichia coli K 12 substr W3110 uid161931
Escherichia coli P12b uid162061
Escherichia coli ATCC 8739 uid58783
Escherichia coli BL21 DE3 uid161947
Escherichia coli BL21 DE3 uid161949
Escherichia coli B REL606 uid58803
Escherichia coli BL21 Gold DE3 pLysS AG uid59245
Shigella flexneri 5 8401 uid58583
Escherichia coli KO11FL uid162099
Escherichia coli KO11FL uid52593
Escherichia coli W uid162101
Escherichia coli O111 H 11128 uid41023
Escherichia coli E24377A uid58395
Escherichia coli O26 H11 11368 uid41021
Escherichia coli ETEC H10407 uid161993
Escherichia coli UMNK88 uid161991
Shigella boydii CDC 3083 94 uid58415
Shigella boydii Sb227 uid58215
Escherichia coli O103 H2 12009 uid41013
Escherichia coli SE11 uid59425
Escherichia coli HS uid58393
Shigella sonnei 53G uid84383
Shigella sonnei Ss046 uid58217
ileS

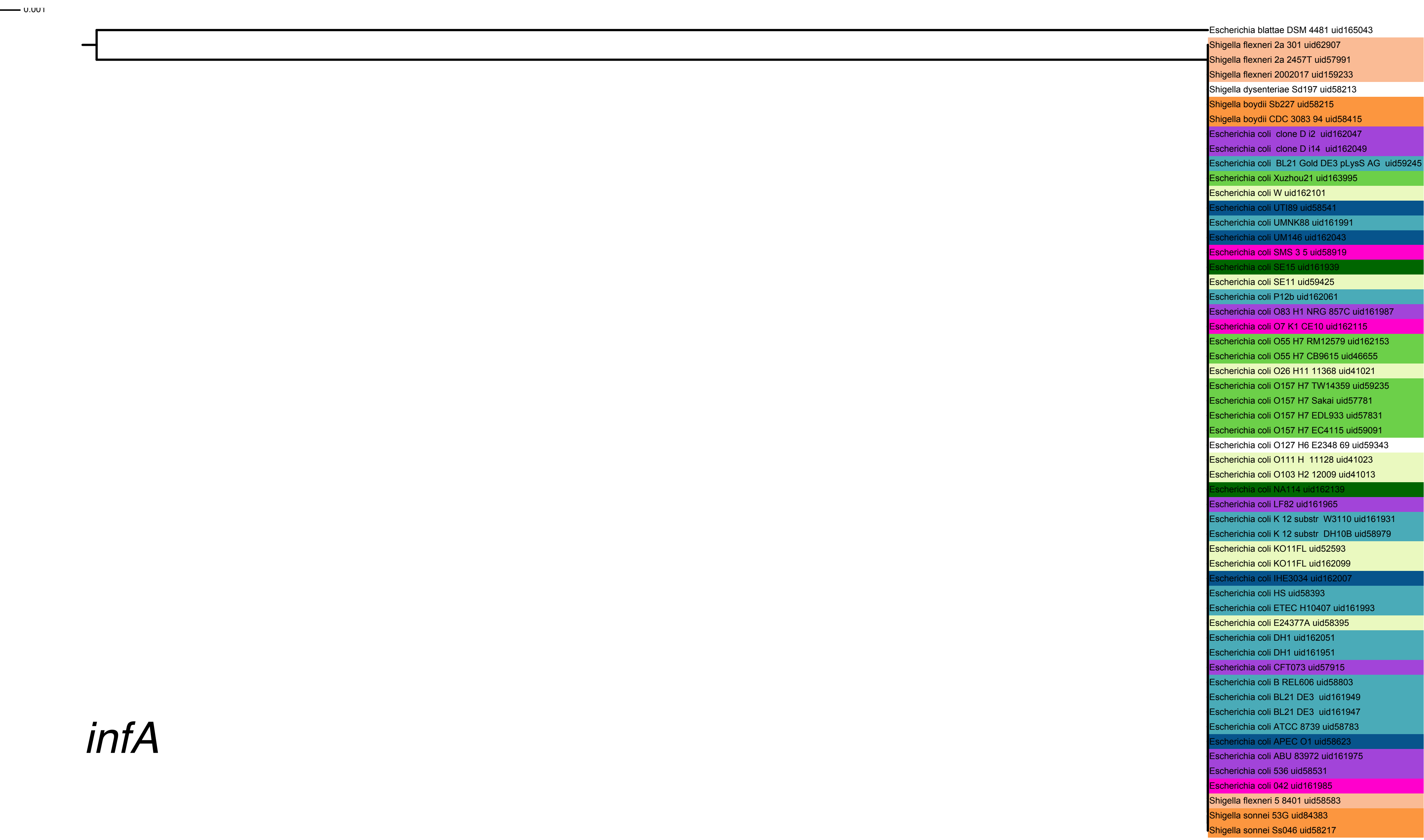

0.001
Escherichia blattae DSM 4481 uid165043
Shigella flexneri 2a 301 uid62907
Shigella flexneri 2a 2457T uid57991
Shigella flexneri 2002017 uid159233
Shigella dysenteriae Sd197 uid58213
Shigella boydii Sb227 uid58215
Shigella boydii CDC 3083 94 uid58415
Escherichia coli clone D i2 uid162047
Escherichia coli clone D i14 uid162049
Escherichia coli BL21 Gold DE3 pLysS AG uid59245
Escherichia coli Xuzhou21 uid163995
Escherichia coli W uid162101
Escherichia coli UTI89 uid58541
Escherichia coli UMNK88 uid161991
Escherichia coli UM146 uid162043
Escherichia coli SMS 3 5 uid58919
Escherichia coli SE15 uid161939
Escherichia coli SE11 uid59425
Escherichia coli P12b uid162061
Escherichia coli O83 H1 NRG 857C uid161987
Escherichia coli O7 K1 CE10 uid162115
Escherichia coli O55 H7 RM12579 uid162153
Escherichia coli O55 H7 CB9615 uid46655
Escherichia coli O26 H11 11368 uid41021
Escherichia coli O157 H7 TW14359 uid59235
Escherichia coli O157 H7 Sakai uid57781
Escherichia coli O157 H7 EDL933 uid57831
Escherichia coli O157 H7 EC4115 uid59091
Escherichia coli O127 H6 E2348 69 uid59343
Escherichia coli O111 H 11128 uid41023
Escherichia coli O103 H2 12009 uid41013
Escherichia coli NA114 uid162139
Escherichia coli LF82 uid161965
Escherichia coli K 12 substr W3110 uid161931
Escherichia coli K 12 substr DH10B uid58979
Escherichia coli KO11FL uid52593
Escherichia coli KO11FL uid162099
Escherichia coli IHE3034 uid162007
Escherichia coli HS uid58393
Escherichia coli ETEC H10407 uid161993
Escherichia coli E24377A uid58395
Escherichia coli DH1 uid162051
Escherichia coli DH1 uid161951
Escherichia coli CFT073 uid57915
Escherichia coli B REL606 uid58803
Escherichia coli BL21 DE3 uid161949
Escherichia coli BL21 DE3 uid161947
Escherichia coli ATCC 8739 uid58783
Escherichia coli APEC O1 uid58623
Escherichia coli ABU 83972 uid161975
Escherichia coli 536 uid58531
Escherichia coli 042 uid161985
Shigella flexneri 5 8401 uid58583
Shigella sonnei 53G uid84383
Shigella sonnei Ss046 uid58217

infA

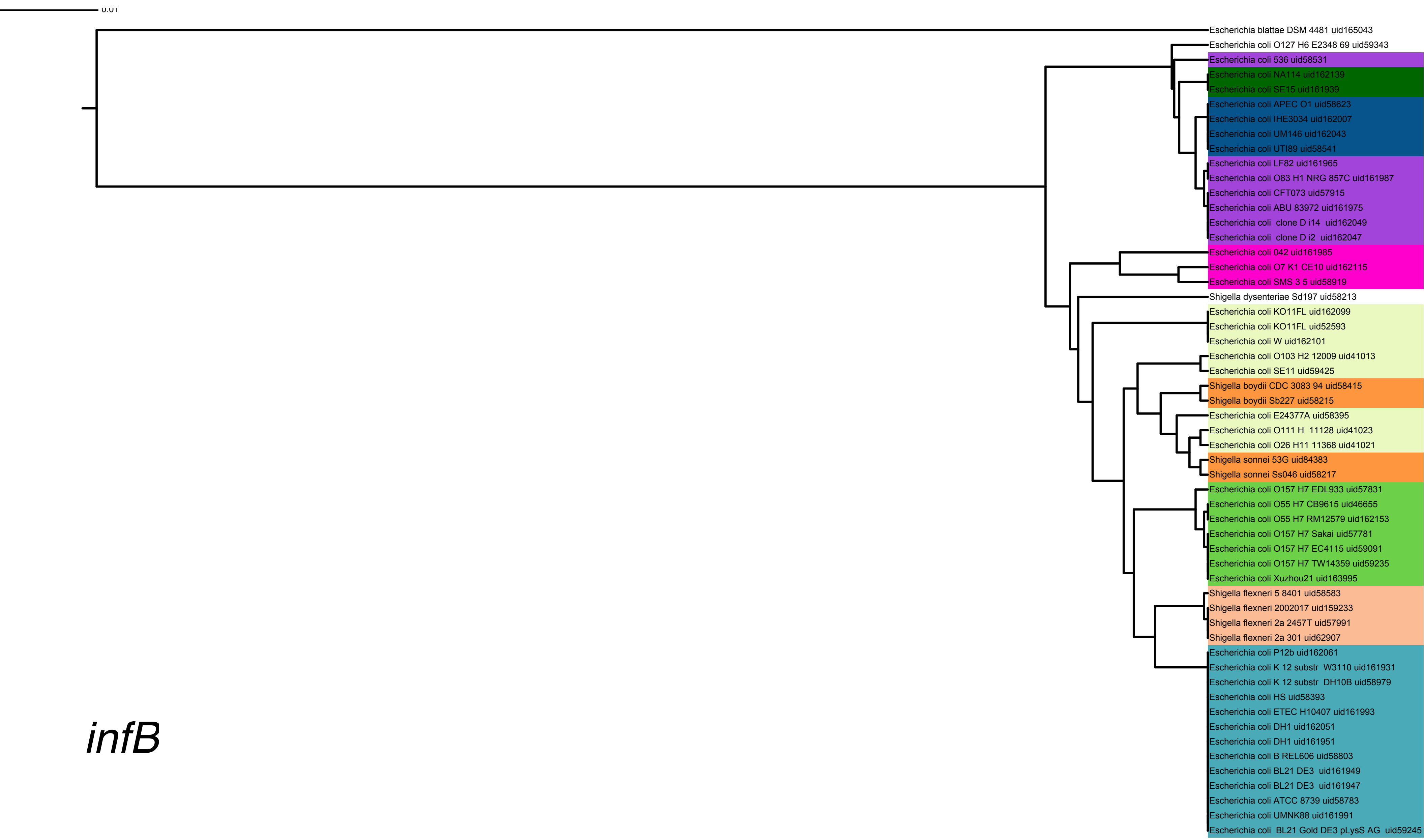

0.01
Escherichia blattae DSM 4481 uid165043
Escherichia coli O127 H6 E2348 69 uid59343
Escherichia coli 536 uid58531
Escherichia coli NA114 uid162139
Escherichia coli SE15 uid161939
Escherichia coli APEC O1 uid58623
Escherichia coli IHE3034 uid162007
Escherichia coli UM146 uid162043
Escherichia coli UTI89 uid58541
Escherichia coli LF82 uid161965
Escherichia coli O83 H1 NRG 857C uid161987
Escherichia coli CFT073 uid57915
Escherichia coli ABU 83972 uid161975
Escherichia coli clone D i14 uid162049
Escherichia coli clone D i2 uid162047
Escherichia coli 042 uid161985
Escherichia coli O7 K1 CE10 uid162115
Escherichia coli SMS 3 5 uid58919
Shigella dysenteriae Sd197 uid58213
Escherichia coli KO11FL uid162099
Escherichia coli KO11FL uid52593
Escherichia coli W uid162101
Escherichia coli O103 H2 12009 uid41013
Escherichia coli SE11 uid59425
Shigella boydii CDC 3083 94 uid58415
Shigella boydii Sb227 uid58215
Escherichia coli E24377A uid58395
Escherichia coli O111 H 11128 uid41023
Escherichia coli O26 H11 11368 uid41021
Shigella sonnei 53G uid84383
Shigella sonnei Ss046 uid58217
Escherichia coli O157 H7 EDL933 uid57831
Escherichia coli O55 H7 CB9615 uid46655
Escherichia coli O55 H7 RM12579 uid162153
Escherichia coli O157 H7 Sakai uid57781
Escherichia coli O157 H7 EC4115 uid59091
Escherichia coli O157 H7 TW14359 uid59235
Escherichia coli Xuzhou21 uid163995
Shigella flexneri 5 8401 uid58583
Shigella flexneri 2002017 uid159233
Shigella flexneri 2a 2457T uid57991
Shigella flexneri 2a 301 uid62907
Escherichia coli P12b uid162061
Escherichia coli K 12 substr W3110 uid161931
Escherichia coli K 12 substr DH10B uid58979
Escherichia coli HS uid58393
Escherichia coli ETEC H10407 uid161993
Escherichia coli DH1 uid162051
Escherichia coli DH1 uid161951
Escherichia coli B REL606 uid58803
Escherichia coli BL21 DE3 uid161949
Escherichia coli BL21 DE3 uid161947
Escherichia coli ATCC 8739 uid58783
Escherichia coli UMNK88 uid161991
Escherichia coli BL21 Gold DE3 pLysS AG uid59245
infB

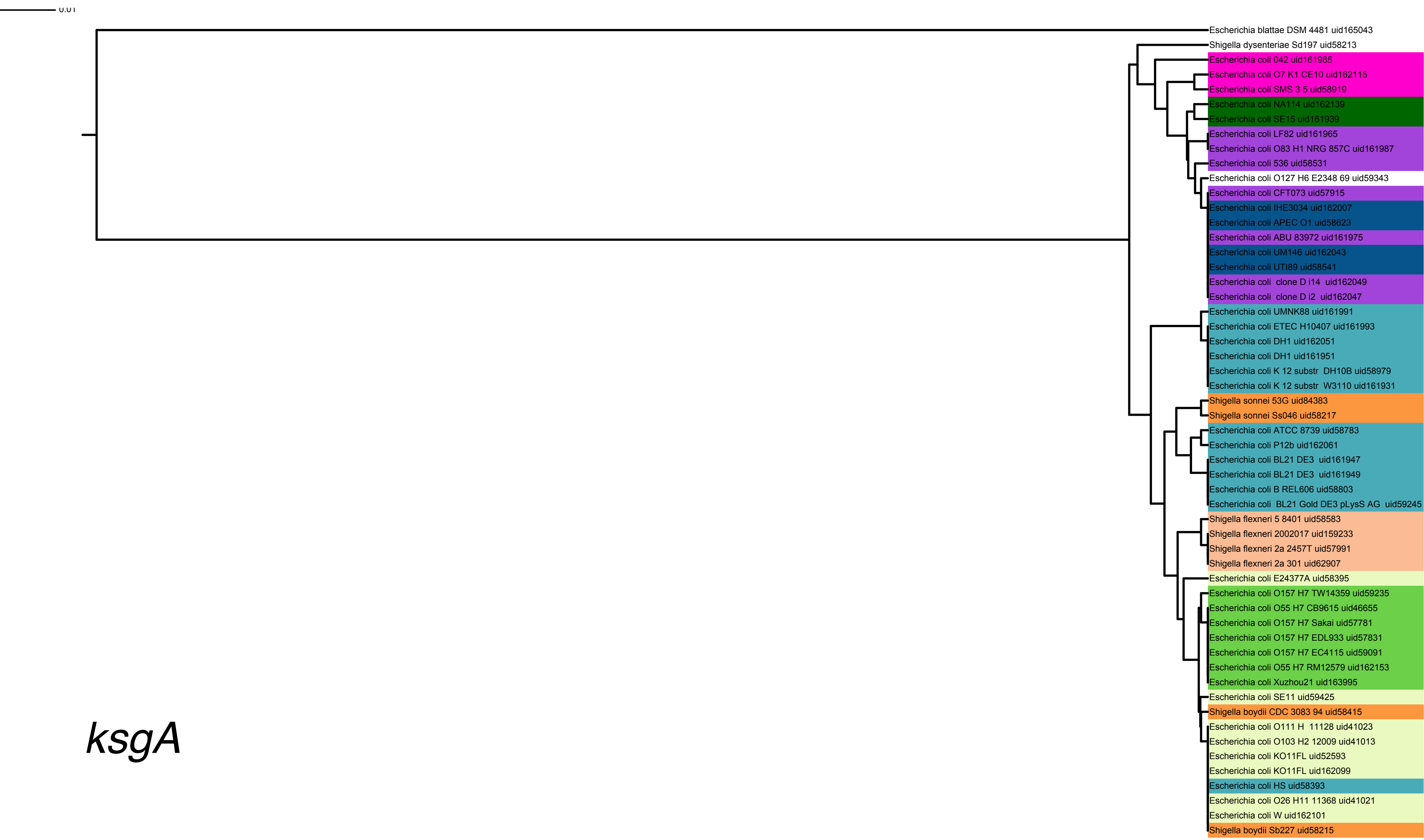
0.01
Escherichia blattae DSM 4481 uid165043
Shigella dysenteriae Sd197 uid58213
Escherichia coli 042 uid161985
Escherichia coli O7 K1 CE10 uid162115
Escherichia coli SMS 3 5 uid58919
Escherichia coli NA114 uid162139
Escherichia coli SE15 uid161939
Escherichia coli LF82 uid161965
Escherichia coli O83 H1 NRG 857C uid161987
Escherichia coli 536 uid58531
Escherichia coli O127 H6 E2348 69 uid59343
Escherichia coli CFT073 uid57915
Escherichia coli IHE3034 uid162007
Escherichia coli APEC O1 uid58623
Escherichia coli ABU 83972 uid161975
Escherichia coli UM146 uid162043
Escherichia coli UTI89 uid58541
Escherichia coli clone D i14 uid162049
Escherichia coli clone D i2 uid162047
Escherichia coli UMNK88 uid161991
Escherichia coli ETEC H10407 uid161993
Escherichia coli DH1 uid162051
Escherichia coli DH1 uid161951
Escherichia coli K 12 substr DH10B uid58979
Escherichia coli K 12 substr W3110 uid161931
Shigella sonnei 53G uid84383
Shigella sonnei Ss046 uid58217
Escherichia coli ATCC 8739 uid58783
Escherichia coli P12b uid162061
Escherichia coli BL21 DE3 uid161947
Escherichia coli BL21 DE3 uid161949
Escherichia coli B REL606 uid58803
Escherichia coli BL21 Gold DE3 pLysS AG uid59245
Shigella flexneri 5 8401 uid58583
Shigella flexneri 2002017 uid159233
Shigella flexneri 2a 2457T uid57991
Shigella flexneri 2a 301 uid62907
Escherichia coli E24377A uid58395
Escherichia coli O157 H7 TW14359 uid59235
Escherichia coli O55 H7 CB9615 uid46655
Escherichia coli O157 H7 Sakai uid57781
Escherichia coli O157 H7 EDL933 uid57831
Escherichia coli O157 H7 EC4115 uid59091
Escherichia coli O55 H7 RM12579 uid162153
Escherichia coli Xuzhou21 uid163995
Escherichia coli SE11 uid59425
Shigella boydii CDC 3083 94 uid58415
Escherichia coli O111 H 11128 uid41023
Escherichia coli O103 H2 12009 uid41013
Escherichia coli KO11FL uid52593
Escherichia coli KO11FL uid162099
Escherichia coli HS uid58393
Escherichia coli O26 H11 11368 uid41021
Escherichia coli W uid162101
Shigella boydii Sb227 uid58215
ksgA

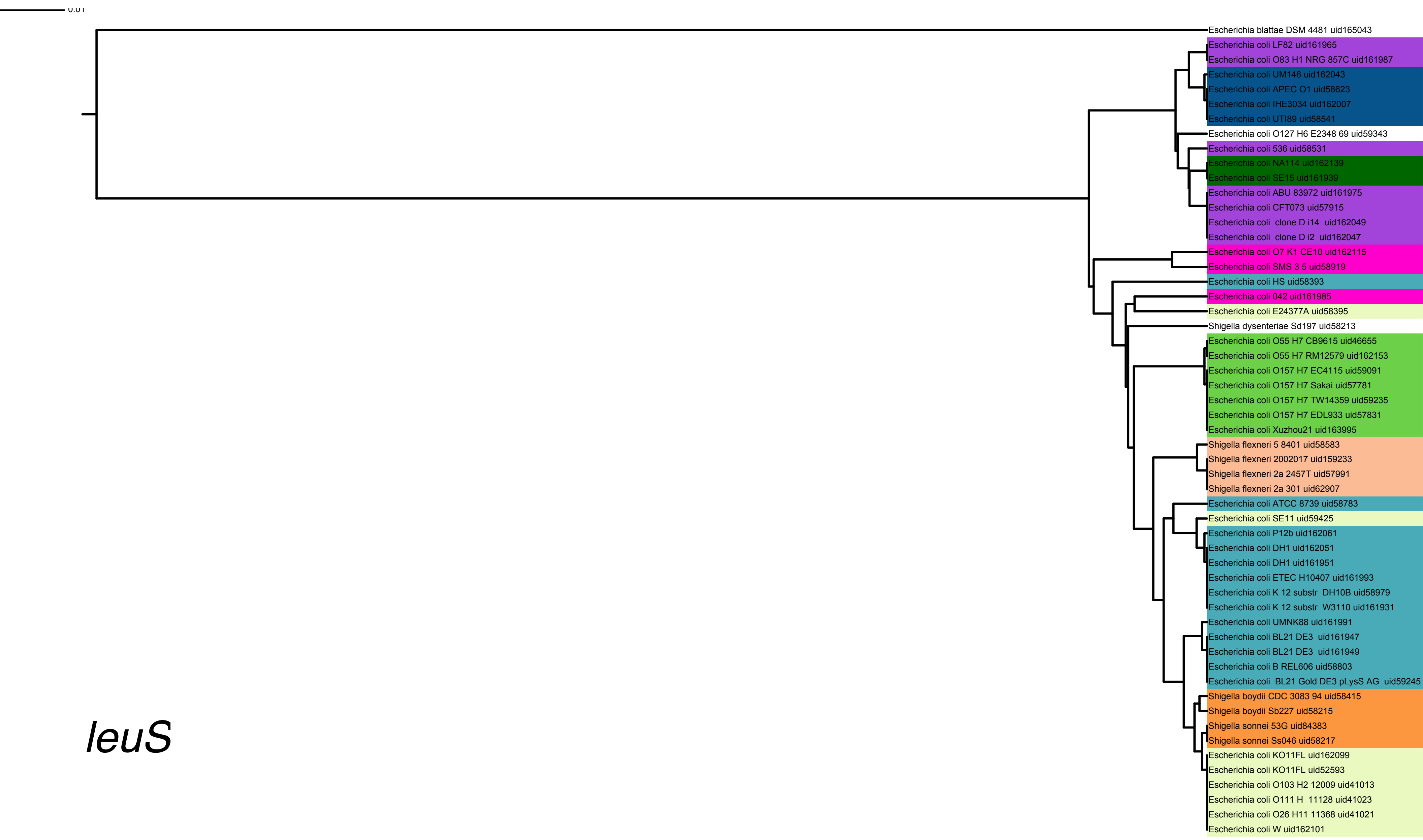
0.01
Escherichia blattae DSM 4481 uid165043
Escherichia coli LF82 uid161965
Escherichia coli O83 H1 NRG 857C uid161987
Escherichia coli UM146 uid162043
Escherichia coli APEC O1 uid58623
Escherichia coli IHE3034 uid162007
Escherichia coli UTI89 uid58541
Escherichia coli O127 H6 E2348 69 uid59343
Escherichia coli 536 uid58531
Escherichia coli NA114 uid162139
Escherichia coli SE15 uid161939
Escherichia coli ABU 83972 uid161975
Escherichia coli CFT073 uid57915
Escherichia coli clone D i14 uid162049
Escherichia coli clone D i2 uid162047
Escherichia coli O7 K1 CE10 uid162115
Escherichia coli SMS 3 5 uid58919
Escherichia coli HS uid58393
Escherichia coli 042 uid161985
Escherichia coli E24377A uid58395
Shigella dysenteriae Sd197 uid58213
Escherichia coli O55 H7 CB9615 uid46655
Escherichia coli O55 H7 RM12579 uid162153
Escherichia coli O157 H7 EC4115 uid59091
Escherichia coli O157 H7 Sakai uid57781
Escherichia coli O157 H7 TW14359 uid59235
Escherichia coli O157 H7 EDL933 uid57831
Escherichia coli Xuzhou21 uid163995
Shigella flexneri 5 8401 uid58583
Shigella flexneri 2002017 uid159233
Shigella flexneri 2a 2457T uid57991
Shigella flexneri 2a 301 uid62907
Escherichia coli ATCC 8739 uid58783
Escherichia coli SE11 uid59425
Escherichia coli P12b uid162061
Escherichia coli DH1 uid162051
Escherichia coli DH1 uid161951
Escherichia coli ETEC H10407 uid161993
Escherichia coli K 12 substr DH10B uid58979
Escherichia coli K 12 substr W3110 uid161931
Escherichia coli UMNK88 uid161991
Escherichia coli BL21 DE3 uid161947
Escherichia coli BL21 DE3 uid161949
Escherichia coli B REL606 uid58803
Escherichia coli BL21 Gold DE3 pLysS AG uid59245
Shigella boydii CDC 3083 94 uid58415
Shigella boydii Sb227 uid58215
Shigella sonnei 53G uid84383
Shigella sonnei Ss046 uid58217
Escherichia coli KO11FL uid162099
Escherichia coli KO11FL uid52593
Escherichia coli O103 H2 12009 uid41013
Escherichia coli O111 H 11128 uid41023
Escherichia coli O26 H11 11368 uid41021
Escherichia coli W uid162101
leuS

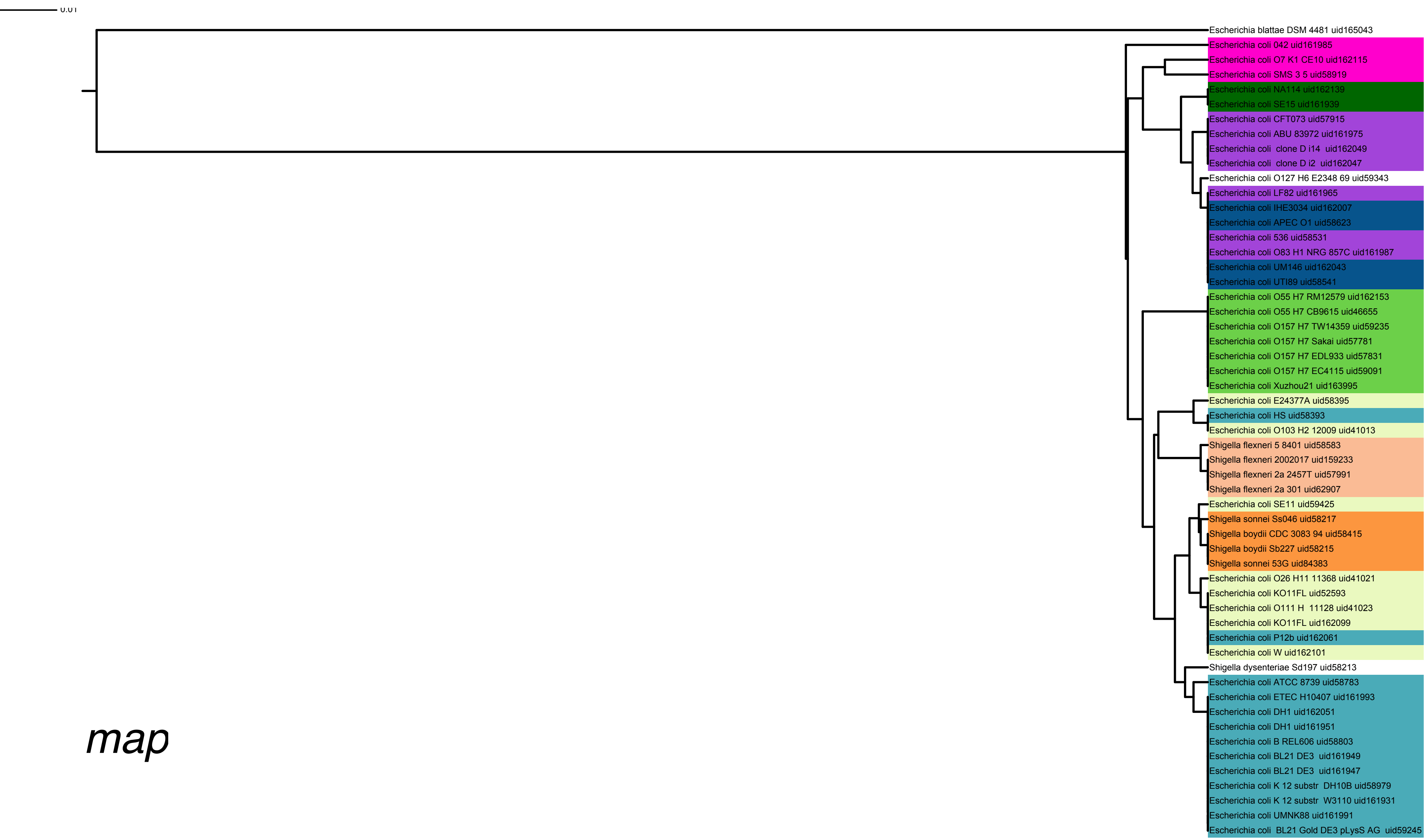
0.01
Escherichia blattae DSM 4481 uid165043
Escherichia coli 042 uid161985
Escherichia coli O7 K1 CE10 uid162115
Escherichia coli SMS 3 5 uid58919
Escherichia coli NA114 uid162139
Escherichia coli SE15 uid161939
Escherichia coli CFT073 uid57915
Escherichia coli ABU 83972 uid161975
Escherichia coli clone D i14 uid162049
Escherichia coli clone D i2 uid162047
Escherichia coli O127 H6 E2348 69 uid59343
Escherichia coli LF82 uid161965
Escherichia coli IHE3034 uid162007
Escherichia coli APEC O1 uid58623
Escherichia coli 536 uid58531
Escherichia coli O83 H1 NRG 857C uid161987
Escherichia coli UM146 uid162043
Escherichia coli UTI89 uid58541
Escherichia coli O55 H7 RM12579 uid162153
Escherichia coli O55 H7 CB9615 uid46655
Escherichia coli O157 H7 TW14359 uid59235
Escherichia coli O157 H7 Sakai uid57781
Escherichia coli O157 H7 EDL933 uid57831
Escherichia coli O157 H7 EC4115 uid59091
Escherichia coli Xuzhou21 uid163995
Escherichia coli E24377A uid58395
Escherichia coli HS uid58393
Escherichia coli O103 H2 12009 uid41013
Shigella flexneri 5 8401 uid58583
Shigella flexneri 2002017 uid159233
Shigella flexneri 2a 2457T uid57991
Shigella flexneri 2a 301 uid62907
Escherichia coli SE11 uid59425
Shigella sonnei Ss046 uid58217
Shigella boydii CDC 3083 94 uid58415
Shigella boydii Sb227 uid58215
Shigella sonnei 53G uid84383
Escherichia coli O26 H11 11368 uid41021
Escherichia coli KO11FL uid52593
Escherichia coli O111 H 11128 uid41023
Escherichia coli KO11FL uid162099
Escherichia coli P12b uid162061
Escherichia coli W uid162101
Shigella dysenteriae Sd197 uid58213
Escherichia coli ATCC 8739 uid58783
Escherichia coli ETEC H10407 uid161993
Escherichia coli DH1 uid162051
Escherichia coli DH1 uid161951
Escherichia coli B REL606 uid58803
Escherichia coli BL21 DE3 uid161949
Escherichia coli BL21 DE3 uid161947
Escherichia coli K 12 substr DH10B uid58979
Escherichia coli K 12 substr W3110 uid161931
Escherichia coli UMNK88 uid161991
Escherichia coli BL21 Gold DE3 pLysS AG uid59245
map

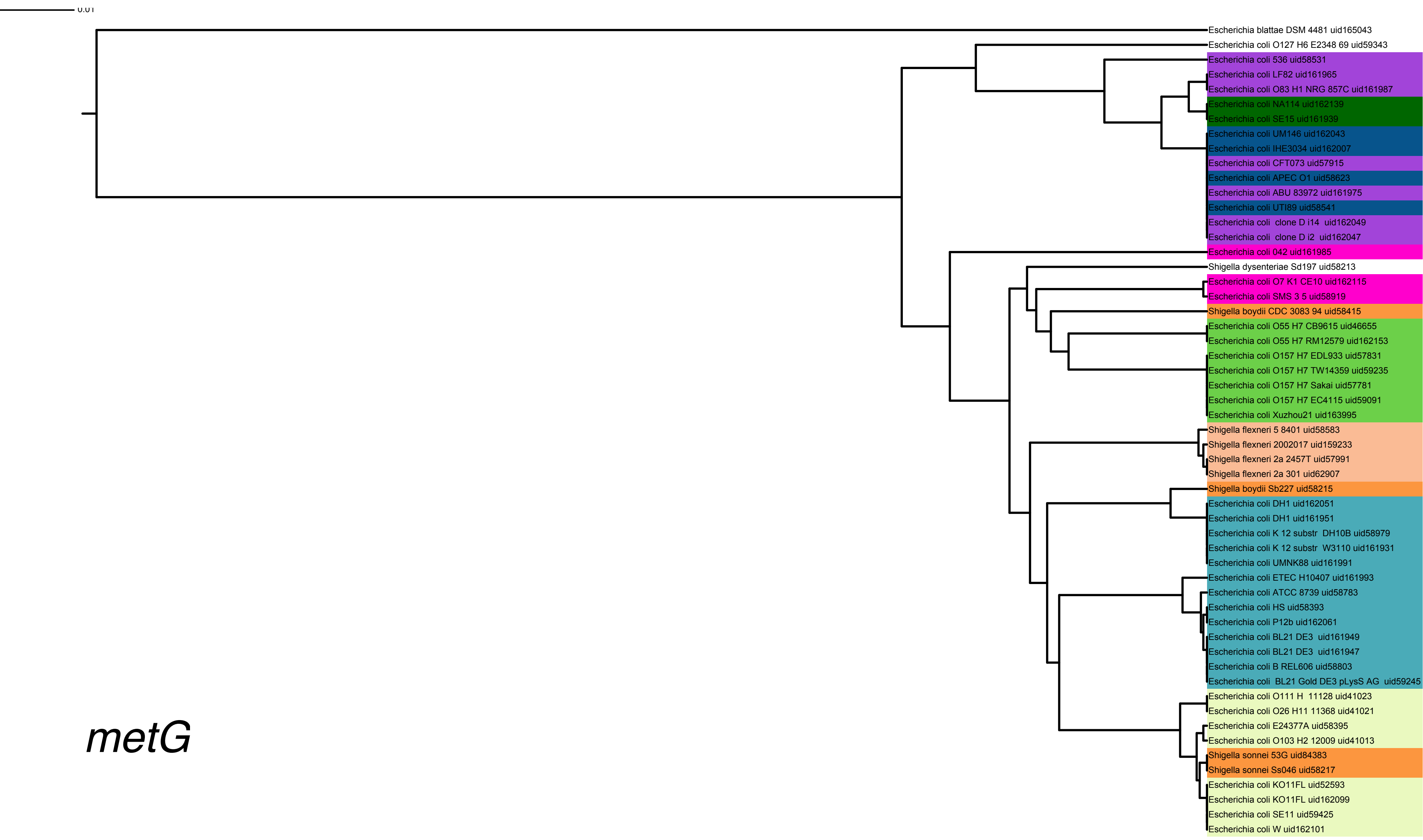
0.01
Escherichia blattae DSM 4481 uid165043
Escherichia coli O127 H6 E2348 69 uid59343
Escherichia coli 536 uid58531
Escherichia coli LF82 uid161965
Escherichia coli O83 H1 NRG 857C uid161987
Escherichia coli NA114 uid162139
Escherichia coli SE15 uid161939
Escherichia coli UM146 uid162043
Escherichia coli IHE3034 uid162007
Escherichia coli CFT073 uid57915
Escherichia coli APEC O1 uid58623
Escherichia coli ABU 83972 uid161975
Escherichia coli UTI89 uid58541
Escherichia coli clone D i14 uid162049
Escherichia coli clone D i2 uid162047
Escherichia coli 042 uid161985
Shigella dysenteriae Sd197 uid58213
Escherichia coli O7 K1 CE10 uid162115
Escherichia coli SMS 3 5 uid58919
Shigella boydii CDC 3083 94 uid58415
Escherichia coli O55 H7 CB9615 uid46655
Escherichia coli O55 H7 RM12579 uid162153
Escherichia coli O157 H7 EDL933 uid57831
Escherichia coli O157 H7 TW14359 uid59235
Escherichia coli O157 H7 Sakai uid57781
Escherichia coli O157 H7 EC4115 uid59091
Escherichia coli Xuzhou21 uid163995
Shigella flexneri 5 8401 uid58583
Shigella flexneri 2002017 uid159233
Shigella flexneri 2a 2457T uid57991
Shigella flexneri 2a 301 uid62907
Shigella boydii Sb227 uid58215
Escherichia coli DH1 uid162051
Escherichia coli DH1 uid161951
Escherichia coli K 12 substr DH10B uid58979
Escherichia coli K 12 substr W3110 uid161931
Escherichia coli UMNK88 uid161991
Escherichia coli ETEC H10407 uid161993
Escherichia coli ATCC 8739 uid58783
Escherichia coli HS uid58393
Escherichia coli P12b uid162061
Escherichia coli BL21 DE3 uid161949
Escherichia coli BL21 DE3 uid161947
Escherichia coli B REL606 uid58803
Escherichia coli BL21 Gold DE3 pLysS AG uid59245
Escherichia coli O111 H 11128 uid41023
Escherichia coli O26 H11 11368 uid41021
Escherichia coli E24377A uid58395
Escherichia coli O103 H2 12009 uid41013
Shigella sonnei 53G uid84383
Shigella sonnei Ss046 uid58217
Escherichia coli KO11FL uid52593
Escherichia coli KO11FL uid162099
Escherichia coli SE11 uid59425
Escherichia coli W uid162101
metG

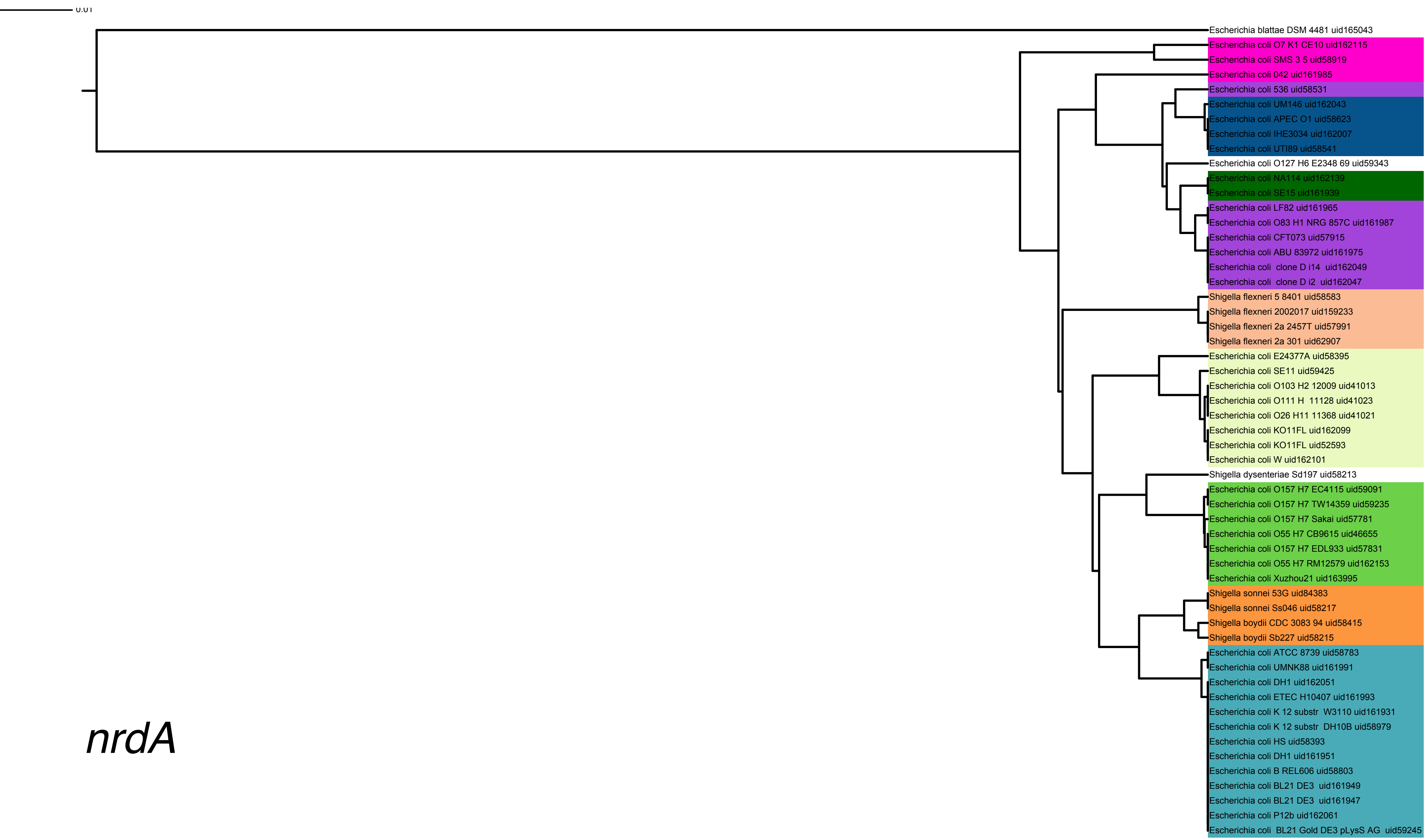
0.01
Escherichia blattae DSM 4481 uid165043
Escherichia coli O7 K1 CE10 uid162115
Escherichia coli SMS 3 5 uid58919
Escherichia coli 042 uid161985
Escherichia coli 536 uid58531
Escherichia coli UM146 uid162043
Escherichia coli APEC O1 uid58623
Escherichia coli IHE3034 uid162007
Escherichia coli UTI89 uid58541
Escherichia coli O127 H6 E2348 69 uid59343
Escherichia coli NA114 uid162139
Escherichia coli SE15 uid161939
Escherichia coli LF82 uid161965
Escherichia coli O83 H1 NRG 857C uid161987
Escherichia coli CFT073 uid57915
Escherichia coli ABU 83972 uid161975
Escherichia coli clone D i14 uid162049
Escherichia coli clone D i2 uid162047
Shigella flexneri 5 8401 uid58583
Shigella flexneri 2002017 uid159233
Shigella flexneri 2a 2457T uid57991
Shigella flexneri 2a 301 uid62907
Escherichia coli E24377A uid58395
Escherichia coli SE11 uid59425
Escherichia coli O103 H2 12009 uid41013
Escherichia coli O111 H 11128 uid41023
Escherichia coli O26 H11 11368 uid41021
Escherichia coli KO11FL uid162099
Escherichia coli KO11FL uid52593
Escherichia coli W uid162101
Shigella dysenteriae Sd197 uid58213
Escherichia coli O157 H7 EC4115 uid59091
Escherichia coli O157 H7 TW14359 uid59235
Escherichia coli O157 H7 Sakai uid57781
Escherichia coli O55 H7 CB9615 uid46655
Escherichia coli O157 H7 EDL933 uid57831
Escherichia coli O55 H7 RM12579 uid162153
Escherichia coli Xuzhou21 uid163995
Shigella sonnei 53G uid84383
Shigella sonnei Ss046 uid58217
Shigella boydii CDC 3083 94 uid58415
Shigella boydii Sb227 uid58215
Escherichia coli ATCC 8739 uid58783
Escherichia coli UMNK88 uid161991
Escherichia coli DH1 uid162051
Escherichia coli ETEC H10407 uid161993
Escherichia coli K 12 substr W3110 uid161931
Escherichia coli K 12 substr DH10B uid58979
Escherichia coli HS uid58393
Escherichia coli DH1 uid161951
Escherichia coli B REL606 uid58803
Escherichia coli BL21 DE3 uid161949
Escherichia coli BL21 DE3 uid161947
Escherichia coli P12b uid162061
Escherichia coli BL21 Gold DE3 pLysS AG uid59245
nrdA

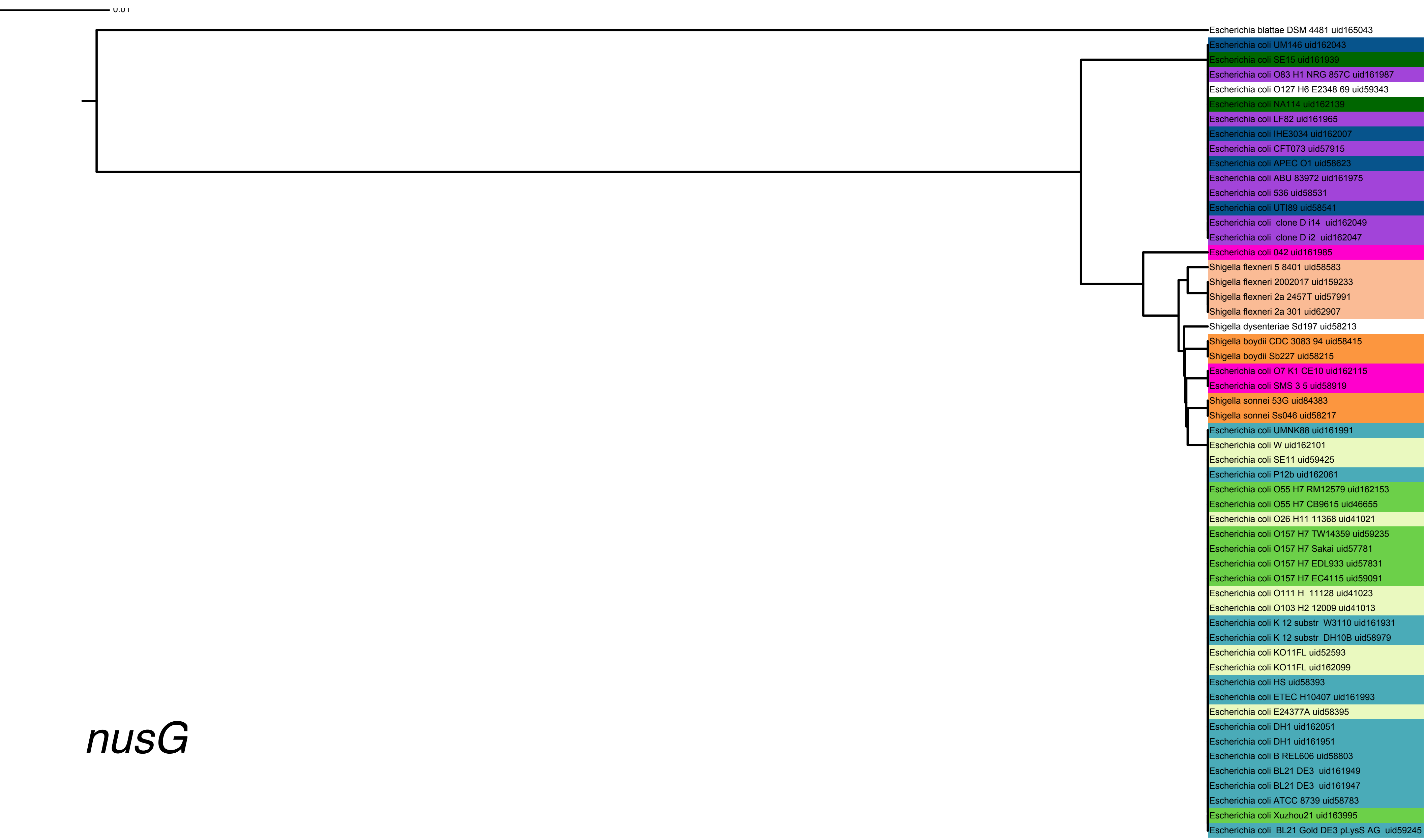
0.01
Escherichia blattae DSM 4481 uid165043
Escherichia coli UM146 uid162043
Escherichia coli SE15 uid161939
Escherichia coli O83 H1 NRG 857C uid161987
Escherichia coli O127 H6 E2348 69 uid59343
Escherichia coli NA114 uid162139
Escherichia coli LF82 uid161965
Escherichia coli IHE3034 uid162007
Escherichia coli CFT073 uid57915
Escherichia coli APEC O1 uid58623
Escherichia coli ABU 83972 uid161975
Escherichia coli 536 uid58531
Escherichia coli UTI89 uid58541
Escherichia coli clone D i14 uid162049
Escherichia coli clone D i2 uid162047
Escherichia coli 042 uid161985
Shigella flexneri 5 8401 uid58583
Shigella flexneri 2002017 uid159233
Shigella flexneri 2a 2457T uid57991
Shigella flexneri 2a 301 uid62907
Shigella dysenteriae Sd197 uid58213
Shigella boydii CDC 3083 94 uid58415
Shigella boydii Sb227 uid58215
Escherichia coli O7 K1 CE10 uid162115
Escherichia coli SMS 3 5 uid58919
Shigella sonnei 53G uid84383
Shigella sonnei Ss046 uid58217
Escherichia coli UMNK88 uid161991
Escherichia coli W uid162101
Escherichia coli SE11 uid59425
Escherichia coli P12b uid162061
Escherichia coli O55 H7 RM12579 uid162153
Escherichia coli O55 H7 CB9615 uid46655
Escherichia coli O26 H11 11368 uid41021
Escherichia coli O157 H7 TW14359 uid59235
Escherichia coli O157 H7 Sakai uid57781
Escherichia coli O157 H7 EDL933 uid57831
Escherichia coli O157 H7 EC4115 uid59091
Escherichia coli O111 H 11128 uid41023
Escherichia coli O103 H2 12009 uid41013
Escherichia coli K 12 substr W3110 uid161931
Escherichia coli K 12 substr DH10B uid58979
Escherichia coli KO11FL uid52593
Escherichia coli KO11FL uid162099
Escherichia coli HS uid58393
Escherichia coli ETEC H10407 uid161993
Escherichia coli E24377A uid58395
Escherichia coli DH1 uid162051
Escherichia coli DH1 uid161951
Escherichia coli B REL606 uid58803
Escherichia coli BL21 DE3 uid161949
Escherichia coli BL21 DE3 uid161947
Escherichia coli ATCC 8739 uid58783
Escherichia coli Xuzhou21 uid163995
Escherichia coli BL21 Gold DE3 pLysS AG uid59245

nusG

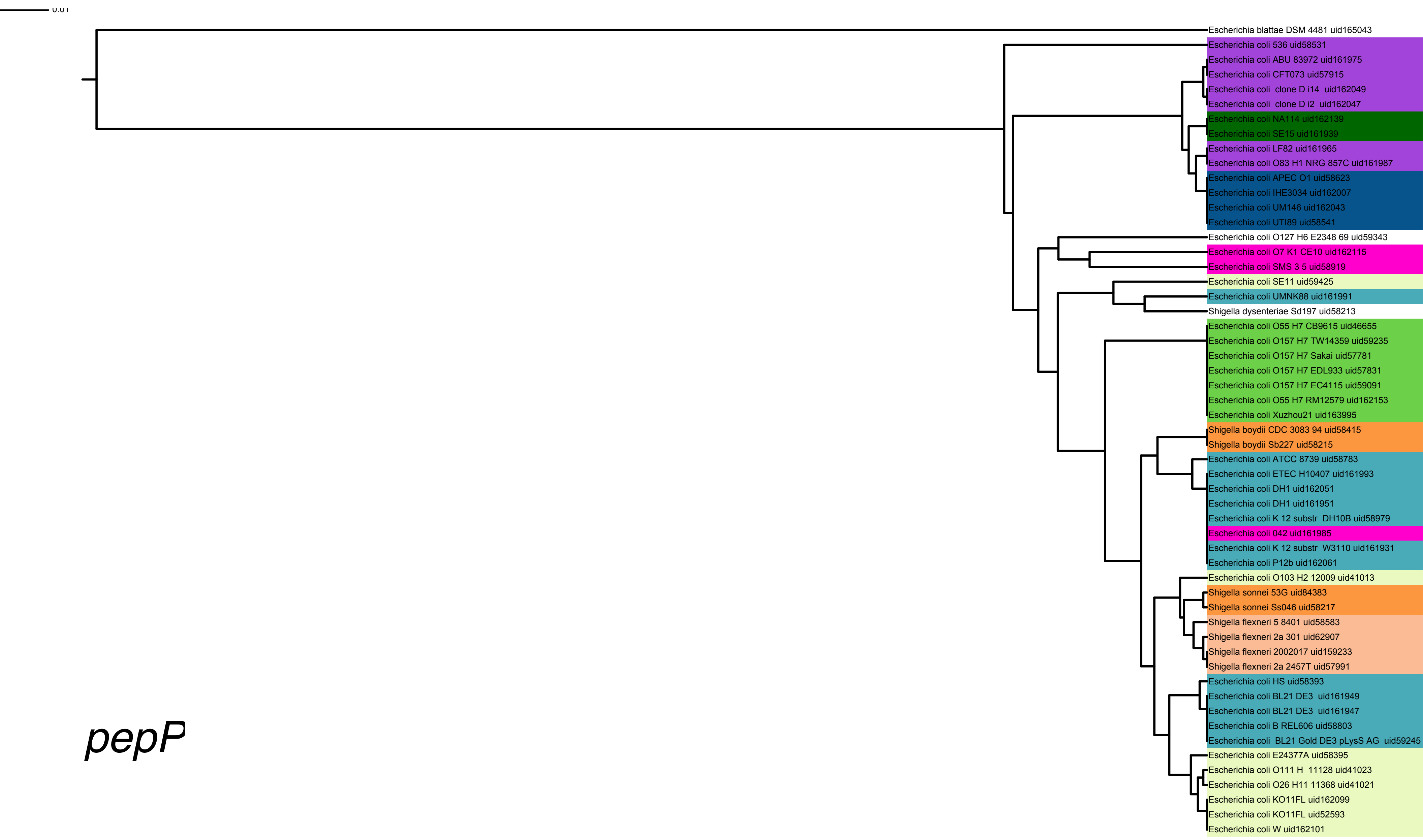
0.01
Escherichia blattae DSM 4481 uid165043
Escherichia coli 536 uid58531
Escherichia coli ABU 83972 uid161975
Escherichia coli CFT073 uid57915
Escherichia coli clone D i14 uid162049
Escherichia coli clone D i2 uid162047
Escherichia coli NA114 uid162139
Escherichia coli SE15 uid161939
Escherichia coli LF82 uid161965
Escherichia coli O83 H1 NRG 857C uid161987
Escherichia coli APEC O1 uid58623
Escherichia coli IHE3034 uid162007
Escherichia coli UM146 uid162043
Escherichia coli UTI89 uid58541
Escherichia coli O127 H6 E2348 69 uid59343
Escherichia coli O7 K1 CE10 uid162115
Escherichia coli SMS 3 5 uid58919
Escherichia coli SE11 uid59425
Escherichia coli UMNK88 uid161991
Shigella dysenteriae Sd197 uid58213
Escherichia coli O55 H7 CB9615 uid46655
Escherichia coli O157 H7 TW14359 uid59235
Escherichia coli O157 H7 Sakai uid57781
Escherichia coli O157 H7 EDL933 uid57831
Escherichia coli O157 H7 EC4115 uid59091
Escherichia coli O55 H7 RM12579 uid162153
Escherichia coli Xuzhou21 uid163995
Shigella boydii CDC 3083 94 uid58415
Shigella boydii Sb227 uid58215
Escherichia coli ATCC 8739 uid58783
Escherichia coli ETEC H10407 uid161993
Escherichia coli DH1 uid162051
Escherichia coli DH1 uid161951
Escherichia coli K 12 substr DH10B uid58979
Escherichia coli 042 uid161985
Escherichia coli K 12 substr W3110 uid161931
Escherichia coli P12b uid162061
Escherichia coli O103 H2 12009 uid41013
Shigella sonnei 53G uid84383
Shigella sonnei Ss046 uid58217
Shigella flexneri 5 8401 uid58583
Shigella flexneri 2a 301 uid62907
Shigella flexneri 2002017 uid159233
Shigella flexneri 2a 2457T uid57991
Escherichia coli HS uid58393
Escherichia coli BL21 DE3 uid161949
Escherichia coli BL21 DE3 uid161947
Escherichia coli B REL606 uid58803
Escherichia coli BL21 Gold DE3 pLysS AG uid59245
Escherichia coli E24377A uid58395
Escherichia coli O111 H 11128 uid41023
Escherichia coli O26 H11 11368 uid41021
Escherichia coli KO11FL uid162099
Escherichia coli KO11FL uid52593
Escherichia coli W uid162101
pepP

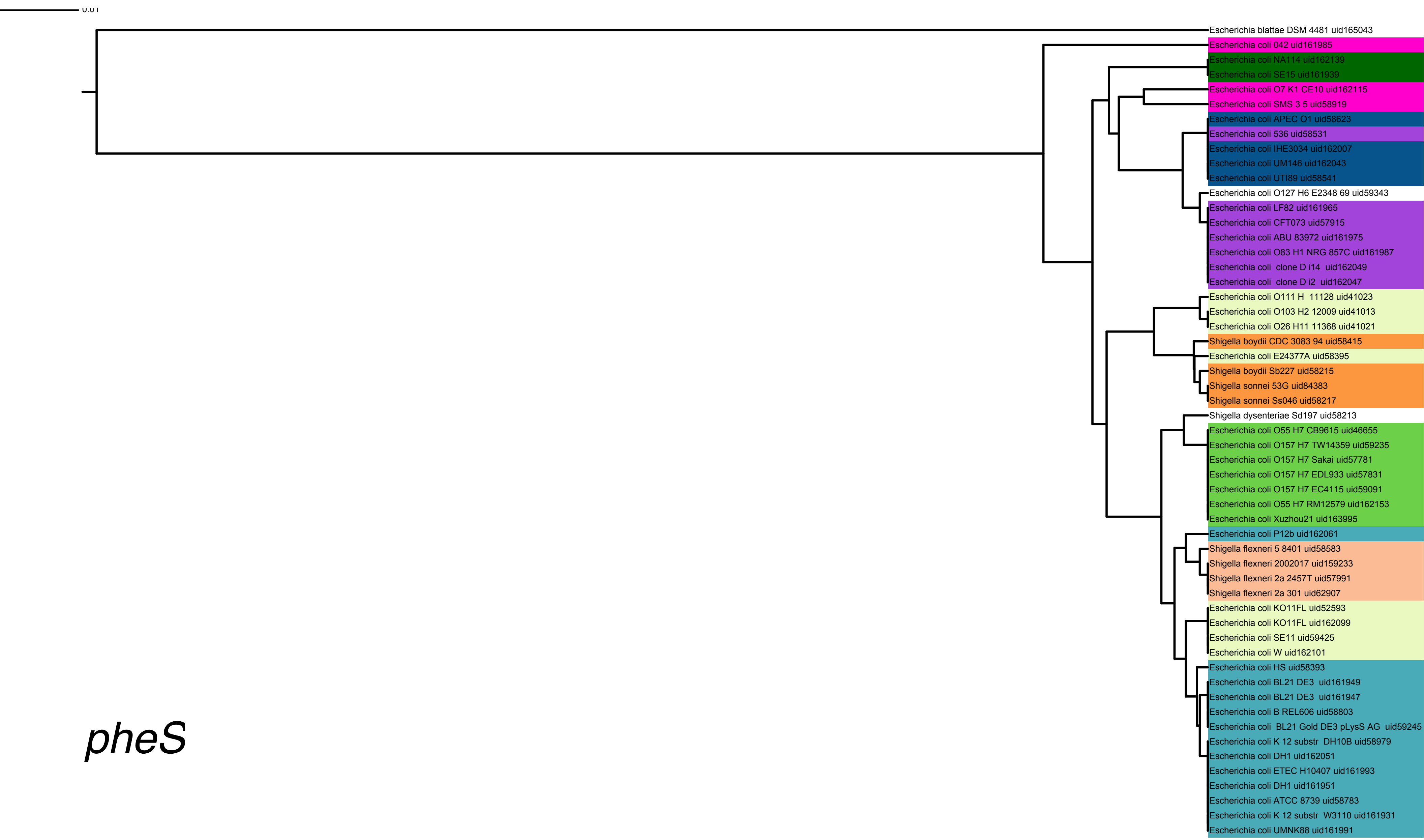
0.01
Escherichia blattae DSM 4481 uid165043
Escherichia coli 042 uid161985
Escherichia coli NA114 uid162139
Escherichia coli SE15 uid161939
Escherichia coli O7 K1 CE10 uid162115
Escherichia coli SMS 3 5 uid58919
Escherichia coli APEC O1 uid58623
Escherichia coli 536 uid58531
Escherichia coli IHE3034 uid162007
Escherichia coli UM146 uid162043
Escherichia coli UTI89 uid58541
Escherichia coli O127 H6 E2348 69 uid59343
Escherichia coli LF82 uid161965
Escherichia coli CFT073 uid57915
Escherichia coli ABU 83972 uid161975
Escherichia coli O83 H1 NRG 857C uid161987
Escherichia coli clone D i14 uid162049
Escherichia coli clone D i2 uid162047
Escherichia coli O111 H 11128 uid41023
Escherichia coli O103 H2 12009 uid41013
Escherichia coli O26 H11 11368 uid41021
Shigella boydii CDC 3083 94 uid58415
Escherichia coli E24377A uid58395
Shigella boydii Sb227 uid58215
Shigella sonnei 53G uid84383
Shigella sonnei Ss046 uid58217
Shigella dysenteriae Sd197 uid58213
Escherichia coli O55 H7 CB9615 uid46655
Escherichia coli O157 H7 TW14359 uid59235
Escherichia coli O157 H7 Sakai uid57781
Escherichia coli O157 H7 EDL933 uid57831
Escherichia coli O157 H7 EC4115 uid59091
Escherichia coli O55 H7 RM12579 uid162153
Escherichia coli Xuzhou21 uid163995
Escherichia coli P12b uid162061
Shigella flexneri 5 8401 uid58583
Shigella flexneri 2002017 uid159233
Shigella flexneri 2a 2457T uid57991
Shigella flexneri 2a 301 uid62907
Escherichia coli KO11FL uid52593
Escherichia coli KO11FL uid162099
Escherichia coli SE11 uid59425
Escherichia coli W uid162101
Escherichia coli HS uid58393
Escherichia coli BL21 DE3 uid161949
Escherichia coli BL21 DE3 uid161947
Escherichia coli B REL606 uid58803
Escherichia coli BL21 Gold DE3 pLysS AG uid59245
Escherichia coli K 12 substr DH10B uid58979
Escherichia coli DH1 uid162051
Escherichia coli ETEC H10407 uid161993
Escherichia coli DH1 uid161951
Escherichia coli ATCC 8739 uid58783
Escherichia coli K 12 substr W3110 uid161931
Escherichia coli UMNK88 uid161991
pheS

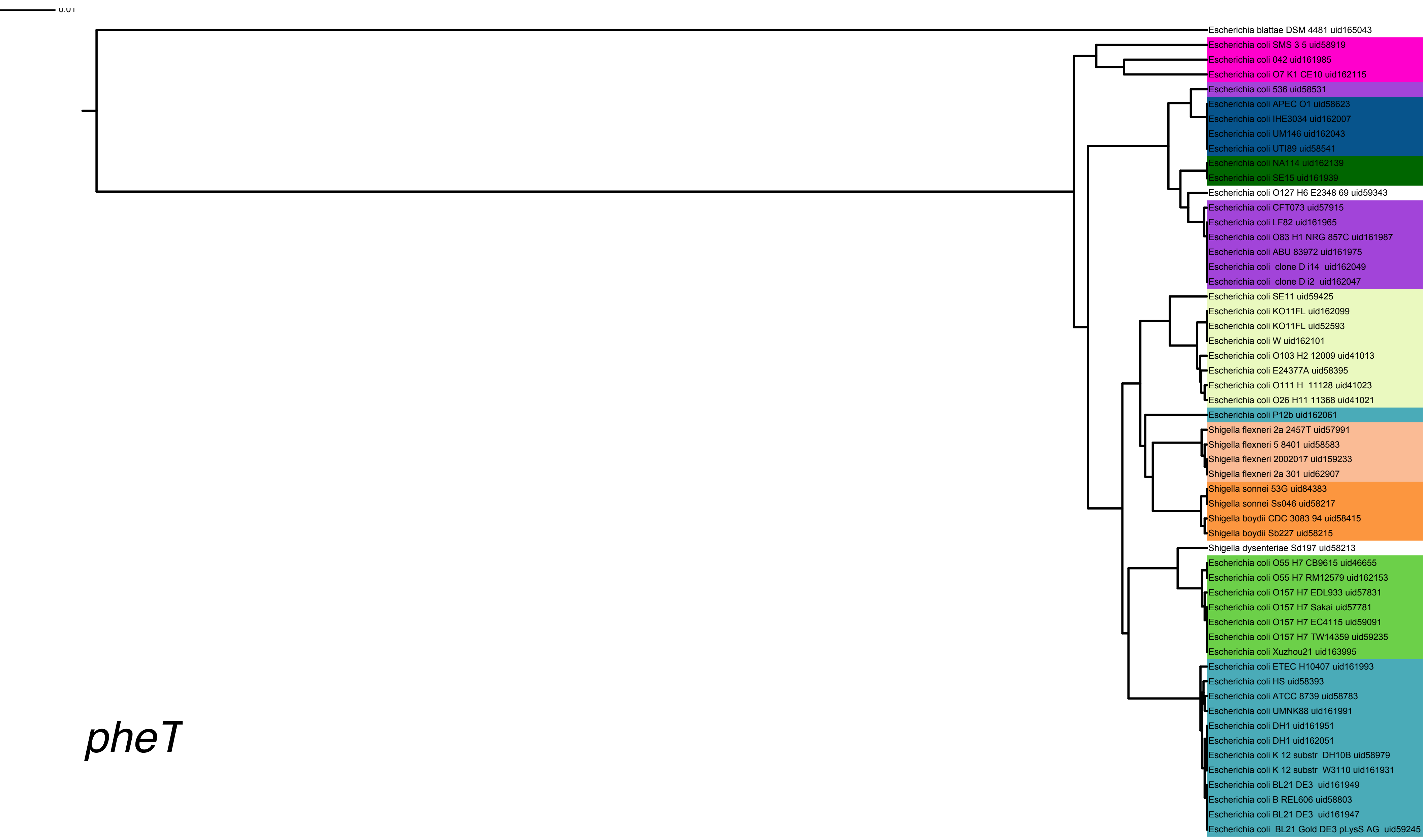

0.01
Escherichia blattae DSM 4481 uid165043
Escherichia coli SMS 3 5 uid58919
Escherichia coli 042 uid161985
Escherichia coli O7 K1 CE10 uid162115
Escherichia coli 536 uid58531
Escherichia coli APEC O1 uid58623
Escherichia coli IHE3034 uid162007
Escherichia coli UM146 uid162043
Escherichia coli UTI89 uid58541
Escherichia coli NA114 uid162139
Escherichia coli SE15 uid161939
Escherichia coli O127 H6 E2348 69 uid59343
Escherichia coli CFT073 uid57915
Escherichia coli LF82 uid161965
Escherichia coli O83 H1 NRG 857C uid161987
Escherichia coli ABU 83972 uid161975
Escherichia coli clone D i14 uid162049
Escherichia coli clone D i2 uid162047
Escherichia coli SE11 uid59425
Escherichia coli KO11FL uid162099
Escherichia coli KO11FL uid52593
Escherichia coli W uid162101
Escherichia coli O103 H2 12009 uid41013
Escherichia coli E24377A uid58395
Escherichia coli O111 H 11128 uid41023
Escherichia coli O26 H11 11368 uid41021
Escherichia coli P12b uid162061
Shigella flexneri 2a 2457T uid57991
Shigella flexneri 5 8401 uid58583
Shigella flexneri 2002017 uid159233
Shigella flexneri 2a 301 uid62907
Shigella sonnei 53G uid84383
Shigella sonnei Ss046 uid58217
Shigella boydii CDC 3083 94 uid58415
Shigella boydii Sb227 uid58215
Shigella dysenteriae Sd197 uid58213
Escherichia coli O55 H7 CB9615 uid46655
Escherichia coli O55 H7 RM12579 uid162153
Escherichia coli O157 H7 EDL933 uid57831
Escherichia coli O157 H7 Sakai uid57781
Escherichia coli O157 H7 EC4115 uid59091
Escherichia coli O157 H7 TW14359 uid59235
Escherichia coli Xuzhou21 uid163995
Escherichia coli ETEC H10407 uid161993
Escherichia coli HS uid58393
Escherichia coli ATCC 8739 uid58783
Escherichia coli UMNK88 uid161991
Escherichia coli DH1 uid161951
Escherichia coli DH1 uid162051
Escherichia coli K 12 substr DH10B uid58979
Escherichia coli K 12 substr W3110 uid161931
Escherichia coli BL21 DE3 uid161949
Escherichia coli B REL606 uid58803
Escherichia coli BL21 DE3 uid161947
Escherichia coli BL21 Gold DE3 pLysS AG uid59245
pheT

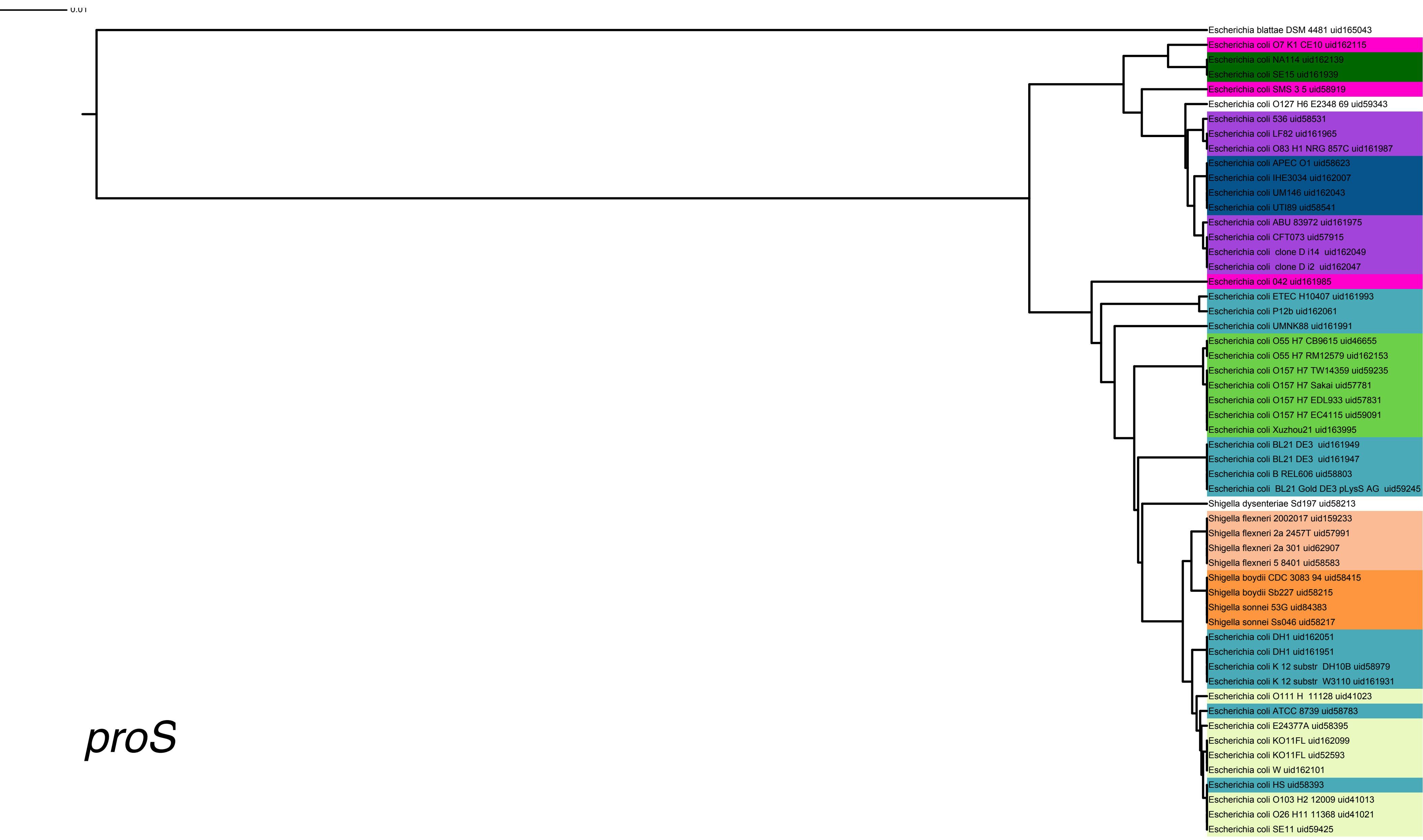
0.01
Escherichia blattae DSM 4481 uid165043
Escherichia coli O7 K1 CE10 uid162115
Escherichia coli NA114 uid162139
Escherichia coli SE15 uid161939
Escherichia coli SMS 3 5 uid58919
Escherichia coli O127 H6 E2348 69 uid59343
Escherichia coli 536 uid58531
Escherichia coli LF82 uid161965
Escherichia coli O83 H1 NRG 857C uid161987
Escherichia coli APEC O1 uid58623
Escherichia coli IHE3034 uid162007
Escherichia coli UM146 uid162043
Escherichia coli UTI89 uid58541
Escherichia coli ABU 83972 uid161975
Escherichia coli CFT073 uid57915
Escherichia coli clone D i14 uid162049
Escherichia coli clone D i2 uid162047
Escherichia coli 042 uid161985
Escherichia coli ETEC H10407 uid161993
Escherichia coli P12b uid162061
Escherichia coli UMNK88 uid161991
Escherichia coli O55 H7 CB9615 uid46655
Escherichia coli O55 H7 RM12579 uid162153
Escherichia coli O157 H7 TW14359 uid59235
Escherichia coli O157 H7 Sakai uid57781
Escherichia coli O157 H7 EDL933 uid57831
Escherichia coli O157 H7 EC4115 uid59091
Escherichia coli Xuzhou21 uid163995
Escherichia coli BL21 DE3 uid161949
Escherichia coli BL21 DE3 uid161947
Escherichia coli B REL606 uid58803
Escherichia coli BL21 Gold DE3 pLysS AG uid59245
Shigella dysenteriae Sd197 uid58213
Shigella flexneri 2002017 uid159233
Shigella flexneri 2a 2457T uid57991
Shigella flexneri 2a 301 uid62907
Shigella flexneri 5 8401 uid58583
Shigella boydii CDC 3083 94 uid58415
Shigella boydii Sb227 uid58215
Shigella sonnei 53G uid84383
Shigella sonnei Ss046 uid58217
Escherichia coli DH1 uid162051
Escherichia coli DH1 uid161951
Escherichia coli K 12 substr DH10B uid58979
Escherichia coli K 12 substr W3110 uid161931
Escherichia coli O111 H 11128 uid41023
Escherichia coli ATCC 8739 uid58783
Escherichia coli E24377A uid58395
Escherichia coli KO11FL uid162099
Escherichia coli KO11FL uid52593
Escherichia coli W uid162101
Escherichia coli HS uid58393
Escherichia coli O103 H2 12009 uid41013
Escherichia coli O26 H11 11368 uid41021
Escherichia coli SE11 uid59425

proS

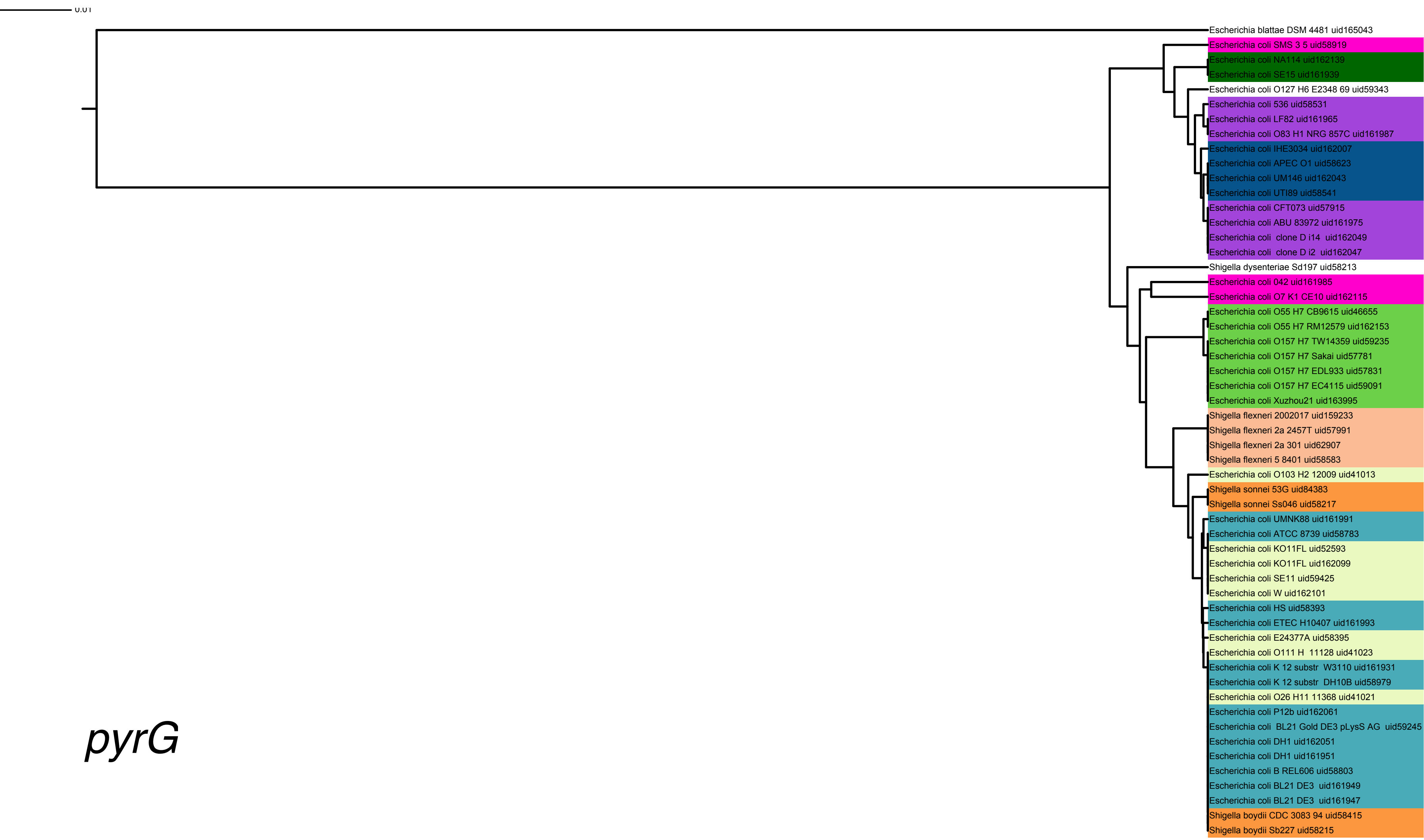

0.01
Escherichia blattae DSM 4481 uid165043
Escherichia coli SMS 3 5 uid58919
Escherichia coli NA114 uid162139
Escherichia coli SE15 uid161939
Escherichia coli O127 H6 E2348 69 uid59343
Escherichia coli 536 uid58531
Escherichia coli LF82 uid161965
Escherichia coli O83 H1 NRG 857C uid161987
Escherichia coli IHE3034 uid162007
Escherichia coli APEC O1 uid58623
Escherichia coli UM146 uid162043
Escherichia coli UTI89 uid58541
Escherichia coli CFT073 uid57915
Escherichia coli ABU 83972 uid161975
Escherichia coli clone D i14 uid162049
Escherichia coli clone D i2 uid162047
Shigella dysenteriae Sd197 uid58213
Escherichia coli 042 uid161985
Escherichia coli O7 K1 CE10 uid162115
Escherichia coli O55 H7 CB9615 uid46655
Escherichia coli O55 H7 RM12579 uid162153
Escherichia coli O157 H7 TW14359 uid59235
Escherichia coli O157 H7 Sakai uid57781
Escherichia coli O157 H7 EDL933 uid57831
Escherichia coli O157 H7 EC4115 uid59091
Escherichia coli Xuzhou21 uid163995
Shigella flexneri 2002017 uid159233
Shigella flexneri 2a 2457T uid57991
Shigella flexneri 2a 301 uid62907
Shigella flexneri 5 8401 uid58583
Escherichia coli O103 H2 12009 uid41013
Shigella sonnei 53G uid84383
Shigella sonnei Ss046 uid58217
Escherichia coli UMNK88 uid161991
Escherichia coli ATCC 8739 uid58783
Escherichia coli KO11FL uid52593
Escherichia coli KO11FL uid162099
Escherichia coli SE11 uid59425
Escherichia coli W uid162101
Escherichia coli HS uid58393
Escherichia coli ETEC H10407 uid161993
Escherichia coli E24377A uid58395
Escherichia coli O111 H 11128 uid41023
Escherichia coli K 12 substr W3110 uid161931
Escherichia coli K 12 substr DH10B uid58979
Escherichia coli O26 H11 11368 uid41021
Escherichia coli P12b uid162061
Escherichia coli BL21 Gold DE3 pLysS AG uid59245
Escherichia coli DH1 uid162051
Escherichia coli DH1 uid161951
Escherichia coli B REL606 uid58803
Escherichia coli BL21 DE3 uid161949
Escherichia coli BL21 DE3 uid161947
Shigella boydii CDC 3083 94 uid58415
Shigella boydii Sb227 uid58215
pyrG

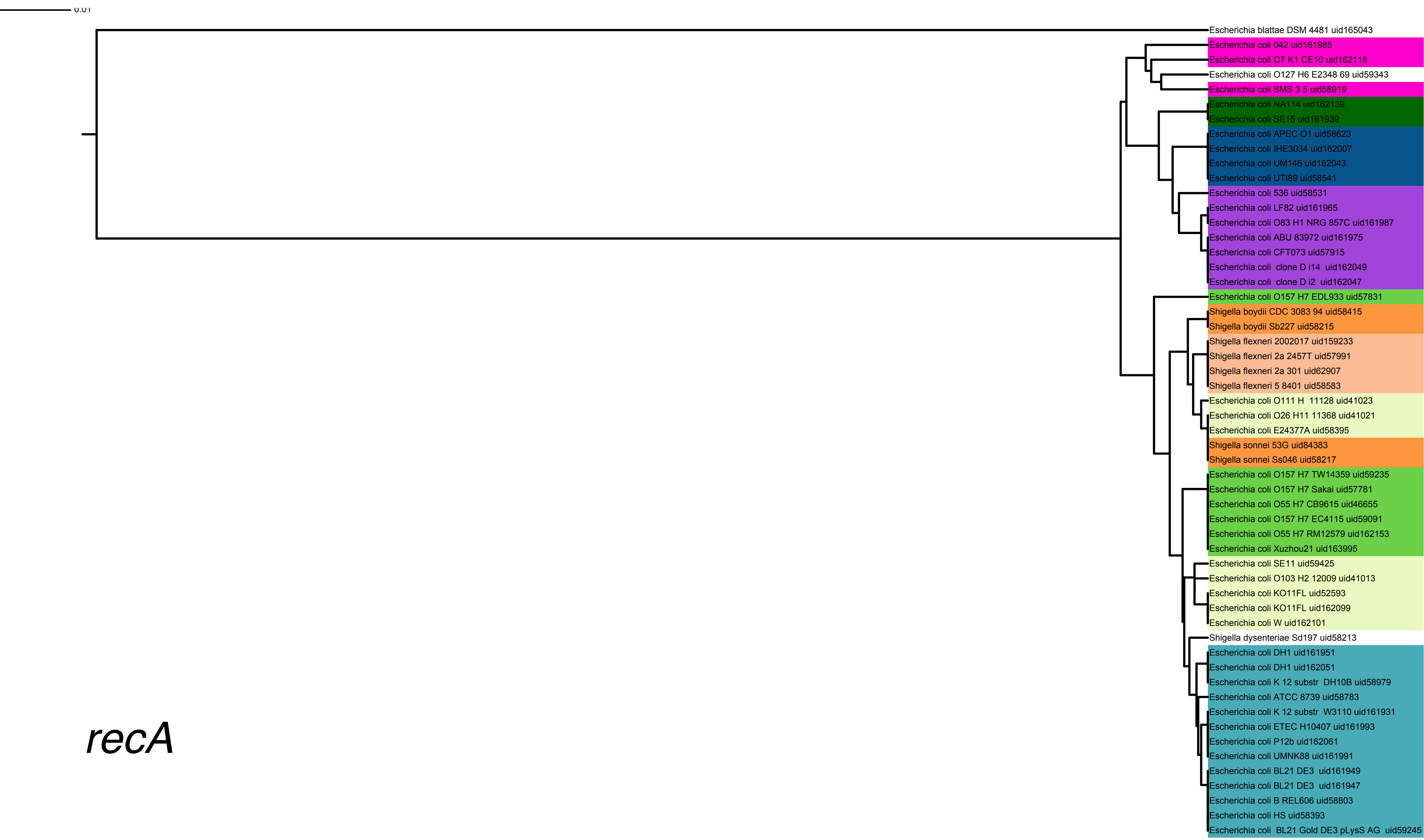
0.01
Escherichia blattae DSM 4481 uid165043
Escherichia coli 042 uid161985
Escherichia coli O7 K1 CE10 uid162115
Escherichia coli O127 H6 E2348 69 uid59343
Escherichia coli SMS 3 5 uid58919
Escherichia coli NA114 uid162139
Escherichia coli SE15 uid161939
Escherichia coli APEC O1 uid58623
Escherichia coli IHE3034 uid162007
Escherichia coli UM146 uid162043
Escherichia coli UTI89 uid58541
Escherichia coli 536 uid58531
Escherichia coli LF82 uid161965
Escherichia coli O83 H1 NRG 857C uid161987
Escherichia coli ABU 83972 uid161975
Escherichia coli CFT073 uid57915
Escherichia coli clone D i14 uid162049
Escherichia coli clone D i2 uid162047
Escherichia coli O157 H7 EDL933 uid57831
Shigella boydii CDC 3083 94 uid58415
Shigella boydii Sb227 uid58215
Shigella flexneri 2002017 uid159233
Shigella flexneri 2a 2457T uid57991
Shigella flexneri 2a 301 uid62907
Shigella flexneri 5 8401 uid58583
Escherichia coli O111 H 11128 uid41023
Escherichia coli O26 H11 11368 uid41021
Escherichia coli E24377A uid58395
Shigella sonnei 53G uid84383
Shigella sonnei Ss046 uid58217
Escherichia coli O157 H7 TW14359 uid59235
Escherichia coli O157 H7 Sakai uid57781
Escherichia coli O55 H7 CB9615 uid46655
Escherichia coli O157 H7 EC4115 uid59091
Escherichia coli O55 H7 RM12579 uid162153
Escherichia coli Xuzhou21 uid163995
Escherichia coli SE11 uid59425
Escherichia coli O103 H2 12009 uid41013
Escherichia coli KO11FL uid52593
Escherichia coli KO11FL uid162099
Escherichia coli W uid162101
Shigella dysenteriae Sd197 uid58213
Escherichia coli DH1 uid161951
Escherichia coli DH1 uid162051
Escherichia coli K 12 substr DH10B uid58979
Escherichia coli ATCC 8739 uid58783
Escherichia coli K 12 substr W3110 uid161931
Escherichia coli ETEC H10407 uid161993
Escherichia coli P12b uid162061
Escherichia coli UMNK88 uid161991
Escherichia coli BL21 DE3 uid161949
Escherichia coli BL21 DE3 uid161947
Escherichia coli B REL606 uid58803
Escherichia coli HS uid58393
Escherichia coli BL21 Gold DE3 pLysS AG uid59245
recA

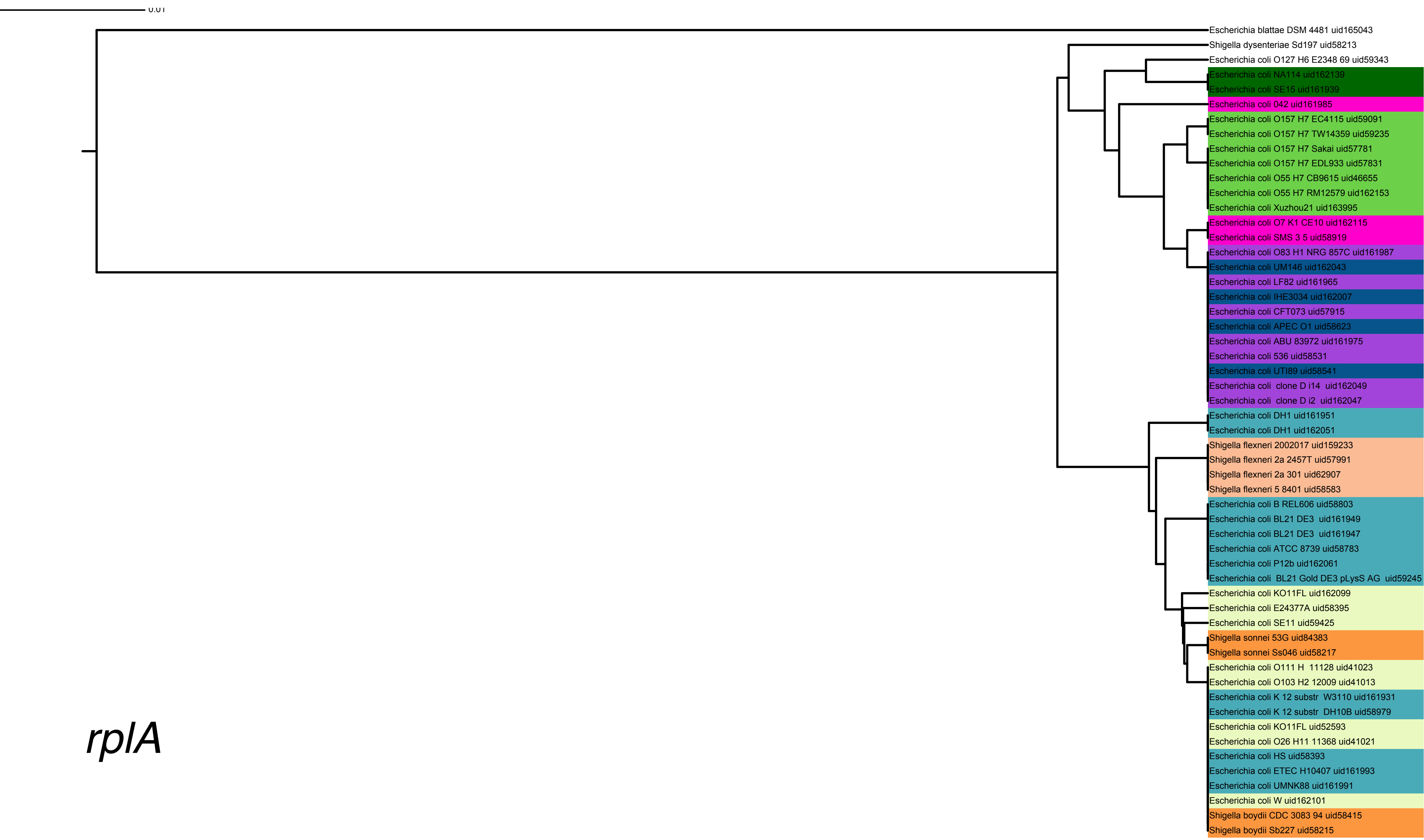

0.01
Escherichia blattae DSM 4481 uid165043
Shigella dysenteriae Sd197 uid58213
Escherichia coli O127 H6 E2348 69 uid59343
Escherichia coli NA114 uid162139
Escherichia coli SE15 uid161939
Escherichia coli 042 uid161985
Escherichia coli O157 H7 EC4115 uid59091
Escherichia coli O157 H7 TW14359 uid59235
Escherichia coli O157 H7 Sakai uid57781
Escherichia coli O157 H7 EDL933 uid57831
Escherichia coli O55 H7 CB9615 uid46655
Escherichia coli O55 H7 RM12579 uid162153
Escherichia coli Xuzhou21 uid163995
Escherichia coli O7 K1 CE10 uid162115
Escherichia coli SMS 3 5 uid58919
Escherichia coli O83 H1 NRG 857C uid161987
Escherichia coli UM146 uid162043
Escherichia coli LF82 uid161965
Escherichia coli IHE3034 uid162007
Escherichia coli CFT073 uid57915
Escherichia coli APEC O1 uid58623
Escherichia coli ABU 83972 uid161975
Escherichia coli 536 uid58531
Escherichia coli UTI89 uid58541
Escherichia coli clone D i14 uid162049
Escherichia coli clone D i2 uid162047
Escherichia coli DH1 uid161951
Escherichia coli DH1 uid162051
Shigella flexneri 2002017 uid159233
Shigella flexneri 2a 2457T uid57991
Shigella flexneri 2a 301 uid62907
Shigella flexneri 5 8401 uid58583
Escherichia coli B REL606 uid58803
Escherichia coli BL21 DE3 uid161949
Escherichia coli BL21 DE3 uid161947
Escherichia coli ATCC 8739 uid58783
Escherichia coli P12b uid162061
Escherichia coli BL21 Gold DE3 pLysS AG uid59245
Escherichia coli KO11FL uid162099
Escherichia coli E24377A uid58395
Escherichia coli SE11 uid59425
Shigella sonnei 53G uid84383
Shigella sonnei Ss046 uid58217
Escherichia coli O111 H 11128 uid41023
Escherichia coli O103 H2 12009 uid41013
Escherichia coli K 12 substr W3110 uid161931
Escherichia coli K 12 substr DH10B uid58979
Escherichia coli KO11FL uid52593
Escherichia coli O26 H11 11368 uid41021
Escherichia coli HS uid58393
Escherichia coli ETEC H10407 uid161993
Escherichia coli UMNK88 uid161991
Escherichia coli W uid162101
Shigella boydii CDC 3083 94 uid58415
Shigella boydii Sb227 uid58215
rplA

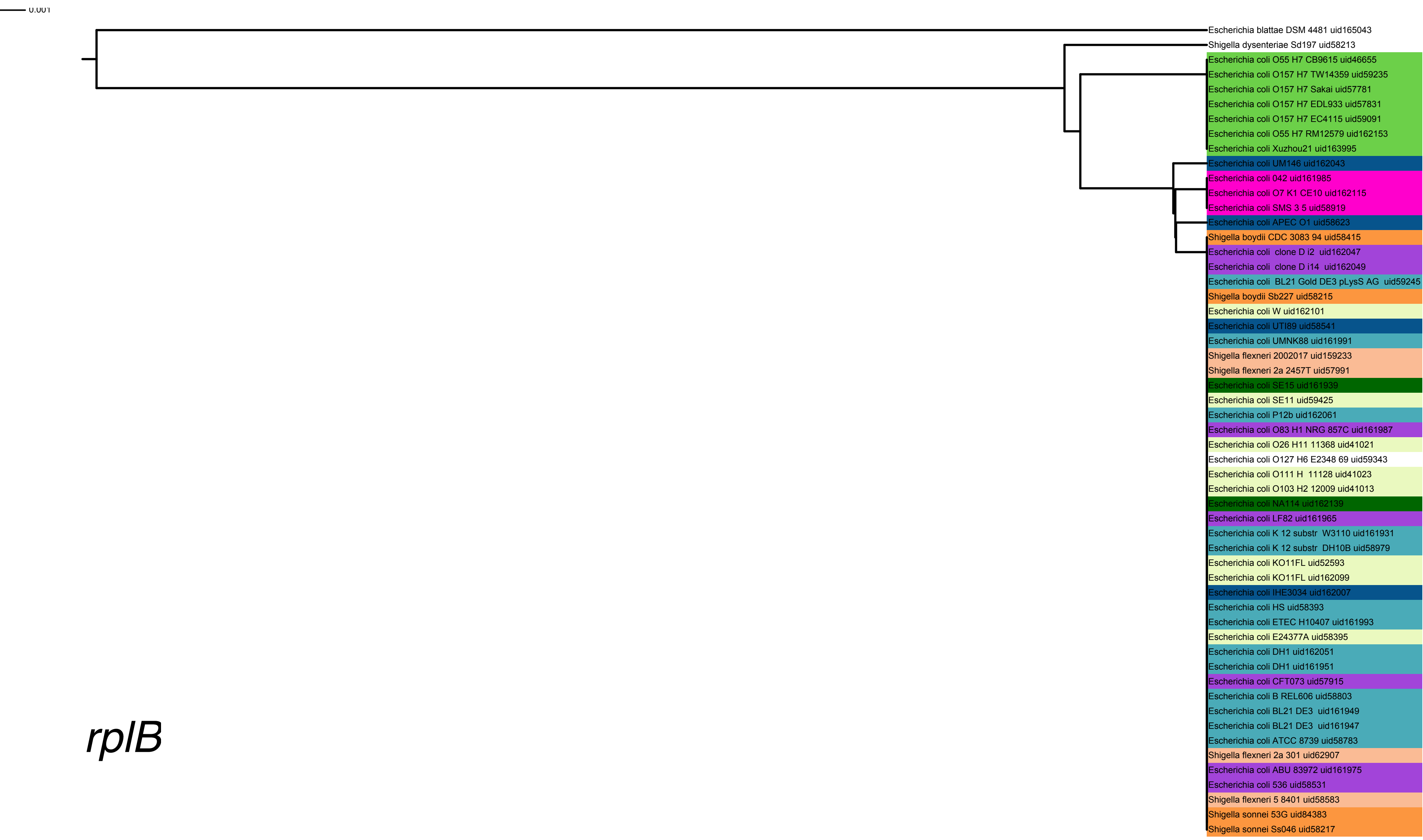
0.001
Escherichia blattae DSM 4481 uid165043
Shigella dysenteriae Sd197 uid58213
Escherichia coli O55 H7 CB9615 uid46655
Escherichia coli O157 H7 TW14359 uid59235
Escherichia coli O157 H7 Sakai uid57781
Escherichia coli O157 H7 EDL933 uid57831
Escherichia coli O157 H7 EC4115 uid59091
Escherichia coli O55 H7 RM12579 uid162153
Escherichia coli Xuzhou21 uid163995
Escherichia coli UM146 uid162043
Escherichia coli 042 uid161985
Escherichia coli O7 K1 CE10 uid162115
Escherichia coli SMS 3 5 uid58919
Escherichia coli APEC O1 uid58623
Shigella boydii CDC 3083 94 uid58415
Escherichia coli clone D i2 uid162047
Escherichia coli clone D i14 uid162049
Escherichia coli BL21 Gold DE3 pLysS AG uid59245
Shigella boydii Sb227 uid58215
Escherichia coli W uid162101
Escherichia coli UTI89 uid58541
Escherichia coli UMNK88 uid161991
Shigella flexneri 2002017 uid159233
Shigella flexneri 2a 2457T uid57991
Escherichia coli SE15 uid161939
Escherichia coli SE11 uid59425
Escherichia coli P12b uid162061
Escherichia coli O83 H1 NRG 857C uid161987
Escherichia coli O26 H11 11368 uid41021
Escherichia coli O127 H6 E2348 69 uid59343
Escherichia coli O111 H 11128 uid41023
Escherichia coli O103 H2 12009 uid41013
Escherichia coli NA114 uid162139
Escherichia coli LF82 uid161965
Escherichia coli K 12 substr W3110 uid161931
Escherichia coli K 12 substr DH10B uid58979
Escherichia coli KO11FL uid52593
Escherichia coli KO11FL uid162099
Escherichia coli IHE3034 uid162007
Escherichia coli HS uid58393
Escherichia coli ETEC H10407 uid161993
Escherichia coli E24377A uid58395
Escherichia coli DH1 uid162051
Escherichia coli DH1 uid161951
Escherichia coli CFT073 uid57915
Escherichia coli B REL606 uid58803
Escherichia coli BL21 DE3 uid161949
Escherichia coli BL21 DE3 uid161947
Escherichia coli ATCC 8739 uid58783
Shigella flexneri 2a 301 uid62907
Escherichia coli ABU 83972 uid161975
Escherichia coli 536 uid58531
Shigella flexneri 5 8401 uid58583
Shigella sonnei 53G uid84383
Shigella sonnei Ss046 uid58217
rplB

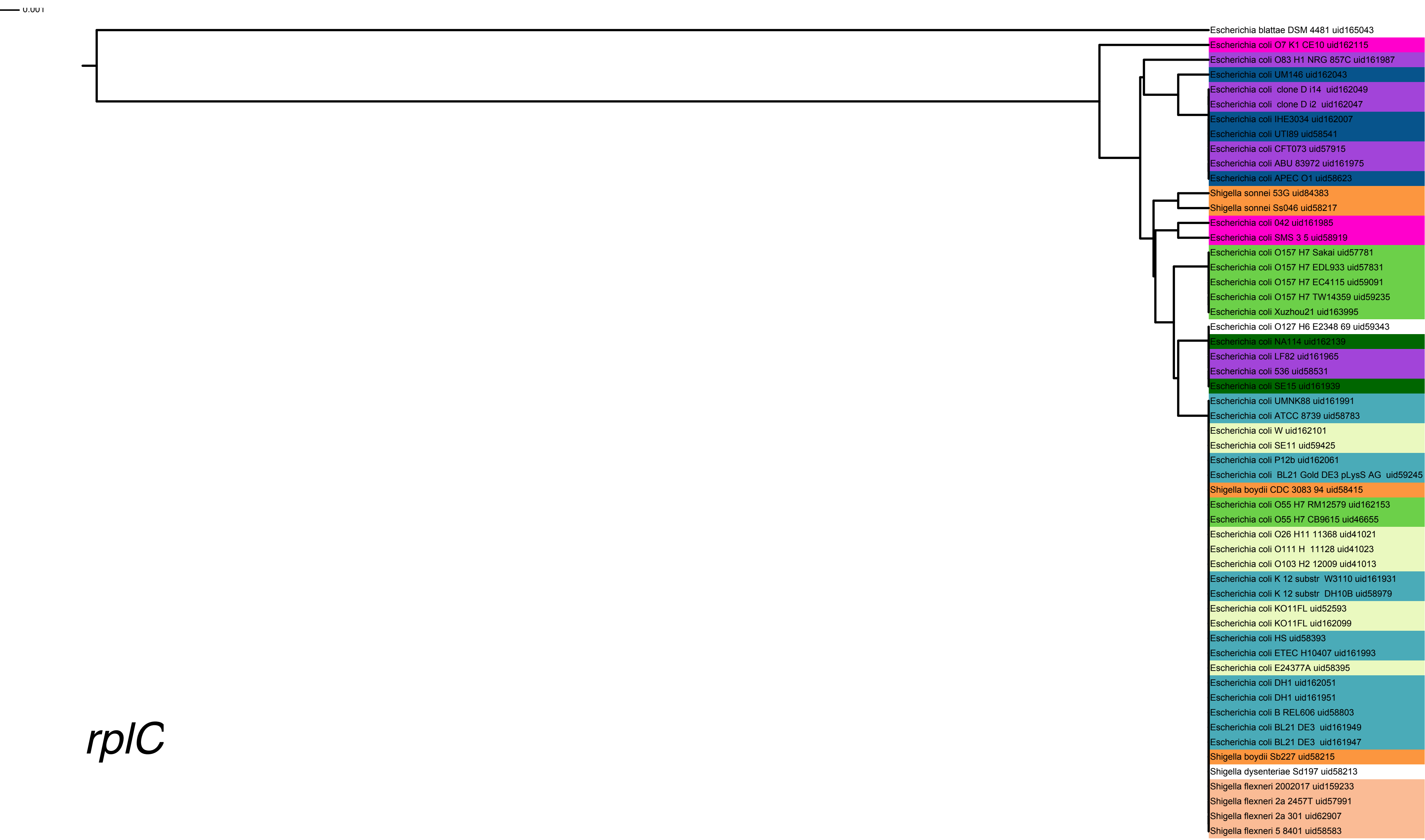
0.001
Escherichia blattae DSM 4481 uid165043
Escherichia coli O7 K1 CE10 uid162115
Escherichia coli O83 H1 NRG 857C uid161987
Escherichia coli UM146 uid162043
Escherichia coli clone D i14 uid162049
Escherichia coli clone D i2 uid162047
Escherichia coli IHE3034 uid162007
Escherichia coli UTI89 uid58541
Escherichia coli CFT073 uid57915
Escherichia coli ABU 83972 uid161975
Escherichia coli APEC O1 uid58623
Shigella sonnei 53G uid84383
Shigella sonnei Ss046 uid58217
Escherichia coli 042 uid161985
Escherichia coli SMS 3 5 uid58919
Escherichia coli O157 H7 Sakai uid57781
Escherichia coli O157 H7 EDL933 uid57831
Escherichia coli O157 H7 EC4115 uid59091
Escherichia coli O157 H7 TW14359 uid59235
Escherichia coli Xuzhou21 uid163995
Escherichia coli O127 H6 E2348 69 uid59343
Escherichia coli NA114 uid162139
Escherichia coli LF82 uid161965
Escherichia coli 536 uid58531
Escherichia coli SE15 uid161939
Escherichia coli UMNK88 uid161991
Escherichia coli ATCC 8739 uid58783
Escherichia coli W uid162101
Escherichia coli SE11 uid59425
Escherichia coli P12b uid162061
Escherichia coli BL21 Gold DE3 pLysS AG uid59245
Shigella boydii CDC 3083 94 uid58415
Escherichia coli O55 H7 RM12579 uid162153
Escherichia coli O55 H7 CB9615 uid46655
Escherichia coli O26 H11 11368 uid41021
Escherichia coli O111 H 11128 uid41023
Escherichia coli O103 H2 12009 uid41013
Escherichia coli K 12 substr W3110 uid161931
Escherichia coli K 12 substr DH10B uid58979
Escherichia coli KO11FL uid52593
Escherichia coli KO11FL uid162099
Escherichia coli HS uid58393
Escherichia coli ETEC H10407 uid161993
Escherichia coli E24377A uid58395
Escherichia coli DH1 uid162051
Escherichia coli DH1 uid161951
Escherichia coli B REL606 uid58803
Escherichia coli BL21 DE3 uid161949
Escherichia coli BL21 DE3 uid161947
Shigella boydii Sb227 uid58215
Shigella dysenteriae Sd197 uid58213
Shigella flexneri 2002017 uid159233
Shigella flexneri 2a 2457T uid57991
Shigella flexneri 2a 301 uid62907
Shigella flexneri 5 8401 uid58583
rplC

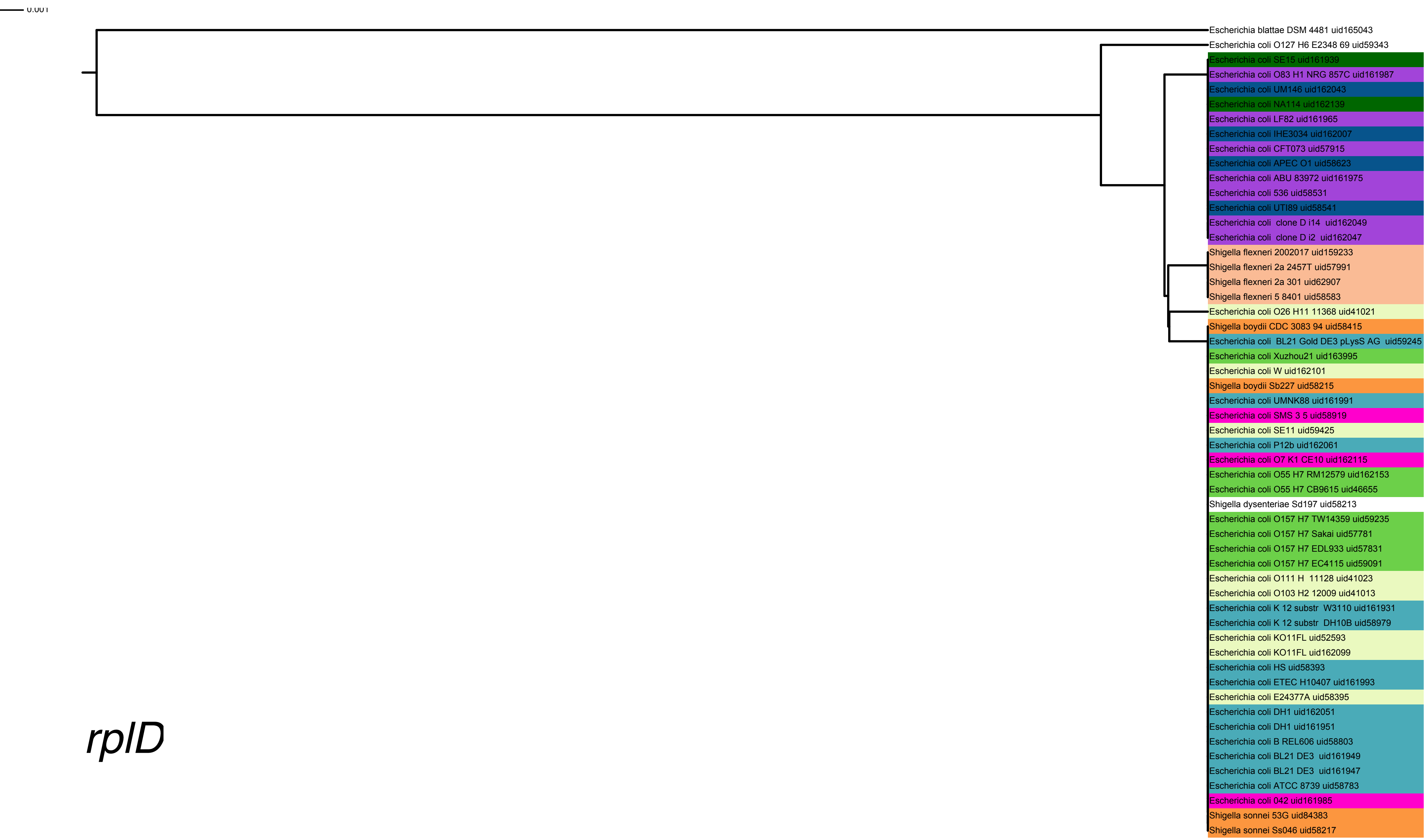
0.001
Escherichia blattae DSM 4481 uid165043
Escherichia coli O127 H6 E2348 69 uid59343
Escherichia coli SE15 uid161939
Escherichia coli O83 H1 NRG 857C uid161987
Escherichia coli UM146 uid162043
Escherichia coli NA114 uid162139
Escherichia coli LF82 uid161965
Escherichia coli IHE3034 uid162007
Escherichia coli CFT073 uid57915
Escherichia coli APEC O1 uid58623
Escherichia coli ABU 83972 uid161975
Escherichia coli 536 uid58531
Escherichia coli UTI89 uid58541
Escherichia coli clone D i14 uid162049
Escherichia coli clone D i2 uid162047
Shigella flexneri 2002017 uid159233
Shigella flexneri 2a 2457T uid57991
Shigella flexneri 2a 301 uid62907
Shigella flexneri 5 8401 uid58583
Escherichia coli O26 H11 11368 uid41021
Shigella boydii CDC 3083 94 uid58415
Escherichia coli BL21 Gold DE3 pLysS AG uid59245
Escherichia coli Xuzhou21 uid163995
Escherichia coli W uid162101
Shigella boydii Sb227 uid58215
Escherichia coli UMNK88 uid161991
Escherichia coli SMS 3 5 uid58919
Escherichia coli SE11 uid59425
Escherichia coli P12b uid162061
Escherichia coli O7 K1 CE10 uid162115
Escherichia coli O55 H7 RM12579 uid162153
Escherichia coli O55 H7 CB9615 uid46655
Shigella dysenteriae Sd197 uid58213
Escherichia coli O157 H7 TW14359 uid59235
Escherichia coli O157 H7 Sakai uid57781
Escherichia coli O157 H7 EDL933 uid57831
Escherichia coli O157 H7 EC4115 uid59091
Escherichia coli O111 H 11128 uid41023
Escherichia coli O103 H2 12009 uid41013
Escherichia coli K 12 substr W3110 uid161931
Escherichia coli K 12 substr DH10B uid58979
Escherichia coli KO11FL uid52593
Escherichia coli KO11FL uid162099
Escherichia coli HS uid58393
Escherichia coli ETEC H10407 uid161993
Escherichia coli E24377A uid58395
Escherichia coli DH1 uid162051
Escherichia coli DH1 uid161951
Escherichia coli B REL606 uid58803
Escherichia coli BL21 DE3 uid161949
Escherichia coli BL21 DE3 uid161947
Escherichia coli ATCC 8739 uid58783
Escherichia coli 042 uid161985
Shigella sonnei 53G uid84383
Shigella sonnei Ss046 uid58217
rplD

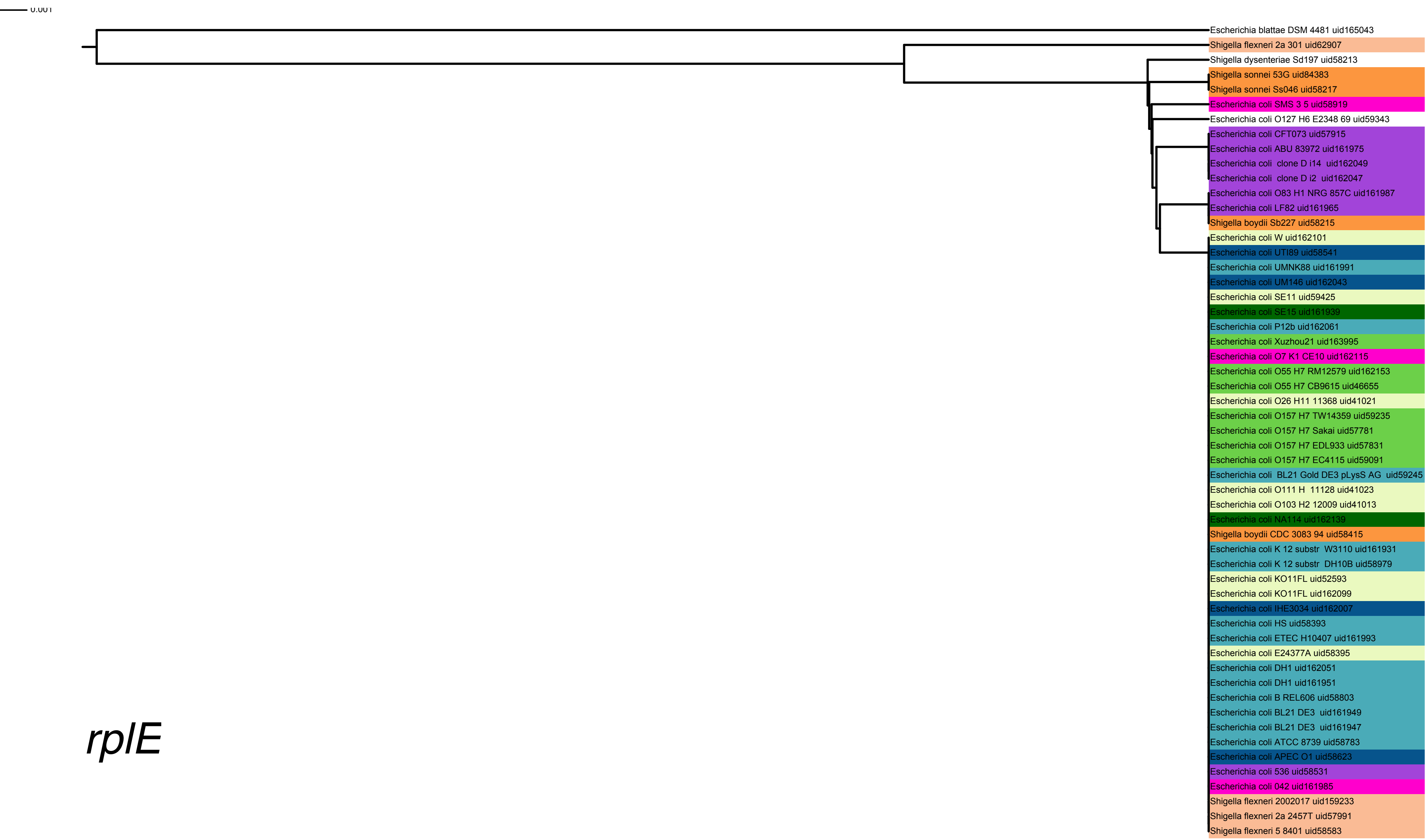
0.001
Escherichia blattae DSM 4481 uid165043
Shigella flexneri 2a 301 uid62907
Shigella dysenteriae Sd197 uid58213
Shigella sonnei 53G uid84383
Shigella sonnei Ss046 uid58217
Escherichia coli SMS 3 5 uid58919
Escherichia coli O127 H6 E2348 69 uid59343
Escherichia coli CFT073 uid57915
Escherichia coli ABU 83972 uid161975
Escherichia coli clone D i14 uid162049
Escherichia coli clone D i2 uid162047
Escherichia coli O83 H1 NRG 857C uid161987
Escherichia coli LF82 uid161965
Shigella boydii Sb227 uid58215
Escherichia coli W uid162101
Escherichia coli UTI89 uid58541
Escherichia coli UMNK88 uid161991
Escherichia coli UM146 uid162043
Escherichia coli SE11 uid59425
Escherichia coli SE15 uid161939
Escherichia coli P12b uid162061
Escherichia coli Xuzhou21 uid163995
Escherichia coli O7 K1 CE10 uid162115
Escherichia coli O55 H7 RM12579 uid162153
Escherichia coli O55 H7 CB9615 uid46655
Escherichia coli O26 H11 11368 uid41021
Escherichia coli O157 H7 TW14359 uid59235
Escherichia coli O157 H7 Sakai uid57781
Escherichia coli O157 H7 EDL933 uid57831
Escherichia coli O157 H7 EC4115 uid59091
Escherichia coli BL21 Gold DE3 pLysS AG uid59245
Escherichia coli O111 H 11128 uid41023
Escherichia coli O103 H2 12009 uid41013
Escherichia coli NA114 uid162139
Shigella boydii CDC 3083 94 uid58415
Escherichia coli K 12 substr W3110 uid161931
Escherichia coli K 12 substr DH10B uid58979
Escherichia coli KO11FL uid52593
Escherichia coli KO11FL uid162099
Escherichia coli IHE3034 uid162007
Escherichia coli HS uid58393
Escherichia coli ETEC H10407 uid161993
Escherichia coli E24377A uid58395
Escherichia coli DH1 uid162051
Escherichia coli DH1 uid161951
Escherichia coli B REL606 uid58803
Escherichia coli BL21 DE3 uid161949
Escherichia coli BL21 DE3 uid161947
Escherichia coli ATCC 8739 uid58783
Escherichia coli APEC O1 uid58623
Escherichia coli 536 uid58531
Escherichia coli 042 uid161985
Shigella flexneri 2002017 uid159233
Shigella flexneri 2a 2457T uid57991
Shigella flexneri 5 8401 uid58583
rplE

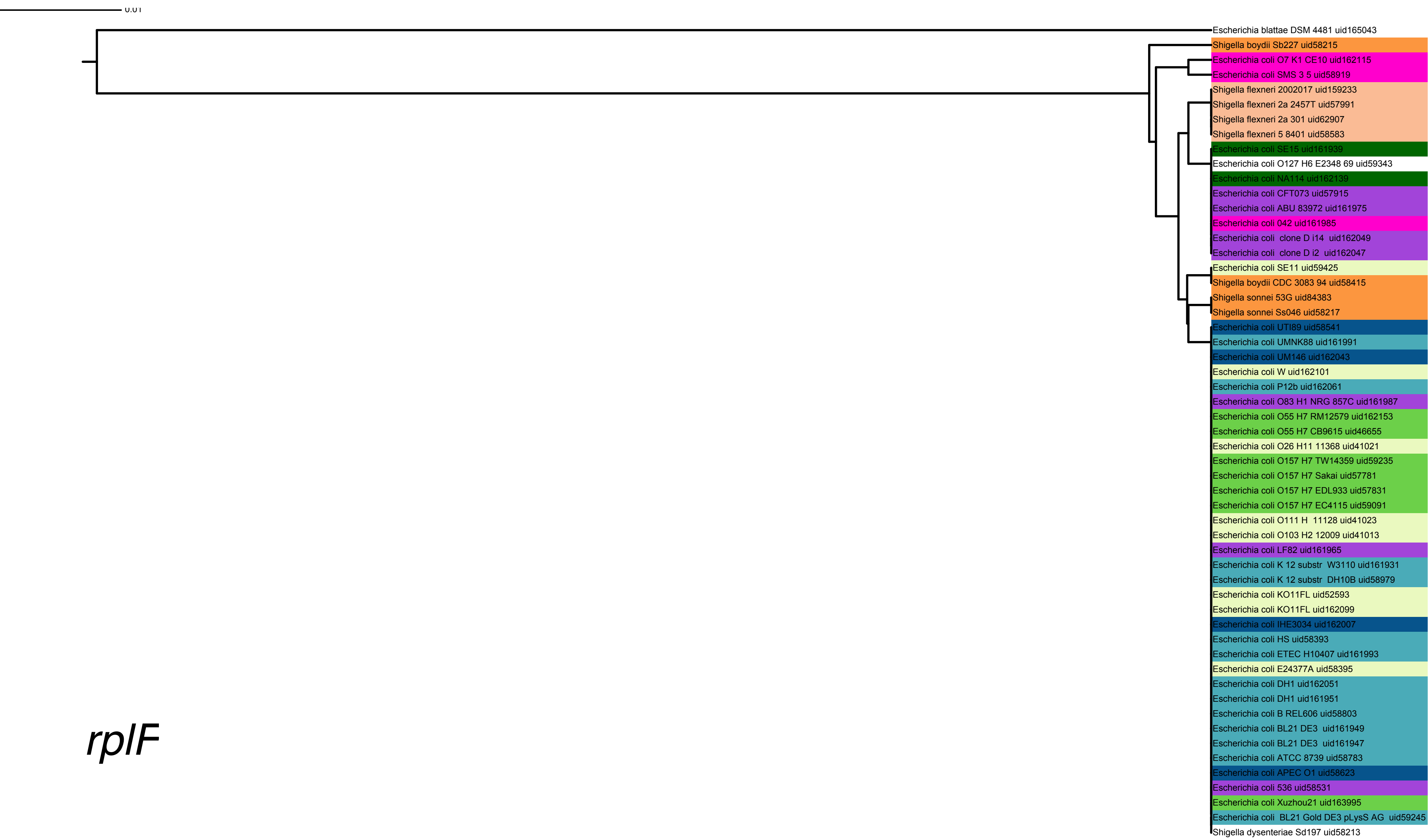

0.01
Escherichia blattae DSM 4481 uid165043
Shigella boydii Sb227 uid58215
Escherichia coli O7 K1 CE10 uid162115
Escherichia coli SMS 3 5 uid58919
Shigella flexneri 2002017 uid159233
Shigella flexneri 2a 2457T uid57991
Shigella flexneri 2a 301 uid62907
Shigella flexneri 5 8401 uid58583
Escherichia coli SE15 uid161939
Escherichia coli O127 H6 E2348 69 uid59343
Escherichia coli NA114 uid162139
Escherichia coli CFT073 uid57915
Escherichia coli ABU 83972 uid161975
Escherichia coli 042 uid161985
Escherichia coli clone D i14 uid162049
Escherichia coli clone D i2 uid162047
Escherichia coli SE11 uid59425
Shigella boydii CDC 3083 94 uid58415
Shigella sonnei 53G uid84383
Shigella sonnei Ss046 uid58217
Escherichia coli UTI89 uid58541
Escherichia coli UMNK88 uid161991
Escherichia coli UM146 uid162043
Escherichia coli W uid162101
Escherichia coli P12b uid162061
Escherichia coli O83 H1 NRG 857C uid161987
Escherichia coli O55 H7 RM12579 uid162153
Escherichia coli O55 H7 CB9615 uid46655
Escherichia coli O26 H11 11368 uid41021
Escherichia coli O157 H7 TW14359 uid59235
Escherichia coli O157 H7 Sakai uid57781
Escherichia coli O157 H7 EDL933 uid57831
Escherichia coli O157 H7 EC4115 uid59091
Escherichia coli O111 H 11128 uid41023
Escherichia coli O103 H2 12009 uid41013
Escherichia coli LF82 uid161965
Escherichia coli K 12 substr W3110 uid161931
Escherichia coli K 12 substr DH10B uid58979
Escherichia coli KO11FL uid52593
Escherichia coli KO11FL uid162099
Escherichia coli IHE3034 uid162007
Escherichia coli HS uid58393
Escherichia coli ETEC H10407 uid161993
Escherichia coli E24377A uid58395
Escherichia coli DH1 uid162051
Escherichia coli DH1 uid161951
Escherichia coli B REL606 uid58803
Escherichia coli BL21 DE3 uid161949
Escherichia coli BL21 DE3 uid161947
Escherichia coli ATCC 8739 uid58783
Escherichia coli APEC O1 uid58623
Escherichia coli 536 uid58531
Escherichia coli Xuzhou21 uid163995
Escherichia coli BL21 Gold DE3 pLysS AG uid59245
Shigella dysenteriae Sd197 uid58213
rplF

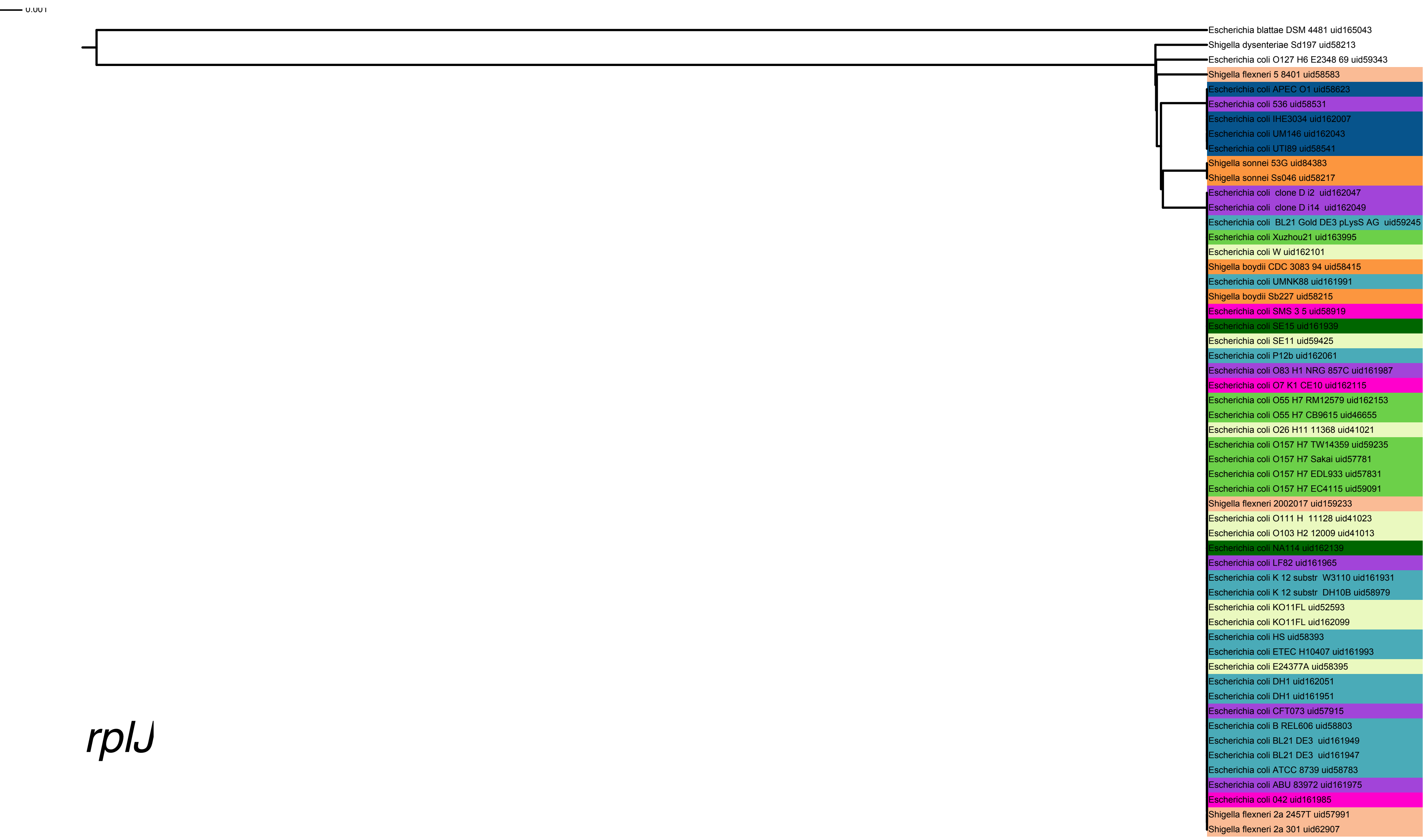
0.001
Escherichia blattae DSM 4481 uid165043
Shigella dysenteriae Sd197 uid58213
Escherichia coli O127 H6 E2348 69 uid59343
Shigella flexneri 5 8401 uid58583
Escherichia coli APEC O1 uid58623
Escherichia coli 536 uid58531
Escherichia coli IHE3034 uid162007
Escherichia coli UM146 uid162043
Escherichia coli UTI89 uid58541
Shigella sonnei 53G uid84383
Shigella sonnei Ss046 uid58217
Escherichia coli clone D i2 uid162047
Escherichia coli clone D i14 uid162049
Escherichia coli BL21 Gold DE3 pLysS AG uid59245
Escherichia coli Xuzhou21 uid163995
Escherichia coli W uid162101
Shigella boydii CDC 3083 94 uid58415
Escherichia coli UMNK88 uid161991
Shigella boydii Sb227 uid58215
Escherichia coli SMS 3 5 uid58919
Escherichia coli SE15 uid161939
Escherichia coli SE11 uid59425
Escherichia coli P12b uid162061
Escherichia coli O83 H1 NRG 857C uid161987
Escherichia coli O7 K1 CE10 uid162115
Escherichia coli O55 H7 RM12579 uid162153
Escherichia coli O55 H7 CB9615 uid46655
Escherichia coli O26 H11 11368 uid41021
Escherichia coli O157 H7 TW14359 uid59235
Escherichia coli O157 H7 Sakai uid57781
Escherichia coli O157 H7 EDL933 uid57831
Escherichia coli O157 H7 EC4115 uid59091
Shigella flexneri 2002017 uid159233
Escherichia coli O111 H 11128 uid41023
Escherichia coli O103 H2 12009 uid41013
Escherichia coli NA114 uid162139
Escherichia coli LF82 uid161965
Escherichia coli K 12 substr W3110 uid161931
Escherichia coli K 12 substr DH10B uid58979
Escherichia coli KO11FL uid52593
Escherichia coli KO11FL uid162099
Escherichia coli HS uid58393
Escherichia coli ETEC H10407 uid161993
Escherichia coli E24377A uid58395
Escherichia coli DH1 uid162051
Escherichia coli DH1 uid161951
Escherichia coli CFT073 uid57915
Escherichia coli B REL606 uid58803
Escherichia coli BL21 DE3 uid161949
Escherichia coli BL21 DE3 uid161947
Escherichia coli ATCC 8739 uid58783
Escherichia coli ABU 83972 uid161975
Escherichia coli 042 uid161985
Shigella flexneri 2a 2457T uid57991
Shigella flexneri 2a 301 uid62907
rplJ

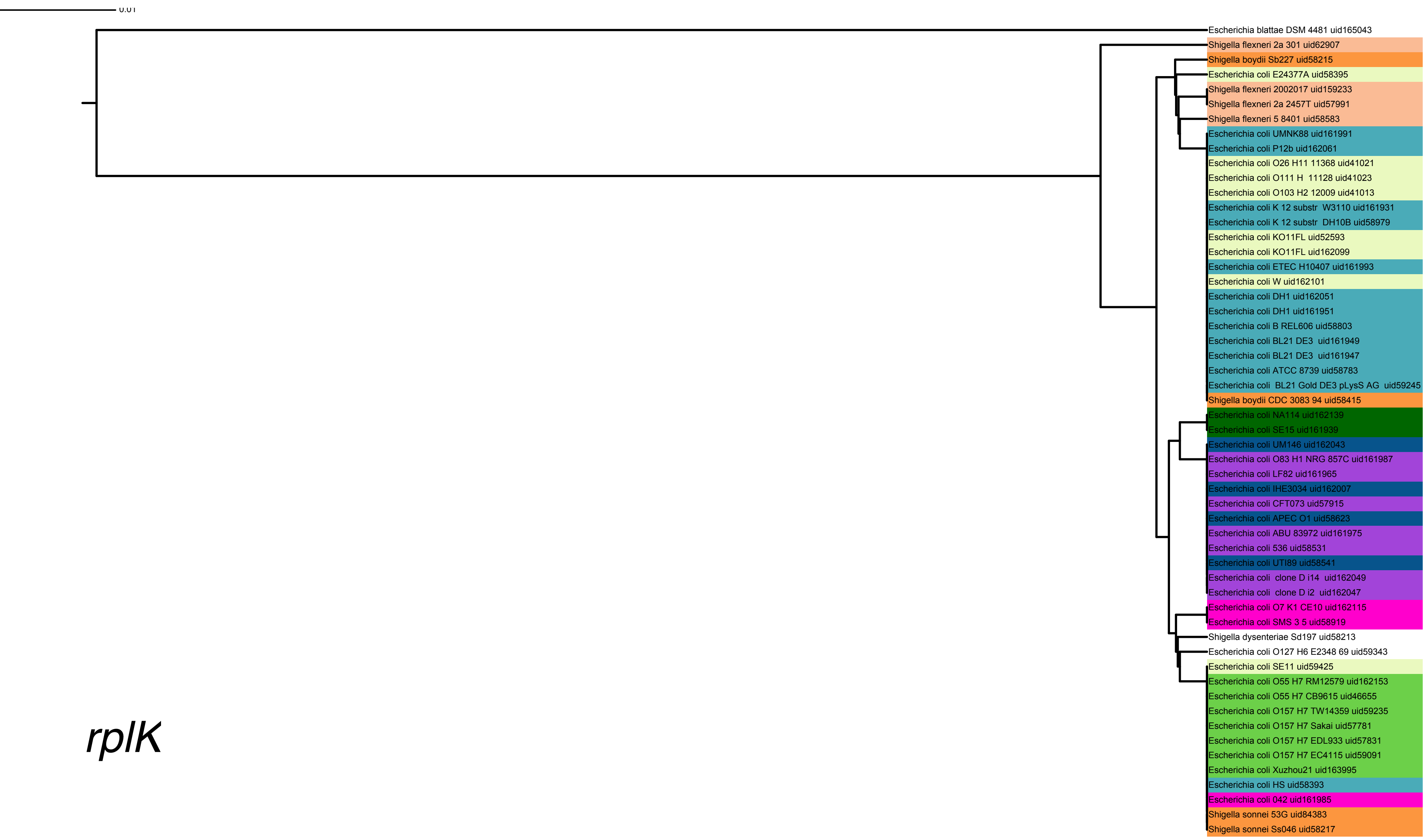

0.01
Escherichia blattae DSM 4481 uid165043
Shigella flexneri 2a 301 uid62907
Shigella boydii Sb227 uid58215
Escherichia coli E24377A uid58395
Shigella flexneri 2002017 uid159233
Shigella flexneri 2a 2457T uid57991
Shigella flexneri 5 8401 uid58583
Escherichia coli UMNK88 uid161991
Escherichia coli P12b uid162061
Escherichia coli O26 H11 11368 uid41021
Escherichia coli O111 H 11128 uid41023
Escherichia coli O103 H2 12009 uid41013
Escherichia coli K 12 substr W3110 uid161931
Escherichia coli K 12 substr DH10B uid58979
Escherichia coli KO11FL uid52593
Escherichia coli KO11FL uid162099
Escherichia coli ETEC H10407 uid161993
Escherichia coli W uid162101
Escherichia coli DH1 uid162051
Escherichia coli DH1 uid161951
Escherichia coli B REL606 uid58803
Escherichia coli BL21 DE3 uid161949
Escherichia coli BL21 DE3 uid161947
Escherichia coli ATCC 8739 uid58783
Escherichia coli BL21 Gold DE3 pLysS AG uid59245
Shigella boydii CDC 3083 94 uid58415
Escherichia coli NA114 uid162139
Escherichia coli SE15 uid161939
Escherichia coli UM146 uid162043
Escherichia coli O83 H1 NRG 857C uid161987
Escherichia coli LF82 uid161965
Escherichia coli IHE3034 uid162007
Escherichia coli CFT073 uid57915
Escherichia coli APEC O1 uid58623
Escherichia coli ABU 83972 uid161975
Escherichia coli 536 uid58531
Escherichia coli UTI89 uid58541
Escherichia coli clone D i14 uid162049
Escherichia coli clone D i2 uid162047
Escherichia coli O7 K1 CE10 uid162115
Escherichia coli SMS 3 5 uid58919
Shigella dysenteriae Sd197 uid58213
Escherichia coli O127 H6 E2348 69 uid59343
Escherichia coli SE11 uid59425
Escherichia coli O55 H7 RM12579 uid162153
Escherichia coli O55 H7 CB9615 uid46655
Escherichia coli O157 H7 TW14359 uid59235
Escherichia coli O157 H7 Sakai uid57781
Escherichia coli O157 H7 EDL933 uid57831
Escherichia coli O157 H7 EC4115 uid59091
Escherichia coli Xuzhou21 uid163995
Escherichia coli HS uid58393
Escherichia coli 042 uid161985
Shigella sonnei 53G uid84383
Shigella sonnei Ss046 uid58217
rplK

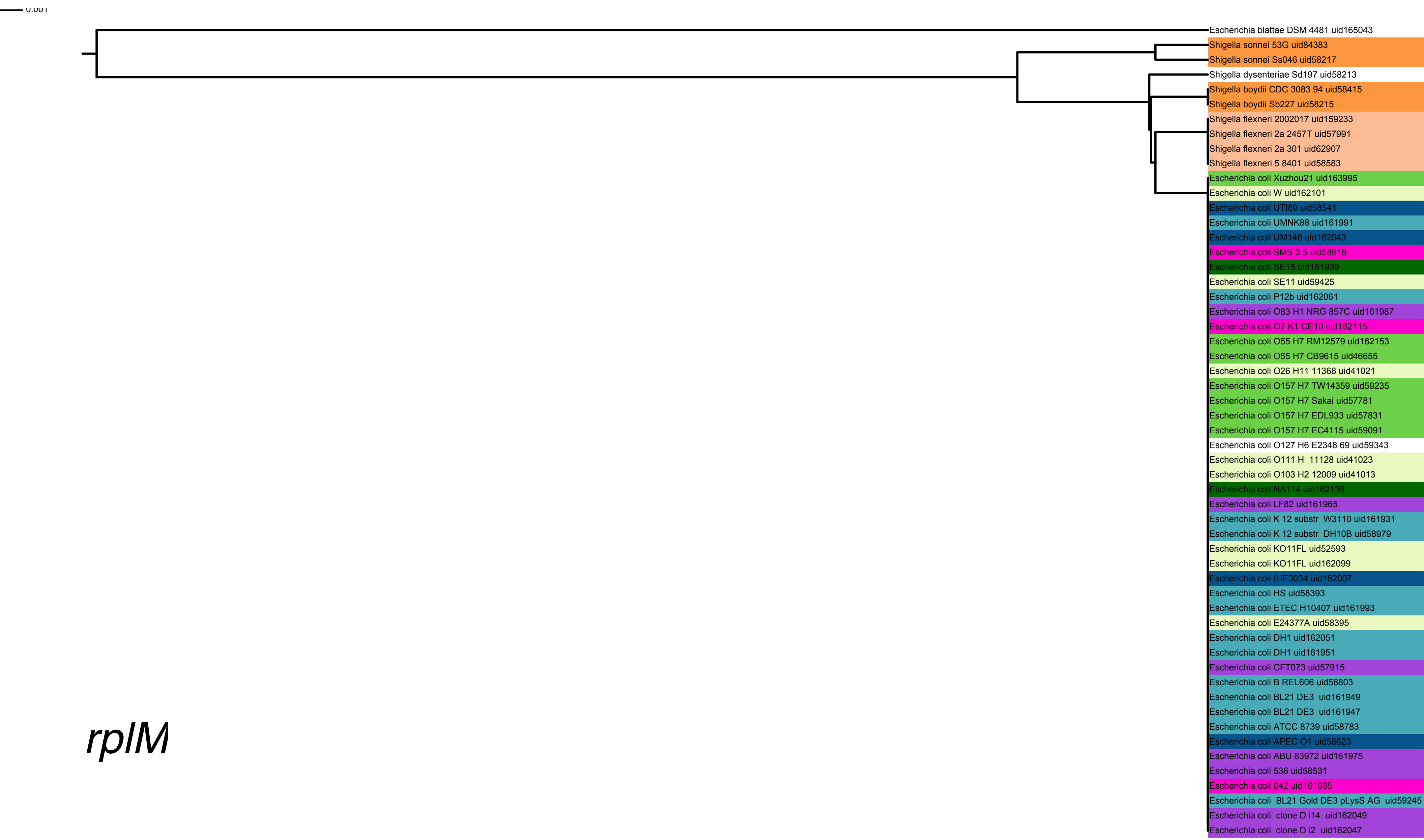
0.001
Escherichia blattae DSM 4481 uid165043
Shigella sonnei 53G uid84383
Shigella sonnei Ss046 uid58217
Shigella dysenteriae Sd197 uid58213
Shigella boydii CDC 3083 94 uid58415
Shigella boydii Sb227 uid58215
Shigella flexneri 2002017 uid159233
Shigella flexneri 2a 2457T uid57991
Shigella flexneri 2a 301 uid62907
Shigella flexneri 5 8401 uid58583
Escherichia coli Xuzhou21 uid163995
Escherichia coli W uid162101
Escherichia coli UTI89 uid58541
Escherichia coli UMNK88 uid161991
Escherichia coli UM146 uid162043
Escherichia coli SMS 3 5 uid58919
Escherichia coli SE15 uid161939
Escherichia coli SE11 uid59425
Escherichia coli P12b uid162061
Escherichia coli O83 H1 NRG 857C uid161987
Escherichia coli O7 K1 CE10 uid162115
Escherichia coli O55 H7 RM12579 uid162153
Escherichia coli O55 H7 CB9615 uid46655
Escherichia coli O26 H11 11368 uid41021
Escherichia coli O157 H7 TW14359 uid59235
Escherichia coli O157 H7 Sakai uid57781
Escherichia coli O157 H7 EDL933 uid57831
Escherichia coli O157 H7 EC4115 uid59091
Escherichia coli O127 H6 E2348 69 uid59343
Escherichia coli O111 H 11128 uid41023
Escherichia coli O103 H2 12009 uid41013
Escherichia coli NA114 uid162139
Escherichia coli LF82 uid161965
Escherichia coli K 12 substr W3110 uid161931
Escherichia coli K 12 substr DH10B uid58979
Escherichia coli KO11FL uid52593
Escherichia coli KO11FL uid162099
Escherichia coli IHE3034 uid162007
Escherichia coli HS uid58393
Escherichia coli ETEC H10407 uid161993
Escherichia coli E24377A uid58395
Escherichia coli DH1 uid162051
Escherichia coli DH1 uid161951
Escherichia coli CFT073 uid57915
Escherichia coli B REL606 uid58803
Escherichia coli BL21 DE3 uid161949
Escherichia coli BL21 DE3 uid161947
Escherichia coli ATCC 8739 uid58783
Escherichia coli APEC O1 uid58623
Escherichia coli ABU 83972 uid161975
Escherichia coli 536 uid58531
Escherichia coli 042 uid161985
Escherichia coli BL21 Gold DE3 pLysS AG uid59245
Escherichia coli clone D i14 uid162049
Escherichia coli clone D i2 uid162047

rplM

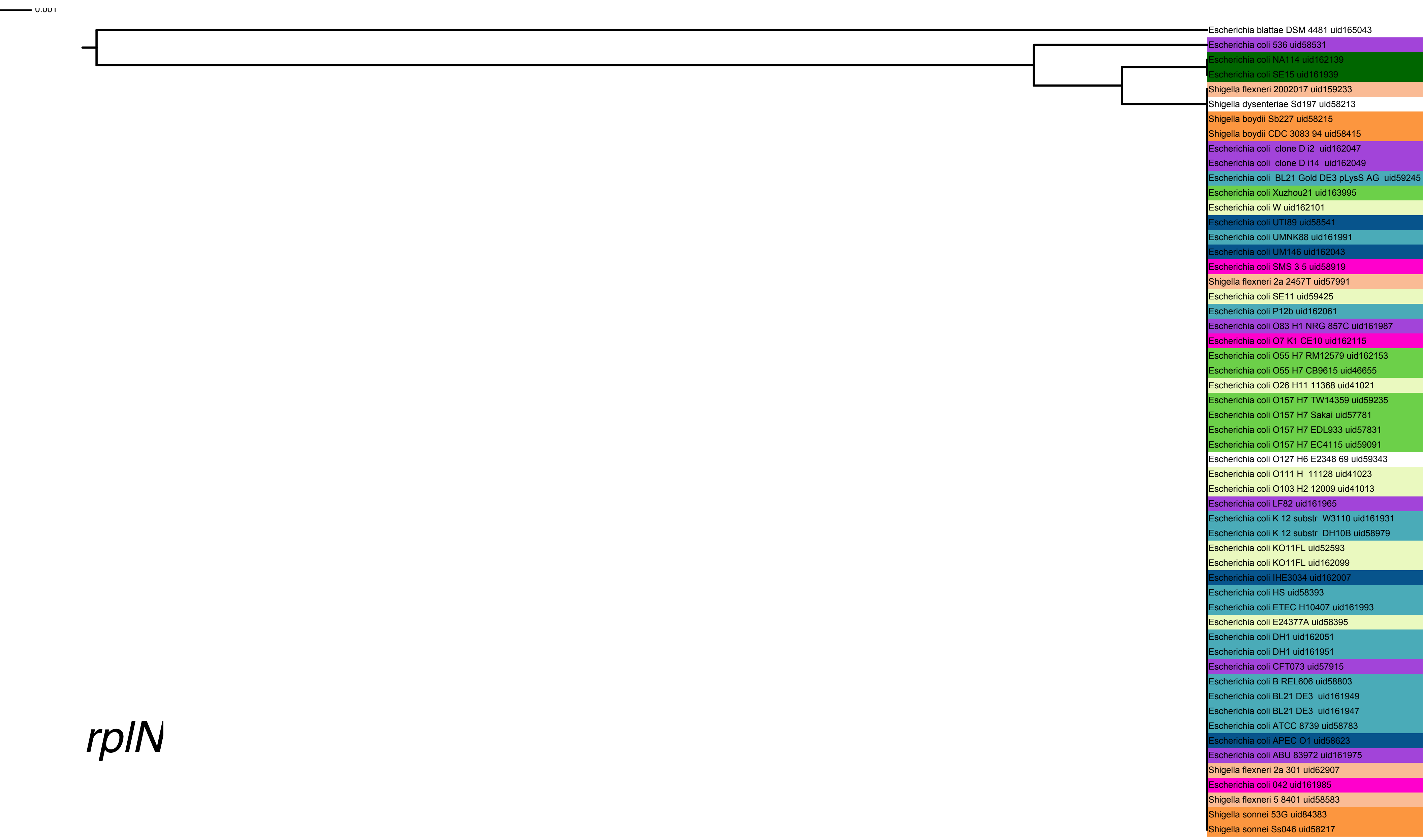
0.001
Escherichia blattae DSM 4481 uid165043
Escherichia coli 536 uid58531
Escherichia coli NA114 uid162139
Escherichia coli SE15 uid161939
Shigella flexneri 2002017 uid159233
Shigella dysenteriae Sd197 uid58213
Shigella boydii Sb227 uid58215
Shigella boydii CDC 3083 94 uid58415
Escherichia coli clone D i2 uid162047
Escherichia coli clone D i14 uid162049
Escherichia coli BL21 Gold DE3 pLysS AG uid59245
Escherichia coli Xuzhou21 uid163995
Escherichia coli W uid162101
Escherichia coli UTI89 uid58541
Escherichia coli UMNK88 uid161991
Escherichia coli UM146 uid162043
Escherichia coli SMS 3 5 uid58919
Shigella flexneri 2a 2457T uid57991
Escherichia coli SE11 uid59425
Escherichia coli P12b uid162061
Escherichia coli O83 H1 NRG 857C uid161987
Escherichia coli O7 K1 CE10 uid162115
Escherichia coli O55 H7 RM12579 uid162153
Escherichia coli O55 H7 CB9615 uid46655
Escherichia coli O26 H11 11368 uid41021
Escherichia coli O157 H7 TW14359 uid59235
Escherichia coli O157 H7 Sakai uid57781
Escherichia coli O157 H7 EDL933 uid57831
Escherichia coli O157 H7 EC4115 uid59091
Escherichia coli O127 H6 E2348 69 uid59343
Escherichia coli O111 H 11128 uid41023
Escherichia coli O103 H2 12009 uid41013
Escherichia coli LF82 uid161965
Escherichia coli K 12 substr W3110 uid161931
Escherichia coli K 12 substr DH10B uid58979
Escherichia coli KO11FL uid52593
Escherichia coli KO11FL uid162099
Escherichia coli IHE3034 uid162007
Escherichia coli HS uid58393
Escherichia coli ETEC H10407 uid161993
Escherichia coli E24377A uid58395
Escherichia coli DH1 uid162051
Escherichia coli DH1 uid161951
Escherichia coli CFT073 uid57915
Escherichia coli B REL606 uid58803
Escherichia coli BL21 DE3 uid161949
Escherichia coli BL21 DE3 uid161947
Escherichia coli ATCC 8739 uid58783
Escherichia coli APEC O1 uid58623
Escherichia coli ABU 83972 uid161975
Shigella flexneri 2a 301 uid62907
Escherichia coli 042 uid161985
Shigella flexneri 5 8401 uid58583
Shigella sonnei 53G uid84383
Shigella sonnei Ss046 uid58217
rplN

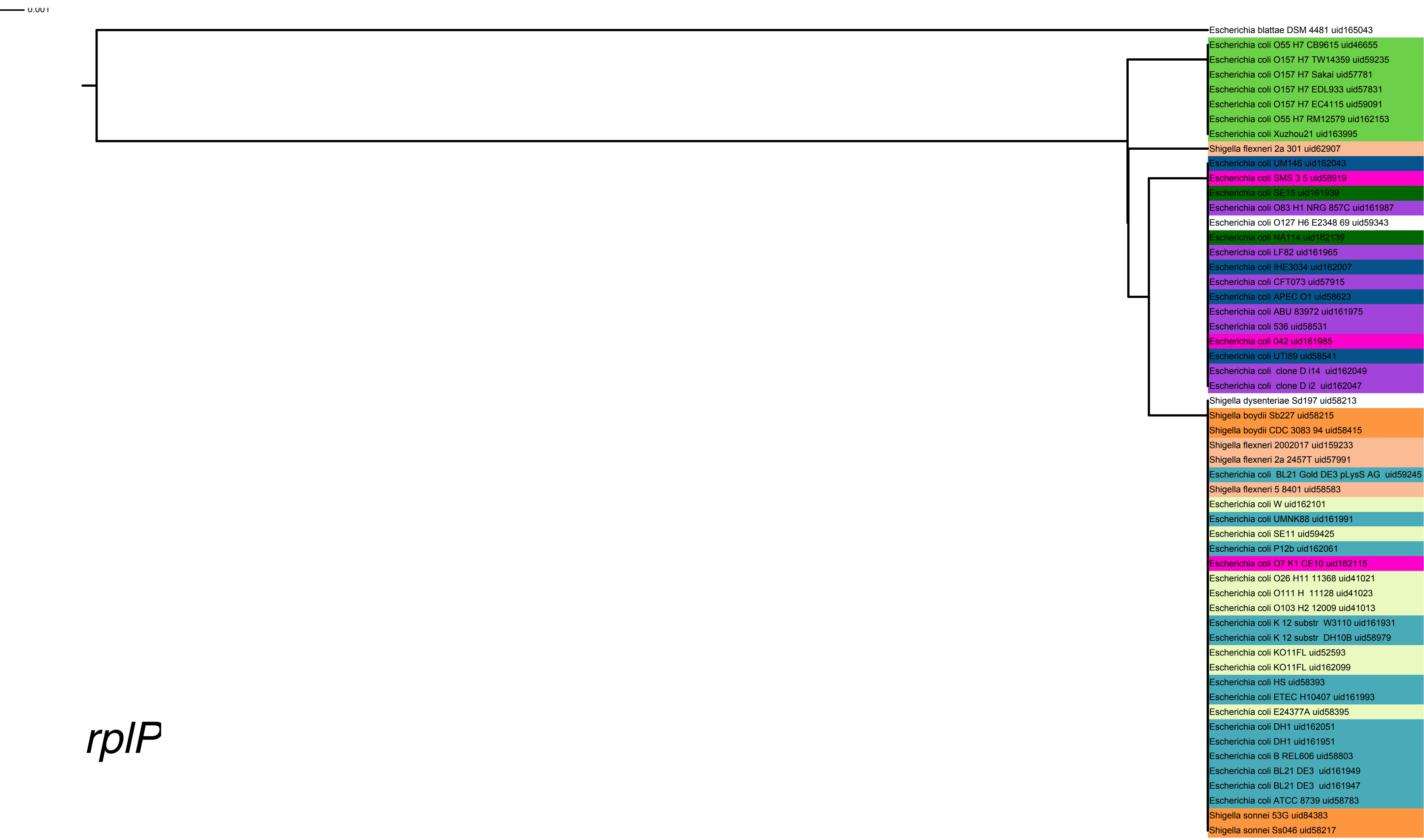
0.001
Escherichia blattae DSM 4481 uid165043
Escherichia coli O55 H7 CB9615 uid46655
Escherichia coli O157 H7 TW14359 uid59235
Escherichia coli O157 H7 Sakai uid57781
Escherichia coli O157 H7 EDL933 uid57831
Escherichia coli O157 H7 EC4115 uid59091
Escherichia coli O55 H7 RM12579 uid162153
Escherichia coli Xuzhou21 uid163995
Shigella flexneri 2a 301 uid62907
Escherichia coli UM146 uid162043
Escherichia coli SMS 3 5 uid58919
Escherichia coli SE15 uid161939
Escherichia coli O83 H1 NRG 857C uid161987
Escherichia coli O127 H6 E2348 69 uid59343
Escherichia coli NA114 uid162139
Escherichia coli LF82 uid161965
Escherichia coli IHE3034 uid162007
Escherichia coli CFT073 uid57915
Escherichia coli APEC O1 uid58623
Escherichia coli ABU 83972 uid161975
Escherichia coli 536 uid58531
Escherichia coli 042 uid161985
Escherichia coli UTI89 uid58541
Escherichia coli clone D i14 uid162049
Escherichia coli clone D i2 uid162047
Shigella dysenteriae Sd197 uid58213
Shigella boydii Sb227 uid58215
Shigella boydii CDC 3083 94 uid58415
Shigella flexneri 2002017 uid159233
Shigella flexneri 2a 2457T uid57991
Escherichia coli BL21 Gold DE3 pLysS AG uid59245
Shigella flexneri 5 8401 uid58583
Escherichia coli W uid162101
Escherichia coli UMNK88 uid161991
Escherichia coli SE11 uid59425
Escherichia coli P12b uid162061
Escherichia coli O7 K1 CE10 uid162115
Escherichia coli O26 H11 11368 uid41021
Escherichia coli O111 H 11128 uid41023
Escherichia coli O103 H2 12009 uid41013
Escherichia coli K 12 substr W3110 uid161931
Escherichia coli K 12 substr DH10B uid58979
Escherichia coli KO11FL uid52593
Escherichia coli KO11FL uid162099
Escherichia coli HS uid58393
Escherichia coli ETEC H10407 uid161993
Escherichia coli E24377A uid58395
Escherichia coli DH1 uid162051
Escherichia coli DH1 uid161951
Escherichia coli B REL606 uid58803
Escherichia coli BL21 DE3 uid161949
Escherichia coli BL21 DE3 uid161947
Escherichia coli ATCC 8739 uid58783
Shigella sonnei 53G uid84383
Shigella sonnei Ss046 uid58217
rplP

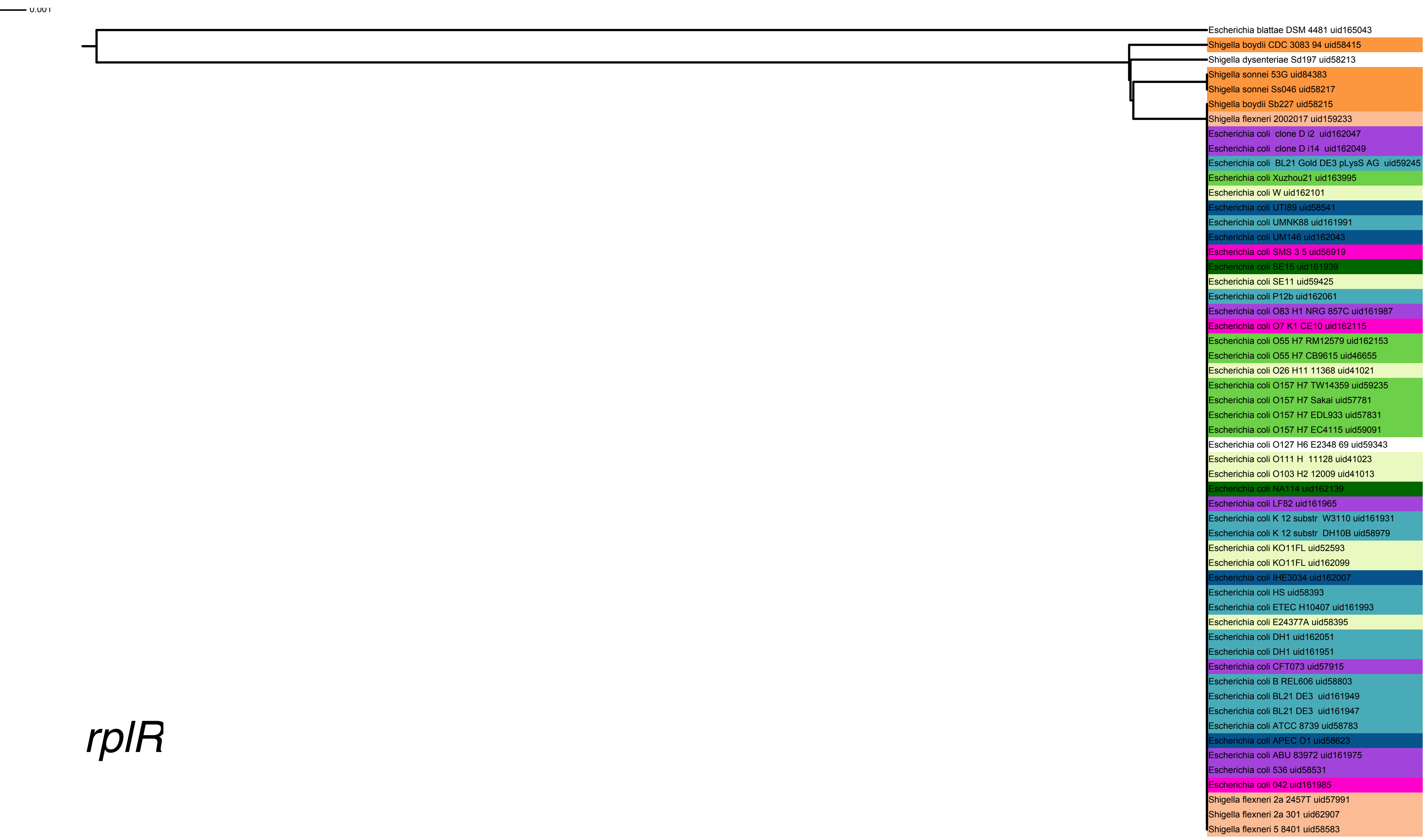

0.001
Escherichia blattae DSM 4481 uid165043
Shigella boydii CDC 3083 94 uid58415
Shigella dysenteriae Sd197 uid58213
Shigella sonnei 53G uid84383
Shigella sonnei Ss046 uid58217
Shigella boydii Sb227 uid58215
Shigella flexneri 2002017 uid159233
Escherichia coli clone D i2 uid162047
Escherichia coli clone D i14 uid162049
Escherichia coli BL21 Gold DE3 pLysS AG uid59245
Escherichia coli Xuzhou21 uid163995
Escherichia coli W uid162101
Escherichia coli UTI89 uid58541
Escherichia coli UMNK88 uid161991
Escherichia coli UM146 uid162043
Escherichia coli SMS 3 5 uid58919
Escherichia coli SE15 uid161939
Escherichia coli SE11 uid59425
Escherichia coli P12b uid162061
Escherichia coli O83 H1 NRG 857C uid161987
Escherichia coli O7 K1 CE10 uid162115
Escherichia coli O55 H7 RM12579 uid162153
Escherichia coli O55 H7 CB9615 uid46655
Escherichia coli O26 H11 11368 uid41021
Escherichia coli O157 H7 TW14359 uid59235
Escherichia coli O157 H7 Sakai uid57781
Escherichia coli O157 H7 EDL933 uid57831
Escherichia coli O157 H7 EC4115 uid59091
Escherichia coli O127 H6 E2348 69 uid59343
Escherichia coli O111 H 11128 uid41023
Escherichia coli O103 H2 12009 uid41013
Escherichia coli NA114 uid162139
Escherichia coli LF82 uid161965
Escherichia coli K 12 substr W3110 uid161931
Escherichia coli K 12 substr DH10B uid58979
Escherichia coli KO11FL uid52593
Escherichia coli KO11FL uid162099
Escherichia coli IHE3034 uid162007
Escherichia coli HS uid58393
Escherichia coli ETEC H10407 uid161993
Escherichia coli E24377A uid58395
Escherichia coli DH1 uid162051
Escherichia coli DH1 uid161951
Escherichia coli CFT073 uid57915
Escherichia coli B REL606 uid58803
Escherichia coli BL21 DE3 uid161949
Escherichia coli BL21 DE3 uid161947
Escherichia coli ATCC 8739 uid58783
Escherichia coli APEC O1 uid58623
Escherichia coli ABU 83972 uid161975
Escherichia coli 536 uid58531
Escherichia coli 042 uid161985
Shigella flexneri 2a 2457T uid57991
Shigella flexneri 2a 301 uid62907
Shigella flexneri 5 8401 uid58583
rplR

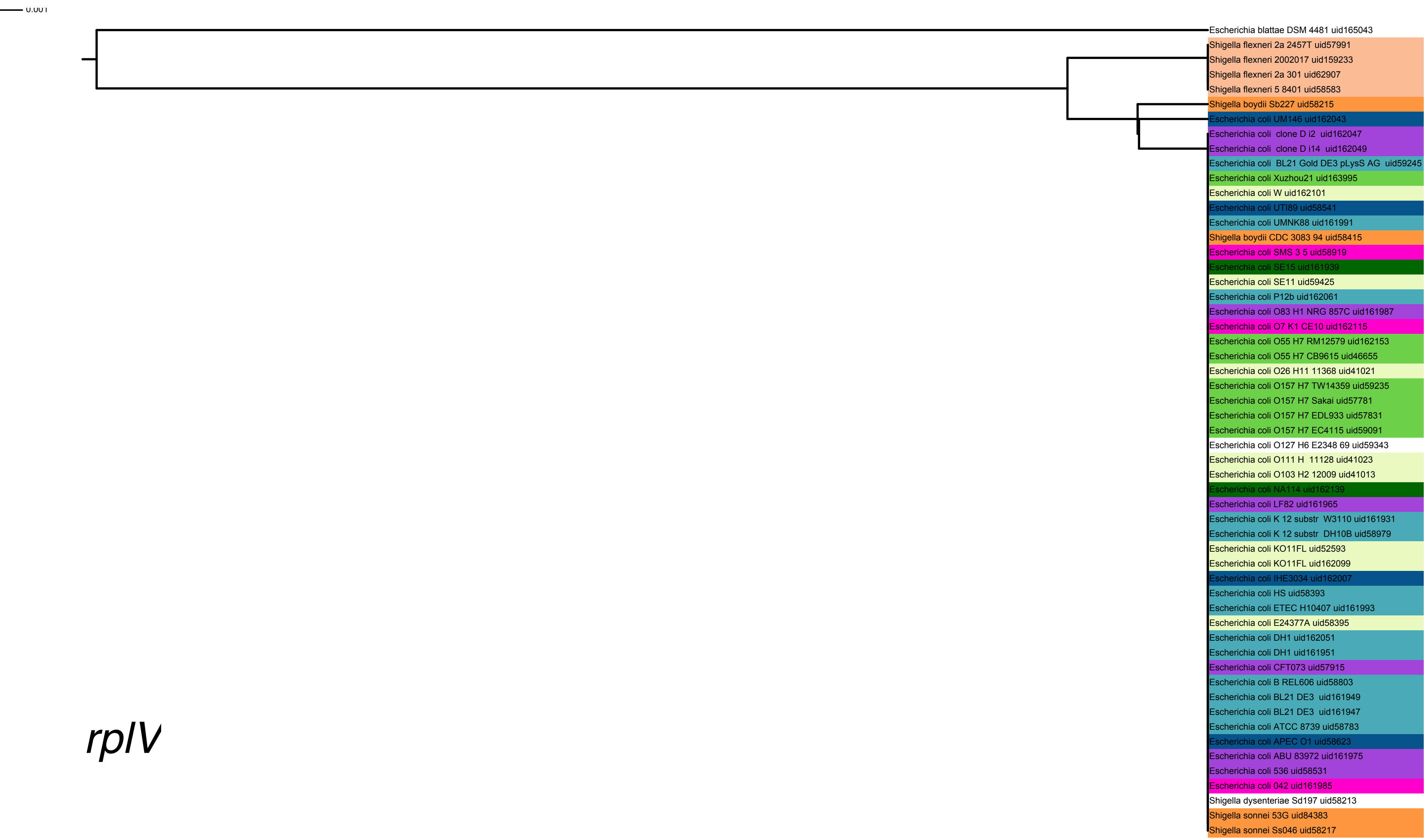
0.001
Escherichia blattae DSM 4481 uid165043
Shigella flexneri 2a 2457T uid57991
Shigella flexneri 2002017 uid159233
Shigella flexneri 2a 301 uid62907
Shigella flexneri 5 8401 uid58583
Shigella boydii Sb227 uid58215
Escherichia coli UM146 uid162043
Escherichia coli clone D i2 uid162047
Escherichia coli clone D i14 uid162049
Escherichia coli BL21 Gold DE3 pLysS AG uid59245
Escherichia coli Xuzhou21 uid163995
Escherichia coli W uid162101
Escherichia coli UTI89 uid58541
Escherichia coli UMNK88 uid161991
Shigella boydii CDC 3083 94 uid58415
Escherichia coli SMS 3 5 uid58919
Escherichia coli SE15 uid161939
Escherichia coli SE11 uid59425
Escherichia coli P12b uid162061
Escherichia coli O83 H1 NRG 857C uid161987
Escherichia coli O7 K1 CE10 uid162115
Escherichia coli O55 H7 RM12579 uid162153
Escherichia coli O55 H7 CB9615 uid46655
Escherichia coli O26 H11 11368 uid41021
Escherichia coli O157 H7 TW14359 uid59235
Escherichia coli O157 H7 Sakai uid57781
Escherichia coli O157 H7 EDL933 uid57831
Escherichia coli O157 H7 EC4115 uid59091
Escherichia coli O127 H6 E2348 69 uid59343
Escherichia coli O111 H 11128 uid41023
Escherichia coli O103 H2 12009 uid41013
Escherichia coli NA114 uid162139
Escherichia coli LF82 uid161965
Escherichia coli K 12 substr W3110 uid161931
Escherichia coli K 12 substr DH10B uid58979
Escherichia coli KO11FL uid52593
Escherichia coli KO11FL uid162099
Escherichia coli IHE3034 uid162007
Escherichia coli HS uid58393
Escherichia coli ETEC H10407 uid161993
Escherichia coli E24377A uid58395
Escherichia coli DH1 uid162051
Escherichia coli DH1 uid161951
Escherichia coli CFT073 uid57915
Escherichia coli B REL606 uid58803
Escherichia coli BL21 DE3 uid161949
Escherichia coli BL21 DE3 uid161947
Escherichia coli ATCC 8739 uid58783
Escherichia coli APEC O1 uid58623
Escherichia coli ABU 83972 uid161975
Escherichia coli 536 uid58531
Escherichia coli 042 uid161985
Shigella dysenteriae Sd197 uid58213
Shigella sonnei 53G uid84383
Shigella sonnei Ss046 uid58217
rplV

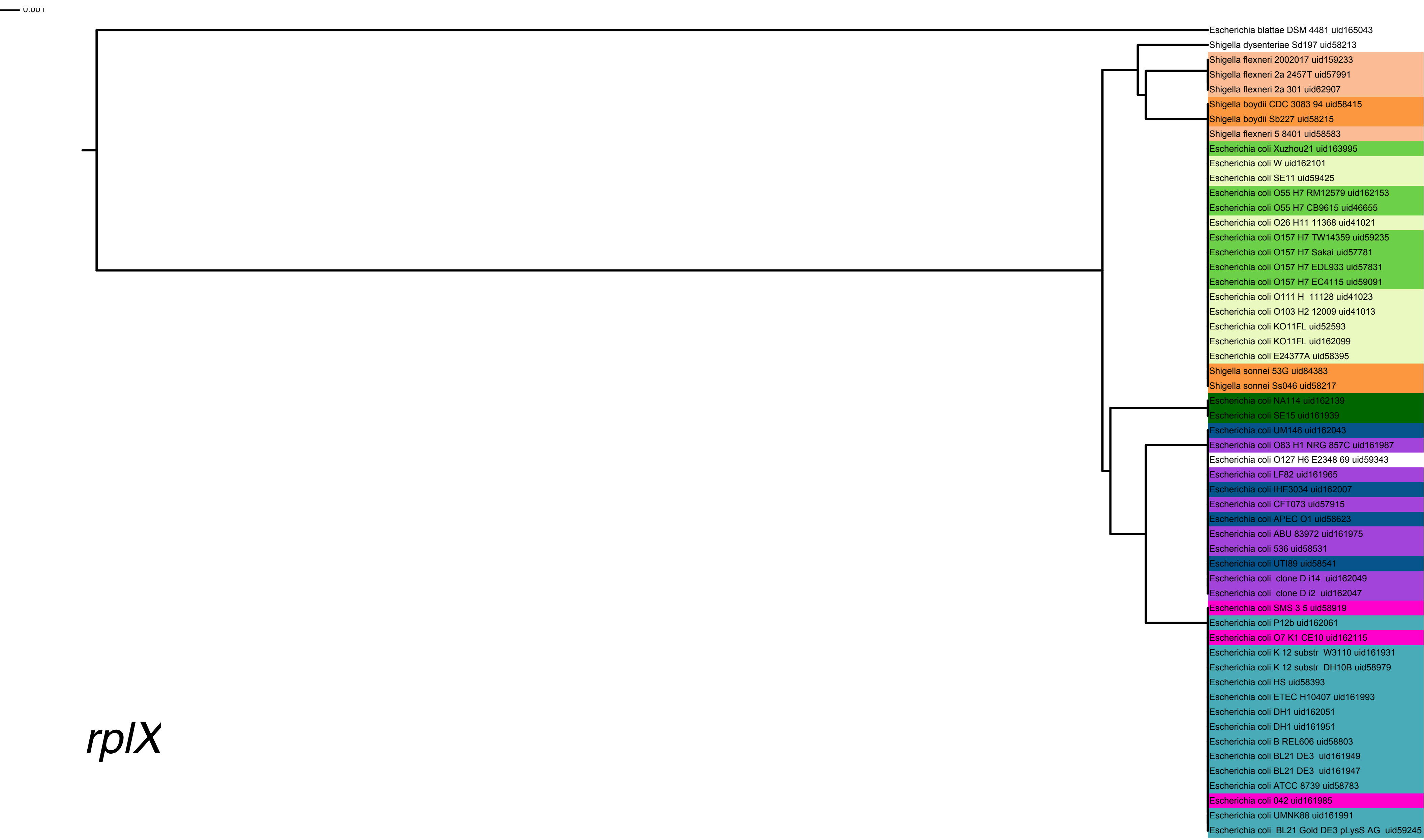
0.001
Escherichia blattae DSM 4481 uid165043
Shigella dysenteriae Sd197 uid58213
Shigella flexneri 2002017 uid159233
Shigella flexneri 2a 2457T uid57991
Shigella flexneri 2a 301 uid62907
Shigella boydii CDC 3083 94 uid58415
Shigella boydii Sb227 uid58215
Shigella flexneri 5 8401 uid58583
Escherichia coli Xuzhou21 uid163995
Escherichia coli W uid162101
Escherichia coli SE11 uid59425
Escherichia coli O55 H7 RM12579 uid162153
Escherichia coli O55 H7 CB9615 uid46655
Escherichia coli O26 H11 11368 uid41021
Escherichia coli O157 H7 TW14359 uid59235
Escherichia coli O157 H7 Sakai uid57781
Escherichia coli O157 H7 EDL933 uid57831
Escherichia coli O157 H7 EC4115 uid59091
Escherichia coli O111 H 11128 uid41023
Escherichia coli O103 H2 12009 uid41013
Escherichia coli KO11FL uid52593
Escherichia coli KO11FL uid162099
Escherichia coli E24377A uid58395
Shigella sonnei 53G uid84383
Shigella sonnei Ss046 uid58217
Escherichia coli NA114 uid162139
Escherichia coli SE15 uid161939
Escherichia coli UM146 uid162043
Escherichia coli O83 H1 NRG 857C uid161987
Escherichia coli O127 H6 E2348 69 uid59343
Escherichia coli LF82 uid161965
Escherichia coli IHE3034 uid162007
Escherichia coli CFT073 uid57915
Escherichia coli APEC O1 uid58623
Escherichia coli ABU 83972 uid161975
Escherichia coli 536 uid58531
Escherichia coli UTI89 uid58541
Escherichia coli clone D i14 uid162049
Escherichia coli clone D i2 uid162047
Escherichia coli SMS 3 5 uid58919
Escherichia coli P12b uid162061
Escherichia coli O7 K1 CE10 uid162115
Escherichia coli K 12 substr W3110 uid161931
Escherichia coli K 12 substr DH10B uid58979
Escherichia coli HS uid58393
Escherichia coli ETEC H10407 uid161993
Escherichia coli DH1 uid162051
Escherichia coli DH1 uid161951
Escherichia coli B REL606 uid58803
Escherichia coli BL21 DE3 uid161949
Escherichia coli BL21 DE3 uid161947
Escherichia coli ATCC 8739 uid58783
Escherichia coli 042 uid161985
Escherichia coli UMNK88 uid161991
Escherichia coli BL21 Gold DE3 pLysS AG uid59245
rplX

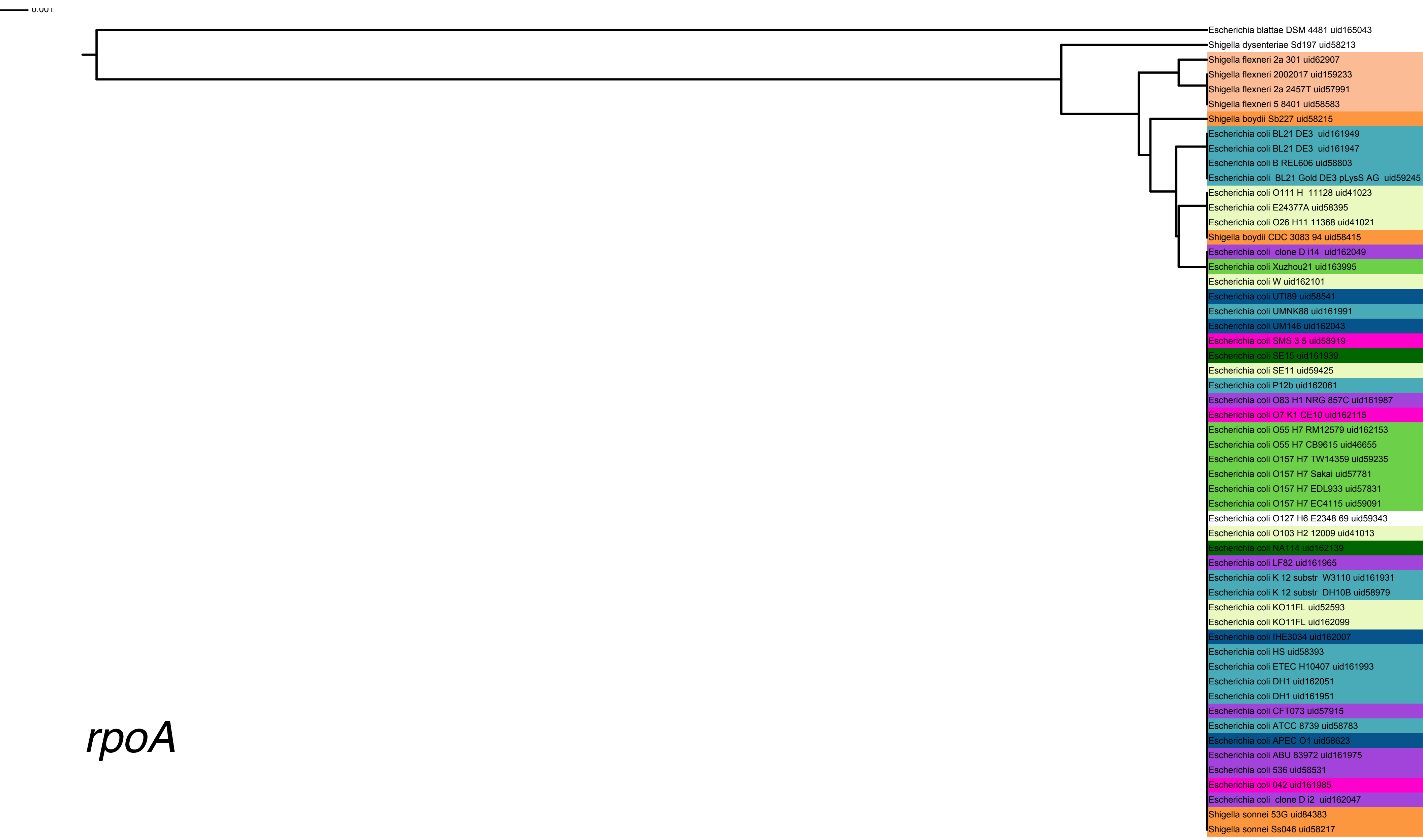
0.001
Escherichia blattae DSM 4481 uid165043
Shigella dysenteriae Sd197 uid58213
Shigella flexneri 2a 301 uid62907
Shigella flexneri 2002017 uid159233
Shigella flexneri 2a 2457T uid57991
Shigella flexneri 5 8401 uid58583
Shigella boydii Sb227 uid58215
Escherichia coli BL21 DE3 uid161949
Escherichia coli BL21 DE3 uid161947
Escherichia coli B REL606 uid58803
Escherichia coli BL21 Gold DE3 pLysS AG uid59245
Escherichia coli O111 H 11128 uid41023
Escherichia coli E24377A uid58395
Escherichia coli O26 H11 11368 uid41021
Shigella boydii CDC 3083 94 uid58415
Escherichia coli clone D i14 uid162049
Escherichia coli Xuzhou21 uid163995
Escherichia coli W uid162101
Escherichia coli UTI89 uid58541
Escherichia coli UMNK88 uid161991
Escherichia coli UM146 uid162043
Escherichia coli SMS 3 5 uid58919
Escherichia coli SE15 uid161939
Escherichia coli SE11 uid59425
Escherichia coli P12b uid162061
Escherichia coli O83 H1 NRG 857C uid161987
Escherichia coli O7 K1 CE10 uid162115
Escherichia coli O55 H7 RM12579 uid162153
Escherichia coli O55 H7 CB9615 uid46655
Escherichia coli O157 H7 TW14359 uid59235
Escherichia coli O157 H7 Sakai uid57781
Escherichia coli O157 H7 EDL933 uid57831
Escherichia coli O157 H7 EC4115 uid59091
Escherichia coli O127 H6 E2348 69 uid59343
Escherichia coli O103 H2 12009 uid41013
Escherichia coli NA114 uid162139
Escherichia coli LF82 uid161965
Escherichia coli K 12 substr W3110 uid161931
Escherichia coli K 12 substr DH10B uid58979
Escherichia coli KO11FL uid52593
Escherichia coli KO11FL uid162099
Escherichia coli IHE3034 uid162007
Escherichia coli HS uid58393
Escherichia coli ETEC H10407 uid161993
Escherichia coli DH1 uid162051
Escherichia coli DH1 uid161951
Escherichia coli CFT073 uid57915
Escherichia coli ATCC 8739 uid58783
Escherichia coli APEC O1 uid58623
Escherichia coli ABU 83972 uid161975
Escherichia coli 536 uid58531
Escherichia coli 042 uid161985
Escherichia coli clone D i2 uid162047
Shigella sonnei 53G uid84383
Shigella sonnei Ss046 uid58217
rpoA

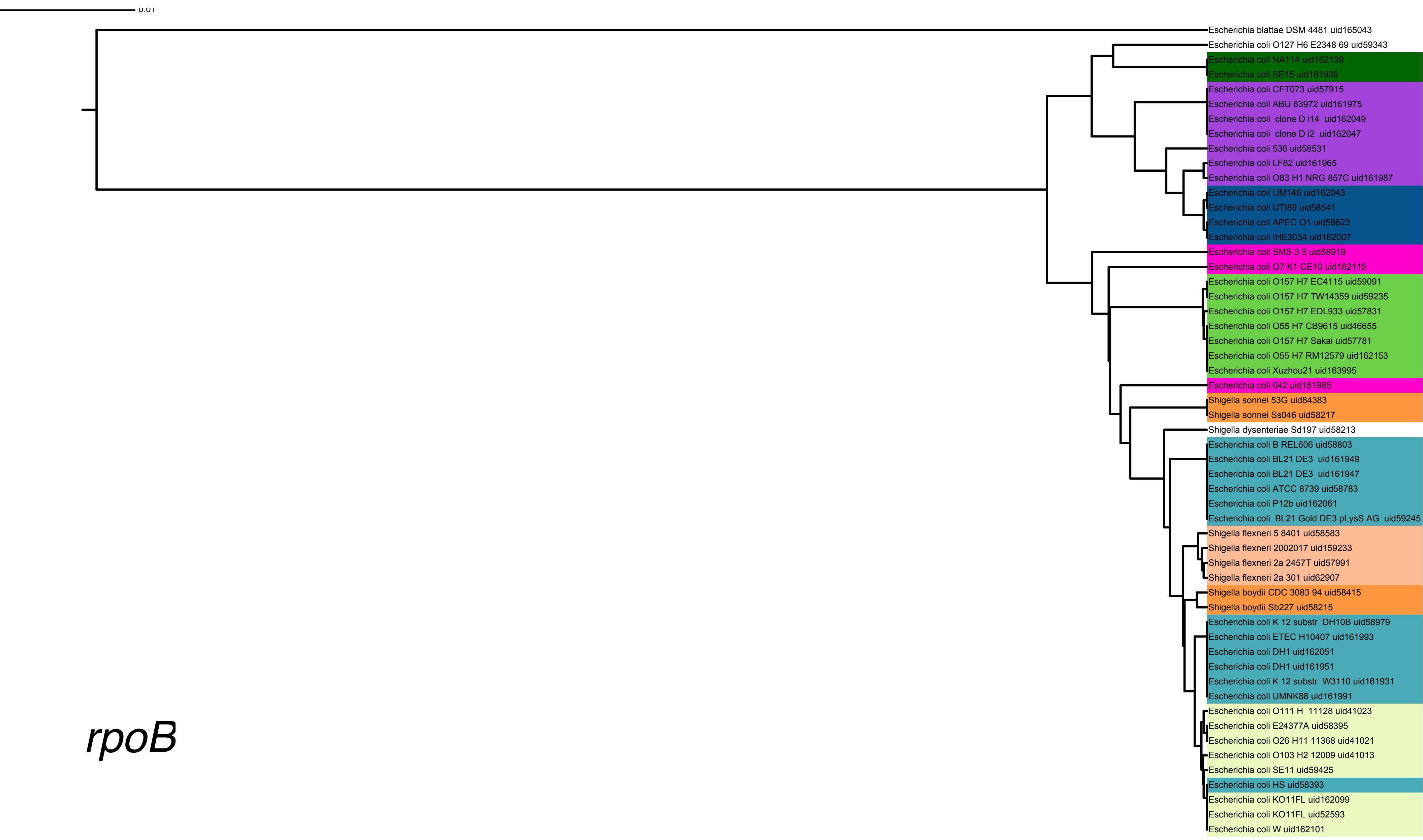
0.01
Escherichia blattae DSM 4481 uid165043
Escherichia coli O127 H6 E2348 69 uid59343
Escherichia coli NA114 uid162139
Escherichia coli SE15 uid161939
Escherichia coli CFT073 uid57915
Escherichia coli ABU 83972 uid161975
Escherichia coli clone D i14 uid162049
Escherichia coli clone D i2 uid162047
Escherichia coli 536 uid58531
Escherichia coli LF82 uid161965
Escherichia coli O83 H1 NRG 857C uid161987
Escherichia coli UM146 uid162043
Escherichia coli UTI89 uid58541
Escherichia coli APEC O1 uid58623
Escherichia coli IHE3034 uid162007
Escherichia coli SMS 3 5 uid58919
Escherichia coli O7 K1 CE10 uid162115
Escherichia coli O157 H7 EC4115 uid59091
Escherichia coli O157 H7 TW14359 uid59235
Escherichia coli O157 H7 EDL933 uid57831
Escherichia coli O55 H7 CB9615 uid46655
Escherichia coli O157 H7 Sakai uid57781
Escherichia coli O55 H7 RM12579 uid162153
Escherichia coli Xuzhou21 uid163995
Escherichia coli 042 uid161985
Shigella sonnei 53G uid84383
Shigella sonnei Ss046 uid58217
Shigella dysenteriae Sd197 uid58213
Escherichia coli B REL606 uid58803
Escherichia coli BL21 DE3 uid161949
Escherichia coli BL21 DE3 uid161947
Escherichia coli ATCC 8739 uid58783
Escherichia coli P12b uid162061
Escherichia coli BL21 Gold DE3 pLysS AG uid59245
Shigella flexneri 5 8401 uid58583
Shigella flexneri 2002017 uid159233
Shigella flexneri 2a 2457T uid57991
Shigella flexneri 2a 301 uid62907
Shigella boydii CDC 3083 94 uid58415
Shigella boydii Sb227 uid58215
Escherichia coli K 12 substr DH10B uid58979
Escherichia coli ETEC H10407 uid161993
Escherichia coli DH1 uid162051
Escherichia coli DH1 uid161951
Escherichia coli K 12 substr W3110 uid161931
Escherichia coli UMNK88 uid161991
Escherichia coli O111 H 11128 uid41023
Escherichia coli E24377A uid58395
Escherichia coli O26 H11 11368 uid41021
Escherichia coli O103 H2 12009 uid41013
Escherichia coli SE11 uid59425
Escherichia coli HS uid58393
Escherichia coli KO11FL uid162099
Escherichia coli KO11FL uid52593
Escherichia coli W uid162101
rpoB

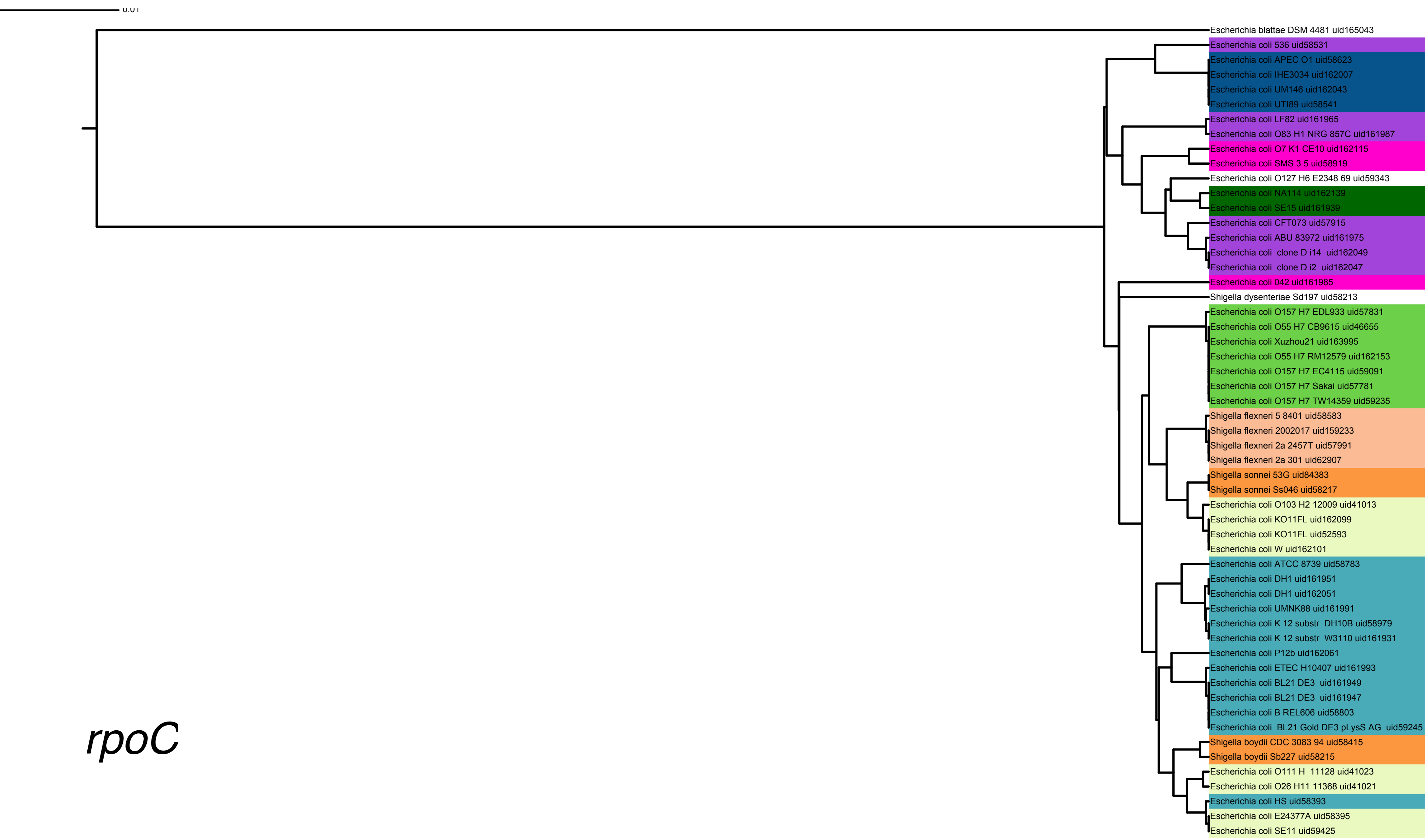

0.01
Escherichia blattae DSM 4481 uid165043
Escherichia coli 536 uid58531
Escherichia coli APEC O1 uid58623
Escherichia coli IHE3034 uid162007
Escherichia coli UM146 uid162043
Escherichia coli UTI89 uid58541
Escherichia coli LF82 uid161965
Escherichia coli O83 H1 NRG 857C uid161987
Escherichia coli O7 K1 CE10 uid162115
Escherichia coli SMS 3 5 uid58919
Escherichia coli O127 H6 E2348 69 uid59343
Escherichia coli NA114 uid162139
Escherichia coli SE15 uid161939
Escherichia coli CFT073 uid57915
Escherichia coli ABU 83972 uid161975
Escherichia coli clone D i14 uid162049
Escherichia coli clone D i2 uid162047
Escherichia coli 042 uid161985
Shigella dysenteriae Sd197 uid58213
Escherichia coli O157 H7 EDL933 uid57831
Escherichia coli O55 H7 CB9615 uid46655
Escherichia coli Xuzhou21 uid163995
Escherichia coli O55 H7 RM12579 uid162153
Escherichia coli O157 H7 EC4115 uid59091
Escherichia coli O157 H7 Sakai uid57781
Escherichia coli O157 H7 TW14359 uid59235
Shigella flexneri 5 8401 uid58583
Shigella flexneri 2002017 uid159233
Shigella flexneri 2a 2457T uid57991
Shigella flexneri 2a 301 uid62907
Shigella sonnei 53G uid84383
Shigella sonnei Ss046 uid58217
Escherichia coli O103 H2 12009 uid41013
Escherichia coli KO11FL uid162099
Escherichia coli KO11FL uid52593
Escherichia coli W uid162101
Escherichia coli ATCC 8739 uid58783
Escherichia coli DH1 uid161951
Escherichia coli DH1 uid162051
Escherichia coli UMNK88 uid161991
Escherichia coli K 12 substr DH10B uid58979
Escherichia coli K 12 substr W3110 uid161931
Escherichia coli P12b uid162061
Escherichia coli ETEC H10407 uid161993
Escherichia coli BL21 DE3 uid161949
Escherichia coli BL21 DE3 uid161947
Escherichia coli B REL606 uid58803
Escherichia coli BL21 Gold DE3 pLysS AG uid59245
Shigella boydii CDC 3083 94 uid58415
Shigella boydii Sb227 uid58215
Escherichia coli O111 H 11128 uid41023
Escherichia coli O26 H11 11368 uid41021
Escherichia coli HS uid58393
Escherichia coli E24377A uid58395
Escherichia coli SE11 uid59425
rpoC

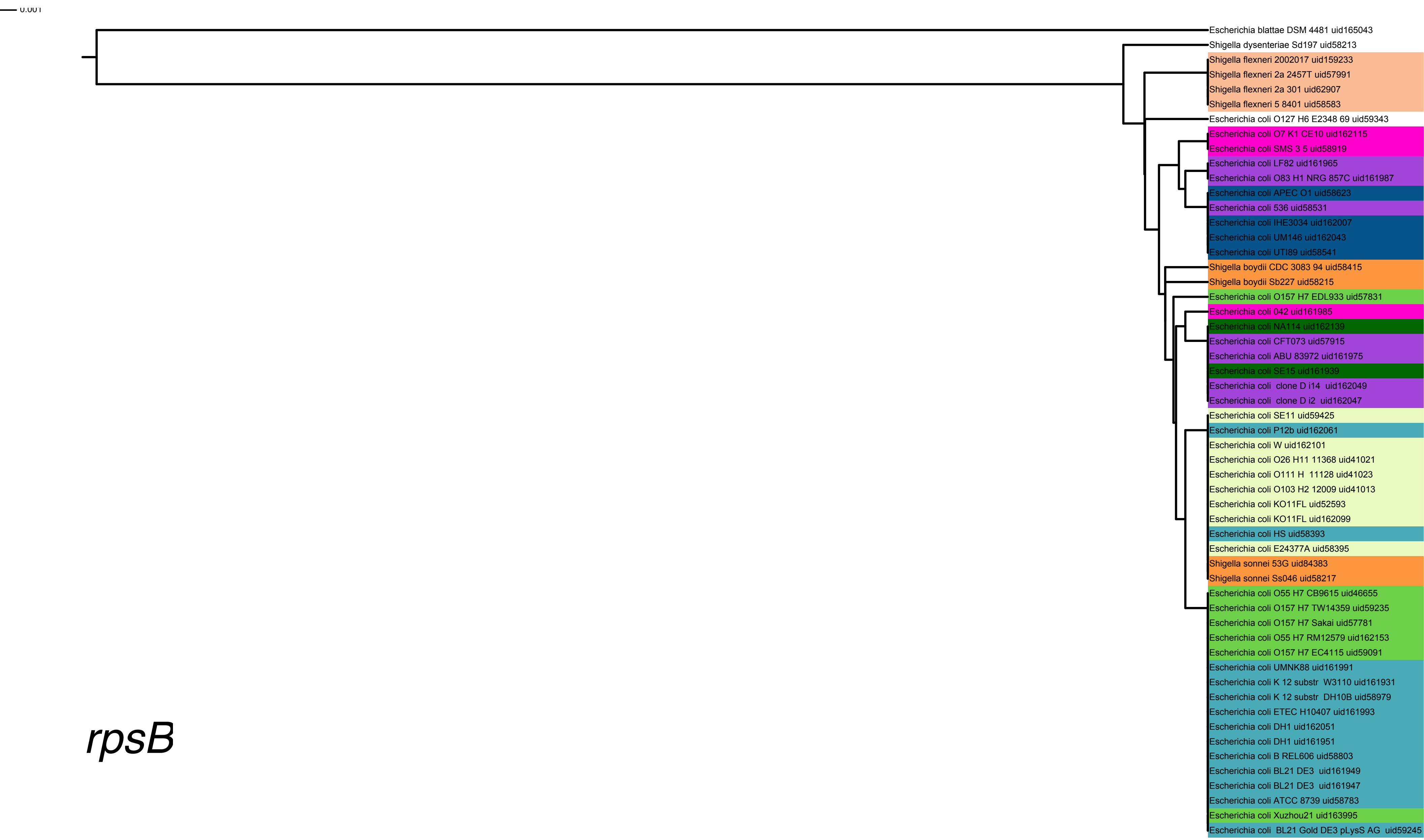

0.001
Escherichia blattae DSM 4481 uid165043
Shigella dysenteriae Sd197 uid58213
Shigella flexneri 2002017 uid159233
Shigella flexneri 2a 2457T uid57991
Shigella flexneri 2a 301 uid62907
Shigella flexneri 5 8401 uid58583
Escherichia coli O127 H6 E2348 69 uid59343
Escherichia coli O7 K1 CE10 uid162115
Escherichia coli SMS 3 5 uid58919
Escherichia coli LF82 uid161965
Escherichia coli O83 H1 NRG 857C uid161987
Escherichia coli APEC O1 uid58623
Escherichia coli 536 uid58531
Escherichia coli IHE3034 uid162007
Escherichia coli UM146 uid162043
Escherichia coli UTI89 uid58541
Shigella boydii CDC 3083 94 uid58415
Shigella boydii Sb227 uid58215
Escherichia coli O157 H7 EDL933 uid57831
Escherichia coli 042 uid161985
Escherichia coli NA114 uid162139
Escherichia coli CFT073 uid57915
Escherichia coli ABU 83972 uid161975
Escherichia coli SE15 uid161939
Escherichia coli clone D i14 uid162049
Escherichia coli clone D i2 uid162047
Escherichia coli SE11 uid59425
Escherichia coli P12b uid162061
Escherichia coli W uid162101
Escherichia coli O26 H11 11368 uid41021
Escherichia coli O111 H 11128 uid41023
Escherichia coli O103 H2 12009 uid41013
Escherichia coli KO11FL uid52593
Escherichia coli KO11FL uid162099
Escherichia coli HS uid58393
Escherichia coli E24377A uid58395
Shigella sonnei 53G uid84383
Shigella sonnei Ss046 uid58217
Escherichia coli O55 H7 CB9615 uid46655
Escherichia coli O157 H7 TW14359 uid59235
Escherichia coli O157 H7 Sakai uid57781
Escherichia coli O55 H7 RM12579 uid162153
Escherichia coli O157 H7 EC4115 uid59091
Escherichia coli UMNK88 uid161991
Escherichia coli K 12 substr W3110 uid161931
Escherichia coli K 12 substr DH10B uid58979
Escherichia coli ETEC H10407 uid161993
Escherichia coli DH1 uid162051
Escherichia coli DH1 uid161951
Escherichia coli B REL606 uid58803
Escherichia coli BL21 DE3 uid161949
Escherichia coli BL21 DE3 uid161947
Escherichia coli ATCC 8739 uid58783
Escherichia coli Xuzhou21 uid163995
Escherichia coli BL21 Gold DE3 pLysS AG uid59245
rpsB

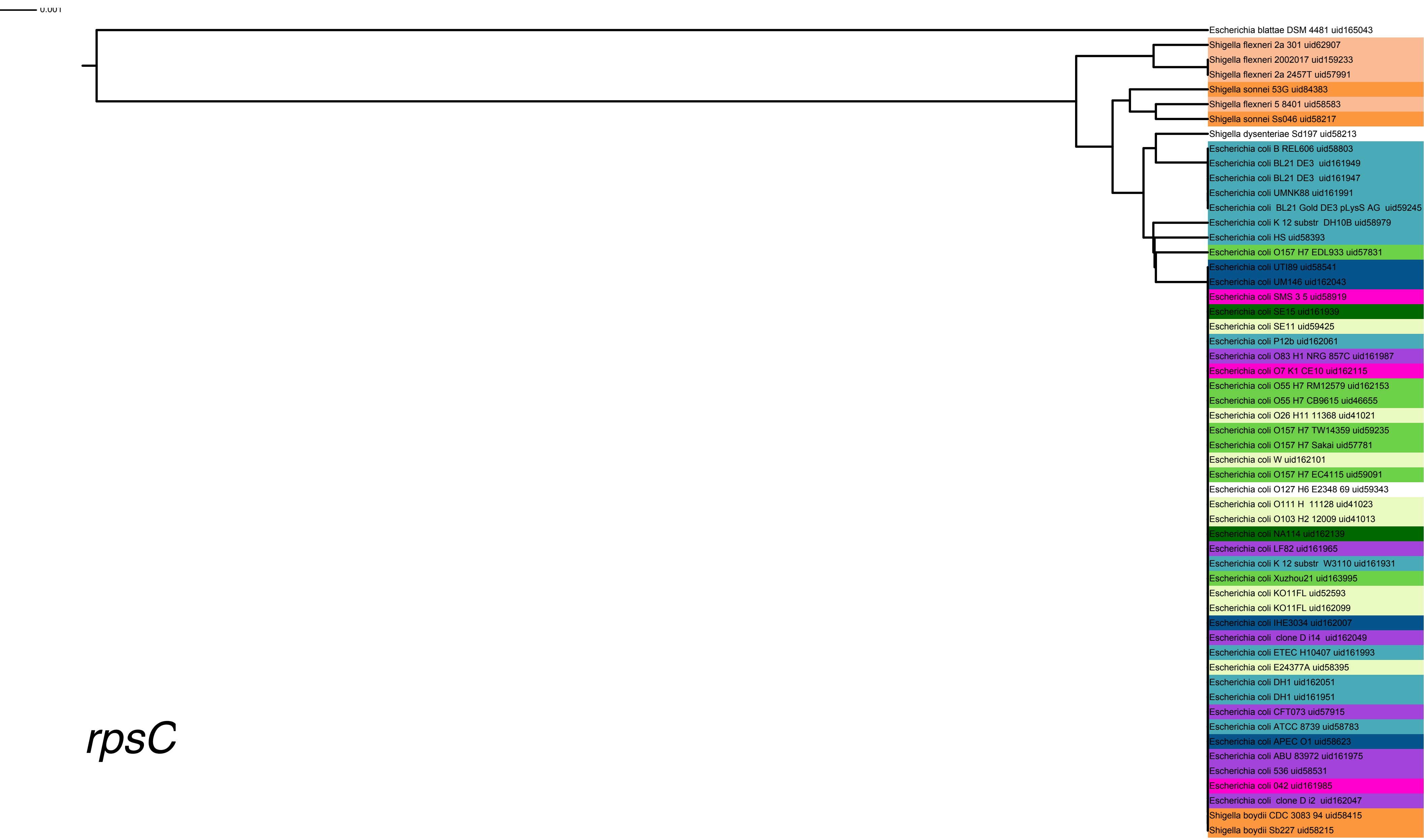
0.001
Escherichia blattae DSM 4481 uid165043
Shigella flexneri 2a 301 uid62907
Shigella flexneri 2002017 uid159233
Shigella flexneri 2a 2457T uid57991
Shigella sonnei 53G uid84383
Shigella flexneri 5 8401 uid58583
Shigella sonnei Ss046 uid58217
Shigella dysenteriae Sd197 uid58213
Escherichia coli B REL606 uid58803
Escherichia coli BL21 DE3 uid161949
Escherichia coli BL21 DE3 uid161947
Escherichia coli UMNK88 uid161991
Escherichia coli BL21 Gold DE3 pLysS AG uid59245
Escherichia coli K 12 substr DH10B uid58979
Escherichia coli HS uid58393
Escherichia coli O157 H7 EDL933 uid57831
Escherichia coli UTI89 uid58541
Escherichia coli UM146 uid162043
Escherichia coli SMS 3 5 uid58919
Escherichia coli SE15 uid161939
Escherichia coli SE11 uid59425
Escherichia coli P12b uid162061
Escherichia coli O83 H1 NRG 857C uid161987
Escherichia coli O7 K1 CE10 uid162115
Escherichia coli O55 H7 RM12579 uid162153
Escherichia coli O55 H7 CB9615 uid46655
Escherichia coli O26 H11 11368 uid41021
Escherichia coli O157 H7 TW14359 uid59235
Escherichia coli O157 H7 Sakai uid57781
Escherichia coli W uid162101
Escherichia coli O157 H7 EC4115 uid59091
Escherichia coli O127 H6 E2348 69 uid59343
Escherichia coli O111 H 11128 uid41023
Escherichia coli O103 H2 12009 uid41013
Escherichia coli NA114 uid162139
Escherichia coli LF82 uid161965
Escherichia coli K 12 substr W3110 uid161931
Escherichia coli Xuzhou21 uid163995
Escherichia coli KO11FL uid52593
Escherichia coli KO11FL uid162099
Escherichia coli IHE3034 uid162007
Escherichia coli clone D i14 uid162049
Escherichia coli ETEC H10407 uid161993
Escherichia coli E24377A uid58395
Escherichia coli DH1 uid162051
Escherichia coli DH1 uid161951
Escherichia coli CFT073 uid57915
Escherichia coli ATCC 8739 uid58783
Escherichia coli APEC O1 uid58623
Escherichia coli ABU 83972 uid161975
Escherichia coli 536 uid58531
Escherichia coli 042 uid161985
Escherichia coli clone D i2 uid162047
Shigella boydii CDC 3083 94 uid58415
Shigella boydii Sb227 uid58215
rpsC

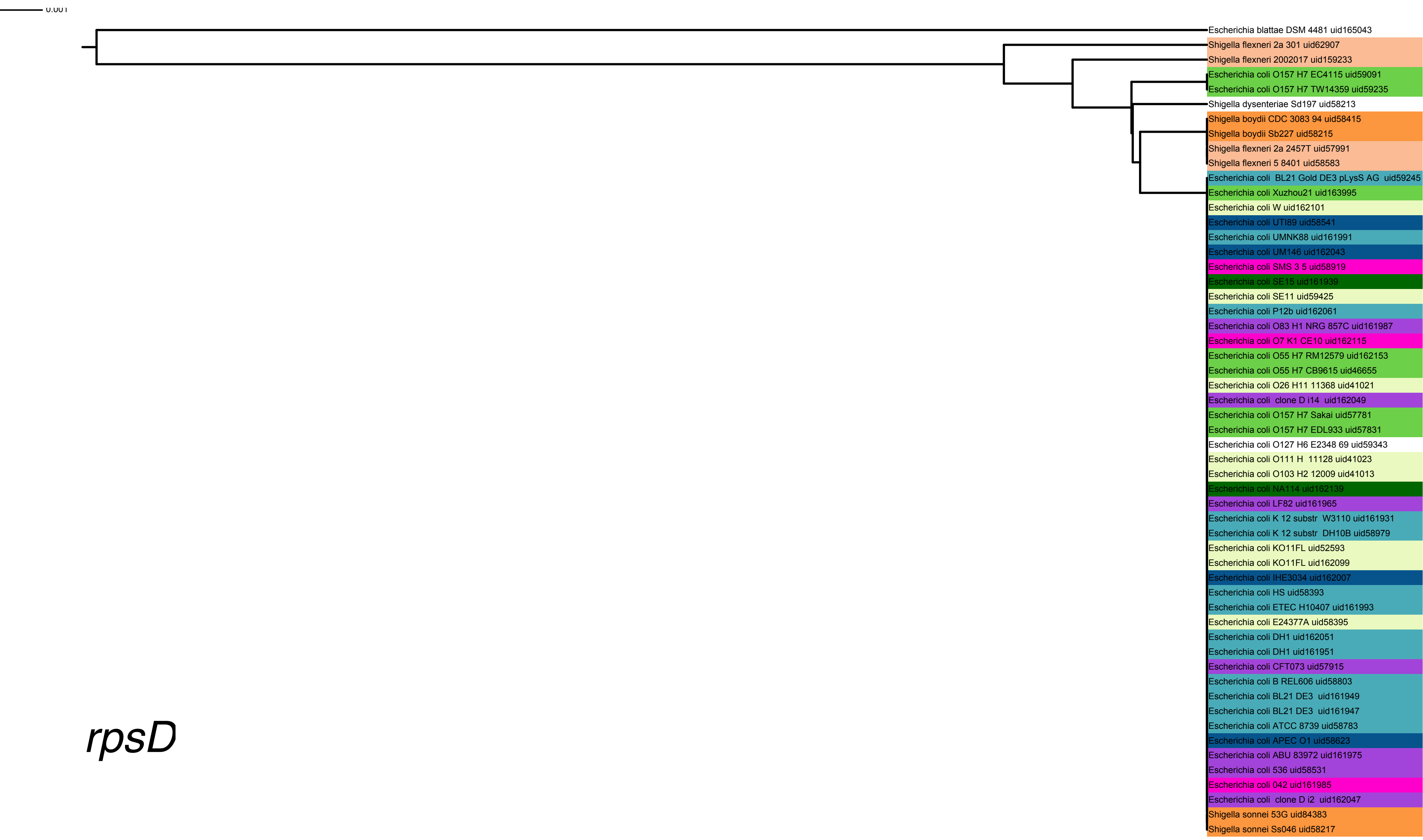

0.001
Escherichia blattae DSM 4481 uid165043
Shigella flexneri 2a 301 uid62907
Shigella flexneri 2002017 uid159233
Escherichia coli O157 H7 EC4115 uid59091
Escherichia coli O157 H7 TW14359 uid59235
Shigella dysenteriae Sd197 uid58213
Shigella boydii CDC 3083 94 uid58415
Shigella boydii Sb227 uid58215
Shigella flexneri 2a 2457T uid57991
Shigella flexneri 5 8401 uid58583
Escherichia coli BL21 Gold DE3 pLysS AG uid59245
Escherichia coli Xuzhou21 uid163995
Escherichia coli W uid162101
Escherichia coli UTI89 uid58541
Escherichia coli UMNK88 uid161991
Escherichia coli UM146 uid162043
Escherichia coli SMS 3 5 uid58919
Escherichia coli SE15 uid161939
Escherichia coli SE11 uid59425
Escherichia coli P12b uid162061
Escherichia coli O83 H1 NRG 857C uid161987
Escherichia coli O7 K1 CE10 uid162115
Escherichia coli O55 H7 RM12579 uid162153
Escherichia coli O55 H7 CB9615 uid46655
Escherichia coli O26 H11 11368 uid41021
Escherichia coli clone D i14 uid162049
Escherichia coli O157 H7 Sakai uid57781
Escherichia coli O157 H7 EDL933 uid57831
Escherichia coli O127 H6 E2348 69 uid59343
Escherichia coli O111 H 11128 uid41023
Escherichia coli O103 H2 12009 uid41013
Escherichia coli NA114 uid162139
Escherichia coli LF82 uid161965
Escherichia coli K 12 substr W3110 uid161931
Escherichia coli K 12 substr DH10B uid58979
Escherichia coli KO11FL uid52593
Escherichia coli KO11FL uid162099
Escherichia coli IHE3034 uid162007
Escherichia coli HS uid58393
Escherichia coli ETEC H10407 uid161993
Escherichia coli E24377A uid58395
Escherichia coli DH1 uid162051
Escherichia coli DH1 uid161951
Escherichia coli CFT073 uid57915
Escherichia coli B REL606 uid58803
Escherichia coli BL21 DE3 uid161949
Escherichia coli BL21 DE3 uid161947
Escherichia coli ATCC 8739 uid58783
Escherichia coli APEC O1 uid58623
Escherichia coli ABU 83972 uid161975
Escherichia coli 536 uid58531
Escherichia coli 042 uid161985
Escherichia coli clone D i2 uid162047
Shigella sonnei 53G uid84383
Shigella sonnei Ss046 uid58217
rpsD

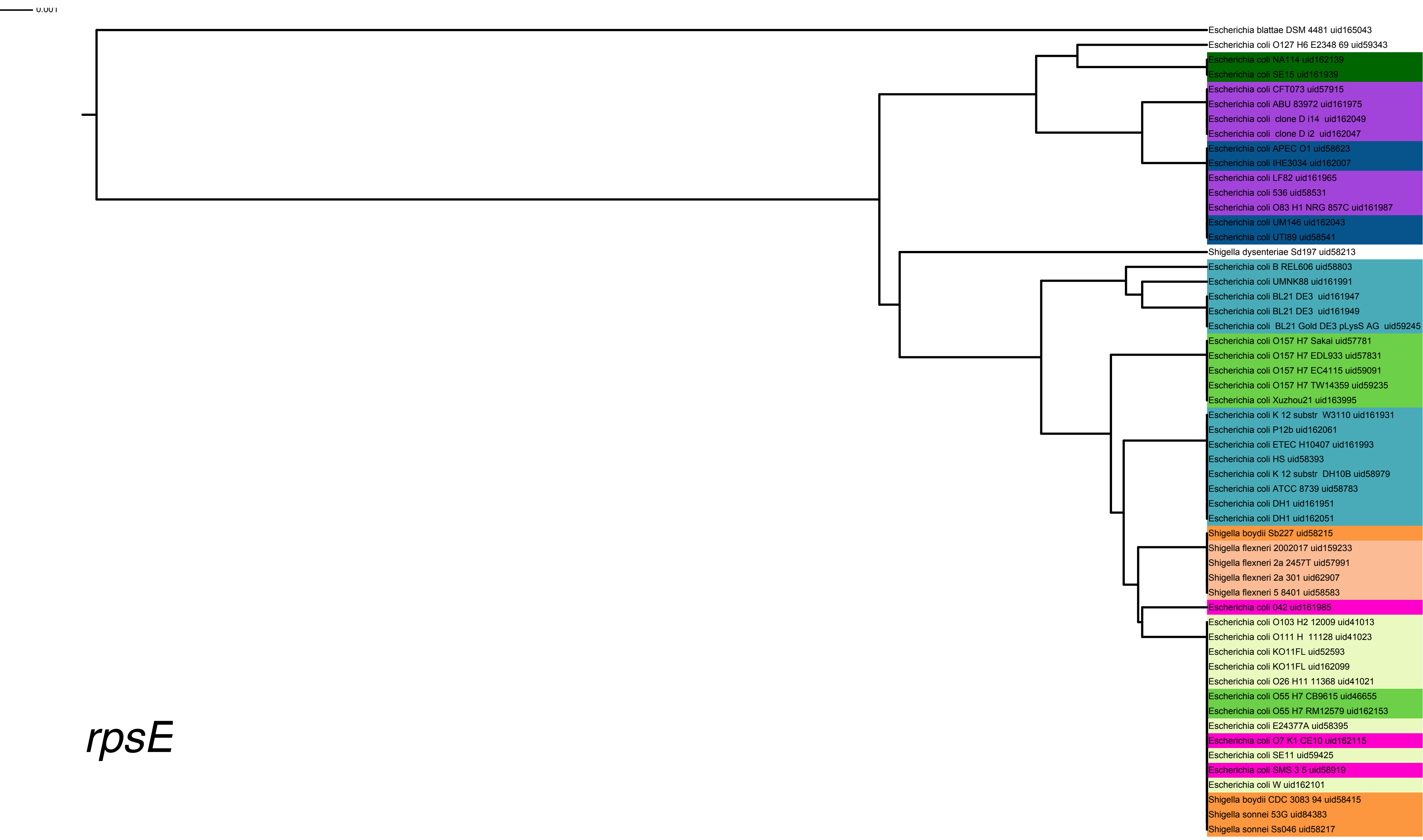
0.001
Escherichia blattae DSM 4481 uid165043
Escherichia coli O127 H6 E2348 69 uid59343
Escherichia coli NA114 uid162139
Escherichia coli SE15 uid161939
Escherichia coli CFT073 uid57915
Escherichia coli ABU 83972 uid161975
Escherichia coli clone D i14 uid162049
Escherichia coli clone D i2 uid162047
Escherichia coli APEC O1 uid58623
Escherichia coli IHE3034 uid162007
Escherichia coli LF82 uid161965
Escherichia coli 536 uid58531
Escherichia coli O83 H1 NRG 857C uid161987
Escherichia coli UM146 uid162043
Escherichia coli UTI89 uid58541
Shigella dysenteriae Sd197 uid58213
Escherichia coli B REL606 uid58803
Escherichia coli UMNK88 uid161991
Escherichia coli BL21 DE3 uid161947
Escherichia coli BL21 DE3 uid161949
Escherichia coli BL21 Gold DE3 pLysS AG uid59245
Escherichia coli O157 H7 Sakai uid57781
Escherichia coli O157 H7 EDL933 uid57831
Escherichia coli O157 H7 EC4115 uid59091
Escherichia coli O157 H7 TW14359 uid59235
Escherichia coli Xuzhou21 uid163995
Escherichia coli K 12 substr W3110 uid161931
Escherichia coli P12b uid162061
Escherichia coli ETEC H10407 uid161993
Escherichia coli HS uid58393
Escherichia coli K 12 substr DH10B uid58979
Escherichia coli ATCC 8739 uid58783
Escherichia coli DH1 uid161951
Escherichia coli DH1 uid162051
Shigella boydii Sb227 uid58215
Shigella flexneri 2002017 uid159233
Shigella flexneri 2a 2457T uid57991
Shigella flexneri 2a 301 uid62907
Shigella flexneri 5 8401 uid58583
Escherichia coli 042 uid161985
Escherichia coli O103 H2 12009 uid41013
Escherichia coli O111 H 11128 uid41023
Escherichia coli KO11FL uid52593
Escherichia coli KO11FL uid162099
Escherichia coli O26 H11 11368 uid41021
Escherichia coli O55 H7 CB9615 uid46655
Escherichia coli O55 H7 RM12579 uid162153
Escherichia coli E24377A uid58395
Escherichia coli O7 K1 CE10 uid162115
Escherichia coli SE11 uid59425
Escherichia coli SMS 3 5 uid58919
Escherichia coli W uid162101
Shigella boydii CDC 3083 94 uid58415
Shigella sonnei 53G uid84383
Shigella sonnei Ss046 uid58217
rpsE

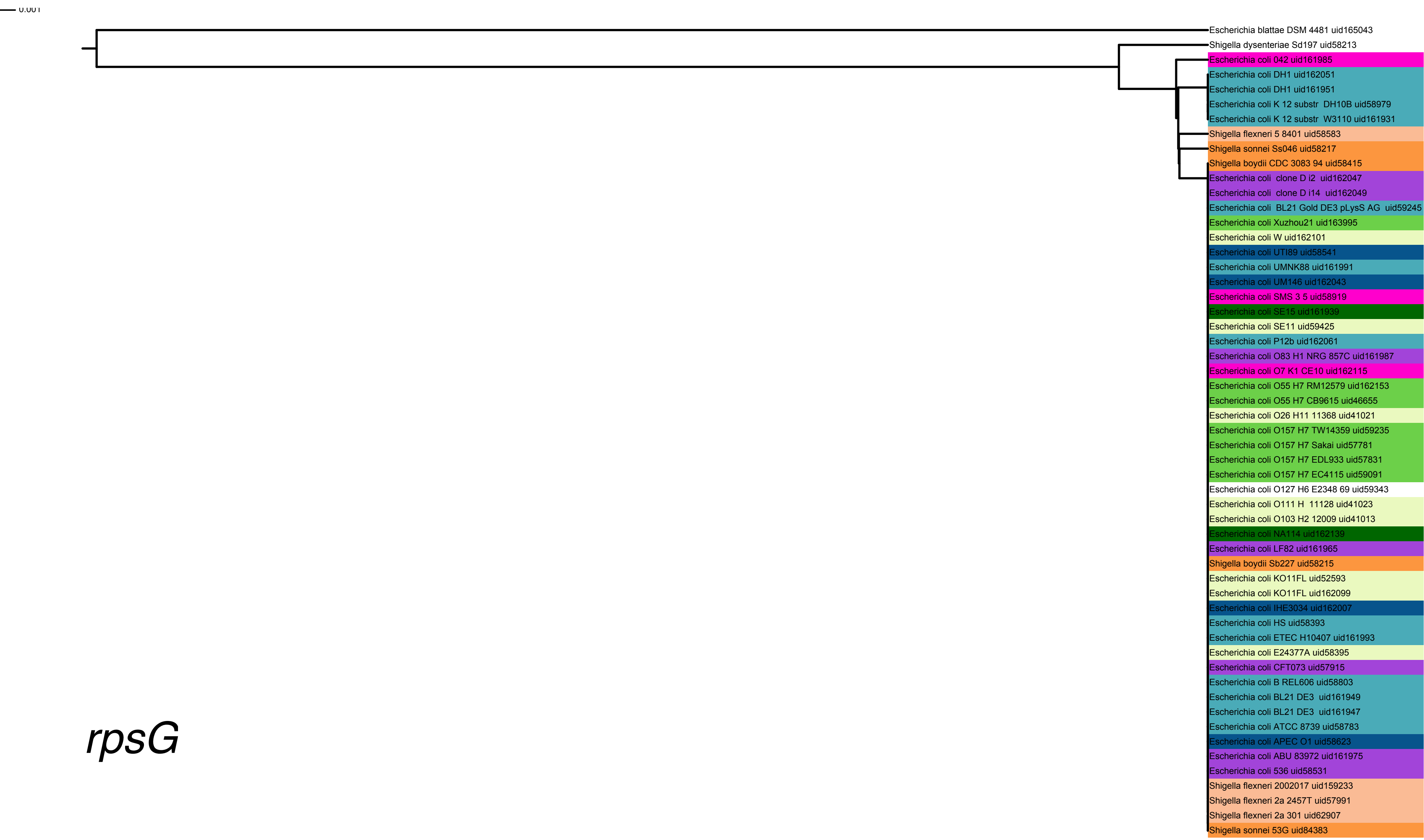
0.001
Escherichia blattae DSM 4481 uid165043
Shigella dysenteriae Sd197 uid58213
Escherichia coli 042 uid161985
Escherichia coli DH1 uid162051
Escherichia coli DH1 uid161951
Escherichia coli K 12 substr DH10B uid58979
Escherichia coli K 12 substr W3110 uid161931
Shigella flexneri 5 8401 uid58583
Shigella sonnei Ss046 uid58217
Shigella boydii CDC 3083 94 uid58415
Escherichia coli clone D i2 uid162047
Escherichia coli clone D i14 uid162049
Escherichia coli BL21 Gold DE3 pLysS AG uid59245
Escherichia coli Xuzhou21 uid163995
Escherichia coli W uid162101
Escherichia coli UTI89 uid58541
Escherichia coli UMNK88 uid161991
Escherichia coli UM146 uid162043
Escherichia coli SMS 3 5 uid58919
Escherichia coli SE15 uid161939
Escherichia coli SE11 uid59425
Escherichia coli P12b uid162061
Escherichia coli O83 H1 NRG 857C uid161987
Escherichia coli O7 K1 CE10 uid162115
Escherichia coli O55 H7 RM12579 uid162153
Escherichia coli O55 H7 CB9615 uid46655
Escherichia coli O26 H11 11368 uid41021
Escherichia coli O157 H7 TW14359 uid59235
Escherichia coli O157 H7 Sakai uid57781
Escherichia coli O157 H7 EDL933 uid57831
Escherichia coli O157 H7 EC4115 uid59091
Escherichia coli O127 H6 E2348 69 uid59343
Escherichia coli O111 H 11128 uid41023
Escherichia coli O103 H2 12009 uid41013
Escherichia coli NA114 uid162139
Escherichia coli LF82 uid161965
Shigella boydii Sb227 uid58215
Escherichia coli KO11FL uid52593
Escherichia coli KO11FL uid162099
Escherichia coli IHE3034 uid162007
Escherichia coli HS uid58393
Escherichia coli ETEC H10407 uid161993
Escherichia coli E24377A uid58395
Escherichia coli CFT073 uid57915
Escherichia coli B REL606 uid58803
Escherichia coli BL21 DE3 uid161949
Escherichia coli BL21 DE3 uid161947
Escherichia coli ATCC 8739 uid58783
Escherichia coli APEC O1 uid58623
Escherichia coli ABU 83972 uid161975
Escherichia coli 536 uid58531
Shigella flexneri 2002017 uid159233
Shigella flexneri 2a 2457T uid57991
Shigella flexneri 2a 301 uid62907
Shigella sonnei 53G uid84383
rpsG

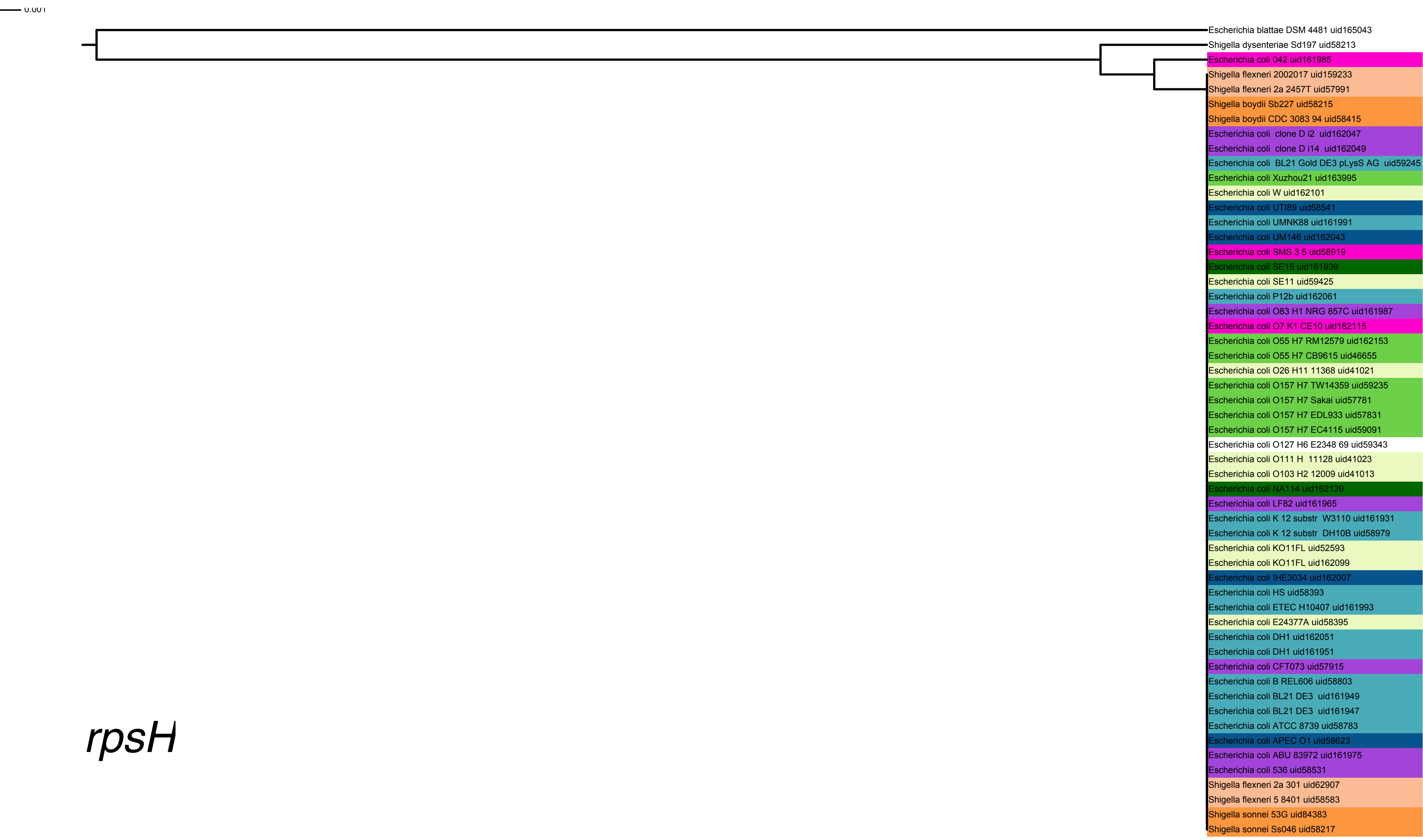
0.001
Escherichia blattae DSM 4481 uid165043
Shigella dysenteriae Sd197 uid58213
Escherichia coli 042 uid161985
Shigella flexneri 2002017 uid159233
Shigella flexneri 2a 2457T uid57991
Shigella boydii Sb227 uid58215
Shigella boydii CDC 3083 94 uid58415
Escherichia coli clone D i2 uid162047
Escherichia coli clone D i14 uid162049
Escherichia coli BL21 Gold DE3 pLysS AG uid59245
Escherichia coli Xuzhou21 uid163995
Escherichia coli W uid162101
Escherichia coli UTI89 uid58541
Escherichia coli UMNK88 uid161991
Escherichia coli UM146 uid162043
Escherichia coli SMS 3 5 uid58919
Escherichia coli SE15 uid161939
Escherichia coli SE11 uid59425
Escherichia coli P12b uid162061
Escherichia coli O83 H1 NRG 857C uid161987
Escherichia coli O7 K1 CE10 uid162115
Escherichia coli O55 H7 RM12579 uid162153
Escherichia coli O55 H7 CB9615 uid46655
Escherichia coli O26 H11 11368 uid41021
Escherichia coli O157 H7 TW14359 uid59235
Escherichia coli O157 H7 Sakai uid57781
Escherichia coli O157 H7 EDL933 uid57831
Escherichia coli O157 H7 EC4115 uid59091
Escherichia coli O127 H6 E2348 69 uid59343
Escherichia coli O111 H 11128 uid41023
Escherichia coli O103 H2 12009 uid41013
Escherichia coli NA114 uid162139
Escherichia coli LF82 uid161965
Escherichia coli K 12 substr W3110 uid161931
Escherichia coli K 12 substr DH10B uid58979
Escherichia coli KO11FL uid52593
Escherichia coli KO11FL uid162099
Escherichia coli IHE3034 uid162007
Escherichia coli HS uid58393
Escherichia coli ETEC H10407 uid161993
Escherichia coli E24377A uid58395
Escherichia coli DH1 uid162051
Escherichia coli DH1 uid161951
Escherichia coli CFT073 uid57915
Escherichia coli B REL606 uid58803
Escherichia coli BL21 DE3 uid161949
Escherichia coli BL21 DE3 uid161947
Escherichia coli ATCC 8739 uid58783
Escherichia coli APEC O1 uid58623
Escherichia coli ABU 83972 uid161975
Escherichia coli 536 uid58531
Shigella flexneri 2a 301 uid62907
Shigella flexneri 5 8401 uid58583
Shigella sonnei 53G uid84383
Shigella sonnei Ss046 uid58217
rpsH

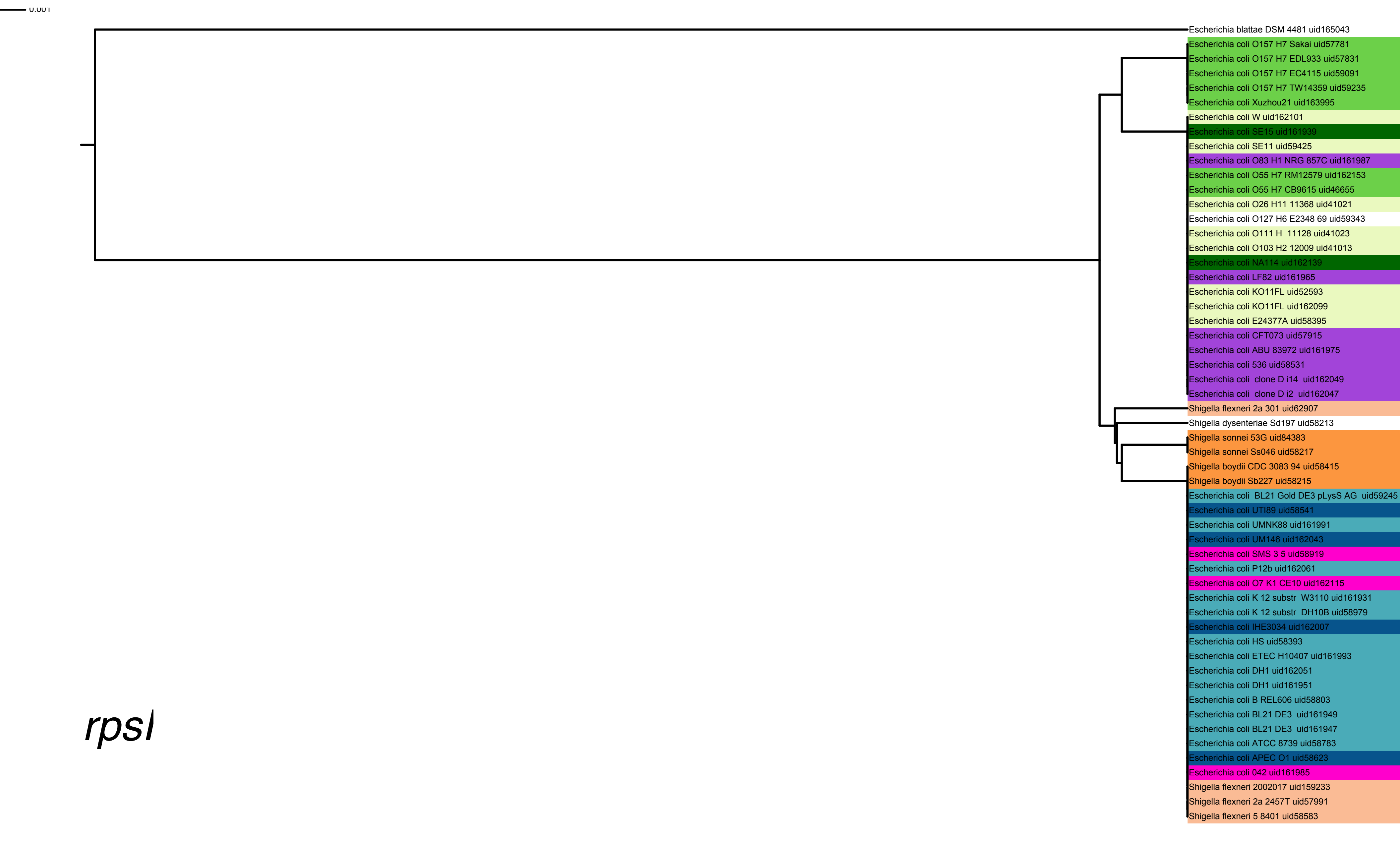
0.001
Escherichia blattae DSM 4481 uid165043
Escherichia coli O157 H7 Sakai uid57781
Escherichia coli O157 H7 EDL933 uid57831
Escherichia coli O157 H7 EC4115 uid59091
Escherichia coli O157 H7 TW14359 uid59235
Escherichia coli Xuzhou21 uid163995
Escherichia coli W uid162101
Escherichia coli SE15 uid161939
Escherichia coli SE11 uid59425
Escherichia coli O83 H1 NRG 857C uid161987
Escherichia coli O55 H7 RM12579 uid162153
Escherichia coli O55 H7 CB9615 uid46655
Escherichia coli O26 H11 11368 uid41021
Escherichia coli O127 H6 E2348 69 uid59343
Escherichia coli O111 H 11128 uid41023
Escherichia coli O103 H2 12009 uid41013
Escherichia coli NA114 uid162139
Escherichia coli LF82 uid161965
Escherichia coli KO11FL uid52593
Escherichia coli KO11FL uid162099
Escherichia coli E24377A uid58395
Escherichia coli CFT073 uid57915
Escherichia coli ABU 83972 uid161975
Escherichia coli 536 uid58531
Escherichia coli clone D i14 uid162049
Escherichia coli clone D i2 uid162047
Shigella flexneri 2a 301 uid62907
Shigella dysenteriae Sd197 uid58213
Shigella sonnei 53G uid84383
Shigella sonnei Ss046 uid58217
Shigella boydii CDC 3083 94 uid58415
Shigella boydii Sb227 uid58215
Escherichia coli BL21 Gold DE3 pLysS AG uid59245
Escherichia coli UTI89 uid58541
Escherichia coli UMNK88 uid161991
Escherichia coli UM146 uid162043
Escherichia coli SMS 3 5 uid58919
Escherichia coli P12b uid162061
Escherichia coli O7 K1 CE10 uid162115
Escherichia coli K 12 substr W3110 uid161931
Escherichia coli K 12 substr DH10B uid58979
Escherichia coli IHE3034 uid162007
Escherichia coli HS uid58393
Escherichia coli ETEC H10407 uid161993
Escherichia coli DH1 uid162051
Escherichia coli DH1 uid161951
Escherichia coli B REL606 uid58803
Escherichia coli BL21 DE3 uid161949
Escherichia coli BL21 DE3 uid161947
Escherichia coli ATCC 8739 uid58783
Escherichia coli APEC O1 uid58623
Escherichia coli 042 uid161985
Shigella flexneri 2002017 uid159233
Shigella flexneri 2a 2457T uid57991
Shigella flexneri 5 8401 uid58583
rpsl

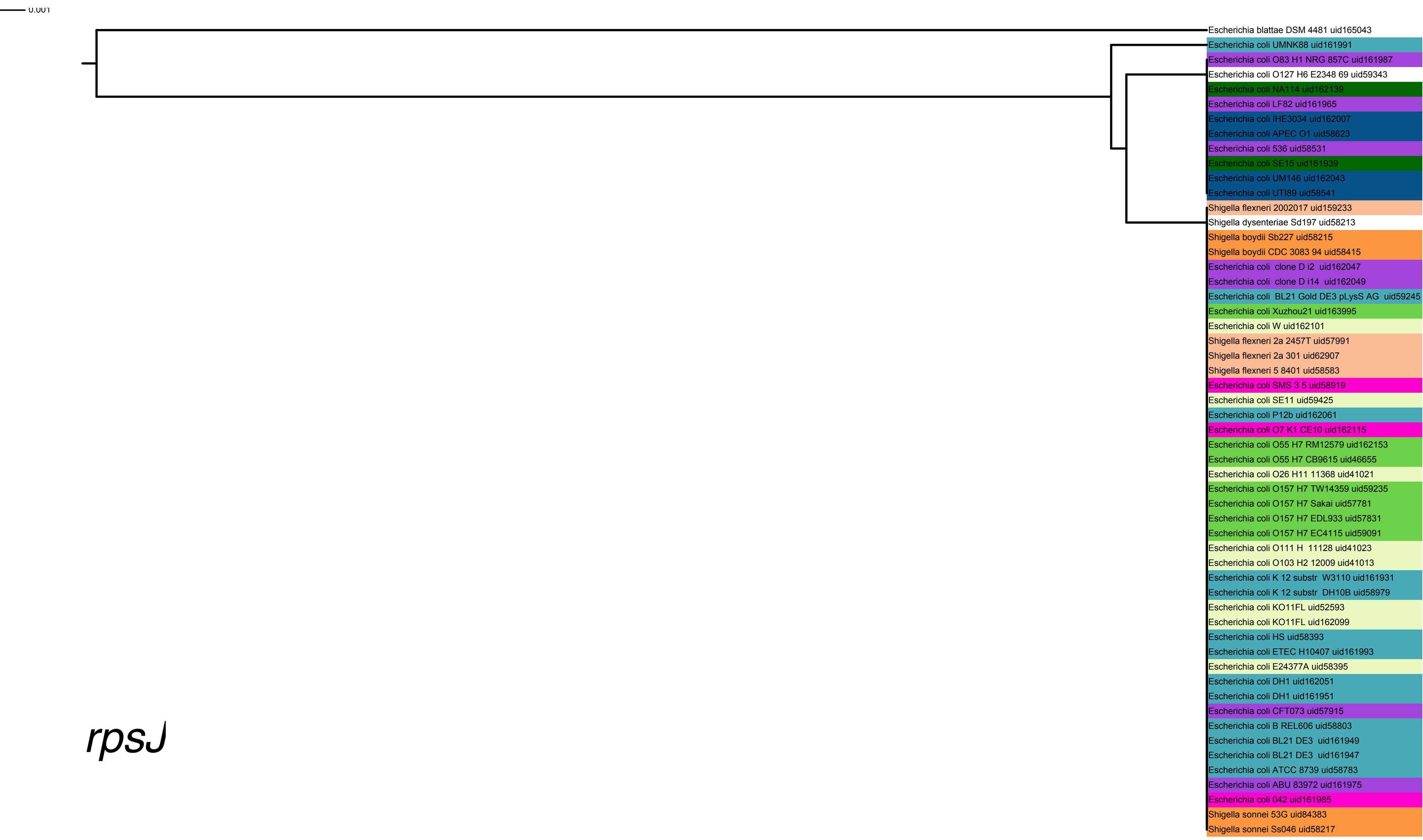
0.001
Escherichia blattae DSM 4481 uid165043
Escherichia coli UMNK88 uid161991
Escherichia coli O83 H1 NRG 857C uid161987
Escherichia coli O127 H6 E2348 69 uid59343
Escherichia coli NA114 uid162139
Escherichia coli LF82 uid161965
Escherichia coli IHE3034 uid162007
Escherichia coli APEC O1 uid58623
Escherichia coli 536 uid58531
Escherichia coli SE15 uid161939
Escherichia coli UM146 uid162043
Escherichia coli UTI89 uid58541
Shigella flexneri 2002017 uid159233
Shigella dysenteriae Sd197 uid58213
Shigella boydii Sb227 uid58215
Shigella boydii CDC 3083 94 uid58415
Escherichia coli clone D i2 uid162047
Escherichia coli clone D i14 uid162049
Escherichia coli BL21 Gold DE3 pLysS AG uid59245
Escherichia coli Xuzhou21 uid163995
Escherichia coli W uid162101
Shigella flexneri 2a 2457T uid57991
Shigella flexneri 2a 301 uid62907
Shigella flexneri 5 8401 uid58583
Escherichia coli SMS 3 5 uid58919
Escherichia coli SE11 uid59425
Escherichia coli P12b uid162061
Escherichia coli O7 K1 CE10 uid162115
Escherichia coli O55 H7 RM12579 uid162153
Escherichia coli O55 H7 CB9615 uid46655
Escherichia coli O26 H11 11368 uid41021
Escherichia coli O157 H7 TW14359 uid59235
Escherichia coli O157 H7 Sakai uid57781
Escherichia coli O157 H7 EDL933 uid57831
Escherichia coli O157 H7 EC4115 uid59091
Escherichia coli O111 H 11128 uid41023
Escherichia coli O103 H2 12009 uid41013
Escherichia coli K 12 substr W3110 uid161931
Escherichia coli K 12 substr DH10B uid58979
Escherichia coli KO11FL uid52593
Escherichia coli KO11FL uid162099
Escherichia coli HS uid58393
Escherichia coli ETEC H10407 uid161993
Escherichia coli E24377A uid58395
Escherichia coli DH1 uid162051
Escherichia coli DH1 uid161951
Escherichia coli CFT073 uid57915
Escherichia coli B REL606 uid58803
Escherichia coli BL21 DE3 uid161949
Escherichia coli BL21 DE3 uid161947
Escherichia coli ATCC 8739 uid58783
Escherichia coli ABU 83972 uid161975
Escherichia coli 042 uid161985
Shigella sonnei 53G uid84383
Shigella sonnei Ss046 uid58217
rpsJ

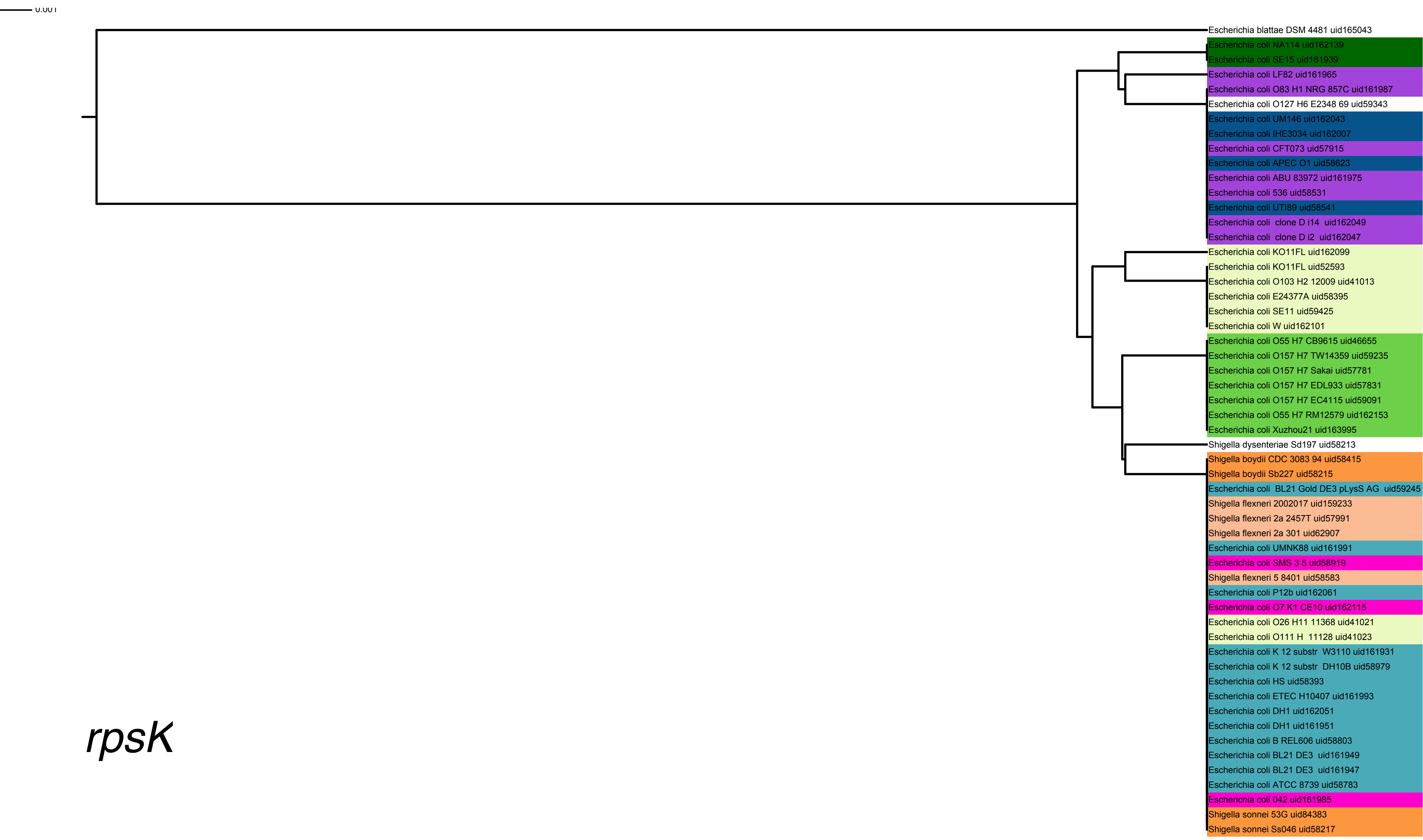
0.001
Escherichia blattae DSM 4481 uid165043
Escherichia coli NA114 uid162139
Escherichia coli SE15 uid161939
Escherichia coli LF82 uid161965
Escherichia coli O83 H1 NRG 857C uid161987
Escherichia coli O127 H6 E2348 69 uid59343
Escherichia coli UM146 uid162043
Escherichia coli IHE3034 uid162007
Escherichia coli CFT073 uid57915
Escherichia coli APEC O1 uid58623
Escherichia coli ABU 83972 uid161975
Escherichia coli 536 uid58531
Escherichia coli UTI89 uid58541
Escherichia coli clone D i14 uid162049
Escherichia coli clone D i2 uid162047
Escherichia coli KO11FL uid162099
Escherichia coli KO11FL uid52593
Escherichia coli O103 H2 12009 uid41013
Escherichia coli E24377A uid58395
Escherichia coli SE11 uid59425
Escherichia coli W uid162101
Escherichia coli O55 H7 CB9615 uid46655
Escherichia coli O157 H7 TW14359 uid59235
Escherichia coli O157 H7 Sakai uid57781
Escherichia coli O157 H7 EDL933 uid57831
Escherichia coli O157 H7 EC4115 uid59091
Escherichia coli O55 H7 RM12579 uid162153
Escherichia coli Xuzhou21 uid163995
Shigella dysenteriae Sd197 uid58213
Shigella boydii CDC 3083 94 uid58415
Shigella boydii Sb227 uid58215
Escherichia coli BL21 Gold DE3 pLysS AG uid59245
Shigella flexneri 2002017 uid159233
Shigella flexneri 2a 2457T uid57991
Shigella flexneri 2a 301 uid62907
Escherichia coli UMNK88 uid161991
Escherichia coli SMS 3 5 uid58919
Shigella flexneri 5 8401 uid58583
Escherichia coli P12b uid162061
Escherichia coli O7 K1 CE10 uid162115
Escherichia coli O26 H11 11368 uid41021
Escherichia coli O111 H 11128 uid41023
Escherichia coli K 12 substr W3110 uid161931
Escherichia coli K 12 substr DH10B uid58979
Escherichia coli HS uid58393
Escherichia coli ETEC H10407 uid161993
Escherichia coli DH1 uid162051
Escherichia coli DH1 uid161951
Escherichia coli B REL606 uid58803
Escherichia coli BL21 DE3 uid161949
Escherichia coli BL21 DE3 uid161947
Escherichia coli ATCC 8739 uid58783
Escherichia coli 042 uid161985
Shigella sonnei 53G uid84383
Shigella sonnei Ss046 uid58217
rpsK

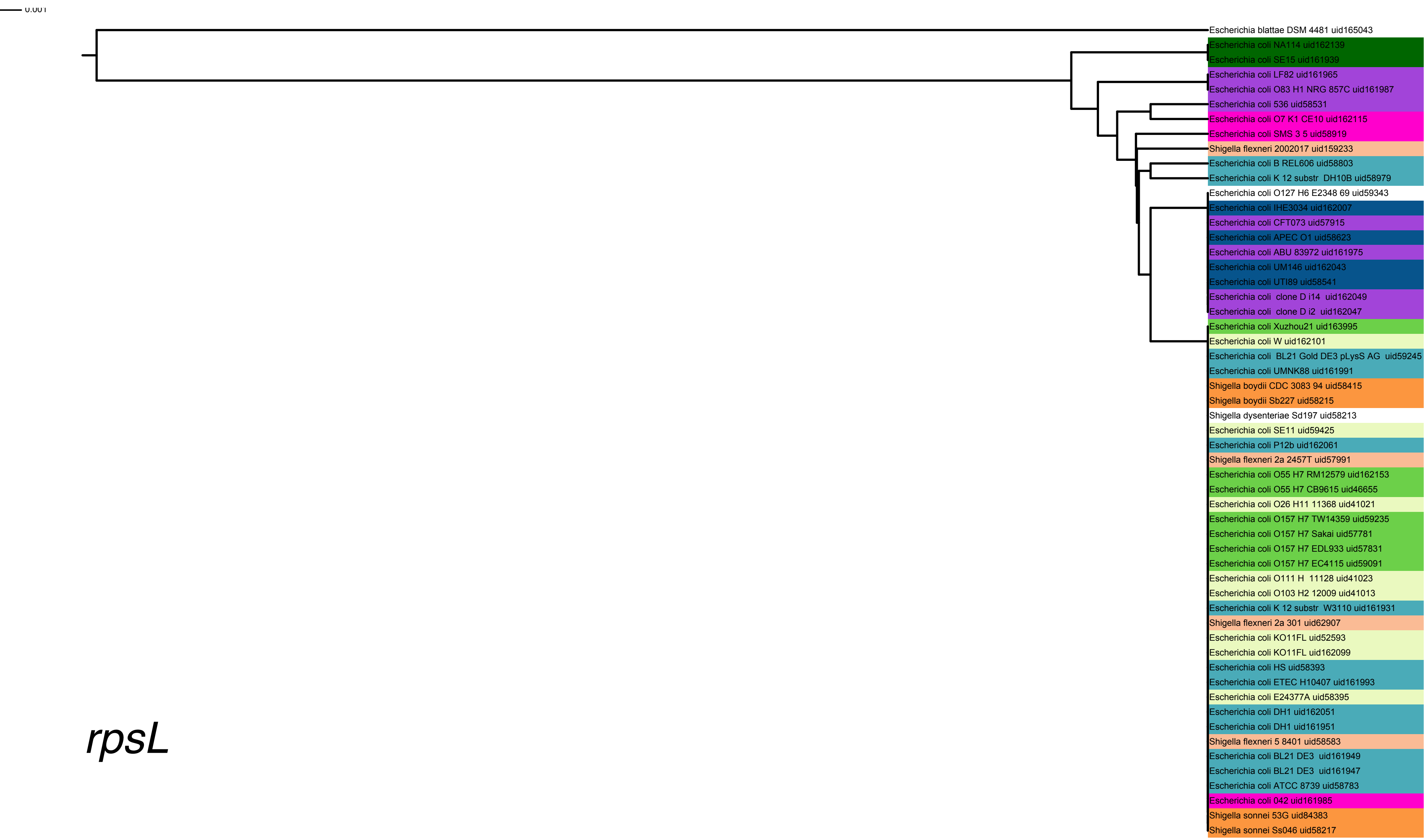
0.001
Escherichia blattae DSM 4481 uid165043
Escherichia coli NA114 uid162139
Escherichia coli SE15 uid161939
Escherichia coli LF82 uid161965
Escherichia coli O83 H1 NRG 857C uid161987
Escherichia coli 536 uid58531
Escherichia coli O7 K1 CE10 uid162115
Escherichia coli SMS 3 5 uid58919
Shigella flexneri 2002017 uid159233
Escherichia coli B REL606 uid58803
Escherichia coli K 12 substr DH10B uid58979
Escherichia coli O127 H6 E2348 69 uid59343
Escherichia coli IHE3034 uid162007
Escherichia coli CFT073 uid57915
Escherichia coli APEC O1 uid58623
Escherichia coli ABU 83972 uid161975
Escherichia coli UM146 uid162043
Escherichia coli UTI89 uid58541
Escherichia coli clone D i14 uid162049
Escherichia coli clone D i2 uid162047
Escherichia coli Xuzhou21 uid163995
Escherichia coli W uid162101
Escherichia coli BL21 Gold DE3 pLysS AG uid59245
Escherichia coli UMNK88 uid161991
Shigella boydii CDC 3083 94 uid58415
Shigella boydii Sb227 uid58215
Shigella dysenteriae Sd197 uid58213
Escherichia coli SE11 uid59425
Escherichia coli P12b uid162061
Shigella flexneri 2a 2457T uid57991
Escherichia coli O55 H7 RM12579 uid162153
Escherichia coli O55 H7 CB9615 uid46655
Escherichia coli O26 H11 11368 uid41021
Escherichia coli O157 H7 TW14359 uid59235
Escherichia coli O157 H7 Sakai uid57781
Escherichia coli O157 H7 EDL933 uid57831
Escherichia coli O157 H7 EC4115 uid59091
Escherichia coli O111 H 11128 uid41023
Escherichia coli O103 H2 12009 uid41013
Escherichia coli K 12 substr W3110 uid161931
Shigella flexneri 2a 301 uid62907
Escherichia coli KO11FL uid52593
Escherichia coli KO11FL uid162099
Escherichia coli HS uid58393
Escherichia coli ETEC H10407 uid161993
Escherichia coli E24377A uid58395
Escherichia coli DH1 uid162051
Escherichia coli DH1 uid161951
Shigella flexneri 5 8401 uid58583
Escherichia coli BL21 DE3 uid161949
Escherichia coli BL21 DE3 uid161947
Escherichia coli ATCC 8739 uid58783
Escherichia coli 042 uid161985
Shigella sonnei 53G uid84383
Shigella sonnei Ss046 uid58217

rpsL

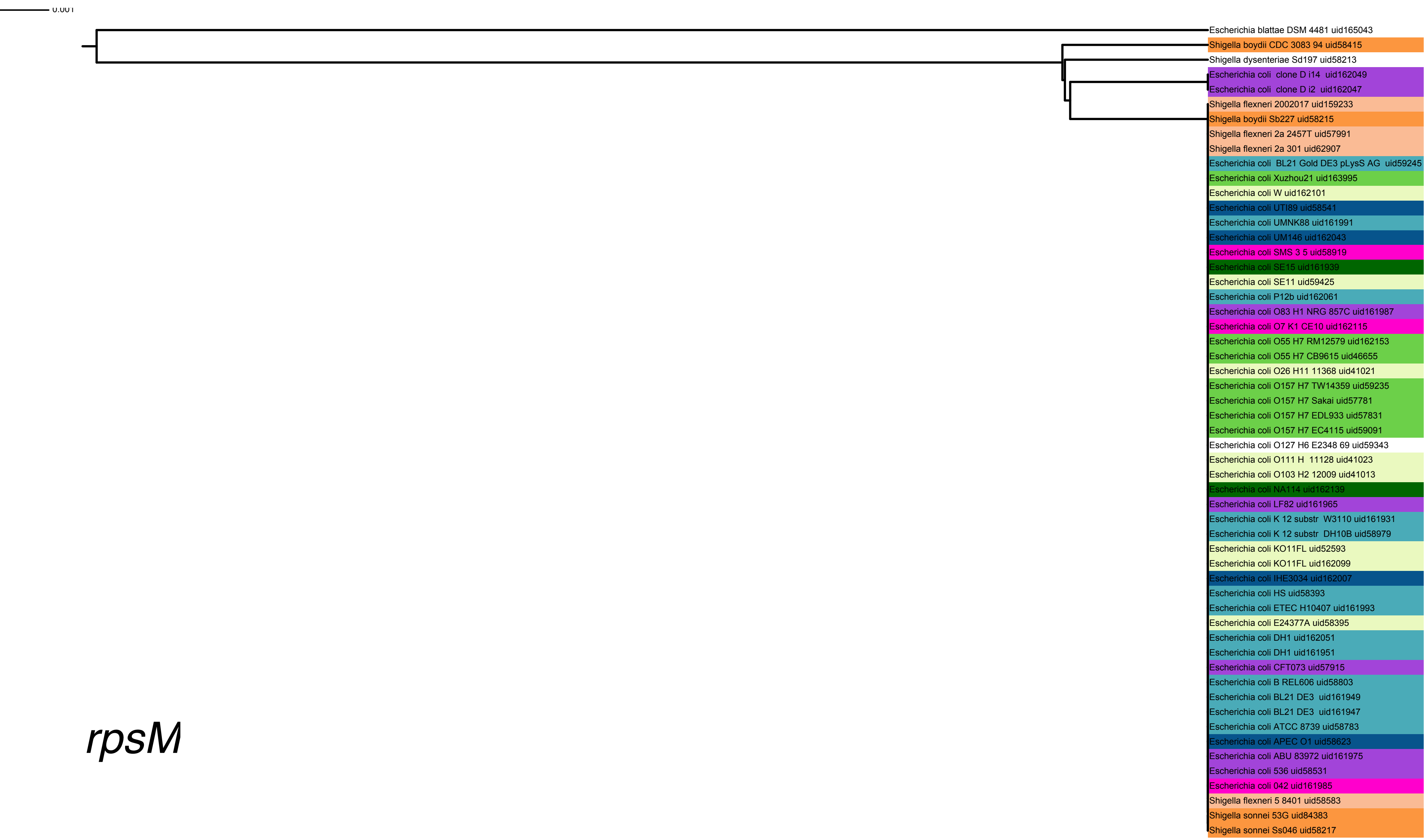
0.001
Escherichia blattae DSM 4481 uid165043
Shigella boydii CDC 3083 94 uid58415
Shigella dysenteriae Sd197 uid58213
Escherichia coli clone D i14 uid162049
Escherichia coli clone D i2 uid162047
Shigella flexneri 2002017 uid159233
Shigella boydii Sb227 uid58215
Shigella flexneri 2a 2457T uid57991
Shigella flexneri 2a 301 uid62907
Escherichia coli BL21 Gold DE3 pLysS AG uid59245
Escherichia coli Xuzhou21 uid163995
Escherichia coli W uid162101
Escherichia coli UTI89 uid58541
Escherichia coli UMNK88 uid161991
Escherichia coli UM146 uid162043
Escherichia coli SMS 3 5 uid58919
Escherichia coli SE15 uid161939
Escherichia coli SE11 uid59425
Escherichia coli P12b uid162061
Escherichia coli O83 H1 NRG 857C uid161987
Escherichia coli O7 K1 CE10 uid162115
Escherichia coli O55 H7 RM12579 uid162153
Escherichia coli O55 H7 CB9615 uid46655
Escherichia coli O26 H11 11368 uid41021
Escherichia coli O157 H7 TW14359 uid59235
Escherichia coli O157 H7 Sakai uid57781
Escherichia coli O157 H7 EDL933 uid57831
Escherichia coli O157 H7 EC4115 uid59091
Escherichia coli O127 H6 E2348 69 uid59343
Escherichia coli O111 H 11128 uid41023
Escherichia coli O103 H2 12009 uid41013
Escherichia coli NA114 uid162139
Escherichia coli LF82 uid161965
Escherichia coli K 12 substr W3110 uid161931
Escherichia coli K 12 substr DH10B uid58979
Escherichia coli KO11FL uid52593
Escherichia coli KO11FL uid162099
Escherichia coli IHE3034 uid162007
Escherichia coli HS uid58393
Escherichia coli ETEC H10407 uid161993
Escherichia coli E24377A uid58395
Escherichia coli DH1 uid162051
Escherichia coli DH1 uid161951
Escherichia coli CFT073 uid57915
Escherichia coli B REL606 uid58803
Escherichia coli BL21 DE3 uid161949
Escherichia coli BL21 DE3 uid161947
Escherichia coli ATCC 8739 uid58783
Escherichia coli APEC O1 uid58623
Escherichia coli ABU 83972 uid161975
Escherichia coli 536 uid58531
Escherichia coli 042 uid161985
Shigella flexneri 5 8401 uid58583
Shigella sonnei 53G uid84383
Shigella sonnei Ss046 uid58217
rpsM

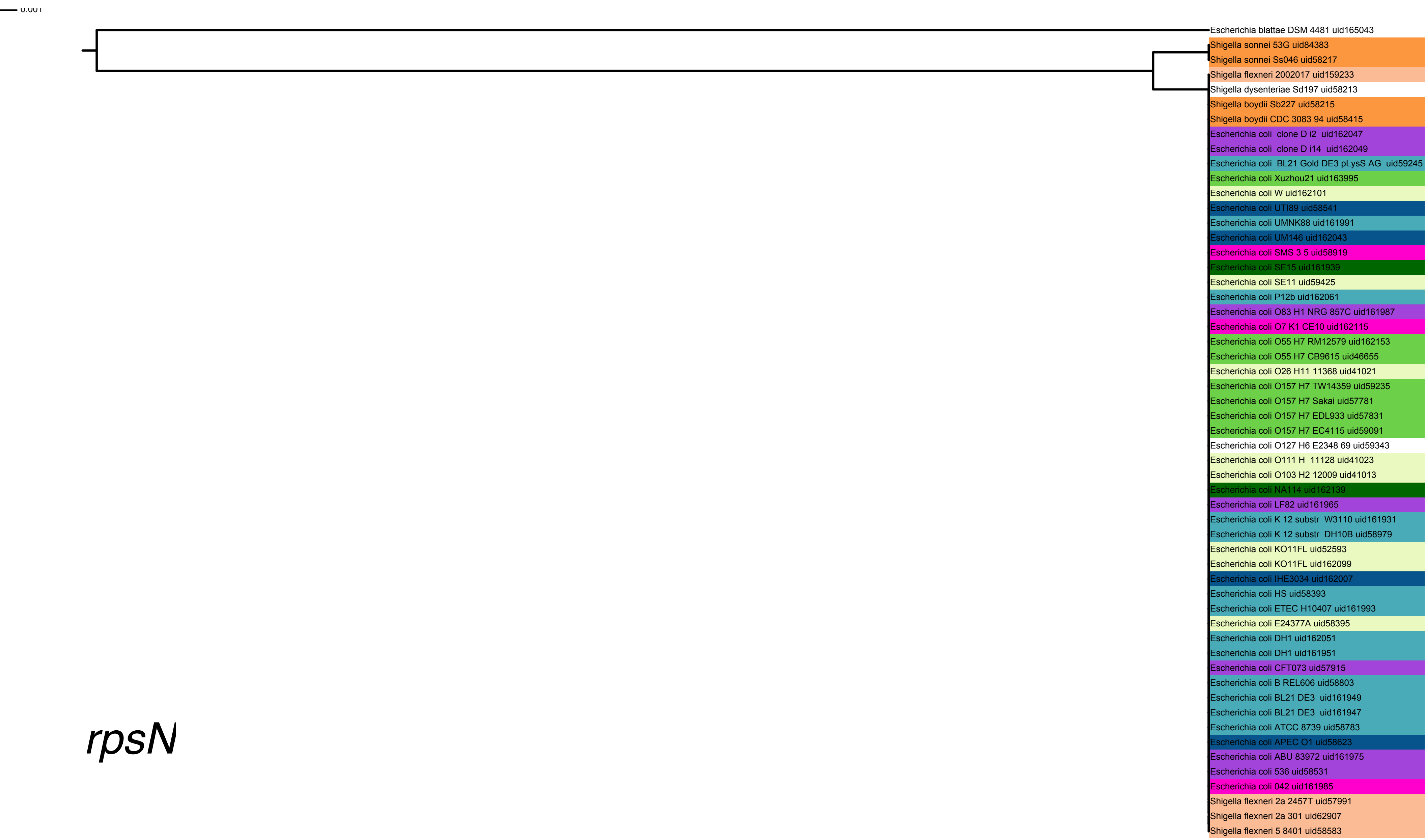
0.001
Escherichia blattae DSM 4481 uid165043
Shigella sonnei 53G uid84383
Shigella sonnei Ss046 uid58217
Shigella flexneri 2002017 uid159233
Shigella dysenteriae Sd197 uid58213
Shigella boydii Sb227 uid58215
Shigella boydii CDC 3083 94 uid58415
Escherichia coli clone D i2 uid162047
Escherichia coli clone D i14 uid162049
Escherichia coli BL21 Gold DE3 pLysS AG uid59245
Escherichia coli Xuzhou21 uid163995
Escherichia coli W uid162101
Escherichia coli UTI89 uid58541
Escherichia coli UMNK88 uid161991
Escherichia coli UM146 uid162043
Escherichia coli SMS 3 5 uid58919
Escherichia coli SE15 uid161939
Escherichia coli SE11 uid59425
Escherichia coli P12b uid162061
Escherichia coli O83 H1 NRG 857C uid161987
Escherichia coli O7 K1 CE10 uid162115
Escherichia coli O55 H7 RM12579 uid162153
Escherichia coli O55 H7 CB9615 uid46655
Escherichia coli O26 H11 11368 uid41021
Escherichia coli O157 H7 TW14359 uid59235
Escherichia coli O157 H7 Sakai uid57781
Escherichia coli O157 H7 EDL933 uid57831
Escherichia coli O157 H7 EC4115 uid59091
Escherichia coli O127 H6 E2348 69 uid59343
Escherichia coli O111 H 11128 uid41023
Escherichia coli O103 H2 12009 uid41013
Escherichia coli NA114 uid162139
Escherichia coli LF82 uid161965
Escherichia coli K 12 substr W3110 uid161931
Escherichia coli K 12 substr DH10B uid58979
Escherichia coli KO11FL uid52593
Escherichia coli KO11FL uid162099
Escherichia coli IHE3034 uid162007
Escherichia coli HS uid58393
Escherichia coli ETEC H10407 uid161993
Escherichia coli E24377A uid58395
Escherichia coli DH1 uid162051
Escherichia coli DH1 uid161951
Escherichia coli CFT073 uid57915
Escherichia coli B REL606 uid58803
Escherichia coli BL21 DE3 uid161949
Escherichia coli BL21 DE3 uid161947
Escherichia coli ATCC 8739 uid58783
Escherichia coli APEC O1 uid58623
Escherichia coli ABU 83972 uid161975
Escherichia coli 536 uid58531
Escherichia coli 042 uid161985
Shigella flexneri 2a 2457T uid57991
Shigella flexneri 2a 301 uid62907
Shigella flexneri 5 8401 uid58583
rpsN

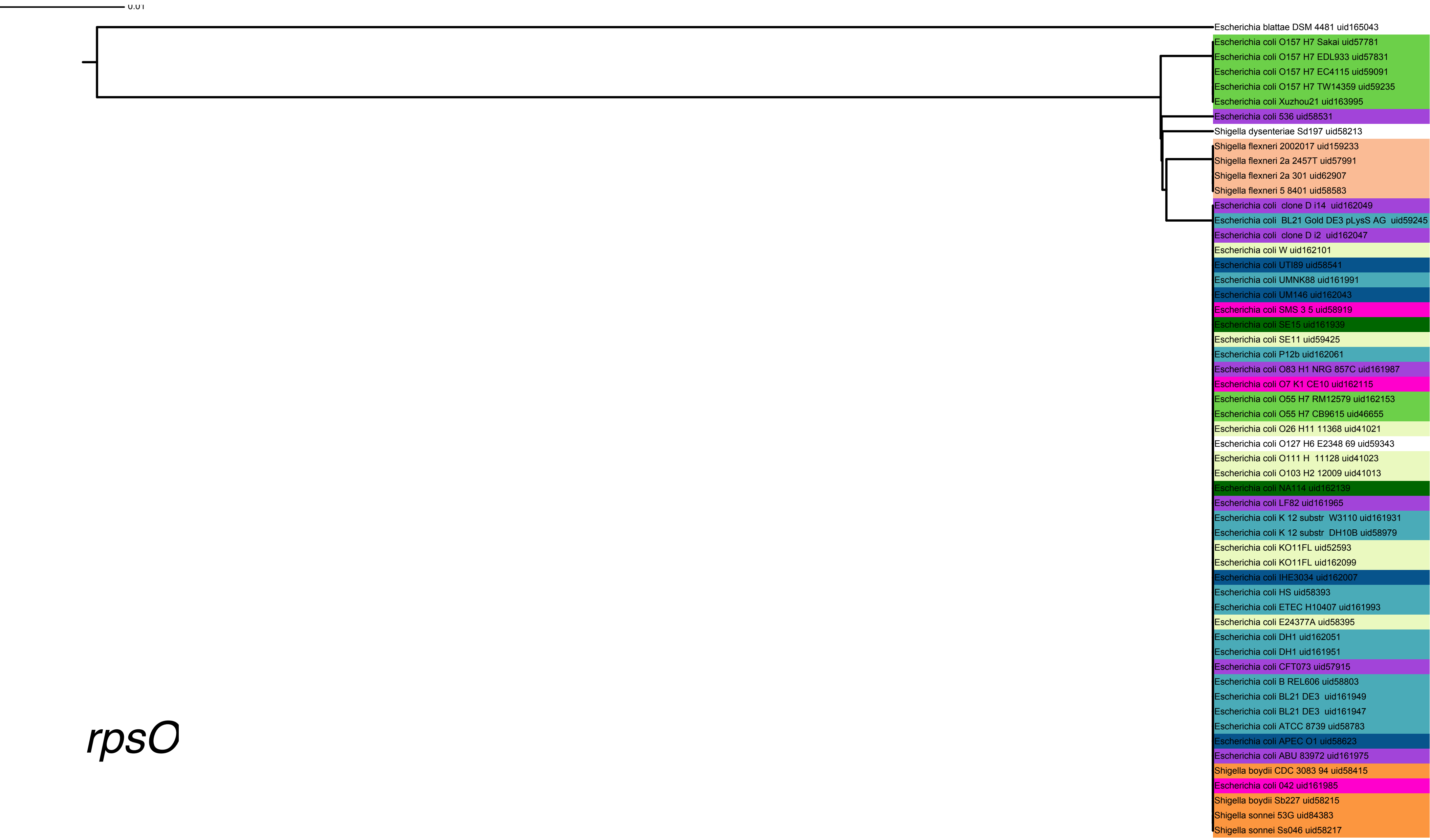
0.01
Escherichia blattae DSM 4481 uid165043
Escherichia coli O157 H7 Sakai uid57781
Escherichia coli O157 H7 EDL933 uid57831
Escherichia coli O157 H7 EC4115 uid59091
Escherichia coli O157 H7 TW14359 uid59235
Escherichia coli Xuzhou21 uid163995
Escherichia coli 536 uid58531
Shigella dysenteriae Sd197 uid58213
Shigella flexneri 2002017 uid159233
Shigella flexneri 2a 2457T uid57991
Shigella flexneri 2a 301 uid62907
Shigella flexneri 5 8401 uid58583
Escherichia coli clone D i14 uid162049
Escherichia coli BL21 Gold DE3 pLysS AG uid59245
Escherichia coli clone D i2 uid162047
Escherichia coli W uid162101
Escherichia coli UTI89 uid58541
Escherichia coli UMNK88 uid161991
Escherichia coli UM146 uid162043
Escherichia coli SMS 3 5 uid58919
Escherichia coli SE15 uid161939
Escherichia coli SE11 uid59425
Escherichia coli P12b uid162061
Escherichia coli O83 H1 NRG 857C uid161987
Escherichia coli O7 K1 CE10 uid162115
Escherichia coli O55 H7 RM12579 uid162153
Escherichia coli O55 H7 CB9615 uid46655
Escherichia coli O26 H11 11368 uid41021
Escherichia coli O127 H6 E2348 69 uid59343
Escherichia coli O111 H 11128 uid41023
Escherichia coli O103 H2 12009 uid41013
Escherichia coli NA114 uid162139
Escherichia coli LF82 uid161965
Escherichia coli K 12 substr W3110 uid161931
Escherichia coli K 12 substr DH10B uid58979
Escherichia coli KO11FL uid52593
Escherichia coli KO11FL uid162099
Escherichia coli IHE3034 uid162007
Escherichia coli HS uid58393
Escherichia coli ETEC H10407 uid161993
Escherichia coli E24377A uid58395
Escherichia coli DH1 uid162051
Escherichia coli DH1 uid161951
Escherichia coli CFT073 uid57915
Escherichia coli B REL606 uid58803
Escherichia coli BL21 DE3 uid161949
Escherichia coli BL21 DE3 uid161947
Escherichia coli ATCC 8739 uid58783
Escherichia coli APEC O1 uid58623
Escherichia coli ABU 83972 uid161975
Shigella boydii CDC 3083 94 uid58415
Escherichia coli 042 uid161985
Shigella boydii Sb227 uid58215
Shigella sonnei 53G uid84383
Shigella sonnei Ss046 uid58217
rpsO

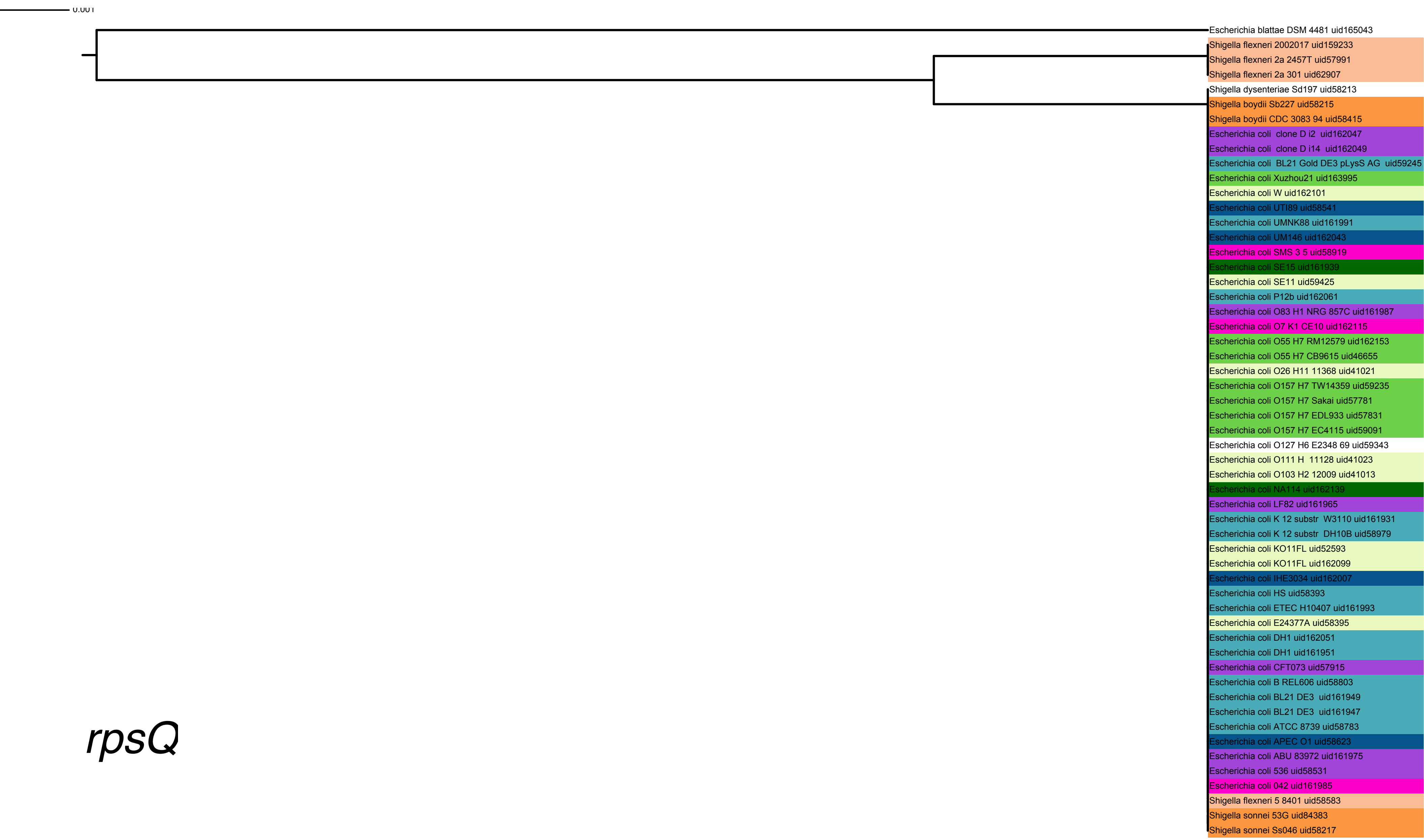
0.001
Escherichia blattae DSM 4481 uid165043
Shigella flexneri 2002017 uid159233
Shigella flexneri 2a 2457T uid57991
Shigella flexneri 2a 301 uid62907
Shigella dysenteriae Sd197 uid58213
Shigella boydii Sb227 uid58215
Shigella boydii CDC 3083 94 uid58415
Escherichia coli clone D i2 uid162047
Escherichia coli clone D i14 uid162049
Escherichia coli BL21 Gold DE3 pLysS AG uid59245
Escherichia coli Xuzhou21 uid163995
Escherichia coli W uid162101
Escherichia coli UTI89 uid58541
Escherichia coli UMNK88 uid161991
Escherichia coli UM146 uid162043
Escherichia coli SMS 3 5 uid58919
Escherichia coli SE15 uid161939
Escherichia coli SE11 uid59425
Escherichia coli P12b uid162061
Escherichia coli O83 H1 NRG 857C uid161987
Escherichia coli O7 K1 CE10 uid162115
Escherichia coli O55 H7 RM12579 uid162153
Escherichia coli O55 H7 CB9615 uid46655
Escherichia coli O26 H11 11368 uid41021
Escherichia coli O157 H7 TW14359 uid59235
Escherichia coli O157 H7 Sakai uid57781
Escherichia coli O157 H7 EDL933 uid57831
Escherichia coli O157 H7 EC4115 uid59091
Escherichia coli O127 H6 E2348 69 uid59343
Escherichia coli O111 H 11128 uid41023
Escherichia coli O103 H2 12009 uid41013
Escherichia coli NA114 uid162139
Escherichia coli LF82 uid161965
Escherichia coli K 12 substr W3110 uid161931
Escherichia coli K 12 substr DH10B uid58979
Escherichia coli KO11FL uid52593
Escherichia coli KO11FL uid162099
Escherichia coli IHE3034 uid162007
Escherichia coli HS uid58393
Escherichia coli ETEC H10407 uid161993
Escherichia coli E24377A uid58395
Escherichia coli DH1 uid162051
Escherichia coli DH1 uid161951
Escherichia coli CFT073 uid57915
Escherichia coli B REL606 uid58803
Escherichia coli BL21 DE3 uid161949
Escherichia coli BL21 DE3 uid161947
Escherichia coli ATCC 8739 uid58783
Escherichia coli APEC O1 uid58623
Escherichia coli ABU 83972 uid161975
Escherichia coli 536 uid58531
Escherichia coli 042 uid161985
Shigella flexneri 5 8401 uid58583
Shigella sonnei 53G uid84383
Shigella sonnei Ss046 uid58217

rpsQ

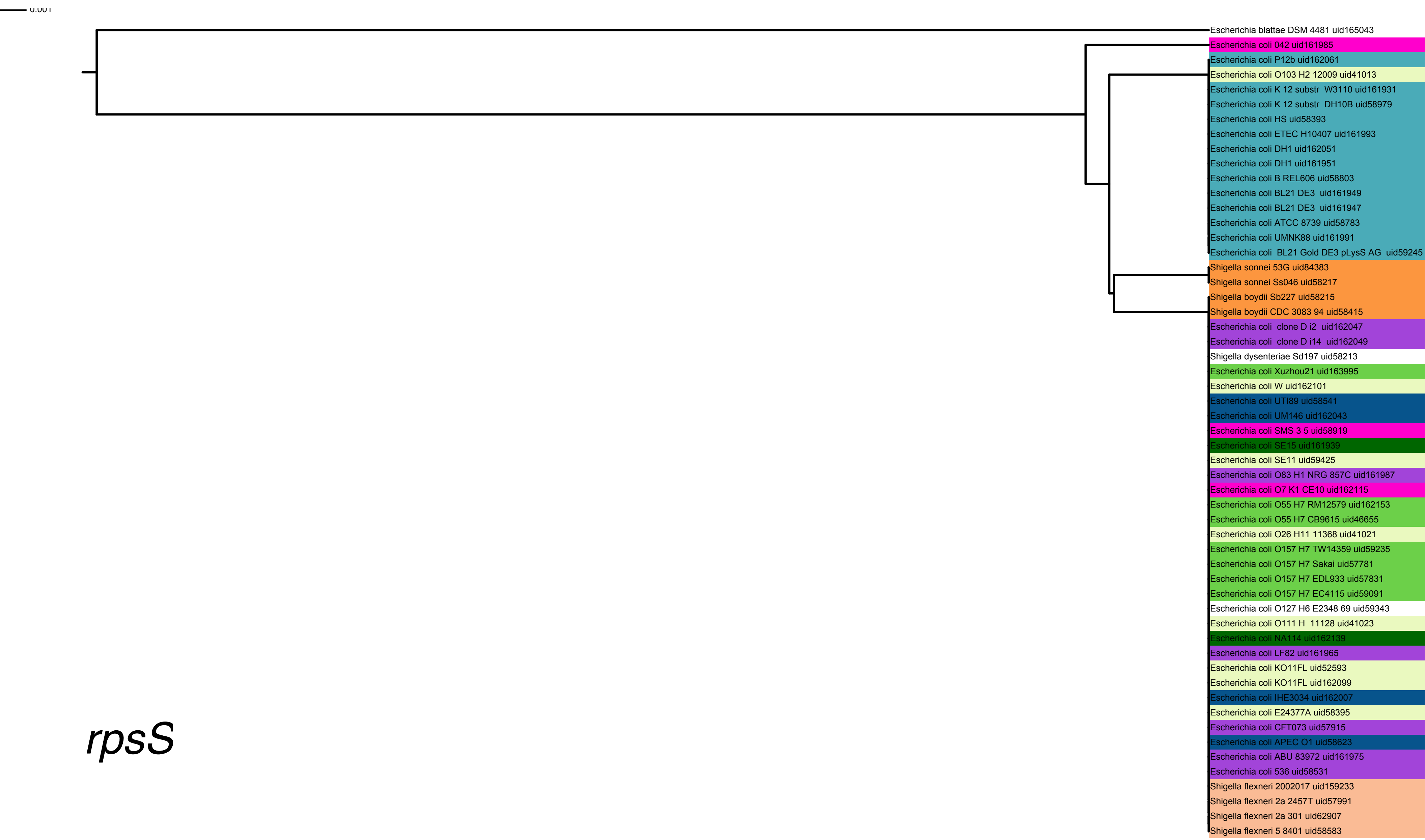
0.001
Escherichia blattae DSM 4481 uid165043
Escherichia coli 042 uid161985
Escherichia coli P12b uid162061
Escherichia coli O103 H2 12009 uid41013
Escherichia coli K 12 substr W3110 uid161931
Escherichia coli K 12 substr DH10B uid58979
Escherichia coli HS uid58393
Escherichia coli ETEC H10407 uid161993
Escherichia coli DH1 uid162051
Escherichia coli DH1 uid161951
Escherichia coli B REL606 uid58803
Escherichia coli BL21 DE3 uid161949
Escherichia coli BL21 DE3 uid161947
Escherichia coli ATCC 8739 uid58783
Escherichia coli UMNK88 uid161991
Escherichia coli BL21 Gold DE3 pLysS AG uid59245
Shigella sonnei 53G uid84383
Shigella sonnei Ss046 uid58217
Shigella boydii Sb227 uid58215
Shigella boydii CDC 3083 94 uid58415
Escherichia coli clone D i2 uid162047
Escherichia coli clone D i14 uid162049
Shigella dysenteriae Sd197 uid58213
Escherichia coli Xuzhou21 uid163995
Escherichia coli W uid162101
Escherichia coli UTI89 uid58541
Escherichia coli UM146 uid162043
Escherichia coli SMS 3 5 uid58919
Escherichia coli SE15 uid161939
Escherichia coli SE11 uid59425
Escherichia coli O83 H1 NRG 857C uid161987
Escherichia coli O7 K1 CE10 uid162115
Escherichia coli O55 H7 RM12579 uid162153
Escherichia coli O55 H7 CB9615 uid46655
Escherichia coli O26 H11 11368 uid41021
Escherichia coli O157 H7 TW14359 uid59235
Escherichia coli O157 H7 Sakai uid57781
Escherichia coli O157 H7 EDL933 uid57831
Escherichia coli O157 H7 EC4115 uid59091
Escherichia coli O127 H6 E2348 69 uid59343
Escherichia coli O111 H 11128 uid41023
Escherichia coli NA114 uid162139
Escherichia coli LF82 uid161965
Escherichia coli KO11FL uid52593
Escherichia coli KO11FL uid162099
Escherichia coli IHE3034 uid162007
Escherichia coli E24377A uid58395
Escherichia coli CFT073 uid57915
Escherichia coli APEC O1 uid58623
Escherichia coli ABU 83972 uid161975
Escherichia coli 536 uid58531
Shigella flexneri 2002017 uid159233
Shigella flexneri 2a 2457T uid57991
Shigella flexneri 2a 301 uid62907
Shigella flexneri 5 8401 uid58583

rpsS

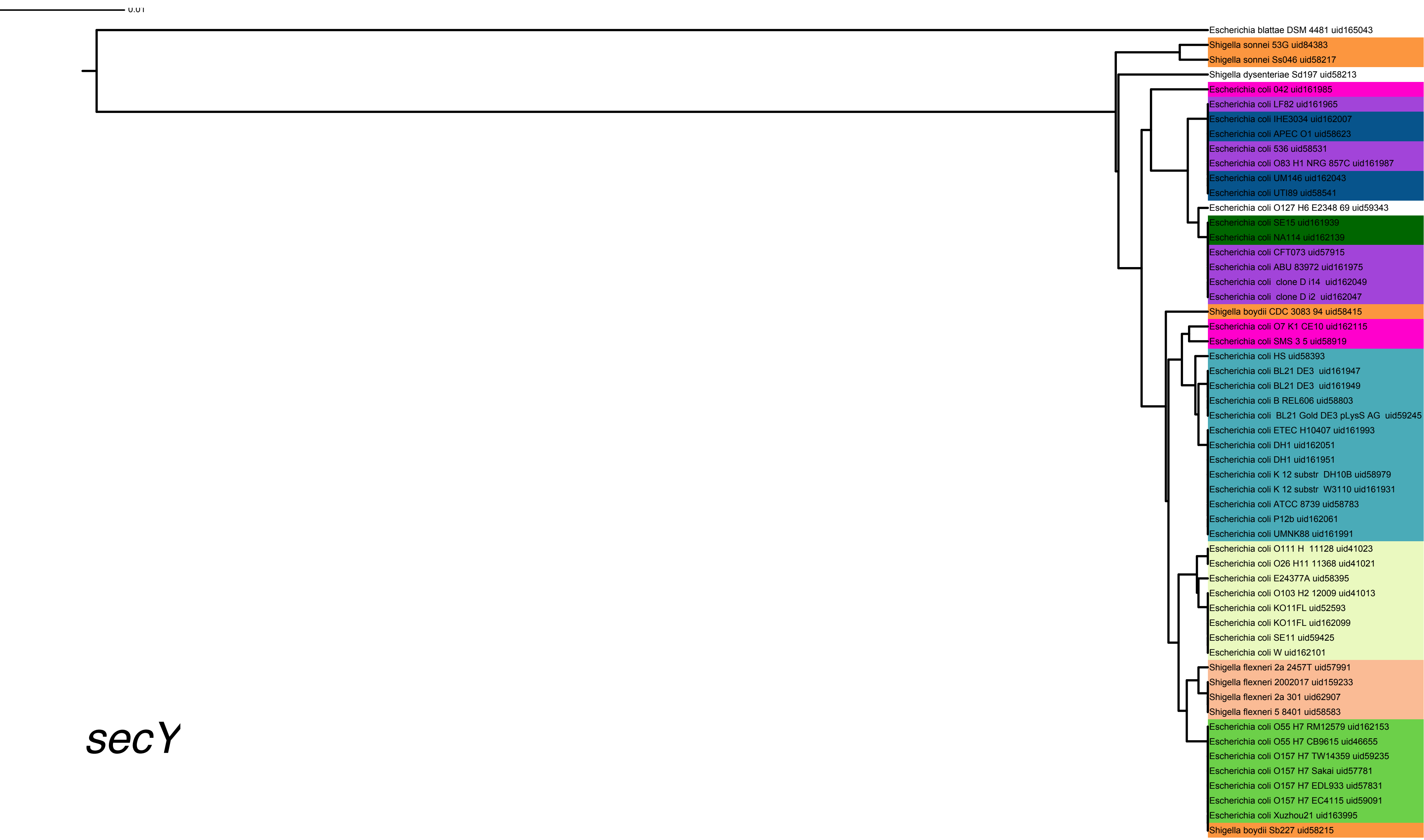
0.01
Escherichia blattae DSM 4481 uid165043
Shigella sonnei 53G uid84383
Shigella sonnei Ss046 uid58217
Shigella dysenteriae Sd197 uid58213
Escherichia coli 042 uid161985
Escherichia coli LF82 uid161965
Escherichia coli IHE3034 uid162007
Escherichia coli APEC O1 uid58623
Escherichia coli 536 uid58531
Escherichia coli O83 H1 NRG 857C uid161987
Escherichia coli UM146 uid162043
Escherichia coli UTI89 uid58541
Escherichia coli O127 H6 E2348 69 uid59343
Escherichia coli SE15 uid161939
Escherichia coli NA114 uid162139
Escherichia coli CFT073 uid57915
Escherichia coli ABU 83972 uid161975
Escherichia coli clone D i14 uid162049
Escherichia coli clone D i2 uid162047
Shigella boydii CDC 3083 94 uid58415
Escherichia coli O7 K1 CE10 uid162115
Escherichia coli SMS 3 5 uid58919
Escherichia coli HS uid58393
Escherichia coli BL21 DE3 uid161947
Escherichia coli BL21 DE3 uid161949
Escherichia coli B REL606 uid58803
Escherichia coli BL21 Gold DE3 pLysS AG uid59245
Escherichia coli ETEC H10407 uid161993
Escherichia coli DH1 uid162051
Escherichia coli DH1 uid161951
Escherichia coli K 12 substr DH10B uid58979
Escherichia coli K 12 substr W3110 uid161931
Escherichia coli ATCC 8739 uid58783
Escherichia coli P12b uid162061
Escherichia coli UMNK88 uid161991
Escherichia coli O111 H 11128 uid41023
Escherichia coli O26 H11 11368 uid41021
Escherichia coli E24377A uid58395
Escherichia coli O103 H2 12009 uid41013
Escherichia coli KO11FL uid52593
Escherichia coli KO11FL uid162099
Escherichia coli SE11 uid59425
Escherichia coli W uid162101
Shigella flexneri 2a 2457T uid57991
Shigella flexneri 2002017 uid159233
Shigella flexneri 2a 301 uid62907
Shigella flexneri 5 8401 uid58583
Escherichia coli O55 H7 RM12579 uid162153
Escherichia coli O55 H7 CB9615 uid46655
Escherichia coli O157 H7 TW14359 uid59235
Escherichia coli O157 H7 Sakai uid57781
Escherichia coli O157 H7 EDL933 uid57831
Escherichia coli O157 H7 EC4115 uid59091
Escherichia coli Xuzhou21 uid163995
Shigella boydii Sb227 uid58215
secY

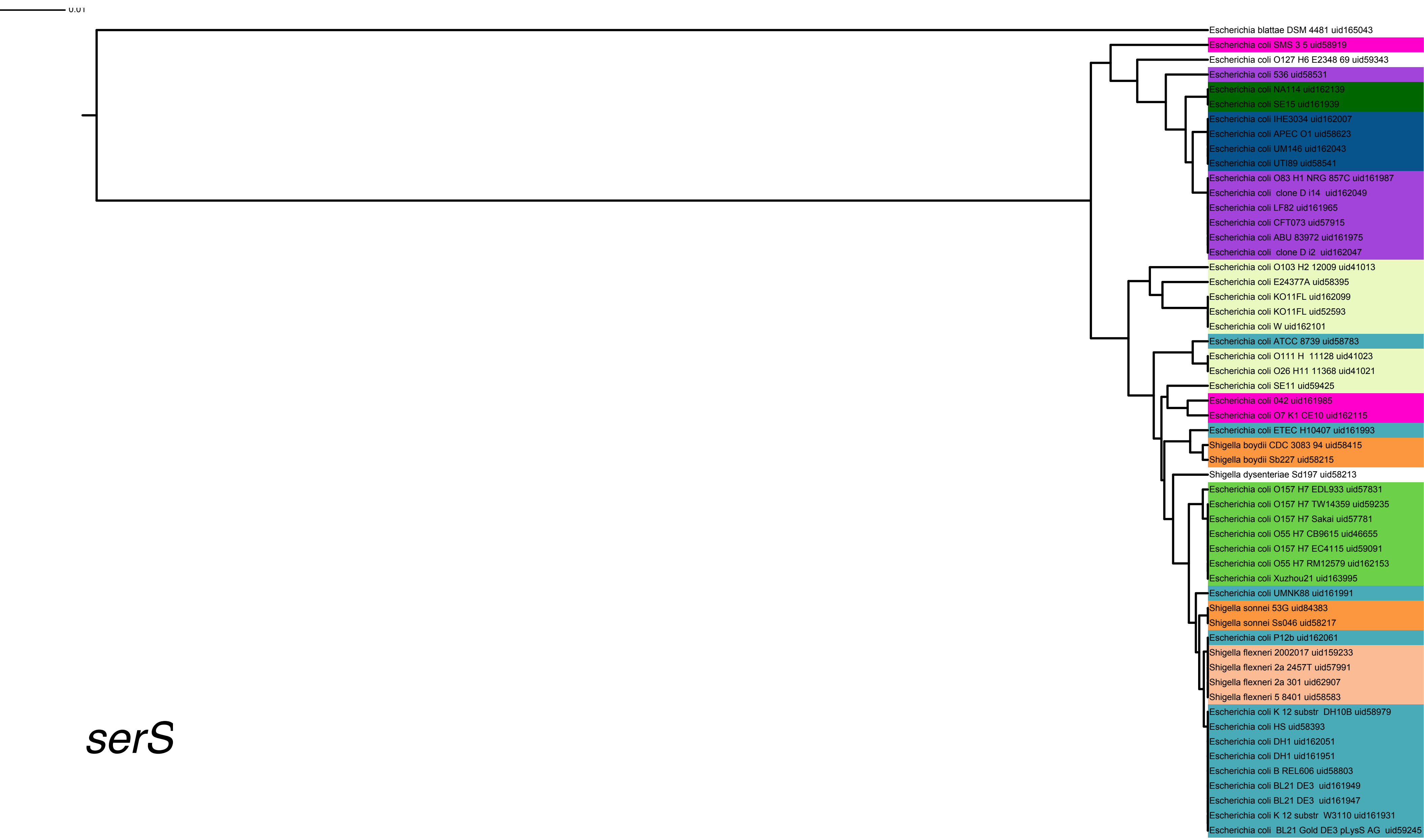
0.01
Escherichia blattae DSM 4481 uid165043
Escherichia coli SMS 3 5 uid58919
Escherichia coli O127 H6 E2348 69 uid59343
Escherichia coli 536 uid58531
Escherichia coli NA114 uid162139
Escherichia coli SE15 uid161939
Escherichia coli IHE3034 uid162007
Escherichia coli APEC O1 uid58623
Escherichia coli UM146 uid162043
Escherichia coli UTI89 uid58541
Escherichia coli O83 H1 NRG 857C uid161987
Escherichia coli clone D i14 uid162049
Escherichia coli LF82 uid161965
Escherichia coli CFT073 uid57915
Escherichia coli ABU 83972 uid161975
Escherichia coli clone D i2 uid162047
Escherichia coli O103 H2 12009 uid41013
Escherichia coli E24377A uid58395
Escherichia coli KO11FL uid162099
Escherichia coli KO11FL uid52593
Escherichia coli W uid162101
Escherichia coli ATCC 8739 uid58783
Escherichia coli O111 H 11128 uid41023
Escherichia coli O26 H11 11368 uid41021
Escherichia coli SE11 uid59425
Escherichia coli 042 uid161985
Escherichia coli O7 K1 CE10 uid162115
Escherichia coli ETEC H10407 uid161993
Shigella boydii CDC 3083 94 uid58415
Shigella boydii Sb227 uid58215
Shigella dysenteriae Sd197 uid58213
Escherichia coli O157 H7 EDL933 uid57831
Escherichia coli O157 H7 TW14359 uid59235
Escherichia coli O157 H7 Sakai uid57781
Escherichia coli O55 H7 CB9615 uid46655
Escherichia coli O157 H7 EC4115 uid59091
Escherichia coli O55 H7 RM12579 uid162153
Escherichia coli Xuzhou21 uid163995
Escherichia coli UMNK88 uid161991
Shigella sonnei 53G uid84383
Shigella sonnei Ss046 uid58217
Escherichia coli P12b uid162061
Shigella flexneri 2002017 uid159233
Shigella flexneri 2a 2457T uid57991
Shigella flexneri 2a 301 uid62907
Shigella flexneri 5 8401 uid58583
Escherichia coli K 12 substr DH10B uid58979
Escherichia coli HS uid58393
Escherichia coli DH1 uid162051
Escherichia coli DH1 uid161951
Escherichia coli B REL606 uid58803
Escherichia coli BL21 DE3 uid161949
Escherichia coli BL21 DE3 uid161947
Escherichia coli K 12 substr W3110 uid161931
Escherichia coli BL21 Gold DE3 pLysS AG uid59245
serS

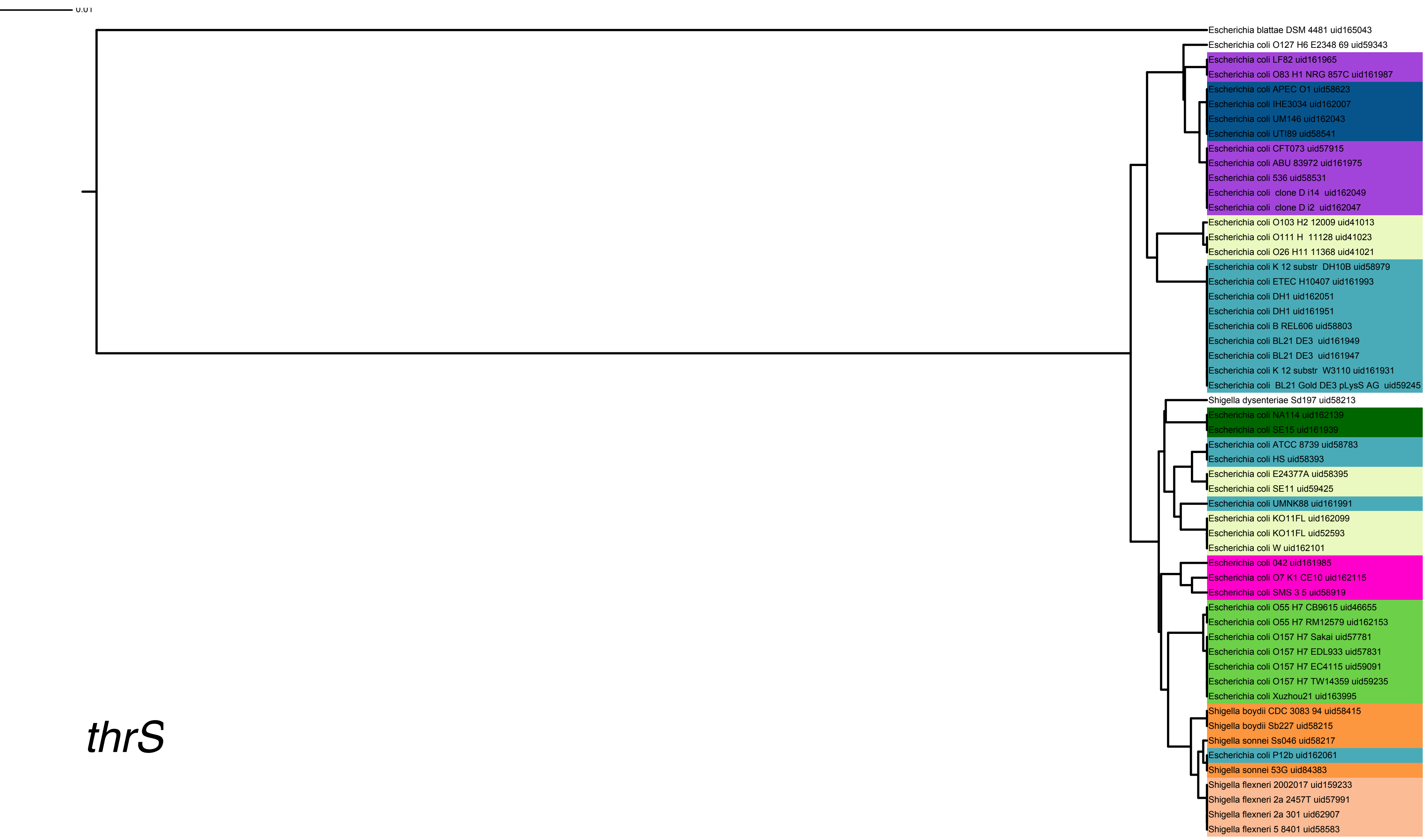
0.01
Escherichia blattae DSM 4481 uid165043
Escherichia coli O127 H6 E2348 69 uid59343
Escherichia coli LF82 uid161965
Escherichia coli O83 H1 NRG 857C uid161987
Escherichia coli APEC O1 uid58623
Escherichia coli IHE3034 uid162007
Escherichia coli UM146 uid162043
Escherichia coli UTI89 uid58541
Escherichia coli CFT073 uid57915
Escherichia coli ABU 83972 uid161975
Escherichia coli 536 uid58531
Escherichia coli clone D i14 uid162049
Escherichia coli clone D i2 uid162047
Escherichia coli O103 H2 12009 uid41013
Escherichia coli O111 H 11128 uid41023
Escherichia coli O26 H11 11368 uid41021
Escherichia coli K 12 substr DH10B uid58979
Escherichia coli ETEC H10407 uid161993
Escherichia coli DH1 uid162051
Escherichia coli DH1 uid161951
Escherichia coli B REL606 uid58803
Escherichia coli BL21 DE3 uid161949
Escherichia coli BL21 DE3 uid161947
Escherichia coli K 12 substr W3110 uid161931
Escherichia coli BL21 Gold DE3 pLysS AG uid59245
Shigella dysenteriae Sd197 uid58213
Escherichia coli NA114 uid162139
Escherichia coli SE15 uid161939
Escherichia coli ATCC 8739 uid58783
Escherichia coli HS uid58393
Escherichia coli E24377A uid58395
Escherichia coli SE11 uid59425
Escherichia coli UMNK88 uid161991
Escherichia coli KO11FL uid162099
Escherichia coli KO11FL uid52593
Escherichia coli W uid162101
Escherichia coli 042 uid161985
Escherichia coli O7 K1 CE10 uid162115
Escherichia coli SMS 3 5 uid58919
Escherichia coli O55 H7 CB9615 uid46655
Escherichia coli O55 H7 RM12579 uid162153
Escherichia coli O157 H7 Sakai uid57781
Escherichia coli O157 H7 EDL933 uid57831
Escherichia coli O157 H7 EC4115 uid59091
Escherichia coli O157 H7 TW14359 uid59235
Escherichia coli Xuzhou21 uid163995
Shigella boydii CDC 3083 94 uid58415
Shigella boydii Sb227 uid58215
Shigella sonnei Ss046 uid58217
Escherichia coli P12b uid162061
Shigella sonnei 53G uid84383
Shigella flexneri 2002017 uid159233
Shigella flexneri 2a 2457T uid57991
Shigella flexneri 2a 301 uid62907
Shigella flexneri 5 8401 uid58583
thrS

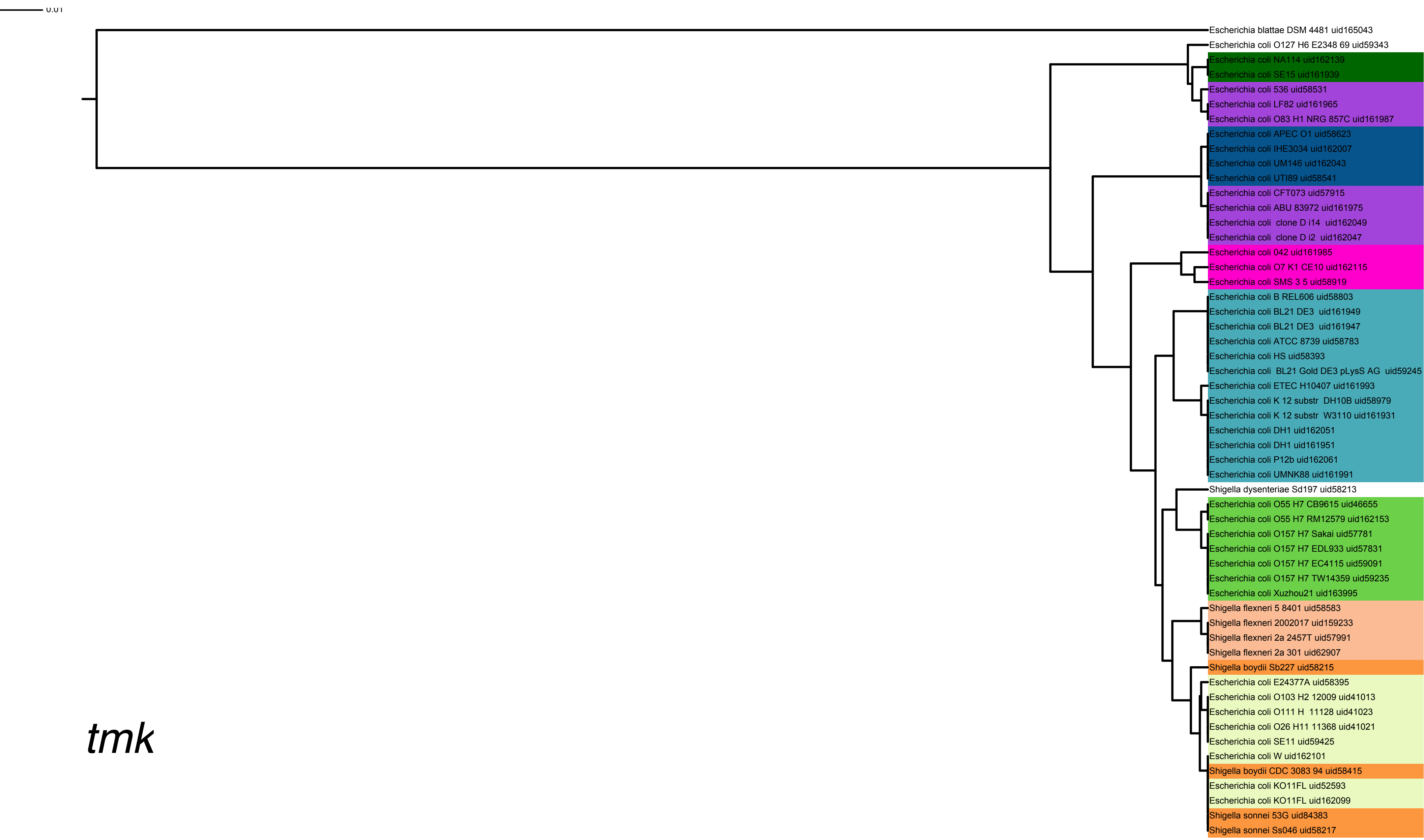
0.01
Escherichia blattae DSM 4481 uid165043
Escherichia coli O127 H6 E2348 69 uid59343
Escherichia coli NA114 uid162139
Escherichia coli SE15 uid161939
Escherichia coli 536 uid58531
Escherichia coli LF82 uid161965
Escherichia coli O83 H1 NRG 857C uid161987
Escherichia coli APEC O1 uid58623
Escherichia coli IHE3034 uid162007
Escherichia coli UM146 uid162043
Escherichia coli UTI89 uid58541
Escherichia coli CFT073 uid57915
Escherichia coli ABU 83972 uid161975
Escherichia coli clone D i14 uid162049
Escherichia coli clone D i2 uid162047
Escherichia coli 042 uid161985
Escherichia coli O7 K1 CE10 uid162115
Escherichia coli SMS 3 5 uid58919
Escherichia coli B REL606 uid58803
Escherichia coli BL21 DE3 uid161949
Escherichia coli BL21 DE3 uid161947
Escherichia coli ATCC 8739 uid58783
Escherichia coli HS uid58393
Escherichia coli BL21 Gold DE3 pLysS AG uid59245
Escherichia coli ETEC H10407 uid161993
Escherichia coli K 12 substr DH10B uid58979
Escherichia coli K 12 substr W3110 uid161931
Escherichia coli DH1 uid162051
Escherichia coli DH1 uid161951
Escherichia coli P12b uid162061
Escherichia coli UMNK88 uid161991
Shigella dysenteriae Sd197 uid58213
Escherichia coli O55 H7 CB9615 uid46655
Escherichia coli O55 H7 RM12579 uid162153
Escherichia coli O157 H7 Sakai uid57781
Escherichia coli O157 H7 EDL933 uid57831
Escherichia coli O157 H7 EC4115 uid59091
Escherichia coli O157 H7 TW14359 uid59235
Escherichia coli Xuzhou21 uid163995
Shigella flexneri 5 8401 uid58583
Shigella flexneri 2002017 uid159233
Shigella flexneri 2a 2457T uid57991
Shigella flexneri 2a 301 uid62907
Shigella boydii Sb227 uid58215
Escherichia coli E24377A uid58395
Escherichia coli O103 H2 12009 uid41013
Escherichia coli O111 H 11128 uid41023
Escherichia coli O26 H11 11368 uid41021
Escherichia coli SE11 uid59425
Escherichia coli W uid162101
Shigella boydii CDC 3083 94 uid58415
Escherichia coli KO11FL uid52593
Escherichia coli KO11FL uid162099
Shigella sonnei 53G uid84383
Shigella sonnei Ss046 uid58217
tmk

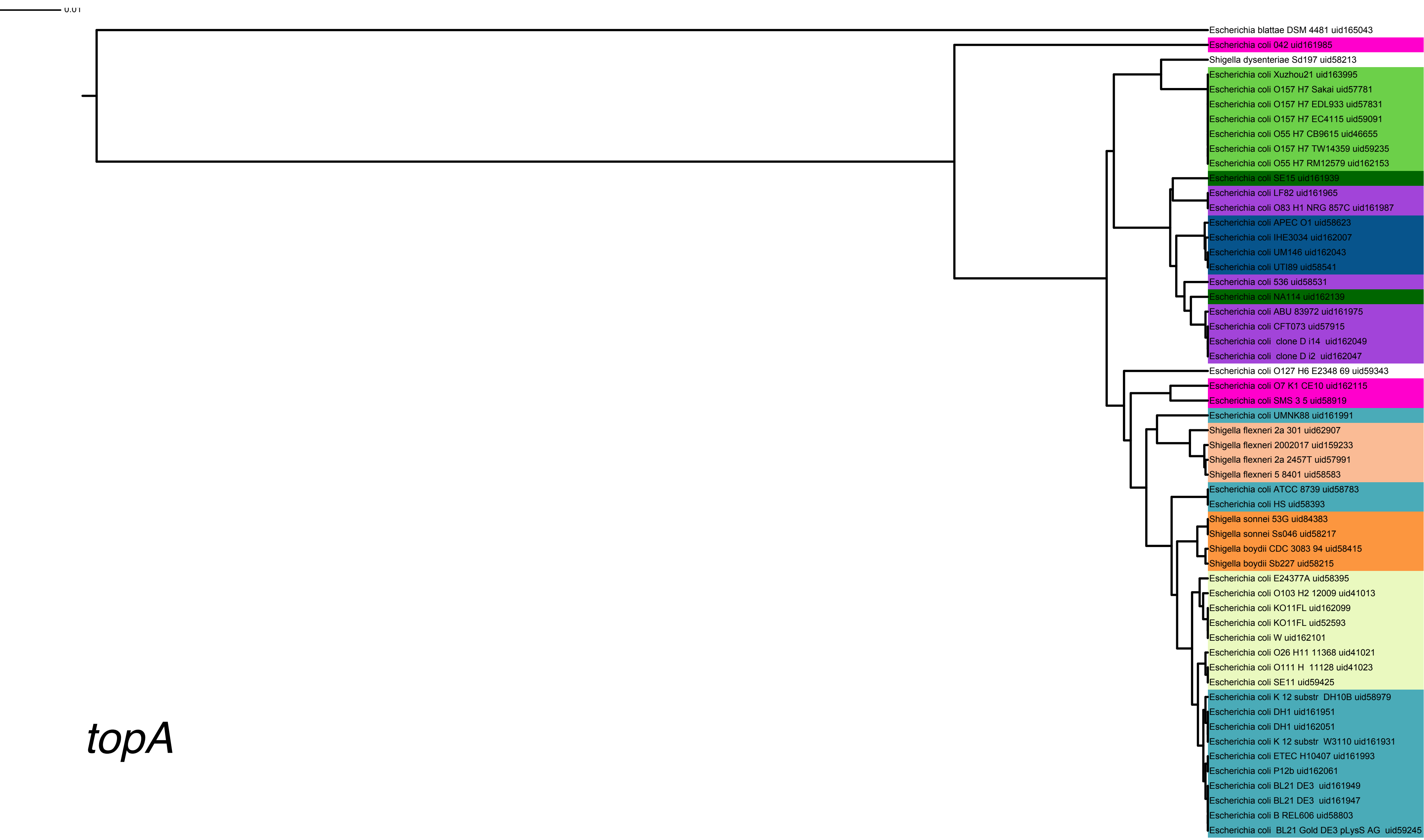

0.01
Escherichia blattae DSM 4481 uid165043
Escherichia coli 042 uid161985
Shigella dysenteriae Sd197 uid58213
Escherichia coli Xuzhou21 uid163995
Escherichia coli O157 H7 Sakai uid57781
Escherichia coli O157 H7 EDL933 uid57831
Escherichia coli O157 H7 EC4115 uid59091
Escherichia coli O55 H7 CB9615 uid46655
Escherichia coli O157 H7 TW14359 uid59235
Escherichia coli O55 H7 RM12579 uid162153
Escherichia coli SE15 uid161939
Escherichia coli LF82 uid161965
Escherichia coli O83 H1 NRG 857C uid161987
Escherichia coli APEC O1 uid58623
Escherichia coli IHE3034 uid162007
Escherichia coli UM146 uid162043
Escherichia coli UTI89 uid58541
Escherichia coli 536 uid58531
Escherichia coli NA114 uid162139
Escherichia coli ABU 83972 uid161975
Escherichia coli CFT073 uid57915
Escherichia coli clone D i14 uid162049
Escherichia coli clone D i2 uid162047
Escherichia coli O127 H6 E2348 69 uid59343
Escherichia coli O7 K1 CE10 uid162115
Escherichia coli SMS 3 5 uid58919
Escherichia coli UMNK88 uid161991
Shigella flexneri 2a 301 uid62907
Shigella flexneri 2002017 uid159233
Shigella flexneri 2a 2457T uid57991
Shigella flexneri 5 8401 uid58583
Escherichia coli ATCC 8739 uid58783
Escherichia coli HS uid58393
Shigella sonnei 53G uid84383
Shigella sonnei Ss046 uid58217
Shigella boydii CDC 3083 94 uid58415
Shigella boydii Sb227 uid58215
Escherichia coli E24377A uid58395
Escherichia coli O103 H2 12009 uid41013
Escherichia coli KO11FL uid162099
Escherichia coli KO11FL uid52593
Escherichia coli W uid162101
Escherichia coli O26 H11 11368 uid41021
Escherichia coli O111 H 11128 uid41023
Escherichia coli SE11 uid59425
Escherichia coli K 12 substr DH10B uid58979
Escherichia coli DH1 uid161951
Escherichia coli DH1 uid162051
Escherichia coli K 12 substr W3110 uid161931
Escherichia coli ETEC H10407 uid161993
Escherichia coli P12b uid162061
Escherichia coli BL21 DE3 uid161949
Escherichia coli BL21 DE3 uid161947
Escherichia coli B REL606 uid58803
Escherichia coli BL21 Gold DE3 pLysS AG uid59245

topA

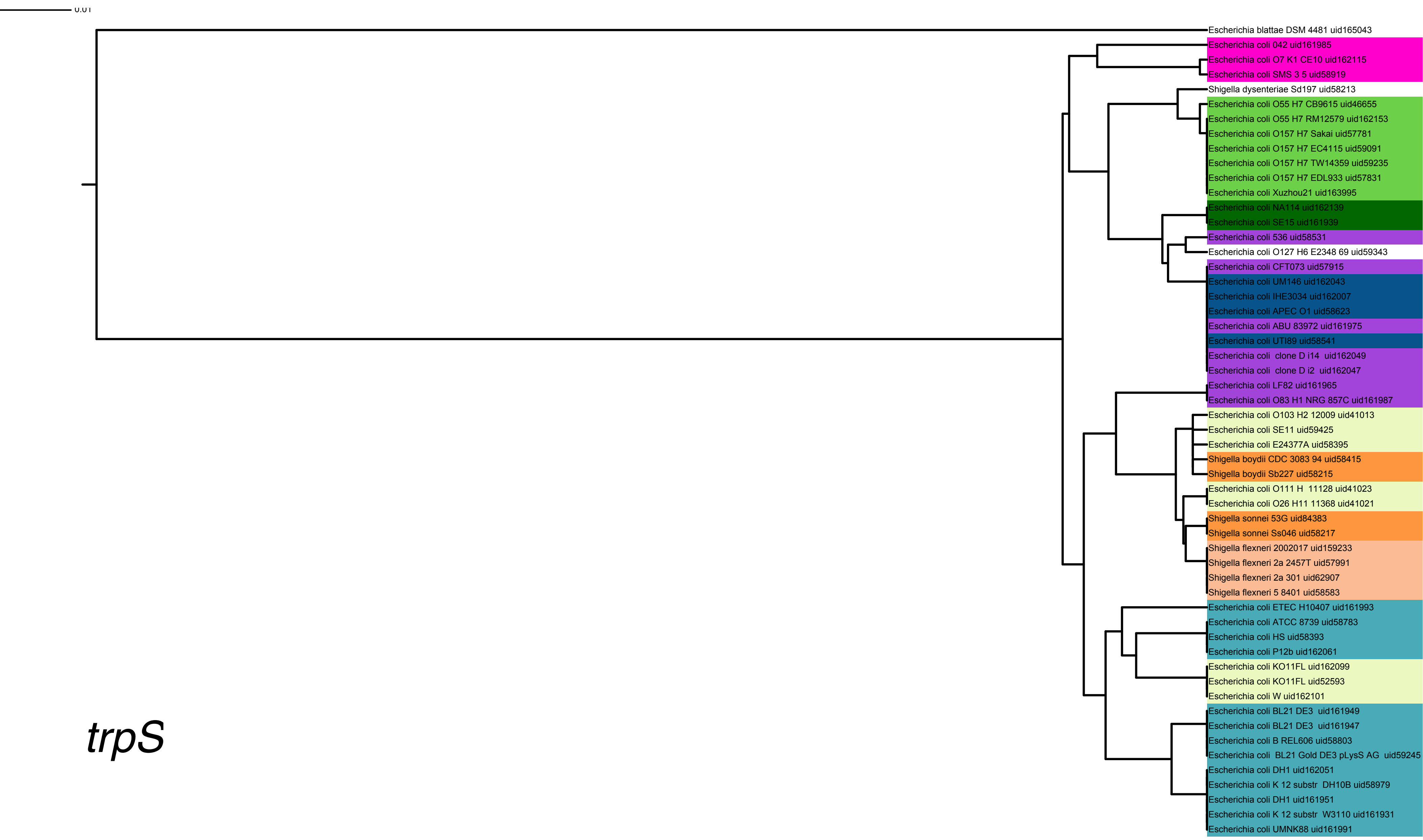

0.01
Escherichia blattae DSM 4481 uid165043
Escherichia coli 042 uid161985
Escherichia coli O7 K1 CE10 uid162115
Escherichia coli SMS 3 5 uid58919
Shigella dysenteriae Sd197 uid58213
Escherichia coli O55 H7 CB9615 uid46655
Escherichia coli O55 H7 RM12579 uid162153
Escherichia coli O157 H7 Sakai uid57781
Escherichia coli O157 H7 EC4115 uid59091
Escherichia coli O157 H7 TW14359 uid59235
Escherichia coli O157 H7 EDL933 uid57831
Escherichia coli Xuzhou21 uid163995
Escherichia coli NA114 uid162139
Escherichia coli SE15 uid161939
Escherichia coli 536 uid58531
Escherichia coli O127 H6 E2348 69 uid59343
Escherichia coli CFT073 uid57915
Escherichia coli UM146 uid162043
Escherichia coli IHE3034 uid162007
Escherichia coli APEC O1 uid58623
Escherichia coli ABU 83972 uid161975
Escherichia coli UTI89 uid58541
Escherichia coli clone D i14 uid162049
Escherichia coli clone D i2 uid162047
Escherichia coli LF82 uid161965
Escherichia coli O83 H1 NRG 857C uid161987
Escherichia coli O103 H2 12009 uid41013
Escherichia coli SE11 uid59425
Escherichia coli E24377A uid58395
Shigella boydii CDC 3083 94 uid58415
Shigella boydii Sb227 uid58215
Escherichia coli O111 H 11128 uid41023
Escherichia coli O26 H11 11368 uid41021
Shigella sonnei 53G uid84383
Shigella sonnei Ss046 uid58217
Shigella flexneri 2002017 uid159233
Shigella flexneri 2a 2457T uid57991
Shigella flexneri 2a 301 uid62907
Shigella flexneri 5 8401 uid58583
Escherichia coli ETEC H10407 uid161993
Escherichia coli ATCC 8739 uid58783
Escherichia coli HS uid58393
Escherichia coli P12b uid162061
Escherichia coli KO11FL uid162099
Escherichia coli KO11FL uid52593
Escherichia coli W uid162101
Escherichia coli BL21 DE3 uid161949
Escherichia coli BL21 DE3 uid161947
Escherichia coli B REL606 uid58803
Escherichia coli BL21 Gold DE3 pLysS AG uid59245
Escherichia coli DH1 uid162051
Escherichia coli K 12 substr DH10B uid58979
Escherichia coli DH1 uid161951
Escherichia coli K 12 substr W3110 uid161931
Escherichia coli UMNK88 uid161991
trpS

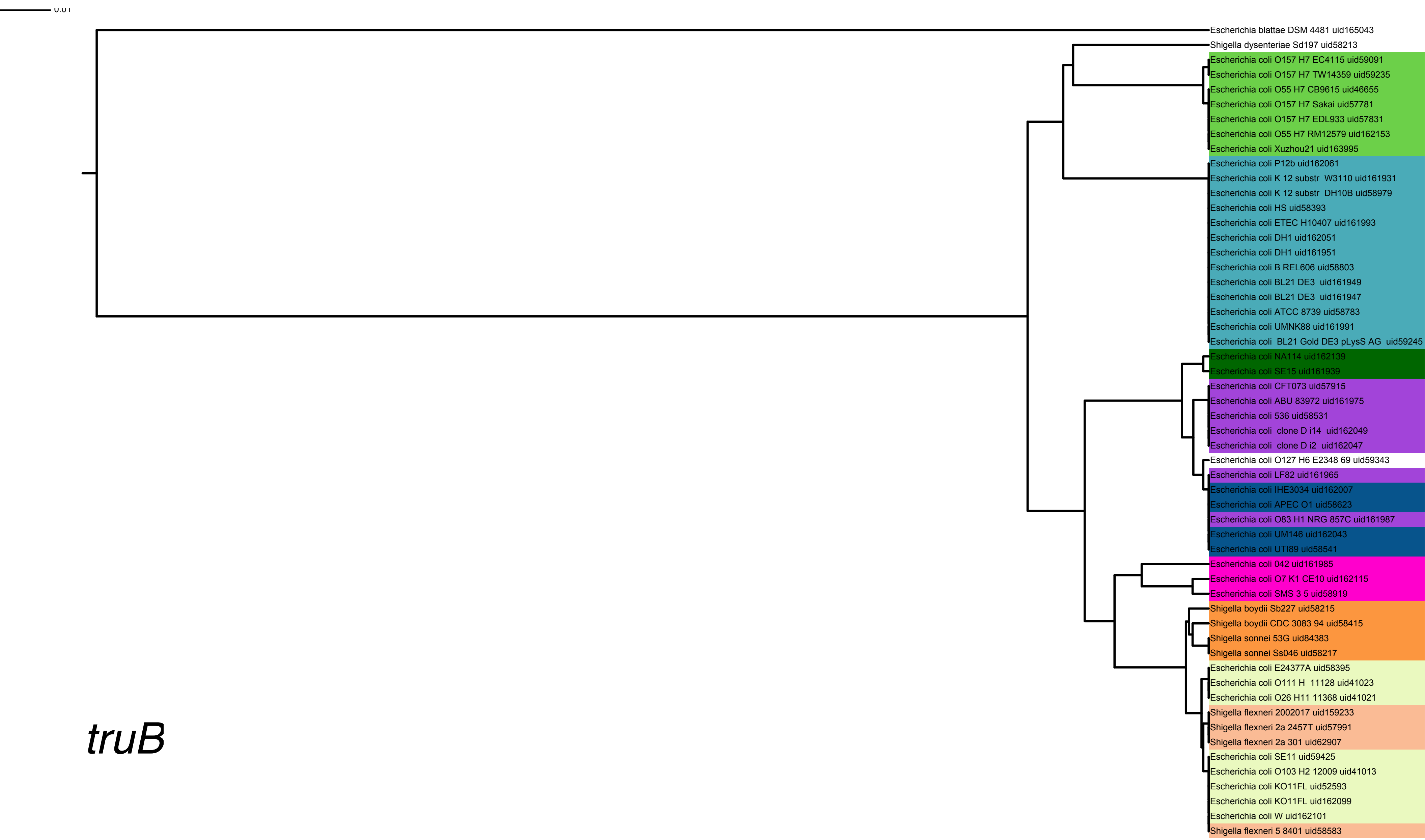
0.01
Escherichia blattae DSM 4481 uid165043
Shigella dysenteriae Sd197 uid58213
Escherichia coli O157 H7 EC4115 uid59091
Escherichia coli O157 H7 TW14359 uid59235
Escherichia coli O55 H7 CB9615 uid46655
Escherichia coli O157 H7 Sakai uid57781
Escherichia coli O157 H7 EDL933 uid57831
Escherichia coli O55 H7 RM12579 uid162153
Escherichia coli Xuzhou21 uid163995
Escherichia coli P12b uid162061
Escherichia coli K 12 substr W3110 uid161931
Escherichia coli K 12 substr DH10B uid58979
Escherichia coli HS uid58393
Escherichia coli ETEC H10407 uid161993
Escherichia coli DH1 uid162051
Escherichia coli DH1 uid161951
Escherichia coli B REL606 uid58803
Escherichia coli BL21 DE3 uid161949
Escherichia coli BL21 DE3 uid161947
Escherichia coli ATCC 8739 uid58783
Escherichia coli UMNK88 uid161991
Escherichia coli BL21 Gold DE3 pLysS AG uid59245
Escherichia coli NA114 uid162139
Escherichia coli SE15 uid161939
Escherichia coli CFT073 uid57915
Escherichia coli ABU 83972 uid161975
Escherichia coli 536 uid58531
Escherichia coli clone D i14 uid162049
Escherichia coli clone D i2 uid162047
Escherichia coli O127 H6 E2348 69 uid59343
Escherichia coli LF82 uid161965
Escherichia coli IHE3034 uid162007
Escherichia coli APEC O1 uid58623
Escherichia coli O83 H1 NRG 857C uid161987
Escherichia coli UM146 uid162043
Escherichia coli UTI89 uid58541
Escherichia coli 042 uid161985
Escherichia coli O7 K1 CE10 uid162115
Escherichia coli SMS 3 5 uid58919
Shigella boydii Sb227 uid58215
Shigella boydii CDC 3083 94 uid58415
Shigella sonnei 53G uid84383
Shigella sonnei Ss046 uid58217
Escherichia coli E24377A uid58395
Escherichia coli O111 H 11128 uid41023
Escherichia coli O26 H11 11368 uid41021
Shigella flexneri 2002017 uid159233
Shigella flexneri 2a 2457T uid57991
Shigella flexneri 2a 301 uid62907
Escherichia coli SE11 uid59425
Escherichia coli O103 H2 12009 uid41013
Escherichia coli KO11FL uid52593
Escherichia coli KO11FL uid162099
Escherichia coli W uid162101
Shigella flexneri 5 8401 uid58583
truB

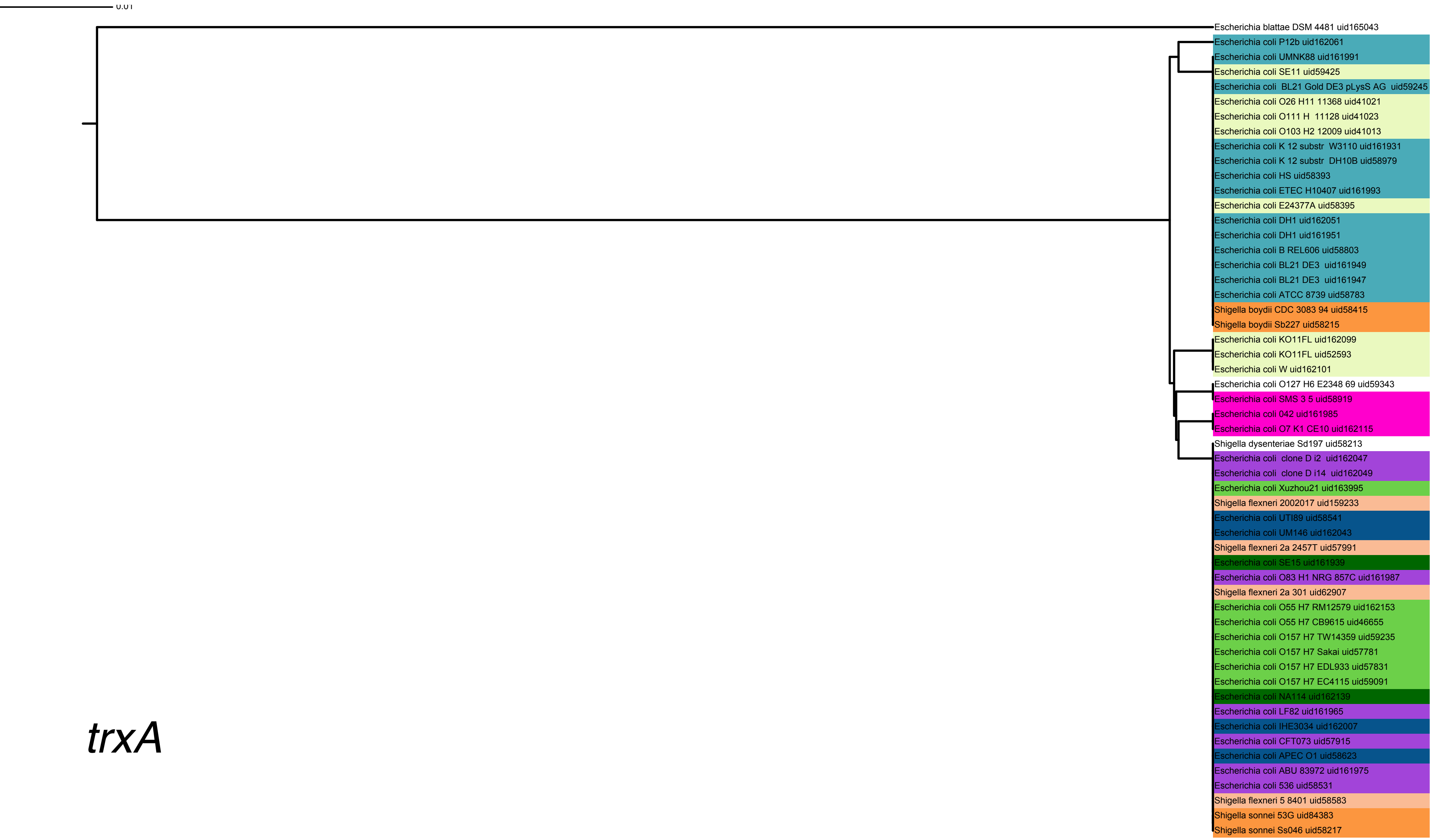
0.01
Escherichia blattae DSM 4481 uid165043
Escherichia coli P12b uid162061
Escherichia coli UMNK88 uid161991
Escherichia coli SE11 uid59425
Escherichia coli BL21 Gold DE3 pLysS AG uid59245
Escherichia coli O26 H11 11368 uid41021
Escherichia coli O111 H 11128 uid41023
Escherichia coli O103 H2 12009 uid41013
Escherichia coli K 12 substr W3110 uid161931
Escherichia coli K 12 substr DH10B uid58979
Escherichia coli HS uid58393
Escherichia coli ETEC H10407 uid161993
Escherichia coli E24377A uid58395
Escherichia coli DH1 uid162051
Escherichia coli DH1 uid161951
Escherichia coli B REL606 uid58803
Escherichia coli BL21 DE3 uid161949
Escherichia coli BL21 DE3 uid161947
Escherichia coli ATCC 8739 uid58783
Shigella boydii CDC 3083 94 uid58415
Shigella boydii Sb227 uid58215
Escherichia coli KO11FL uid162099
Escherichia coli KO11FL uid52593
Escherichia coli W uid162101
Escherichia coli O127 H6 E2348 69 uid59343
Escherichia coli SMS 3 5 uid58919
Escherichia coli 042 uid161985
Escherichia coli O7 K1 CE10 uid162115
Shigella dysenteriae Sd197 uid58213
Escherichia coli clone D i2 uid162047
Escherichia coli clone D i14 uid162049
Escherichia coli Xuzhou21 uid163995
Shigella flexneri 2002017 uid159233
Escherichia coli UTI89 uid58541
Escherichia coli UM146 uid162043
Shigella flexneri 2a 2457T uid57991
Escherichia coli SE15 uid161939
Escherichia coli O83 H1 NRG 857C uid161987
Shigella flexneri 2a 301 uid62907
Escherichia coli O55 H7 RM12579 uid162153
Escherichia coli O55 H7 CB9615 uid46655
Escherichia coli O157 H7 TW14359 uid59235
Escherichia coli O157 H7 Sakai uid57781
Escherichia coli O157 H7 EDL933 uid57831
Escherichia coli O157 H7 EC4115 uid59091
Escherichia coli NA114 uid162139
Escherichia coli LF82 uid161965
Escherichia coli IHE3034 uid162007
Escherichia coli CFT073 uid57915
Escherichia coli APEC O1 uid58623
Escherichia coli ABU 83972 uid161975
Escherichia coli 536 uid58531
Shigella flexneri 5 8401 uid58583
Shigella sonnei 53G uid84383
Shigella sonnei Ss046 uid58217
trxA

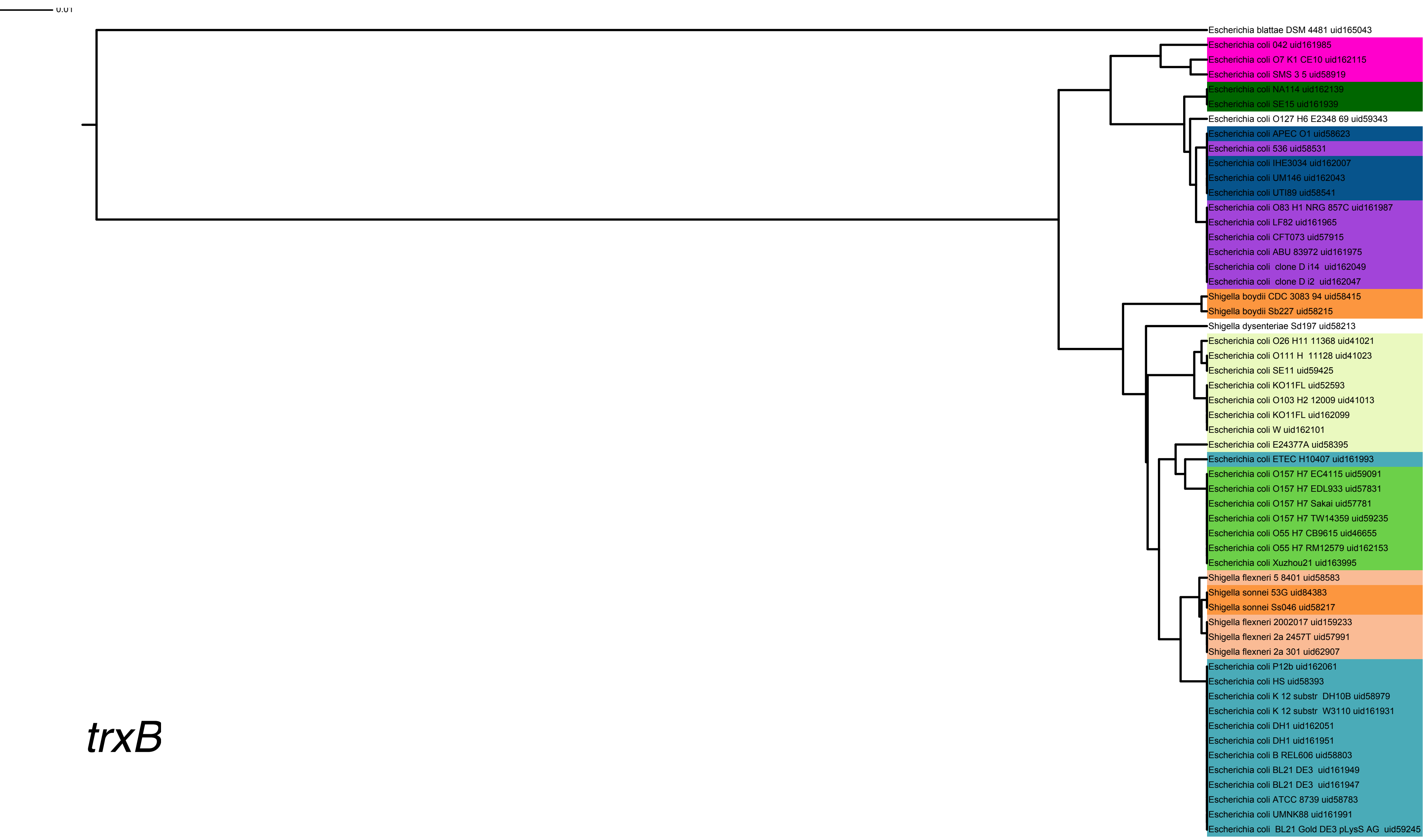
0.01
Escherichia blattae DSM 4481 uid165043
Escherichia coli 042 uid161985
Escherichia coli O7 K1 CE10 uid162115
Escherichia coli SMS 3 5 uid58919
Escherichia coli NA114 uid162139
Escherichia coli SE15 uid161939
Escherichia coli O127 H6 E2348 69 uid59343
Escherichia coli APEC O1 uid58623
Escherichia coli 536 uid58531
Escherichia coli IHE3034 uid162007
Escherichia coli UM146 uid162043
Escherichia coli UTI89 uid58541
Escherichia coli O83 H1 NRG 857C uid161987
Escherichia coli LF82 uid161965
Escherichia coli CFT073 uid57915
Escherichia coli ABU 83972 uid161975
Escherichia coli clone D i14 uid162049
Escherichia coli clone D i2 uid162047
Shigella boydii CDC 3083 94 uid58415
Shigella boydii Sb227 uid58215
Shigella dysenteriae Sd197 uid58213
Escherichia coli O26 H11 11368 uid41021
Escherichia coli O111 H 11128 uid41023
Escherichia coli SE11 uid59425
Escherichia coli KO11FL uid52593
Escherichia coli O103 H2 12009 uid41013
Escherichia coli KO11FL uid162099
Escherichia coli W uid162101
Escherichia coli E24377A uid58395
Escherichia coli ETEC H10407 uid161993
Escherichia coli O157 H7 EC4115 uid59091
Escherichia coli O157 H7 EDL933 uid57831
Escherichia coli O157 H7 Sakai uid57781
Escherichia coli O157 H7 TW14359 uid59235
Escherichia coli O55 H7 CB9615 uid46655
Escherichia coli O55 H7 RM12579 uid162153
Escherichia coli Xuzhou21 uid163995
Shigella flexneri 5 8401 uid58583
Shigella sonnei 53G uid84383
Shigella sonnei Ss046 uid58217
Shigella flexneri 2002017 uid159233
Shigella flexneri 2a 2457T uid57991
Shigella flexneri 2a 301 uid62907
Escherichia coli P12b uid162061
Escherichia coli HS uid58393
Escherichia coli K 12 substr DH10B uid58979
Escherichia coli K 12 substr W3110 uid161931
Escherichia coli DH1 uid162051
Escherichia coli DH1 uid161951
Escherichia coli B REL606 uid58803
Escherichia coli BL21 DE3 uid161949
Escherichia coli BL21 DE3 uid161947
Escherichia coli ATCC 8739 uid58783
Escherichia coli UMNK88 uid161991
Escherichia coli BL21 Gold DE3 pLysS AG uid59245
trxB

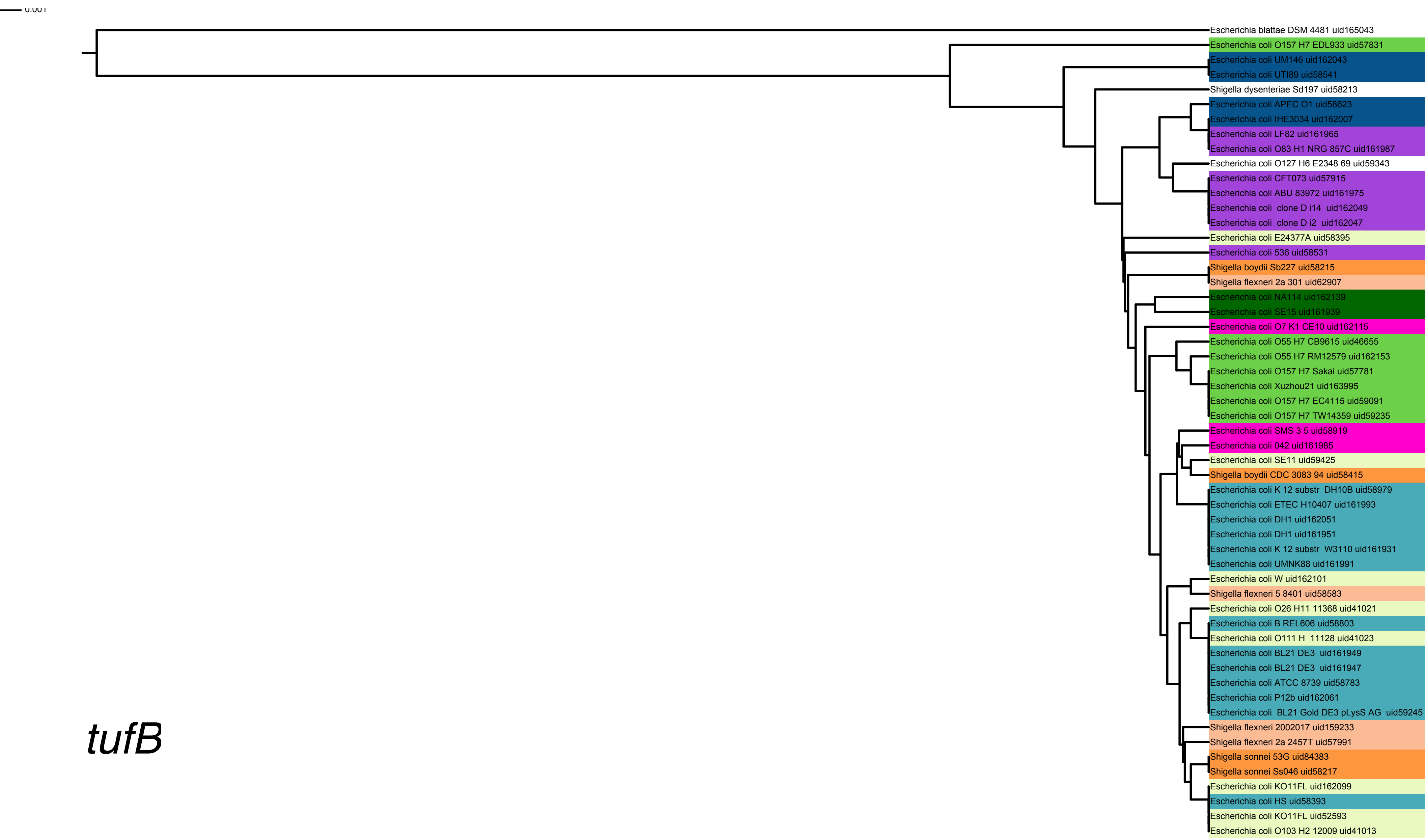
0.001
Escherichia blattae DSM 4481 uid165043
Escherichia coli O157 H7 EDL933 uid57831
Escherichia coli UM146 uid162043
Escherichia coli UTI89 uid58541
Shigella dysenteriae Sd197 uid58213
Escherichia coli APEC O1 uid58623
Escherichia coli IHE3034 uid162007
Escherichia coli LF82 uid161965
Escherichia coli O83 H1 NRG 857C uid161987
Escherichia coli O127 H6 E2348 69 uid59343
Escherichia coli CFT073 uid57915
Escherichia coli ABU 83972 uid161975
Escherichia coli clone D i14 uid162049
Escherichia coli clone D i2 uid162047
Escherichia coli E24377A uid58395
Escherichia coli 536 uid58531
Shigella boydii Sb227 uid58215
Shigella flexneri 2a 301 uid62907
Escherichia coli NA114 uid162139
Escherichia coli SE15 uid161939
Escherichia coli O7 K1 CE10 uid162115
Escherichia coli O55 H7 CB9615 uid46655
Escherichia coli O55 H7 RM12579 uid162153
Escherichia coli O157 H7 Sakai uid57781
Escherichia coli Xuzhou21 uid163995
Escherichia coli O157 H7 EC4115 uid59091
Escherichia coli O157 H7 TW14359 uid59235
Escherichia coli SMS 3 5 uid58919
Escherichia coli 042 uid161985
Escherichia coli SE11 uid59425
Shigella boydii CDC 3083 94 uid58415
Escherichia coli K 12 substr DH10B uid58979
Escherichia coli ETEC H10407 uid161993
Escherichia coli DH1 uid162051
Escherichia coli DH1 uid161951
Escherichia coli K 12 substr W3110 uid161931
Escherichia coli UMNK88 uid161991
Escherichia coli W uid162101
Shigella flexneri 5 8401 uid58583
Escherichia coli O26 H11 11368 uid41021
Escherichia coli B REL606 uid58803
Escherichia coli O111 H 11128 uid41023
Escherichia coli BL21 DE3 uid161949
Escherichia coli BL21 DE3 uid161947
Escherichia coli ATCC 8739 uid58783
Escherichia coli P12b uid162061
Escherichia coli BL21 Gold DE3 pLysS AG uid59245
Shigella flexneri 2002017 uid159233
Shigella flexneri 2a 2457T uid57991
Shigella sonnei 53G uid84383
Shigella sonnei Ss046 uid58217
Escherichia coli KO11FL uid162099
Escherichia coli HS uid58393
Escherichia coli KO11FL uid52593
Escherichia coli O103 H2 12009 uid41013
tufB

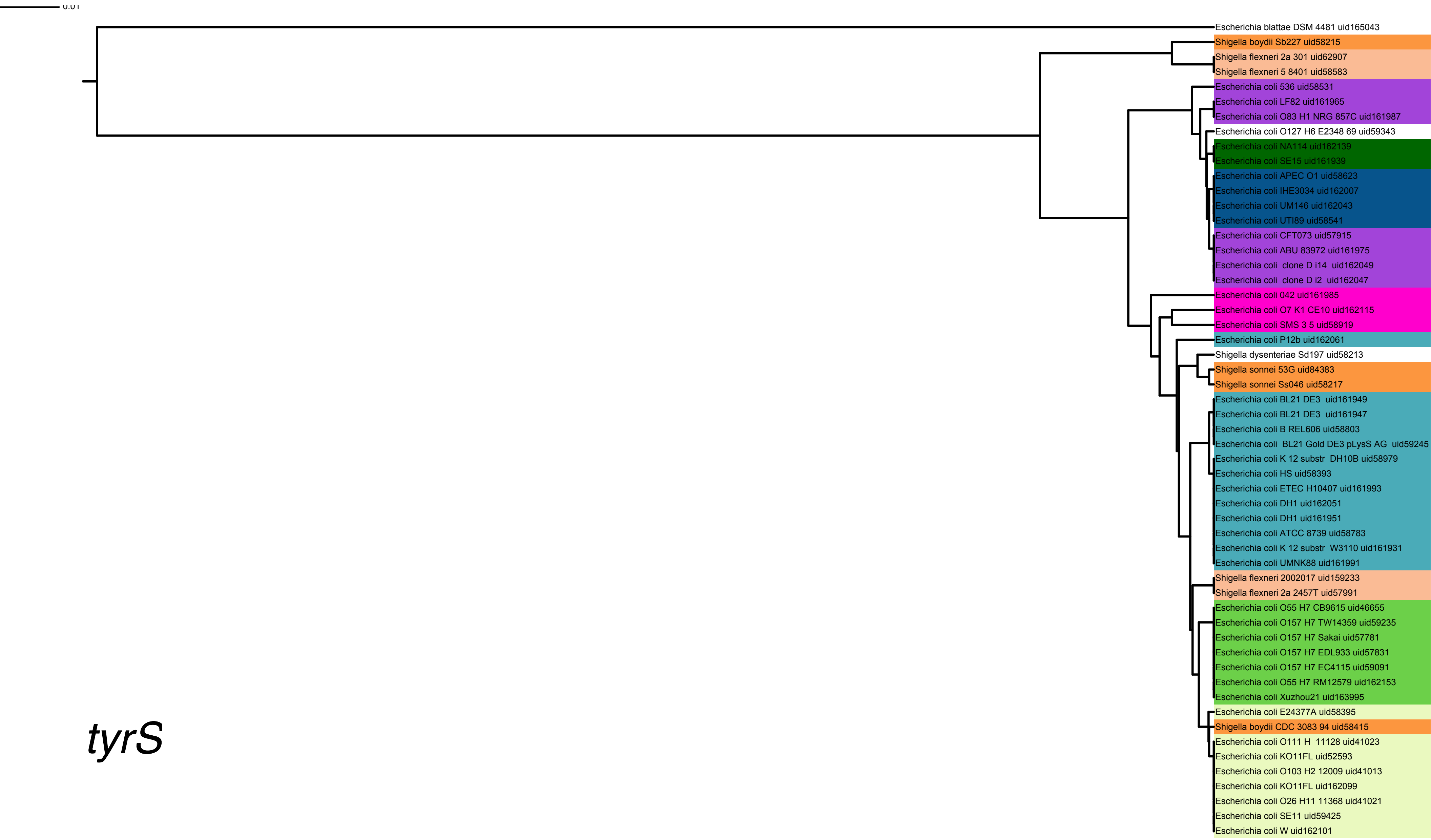
0.01
Escherichia blattae DSM 4481 uid165043
Shigella boydii Sb227 uid58215
Shigella flexneri 2a 301 uid62907
Shigella flexneri 5 8401 uid58583
Escherichia coli 536 uid58531
Escherichia coli LF82 uid161965
Escherichia coli O83 H1 NRG 857C uid161987
Escherichia coli O127 H6 E2348 69 uid59343
Escherichia coli NA114 uid162139
Escherichia coli SE15 uid161939
Escherichia coli APEC O1 uid58623
Escherichia coli IHE3034 uid162007
Escherichia coli UM146 uid162043
Escherichia coli UTI89 uid58541
Escherichia coli CFT073 uid57915
Escherichia coli ABU 83972 uid161975
Escherichia coli clone D i14 uid162049
Escherichia coli clone D i2 uid162047
Escherichia coli 042 uid161985
Escherichia coli O7 K1 CE10 uid162115
Escherichia coli SMS 3 5 uid58919
Escherichia coli P12b uid162061
Shigella dysenteriae Sd197 uid58213
Shigella sonnei 53G uid84383
Shigella sonnei Ss046 uid58217
Escherichia coli BL21 DE3 uid161949
Escherichia coli BL21 DE3 uid161947
Escherichia coli B REL606 uid58803
Escherichia coli BL21 Gold DE3 pLysS AG uid59245
Escherichia coli K 12 substr DH10B uid58979
Escherichia coli HS uid58393
Escherichia coli ETEC H10407 uid161993
Escherichia coli DH1 uid162051
Escherichia coli DH1 uid161951
Escherichia coli ATCC 8739 uid58783
Escherichia coli K 12 substr W3110 uid161931
Escherichia coli UMNK88 uid161991
Shigella flexneri 2002017 uid159233
Shigella flexneri 2a 2457T uid57991
Escherichia coli O55 H7 CB9615 uid46655
Escherichia coli O157 H7 TW14359 uid59235
Escherichia coli O157 H7 Sakai uid57781
Escherichia coli O157 H7 EDL933 uid57831
Escherichia coli O157 H7 EC4115 uid59091
Escherichia coli O55 H7 RM12579 uid162153
Escherichia coli Xuzhou21 uid163995
Escherichia coli E24377A uid58395
Shigella boydii CDC 3083 94 uid58415
Escherichia coli O111 H 11128 uid41023
Escherichia coli KO11FL uid52593
Escherichia coli O103 H2 12009 uid41013
Escherichia coli KO11FL uid162099
Escherichia coli O26 H11 11368 uid41021
Escherichia coli SE11 uid59425
Escherichia coli W uid162101

tyrS

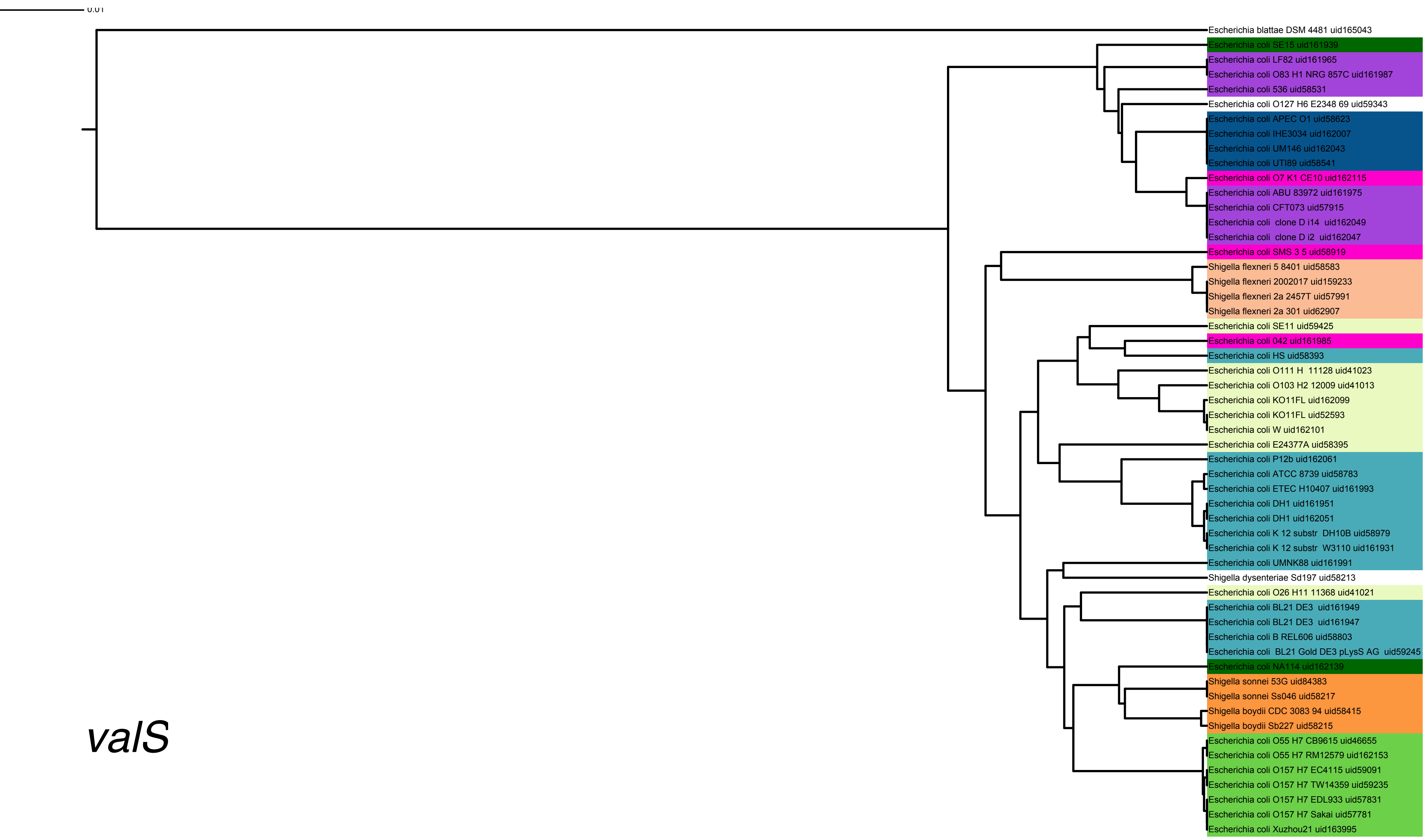
0.01
Escherichia blattae DSM 4481 uid165043
Escherichia coli SE15 uid161939
Escherichia coli LF82 uid161965
Escherichia coli O83 H1 NRG 857C uid161987
Escherichia coli 536 uid58531
Escherichia coli O127 H6 E2348 69 uid59343
Escherichia coli APEC O1 uid58623
Escherichia coli IHE3034 uid162007
Escherichia coli UM146 uid162043
Escherichia coli UTI89 uid58541
Escherichia coli O7 K1 CE10 uid162115
Escherichia coli ABU 83972 uid161975
Escherichia coli CFT073 uid57915
Escherichia coli clone D i14 uid162049
Escherichia coli clone D i2 uid162047
Escherichia coli SMS 3 5 uid58919
Shigella flexneri 5 8401 uid58583
Shigella flexneri 2002017 uid159233
Shigella flexneri 2a 2457T uid57991
Shigella flexneri 2a 301 uid62907
Escherichia coli SE11 uid59425
Escherichia coli 042 uid161985
Escherichia coli HS uid58393
Escherichia coli O111 H 11128 uid41023
Escherichia coli O103 H2 12009 uid41013
Escherichia coli KO11FL uid162099
Escherichia coli KO11FL uid52593
Escherichia coli W uid162101
Escherichia coli E24377A uid58395
Escherichia coli P12b uid162061
Escherichia coli ATCC 8739 uid58783
Escherichia coli ETEC H10407 uid161993
Escherichia coli DH1 uid161951
Escherichia coli DH1 uid162051
Escherichia coli K 12 substr DH10B uid58979
Escherichia coli K 12 substr W3110 uid161931
Escherichia coli UMNK88 uid161991
Shigella dysenteriae Sd197 uid58213
Escherichia coli O26 H11 11368 uid41021
Escherichia coli BL21 DE3 uid161949
Escherichia coli BL21 DE3 uid161947
Escherichia coli B REL606 uid58803
Escherichia coli BL21 Gold DE3 pLysS AG uid59245
Escherichia coli NA114 uid162139
Shigella sonnei 53G uid84383
Shigella sonnei Ss046 uid58217
Shigella boydii CDC 3083 94 uid58415
Shigella boydii Sb227 uid58215
Escherichia coli O55 H7 CB9615 uid46655
Escherichia coli O55 H7 RM12579 uid162153
Escherichia coli O157 H7 EC4115 uid59091
Escherichia coli O157 H7 TW14359 uid59235
Escherichia coli O157 H7 EDL933 uid57831
Escherichia coli O157 H7 Sakai uid57781
Escherichia coli Xuzhou21 uid163995
valS
