# Supplementary material for: Marker genes that are less conserved in their sequences are useful for predicting genome-wide similarity levels between closely related prokaryotic strains
Source: Microbiome. 2016 May 3;4:18. doi: 10.1186/s40168-016-0162-5 (PMC4853863; doi:10.1186/s40168-016-0162-5)
Supplement: Additional file 1: Figure S1. — The number of genomes in which each marker gene is identified. Out of the 79 potential marker genes, 73 are present in at least 90 % of the genomes. Figure S2. Spearman’s correlation between each marker gene and the average AAI for all complete genomes. Genes are ordered in the same way as in Fig. 3. Figure S3. Trees generated based on AAI and on percent identities of each marker gene (including 16s rRNA), for the Escherichia/Shigella clade. Figure S4. Trees generated based on AAI and on percent identities of each marker gene (including 16s rRNA), for the Streptococcus clade. Figure S5. Trees generated based on AAI and on percent identities of each marker gene (including 16s rRNA), for the Bacillus clade. Table S1. List of 79 potential marker genes surveyed, out of which 73 were found to be present in at least 90 % of the genomes. Table S2. Alternative names of 79 potential marker genes surveyed. Table S3. Split distances between UPGMA tree generated using AAI and that generated using the percent identities of each marker gene, shown in correspondence with the average percent identity ranks of the marker genes. Table S4. Designed primers for each of the 10 genes that were least conserved in their sequences in the Escherichia/Shigella lineage. (ZIP 3355 kb) [file 40168_2016_162_MOESM1_ESM.zip › FigureS4.pdf]

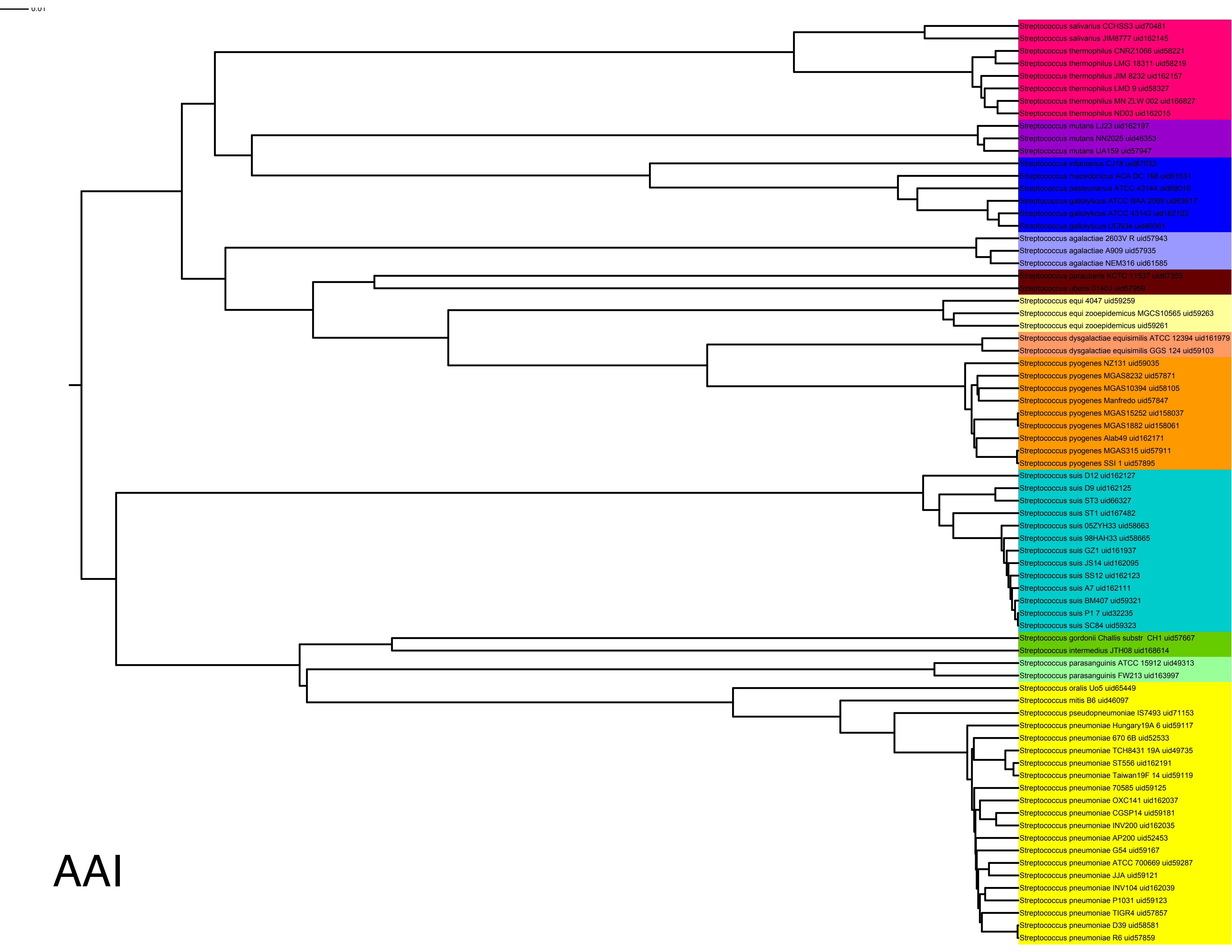
0.01
Streptococcus salivarius CCHSS3 uid70481
Streptococcus salivarius JIM8777 uid162145
Streptococcus thermophilus CNRZ1066 uid58221
Streptococcus thermophilus LMG 18311 uid58219
Streptococcus thermophilus JIM 8232 uid162157
Streptococcus thermophilus LMD 9 uid58327
Streptococcus thermophilus MN ZLW 002 uid166827
Streptococcus thermophilus ND03 uid162015
Streptococcus mutans LJ23 uid162197
Streptococcus mutans NN2025 uid46353
Streptococcus mutans UA159 uid57947
Streptococcus infantarius CJ18 uid87033
Streptococcus macedonicus ACA DC 198 uid81631
Streptococcus pasteurianus ATCC 43144 uid68019
Streptococcus gallolyticus ATCC BAA 2069 uid63617
Streptococcus gallolyticus ATCC 43143 uid162103
Streptococcus gallolyticus UCN34 uid46061
Streptococcus agalactiae 2603V R uid57943
Streptococcus agalactiae A909 uid57935
Streptococcus agalactiae NEM316 uid61585
Streptococcus parauberis KCTC 11537 uid67355
Streptococcus uberis 0140J uid57959
Streptococcus equi 4047 uid59259
Streptococcus equi zooepidemicus MGCS10565 uid59263
Streptococcus equi zooepidemicus uid59261
Streptococcus dysgalactiae equisimilis ATCC 12394 uid161979
Streptococcus dysgalactiae equisimilis GGS 124 uid59103
Streptococcus pyogenes NZ131 uid59035
Streptococcus pyogenes MGAS8232 uid57871
Streptococcus pyogenes MGAS10394 uid58105
Streptococcus pyogenes Manfredo uid57847
Streptococcus pyogenes MGAS15252 uid158037
Streptococcus pyogenes MGAS1882 uid158061
Streptococcus pyogenes Alab49 uid162171
Streptococcus pyogenes MGAS315 uid57911
Streptococcus pyogenes SSI 1 uid57895
Streptococcus suis D12 uid162127
Streptococcus suis D9 uid162125
Streptococcus suis ST3 uid66327
Streptococcus suis ST1 uid167482
Streptococcus suis 05ZYH33 uid58663
Streptococcus suis 98HAH33 uid58665
Streptococcus suis GZ1 uid161937
Streptococcus suis JS14 uid162095
Streptococcus suis SS12 uid162123
Streptococcus suis A7 uid162111
Streptococcus suis BM407 uid59321
Streptococcus suis P1 7 uid32235
Streptococcus suis SC84 uid59323
Streptococcus gordonii Challis substr CH1 uid57667
Streptococcus intermedius JTH08 uid168614
Streptococcus parasanguinis ATCC 15912 uid49313
Streptococcus parasanguinis FW213 uid163997
Streptococcus oralis Uo5 uid65449
Streptococcus mitis B6 uid46097
Streptococcus pseudopneumoniae IS7493 uid71153
Streptococcus pneumoniae Hungary19A 6 uid59117
Streptococcus pneumoniae 670 6B uid52533
Streptococcus pneumoniae TCH8431 19A uid49735
Streptococcus pneumoniae ST556 uid162191
Streptococcus pneumoniae Taiwan19F 14 uid59119
Streptococcus pneumoniae 70585 uid59125
Streptococcus pneumoniae OXC141 uid162037
Streptococcus pneumoniae CGSP14 uid59181
Streptococcus pneumoniae INV200 uid162035
Streptococcus pneumoniae AP200 uid52453
Streptococcus pneumoniae G54 uid59167
Streptococcus pneumoniae ATCC 700669 uid59287
Streptococcus pneumoniae JJA uid59121
Streptococcus pneumoniae INV104 uid162039
Streptococcus pneumoniae P1031 uid59123
Streptococcus pneumoniae TIGR4 uid57857
Streptococcus pneumoniae D39 uid58581
Streptococcus pneumoniae R6 uid57859
AAI

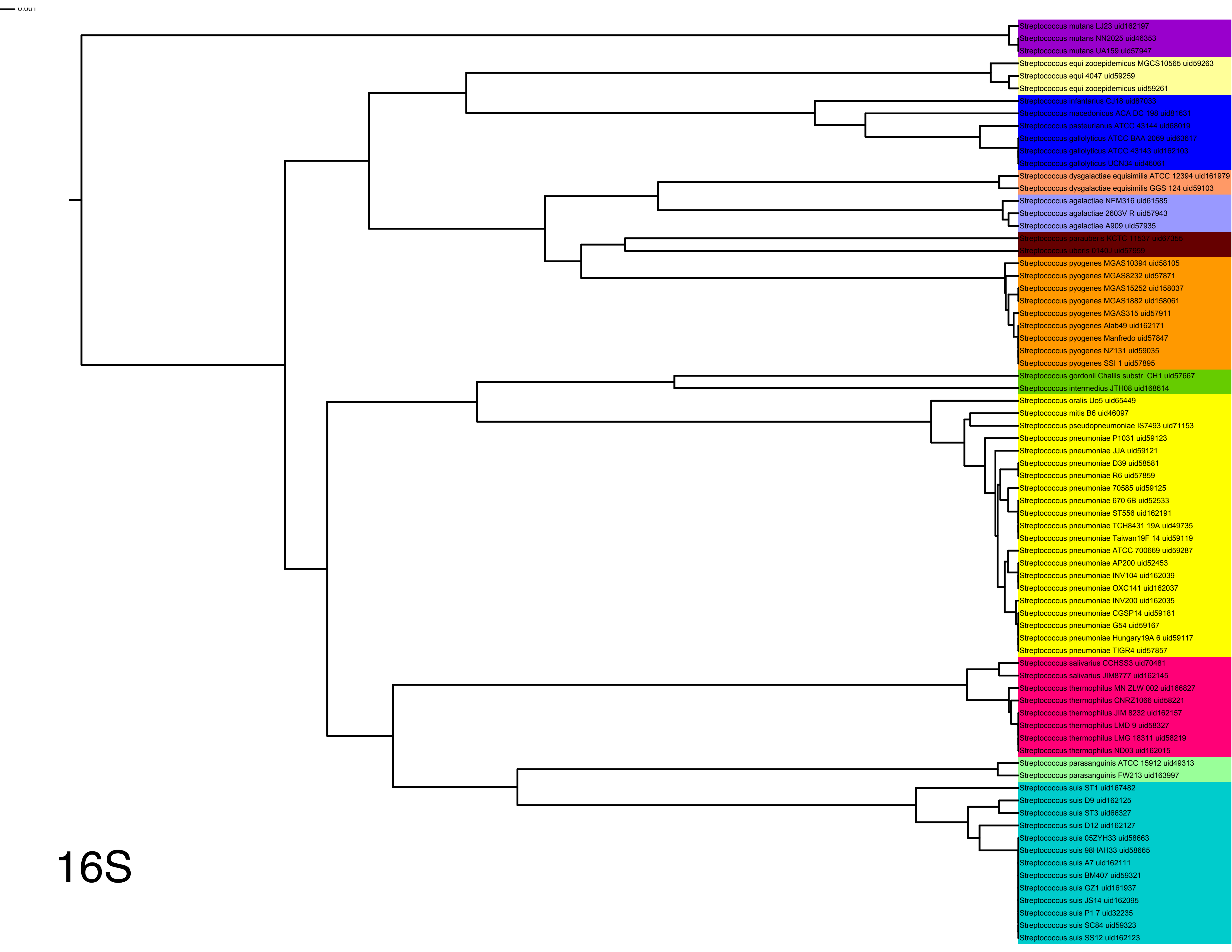
0.001
Streptococcus mutans LJ23 uid162197
Streptococcus mutans NN2025 uid46353
Streptococcus mutans UA159 uid57947
Streptococcus equi zooepidemicus MGCS10565 uid59263
Streptococcus equi 4047 uid59259
Streptococcus equi zooepidemicus uid59261
Streptococcus infantarius CJ18 uid87033
Streptococcus macedonicus ACA DC 198 uid81631
Streptococcus pasteurianus ATCC 43144 uid68019
Streptococcus gallolyticus ATCC BAA 2069 uid63617
Streptococcus gallolyticus ATCC 43143 uid162103
Streptococcus gallolyticus UCN34 uid46061
Streptococcus dysgalactiae equisimilis ATCC 12394 uid161979
Streptococcus dysgalactiae equisimilis GGS 124 uid59103
Streptococcus agalactiae NEM316 uid61585
Streptococcus agalactiae 2603V R uid57943
Streptococcus agalactiae A909 uid57935
Streptococcus parauberis KCTC 11537 uid67355
Streptococcus uberis 0140J uid57959
Streptococcus pyogenes MGAS10394 uid58105
Streptococcus pyogenes MGAS8232 uid57871
Streptococcus pyogenes MGAS15252 uid158037
Streptococcus pyogenes MGAS1882 uid158061
Streptococcus pyogenes MGAS315 uid57911
Streptococcus pyogenes Alab49 uid162171
Streptococcus pyogenes Manfredo uid57847
Streptococcus pyogenes NZ131 uid59035
Streptococcus pyogenes SSI 1 uid57895
Streptococcus gordonii Challis substr CH1 uid57667
Streptococcus intermedius JTH08 uid168614
Streptococcus oralis Uo5 uid65449
Streptococcus mitis B6 uid46097
Streptococcus pseudopneumoniae IS7493 uid71153
Streptococcus pneumoniae P1031 uid59123
Streptococcus pneumoniae JJA uid59121
Streptococcus pneumoniae D39 uid58581
Streptococcus pneumoniae R6 uid57859
Streptococcus pneumoniae 70585 uid59125
Streptococcus pneumoniae 670 6B uid52533
Streptococcus pneumoniae ST556 uid162191
Streptococcus pneumoniae TCH8431 19A uid49735
Streptococcus pneumoniae Taiwan19F 14 uid59119
Streptococcus pneumoniae ATCC 700669 uid59287
Streptococcus pneumoniae AP200 uid52453
Streptococcus pneumoniae INV104 uid162039
Streptococcus pneumoniae OXC141 uid162037
Streptococcus pneumoniae INV200 uid162035
Streptococcus pneumoniae CGSP14 uid59181
Streptococcus pneumoniae G54 uid59167
Streptococcus pneumoniae Hungary19A 6 uid59117
Streptococcus pneumoniae TIGR4 uid57857
Streptococcus salivarius CCHSS3 uid70481
Streptococcus salivarius JIM8777 uid162145
Streptococcus thermophilus MN ZLW 002 uid166827
Streptococcus thermophilus CNRZ1066 uid58221
Streptococcus thermophilus JIM 8232 uid162157
Streptococcus thermophilus LMD 9 uid58327
Streptococcus thermophilus LMG 18311 uid58219
Streptococcus thermophilus ND03 uid162015
Streptococcus parasanguinis ATCC 15912 uid49313
Streptococcus parasanguinis FW213 uid163997
Streptococcus suis ST1 uid167482
Streptococcus suis D9 uid162125
Streptococcus suis ST3 uid66327
Streptococcus suis D12 uid162127
Streptococcus suis 05ZYH33 uid58663
Streptococcus suis 98HAH33 uid58665
Streptococcus suis A7 uid162111
Streptococcus suis BM407 uid59321
Streptococcus suis GZ1 uid161937
Streptococcus suis JS14 uid162095
Streptococcus suis P1 7 uid32235
Streptococcus suis SC84 uid59323
Streptococcus suis SS12 uid162123
16S

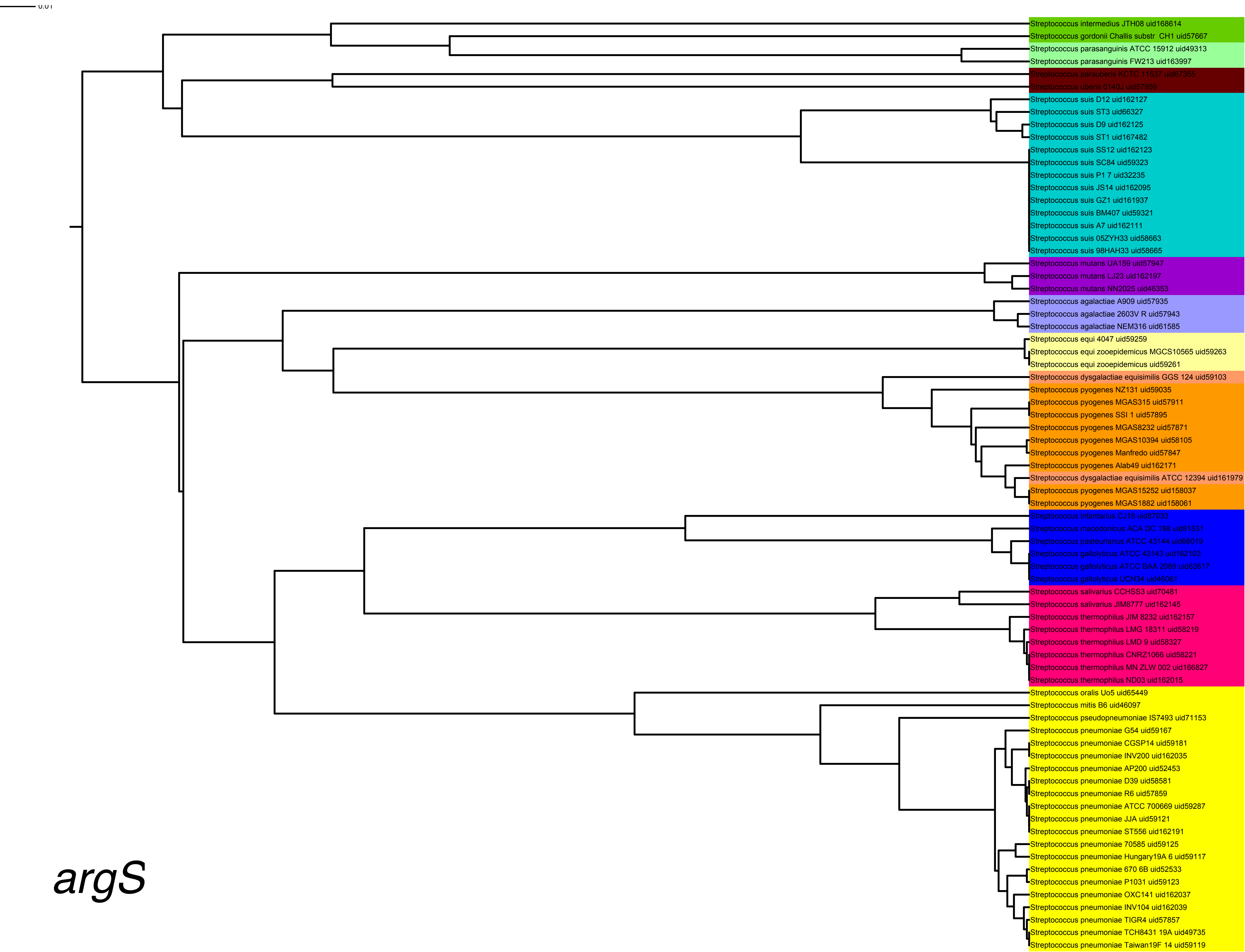
0.01
Streptococcus intermedius JTH08 uid168614
Streptococcus gordonii Challis substr CH1 uid57667
Streptococcus parasanguinis ATCC 15912 uid49313
Streptococcus parasanguinis FW213 uid163997
Streptococcus parauberis KCTC 11537 uid67355
Streptococcus uberis 0140J uid57959
Streptococcus suis D12 uid162127
Streptococcus suis ST3 uid66327
Streptococcus suis D9 uid162125
Streptococcus suis ST1 uid167482
Streptococcus suis SS12 uid162123
Streptococcus suis SC84 uid59323
Streptococcus suis P1 7 uid32235
Streptococcus suis JS14 uid162095
Streptococcus suis GZ1 uid161937
Streptococcus suis BM407 uid59321
Streptococcus suis A7 uid162111
Streptococcus suis 05ZYH33 uid58663
Streptococcus suis 98HAH33 uid58665
Streptococcus mutans UA159 uid57947
Streptococcus mutans LJ23 uid162197
Streptococcus mutans NN2025 uid46353
Streptococcus agalactiae A909 uid57935
Streptococcus agalactiae 2603V R uid57943
Streptococcus agalactiae NEM316 uid61585
Streptococcus equi 4047 uid59259
Streptococcus equi zooepidemicus MGCS10565 uid59263
Streptococcus equi zooepidemicus uid59261
Streptococcus dysgalactiae equisimilis GGS 124 uid59103
Streptococcus pyogenes NZ131 uid59035
Streptococcus pyogenes MGAS315 uid57911
Streptococcus pyogenes SSI 1 uid57895
Streptococcus pyogenes MGAS8232 uid57871
Streptococcus pyogenes MGAS10394 uid58105
Streptococcus pyogenes Manfredo uid57847
Streptococcus pyogenes Alab49 uid162171
Streptococcus dysgalactiae equisimilis ATCC 12394 uid161979
Streptococcus pyogenes MGAS15252 uid158037
Streptococcus pyogenes MGAS1882 uid158061
Streptococcus infantarius CJ18 uid87033
Streptococcus macedonicus ACA DC 198 uid81631
Streptococcus pasteurianus ATCC 43144 uid68019
Streptococcus gallolyticus ATCC 43143 uid162103
Streptococcus gallolyticus ATCC BAA 2069 uid63617
Streptococcus gallolyticus UCN34 uid46061
Streptococcus salivarius CCHSS3 uid70481
Streptococcus salivarius JIM8777 uid162145
Streptococcus thermophilus JIM 8232 uid162157
Streptococcus thermophilus LMG 18311 uid58219
Streptococcus thermophilus LMD 9 uid58327
Streptococcus thermophilus CNRZ1066 uid58221
Streptococcus thermophilus MN ZLW 002 uid166827
Streptococcus thermophilus ND03 uid162015
Streptococcus oralis Uo5 uid65449
Streptococcus mitis B6 uid46097
Streptococcus pseudopneumoniae IS7493 uid71153
Streptococcus pneumoniae G54 uid59167
Streptococcus pneumoniae CGSP14 uid59181
Streptococcus pneumoniae INV200 uid162035
Streptococcus pneumoniae AP200 uid52453
Streptococcus pneumoniae D39 uid58581
Streptococcus pneumoniae R6 uid57859
Streptococcus pneumoniae ATCC 700669 uid59287
Streptococcus pneumoniae JJA uid59121
Streptococcus pneumoniae ST556 uid162191
Streptococcus pneumoniae 70585 uid59125
Streptococcus pneumoniae Hungary19A 6 uid59117
Streptococcus pneumoniae 670 6B uid52533
Streptococcus pneumoniae P1031 uid59123
Streptococcus pneumoniae OXC141 uid162037
Streptococcus pneumoniae INV104 uid162039
Streptococcus pneumoniae TIGR4 uid57857
Streptococcus pneumoniae TCH8431 19A uid49735
Streptococcus pneumoniae Taiwan19F 14 uid59119
argS

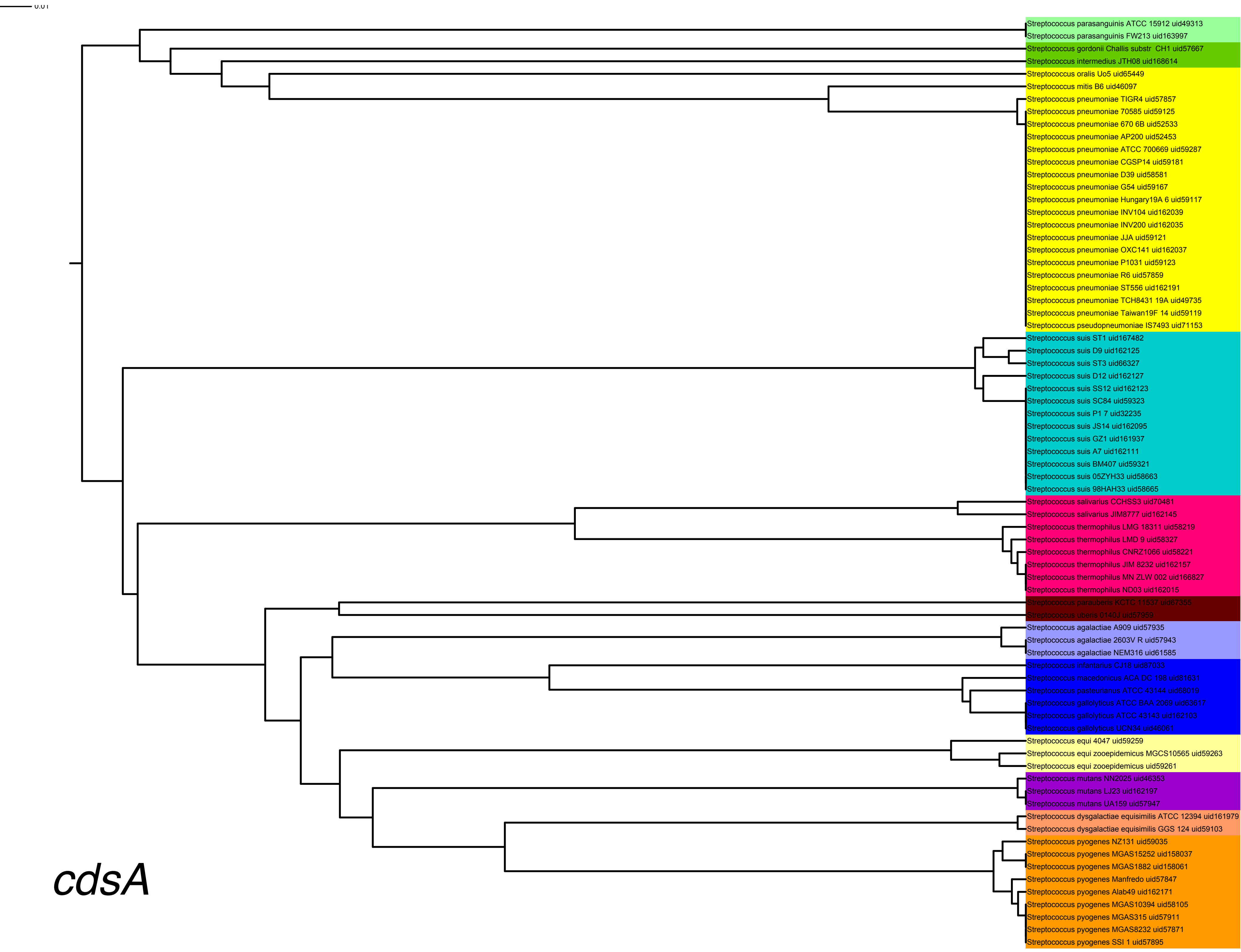
0.01
Streptococcus parasanguinis ATCC 15912 uid49313
Streptococcus parasanguinis FW213 uid163997
Streptococcus gordonii Challis substr CH1 uid57667
Streptococcus intermedius JTH08 uid168614
Streptococcus oralis Uo5 uid65449
Streptococcus mitis B6 uid46097
Streptococcus pneumoniae TIGR4 uid57857
Streptococcus pneumoniae 70585 uid59125
Streptococcus pneumoniae 670 6B uid52533
Streptococcus pneumoniae AP200 uid52453
Streptococcus pneumoniae ATCC 700669 uid59287
Streptococcus pneumoniae CGSP14 uid59181
Streptococcus pneumoniae D39 uid58581
Streptococcus pneumoniae G54 uid59167
Streptococcus pneumoniae Hungary19A 6 uid59117
Streptococcus pneumoniae INV104 uid162039
Streptococcus pneumoniae INV200 uid162035
Streptococcus pneumoniae JJA uid59121
Streptococcus pneumoniae OXC141 uid162037
Streptococcus pneumoniae P1031 uid59123
Streptococcus pneumoniae R6 uid57859
Streptococcus pneumoniae ST556 uid162191
Streptococcus pneumoniae TCH8431 19A uid49735
Streptococcus pneumoniae Taiwan19F 14 uid59119
Streptococcus pseudopneumoniae IS7493 uid71153
Streptococcus suis ST1 uid167482
Streptococcus suis D9 uid162125
Streptococcus suis ST3 uid66327
Streptococcus suis D12 uid162127
Streptococcus suis SS12 uid162123
Streptococcus suis SC84 uid59323
Streptococcus suis P1 7 uid32235
Streptococcus suis JS14 uid162095
Streptococcus suis GZ1 uid161937
Streptococcus suis A7 uid162111
Streptococcus suis BM407 uid59321
Streptococcus suis 05ZYH33 uid58663
Streptococcus suis 98HAH33 uid58665
Streptococcus salivarius CCHSS3 uid70481
Streptococcus salivarius JIM8777 uid162145
Streptococcus thermophilus LMG 18311 uid58219
Streptococcus thermophilus LMD 9 uid58327
Streptococcus thermophilus CNRZ1066 uid58221
Streptococcus thermophilus JIM 8232 uid162157
Streptococcus thermophilus MN ZLW 002 uid166827
Streptococcus thermophilus ND03 uid162015
Streptococcus parauberis KCTC 11537 uid67355
Streptococcus uberis 0140J uid57959
Streptococcus agalactiae A909 uid57935
Streptococcus agalactiae 2603V R uid57943
Streptococcus agalactiae NEM316 uid61585
Streptococcus infantarius CJ18 uid87033
Streptococcus macedonicus ACA DC 198 uid81631
Streptococcus pasteurianus ATCC 43144 uid68019
Streptococcus gallolyticus ATCC BAA 2069 uid63617
Streptococcus gallolyticus ATCC 43143 uid162103
Streptococcus gallolyticus UCN34 uid46061
Streptococcus equi 4047 uid59259
Streptococcus equi zooepidemicus MGCS10565 uid59263
Streptococcus equi zooepidemicus uid59261
Streptococcus mutans NN2025 uid46353
Streptococcus mutans LJ23 uid162197
Streptococcus mutans UA159 uid57947
Streptococcus dysgalactiae equisimilis ATCC 12394 uid161979
Streptococcus dysgalactiae equisimilis GGS 124 uid59103
Streptococcus pyogenes NZ131 uid59035
Streptococcus pyogenes MGAS15252 uid158037
Streptococcus pyogenes MGAS1882 uid158061
Streptococcus pyogenes Manfredo uid57847
Streptococcus pyogenes Alab49 uid162171
Streptococcus pyogenes MGAS10394 uid58105
Streptococcus pyogenes MGAS315 uid57911
Streptococcus pyogenes MGAS8232 uid57871
Streptococcus pyogenes SSI 1 uid57895
cdsA

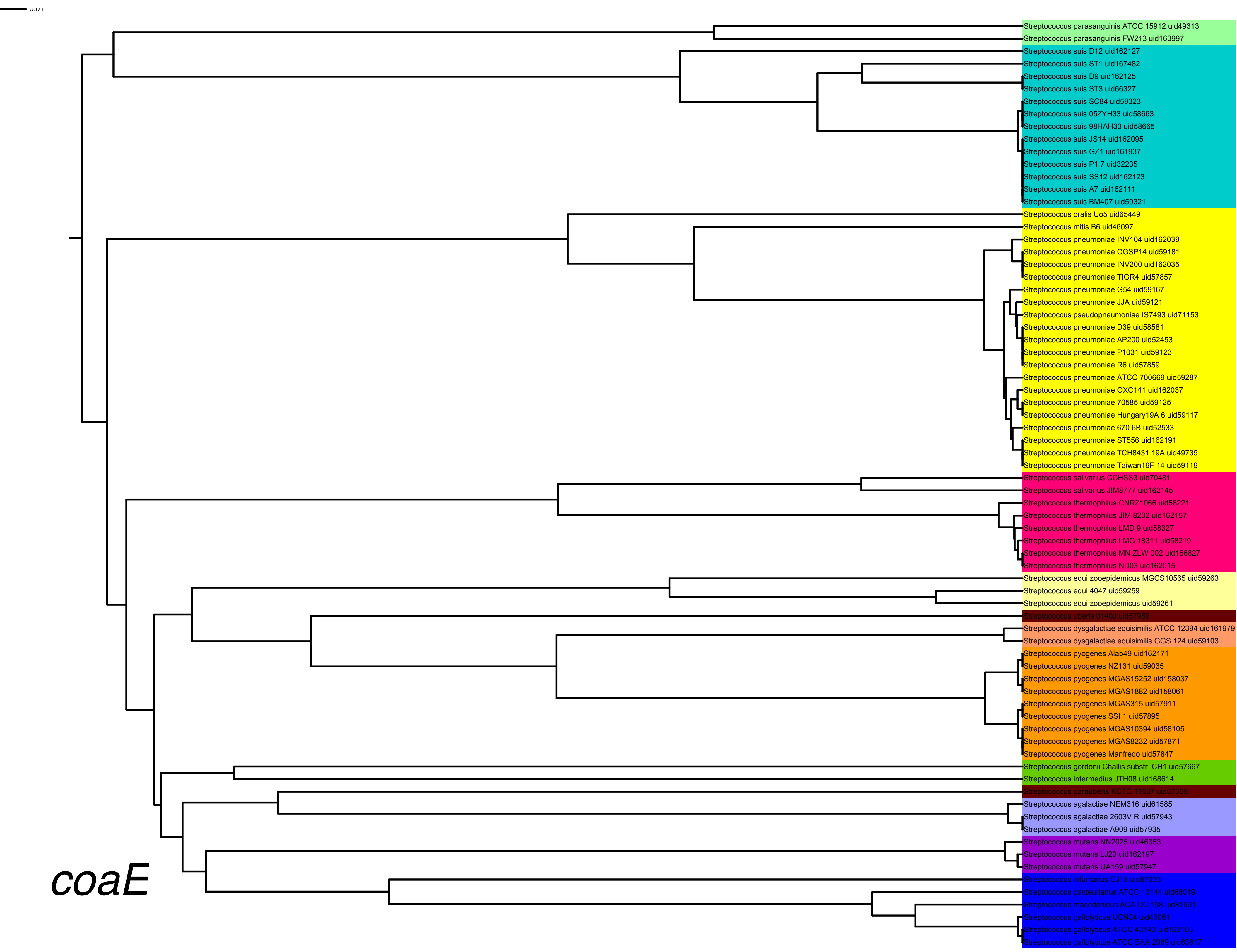
0.01
Streptococcus parasanguinis ATCC 15912 uid49313
Streptococcus parasanguinis FW213 uid163997
Streptococcus suis D12 uid162127
Streptococcus suis ST1 uid167482
Streptococcus suis D9 uid162125
Streptococcus suis ST3 uid66327
Streptococcus suis SC84 uid59323
Streptococcus suis 05ZYH33 uid58663
Streptococcus suis 98HAH33 uid58665
Streptococcus suis JS14 uid162095
Streptococcus suis GZ1 uid161937
Streptococcus suis P1 7 uid32235
Streptococcus suis SS12 uid162123
Streptococcus suis A7 uid162111
Streptococcus suis BM407 uid59321
Streptococcus oralis Uo5 uid65449
Streptococcus mitis B6 uid46097
Streptococcus pneumoniae INV104 uid162039
Streptococcus pneumoniae CGSP14 uid59181
Streptococcus pneumoniae INV200 uid162035
Streptococcus pneumoniae TIGR4 uid57857
Streptococcus pneumoniae G54 uid59167
Streptococcus pneumoniae JJA uid59121
Streptococcus pseudopneumoniae IS7493 uid71153
Streptococcus pneumoniae D39 uid58581
Streptococcus pneumoniae AP200 uid52453
Streptococcus pneumoniae P1031 uid59123
Streptococcus pneumoniae R6 uid57859
Streptococcus pneumoniae ATCC 700669 uid59287
Streptococcus pneumoniae OXC141 uid162037
Streptococcus pneumoniae 70585 uid59125
Streptococcus pneumoniae Hungary19A 6 uid59117
Streptococcus pneumoniae 670 6B uid52533
Streptococcus pneumoniae ST556 uid162191
Streptococcus pneumoniae TCH8431 19A uid49735
Streptococcus pneumoniae Taiwan19F 14 uid59119
Streptococcus salivarius CCHSS3 uid70481
Streptococcus salivarius JIM8777 uid162145
Streptococcus thermophilus CNRZ1066 uid58221
Streptococcus thermophilus JIM 8232 uid162157
Streptococcus thermophilus LMD 9 uid58327
Streptococcus thermophilus LMG 18311 uid58219
Streptococcus thermophilus MN ZLW 002 uid166827
Streptococcus thermophilus ND03 uid162015
Streptococcus equi zooepidemicus MGCS10565 uid59263
Streptococcus equi 4047 uid59259
Streptococcus equi zooepidemicus uid59261
Streptococcus uberis 0140J uid57959
Streptococcus dysgalactiae equisimilis ATCC 12394 uid161979
Streptococcus dysgalactiae equisimilis GGS 124 uid59103
Streptococcus pyogenes Alab49 uid162171
Streptococcus pyogenes NZ131 uid59035
Streptococcus pyogenes MGAS15252 uid158037
Streptococcus pyogenes MGAS1882 uid158061
Streptococcus pyogenes MGAS315 uid57911
Streptococcus pyogenes SSI 1 uid57895
Streptococcus pyogenes MGAS10394 uid58105
Streptococcus pyogenes MGAS8232 uid57871
Streptococcus pyogenes Manfredo uid57847
Streptococcus gordonii Challis substr CH1 uid57667
Streptococcus intermedius JTH08 uid168614
Streptococcus parauberis KCTC 11537 uid67355
Streptococcus agalactiae NEM316 uid61585
Streptococcus agalactiae 2603V R uid57943
Streptococcus agalactiae A909 uid57935
Streptococcus mutans NN2025 uid46353
Streptococcus mutans LJ23 uid162197
Streptococcus mutans UA159 uid57947
Streptococcus infantarius CJ18 uid87033
Streptococcus pasteurianus ATCC 43144 uid68019
Streptococcus macedonicus ACA DC 198 uid81631
Streptococcus gallolyticus UCN34 uid46061
Streptococcus gallolyticus ATCC 43143 uid162103
Streptococcus gallolyticus ATCC BAA 2069 uid63617
coaE

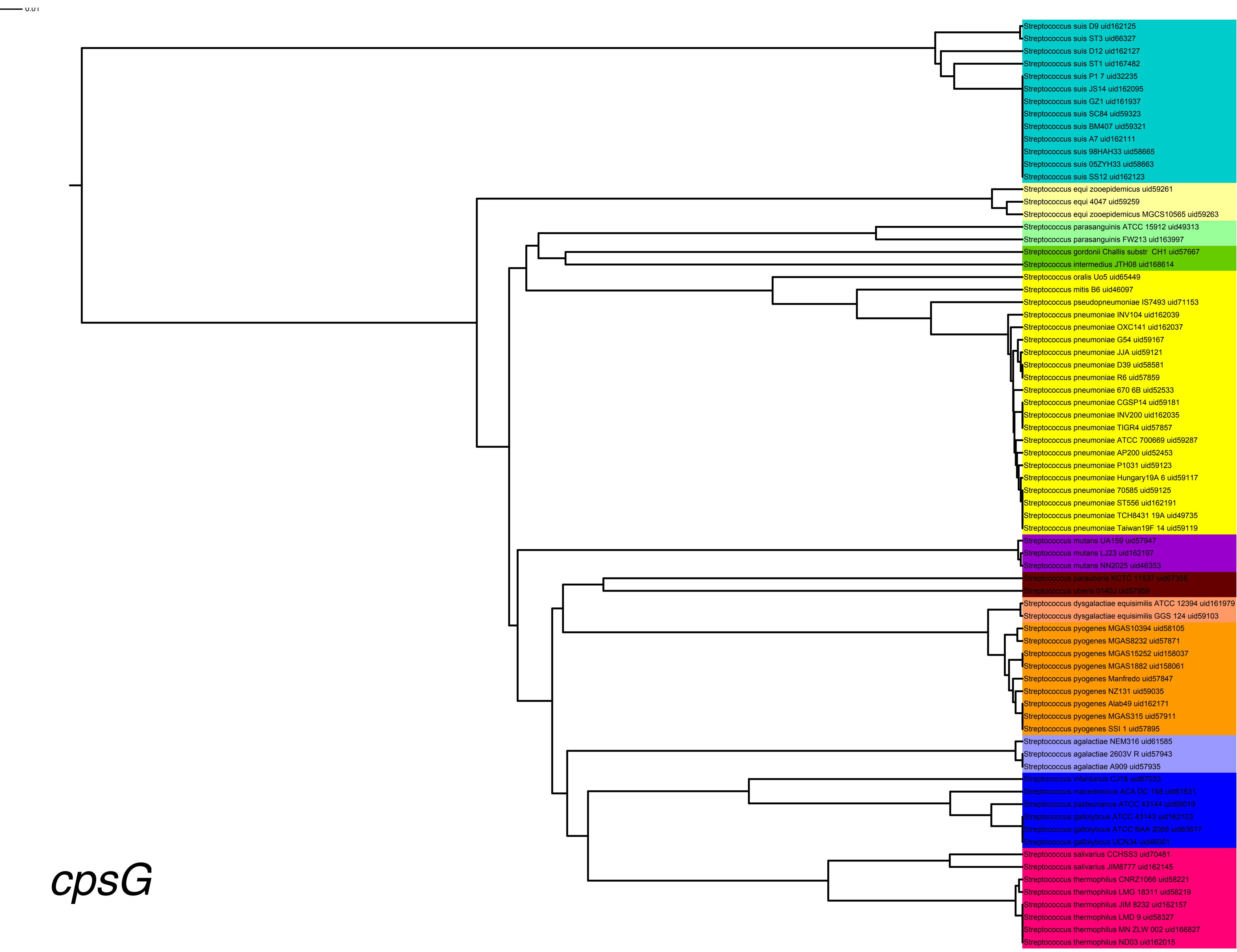

0.01
Streptococcus suis D9 uid162125
Streptococcus suis ST3 uid66327
Streptococcus suis D12 uid162127
Streptococcus suis ST1 uid167482
Streptococcus suis P1 7 uid32235
Streptococcus suis JS14 uid162095
Streptococcus suis GZ1 uid161937
Streptococcus suis SC84 uid59323
Streptococcus suis BM407 uid59321
Streptococcus suis A7 uid162111
Streptococcus suis 98HAH33 uid58665
Streptococcus suis 05ZYH33 uid58663
Streptococcus suis SS12 uid162123
Streptococcus equi zooepidemicus uid59261
Streptococcus equi 4047 uid59259
Streptococcus equi zooepidemicus MGCS10565 uid59263
Streptococcus parasanguinis ATCC 15912 uid49313
Streptococcus parasanguinis FW213 uid163997
Streptococcus gordonii Challis substr CH1 uid57667
Streptococcus intermedius JTH08 uid168614
Streptococcus oralis Uo5 uid65449
Streptococcus mitis B6 uid46097
Streptococcus pseudopneumoniae IS7493 uid71153
Streptococcus pneumoniae INV104 uid162039
Streptococcus pneumoniae OXC141 uid162037
Streptococcus pneumoniae G54 uid59167
Streptococcus pneumoniae JJA uid59121
Streptococcus pneumoniae D39 uid58581
Streptococcus pneumoniae R6 uid57859
Streptococcus pneumoniae 670 6B uid52533
Streptococcus pneumoniae CGSP14 uid59181
Streptococcus pneumoniae INV200 uid162035
Streptococcus pneumoniae TIGR4 uid57857
Streptococcus pneumoniae ATCC 700669 uid59287
Streptococcus pneumoniae AP200 uid52453
Streptococcus pneumoniae P1031 uid59123
Streptococcus pneumoniae Hungary19A 6 uid59117
Streptococcus pneumoniae 70585 uid59125
Streptococcus pneumoniae ST556 uid162191
Streptococcus pneumoniae TCH8431 19A uid49735
Streptococcus pneumoniae Taiwan19F 14 uid59119
Streptococcus mutans UA159 uid57947
Streptococcus mutans LJ23 uid162197
Streptococcus mutans NN2025 uid46353
Streptococcus parauberis KCTC 11537 uid67355
Streptococcus uberis 0140J uid57959
Streptococcus dysgalactiae equisimilis ATCC 12394 uid161979
Streptococcus dysgalactiae equisimilis GGS 124 uid59103
Streptococcus pyogenes MGAS10394 uid58105
Streptococcus pyogenes MGAS8232 uid57871
Streptococcus pyogenes MGAS15252 uid158037
Streptococcus pyogenes MGAS1882 uid158061
Streptococcus pyogenes Manfredo uid57847
Streptococcus pyogenes NZ131 uid59035
Streptococcus pyogenes Alab49 uid162171
Streptococcus pyogenes MGAS315 uid57911
Streptococcus pyogenes SSI 1 uid57895
Streptococcus agalactiae NEM316 uid61585
Streptococcus agalactiae 2603V R uid57943
Streptococcus agalactiae A909 uid57935
Streptococcus infantarius CJ18 uid87033
Streptococcus macedonicus ACA DC 198 uid81631
Streptococcus pasteurianus ATCC 43144 uid68019
Streptococcus gallolyticus ATCC 43143 uid162103
Streptococcus gallolyticus ATCC BAA 2069 uid63617
Streptococcus gallolyticus UCN34 uid46061
Streptococcus salivarius CCHSS3 uid70481
Streptococcus salivarius JIM8777 uid162145
Streptococcus thermophilus CNRZ1066 uid58221
Streptococcus thermophilus LMG 18311 uid58219
Streptococcus thermophilus JIM 8232 uid162157
Streptococcus thermophilus LMD 9 uid58327
Streptococcus thermophilus MN ZLW 002 uid166827
Streptococcus thermophilus ND03 uid162015
cpsG

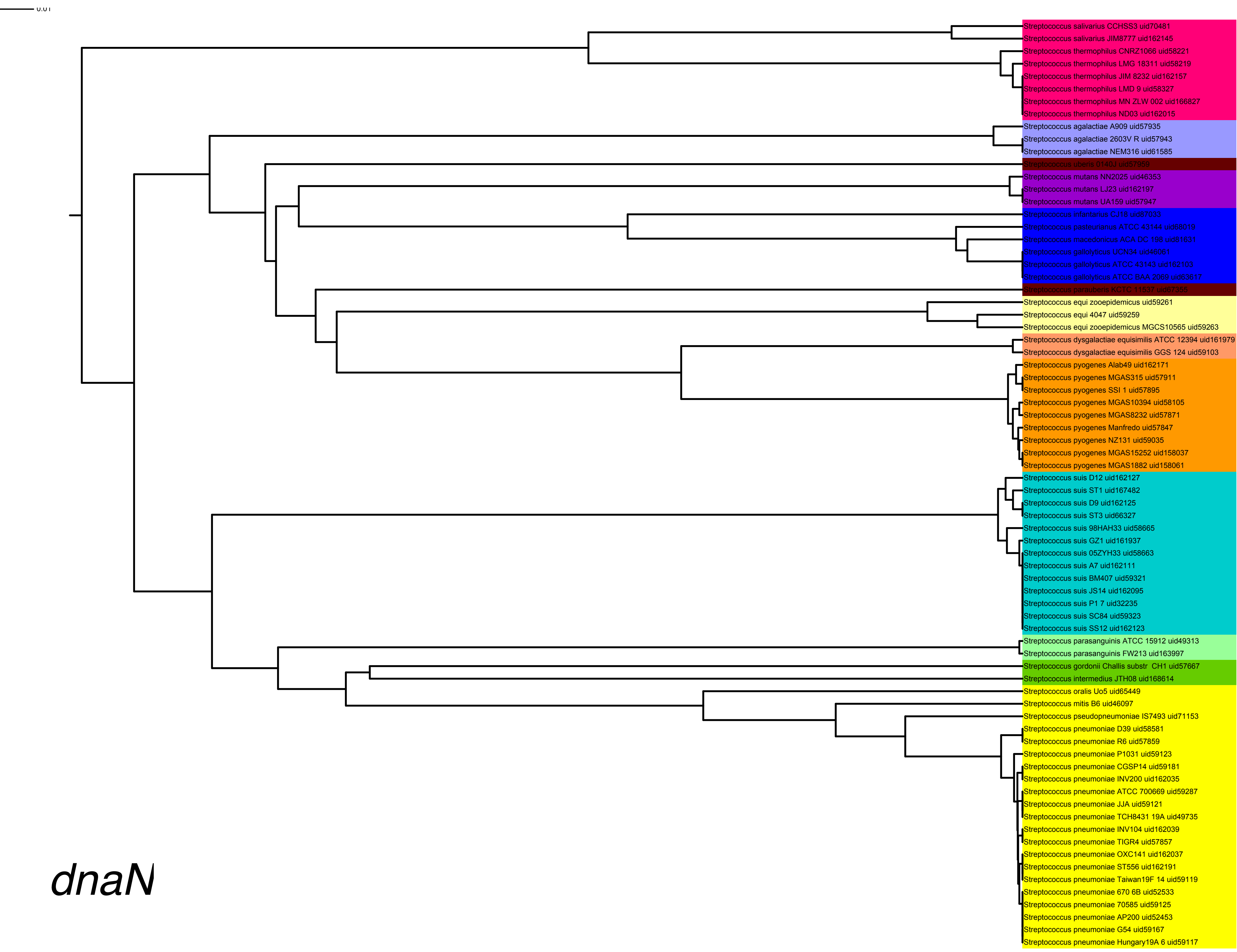
0.01
Streptococcus salivarius CCHSS3 uid70481
Streptococcus salivarius JIM8777 uid162145
Streptococcus thermophilus CNRZ1066 uid58221
Streptococcus thermophilus LMG 18311 uid58219
Streptococcus thermophilus JIM 8232 uid162157
Streptococcus thermophilus LMD 9 uid58327
Streptococcus thermophilus MN ZLW 002 uid166827
Streptococcus thermophilus ND03 uid162015
Streptococcus agalactiae A909 uid57935
Streptococcus agalactiae 2603V R uid57943
Streptococcus agalactiae NEM316 uid61585
Streptococcus uberis 0140J uid57959
Streptococcus mutans NN2025 uid46353
Streptococcus mutans LJ23 uid162197
Streptococcus mutans UA159 uid57947
Streptococcus infantarius CJ18 uid87033
Streptococcus pasteurianus ATCC 43144 uid68019
Streptococcus macedonicus ACA DC 198 uid81631
Streptococcus gallolyticus UCN34 uid46061
Streptococcus gallolyticus ATCC 43143 uid162103
Streptococcus gallolyticus ATCC BAA 2069 uid63617
Streptococcus parauberis KCTC 11537 uid67355
Streptococcus equi zooepidemicus uid59261
Streptococcus equi 4047 uid59259
Streptococcus equi zooepidemicus MGCS10565 uid59263
Streptococcus dysgalactiae equisimilis ATCC 12394 uid161979
Streptococcus dysgalactiae equisimilis GGS 124 uid59103
Streptococcus pyogenes Alab49 uid162171
Streptococcus pyogenes MGAS315 uid57911
Streptococcus pyogenes SSI 1 uid57895
Streptococcus pyogenes MGAS10394 uid58105
Streptococcus pyogenes MGAS8232 uid57871
Streptococcus pyogenes Manfredo uid57847
Streptococcus pyogenes NZ131 uid59035
Streptococcus pyogenes MGAS15252 uid158037
Streptococcus pyogenes MGAS1882 uid158061
Streptococcus suis D12 uid162127
Streptococcus suis ST1 uid167482
Streptococcus suis D9 uid162125
Streptococcus suis ST3 uid66327
Streptococcus suis 98HAH33 uid58665
Streptococcus suis GZ1 uid161937
Streptococcus suis 05ZYH33 uid58663
Streptococcus suis A7 uid162111
Streptococcus suis BM407 uid59321
Streptococcus suis JS14 uid162095
Streptococcus suis P1 7 uid32235
Streptococcus suis SC84 uid59323
Streptococcus suis SS12 uid162123
Streptococcus parasanguinis ATCC 15912 uid49313
Streptococcus parasanguinis FW213 uid163997
Streptococcus gordonii Challis substr CH1 uid57667
Streptococcus intermedius JTH08 uid168614
Streptococcus oralis Uo5 uid65449
Streptococcus mitis B6 uid46097
Streptococcus pseudopneumoniae IS7493 uid71153
Streptococcus pneumoniae D39 uid58581
Streptococcus pneumoniae R6 uid57859
Streptococcus pneumoniae P1031 uid59123
Streptococcus pneumoniae CGSP14 uid59181
Streptococcus pneumoniae INV200 uid162035
Streptococcus pneumoniae ATCC 700669 uid59287
Streptococcus pneumoniae JJA uid59121
Streptococcus pneumoniae TCH8431 19A uid49735
Streptococcus pneumoniae INV104 uid162039
Streptococcus pneumoniae TIGR4 uid57857
Streptococcus pneumoniae OXC141 uid162037
Streptococcus pneumoniae ST556 uid162191
Streptococcus pneumoniae Taiwan19F 14 uid59119
Streptococcus pneumoniae 670 6B uid52533
Streptococcus pneumoniae 70585 uid59125
Streptococcus pneumoniae AP200 uid52453
Streptococcus pneumoniae G54 uid59167
Streptococcus pneumoniae Hungary19A 6 uid59117
dnaN

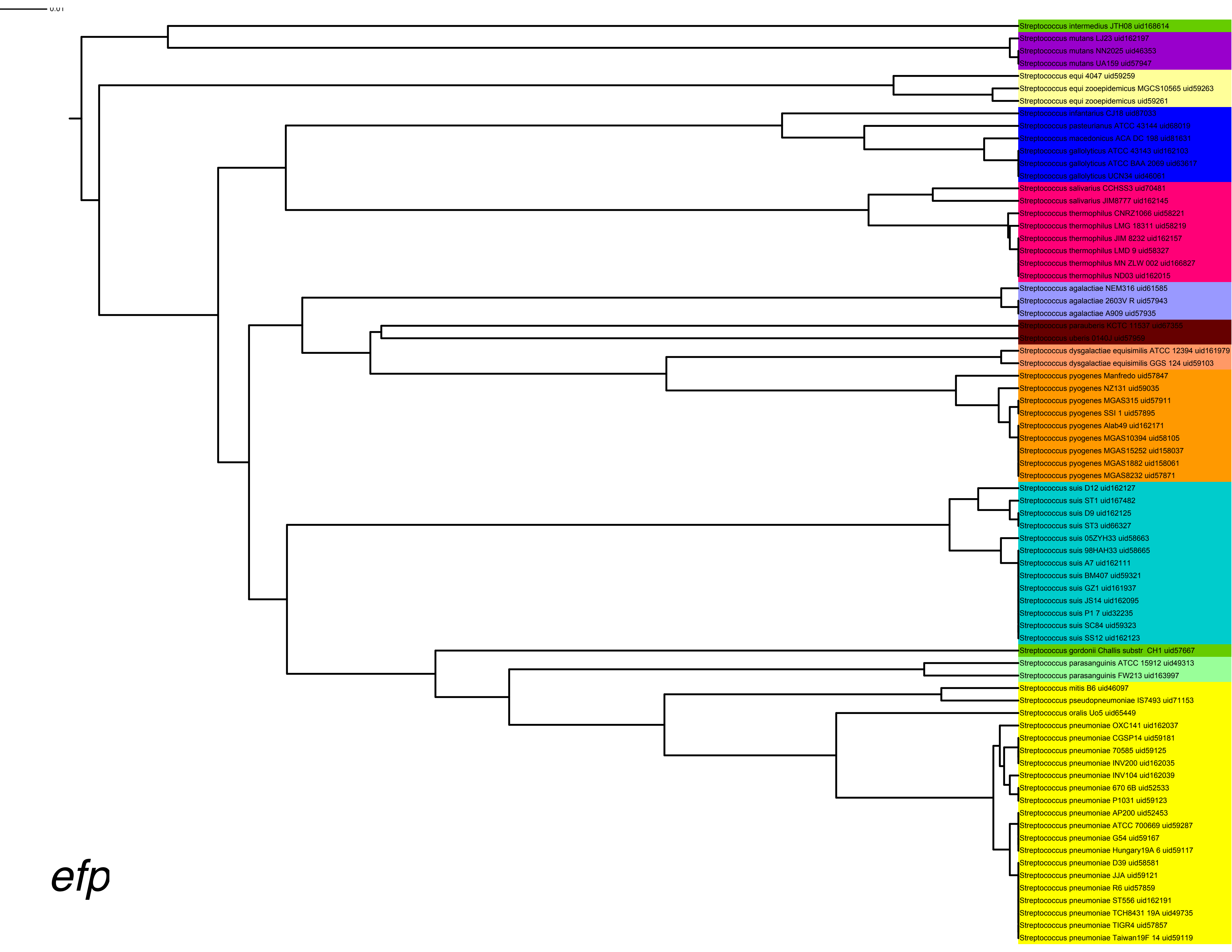
0.01
Streptococcus intermedius JTH08 uid168614
Streptococcus mutans LJ23 uid162197
Streptococcus mutans NN2025 uid46353
Streptococcus mutans UA159 uid57947
Streptococcus equi 4047 uid59259
Streptococcus equi zooepidemicus MGCS10565 uid59263
Streptococcus equi zooepidemicus uid59261
Streptococcus infantarius CJ18 uid87033
Streptococcus pasteurianus ATCC 43144 uid68019
Streptococcus macedonicus ACA DC 198 uid81631
Streptococcus gallolyticus ATCC 43143 uid162103
Streptococcus gallolyticus ATCC BAA 2069 uid63617
Streptococcus gallolyticus UCN34 uid46061
Streptococcus salivarius CCHSS3 uid70481
Streptococcus salivarius JIM8777 uid162145
Streptococcus thermophilus CNRZ1066 uid58221
Streptococcus thermophilus LMG 18311 uid58219
Streptococcus thermophilus JIM 8232 uid162157
Streptococcus thermophilus LMD 9 uid58327
Streptococcus thermophilus MN ZLW 002 uid166827
Streptococcus thermophilus ND03 uid162015
Streptococcus agalactiae NEM316 uid61585
Streptococcus agalactiae 2603V R uid57943
Streptococcus agalactiae A909 uid57935
Streptococcus parauberis KCTC 11537 uid67355
Streptococcus uberis 0140J uid57959
Streptococcus dysgalactiae equisimilis ATCC 12394 uid161979
Streptococcus dysgalactiae equisimilis GGS 124 uid59103
Streptococcus pyogenes Manfredo uid57847
Streptococcus pyogenes NZ131 uid59035
Streptococcus pyogenes MGAS315 uid57911
Streptococcus pyogenes SSI 1 uid57895
Streptococcus pyogenes Alab49 uid162171
Streptococcus pyogenes MGAS10394 uid58105
Streptococcus pyogenes MGAS15252 uid158037
Streptococcus pyogenes MGAS1882 uid158061
Streptococcus pyogenes MGAS8232 uid57871
Streptococcus suis D12 uid162127
Streptococcus suis ST1 uid167482
Streptococcus suis D9 uid162125
Streptococcus suis ST3 uid66327
Streptococcus suis 05ZYH33 uid58663
Streptococcus suis 98HAH33 uid58665
Streptococcus suis A7 uid162111
Streptococcus suis BM407 uid59321
Streptococcus suis GZ1 uid161937
Streptococcus suis JS14 uid162095
Streptococcus suis P1 7 uid32235
Streptococcus suis SC84 uid59323
Streptococcus suis SS12 uid162123
Streptococcus gordonii Challis substr CH1 uid57667
Streptococcus parasanguinis ATCC 15912 uid49313
Streptococcus parasanguinis FW213 uid163997
Streptococcus mitis B6 uid46097
Streptococcus pseudopneumoniae IS7493 uid71153
Streptococcus oralis Uo5 uid65449
Streptococcus pneumoniae OXC141 uid162037
Streptococcus pneumoniae CGSP14 uid59181
Streptococcus pneumoniae 70585 uid59125
Streptococcus pneumoniae INV200 uid162035
Streptococcus pneumoniae INV104 uid162039
Streptococcus pneumoniae 670 6B uid52533
Streptococcus pneumoniae P1031 uid59123
Streptococcus pneumoniae AP200 uid52453
Streptococcus pneumoniae ATCC 700669 uid59287
Streptococcus pneumoniae G54 uid59167
Streptococcus pneumoniae Hungary19A 6 uid59117
Streptococcus pneumoniae D39 uid58581
Streptococcus pneumoniae JJA uid59121
Streptococcus pneumoniae R6 uid57859
Streptococcus pneumoniae ST556 uid162191
Streptococcus pneumoniae TCH8431 19A uid49735
Streptococcus pneumoniae TIGR4 uid57857
Streptococcus pneumoniae Taiwan19F 14 uid59119
efp

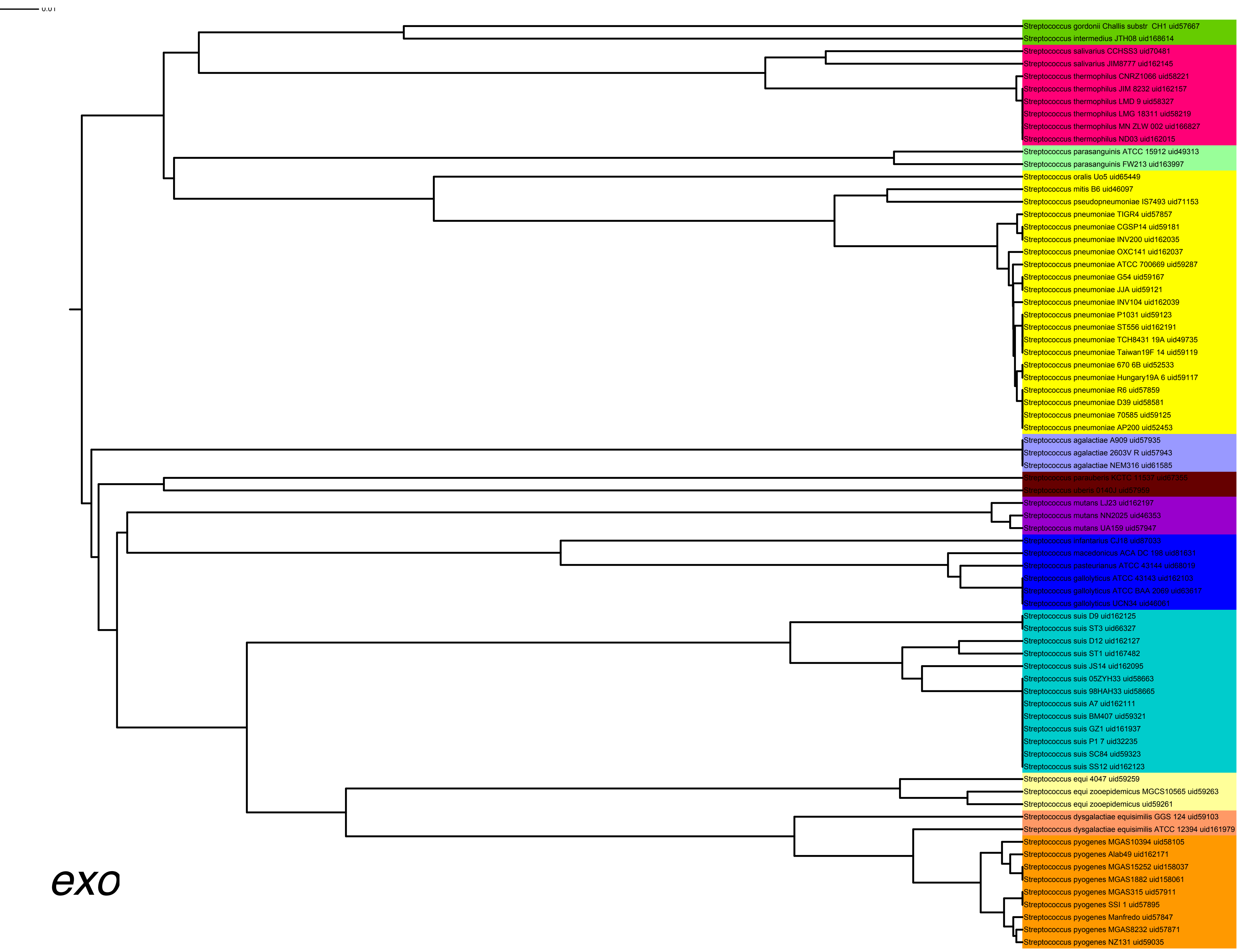

0.01
Streptococcus gordonii Challis substr CH1 uid57667
Streptococcus intermedius JTH08 uid168614
Streptococcus salivarius CCHSS3 uid70481
Streptococcus salivarius JIM8777 uid162145
Streptococcus thermophilus CNRZ1066 uid58221
Streptococcus thermophilus JIM 8232 uid162157
Streptococcus thermophilus LMD 9 uid58327
Streptococcus thermophilus LMG 18311 uid58219
Streptococcus thermophilus MN ZLW 002 uid166827
Streptococcus thermophilus ND03 uid162015
Streptococcus parasanguinis ATCC 15912 uid49313
Streptococcus parasanguinis FW213 uid163997
Streptococcus oralis Uo5 uid65449
Streptococcus mitis B6 uid46097
Streptococcus pseudopneumoniae IS7493 uid71153
Streptococcus pneumoniae TIGR4 uid57857
Streptococcus pneumoniae CGSP14 uid59181
Streptococcus pneumoniae INV200 uid162035
Streptococcus pneumoniae OXC141 uid162037
Streptococcus pneumoniae ATCC 700669 uid59287
Streptococcus pneumoniae G54 uid59167
Streptococcus pneumoniae JJA uid59121
Streptococcus pneumoniae INV104 uid162039
Streptococcus pneumoniae P1031 uid59123
Streptococcus pneumoniae ST556 uid162191
Streptococcus pneumoniae TCH8431 19A uid49735
Streptococcus pneumoniae Taiwan19F 14 uid59119
Streptococcus pneumoniae 670 6B uid52533
Streptococcus pneumoniae Hungary19A 6 uid59117
Streptococcus pneumoniae R6 uid57859
Streptococcus pneumoniae D39 uid58581
Streptococcus pneumoniae 70585 uid59125
Streptococcus pneumoniae AP200 uid52453
Streptococcus agalactiae A909 uid57935
Streptococcus agalactiae 2603V R uid57943
Streptococcus agalactiae NEM316 uid61585
Streptococcus parauberis KCTC 11537 uid67355
Streptococcus uberis 0140J uid57959
Streptococcus mutans LJ23 uid162197
Streptococcus mutans NN2025 uid46353
Streptococcus mutans UA159 uid57947
Streptococcus infantarius CJ18 uid87033
Streptococcus macedonicus ACA DC 198 uid81631
Streptococcus pasteurianus ATCC 43144 uid68019
Streptococcus gallolyticus ATCC 43143 uid162103
Streptococcus gallolyticus ATCC BAA 2069 uid63617
Streptococcus gallolyticus UCN34 uid46061
Streptococcus suis D9 uid162125
Streptococcus suis ST3 uid66327
Streptococcus suis D12 uid162127
Streptococcus suis ST1 uid167482
Streptococcus suis JS14 uid162095
Streptococcus suis 05ZYH33 uid58663
Streptococcus suis 98HAH33 uid58665
Streptococcus suis A7 uid162111
Streptococcus suis BM407 uid59321
Streptococcus suis GZ1 uid161937
Streptococcus suis P1 7 uid32235
Streptococcus suis SC84 uid59323
Streptococcus suis SS12 uid162123
Streptococcus equi 4047 uid59259
Streptococcus equi zooepidemicus MGCS10565 uid59263
Streptococcus equi zooepidemicus uid59261
Streptococcus dysgalactiae equisimilis GGS 124 uid59103
Streptococcus dysgalactiae equisimilis ATCC 12394 uid161979
Streptococcus pyogenes MGAS10394 uid58105
Streptococcus pyogenes Alab49 uid162171
Streptococcus pyogenes MGAS15252 uid158037
Streptococcus pyogenes MGAS1882 uid158061
Streptococcus pyogenes MGAS315 uid57911
Streptococcus pyogenes SSI 1 uid57895
Streptococcus pyogenes Manfredo uid57847
Streptococcus pyogenes MGAS8232 uid57871
Streptococcus pyogenes NZ131 uid59035
exo

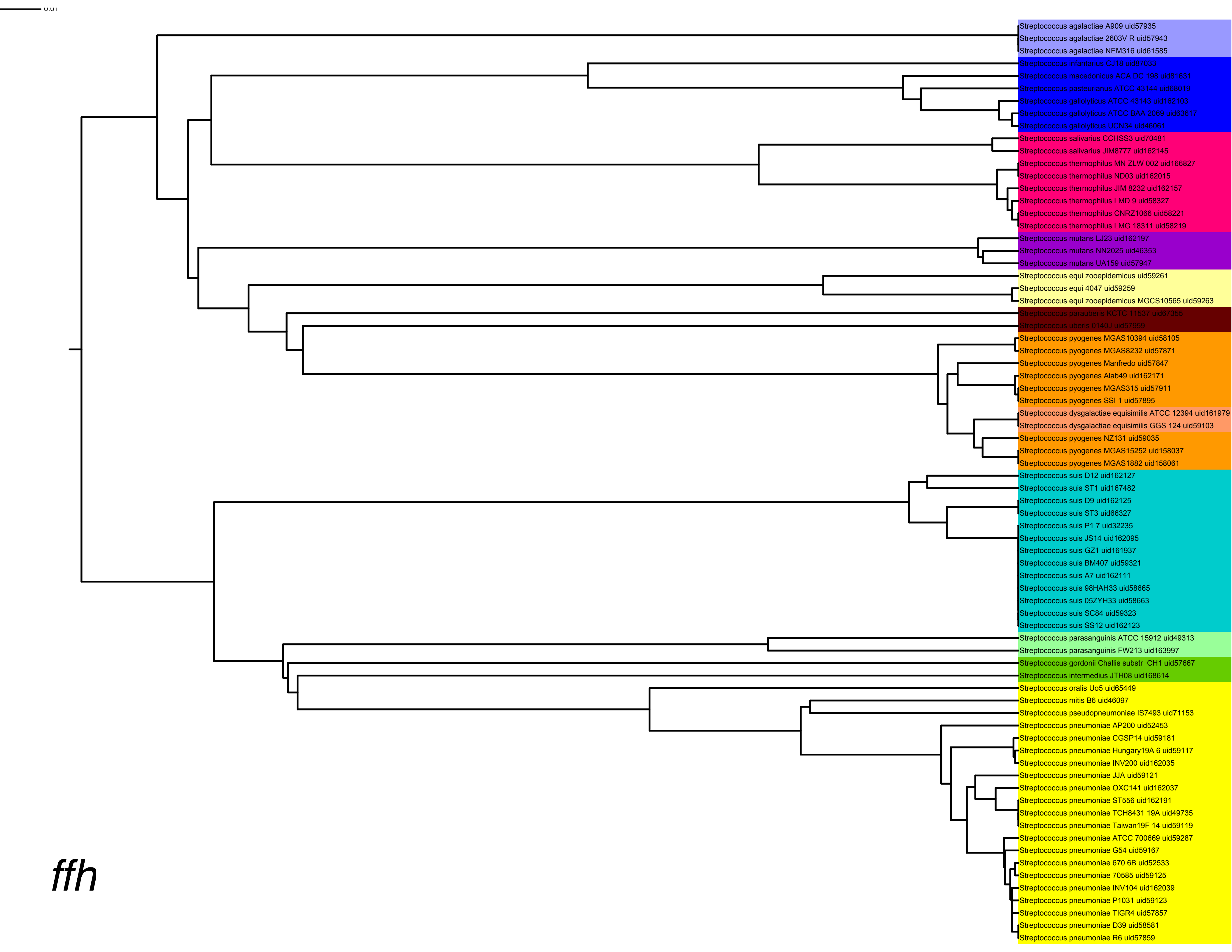
0.01
Streptococcus agalactiae A909 uid57935
Streptococcus agalactiae 2603V R uid57943
Streptococcus agalactiae NEM316 uid61585
Streptococcus infantarius CJ18 uid87033
Streptococcus macedonicus ACA DC 198 uid81631
Streptococcus pasteurianus ATCC 43144 uid68019
Streptococcus gallolyticus ATCC 43143 uid162103
Streptococcus gallolyticus ATCC BAA 2069 uid63617
Streptococcus gallolyticus UCN34 uid46061
Streptococcus salivarius CCHSS3 uid70481
Streptococcus salivarius JIM8777 uid162145
Streptococcus thermophilus MN ZLW 002 uid166827
Streptococcus thermophilus ND03 uid162015
Streptococcus thermophilus JIM 8232 uid162157
Streptococcus thermophilus LMD 9 uid58327
Streptococcus thermophilus CNRZ1066 uid58221
Streptococcus thermophilus LMG 18311 uid58219
Streptococcus mutans LJ23 uid162197
Streptococcus mutans NN2025 uid46353
Streptococcus mutans UA159 uid57947
Streptococcus equi zooepidemicus uid59261
Streptococcus equi 4047 uid59259
Streptococcus equi zooepidemicus MGCS10565 uid59263
Streptococcus parauberis KCTC 11537 uid67355
Streptococcus uberis 0140J uid57959
Streptococcus pyogenes MGAS10394 uid58105
Streptococcus pyogenes MGAS8232 uid57871
Streptococcus pyogenes Manfredo uid57847
Streptococcus pyogenes Alab49 uid162171
Streptococcus pyogenes MGAS315 uid57911
Streptococcus pyogenes SSI 1 uid57895
Streptococcus dysgalactiae equisimilis ATCC 12394 uid161979
Streptococcus dysgalactiae equisimilis GGS 124 uid59103
Streptococcus pyogenes NZ131 uid59035
Streptococcus pyogenes MGAS15252 uid158037
Streptococcus pyogenes MGAS1882 uid158061
Streptococcus suis D12 uid162127
Streptococcus suis ST1 uid167482
Streptococcus suis D9 uid162125
Streptococcus suis ST3 uid66327
Streptococcus suis P1 7 uid32235
Streptococcus suis JS14 uid162095
Streptococcus suis GZ1 uid161937
Streptococcus suis BM407 uid59321
Streptococcus suis A7 uid162111
Streptococcus suis 98HAH33 uid58665
Streptococcus suis 05ZYH33 uid58663
Streptococcus suis SC84 uid59323
Streptococcus suis SS12 uid162123
Streptococcus parasanguinis ATCC 15912 uid49313
Streptococcus parasanguinis FW213 uid163997
Streptococcus gordonii Challis substr CH1 uid57667
Streptococcus intermedius JTH08 uid168614
Streptococcus oralis Uo5 uid65449
Streptococcus mitis B6 uid46097
Streptococcus pseudopneumoniae IS7493 uid71153
Streptococcus pneumoniae AP200 uid52453
Streptococcus pneumoniae CGSP14 uid59181
Streptococcus pneumoniae Hungary19A 6 uid59117
Streptococcus pneumoniae INV200 uid162035
Streptococcus pneumoniae JJA uid59121
Streptococcus pneumoniae OXC141 uid162037
Streptococcus pneumoniae ST556 uid162191
Streptococcus pneumoniae TCH8431 19A uid49735
Streptococcus pneumoniae Taiwan19F 14 uid59119
Streptococcus pneumoniae ATCC 700669 uid59287
Streptococcus pneumoniae G54 uid59167
Streptococcus pneumoniae 670 6B uid52533
Streptococcus pneumoniae 70585 uid59125
Streptococcus pneumoniae INV104 uid162039
Streptococcus pneumoniae P1031 uid59123
Streptococcus pneumoniae TIGR4 uid57857
Streptococcus pneumoniae D39 uid58581
Streptococcus pneumoniae R6 uid57859
ffh

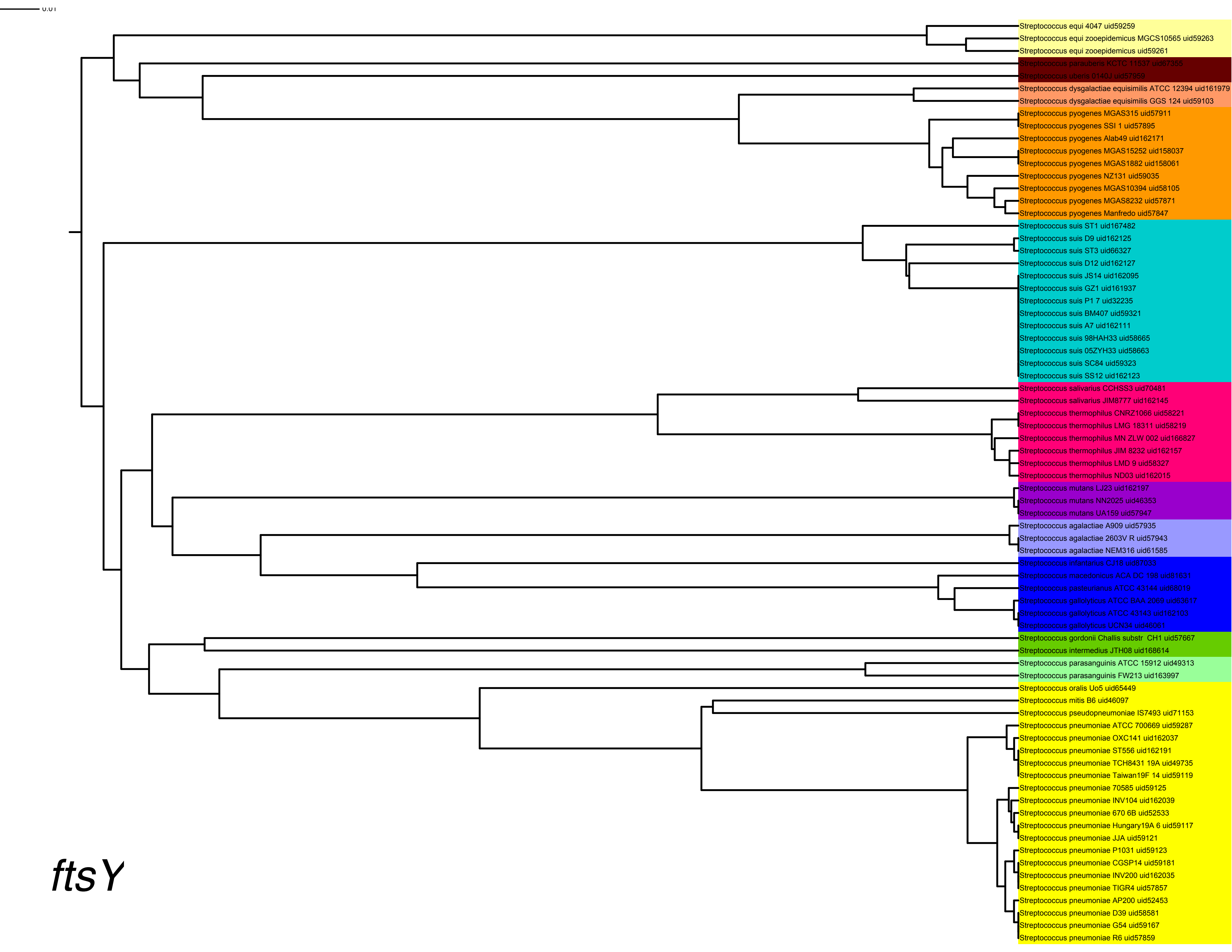
0.01
Streptococcus equi 4047 uid59259
Streptococcus equi zooepidemicus MGCS10565 uid59263
Streptococcus equi zooepidemicus uid59261
Streptococcus parauberis KCTC 11537 uid67355
Streptococcus uberis 0140J uid57959
Streptococcus dysgalactiae equisimilis ATCC 12394 uid161979
Streptococcus dysgalactiae equisimilis GGS 124 uid59103
Streptococcus pyogenes MGAS315 uid57911
Streptococcus pyogenes SSI 1 uid57895
Streptococcus pyogenes Alab49 uid162171
Streptococcus pyogenes MGAS15252 uid158037
Streptococcus pyogenes MGAS1882 uid158061
Streptococcus pyogenes NZ131 uid59035
Streptococcus pyogenes MGAS10394 uid58105
Streptococcus pyogenes MGAS8232 uid57871
Streptococcus pyogenes Manfredo uid57847
Streptococcus suis ST1 uid167482
Streptococcus suis D9 uid162125
Streptococcus suis ST3 uid66327
Streptococcus suis D12 uid162127
Streptococcus suis JS14 uid162095
Streptococcus suis GZ1 uid161937
Streptococcus suis P1 7 uid32235
Streptococcus suis BM407 uid59321
Streptococcus suis A7 uid162111
Streptococcus suis 98HAH33 uid58665
Streptococcus suis 05ZYH33 uid58663
Streptococcus suis SC84 uid59323
Streptococcus suis SS12 uid162123
Streptococcus salivarius CCHSS3 uid70481
Streptococcus salivarius JIM8777 uid162145
Streptococcus thermophilus CNRZ1066 uid58221
Streptococcus thermophilus LMG 18311 uid58219
Streptococcus thermophilus MN ZLW 002 uid166827
Streptococcus thermophilus JIM 8232 uid162157
Streptococcus thermophilus LMD 9 uid58327
Streptococcus thermophilus ND03 uid162015
Streptococcus mutans LJ23 uid162197
Streptococcus mutans NN2025 uid46353
Streptococcus mutans UA159 uid57947
Streptococcus agalactiae A909 uid57935
Streptococcus agalactiae 2603V R uid57943
Streptococcus agalactiae NEM316 uid61585
Streptococcus infantarius CJ18 uid87033
Streptococcus macedonicus ACA DC 198 uid81631
Streptococcus pasteurianus ATCC 43144 uid68019
Streptococcus gallolyticus ATCC BAA 2069 uid63617
Streptococcus gallolyticus ATCC 43143 uid162103
Streptococcus gallolyticus UCN34 uid46061
Streptococcus gordonii Challis substr CH1 uid57667
Streptococcus intermedius JTH08 uid168614
Streptococcus parasanguinis ATCC 15912 uid49313
Streptococcus parasanguinis FW213 uid163997
Streptococcus oralis Uo5 uid65449
Streptococcus mitis B6 uid46097
Streptococcus pseudopneumoniae IS7493 uid71153
Streptococcus pneumoniae ATCC 700669 uid59287
Streptococcus pneumoniae OXC141 uid162037
Streptococcus pneumoniae ST556 uid162191
Streptococcus pneumoniae TCH8431 19A uid49735
Streptococcus pneumoniae Taiwan19F 14 uid59119
Streptococcus pneumoniae 70585 uid59125
Streptococcus pneumoniae INV104 uid162039
Streptococcus pneumoniae 670 6B uid52533
Streptococcus pneumoniae Hungary19A 6 uid59117
Streptococcus pneumoniae JJA uid59121
Streptococcus pneumoniae P1031 uid59123
Streptococcus pneumoniae CGSP14 uid59181
Streptococcus pneumoniae INV200 uid162035
Streptococcus pneumoniae TIGR4 uid57857
Streptococcus pneumoniae AP200 uid52453
Streptococcus pneumoniae D39 uid58581
Streptococcus pneumoniae G54 uid59167
Streptococcus pneumoniae R6 uid57859
ftsY

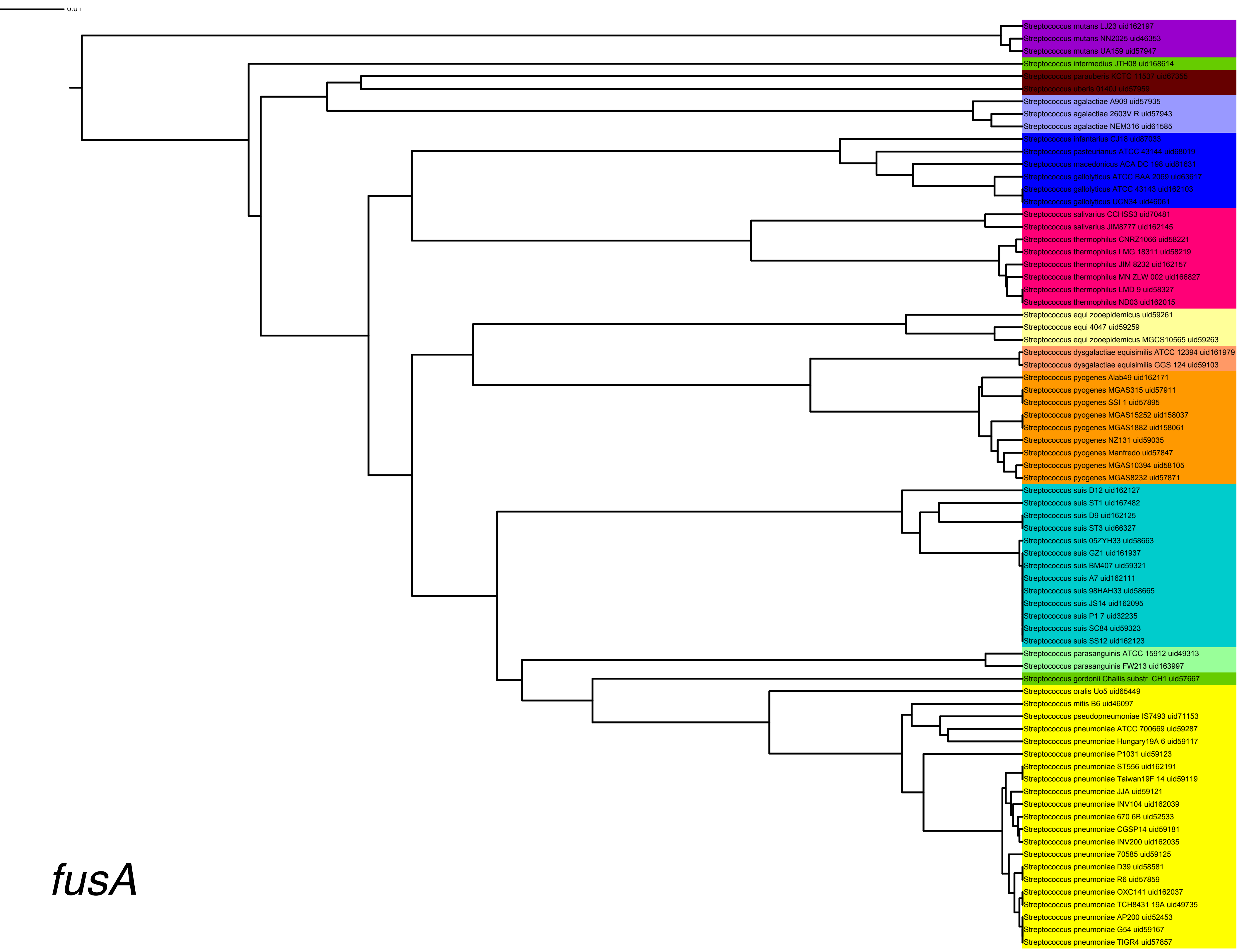

0.01
Streptococcus mutans LJ23 uid162197
Streptococcus mutans NN2025 uid46353
Streptococcus mutans UA159 uid57947
Streptococcus intermedius JTH08 uid168614
Streptococcus parauberis KCTC 11537 uid67355
Streptococcus uberis 0140J uid57959
Streptococcus agalactiae A909 uid57935
Streptococcus agalactiae 2603V R uid57943
Streptococcus agalactiae NEM316 uid61585
Streptococcus infantarius CJ18 uid87033
Streptococcus pasteurianus ATCC 43144 uid68019
Streptococcus macedonicus ACA DC 198 uid81631
Streptococcus gallolyticus ATCC BAA 2069 uid63617
Streptococcus gallolyticus ATCC 43143 uid162103
Streptococcus gallolyticus UCN34 uid46061
Streptococcus salivarius CCHSS3 uid70481
Streptococcus salivarius JIM8777 uid162145
Streptococcus thermophilus CNRZ1066 uid58221
Streptococcus thermophilus LMG 18311 uid58219
Streptococcus thermophilus JIM 8232 uid162157
Streptococcus thermophilus MN ZLW 002 uid166827
Streptococcus thermophilus LMD 9 uid58327
Streptococcus thermophilus ND03 uid162015
Streptococcus equi zooepidemicus uid59261
Streptococcus equi 4047 uid59259
Streptococcus equi zooepidemicus MGCS10565 uid59263
Streptococcus dysgalactiae equisimilis ATCC 12394 uid161979
Streptococcus dysgalactiae equisimilis GGS 124 uid59103
Streptococcus pyogenes Alab49 uid162171
Streptococcus pyogenes MGAS315 uid57911
Streptococcus pyogenes SSI 1 uid57895
Streptococcus pyogenes MGAS15252 uid158037
Streptococcus pyogenes MGAS1882 uid158061
Streptococcus pyogenes NZ131 uid59035
Streptococcus pyogenes Manfredo uid57847
Streptococcus pyogenes MGAS10394 uid58105
Streptococcus pyogenes MGAS8232 uid57871
Streptococcus suis D12 uid162127
Streptococcus suis ST1 uid167482
Streptococcus suis D9 uid162125
Streptococcus suis ST3 uid66327
Streptococcus suis 05ZYH33 uid58663
Streptococcus suis GZ1 uid161937
Streptococcus suis BM407 uid59321
Streptococcus suis A7 uid162111
Streptococcus suis 98HAH33 uid58665
Streptococcus suis JS14 uid162095
Streptococcus suis P1 7 uid32235
Streptococcus suis SC84 uid59323
Streptococcus suis SS12 uid162123
Streptococcus parasanguinis ATCC 15912 uid49313
Streptococcus parasanguinis FW213 uid163997
Streptococcus gordonii Challis substr CH1 uid57667
Streptococcus oralis Uo5 uid65449
Streptococcus mitis B6 uid46097
Streptococcus pseudopneumoniae IS7493 uid71153
Streptococcus pneumoniae ATCC 700669 uid59287
Streptococcus pneumoniae Hungary19A 6 uid59117
Streptococcus pneumoniae P1031 uid59123
Streptococcus pneumoniae ST556 uid162191
Streptococcus pneumoniae Taiwan19F 14 uid59119
Streptococcus pneumoniae JJA uid59121
Streptococcus pneumoniae INV104 uid162039
Streptococcus pneumoniae 670 6B uid52533
Streptococcus pneumoniae CGSP14 uid59181
Streptococcus pneumoniae INV200 uid162035
Streptococcus pneumoniae 70585 uid59125
Streptococcus pneumoniae D39 uid58581
Streptococcus pneumoniae R6 uid57859
Streptococcus pneumoniae OXC141 uid162037
Streptococcus pneumoniae TCH8431 19A uid49735
Streptococcus pneumoniae AP200 uid52453
Streptococcus pneumoniae G54 uid59167
Streptococcus pneumoniae TIGR4 uid57857
fusA

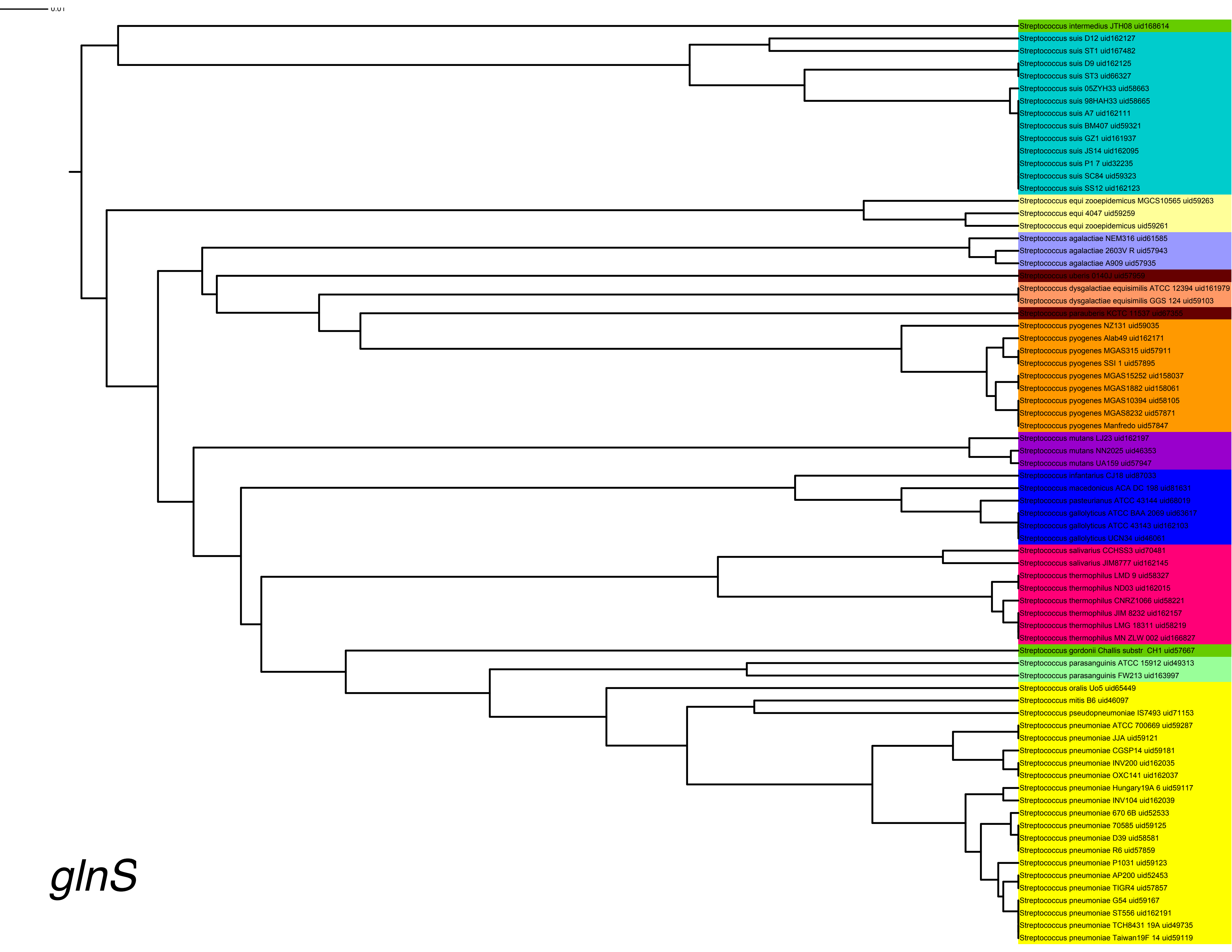

0.01
Streptococcus intermedius JTH08 uid168614
Streptococcus suis D12 uid162127
Streptococcus suis ST1 uid167482
Streptococcus suis D9 uid162125
Streptococcus suis ST3 uid66327
Streptococcus suis 05ZYH33 uid58663
Streptococcus suis 98HAH33 uid58665
Streptococcus suis A7 uid162111
Streptococcus suis BM407 uid59321
Streptococcus suis GZ1 uid161937
Streptococcus suis JS14 uid162095
Streptococcus suis P1 7 uid32235
Streptococcus suis SC84 uid59323
Streptococcus suis SS12 uid162123
Streptococcus equi zooepidemicus MGCS10565 uid59263
Streptococcus equi 4047 uid59259
Streptococcus equi zooepidemicus uid59261
Streptococcus agalactiae NEM316 uid61585
Streptococcus agalactiae 2603V R uid57943
Streptococcus agalactiae A909 uid57935
Streptococcus uberis 0140J uid57959
Streptococcus dysgalactiae equisimilis ATCC 12394 uid161979
Streptococcus dysgalactiae equisimilis GGS 124 uid59103
Streptococcus parauberis KCTC 11537 uid67355
Streptococcus pyogenes NZ131 uid59035
Streptococcus pyogenes Alab49 uid162171
Streptococcus pyogenes MGAS315 uid57911
Streptococcus pyogenes SSI 1 uid57895
Streptococcus pyogenes MGAS15252 uid158037
Streptococcus pyogenes MGAS1882 uid158061
Streptococcus pyogenes MGAS10394 uid58105
Streptococcus pyogenes MGAS8232 uid57871
Streptococcus pyogenes Manfredo uid57847
Streptococcus mutans LJ23 uid162197
Streptococcus mutans NN2025 uid46353
Streptococcus mutans UA159 uid57947
Streptococcus infantarius CJ18 uid87033
Streptococcus macedonicus ACA DC 198 uid81631
Streptococcus pasteurianus ATCC 43144 uid68019
Streptococcus gallolyticus ATCC BAA 2069 uid63617
Streptococcus gallolyticus ATCC 43143 uid162103
Streptococcus gallolyticus UCN34 uid46061
Streptococcus salivarius CCHSS3 uid70481
Streptococcus salivarius JIM8777 uid162145
Streptococcus thermophilus LMD 9 uid58327
Streptococcus thermophilus ND03 uid162015
Streptococcus thermophilus CNRZ1066 uid58221
Streptococcus thermophilus JIM 8232 uid162157
Streptococcus thermophilus LMG 18311 uid58219
Streptococcus thermophilus MN ZLW 002 uid166827
Streptococcus gordonii Challis substr CH1 uid57667
Streptococcus parasanguinis ATCC 15912 uid49313
Streptococcus parasanguinis FW213 uid163997
Streptococcus oralis Uo5 uid65449
Streptococcus mitis B6 uid46097
Streptococcus pseudopneumoniae IS7493 uid71153
Streptococcus pneumoniae ATCC 700669 uid59287
Streptococcus pneumoniae JJA uid59121
Streptococcus pneumoniae CGSP14 uid59181
Streptococcus pneumoniae INV200 uid162035
Streptococcus pneumoniae OXC141 uid162037
Streptococcus pneumoniae Hungary19A 6 uid59117
Streptococcus pneumoniae INV104 uid162039
Streptococcus pneumoniae 670 6B uid52533
Streptococcus pneumoniae 70585 uid59125
Streptococcus pneumoniae D39 uid58581
Streptococcus pneumoniae R6 uid57859
Streptococcus pneumoniae P1031 uid59123
Streptococcus pneumoniae AP200 uid52453
Streptococcus pneumoniae TIGR4 uid57857
Streptococcus pneumoniae G54 uid59167
Streptococcus pneumoniae ST556 uid162191
Streptococcus pneumoniae TCH8431 19A uid49735
Streptococcus pneumoniae Taiwan19F 14 uid59119
glnS

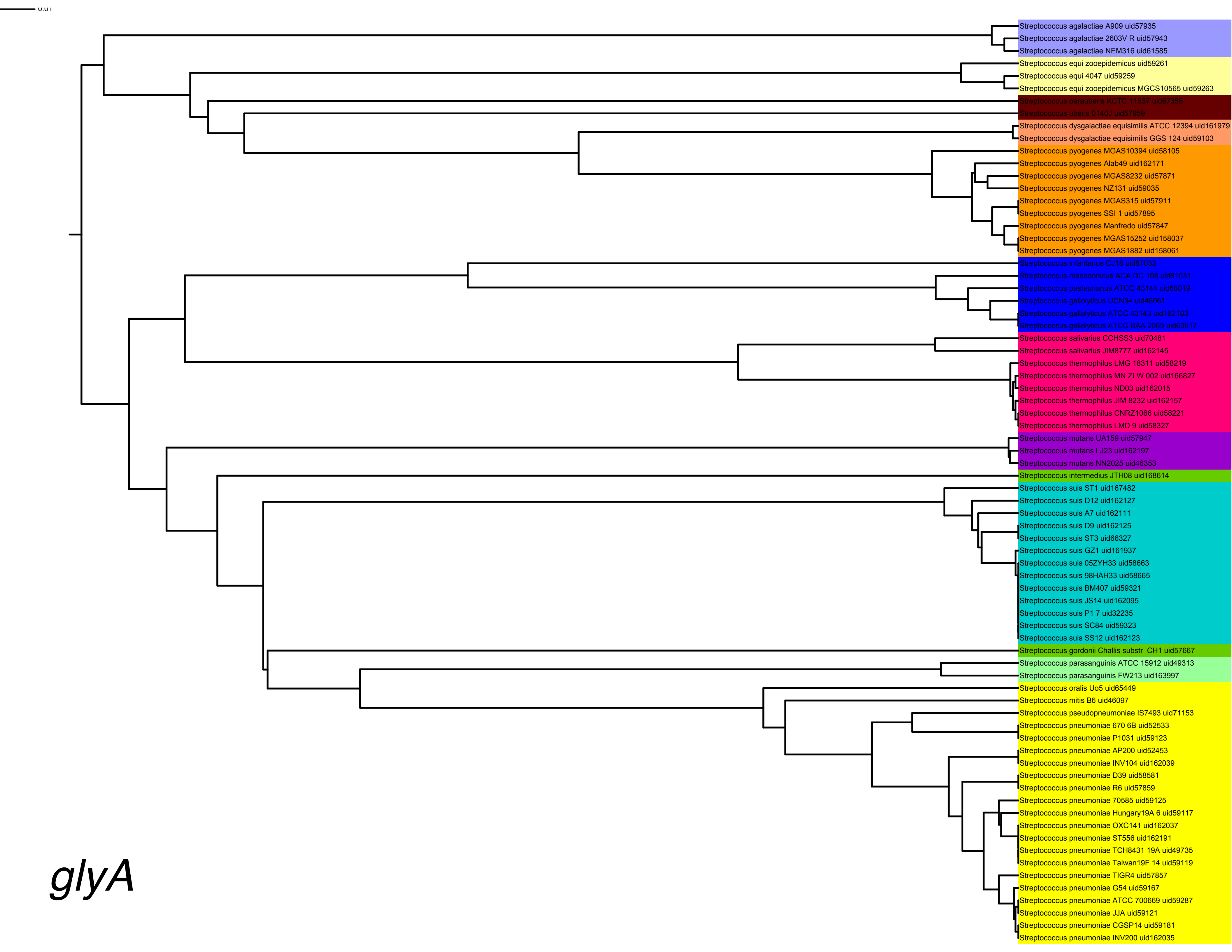
0.01
Streptococcus agalactiae A909 uid57935
Streptococcus agalactiae 2603V R uid57943
Streptococcus agalactiae NEM316 uid61585
Streptococcus equi zooepidemicus uid59261
Streptococcus equi 4047 uid59259
Streptococcus equi zooepidemicus MGCS10565 uid59263
Streptococcus parauberis KCTC 11537 uid67355
Streptococcus uberis 0140J uid57959
Streptococcus dysgalactiae equisimilis ATCC 12394 uid161979
Streptococcus dysgalactiae equisimilis GGS 124 uid59103
Streptococcus pyogenes MGAS10394 uid58105
Streptococcus pyogenes Alab49 uid162171
Streptococcus pyogenes MGAS8232 uid57871
Streptococcus pyogenes NZ131 uid59035
Streptococcus pyogenes MGAS315 uid57911
Streptococcus pyogenes SSI 1 uid57895
Streptococcus pyogenes Manfredo uid57847
Streptococcus pyogenes MGAS15252 uid158037
Streptococcus pyogenes MGAS1882 uid158061
Streptococcus infantarius CJ18 uid87033
Streptococcus macedonicus ACA DC 198 uid81631
Streptococcus pasteurianus ATCC 43144 uid68019
Streptococcus gallolyticus UCN34 uid46061
Streptococcus gallolyticus ATCC 43143 uid162103
Streptococcus gallolyticus ATCC BAA 2069 uid63617
Streptococcus salivarius CCHSS3 uid70481
Streptococcus salivarius JIM8777 uid162145
Streptococcus thermophilus LMG 18311 uid58219
Streptococcus thermophilus MN ZLW 002 uid166827
Streptococcus thermophilus ND03 uid162015
Streptococcus thermophilus JIM 8232 uid162157
Streptococcus thermophilus CNRZ1066 uid58221
Streptococcus thermophilus LMD 9 uid58327
Streptococcus mutans UA159 uid57947
Streptococcus mutans LJ23 uid162197
Streptococcus mutans NN2025 uid46353
Streptococcus intermedius JTH08 uid168614
Streptococcus suis ST1 uid167482
Streptococcus suis D12 uid162127
Streptococcus suis A7 uid162111
Streptococcus suis D9 uid162125
Streptococcus suis ST3 uid66327
Streptococcus suis GZ1 uid161937
Streptococcus suis 05ZYH33 uid58663
Streptococcus suis 98HAH33 uid58665
Streptococcus suis BM407 uid59321
Streptococcus suis JS14 uid162095
Streptococcus suis P1 7 uid32235
Streptococcus suis SC84 uid59323
Streptococcus suis SS12 uid162123
Streptococcus gordonii Challis substr CH1 uid57667
Streptococcus parasanguinis ATCC 15912 uid49313
Streptococcus parasanguinis FW213 uid163997
Streptococcus oralis Uo5 uid65449
Streptococcus mitis B6 uid46097
Streptococcus pseudopneumoniae IS7493 uid71153
Streptococcus pneumoniae 670 6B uid52533
Streptococcus pneumoniae P1031 uid59123
Streptococcus pneumoniae AP200 uid52453
Streptococcus pneumoniae INV104 uid162039
Streptococcus pneumoniae D39 uid58581
Streptococcus pneumoniae R6 uid57859
Streptococcus pneumoniae 70585 uid59125
Streptococcus pneumoniae Hungary19A 6 uid59117
Streptococcus pneumoniae OXC141 uid162037
Streptococcus pneumoniae ST556 uid162191
Streptococcus pneumoniae TCH8431 19A uid49735
Streptococcus pneumoniae Taiwan19F 14 uid59119
Streptococcus pneumoniae TIGR4 uid57857
Streptococcus pneumoniae G54 uid59167
Streptococcus pneumoniae ATCC 700669 uid59287
Streptococcus pneumoniae JJA uid59121
Streptococcus pneumoniae CGSP14 uid59181
Streptococcus pneumoniae INV200 uid162035
glyA

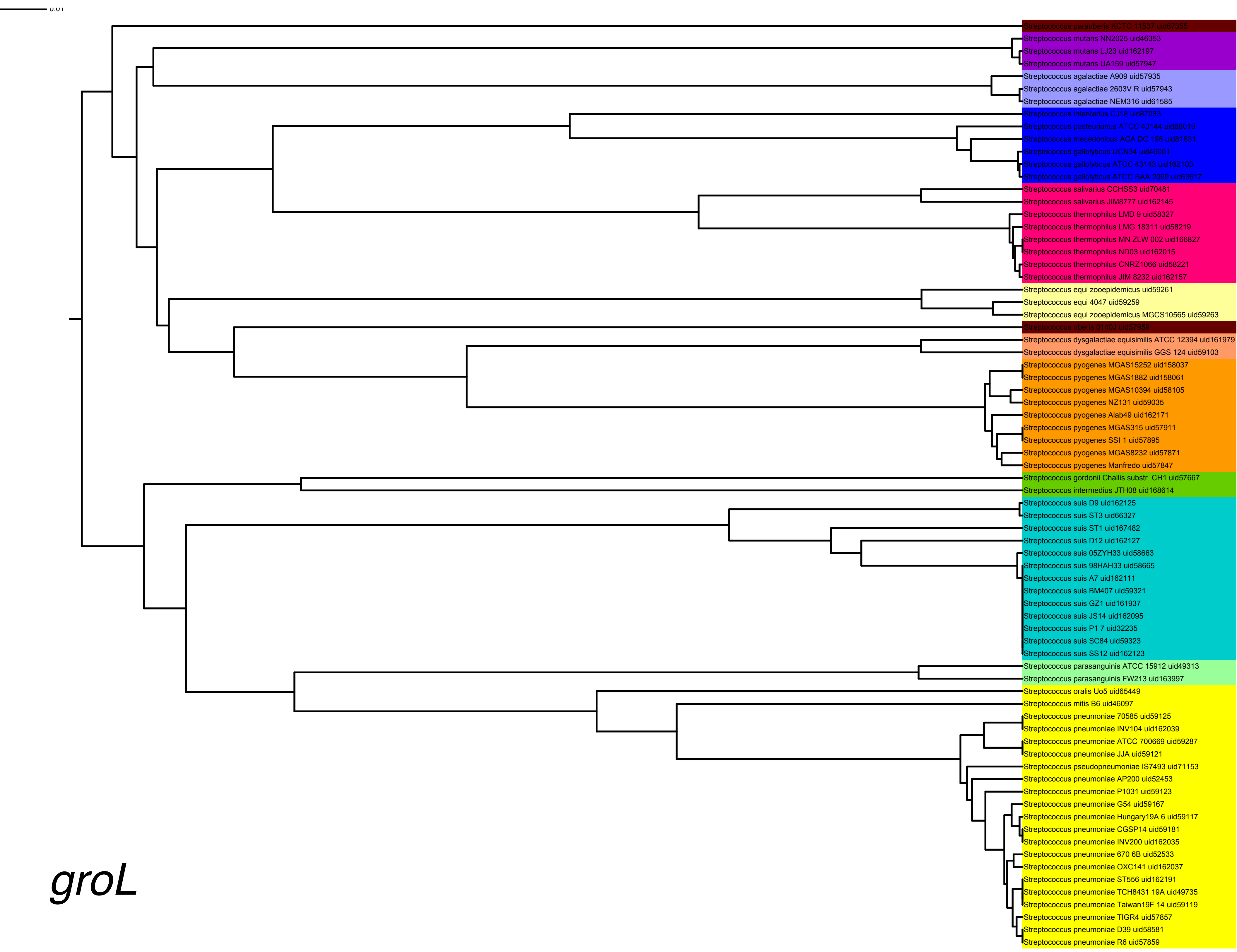
0.01
Streptococcus parauberis KCTC 11537 uid67355
Streptococcus mutans NN2025 uid46353
Streptococcus mutans LJ23 uid162197
Streptococcus mutans UA159 uid57947
Streptococcus agalactiae A909 uid57935
Streptococcus agalactiae 2603V R uid57943
Streptococcus agalactiae NEM316 uid61585
Streptococcus infantarius CJ18 uid87033
Streptococcus pasteurianus ATCC 43144 uid68019
Streptococcus macedonicus ACA DC 198 uid81631
Streptococcus gallolyticus UCN34 uid46061
Streptococcus gallolyticus ATCC 43143 uid162103
Streptococcus gallolyticus ATCC BAA 2069 uid63617
Streptococcus salivarius CCHSS3 uid70481
Streptococcus salivarius JIM8777 uid162145
Streptococcus thermophilus LMD 9 uid58327
Streptococcus thermophilus LMG 18311 uid58219
Streptococcus thermophilus MN ZLW 002 uid166827
Streptococcus thermophilus ND03 uid162015
Streptococcus thermophilus CNRZ1066 uid58221
Streptococcus thermophilus JIM 8232 uid162157
Streptococcus equi zooepidemicus uid59261
Streptococcus equi 4047 uid59259
Streptococcus equi zooepidemicus MGCS10565 uid59263
Streptococcus uberis 0140J uid57959
Streptococcus dysgalactiae equisimilis ATCC 12394 uid161979
Streptococcus dysgalactiae equisimilis GGS 124 uid59103
Streptococcus pyogenes MGAS15252 uid158037
Streptococcus pyogenes MGAS1882 uid158061
Streptococcus pyogenes MGAS10394 uid58105
Streptococcus pyogenes NZ131 uid59035
Streptococcus pyogenes Alab49 uid162171
Streptococcus pyogenes MGAS315 uid57911
Streptococcus pyogenes SSI 1 uid57895
Streptococcus pyogenes MGAS8232 uid57871
Streptococcus pyogenes Manfredo uid57847
Streptococcus gordonii Challis substr CH1 uid57667
Streptococcus intermedius JTH08 uid168614
Streptococcus suis D9 uid162125
Streptococcus suis ST3 uid66327
Streptococcus suis ST1 uid167482
Streptococcus suis D12 uid162127
Streptococcus suis 05ZYH33 uid58663
Streptococcus suis 98HAH33 uid58665
Streptococcus suis A7 uid162111
Streptococcus suis BM407 uid59321
Streptococcus suis GZ1 uid161937
Streptococcus suis JS14 uid162095
Streptococcus suis P1 7 uid32235
Streptococcus suis SC84 uid59323
Streptococcus suis SS12 uid162123
Streptococcus parasanguinis ATCC 15912 uid49313
Streptococcus parasanguinis FW213 uid163997
Streptococcus oralis Uo5 uid65449
Streptococcus mitis B6 uid46097
Streptococcus pneumoniae 70585 uid59125
Streptococcus pneumoniae INV104 uid162039
Streptococcus pneumoniae ATCC 700669 uid59287
Streptococcus pneumoniae JJA uid59121
Streptococcus pseudopneumoniae IS7493 uid71153
Streptococcus pneumoniae AP200 uid52453
Streptococcus pneumoniae P1031 uid59123
Streptococcus pneumoniae G54 uid59167
Streptococcus pneumoniae Hungary19A 6 uid59117
Streptococcus pneumoniae CGSP14 uid59181
Streptococcus pneumoniae INV200 uid162035
Streptococcus pneumoniae 670 6B uid52533
Streptococcus pneumoniae OXC141 uid162037
Streptococcus pneumoniae ST556 uid162191
Streptococcus pneumoniae TCH8431 19A uid49735
Streptococcus pneumoniae Taiwan19F 14 uid59119
Streptococcus pneumoniae TIGR4 uid57857
Streptococcus pneumoniae D39 uid58581
Streptococcus pneumoniae R6 uid57859
groL

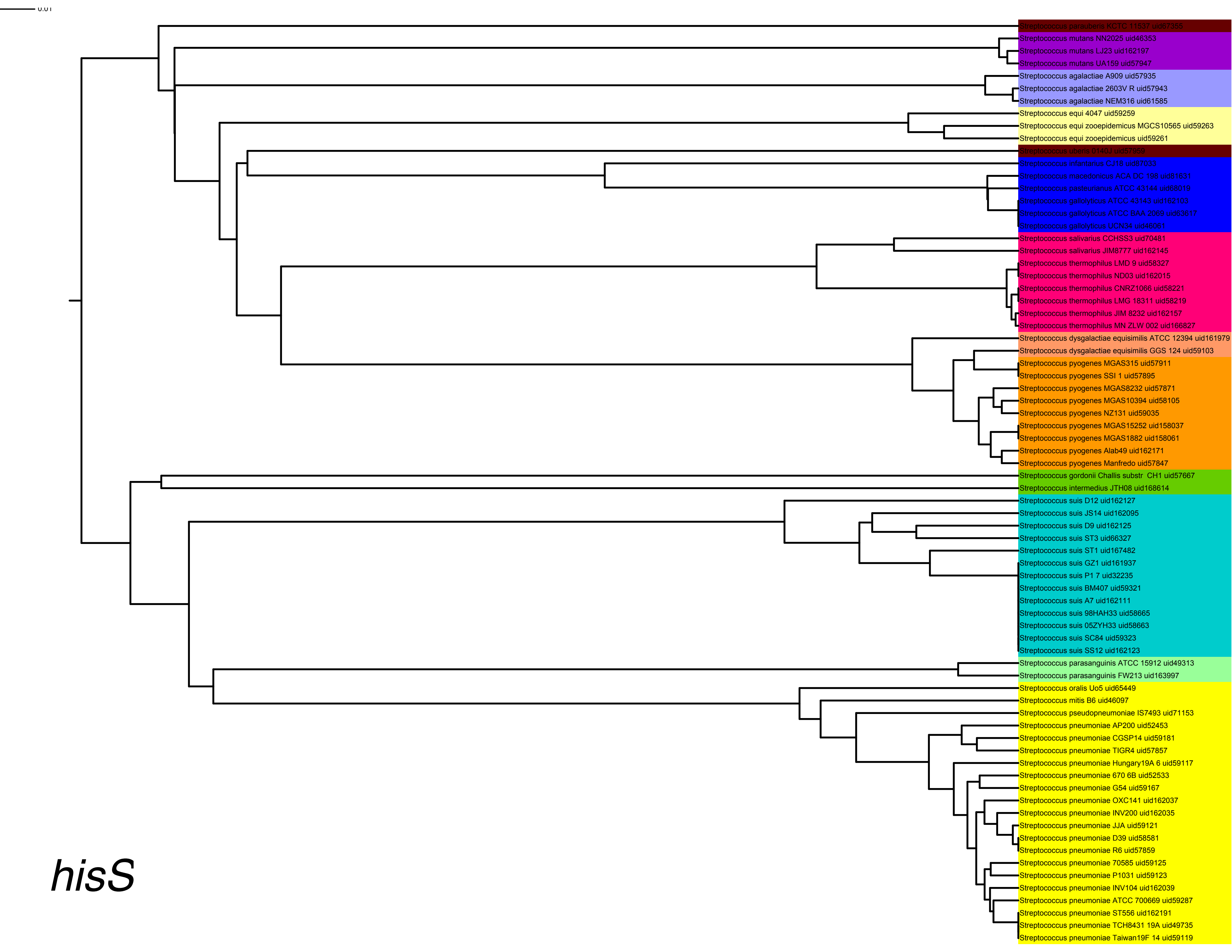
0.01
Streptococcus parauberis KCTC 11537 uid67355
Streptococcus mutans NN2025 uid46353
Streptococcus mutans LJ23 uid162197
Streptococcus mutans UA159 uid57947
Streptococcus agalactiae A909 uid57935
Streptococcus agalactiae 2603V R uid57943
Streptococcus agalactiae NEM316 uid61585
Streptococcus equi 4047 uid59259
Streptococcus equi zooepidemicus MGCS10565 uid59263
Streptococcus equi zooepidemicus uid59261
Streptococcus uberis 0140J uid57959
Streptococcus infantarius CJ18 uid87033
Streptococcus macedonicus ACA DC 198 uid81631
Streptococcus pasteurianus ATCC 43144 uid68019
Streptococcus gallolyticus ATCC 43143 uid162103
Streptococcus gallolyticus ATCC BAA 2069 uid63617
Streptococcus gallolyticus UCN34 uid46061
Streptococcus salivarius CCHSS3 uid70481
Streptococcus salivarius JIM8777 uid162145
Streptococcus thermophilus LMD 9 uid58327
Streptococcus thermophilus ND03 uid162015
Streptococcus thermophilus CNRZ1066 uid58221
Streptococcus thermophilus LMG 18311 uid58219
Streptococcus thermophilus JIM 8232 uid162157
Streptococcus thermophilus MN ZLW 002 uid166827
Streptococcus dysgalactiae equisimilis ATCC 12394 uid161979
Streptococcus dysgalactiae equisimilis GGS 124 uid59103
Streptococcus pyogenes MGAS315 uid57911
Streptococcus pyogenes SSI 1 uid57895
Streptococcus pyogenes MGAS8232 uid57871
Streptococcus pyogenes MGAS10394 uid58105
Streptococcus pyogenes NZ131 uid59035
Streptococcus pyogenes MGAS15252 uid158037
Streptococcus pyogenes MGAS1882 uid158061
Streptococcus pyogenes Alab49 uid162171
Streptococcus pyogenes Manfredo uid57847
Streptococcus gordonii Challis substr CH1 uid57667
Streptococcus intermedius JTH08 uid168614
Streptococcus suis D12 uid162127
Streptococcus suis JS14 uid162095
Streptococcus suis D9 uid162125
Streptococcus suis ST3 uid66327
Streptococcus suis ST1 uid167482
Streptococcus suis GZ1 uid161937
Streptococcus suis P1 7 uid32235
Streptococcus suis BM407 uid59321
Streptococcus suis A7 uid162111
Streptococcus suis 98HAH33 uid58665
Streptococcus suis 05ZYH33 uid58663
Streptococcus suis SC84 uid59323
Streptococcus suis SS12 uid162123
Streptococcus parasanguinis ATCC 15912 uid49313
Streptococcus parasanguinis FW213 uid163997
Streptococcus oralis Uo5 uid65449
Streptococcus mitis B6 uid46097
Streptococcus pseudopneumoniae IS7493 uid71153
Streptococcus pneumoniae AP200 uid52453
Streptococcus pneumoniae CGSP14 uid59181
Streptococcus pneumoniae TIGR4 uid57857
Streptococcus pneumoniae Hungary19A 6 uid59117
Streptococcus pneumoniae 670 6B uid52533
Streptococcus pneumoniae G54 uid59167
Streptococcus pneumoniae OXC141 uid162037
Streptococcus pneumoniae INV200 uid162035
Streptococcus pneumoniae JJA uid59121
Streptococcus pneumoniae D39 uid58581
Streptococcus pneumoniae R6 uid57859
Streptococcus pneumoniae 70585 uid59125
Streptococcus pneumoniae P1031 uid59123
Streptococcus pneumoniae INV104 uid162039
Streptococcus pneumoniae ATCC 700669 uid59287
Streptococcus pneumoniae ST556 uid162191
Streptococcus pneumoniae TCH8431 19A uid49735
Streptococcus pneumoniae Taiwan19F 14 uid59119
hisS

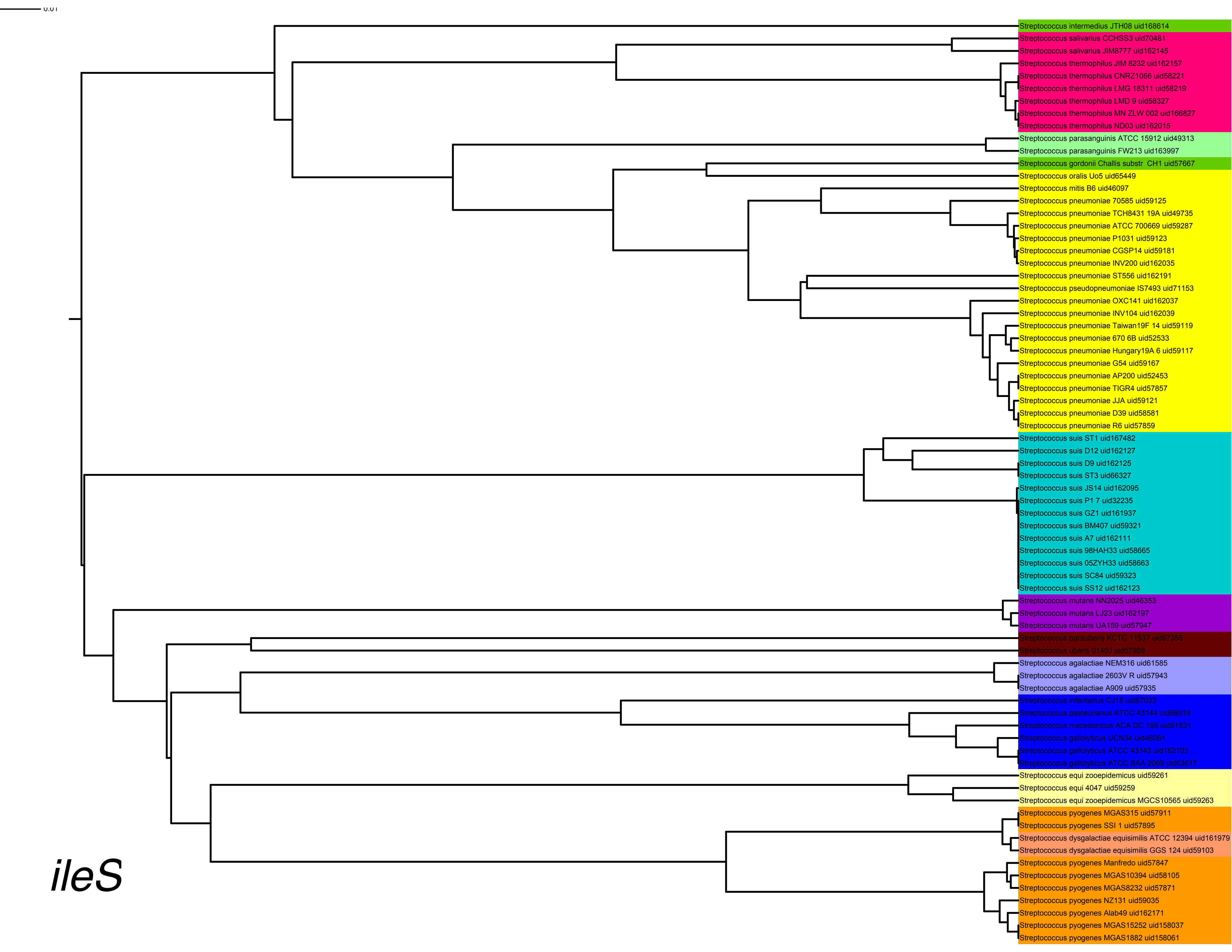
0.01
Streptococcus intermedius JTH08 uid168614
Streptococcus salivarius CCHSS3 uid70481
Streptococcus salivarius JIM8777 uid162145
Streptococcus thermophilus JIM 8232 uid162157
Streptococcus thermophilus CNRZ1066 uid58221
Streptococcus thermophilus LMG 18311 uid58219
Streptococcus thermophilus LMD 9 uid58327
Streptococcus thermophilus MN ZLW 002 uid166827
Streptococcus thermophilus ND03 uid162015
Streptococcus parasanguinis ATCC 15912 uid49313
Streptococcus parasanguinis FW213 uid163997
Streptococcus gordonii Challis substr CH1 uid57667
Streptococcus oralis Uo5 uid65449
Streptococcus mitis B6 uid46097
Streptococcus pneumoniae 70585 uid59125
Streptococcus pneumoniae TCH8431 19A uid49735
Streptococcus pneumoniae ATCC 700669 uid59287
Streptococcus pneumoniae P1031 uid59123
Streptococcus pneumoniae CGSP14 uid59181
Streptococcus pneumoniae INV200 uid162035
Streptococcus pneumoniae ST556 uid162191
Streptococcus pseudopneumoniae IS7493 uid71153
Streptococcus pneumoniae OXC141 uid162037
Streptococcus pneumoniae INV104 uid162039
Streptococcus pneumoniae Taiwan19F 14 uid59119
Streptococcus pneumoniae 670 6B uid52533
Streptococcus pneumoniae Hungary19A 6 uid59117
Streptococcus pneumoniae G54 uid59167
Streptococcus pneumoniae AP200 uid52453
Streptococcus pneumoniae TIGR4 uid57857
Streptococcus pneumoniae JJA uid59121
Streptococcus pneumoniae D39 uid58581
Streptococcus pneumoniae R6 uid57859
Streptococcus suis ST1 uid167482
Streptococcus suis D12 uid162127
Streptococcus suis D9 uid162125
Streptococcus suis ST3 uid66327
Streptococcus suis JS14 uid162095
Streptococcus suis P1 7 uid32235
Streptococcus suis GZ1 uid161937
Streptococcus suis BM407 uid59321
Streptococcus suis A7 uid162111
Streptococcus suis 98HAH33 uid58665
Streptococcus suis 05ZYH33 uid58663
Streptococcus suis SC84 uid59323
Streptococcus suis SS12 uid162123
Streptococcus mutans NN2025 uid46353
Streptococcus mutans LJ23 uid162197
Streptococcus mutans UA159 uid57947
Streptococcus parauberis KCTC 11537 uid67355
Streptococcus uberis 0140J uid57959
Streptococcus agalactiae NEM316 uid61585
Streptococcus agalactiae 2603V R uid57943
Streptococcus agalactiae A909 uid57935
Streptococcus infantarius CJ18 uid87033
Streptococcus pasteurianus ATCC 43144 uid68019
Streptococcus macedonicus ACA DC 198 uid81631
Streptococcus gallolyticus UCN34 uid46061
Streptococcus gallolyticus ATCC 43143 uid162103
Streptococcus gallolyticus ATCC BAA 2069 uid63617
Streptococcus equi zooepidemicus uid59261
Streptococcus equi 4047 uid59259
Streptococcus equi zooepidemicus MGCS10565 uid59263
Streptococcus pyogenes MGAS315 uid57911
Streptococcus pyogenes SSI 1 uid57895
Streptococcus dysgalactiae equisimilis ATCC 12394 uid161979
Streptococcus dysgalactiae equisimilis GGS 124 uid59103
Streptococcus pyogenes Manfredo uid57847
Streptococcus pyogenes MGAS10394 uid58105
Streptococcus pyogenes MGAS8232 uid57871
Streptococcus pyogenes NZ131 uid59035
Streptococcus pyogenes Alab49 uid162171
Streptococcus pyogenes MGAS15252 uid158037
Streptococcus pyogenes MGAS1882 uid158061
ileS

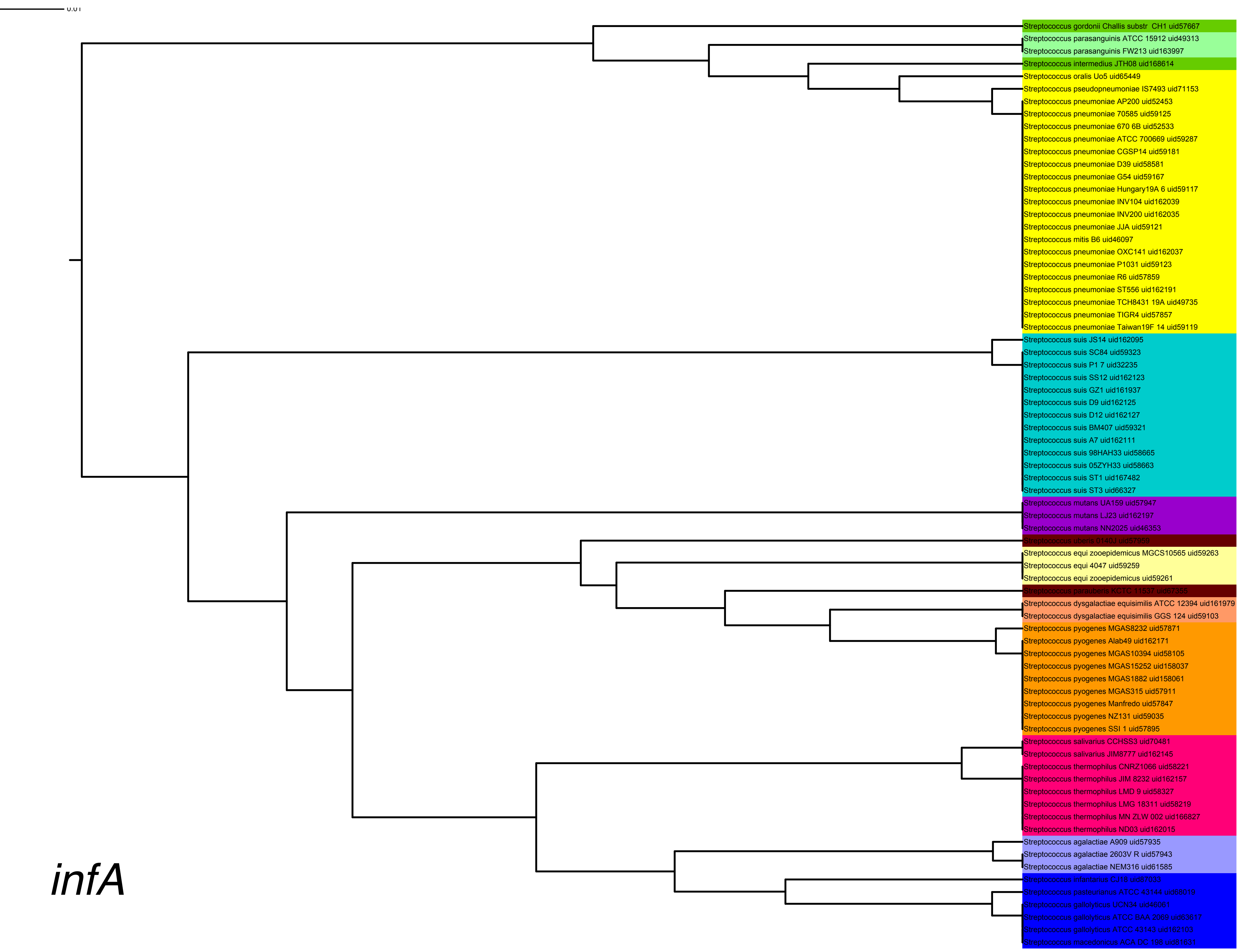
0.01
Streptococcus gordonii Challis substr CH1 uid57667
Streptococcus parasanguinis ATCC 15912 uid49313
Streptococcus parasanguinis FW213 uid163997
Streptococcus intermedius JTH08 uid168614
Streptococcus oralis Uo5 uid65449
Streptococcus pseudopneumoniae IS7493 uid71153
Streptococcus pneumoniae AP200 uid52453
Streptococcus pneumoniae 70585 uid59125
Streptococcus pneumoniae 670 6B uid52533
Streptococcus pneumoniae ATCC 700669 uid59287
Streptococcus pneumoniae CGSP14 uid59181
Streptococcus pneumoniae D39 uid58581
Streptococcus pneumoniae G54 uid59167
Streptococcus pneumoniae Hungary19A 6 uid59117
Streptococcus pneumoniae INV104 uid162039
Streptococcus pneumoniae INV200 uid162035
Streptococcus pneumoniae JJA uid59121
Streptococcus mitis B6 uid46097
Streptococcus pneumoniae OXC141 uid162037
Streptococcus pneumoniae P1031 uid59123
Streptococcus pneumoniae R6 uid57859
Streptococcus pneumoniae ST556 uid162191
Streptococcus pneumoniae TCH8431 19A uid49735
Streptococcus pneumoniae TIGR4 uid57857
Streptococcus pneumoniae Taiwan19F 14 uid59119
Streptococcus suis JS14 uid162095
Streptococcus suis SC84 uid59323
Streptococcus suis P1 7 uid32235
Streptococcus suis SS12 uid162123
Streptococcus suis GZ1 uid161937
Streptococcus suis D9 uid162125
Streptococcus suis D12 uid162127
Streptococcus suis BM407 uid59321
Streptococcus suis A7 uid162111
Streptococcus suis 98HAH33 uid58665
Streptococcus suis 05ZYH33 uid58663
Streptococcus suis ST1 uid167482
Streptococcus suis ST3 uid66327
Streptococcus mutans UA159 uid57947
Streptococcus mutans LJ23 uid162197
Streptococcus mutans NN2025 uid46353
Streptococcus uberis 0140J uid57959
Streptococcus equi zooepidemicus MGCS10565 uid59263
Streptococcus equi 4047 uid59259
Streptococcus equi zooepidemicus uid59261
Streptococcus parauberis KCTC 11537 uid67355
Streptococcus dysgalactiae equisimilis ATCC 12394 uid161979
Streptococcus dysgalactiae equisimilis GGS 124 uid59103
Streptococcus pyogenes MGAS8232 uid57871
Streptococcus pyogenes Alab49 uid162171
Streptococcus pyogenes MGAS10394 uid58105
Streptococcus pyogenes MGAS15252 uid158037
Streptococcus pyogenes MGAS1882 uid158061
Streptococcus pyogenes MGAS315 uid57911
Streptococcus pyogenes Manfredo uid57847
Streptococcus pyogenes NZ131 uid59035
Streptococcus pyogenes SSI 1 uid57895
Streptococcus salivarius CCHSS3 uid70481
Streptococcus salivarius JIM8777 uid162145
Streptococcus thermophilus CNRZ1066 uid58221
Streptococcus thermophilus JIM 8232 uid162157
Streptococcus thermophilus LMD 9 uid58327
Streptococcus thermophilus LMG 18311 uid58219
Streptococcus thermophilus MN ZLW 002 uid166827
Streptococcus thermophilus ND03 uid162015
Streptococcus agalactiae A909 uid57935
Streptococcus agalactiae 2603V R uid57943
Streptococcus agalactiae NEM316 uid61585
Streptococcus infantarius CJ18 uid87033
Streptococcus pasteurianus ATCC 43144 uid68019
Streptococcus gallolyticus UCN34 uid46061
Streptococcus gallolyticus ATCC BAA 2069 uid63617
Streptococcus gallolyticus ATCC 43143 uid162103
Streptococcus macedonicus ACA DC 198 uid81631
infA

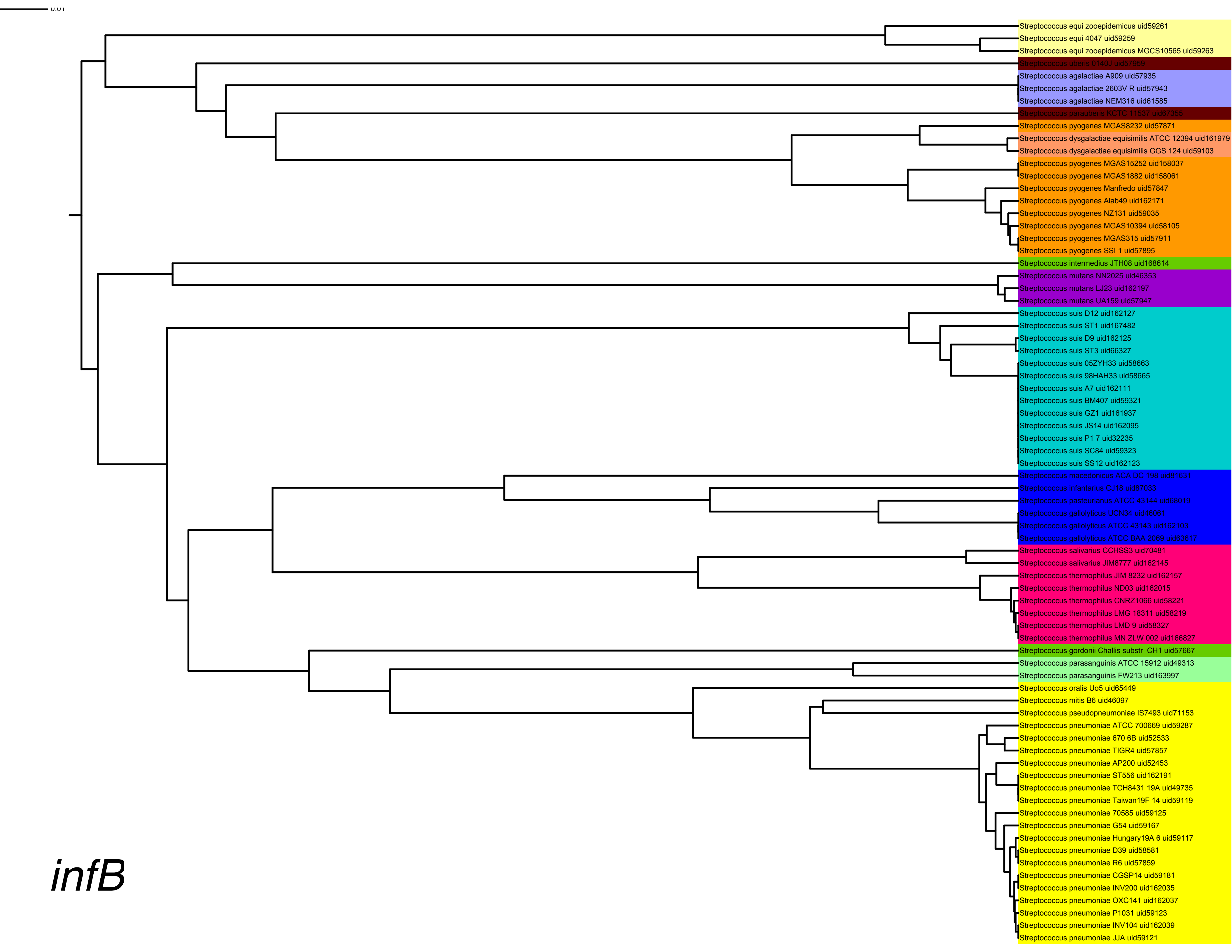
0.01
Streptococcus equi zooepidemicus uid59261
Streptococcus equi 4047 uid59259
Streptococcus equi zooepidemicus MGCS10565 uid59263
Streptococcus uberis 0140J uid57959
Streptococcus agalactiae A909 uid57935
Streptococcus agalactiae 2603V R uid57943
Streptococcus agalactiae NEM316 uid61585
Streptococcus parauberis KCTC 11537 uid67355
Streptococcus pyogenes MGAS8232 uid57871
Streptococcus dysgalactiae equisimilis ATCC 12394 uid161979
Streptococcus dysgalactiae equisimilis GGS 124 uid59103
Streptococcus pyogenes MGAS15252 uid158037
Streptococcus pyogenes MGAS1882 uid158061
Streptococcus pyogenes Manfredo uid57847
Streptococcus pyogenes Alab49 uid162171
Streptococcus pyogenes NZ131 uid59035
Streptococcus pyogenes MGAS10394 uid58105
Streptococcus pyogenes MGAS315 uid57911
Streptococcus pyogenes SSI 1 uid57895
Streptococcus intermedius JTH08 uid168614
Streptococcus mutans NN2025 uid46353
Streptococcus mutans LJ23 uid162197
Streptococcus mutans UA159 uid57947
Streptococcus suis D12 uid162127
Streptococcus suis ST1 uid167482
Streptococcus suis D9 uid162125
Streptococcus suis ST3 uid66327
Streptococcus suis 05ZYH33 uid58663
Streptococcus suis 98HAH33 uid58665
Streptococcus suis A7 uid162111
Streptococcus suis BM407 uid59321
Streptococcus suis GZ1 uid161937
Streptococcus suis JS14 uid162095
Streptococcus suis P1 7 uid32235
Streptococcus suis SC84 uid59323
Streptococcus suis SS12 uid162123
Streptococcus macedonicus ACA DC 198 uid81631
Streptococcus infantarius CJ18 uid87033
Streptococcus pasteurianus ATCC 43144 uid68019
Streptococcus gallolyticus UCN34 uid46061
Streptococcus gallolyticus ATCC 43143 uid162103
Streptococcus gallolyticus ATCC BAA 2069 uid63617
Streptococcus salivarius CCHSS3 uid70481
Streptococcus salivarius JIM8777 uid162145
Streptococcus thermophilus JIM 8232 uid162157
Streptococcus thermophilus ND03 uid162015
Streptococcus thermophilus CNRZ1066 uid58221
Streptococcus thermophilus LMG 18311 uid58219
Streptococcus thermophilus LMD 9 uid58327
Streptococcus thermophilus MN ZLW 002 uid166827
Streptococcus gordonii Challis substr CH1 uid57667
Streptococcus parasanguinis ATCC 15912 uid49313
Streptococcus parasanguinis FW213 uid163997
Streptococcus oralis Uo5 uid65449
Streptococcus mitis B6 uid46097
Streptococcus pseudopneumoniae IS7493 uid71153
Streptococcus pneumoniae ATCC 700669 uid59287
Streptococcus pneumoniae 670 6B uid52533
Streptococcus pneumoniae TIGR4 uid57857
Streptococcus pneumoniae AP200 uid52453
Streptococcus pneumoniae ST556 uid162191
Streptococcus pneumoniae TCH8431 19A uid49735
Streptococcus pneumoniae Taiwan19F 14 uid59119
Streptococcus pneumoniae 70585 uid59125
Streptococcus pneumoniae G54 uid59167
Streptococcus pneumoniae Hungary19A 6 uid59117
Streptococcus pneumoniae D39 uid58581
Streptococcus pneumoniae R6 uid57859
Streptococcus pneumoniae CGSP14 uid59181
Streptococcus pneumoniae INV200 uid162035
Streptococcus pneumoniae OXC141 uid162037
Streptococcus pneumoniae P1031 uid59123
Streptococcus pneumoniae INV104 uid162039
Streptococcus pneumoniae JJA uid59121
infB

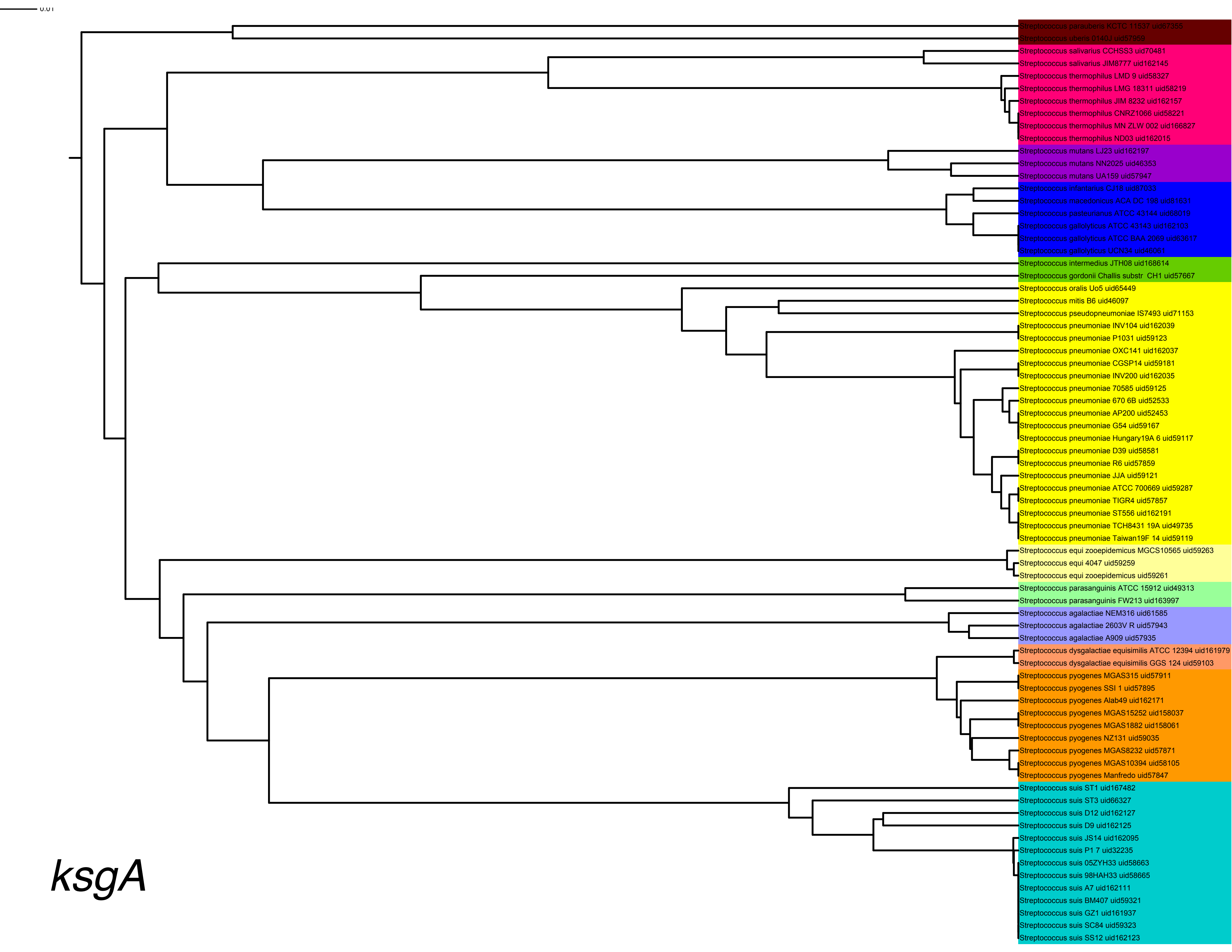
0.01
Streptococcus parauberis KCTC 11537 uid67355
Streptococcus uberis 0140J uid57959
Streptococcus salivarius CCHSS3 uid70481
Streptococcus salivarius JIM8777 uid162145
Streptococcus thermophilus LMD 9 uid58327
Streptococcus thermophilus LMG 18311 uid58219
Streptococcus thermophilus JIM 8232 uid162157
Streptococcus thermophilus CNRZ1066 uid58221
Streptococcus thermophilus MN ZLW 002 uid166827
Streptococcus thermophilus ND03 uid162015
Streptococcus mutans LJ23 uid162197
Streptococcus mutans NN2025 uid46353
Streptococcus mutans UA159 uid57947
Streptococcus infantarius CJ18 uid87033
Streptococcus macedonicus ACA DC 198 uid81631
Streptococcus pasteurianus ATCC 43144 uid68019
Streptococcus gallolyticus ATCC 43143 uid162103
Streptococcus gallolyticus ATCC BAA 2069 uid63617
Streptococcus gallolyticus UCN34 uid46061
Streptococcus intermedius JTH08 uid168614
Streptococcus gordonii Challis substr CH1 uid57667
Streptococcus oralis Uo5 uid65449
Streptococcus mitis B6 uid46097
Streptococcus pseudopneumoniae IS7493 uid71153
Streptococcus pneumoniae INV104 uid162039
Streptococcus pneumoniae P1031 uid59123
Streptococcus pneumoniae OXC141 uid162037
Streptococcus pneumoniae CGSP14 uid59181
Streptococcus pneumoniae INV200 uid162035
Streptococcus pneumoniae 70585 uid59125
Streptococcus pneumoniae 670 6B uid52533
Streptococcus pneumoniae AP200 uid52453
Streptococcus pneumoniae G54 uid59167
Streptococcus pneumoniae Hungary19A 6 uid59117
Streptococcus pneumoniae D39 uid58581
Streptococcus pneumoniae R6 uid57859
Streptococcus pneumoniae JJA uid59121
Streptococcus pneumoniae ATCC 700669 uid59287
Streptococcus pneumoniae TIGR4 uid57857
Streptococcus pneumoniae ST556 uid162191
Streptococcus pneumoniae TCH8431 19A uid49735
Streptococcus pneumoniae Taiwan19F 14 uid59119
Streptococcus equi zooepidemicus MGCS10565 uid59263
Streptococcus equi 4047 uid59259
Streptococcus equi zooepidemicus uid59261
Streptococcus parasanguinis ATCC 15912 uid49313
Streptococcus parasanguinis FW213 uid163997
Streptococcus agalactiae NEM316 uid61585
Streptococcus agalactiae 2603V R uid57943
Streptococcus agalactiae A909 uid57935
Streptococcus dysgalactiae equisimilis ATCC 12394 uid161979
Streptococcus dysgalactiae equisimilis GGS 124 uid59103
Streptococcus pyogenes MGAS315 uid57911
Streptococcus pyogenes SSI 1 uid57895
Streptococcus pyogenes Alab49 uid162171
Streptococcus pyogenes MGAS15252 uid158037
Streptococcus pyogenes MGAS1882 uid158061
Streptococcus pyogenes NZ131 uid59035
Streptococcus pyogenes MGAS8232 uid57871
Streptococcus pyogenes MGAS10394 uid58105
Streptococcus pyogenes Manfredo uid57847
Streptococcus suis ST1 uid167482
Streptococcus suis ST3 uid66327
Streptococcus suis D12 uid162127
Streptococcus suis D9 uid162125
Streptococcus suis JS14 uid162095
Streptococcus suis P1 7 uid32235
Streptococcus suis 05ZYH33 uid58663
Streptococcus suis 98HAH33 uid58665
Streptococcus suis A7 uid162111
Streptococcus suis BM407 uid59321
Streptococcus suis GZ1 uid161937
Streptococcus suis SC84 uid59323
Streptococcus suis SS12 uid162123
ksgA

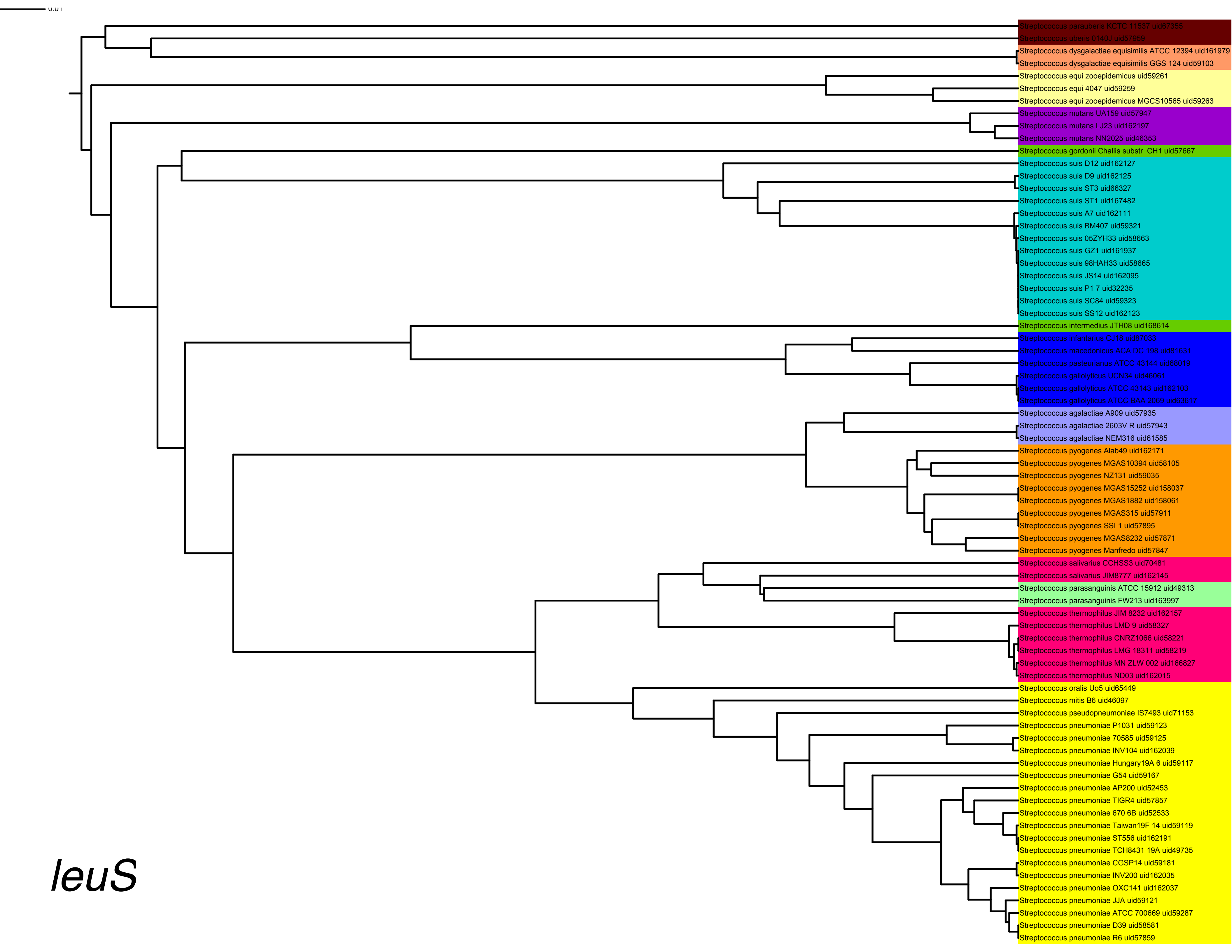
0.01
Streptococcus parauberis KCTC 11537 uid67355
Streptococcus uberis 0140J uid57959
Streptococcus dysgalactiae equisimilis ATCC 12394 uid161979
Streptococcus dysgalactiae equisimilis GGS 124 uid59103
Streptococcus equi zooepidemicus uid59261
Streptococcus equi 4047 uid59259
Streptococcus equi zooepidemicus MGCS10565 uid59263
Streptococcus mutans UA159 uid57947
Streptococcus mutans LJ23 uid162197
Streptococcus mutans NN2025 uid46353
Streptococcus gordonii Challis substr CH1 uid57667
Streptococcus suis D12 uid162127
Streptococcus suis D9 uid162125
Streptococcus suis ST3 uid66327
Streptococcus suis ST1 uid167482
Streptococcus suis A7 uid162111
Streptococcus suis BM407 uid59321
Streptococcus suis 05ZYH33 uid58663
Streptococcus suis GZ1 uid161937
Streptococcus suis 98HAH33 uid58665
Streptococcus suis JS14 uid162095
Streptococcus suis P1 7 uid32235
Streptococcus suis SC84 uid59323
Streptococcus suis SS12 uid162123
Streptococcus intermedius JTH08 uid168614
Streptococcus infantarius CJ18 uid87033
Streptococcus macedonicus ACA DC 198 uid81631
Streptococcus pasteurianus ATCC 43144 uid68019
Streptococcus gallolyticus UCN34 uid46061
Streptococcus gallolyticus ATCC 43143 uid162103
Streptococcus gallolyticus ATCC BAA 2069 uid63617
Streptococcus agalactiae A909 uid57935
Streptococcus agalactiae 2603V R uid57943
Streptococcus agalactiae NEM316 uid61585
Streptococcus pyogenes Alab49 uid162171
Streptococcus pyogenes MGAS10394 uid58105
Streptococcus pyogenes NZ131 uid59035
Streptococcus pyogenes MGAS15252 uid158037
Streptococcus pyogenes MGAS1882 uid158061
Streptococcus pyogenes MGAS315 uid57911
Streptococcus pyogenes SSI 1 uid57895
Streptococcus pyogenes MGAS8232 uid57871
Streptococcus pyogenes Manfredo uid57847
Streptococcus salivarius CCHSS3 uid70481
Streptococcus salivarius JIM8777 uid162145
Streptococcus parasanguinis ATCC 15912 uid49313
Streptococcus parasanguinis FW213 uid163997
Streptococcus thermophilus JIM 8232 uid162157
Streptococcus thermophilus LMD 9 uid58327
Streptococcus thermophilus CNRZ1066 uid58221
Streptococcus thermophilus LMG 18311 uid58219
Streptococcus thermophilus MN ZLW 002 uid166827
Streptococcus thermophilus ND03 uid162015
Streptococcus oralis Uo5 uid65449
Streptococcus mitis B6 uid46097
Streptococcus pseudopneumoniae IS7493 uid71153
Streptococcus pneumoniae P1031 uid59123
Streptococcus pneumoniae 70585 uid59125
Streptococcus pneumoniae INV104 uid162039
Streptococcus pneumoniae Hungary19A 6 uid59117
Streptococcus pneumoniae G54 uid59167
Streptococcus pneumoniae AP200 uid52453
Streptococcus pneumoniae TIGR4 uid57857
Streptococcus pneumoniae 670 6B uid52533
Streptococcus pneumoniae Taiwan19F 14 uid59119
Streptococcus pneumoniae ST556 uid162191
Streptococcus pneumoniae TCH8431 19A uid49735
Streptococcus pneumoniae CGSP14 uid59181
Streptococcus pneumoniae INV200 uid162035
Streptococcus pneumoniae OXC141 uid162037
Streptococcus pneumoniae JJA uid59121
Streptococcus pneumoniae ATCC 700669 uid59287
Streptococcus pneumoniae D39 uid58581
Streptococcus pneumoniae R6 uid57859
leuS

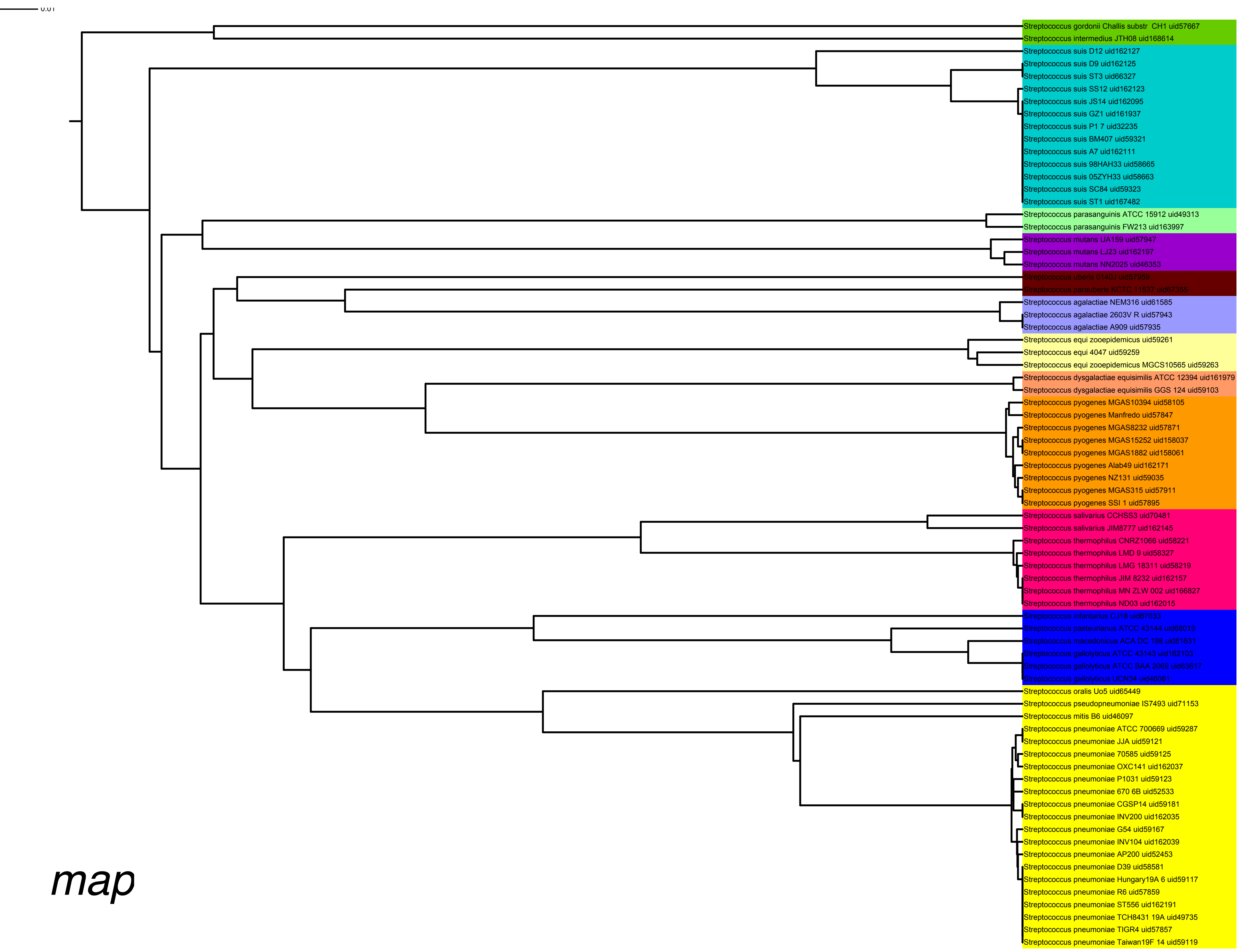
0.01
Streptococcus gordonii Challis substr CH1 uid57667
Streptococcus intermedius JTH08 uid168614
Streptococcus suis D12 uid162127
Streptococcus suis D9 uid162125
Streptococcus suis ST3 uid66327
Streptococcus suis SS12 uid162123
Streptococcus suis JS14 uid162095
Streptococcus suis GZ1 uid161937
Streptococcus suis P1 7 uid32235
Streptococcus suis BM407 uid59321
Streptococcus suis A7 uid162111
Streptococcus suis 98HAH33 uid58665
Streptococcus suis 05ZYH33 uid58663
Streptococcus suis SC84 uid59323
Streptococcus suis ST1 uid167482
Streptococcus parasanguinis ATCC 15912 uid49313
Streptococcus parasanguinis FW213 uid163997
Streptococcus mutans UA159 uid57947
Streptococcus mutans LJ23 uid162197
Streptococcus mutans NN2025 uid46353
Streptococcus uberis 0140J uid57959
Streptococcus parauberis KCTC 11537 uid67355
Streptococcus agalactiae NEM316 uid61585
Streptococcus agalactiae 2603V R uid57943
Streptococcus agalactiae A909 uid57935
Streptococcus equi zooepidemicus uid59261
Streptococcus equi 4047 uid59259
Streptococcus equi zooepidemicus MGCS10565 uid59263
Streptococcus dysgalactiae equisimilis ATCC 12394 uid161979
Streptococcus dysgalactiae equisimilis GGS 124 uid59103
Streptococcus pyogenes MGAS10394 uid58105
Streptococcus pyogenes Manfredo uid57847
Streptococcus pyogenes MGAS8232 uid57871
Streptococcus pyogenes MGAS15252 uid158037
Streptococcus pyogenes MGAS1882 uid158061
Streptococcus pyogenes Alab49 uid162171
Streptococcus pyogenes NZ131 uid59035
Streptococcus pyogenes MGAS315 uid57911
Streptococcus pyogenes SSI 1 uid57895
Streptococcus salivarius CCHSS3 uid70481
Streptococcus salivarius JIM8777 uid162145
Streptococcus thermophilus CNRZ1066 uid58221
Streptococcus thermophilus LMD 9 uid58327
Streptococcus thermophilus LMG 18311 uid58219
Streptococcus thermophilus JIM 8232 uid162157
Streptococcus thermophilus MN ZLW 002 uid166827
Streptococcus thermophilus ND03 uid162015
Streptococcus infantarius CJ18 uid87033
Streptococcus pasteurianus ATCC 43144 uid68019
Streptococcus macedonicus ACA DC 198 uid81631
Streptococcus gallolyticus ATCC 43143 uid162103
Streptococcus gallolyticus ATCC BAA 2069 uid63617
Streptococcus gallolyticus UCN34 uid46061
Streptococcus oralis Uo5 uid65449
Streptococcus pseudopneumoniae IS7493 uid71153
Streptococcus mitis B6 uid46097
Streptococcus pneumoniae ATCC 700669 uid59287
Streptococcus pneumoniae JJA uid59121
Streptococcus pneumoniae 70585 uid59125
Streptococcus pneumoniae OXC141 uid162037
Streptococcus pneumoniae P1031 uid59123
Streptococcus pneumoniae 670 6B uid52533
Streptococcus pneumoniae CGSP14 uid59181
Streptococcus pneumoniae INV200 uid162035
Streptococcus pneumoniae G54 uid59167
Streptococcus pneumoniae INV104 uid162039
Streptococcus pneumoniae AP200 uid52453
Streptococcus pneumoniae D39 uid58581
Streptococcus pneumoniae Hungary19A 6 uid59117
Streptococcus pneumoniae R6 uid57859
Streptococcus pneumoniae ST556 uid162191
Streptococcus pneumoniae TCH8431 19A uid49735
Streptococcus pneumoniae TIGR4 uid57857
Streptococcus pneumoniae Taiwan19F 14 uid59119
map

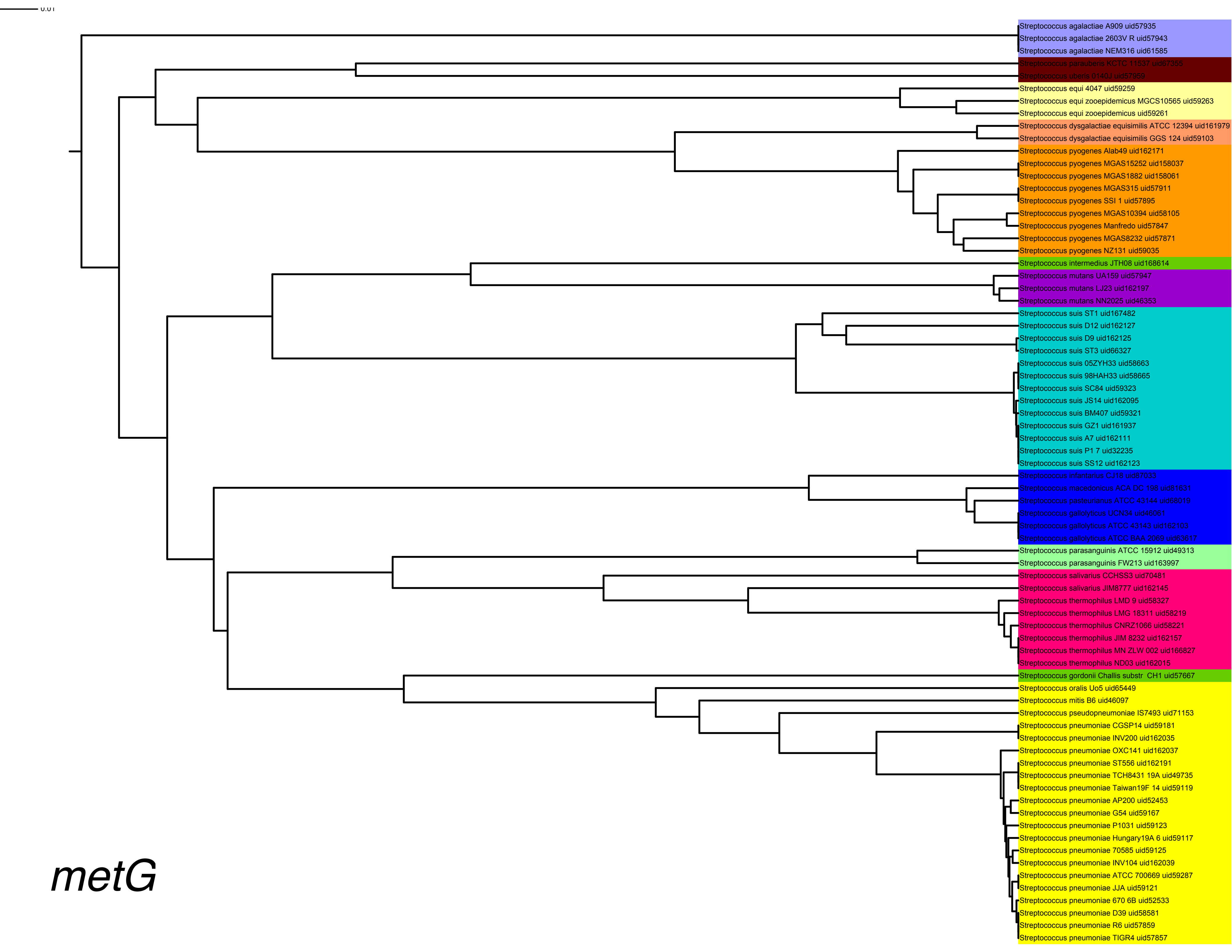
0.01
Streptococcus agalactiae A909 uid57935
Streptococcus agalactiae 2603V R uid57943
Streptococcus agalactiae NEM316 uid61585
Streptococcus parauberis KCTC 11537 uid67355
Streptococcus uberis 0140J uid57959
Streptococcus equi 4047 uid59259
Streptococcus equi zooepidemicus MGCS10565 uid59263
Streptococcus equi zooepidemicus uid59261
Streptococcus dysgalactiae equisimilis ATCC 12394 uid161979
Streptococcus dysgalactiae equisimilis GGS 124 uid59103
Streptococcus pyogenes Alab49 uid162171
Streptococcus pyogenes MGAS15252 uid158037
Streptococcus pyogenes MGAS1882 uid158061
Streptococcus pyogenes MGAS315 uid57911
Streptococcus pyogenes SSI 1 uid57895
Streptococcus pyogenes MGAS10394 uid58105
Streptococcus pyogenes Manfredo uid57847
Streptococcus pyogenes MGAS8232 uid57871
Streptococcus pyogenes NZ131 uid59035
Streptococcus intermedius JTH08 uid168614
Streptococcus mutans UA159 uid57947
Streptococcus mutans LJ23 uid162197
Streptococcus mutans NN2025 uid46353
Streptococcus suis ST1 uid167482
Streptococcus suis D12 uid162127
Streptococcus suis D9 uid162125
Streptococcus suis ST3 uid66327
Streptococcus suis 05ZYH33 uid58663
Streptococcus suis 98HAH33 uid58665
Streptococcus suis SC84 uid59323
Streptococcus suis JS14 uid162095
Streptococcus suis BM407 uid59321
Streptococcus suis GZ1 uid161937
Streptococcus suis A7 uid162111
Streptococcus suis P1 7 uid32235
Streptococcus suis SS12 uid162123
Streptococcus infantarius CJ18 uid87033
Streptococcus macedonicus ACA DC 198 uid81631
Streptococcus pasteurianus ATCC 43144 uid68019
Streptococcus gallolyticus UCN34 uid46061
Streptococcus gallolyticus ATCC 43143 uid162103
Streptococcus gallolyticus ATCC BAA 2069 uid63617
Streptococcus parasanguinis ATCC 15912 uid49313
Streptococcus parasanguinis FW213 uid163997
Streptococcus salivarius CCHSS3 uid70481
Streptococcus salivarius JIM8777 uid162145
Streptococcus thermophilus LMD 9 uid58327
Streptococcus thermophilus LMG 18311 uid58219
Streptococcus thermophilus CNRZ1066 uid58221
Streptococcus thermophilus JIM 8232 uid162157
Streptococcus thermophilus MN ZLW 002 uid166827
Streptococcus thermophilus ND03 uid162015
Streptococcus gordonii Challis substr CH1 uid57667
Streptococcus oralis Uo5 uid65449
Streptococcus mitis B6 uid46097
Streptococcus pseudopneumoniae IS7493 uid71153
Streptococcus pneumoniae CGSP14 uid59181
Streptococcus pneumoniae INV200 uid162035
Streptococcus pneumoniae OXC141 uid162037
Streptococcus pneumoniae ST556 uid162191
Streptococcus pneumoniae TCH8431 19A uid49735
Streptococcus pneumoniae Taiwan19F 14 uid59119
Streptococcus pneumoniae AP200 uid52453
Streptococcus pneumoniae G54 uid59167
Streptococcus pneumoniae P1031 uid59123
Streptococcus pneumoniae Hungary19A 6 uid59117
Streptococcus pneumoniae 70585 uid59125
Streptococcus pneumoniae INV104 uid162039
Streptococcus pneumoniae ATCC 700669 uid59287
Streptococcus pneumoniae JJA uid59121
Streptococcus pneumoniae 670 6B uid52533
Streptococcus pneumoniae D39 uid58581
Streptococcus pneumoniae R6 uid57859
Streptococcus pneumoniae TIGR4 uid57857
metG

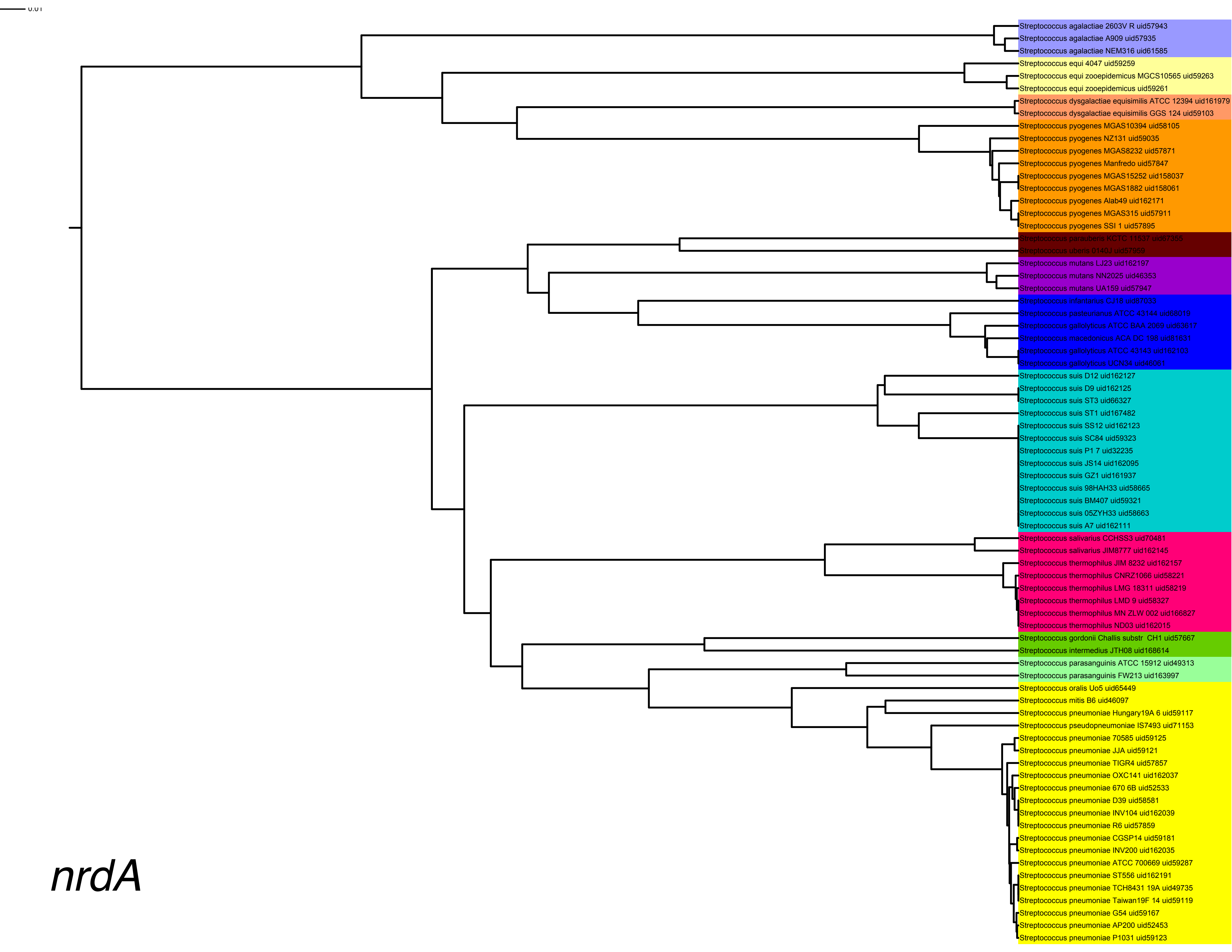
0.01
Streptococcus agalactiae 2603V R uid57943
Streptococcus agalactiae A909 uid57935
Streptococcus agalactiae NEM316 uid61585
Streptococcus equi 4047 uid59259
Streptococcus equi zooepidemicus MGCS10565 uid59263
Streptococcus equi zooepidemicus uid59261
Streptococcus dysgalactiae equisimilis ATCC 12394 uid161979
Streptococcus dysgalactiae equisimilis GGS 124 uid59103
Streptococcus pyogenes MGAS10394 uid58105
Streptococcus pyogenes NZ131 uid59035
Streptococcus pyogenes MGAS8232 uid57871
Streptococcus pyogenes Manfredo uid57847
Streptococcus pyogenes MGAS15252 uid158037
Streptococcus pyogenes MGAS1882 uid158061
Streptococcus pyogenes Alab49 uid162171
Streptococcus pyogenes MGAS315 uid57911
Streptococcus pyogenes SSI 1 uid57895
Streptococcus parauberis KCTC 11537 uid67355
Streptococcus uberis 0140J uid57959
Streptococcus mutans LJ23 uid162197
Streptococcus mutans NN2025 uid46353
Streptococcus mutans UA159 uid57947
Streptococcus infantarius CJ18 uid87033
Streptococcus pasteurianus ATCC 43144 uid68019
Streptococcus gallolyticus ATCC BAA 2069 uid63617
Streptococcus macedonicus ACA DC 198 uid81631
Streptococcus gallolyticus ATCC 43143 uid162103
Streptococcus gallolyticus UCN34 uid46061
Streptococcus suis D12 uid162127
Streptococcus suis D9 uid162125
Streptococcus suis ST3 uid66327
Streptococcus suis ST1 uid167482
Streptococcus suis SS12 uid162123
Streptococcus suis SC84 uid59323
Streptococcus suis P1 7 uid32235
Streptococcus suis JS14 uid162095
Streptococcus suis GZ1 uid161937
Streptococcus suis 98HAH33 uid58665
Streptococcus suis BM407 uid59321
Streptococcus suis 05ZYH33 uid58663
Streptococcus suis A7 uid162111
Streptococcus salivarius CCHSS3 uid70481
Streptococcus salivarius JIM8777 uid162145
Streptococcus thermophilus JIM 8232 uid162157
Streptococcus thermophilus CNRZ1066 uid58221
Streptococcus thermophilus LMG 18311 uid58219
Streptococcus thermophilus LMD 9 uid58327
Streptococcus thermophilus MN ZLW 002 uid166827
Streptococcus thermophilus ND03 uid162015
Streptococcus gordonii Challis substr CH1 uid57667
Streptococcus intermedius JTH08 uid168614
Streptococcus parasanguinis ATCC 15912 uid49313
Streptococcus parasanguinis FW213 uid163997
Streptococcus oralis Uo5 uid65449
Streptococcus mitis B6 uid46097
Streptococcus pneumoniae Hungary19A 6 uid59117
Streptococcus pseudopneumoniae IS7493 uid71153
Streptococcus pneumoniae 70585 uid59125
Streptococcus pneumoniae JJA uid59121
Streptococcus pneumoniae TIGR4 uid57857
Streptococcus pneumoniae OXC141 uid162037
Streptococcus pneumoniae 670 6B uid52533
Streptococcus pneumoniae D39 uid58581
Streptococcus pneumoniae INV104 uid162039
Streptococcus pneumoniae R6 uid57859
Streptococcus pneumoniae CGSP14 uid59181
Streptococcus pneumoniae INV200 uid162035
Streptococcus pneumoniae ATCC 700669 uid59287
Streptococcus pneumoniae ST556 uid162191
Streptococcus pneumoniae TCH8431 19A uid49735
Streptococcus pneumoniae Taiwan19F 14 uid59119
Streptococcus pneumoniae G54 uid59167
Streptococcus pneumoniae AP200 uid52453
Streptococcus pneumoniae P1031 uid59123
nrdA

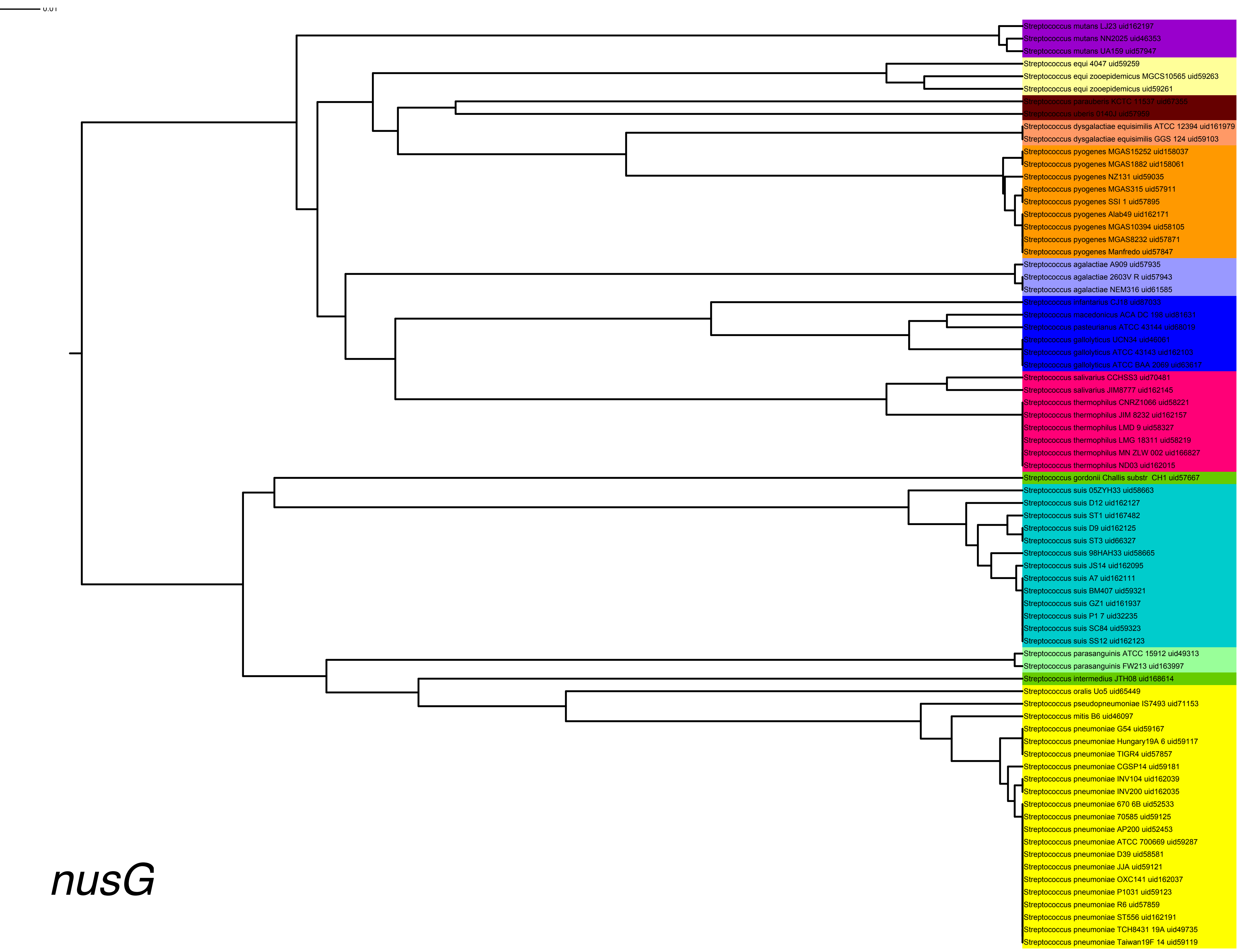
0.01
Streptococcus mutans LJ23 uid162197
Streptococcus mutans NN2025 uid46353
Streptococcus mutans UA159 uid57947
Streptococcus equi 4047 uid59259
Streptococcus equi zooepidemicus MGCS10565 uid59263
Streptococcus equi zooepidemicus uid59261
Streptococcus parauberis KCTC 11537 uid67355
Streptococcus uberis 0140J uid57959
Streptococcus dysgalactiae equisimilis ATCC 12394 uid161979
Streptococcus dysgalactiae equisimilis GGS 124 uid59103
Streptococcus pyogenes MGAS15252 uid158037
Streptococcus pyogenes MGAS1882 uid158061
Streptococcus pyogenes NZ131 uid59035
Streptococcus pyogenes MGAS315 uid57911
Streptococcus pyogenes SSI 1 uid57895
Streptococcus pyogenes Alab49 uid162171
Streptococcus pyogenes MGAS10394 uid58105
Streptococcus pyogenes MGAS8232 uid57871
Streptococcus pyogenes Manfredo uid57847
Streptococcus agalactiae A909 uid57935
Streptococcus agalactiae 2603V R uid57943
Streptococcus agalactiae NEM316 uid61585
Streptococcus infantarius CJ18 uid87033
Streptococcus macedonicus ACA DC 198 uid81631
Streptococcus pasteurianus ATCC 43144 uid68019
Streptococcus gallolyticus UCN34 uid46061
Streptococcus gallolyticus ATCC 43143 uid162103
Streptococcus gallolyticus ATCC BAA 2069 uid63617
Streptococcus salivarius CCHSS3 uid70481
Streptococcus salivarius JIM8777 uid162145
Streptococcus thermophilus CNRZ1066 uid58221
Streptococcus thermophilus JIM 8232 uid162157
Streptococcus thermophilus LMD 9 uid58327
Streptococcus thermophilus LMG 18311 uid58219
Streptococcus thermophilus MN ZLW 002 uid166827
Streptococcus thermophilus ND03 uid162015
Streptococcus gordonii Challis substr CH1 uid57667
Streptococcus suis 05ZYH33 uid58663
Streptococcus suis D12 uid162127
Streptococcus suis ST1 uid167482
Streptococcus suis D9 uid162125
Streptococcus suis ST3 uid66327
Streptococcus suis 98HAH33 uid58665
Streptococcus suis JS14 uid162095
Streptococcus suis A7 uid162111
Streptococcus suis BM407 uid59321
Streptococcus suis GZ1 uid161937
Streptococcus suis P1 7 uid32235
Streptococcus suis SC84 uid59323
Streptococcus suis SS12 uid162123
Streptococcus parasanguinis ATCC 15912 uid49313
Streptococcus parasanguinis FW213 uid163997
Streptococcus intermedius JTH08 uid168614
Streptococcus oralis Uo5 uid65449
Streptococcus pseudopneumoniae IS7493 uid71153
Streptococcus mitis B6 uid46097
Streptococcus pneumoniae G54 uid59167
Streptococcus pneumoniae Hungary19A 6 uid59117
Streptococcus pneumoniae TIGR4 uid57857
Streptococcus pneumoniae CGSP14 uid59181
Streptococcus pneumoniae INV104 uid162039
Streptococcus pneumoniae INV200 uid162035
Streptococcus pneumoniae 670 6B uid52533
Streptococcus pneumoniae 70585 uid59125
Streptococcus pneumoniae AP200 uid52453
Streptococcus pneumoniae ATCC 700669 uid59287
Streptococcus pneumoniae D39 uid58581
Streptococcus pneumoniae JJA uid59121
Streptococcus pneumoniae OXC141 uid162037
Streptococcus pneumoniae P1031 uid59123
Streptococcus pneumoniae R6 uid57859
Streptococcus pneumoniae ST556 uid162191
Streptococcus pneumoniae TCH8431 19A uid49735
Streptococcus pneumoniae Taiwan19F 14 uid59119
nusG

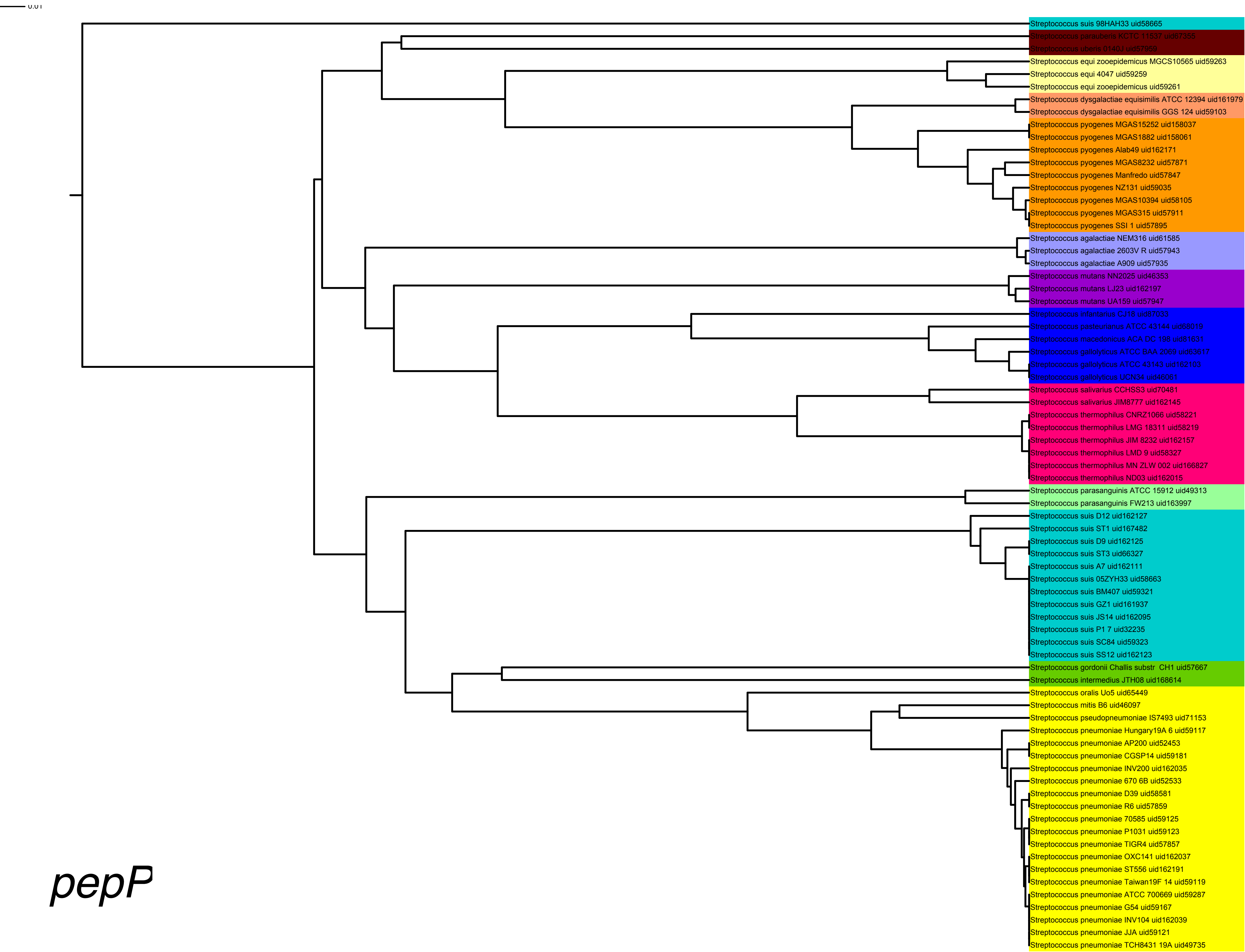
0.01
Streptococcus suis 98HAH33 uid58665
Streptococcus parauberis KCTC 11537 uid67355
Streptococcus uberis 0140J uid57959
Streptococcus equi zooepidemicus MGCS10565 uid59263
Streptococcus equi 4047 uid59259
Streptococcus equi zooepidemicus uid59261
Streptococcus dysgalactiae equisimilis ATCC 12394 uid161979
Streptococcus dysgalactiae equisimilis GGS 124 uid59103
Streptococcus pyogenes MGAS15252 uid158037
Streptococcus pyogenes MGAS1882 uid158061
Streptococcus pyogenes Alab49 uid162171
Streptococcus pyogenes MGAS8232 uid57871
Streptococcus pyogenes Manfredo uid57847
Streptococcus pyogenes NZ131 uid59035
Streptococcus pyogenes MGAS10394 uid58105
Streptococcus pyogenes MGAS315 uid57911
Streptococcus pyogenes SSI 1 uid57895
Streptococcus agalactiae NEM316 uid61585
Streptococcus agalactiae 2603V R uid57943
Streptococcus agalactiae A909 uid57935
Streptococcus mutans NN2025 uid46353
Streptococcus mutans LJ23 uid162197
Streptococcus mutans UA159 uid57947
Streptococcus infantarius CJ18 uid87033
Streptococcus pasteurianus ATCC 43144 uid68019
Streptococcus macedonicus ACA DC 198 uid81631
Streptococcus gallolyticus ATCC BAA 2069 uid63617
Streptococcus gallolyticus ATCC 43143 uid162103
Streptococcus gallolyticus UCN34 uid46061
Streptococcus salivarius CCHSS3 uid70481
Streptococcus salivarius JIM8777 uid162145
Streptococcus thermophilus CNRZ1066 uid58221
Streptococcus thermophilus LMG 18311 uid58219
Streptococcus thermophilus JIM 8232 uid162157
Streptococcus thermophilus LMD 9 uid58327
Streptococcus thermophilus MN ZLW 002 uid166827
Streptococcus thermophilus ND03 uid162015
Streptococcus parasanguinis ATCC 15912 uid49313
Streptococcus parasanguinis FW213 uid163997
Streptococcus suis D12 uid162127
Streptococcus suis ST1 uid167482
Streptococcus suis D9 uid162125
Streptococcus suis ST3 uid66327
Streptococcus suis A7 uid162111
Streptococcus suis 05ZYH33 uid58663
Streptococcus suis BM407 uid59321
Streptococcus suis GZ1 uid161937
Streptococcus suis JS14 uid162095
Streptococcus suis P1 7 uid32235
Streptococcus suis SC84 uid59323
Streptococcus suis SS12 uid162123
Streptococcus gordonii Challis substr CH1 uid57667
Streptococcus intermedius JTH08 uid168614
Streptococcus oralis Uo5 uid65449
Streptococcus mitis B6 uid46097
Streptococcus pseudopneumoniae IS7493 uid71153
Streptococcus pneumoniae Hungary19A 6 uid59117
Streptococcus pneumoniae AP200 uid52453
Streptococcus pneumoniae CGSP14 uid59181
Streptococcus pneumoniae INV200 uid162035
Streptococcus pneumoniae 670 6B uid52533
Streptococcus pneumoniae D39 uid58581
Streptococcus pneumoniae R6 uid57859
Streptococcus pneumoniae 70585 uid59125
Streptococcus pneumoniae P1031 uid59123
Streptococcus pneumoniae TIGR4 uid57857
Streptococcus pneumoniae OXC141 uid162037
Streptococcus pneumoniae ST556 uid162191
Streptococcus pneumoniae Taiwan19F 14 uid59119
Streptococcus pneumoniae ATCC 700669 uid59287
Streptococcus pneumoniae G54 uid59167
Streptococcus pneumoniae INV104 uid162039
Streptococcus pneumoniae JJA uid59121
Streptococcus pneumoniae TCH8431 19A uid49735
pepP

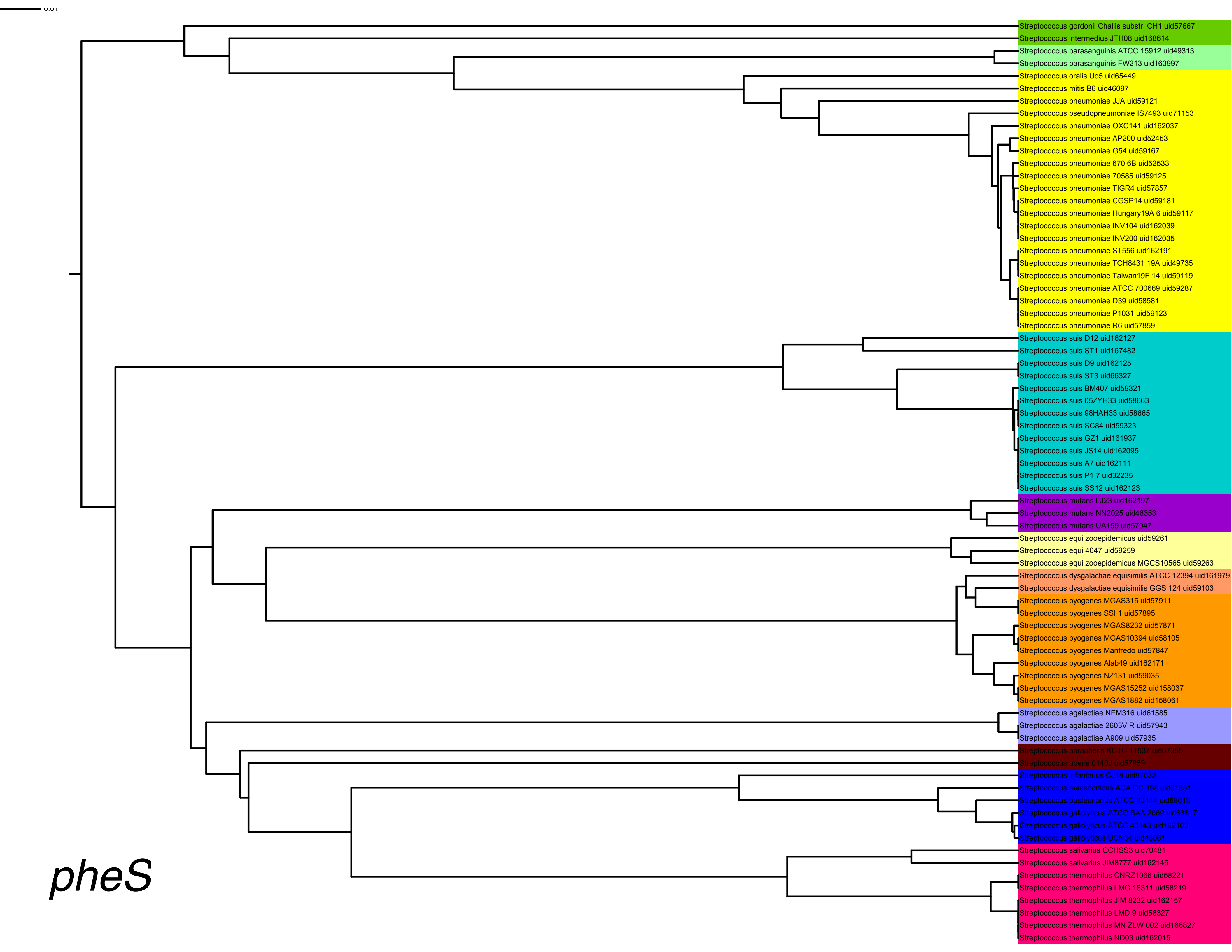

0.01
Streptococcus gordonii Challis substr CH1 uid57667
Streptococcus intermedius JTH08 uid168614
Streptococcus parasanguinis ATCC 15912 uid49313
Streptococcus parasanguinis FW213 uid163997
Streptococcus oralis Uo5 uid65449
Streptococcus mitis B6 uid46097
Streptococcus pneumoniae JJA uid59121
Streptococcus pseudopneumoniae IS7493 uid71153
Streptococcus pneumoniae OXC141 uid162037
Streptococcus pneumoniae AP200 uid52453
Streptococcus pneumoniae G54 uid59167
Streptococcus pneumoniae 670 6B uid52533
Streptococcus pneumoniae 70585 uid59125
Streptococcus pneumoniae TIGR4 uid57857
Streptococcus pneumoniae CGSP14 uid59181
Streptococcus pneumoniae Hungary19A 6 uid59117
Streptococcus pneumoniae INV104 uid162039
Streptococcus pneumoniae INV200 uid162035
Streptococcus pneumoniae ST556 uid162191
Streptococcus pneumoniae TCH8431 19A uid49735
Streptococcus pneumoniae Taiwan19F 14 uid59119
Streptococcus pneumoniae ATCC 700669 uid59287
Streptococcus pneumoniae D39 uid58581
Streptococcus pneumoniae P1031 uid59123
Streptococcus pneumoniae R6 uid57859
Streptococcus suis D12 uid162127
Streptococcus suis ST1 uid167482
Streptococcus suis D9 uid162125
Streptococcus suis ST3 uid66327
Streptococcus suis BM407 uid59321
Streptococcus suis 05ZYH33 uid58663
Streptococcus suis 98HAH33 uid58665
Streptococcus suis SC84 uid59323
Streptococcus suis GZ1 uid161937
Streptococcus suis JS14 uid162095
Streptococcus suis A7 uid162111
Streptococcus suis P1 7 uid32235
Streptococcus suis SS12 uid162123
Streptococcus mutans LJ23 uid162197
Streptococcus mutans NN2025 uid46353
Streptococcus mutans UA159 uid57947
Streptococcus equi zooepidemicus uid59261
Streptococcus equi 4047 uid59259
Streptococcus equi zooepidemicus MGCS10565 uid59263
Streptococcus dysgalactiae equisimilis ATCC 12394 uid161979
Streptococcus dysgalactiae equisimilis GGS 124 uid59103
Streptococcus pyogenes MGAS315 uid57911
Streptococcus pyogenes SSI 1 uid57895
Streptococcus pyogenes MGAS8232 uid57871
Streptococcus pyogenes MGAS10394 uid58105
Streptococcus pyogenes Manfredo uid57847
Streptococcus pyogenes Alab49 uid162171
Streptococcus pyogenes NZ131 uid59035
Streptococcus pyogenes MGAS15252 uid158037
Streptococcus pyogenes MGAS1882 uid158061
Streptococcus agalactiae NEM316 uid61585
Streptococcus agalactiae 2603V R uid57943
Streptococcus agalactiae A909 uid57935
Streptococcus parauberis KCTC 11537 uid67355
Streptococcus uberis 0140J uid57959
Streptococcus infantarius CJ18 uid87033
Streptococcus macedonicus ACA DC 198 uid81631
Streptococcus pasteurianus ATCC 43144 uid68019
Streptococcus gallolyticus ATCC BAA 2069 uid63617
Streptococcus gallolyticus ATCC 43143 uid162103
Streptococcus gallolyticus UCN34 uid46061
Streptococcus salivarius CCHSS3 uid70481
Streptococcus salivarius JIM8777 uid162145
Streptococcus thermophilus CNRZ1066 uid58221
Streptococcus thermophilus LMG 18311 uid58219
Streptococcus thermophilus JIM 8232 uid162157
Streptococcus thermophilus LMD 9 uid58327
Streptococcus thermophilus MN ZLW 002 uid166827
Streptococcus thermophilus ND03 uid162015
pheS

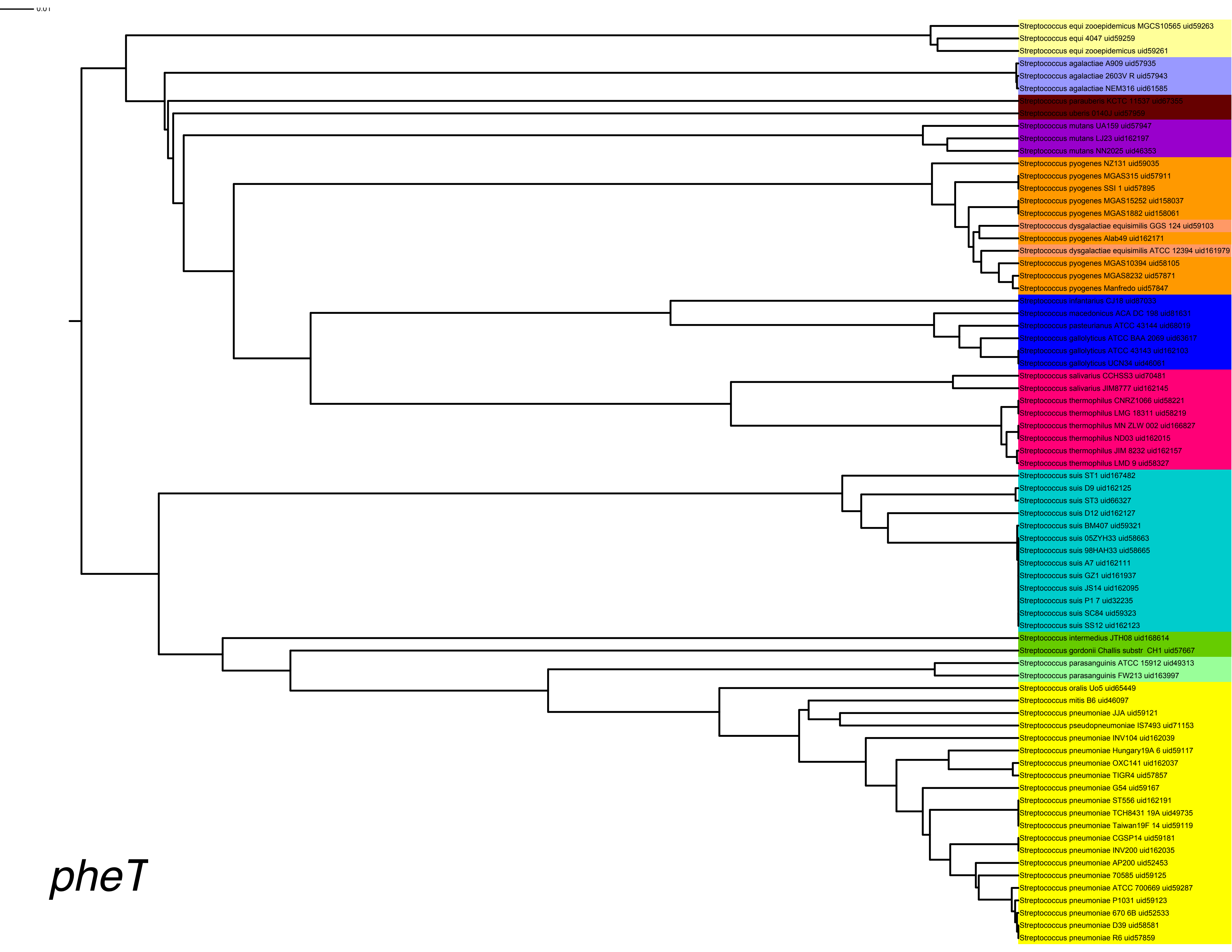
0.01
Streptococcus equi zooepidemicus MGCS10565 uid59263
Streptococcus equi 4047 uid59259
Streptococcus equi zooepidemicus uid59261
Streptococcus agalactiae A909 uid57935
Streptococcus agalactiae 2603V R uid57943
Streptococcus agalactiae NEM316 uid61585
Streptococcus parauberis KCTC 11537 uid67355
Streptococcus uberis 0140J uid57959
Streptococcus mutans UA159 uid57947
Streptococcus mutans LJ23 uid162197
Streptococcus mutans NN2025 uid46353
Streptococcus pyogenes NZ131 uid59035
Streptococcus pyogenes MGAS315 uid57911
Streptococcus pyogenes SSI 1 uid57895
Streptococcus pyogenes MGAS15252 uid158037
Streptococcus pyogenes MGAS1882 uid158061
Streptococcus dysgalactiae equisimilis GGS 124 uid59103
Streptococcus pyogenes Alab49 uid162171
Streptococcus dysgalactiae equisimilis ATCC 12394 uid161979
Streptococcus pyogenes MGAS10394 uid58105
Streptococcus pyogenes MGAS8232 uid57871
Streptococcus pyogenes Manfredo uid57847
Streptococcus infantarius CJ18 uid87033
Streptococcus macedonicus ACA DC 198 uid81631
Streptococcus pasteurianus ATCC 43144 uid68019
Streptococcus gallolyticus ATCC BAA 2069 uid63617
Streptococcus gallolyticus ATCC 43143 uid162103
Streptococcus gallolyticus UCN34 uid46061
Streptococcus salivarius CCHSS3 uid70481
Streptococcus salivarius JIM8777 uid162145
Streptococcus thermophilus CNRZ1066 uid58221
Streptococcus thermophilus LMG 18311 uid58219
Streptococcus thermophilus MN ZLW 002 uid166827
Streptococcus thermophilus ND03 uid162015
Streptococcus thermophilus JIM 8232 uid162157
Streptococcus thermophilus LMD 9 uid58327
Streptococcus suis ST1 uid167482
Streptococcus suis D9 uid162125
Streptococcus suis ST3 uid66327
Streptococcus suis D12 uid162127
Streptococcus suis BM407 uid59321
Streptococcus suis 05ZYH33 uid58663
Streptococcus suis 98HAH33 uid58665
Streptococcus suis A7 uid162111
Streptococcus suis GZ1 uid161937
Streptococcus suis JS14 uid162095
Streptococcus suis P1 7 uid32235
Streptococcus suis SC84 uid59323
Streptococcus suis SS12 uid162123
Streptococcus intermedius JTH08 uid168614
Streptococcus gordonii Challis substr CH1 uid57667
Streptococcus parasanguinis ATCC 15912 uid49313
Streptococcus parasanguinis FW213 uid163997
Streptococcus oralis Uo5 uid65449
Streptococcus mitis B6 uid46097
Streptococcus pneumoniae JJA uid59121
Streptococcus pseudopneumoniae IS7493 uid71153
Streptococcus pneumoniae INV104 uid162039
Streptococcus pneumoniae Hungary19A 6 uid59117
Streptococcus pneumoniae OXC141 uid162037
Streptococcus pneumoniae TIGR4 uid57857
Streptococcus pneumoniae G54 uid59167
Streptococcus pneumoniae ST556 uid162191
Streptococcus pneumoniae TCH8431 19A uid49735
Streptococcus pneumoniae Taiwan19F 14 uid59119
Streptococcus pneumoniae CGSP14 uid59181
Streptococcus pneumoniae INV200 uid162035
Streptococcus pneumoniae AP200 uid52453
Streptococcus pneumoniae 70585 uid59125
Streptococcus pneumoniae ATCC 700669 uid59287
Streptococcus pneumoniae P1031 uid59123
Streptococcus pneumoniae 670 6B uid52533
Streptococcus pneumoniae D39 uid58581
Streptococcus pneumoniae R6 uid57859
pheT

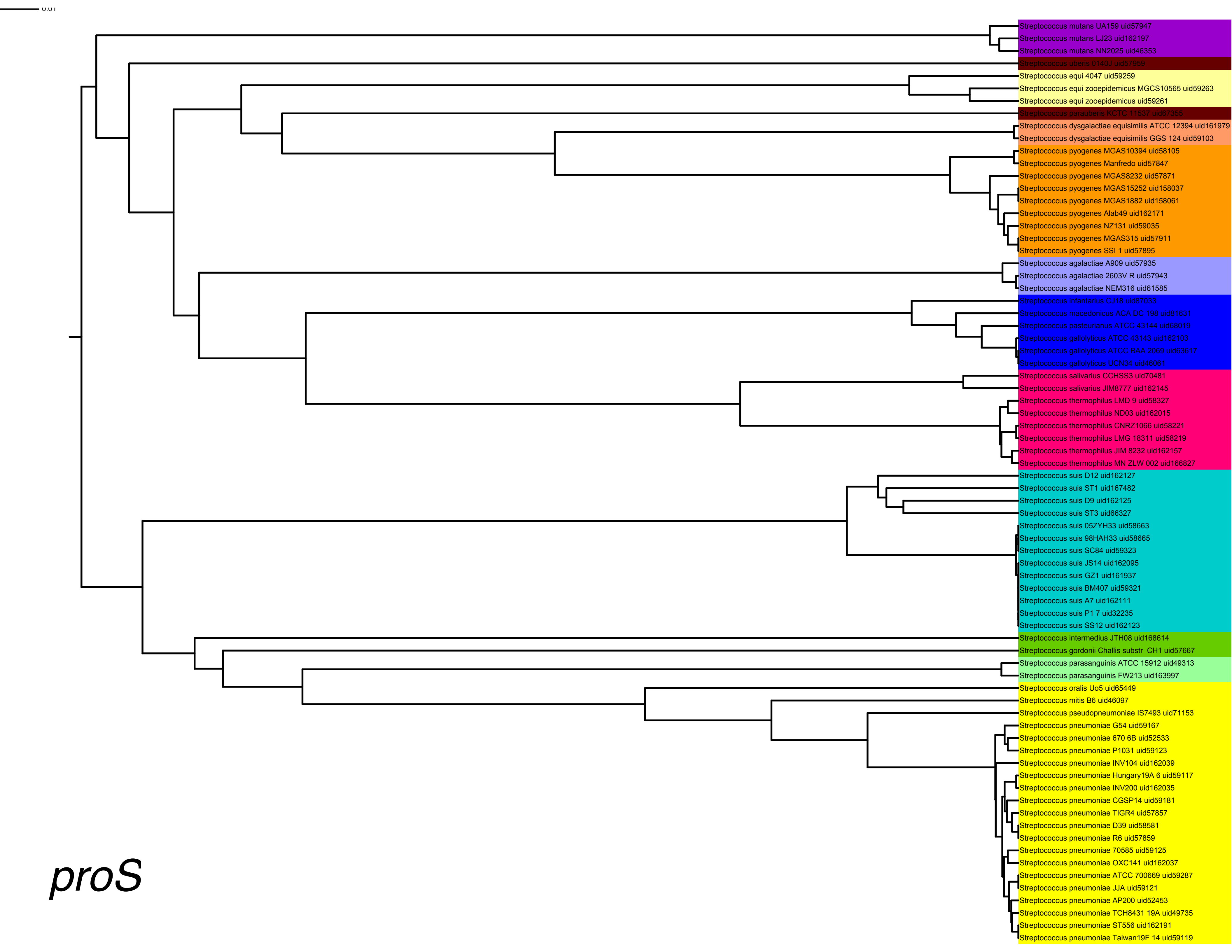
0.01
Streptococcus mutans UA159 uid57947
Streptococcus mutans LJ23 uid162197
Streptococcus mutans NN2025 uid46353
Streptococcus uberis 0140J uid57959
Streptococcus equi 4047 uid59259
Streptococcus equi zooepidemicus MGCS10565 uid59263
Streptococcus equi zooepidemicus uid59261
Streptococcus parauberis KCTC 11537 uid67355
Streptococcus dysgalactiae equisimilis ATCC 12394 uid161979
Streptococcus dysgalactiae equisimilis GGS 124 uid59103
Streptococcus pyogenes MGAS10394 uid58105
Streptococcus pyogenes Manfredo uid57847
Streptococcus pyogenes MGAS8232 uid57871
Streptococcus pyogenes MGAS15252 uid158037
Streptococcus pyogenes MGAS1882 uid158061
Streptococcus pyogenes Alab49 uid162171
Streptococcus pyogenes NZ131 uid59035
Streptococcus pyogenes MGAS315 uid57911
Streptococcus pyogenes SSI 1 uid57895
Streptococcus agalactiae A909 uid57935
Streptococcus agalactiae 2603V R uid57943
Streptococcus agalactiae NEM316 uid61585
Streptococcus infantarius CJ18 uid87033
Streptococcus macedonicus ACA DC 198 uid81631
Streptococcus pasteurianus ATCC 43144 uid68019
Streptococcus gallolyticus ATCC 43143 uid162103
Streptococcus gallolyticus ATCC BAA 2069 uid63617
Streptococcus gallolyticus UCN34 uid46061
Streptococcus salivarius CCHSS3 uid70481
Streptococcus salivarius JIM8777 uid162145
Streptococcus thermophilus LMD 9 uid58327
Streptococcus thermophilus ND03 uid162015
Streptococcus thermophilus CNRZ1066 uid58221
Streptococcus thermophilus LMG 18311 uid58219
Streptococcus thermophilus JIM 8232 uid162157
Streptococcus thermophilus MN ZLW 002 uid166827
Streptococcus suis D12 uid162127
Streptococcus suis ST1 uid167482
Streptococcus suis D9 uid162125
Streptococcus suis ST3 uid66327
Streptococcus suis 05ZYH33 uid58663
Streptococcus suis 98HAH33 uid58665
Streptococcus suis SC84 uid59323
Streptococcus suis JS14 uid162095
Streptococcus suis GZ1 uid161937
Streptococcus suis BM407 uid59321
Streptococcus suis A7 uid162111
Streptococcus suis P1 7 uid32235
Streptococcus suis SS12 uid162123
Streptococcus intermedius JTH08 uid168614
Streptococcus gordonii Challis substr CH1 uid57667
Streptococcus parasanguinis ATCC 15912 uid49313
Streptococcus parasanguinis FW213 uid163997
Streptococcus oralis Uo5 uid65449
Streptococcus mitis B6 uid46097
Streptococcus pseudopneumoniae IS7493 uid71153
Streptococcus pneumoniae G54 uid59167
Streptococcus pneumoniae 670 6B uid52533
Streptococcus pneumoniae P1031 uid59123
Streptococcus pneumoniae INV104 uid162039
Streptococcus pneumoniae Hungary19A 6 uid59117
Streptococcus pneumoniae INV200 uid162035
Streptococcus pneumoniae CGSP14 uid59181
Streptococcus pneumoniae TIGR4 uid57857
Streptococcus pneumoniae D39 uid58581
Streptococcus pneumoniae R6 uid57859
Streptococcus pneumoniae 70585 uid59125
Streptococcus pneumoniae OXC141 uid162037
Streptococcus pneumoniae ATCC 700669 uid59287
Streptococcus pneumoniae JJA uid59121
Streptococcus pneumoniae AP200 uid52453
Streptococcus pneumoniae TCH8431 19A uid49735
Streptococcus pneumoniae ST556 uid162191
Streptococcus pneumoniae Taiwan19F 14 uid59119
proS

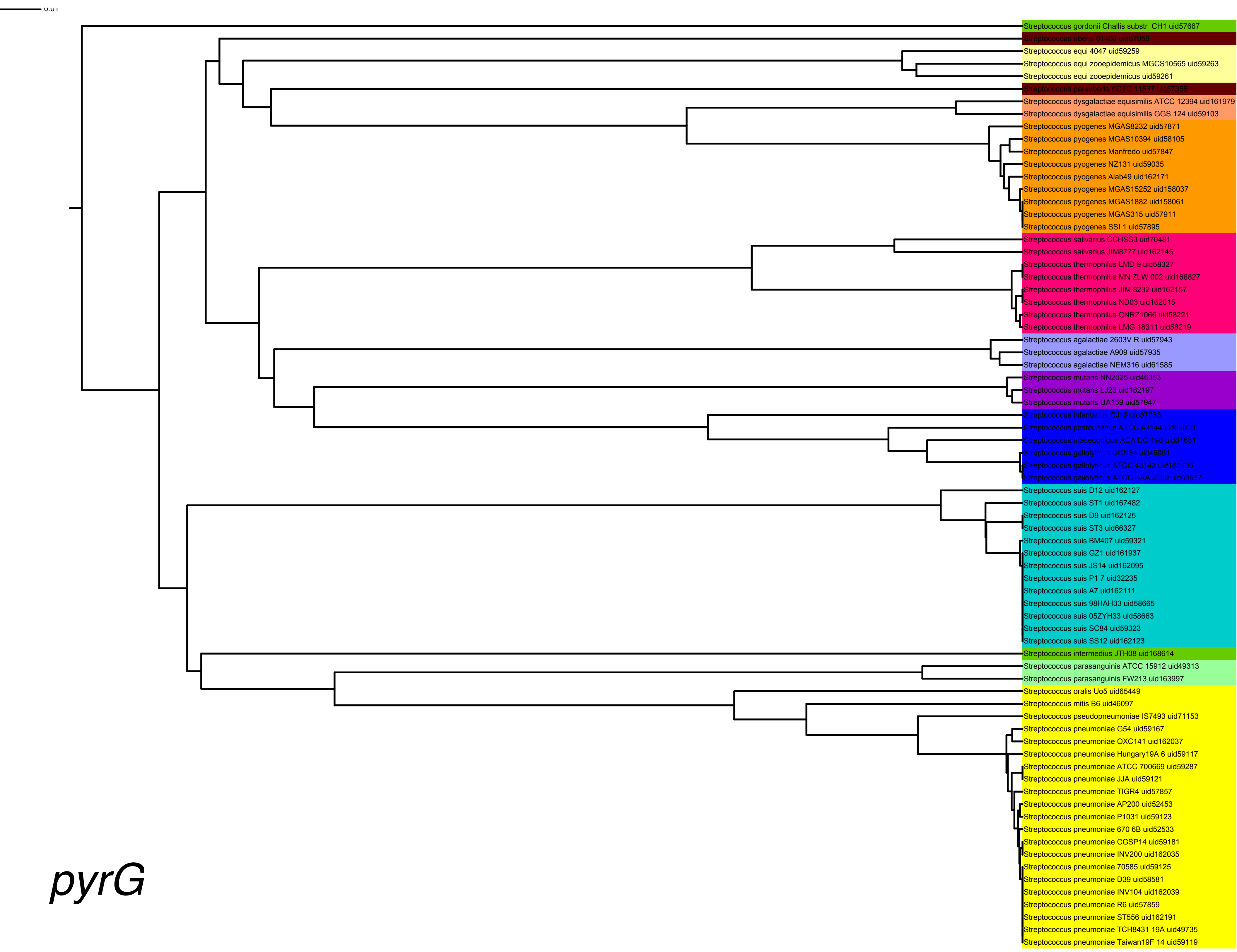
0.01
Streptococcus gordonii Challis substr CH1 uid57667
Streptococcus uberis 0140J uid57959
Streptococcus equi 4047 uid59259
Streptococcus equi zooepidemicus MGCS10565 uid59263
Streptococcus equi zooepidemicus uid59261
Streptococcus parauberis KCTC 11537 uid67355
Streptococcus dysgalactiae equisimilis ATCC 12394 uid161979
Streptococcus dysgalactiae equisimilis GGS 124 uid59103
Streptococcus pyogenes MGAS8232 uid57871
Streptococcus pyogenes MGAS10394 uid58105
Streptococcus pyogenes Manfredo uid57847
Streptococcus pyogenes NZ131 uid59035
Streptococcus pyogenes Alab49 uid162171
Streptococcus pyogenes MGAS15252 uid158037
Streptococcus pyogenes MGAS1882 uid158061
Streptococcus pyogenes MGAS315 uid57911
Streptococcus pyogenes SSI 1 uid57895
Streptococcus salivarius CCHSS3 uid70481
Streptococcus salivarius JIM8777 uid162145
Streptococcus thermophilus LMD 9 uid58327
Streptococcus thermophilus MN ZLW 002 uid166827
Streptococcus thermophilus JIM 8232 uid162157
Streptococcus thermophilus ND03 uid162015
Streptococcus thermophilus CNRZ1066 uid58221
Streptococcus thermophilus LMG 18311 uid58219
Streptococcus agalactiae 2603V R uid57943
Streptococcus agalactiae A909 uid57935
Streptococcus agalactiae NEM316 uid61585
Streptococcus mutans NN2025 uid46353
Streptococcus mutans LJ23 uid162197
Streptococcus mutans UA159 uid57947
Streptococcus infantarius CJ18 uid87033
Streptococcus pasteurianus ATCC 43144 uid68019
Streptococcus macedonicus ACA DC 198 uid81631
Streptococcus gallolyticus UCN34 uid46061
Streptococcus gallolyticus ATCC 43143 uid162103
Streptococcus gallolyticus ATCC BAA 2069 uid63617
Streptococcus suis D12 uid162127
Streptococcus suis ST1 uid167482
Streptococcus suis D9 uid162125
Streptococcus suis ST3 uid66327
Streptococcus suis BM407 uid59321
Streptococcus suis GZ1 uid161937
Streptococcus suis JS14 uid162095
Streptococcus suis P1 7 uid32235
Streptococcus suis A7 uid162111
Streptococcus suis 98HAH33 uid58665
Streptococcus suis 05ZYH33 uid58663
Streptococcus suis SC84 uid59323
Streptococcus suis SS12 uid162123
Streptococcus intermedius JTH08 uid168614
Streptococcus parasanguinis ATCC 15912 uid49313
Streptococcus parasanguinis FW213 uid163997
Streptococcus oralis Uo5 uid65449
Streptococcus mitis B6 uid46097
Streptococcus pseudopneumoniae IS7493 uid71153
Streptococcus pneumoniae G54 uid59167
Streptococcus pneumoniae OXC141 uid162037
Streptococcus pneumoniae Hungary19A 6 uid59117
Streptococcus pneumoniae ATCC 700669 uid59287
Streptococcus pneumoniae JJA uid59121
Streptococcus pneumoniae TIGR4 uid57857
Streptococcus pneumoniae AP200 uid52453
Streptococcus pneumoniae P1031 uid59123
Streptococcus pneumoniae 670 6B uid52533
Streptococcus pneumoniae CGSP14 uid59181
Streptococcus pneumoniae INV200 uid162035
Streptococcus pneumoniae 70585 uid59125
Streptococcus pneumoniae D39 uid58581
Streptococcus pneumoniae INV104 uid162039
Streptococcus pneumoniae R6 uid57859
Streptococcus pneumoniae ST556 uid162191
Streptococcus pneumoniae TCH8431 19A uid49735
Streptococcus pneumoniae Taiwan19F 14 uid59119
pyrG

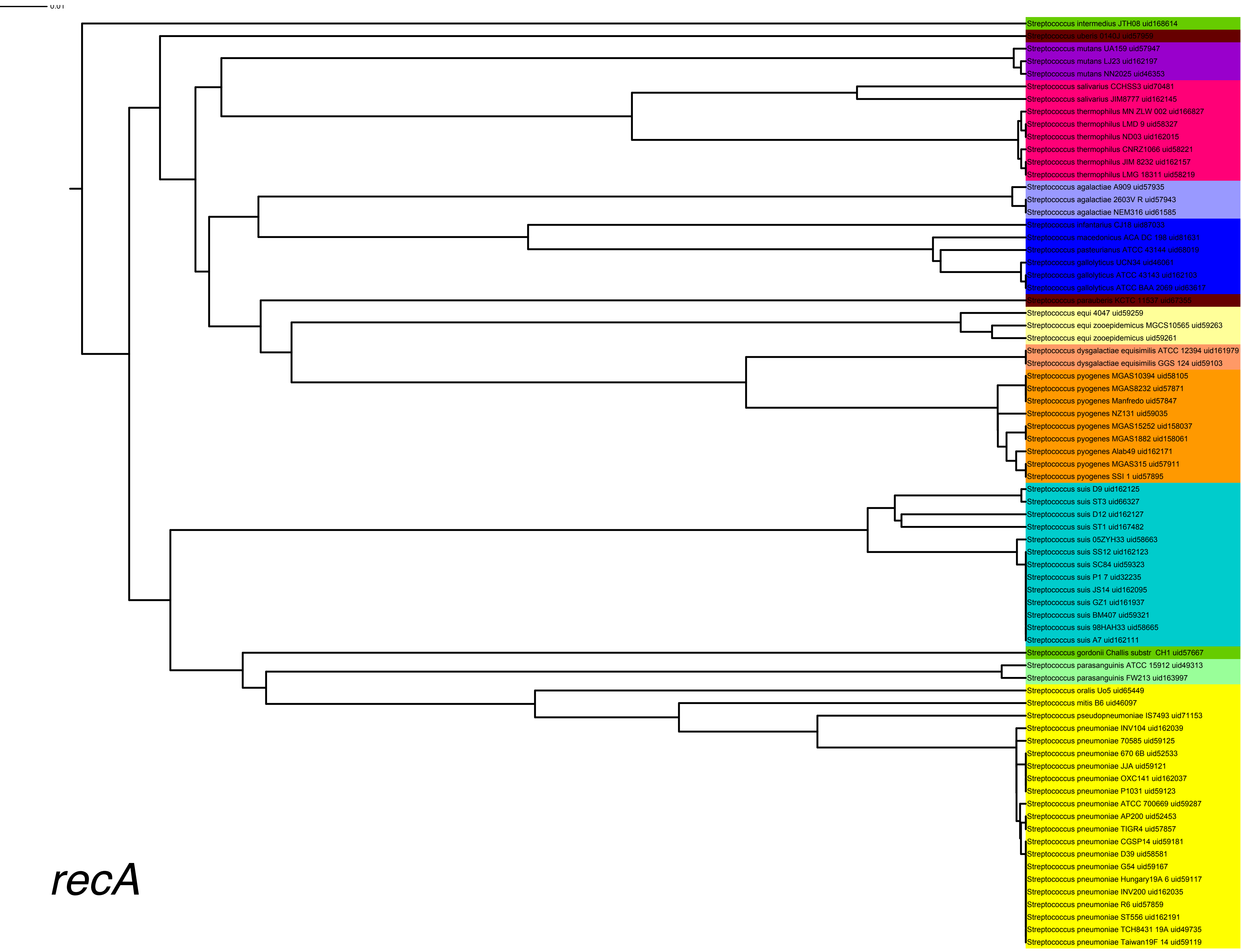
0.01
Streptococcus intermedius JTH08 uid168614
Streptococcus uberis 0140J uid57959
Streptococcus mutans UA159 uid57947
Streptococcus mutans LJ23 uid162197
Streptococcus mutans NN2025 uid46353
Streptococcus salivarius CCHSS3 uid70481
Streptococcus salivarius JIM8777 uid162145
Streptococcus thermophilus MN ZLW 002 uid166827
Streptococcus thermophilus LMD 9 uid58327
Streptococcus thermophilus ND03 uid162015
Streptococcus thermophilus CNRZ1066 uid58221
Streptococcus thermophilus JIM 8232 uid162157
Streptococcus thermophilus LMG 18311 uid58219
Streptococcus agalactiae A909 uid57935
Streptococcus agalactiae 2603V R uid57943
Streptococcus agalactiae NEM316 uid61585
Streptococcus infantarius CJ18 uid87033
Streptococcus macedonicus ACA DC 198 uid81631
Streptococcus pasteurianus ATCC 43144 uid68019
Streptococcus gallolyticus UCN34 uid46061
Streptococcus gallolyticus ATCC 43143 uid162103
Streptococcus gallolyticus ATCC BAA 2069 uid63617
Streptococcus parauberis KCTC 11537 uid87355
Streptococcus equi 4047 uid59259
Streptococcus equi zooepidemicus MGCS10565 uid59263
Streptococcus equi zooepidemicus uid59261
Streptococcus dysgalactiae equisimilis ATCC 12394 uid161979
Streptococcus dysgalactiae equisimilis GGS 124 uid59103
Streptococcus pyogenes MGAS10394 uid58105
Streptococcus pyogenes MGAS8232 uid57871
Streptococcus pyogenes Manfredo uid57847
Streptococcus pyogenes NZ131 uid59035
Streptococcus pyogenes MGAS15252 uid158037
Streptococcus pyogenes MGAS1882 uid158061
Streptococcus pyogenes Alab49 uid162171
Streptococcus pyogenes MGAS315 uid57911
Streptococcus pyogenes SSI 1 uid57895
Streptococcus suis D9 uid162125
Streptococcus suis ST3 uid66327
Streptococcus suis D12 uid162127
Streptococcus suis ST1 uid167482
Streptococcus suis 05ZYH33 uid58663
Streptococcus suis SS12 uid162123
Streptococcus suis SC84 uid59323
Streptococcus suis P1 7 uid32235
Streptococcus suis JS14 uid162095
Streptococcus suis GZ1 uid161937
Streptococcus suis BM407 uid59321
Streptococcus suis 98HAH33 uid58665
Streptococcus suis A7 uid162111
Streptococcus gordonii Challis substr CH1 uid57667
Streptococcus parasanguinis ATCC 15912 uid49313
Streptococcus parasanguinis FW213 uid163997
Streptococcus oralis Uo5 uid65449
Streptococcus mitis B6 uid46097
Streptococcus pseudopneumoniae IS7493 uid71153
Streptococcus pneumoniae INV104 uid162039
Streptococcus pneumoniae 70585 uid59125
Streptococcus pneumoniae 670 6B uid52533
Streptococcus pneumoniae JJA uid59121
Streptococcus pneumoniae OXC141 uid162037
Streptococcus pneumoniae P1031 uid59123
Streptococcus pneumoniae ATCC 700669 uid59287
Streptococcus pneumoniae AP200 uid52453
Streptococcus pneumoniae TIGR4 uid57857
Streptococcus pneumoniae CGSP14 uid59181
Streptococcus pneumoniae D39 uid58581
Streptococcus pneumoniae G54 uid59167
Streptococcus pneumoniae Hungary19A 6 uid59117
Streptococcus pneumoniae INV200 uid162035
Streptococcus pneumoniae R6 uid57859
Streptococcus pneumoniae ST556 uid162191
Streptococcus pneumoniae TCH8431 19A uid49735
Streptococcus pneumoniae Taiwan19F 14 uid59119
recA

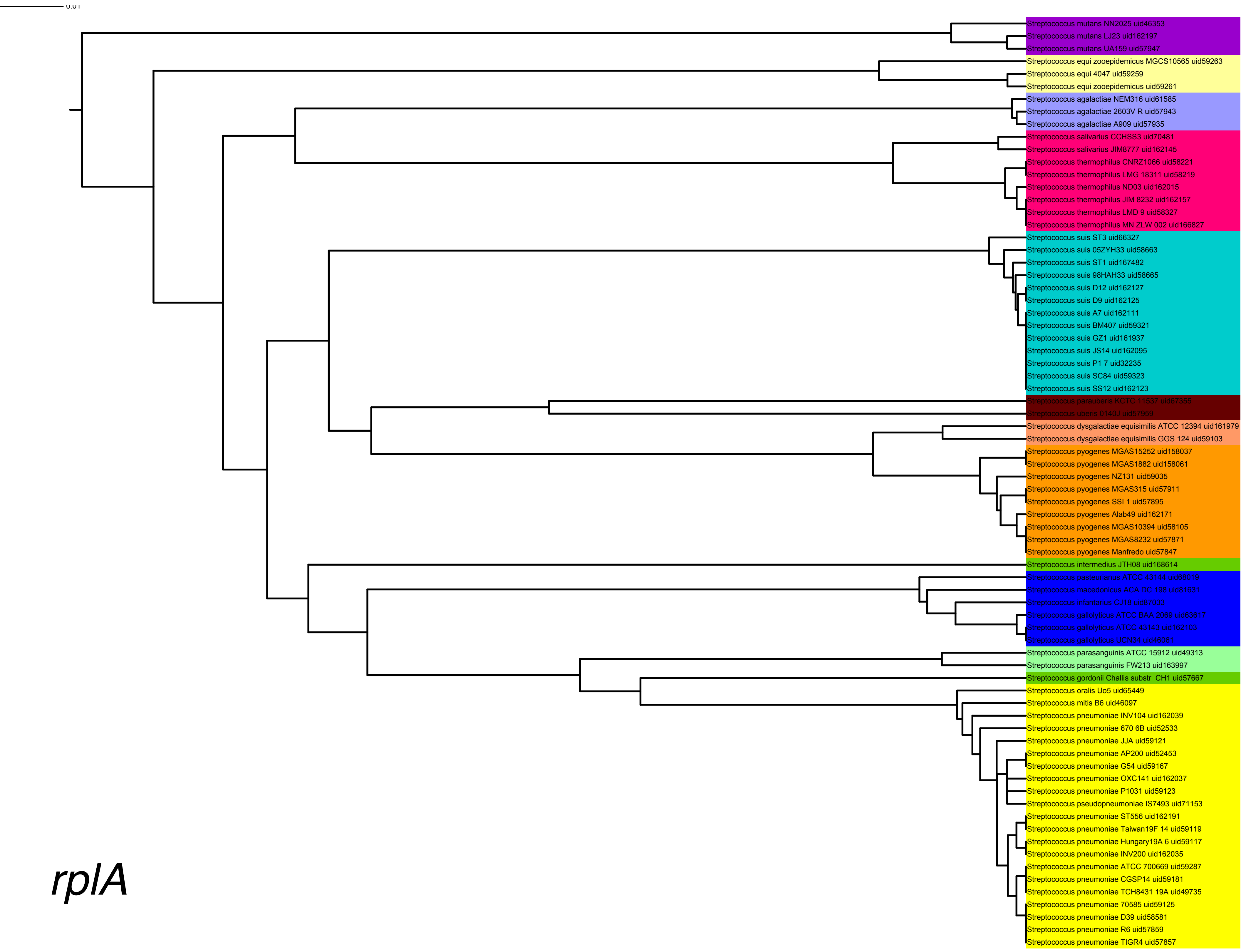

0.01
Streptococcus mutans NN2025 uid46353
Streptococcus mutans LJ23 uid162197
Streptococcus mutans UA159 uid57947
Streptococcus equi zooepidemicus MGCS10565 uid59263
Streptococcus equi 4047 uid59259
Streptococcus equi zooepidemicus uid59261
Streptococcus agalactiae NEM316 uid61585
Streptococcus agalactiae 2603V R uid57943
Streptococcus agalactiae A909 uid57935
Streptococcus salivarius CCHSS3 uid70481
Streptococcus salivarius JIM8777 uid162145
Streptococcus thermophilus CNRZ1066 uid58221
Streptococcus thermophilus LMG 18311 uid58219
Streptococcus thermophilus ND03 uid162015
Streptococcus thermophilus JIM 8232 uid162157
Streptococcus thermophilus LMD 9 uid58327
Streptococcus thermophilus MN ZLW 002 uid166827
Streptococcus suis ST3 uid66327
Streptococcus suis 05ZYH33 uid58663
Streptococcus suis ST1 uid167482
Streptococcus suis 98HAH33 uid58665
Streptococcus suis D12 uid162127
Streptococcus suis D9 uid162125
Streptococcus suis A7 uid162111
Streptococcus suis BM407 uid59321
Streptococcus suis GZ1 uid161937
Streptococcus suis JS14 uid162095
Streptococcus suis P1 7 uid32235
Streptococcus suis SC84 uid59323
Streptococcus suis SS12 uid162123
Streptococcus parauberis KCTC 11537 uid67355
Streptococcus uberis 0140J uid57959
Streptococcus dysgalactiae equisimilis ATCC 12394 uid161979
Streptococcus dysgalactiae equisimilis GGS 124 uid59103
Streptococcus pyogenes MGAS15252 uid158037
Streptococcus pyogenes MGAS1882 uid158061
Streptococcus pyogenes NZ131 uid59035
Streptococcus pyogenes MGAS315 uid57911
Streptococcus pyogenes SSI 1 uid57895
Streptococcus pyogenes Alab49 uid162171
Streptococcus pyogenes MGAS10394 uid58105
Streptococcus pyogenes MGAS8232 uid57871
Streptococcus pyogenes Manfredo uid57847
Streptococcus intermedius JTH08 uid168614
Streptococcus pasteurianus ATCC 43144 uid68019
Streptococcus macedonicus ACA DC 198 uid81631
Streptococcus infantarius CJ18 uid87033
Streptococcus gallolyticus ATCC BAA 2069 uid63617
Streptococcus gallolyticus ATCC 43143 uid162103
Streptococcus gallolyticus UCN34 uid46061
Streptococcus parasanguinis ATCC 15912 uid49313
Streptococcus parasanguinis FW213 uid163997
Streptococcus gordonii Challis substr CH1 uid57667
Streptococcus oralis Uo5 uid65449
Streptococcus mitis B6 uid46097
Streptococcus pneumoniae INV104 uid162039
Streptococcus pneumoniae 670 6B uid52533
Streptococcus pneumoniae JJA uid59121
Streptococcus pneumoniae AP200 uid52453
Streptococcus pneumoniae G54 uid59167
Streptococcus pneumoniae OXC141 uid162037
Streptococcus pneumoniae P1031 uid59123
Streptococcus pseudopneumoniae IS7493 uid71153
Streptococcus pneumoniae ST556 uid162191
Streptococcus pneumoniae Taiwan19F 14 uid59119
Streptococcus pneumoniae Hungary19A 6 uid59117
Streptococcus pneumoniae INV200 uid162035
Streptococcus pneumoniae ATCC 700669 uid59287
Streptococcus pneumoniae CGSP14 uid59181
Streptococcus pneumoniae TCH8431 19A uid49735
Streptococcus pneumoniae 70585 uid59125
Streptococcus pneumoniae D39 uid58581
Streptococcus pneumoniae R6 uid57859
Streptococcus pneumoniae TIGR4 uid57857
rplA

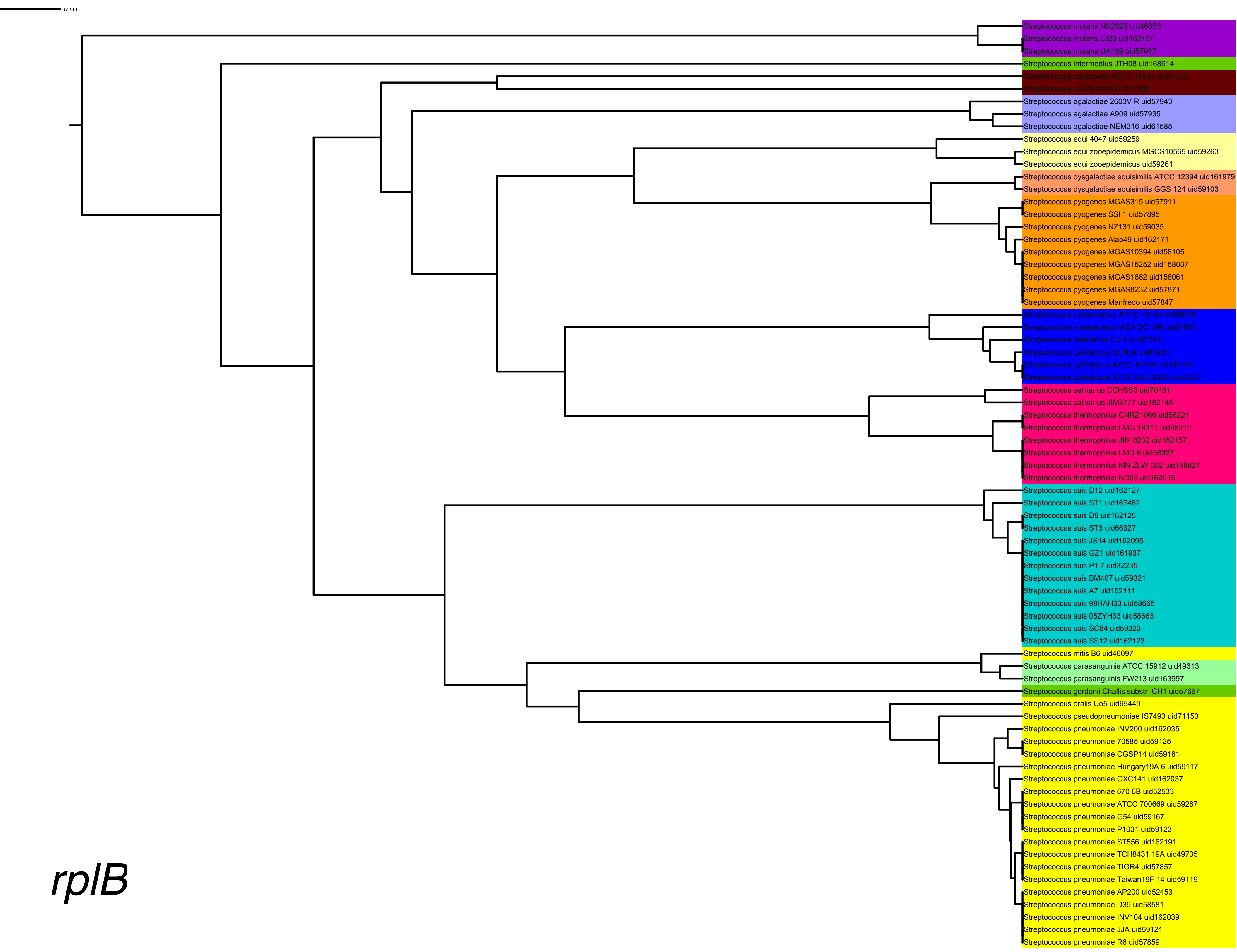
0.01
Streptococcus mutans NN2025 uid46353
Streptococcus mutans LJ23 uid162197
Streptococcus mutans UA159 uid57947
Streptococcus intermedius JTH08 uid168614
Streptococcus parauberis KCTC 11537 uid67355
Streptococcus uberis 0140J uid57959
Streptococcus agalactiae 2603V R uid57943
Streptococcus agalactiae A909 uid57935
Streptococcus agalactiae NEM316 uid61585
Streptococcus equi 4047 uid59259
Streptococcus equi zooepidemicus MGCS10565 uid59263
Streptococcus equi zooepidemicus uid59261
Streptococcus dysgalactiae equisimilis ATCC 12394 uid161979
Streptococcus dysgalactiae equisimilis GGS 124 uid59103
Streptococcus pyogenes MGAS315 uid57911
Streptococcus pyogenes SSI 1 uid57895
Streptococcus pyogenes NZ131 uid59035
Streptococcus pyogenes Alab49 uid162171
Streptococcus pyogenes MGAS10394 uid58105
Streptococcus pyogenes MGAS15252 uid158037
Streptococcus pyogenes MGAS1882 uid158061
Streptococcus pyogenes MGAS8232 uid57871
Streptococcus pyogenes Manfredo uid57847
Streptococcus pasteurianus ATCC 43144 uid68019
Streptococcus macedonicus ACA DC 198 uid81631
Streptococcus infantarius CJ18 uid87033
Streptococcus gallolyticus UCN34 uid46061
Streptococcus gallolyticus ATCC 43143 uid162103
Streptococcus gallolyticus ATCC BAA 2069 uid63617
Streptococcus salivarius CCHSS3 uid70481
Streptococcus salivarius JIM8777 uid162145
Streptococcus thermophilus CNRZ1066 uid58221
Streptococcus thermophilus LMG 18311 uid58219
Streptococcus thermophilus JIM 8232 uid162157
Streptococcus thermophilus LMD 9 uid58327
Streptococcus thermophilus MN ZLW 002 uid166827
Streptococcus thermophilus ND03 uid162015
Streptococcus suis D12 uid162127
Streptococcus suis ST1 uid167482
Streptococcus suis D9 uid162125
Streptococcus suis ST3 uid66327
Streptococcus suis JS14 uid162095
Streptococcus suis GZ1 uid161937
Streptococcus suis P1 7 uid32235
Streptococcus suis BM407 uid59321
Streptococcus suis A7 uid162111
Streptococcus suis 98HAH33 uid58665
Streptococcus suis 05ZYH33 uid58663
Streptococcus suis SC84 uid59323
Streptococcus suis SS12 uid162123
Streptococcus mitis B6 uid46097
Streptococcus parasanguinis ATCC 15912 uid49313
Streptococcus parasanguinis FW213 uid163997
Streptococcus gordonii Challis substr CH1 uid57667
Streptococcus oralis Uo5 uid65449
Streptococcus pseudopneumoniae IS7493 uid71153
Streptococcus pneumoniae INV200 uid162035
Streptococcus pneumoniae 70585 uid59125
Streptococcus pneumoniae CGSP14 uid59181
Streptococcus pneumoniae Hungary19A 6 uid59117
Streptococcus pneumoniae OXC141 uid162037
Streptococcus pneumoniae 670 6B uid52533
Streptococcus pneumoniae ATCC 700669 uid59287
Streptococcus pneumoniae G54 uid59167
Streptococcus pneumoniae P1031 uid59123
Streptococcus pneumoniae ST556 uid162191
Streptococcus pneumoniae TCH8431 19A uid49735
Streptococcus pneumoniae TIGR4 uid57857
Streptococcus pneumoniae Taiwan19F 14 uid59119
Streptococcus pneumoniae AP200 uid52453
Streptococcus pneumoniae D39 uid58581
Streptococcus pneumoniae INV104 uid162039
Streptococcus pneumoniae JJA uid59121
Streptococcus pneumoniae R6 uid57859
rplB

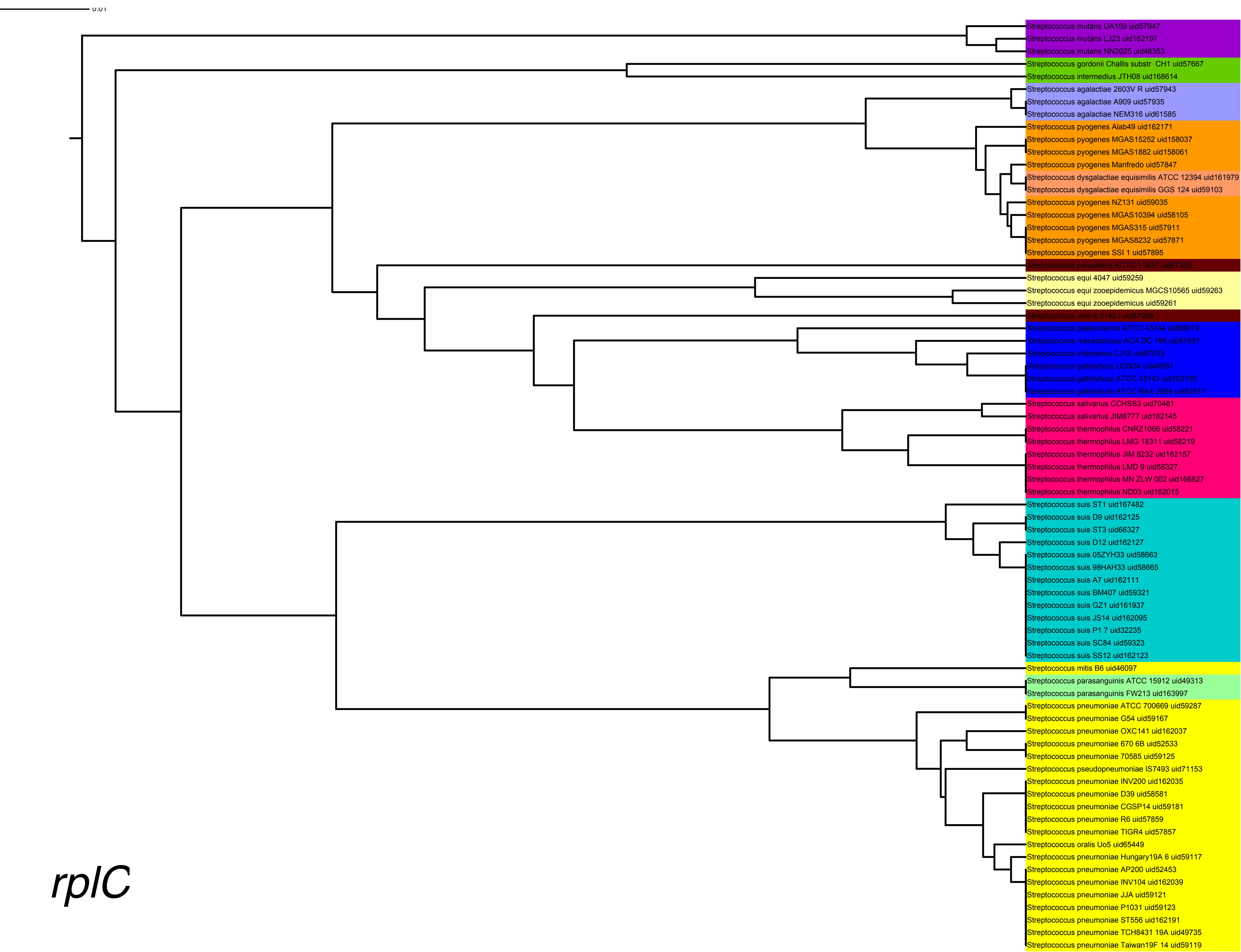
0.01
Streptococcus mutans UA159 uid57947
Streptococcus mutans LJ23 uid162197
Streptococcus mutans NN2025 uid46353
Streptococcus gordonii Challis substr CH1 uid57667
Streptococcus intermedius JTH08 uid168614
Streptococcus agalactiae 2603V R uid57943
Streptococcus agalactiae A909 uid57935
Streptococcus agalactiae NEM316 uid61585
Streptococcus pyogenes Alab49 uid162171
Streptococcus pyogenes MGAS15252 uid158037
Streptococcus pyogenes MGAS1882 uid158061
Streptococcus pyogenes Manfredo uid57847
Streptococcus dysgalactiae equisimilis ATCC 12394 uid161979
Streptococcus dysgalactiae equisimilis GGS 124 uid59103
Streptococcus pyogenes NZ131 uid59035
Streptococcus pyogenes MGAS10394 uid58105
Streptococcus pyogenes MGAS315 uid57911
Streptococcus pyogenes MGAS8232 uid57871
Streptococcus pyogenes SSI 1 uid57895
Streptococcus parauberis KCTC 11537 uid67355
Streptococcus equi 4047 uid59259
Streptococcus equi zooepidemicus MGCS10565 uid59263
Streptococcus equi zooepidemicus uid59261
Streptococcus uberis 0140J uid57959
Streptococcus pasteurianus ATCC 43144 uid68019
Streptococcus macedonicus ACA DC 198 uid81631
Streptococcus infantarius CJ18 uid87033
Streptococcus gallolyticus UCN34 uid46061
Streptococcus gallolyticus ATCC 43143 uid162103
Streptococcus gallolyticus ATCC BAA 2069 uid63617
Streptococcus salivarius CCHSS3 uid70481
Streptococcus salivarius JIM8777 uid162145
Streptococcus thermophilus CNRZ1066 uid58221
Streptococcus thermophilus LMG 18311 uid58219
Streptococcus thermophilus JIM 8232 uid162157
Streptococcus thermophilus LMD 9 uid58327
Streptococcus thermophilus MN ZLW 002 uid166827
Streptococcus thermophilus ND03 uid162015
Streptococcus suis ST1 uid167482
Streptococcus suis D9 uid162125
Streptococcus suis ST3 uid66327
Streptococcus suis D12 uid162127
Streptococcus suis 05ZYH33 uid58663
Streptococcus suis 98HAH33 uid58665
Streptococcus suis A7 uid162111
Streptococcus suis BM407 uid59321
Streptococcus suis GZ1 uid161937
Streptococcus suis JS14 uid162095
Streptococcus suis P1 7 uid32235
Streptococcus suis SC84 uid59323
Streptococcus suis SS12 uid162123
Streptococcus mitis B6 uid46097
Streptococcus parasanguinis ATCC 15912 uid49313
Streptococcus parasanguinis FW213 uid163997
Streptococcus pneumoniae ATCC 700669 uid59287
Streptococcus pneumoniae G54 uid59167
Streptococcus pneumoniae OXC141 uid162037
Streptococcus pneumoniae 670 6B uid52533
Streptococcus pneumoniae 70585 uid59125
Streptococcus pseudopneumoniae IS7493 uid71153
Streptococcus pneumoniae INV200 uid162035
Streptococcus pneumoniae D39 uid58581
Streptococcus pneumoniae CGSP14 uid59181
Streptococcus pneumoniae R6 uid57859
Streptococcus pneumoniae TIGR4 uid57857
Streptococcus oralis Uo5 uid65449
Streptococcus pneumoniae Hungary19A 6 uid59117
Streptococcus pneumoniae AP200 uid52453
Streptococcus pneumoniae INV104 uid162039
Streptococcus pneumoniae JJA uid59121
Streptococcus pneumoniae P1031 uid59123
Streptococcus pneumoniae ST556 uid162191
Streptococcus pneumoniae TCH8431 19A uid49735
Streptococcus pneumoniae Taiwan19F 14 uid59119

rplC

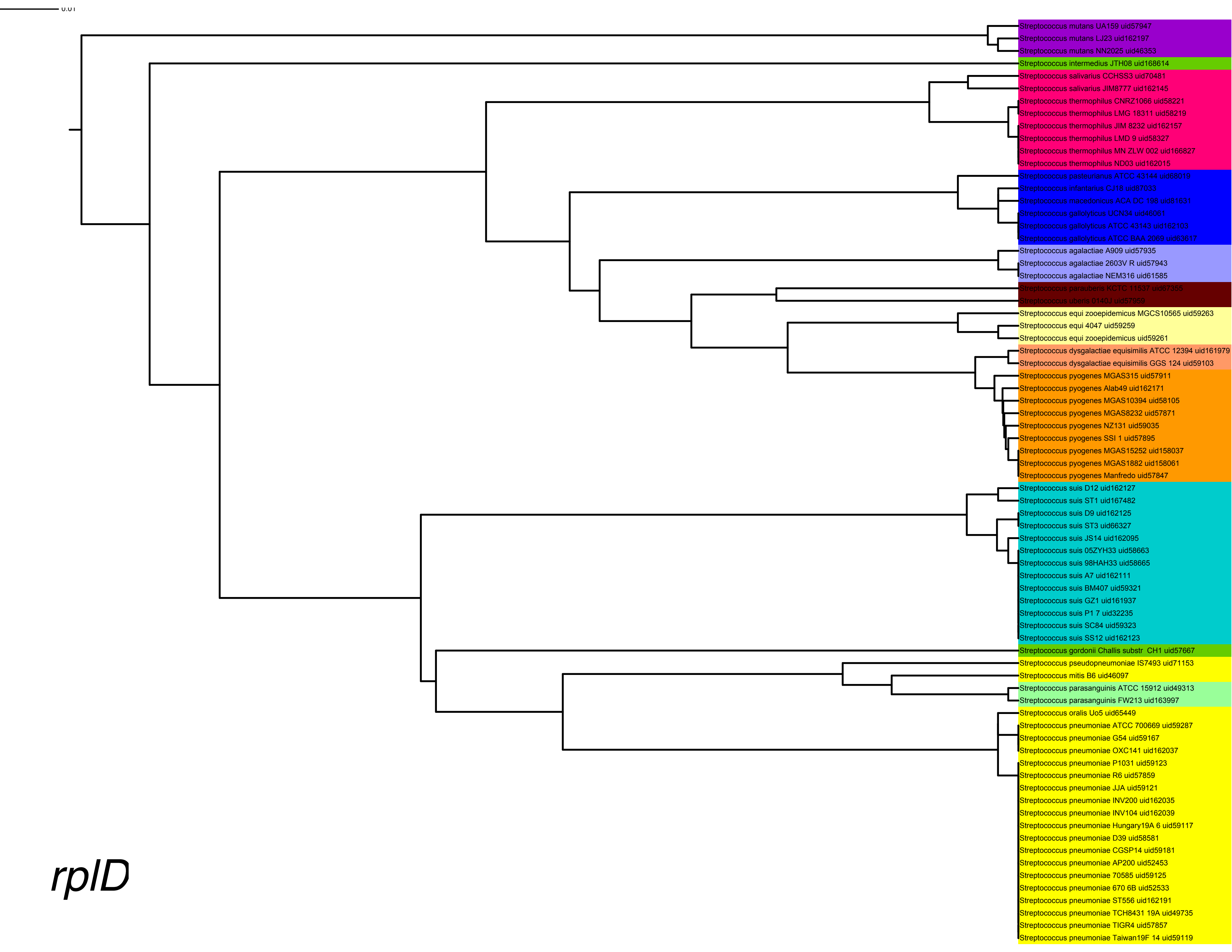
0.01
Streptococcus mutans UA159 uid57947
Streptococcus mutans LJ23 uid162197
Streptococcus mutans NN2025 uid46353
Streptococcus intermedius JTH08 uid168614
Streptococcus salivarius CCHSS3 uid70481
Streptococcus salivarius JIM8777 uid162145
Streptococcus thermophilus CNRZ1066 uid58221
Streptococcus thermophilus LMG 18311 uid58219
Streptococcus thermophilus JIM 8232 uid162157
Streptococcus thermophilus LMD 9 uid58327
Streptococcus thermophilus MN ZLW 002 uid166827
Streptococcus thermophilus ND03 uid162015
Streptococcus pasteurianus ATCC 43144 uid68019
Streptococcus infantarius CJ18 uid87033
Streptococcus macedonicus ACA DC 198 uid81631
Streptococcus gallolyticus UCN34 uid46061
Streptococcus gallolyticus ATCC 43143 uid162103
Streptococcus gallolyticus ATCC BAA 2069 uid63617
Streptococcus agalactiae A909 uid57935
Streptococcus agalactiae 2603V R uid57943
Streptococcus agalactiae NEM316 uid61585
Streptococcus parauberis KCTC 11537 uid67355
Streptococcus uberis 0140J uid57959
Streptococcus equi zooepidemicus MGCS10565 uid59263
Streptococcus equi 4047 uid59259
Streptococcus equi zooepidemicus uid59261
Streptococcus dysgalactiae equisimilis ATCC 12394 uid161979
Streptococcus dysgalactiae equisimilis GGS 124 uid59103
Streptococcus pyogenes MGAS315 uid57911
Streptococcus pyogenes Alab49 uid162171
Streptococcus pyogenes MGAS10394 uid58105
Streptococcus pyogenes MGAS8232 uid57871
Streptococcus pyogenes NZ131 uid59035
Streptococcus pyogenes SSI 1 uid57895
Streptococcus pyogenes MGAS15252 uid158037
Streptococcus pyogenes MGAS1882 uid158061
Streptococcus pyogenes Manfredo uid57847
Streptococcus suis D12 uid162127
Streptococcus suis ST1 uid167482
Streptococcus suis D9 uid162125
Streptococcus suis ST3 uid66327
Streptococcus suis JS14 uid162095
Streptococcus suis 05ZYH33 uid58663
Streptococcus suis 98HAH33 uid58665
Streptococcus suis A7 uid162111
Streptococcus suis BM407 uid59321
Streptococcus suis GZ1 uid161937
Streptococcus suis P1 7 uid32235
Streptococcus suis SC84 uid59323
Streptococcus suis SS12 uid162123
Streptococcus gordonii Challis substr CH1 uid57667
Streptococcus pseudopneumoniae IS7493 uid71153
Streptococcus mitis B6 uid46097
Streptococcus parasanguinis ATCC 15912 uid49313
Streptococcus parasanguinis FW213 uid163997
Streptococcus oralis Uo5 uid65449
Streptococcus pneumoniae ATCC 700669 uid59287
Streptococcus pneumoniae G54 uid59167
Streptococcus pneumoniae OXC141 uid162037
Streptococcus pneumoniae P1031 uid59123
Streptococcus pneumoniae R6 uid57859
Streptococcus pneumoniae JJA uid59121
Streptococcus pneumoniae INV200 uid162035
Streptococcus pneumoniae INV104 uid162039
Streptococcus pneumoniae Hungary19A 6 uid59117
Streptococcus pneumoniae D39 uid58581
Streptococcus pneumoniae CGSP14 uid59181
Streptococcus pneumoniae AP200 uid52453
Streptococcus pneumoniae 70585 uid59125
Streptococcus pneumoniae 670 6B uid52533
Streptococcus pneumoniae ST556 uid162191
Streptococcus pneumoniae TCH8431 19A uid49735
Streptococcus pneumoniae TIGR4 uid57857
Streptococcus pneumoniae Taiwan19F 14 uid59119
rplD

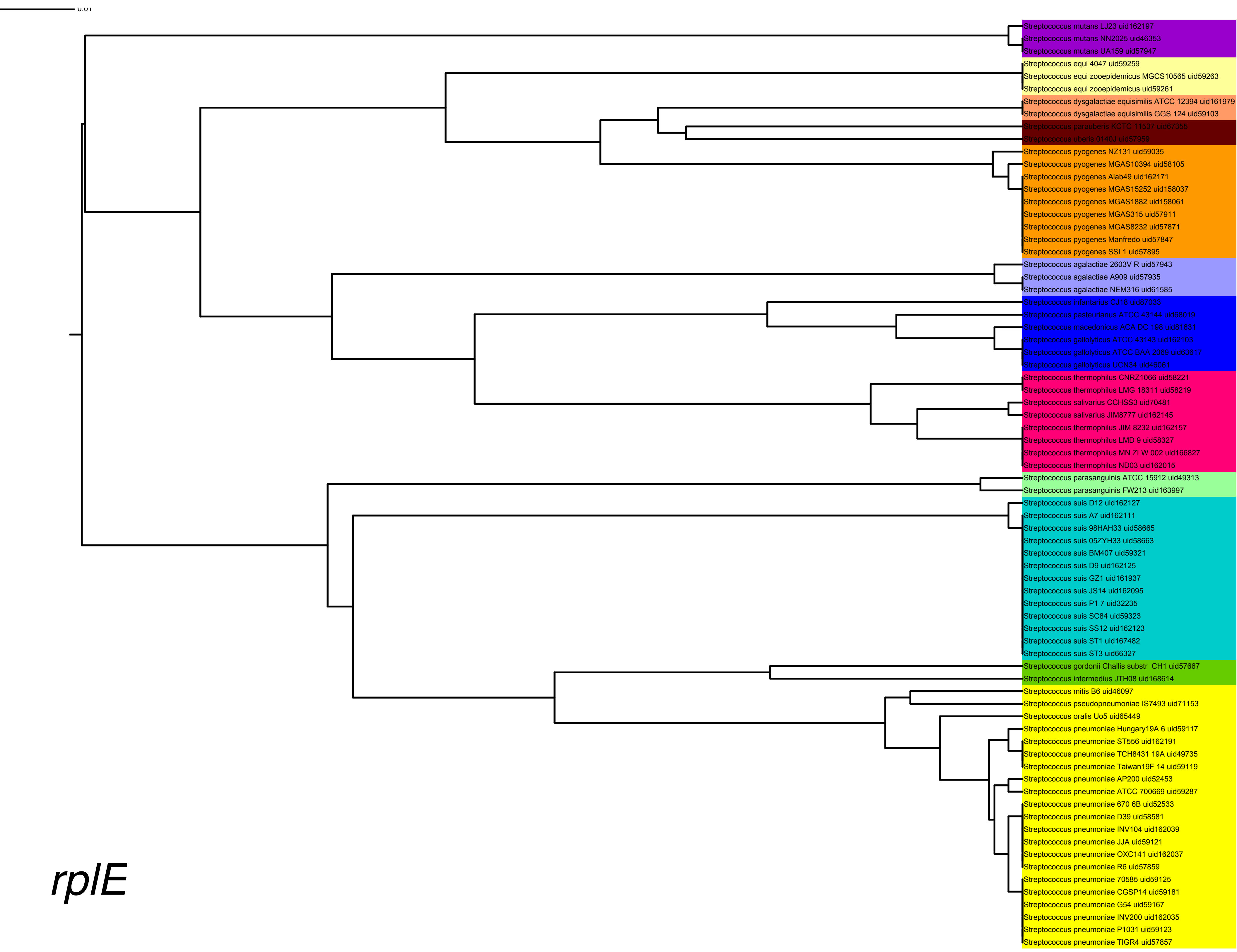
0.01
Streptococcus mutans LJ23 uid162197
Streptococcus mutans NN2025 uid46353
Streptococcus mutans UA159 uid57947
Streptococcus equi 4047 uid59259
Streptococcus equi zooepidemicus MGCS10565 uid59263
Streptococcus equi zooepidemicus uid59261
Streptococcus dysgalactiae equisimilis ATCC 12394 uid161979
Streptococcus dysgalactiae equisimilis GGS 124 uid59103
Streptococcus parauberis KCTC 11537 uid67355
Streptococcus uberis 0140J uid57959
Streptococcus pyogenes NZ131 uid59035
Streptococcus pyogenes MGAS10394 uid58105
Streptococcus pyogenes Alab49 uid162171
Streptococcus pyogenes MGAS15252 uid158037
Streptococcus pyogenes MGAS1882 uid158061
Streptococcus pyogenes MGAS315 uid57911
Streptococcus pyogenes MGAS8232 uid57871
Streptococcus pyogenes Manfredo uid57847
Streptococcus pyogenes SSI 1 uid57895
Streptococcus agalactiae 2603V R uid57943
Streptococcus agalactiae A909 uid57935
Streptococcus agalactiae NEM316 uid61585
Streptococcus infantarius CJ18 uid87033
Streptococcus pasteurianus ATCC 43144 uid68019
Streptococcus macedonicus ACA DC 198 uid81631
Streptococcus gallolyticus ATCC 43143 uid162103
Streptococcus gallolyticus ATCC BAA 2069 uid63617
Streptococcus gallolyticus UCN34 uid46061
Streptococcus thermophilus CNRZ1066 uid58221
Streptococcus thermophilus LMG 18311 uid58219
Streptococcus salivarius CCHSS3 uid70481
Streptococcus salivarius JIM8777 uid162145
Streptococcus thermophilus JIM 8232 uid162157
Streptococcus thermophilus LMD 9 uid58327
Streptococcus thermophilus MN ZLW 002 uid166827
Streptococcus thermophilus ND03 uid162015
Streptococcus parasanguinis ATCC 15912 uid49313
Streptococcus parasanguinis FW213 uid163997
Streptococcus suis D12 uid162127
Streptococcus suis A7 uid162111
Streptococcus suis 98HAH33 uid58665
Streptococcus suis 05ZYH33 uid58663
Streptococcus suis BM407 uid59321
Streptococcus suis D9 uid162125
Streptococcus suis GZ1 uid161937
Streptococcus suis JS14 uid162095
Streptococcus suis P1 7 uid32235
Streptococcus suis SC84 uid59323
Streptococcus suis SS12 uid162123
Streptococcus suis ST1 uid167482
Streptococcus suis ST3 uid66327
Streptococcus gordonii Challis substr CH1 uid57667
Streptococcus intermedius JTH08 uid168614
Streptococcus mitis B6 uid46097
Streptococcus pseudopneumoniae IS7493 uid71153
Streptococcus oralis Uo5 uid65449
Streptococcus pneumoniae Hungary19A 6 uid59117
Streptococcus pneumoniae ST556 uid162191
Streptococcus pneumoniae TCH8431 19A uid49735
Streptococcus pneumoniae Taiwan19F 14 uid59119
Streptococcus pneumoniae AP200 uid52453
Streptococcus pneumoniae ATCC 700669 uid59287
Streptococcus pneumoniae 670 6B uid52533
Streptococcus pneumoniae D39 uid58581
Streptococcus pneumoniae INV104 uid162039
Streptococcus pneumoniae JJA uid59121
Streptococcus pneumoniae OXC141 uid162037
Streptococcus pneumoniae R6 uid57859
Streptococcus pneumoniae 70585 uid59125
Streptococcus pneumoniae CGSP14 uid59181
Streptococcus pneumoniae G54 uid59167
Streptococcus pneumoniae INV200 uid162035
Streptococcus pneumoniae P1031 uid59123
Streptococcus pneumoniae TIGR4 uid57857
rplE

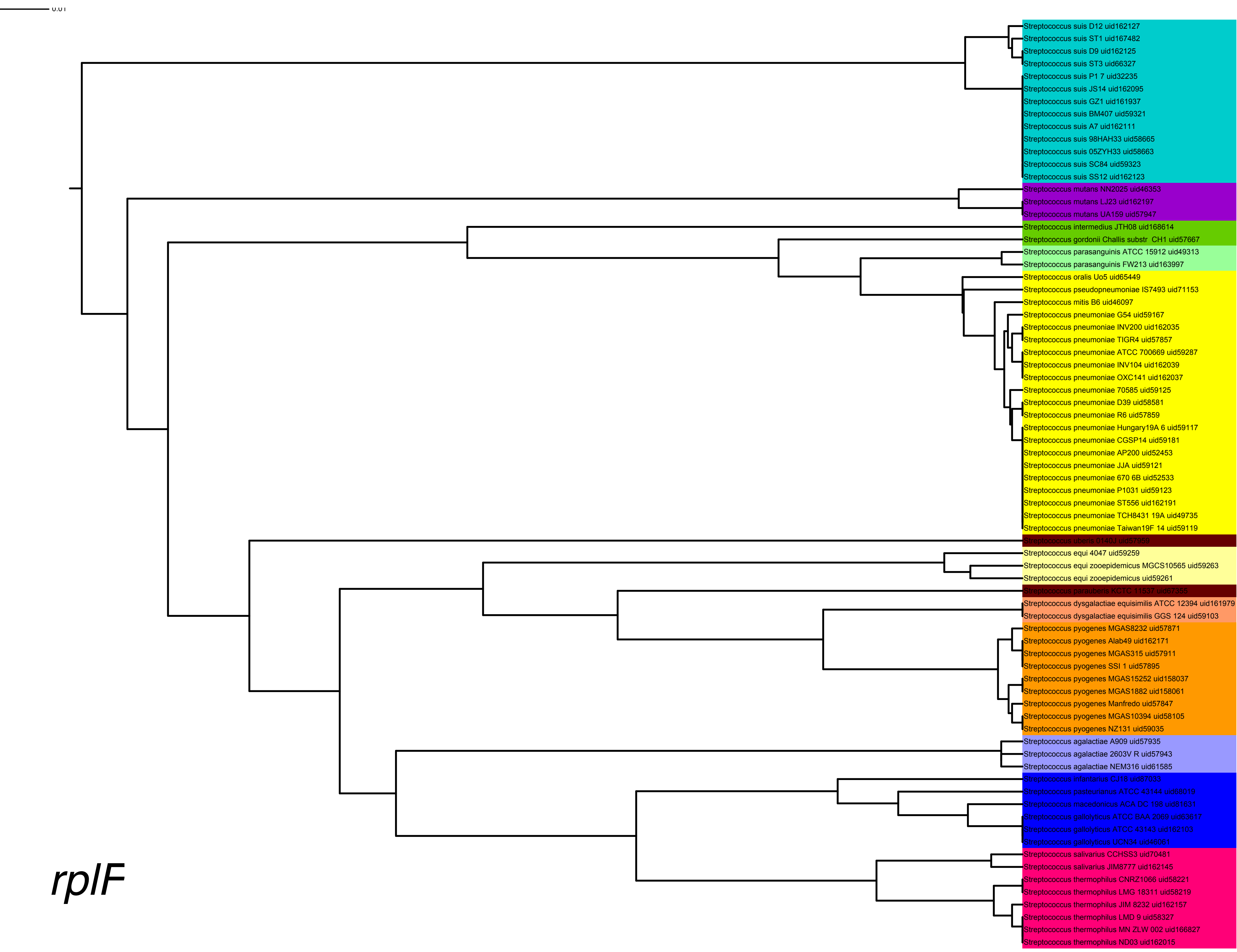
0.01
Streptococcus suis D12 uid162127
Streptococcus suis ST1 uid167482
Streptococcus suis D9 uid162125
Streptococcus suis ST3 uid66327
Streptococcus suis P1 7 uid32235
Streptococcus suis JS14 uid162095
Streptococcus suis GZ1 uid161937
Streptococcus suis BM407 uid59321
Streptococcus suis A7 uid162111
Streptococcus suis 98HAH33 uid58665
Streptococcus suis 05ZYH33 uid58663
Streptococcus suis SC84 uid59323
Streptococcus suis SS12 uid162123
Streptococcus mutans NN2025 uid46353
Streptococcus mutans LJ23 uid162197
Streptococcus mutans UA159 uid57947
Streptococcus intermedius JTH08 uid168614
Streptococcus gordonii Challis substr CH1 uid57667
Streptococcus parasanguinis ATCC 15912 uid49313
Streptococcus parasanguinis FW213 uid163997
Streptococcus oralis Uo5 uid65449
Streptococcus pseudopneumoniae IS7493 uid71153
Streptococcus mitis B6 uid46097
Streptococcus pneumoniae G54 uid59167
Streptococcus pneumoniae INV200 uid162035
Streptococcus pneumoniae TIGR4 uid57857
Streptococcus pneumoniae ATCC 700669 uid59287
Streptococcus pneumoniae INV104 uid162039
Streptococcus pneumoniae OXC141 uid162037
Streptococcus pneumoniae 70585 uid59125
Streptococcus pneumoniae D39 uid58581
Streptococcus pneumoniae R6 uid57859
Streptococcus pneumoniae Hungary19A 6 uid59117
Streptococcus pneumoniae CGSP14 uid59181
Streptococcus pneumoniae AP200 uid52453
Streptococcus pneumoniae JJA uid59121
Streptococcus pneumoniae 670 6B uid52533
Streptococcus pneumoniae P1031 uid59123
Streptococcus pneumoniae ST556 uid162191
Streptococcus pneumoniae TCH8431 19A uid49735
Streptococcus pneumoniae Taiwan19F 14 uid59119
Streptococcus uberis 0140J uid57959
Streptococcus equi 4047 uid59259
Streptococcus equi zooepidemicus MGCS10565 uid59263
Streptococcus equi zooepidemicus uid59261
Streptococcus parauberis KCTC 11537 uid67355
Streptococcus dysgalactiae equisimilis ATCC 12394 uid161979
Streptococcus dysgalactiae equisimilis GGS 124 uid59103
Streptococcus pyogenes MGAS8232 uid57871
Streptococcus pyogenes Alab49 uid162171
Streptococcus pyogenes MGAS315 uid57911
Streptococcus pyogenes SSI 1 uid57895
Streptococcus pyogenes MGAS15252 uid158037
Streptococcus pyogenes MGAS1882 uid158061
Streptococcus pyogenes Manfredo uid57847
Streptococcus pyogenes MGAS10394 uid58105
Streptococcus pyogenes NZ131 uid59035
Streptococcus agalactiae A909 uid57935
Streptococcus agalactiae 2603V R uid57943
Streptococcus agalactiae NEM316 uid61585
Streptococcus infantarius CJ18 uid87033
Streptococcus pasteurianus ATCC 43144 uid68019
Streptococcus macedonicus ACA DC 198 uid81631
Streptococcus gallolyticus ATCC BAA 2069 uid63617
Streptococcus gallolyticus ATCC 43143 uid162103
Streptococcus gallolyticus UCN34 uid46061
Streptococcus salivarius CCHSS3 uid70481
Streptococcus salivarius JIM8777 uid162145
Streptococcus thermophilus CNRZ1066 uid58221
Streptococcus thermophilus LMG 18311 uid58219
Streptococcus thermophilus JIM 8232 uid162157
Streptococcus thermophilus LMD 9 uid58327
Streptococcus thermophilus MN ZLW 002 uid166827
Streptococcus thermophilus ND03 uid162015
rplF

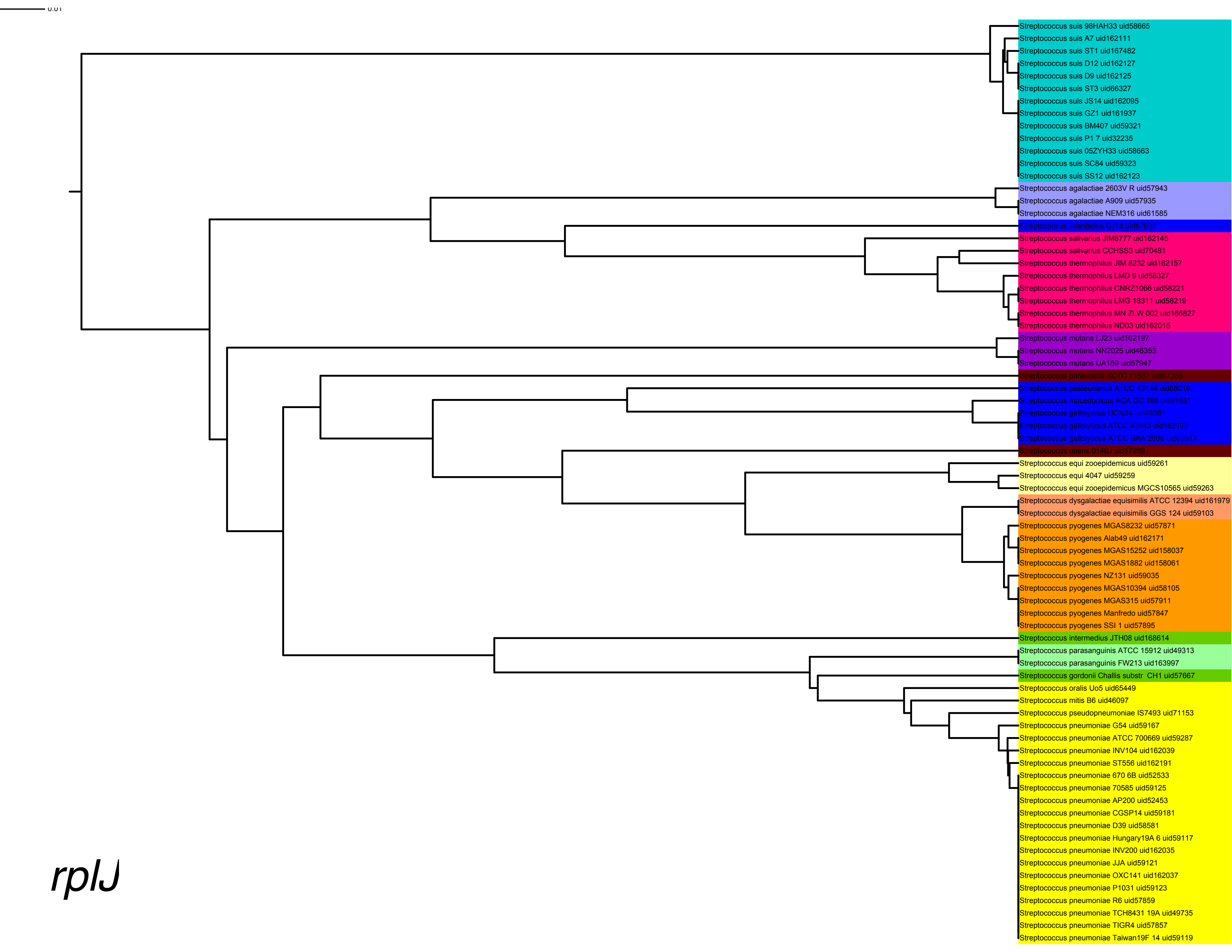
0.01
Streptococcus suis 98HAH33 uid58665
Streptococcus suis A7 uid162111
Streptococcus suis ST1 uid167482
Streptococcus suis D12 uid162127
Streptococcus suis D9 uid162125
Streptococcus suis ST3 uid66327
Streptococcus suis JS14 uid162095
Streptococcus suis GZ1 uid161937
Streptococcus suis BM407 uid59321
Streptococcus suis P1 7 uid32235
Streptococcus suis 05ZYH33 uid58663
Streptococcus suis SC84 uid59323
Streptococcus suis SS12 uid162123
Streptococcus agalactiae 2603V R uid57943
Streptococcus agalactiae A909 uid57935
Streptococcus agalactiae NEM316 uid61585
Streptococcus infantarius CJ18 uid87033
Streptococcus salivarius JIM8777 uid162145
Streptococcus salivarius CCHSS3 uid70481
Streptococcus thermophilus JIM 8232 uid162157
Streptococcus thermophilus LMD 9 uid58327
Streptococcus thermophilus CNRZ1066 uid58221
Streptococcus thermophilus LMG 18311 uid58219
Streptococcus thermophilus MN ZLW 002 uid166827
Streptococcus thermophilus ND03 uid162015
Streptococcus mutans LJ23 uid162197
Streptococcus mutans NN2025 uid46353
Streptococcus mutans UA159 uid57947
Streptococcus parauberis KCTC 11537 uid67355
Streptococcus pasteurianus ATCC 43144 uid68019
Streptococcus macedonicus ACA DC 198 uid81631
Streptococcus gallolyticus UCN34 uid46061
Streptococcus gallolyticus ATCC 43143 uid162103
Streptococcus gallolyticus ATCC BAA 2069 uid63617
Streptococcus uberis 0140J uid57959
Streptococcus equi zooepidemicus uid59261
Streptococcus equi 4047 uid59259
Streptococcus equi zooepidemicus MGCS10565 uid59263
Streptococcus dysgalactiae equisimilis ATCC 12394 uid161979
Streptococcus dysgalactiae equisimilis GGS 124 uid59103
Streptococcus pyogenes MGAS8232 uid57871
Streptococcus pyogenes Alab49 uid162171
Streptococcus pyogenes MGAS15252 uid158037
Streptococcus pyogenes MGAS1882 uid158061
Streptococcus pyogenes NZ131 uid59035
Streptococcus pyogenes MGAS10394 uid58105
Streptococcus pyogenes MGAS315 uid57911
Streptococcus pyogenes Manfredo uid57847
Streptococcus pyogenes SSI 1 uid57895
Streptococcus intermedius JTH08 uid168614
Streptococcus parasanguinis ATCC 15912 uid49313
Streptococcus parasanguinis FW213 uid163997
Streptococcus gordonii Challis substr CH1 uid57667
Streptococcus oralis Uo5 uid65449
Streptococcus mitis B6 uid46097
Streptococcus pseudopneumoniae IS7493 uid71153
Streptococcus pneumoniae G54 uid59167
Streptococcus pneumoniae ATCC 700669 uid59287
Streptococcus pneumoniae INV104 uid162039
Streptococcus pneumoniae ST556 uid162191
Streptococcus pneumoniae 670 6B uid52533
Streptococcus pneumoniae 70585 uid59125
Streptococcus pneumoniae AP200 uid52453
Streptococcus pneumoniae CGSP14 uid59181
Streptococcus pneumoniae D39 uid58581
Streptococcus pneumoniae Hungary19A 6 uid59117
Streptococcus pneumoniae INV200 uid162035
Streptococcus pneumoniae JJA uid59121
Streptococcus pneumoniae OXC141 uid162037
Streptococcus pneumoniae P1031 uid59123
Streptococcus pneumoniae R6 uid57859
Streptococcus pneumoniae TCH8431 19A uid49735
Streptococcus pneumoniae TIGR4 uid57857
Streptococcus pneumoniae Taiwan19F 14 uid59119
rplJ

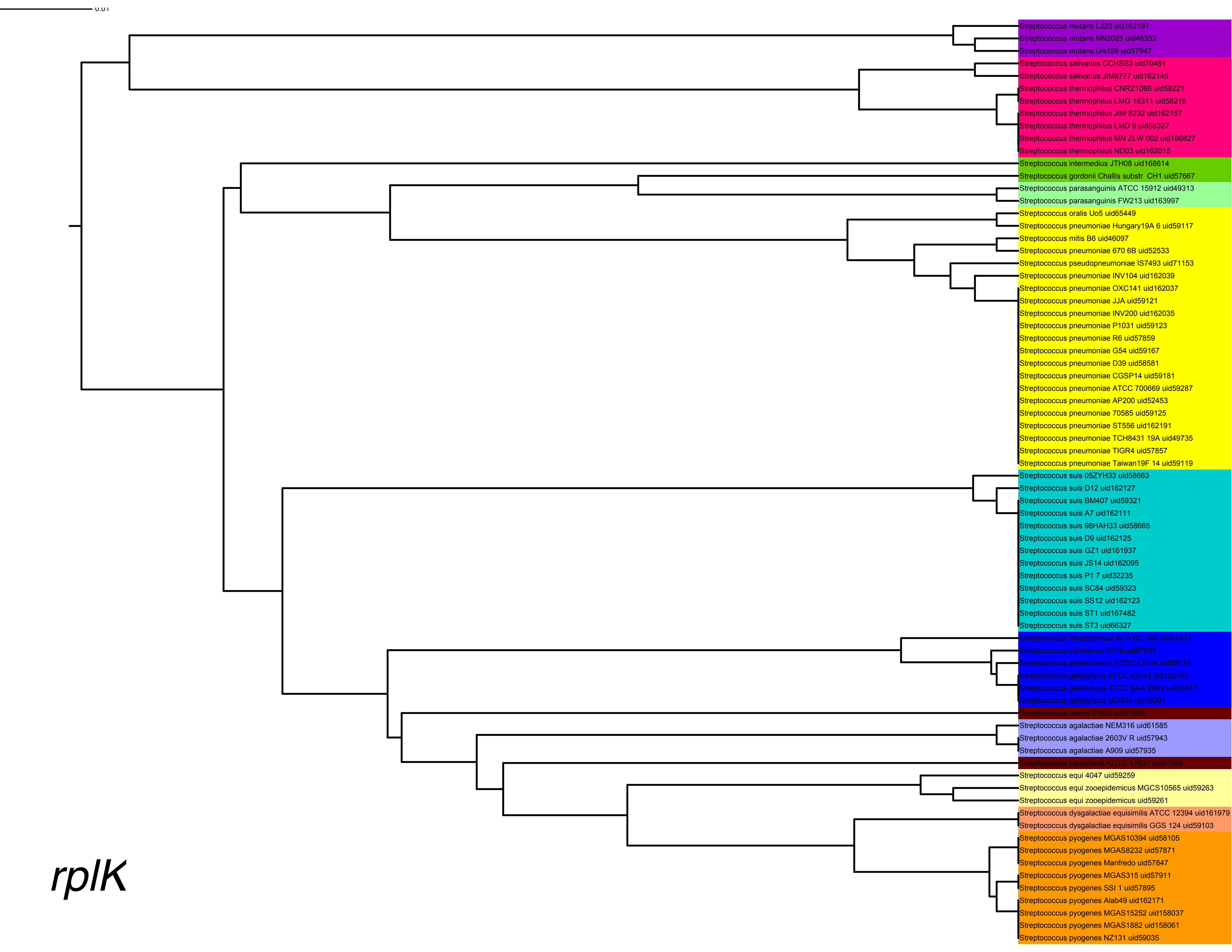
0.01
Streptococcus mutans LJ23 uid162197
Streptococcus mutans NN2025 uid46353
Streptococcus mutans UA159 uid57947
Streptococcus salivarius CCHSS3 uid70481
Streptococcus salivarius JIM8777 uid162145
Streptococcus thermophilus CNRZ1066 uid58221
Streptococcus thermophilus LMG 18311 uid58219
Streptococcus thermophilus JIM 8232 uid162157
Streptococcus thermophilus LMD 9 uid58327
Streptococcus thermophilus MN ZLW 002 uid166827
Streptococcus thermophilus ND03 uid162015
Streptococcus intermedius JTH08 uid168614
Streptococcus gordonii Challis substr CH1 uid57667
Streptococcus parasanguinis ATCC 15912 uid49313
Streptococcus parasanguinis FW213 uid163997
Streptococcus oralis Uo5 uid65449
Streptococcus pneumoniae Hungary19A 6 uid59117
Streptococcus mitis B6 uid46097
Streptococcus pneumoniae 670 6B uid52533
Streptococcus pseudopneumoniae IS7493 uid71153
Streptococcus pneumoniae INV104 uid162039
Streptococcus pneumoniae OXC141 uid162037
Streptococcus pneumoniae JJA uid59121
Streptococcus pneumoniae INV200 uid162035
Streptococcus pneumoniae P1031 uid59123
Streptococcus pneumoniae R6 uid57859
Streptococcus pneumoniae G54 uid59167
Streptococcus pneumoniae D39 uid58581
Streptococcus pneumoniae CGSP14 uid59181
Streptococcus pneumoniae ATCC 700669 uid59287
Streptococcus pneumoniae AP200 uid52453
Streptococcus pneumoniae 70585 uid59125
Streptococcus pneumoniae ST556 uid162191
Streptococcus pneumoniae TCH8431 19A uid49735
Streptococcus pneumoniae TIGR4 uid57857
Streptococcus pneumoniae Taiwan19F 14 uid59119
Streptococcus suis 05ZYH33 uid58663
Streptococcus suis D12 uid162127
Streptococcus suis BM407 uid59321
Streptococcus suis A7 uid162111
Streptococcus suis 98HAH33 uid58665
Streptococcus suis D9 uid162125
Streptococcus suis GZ1 uid161937
Streptococcus suis JS14 uid162095
Streptococcus suis P1 7 uid32235
Streptococcus suis SC84 uid59323
Streptococcus suis SS12 uid162123
Streptococcus suis ST1 uid167482
Streptococcus suis ST3 uid66327
Streptococcus macedonicus ACA DC 198 uid81631
Streptococcus infantarius CJ18 uid87033
Streptococcus pasteurianus ATCC 43144 uid68019
Streptococcus gallolyticus ATCC 43143 uid162103
Streptococcus gallolyticus ATCC BAA 2069 uid63617
Streptococcus gallolyticus UCN34 uid46061
Streptococcus uberis 0140J uid57959
Streptococcus agalactiae NEM316 uid61585
Streptococcus agalactiae 2603V R uid57943
Streptococcus agalactiae A909 uid57935
Streptococcus parauberis KCTC 11537 uid67355
Streptococcus equi 4047 uid59259
Streptococcus equi zooepidemicus MGCS10565 uid59263
Streptococcus equi zooepidemicus uid59261
Streptococcus dysgalactiae equisimilis ATCC 12394 uid161979
Streptococcus dysgalactiae equisimilis GGS 124 uid59103
Streptococcus pyogenes MGAS10394 uid58105
Streptococcus pyogenes MGAS8232 uid57871
Streptococcus pyogenes Manfredo uid57847
Streptococcus pyogenes MGAS315 uid57911
Streptococcus pyogenes SSI 1 uid57895
Streptococcus pyogenes Alab49 uid162171
Streptococcus pyogenes MGAS15252 uid158037
Streptococcus pyogenes MGAS1882 uid158061
Streptococcus pyogenes NZ131 uid59035
rplK

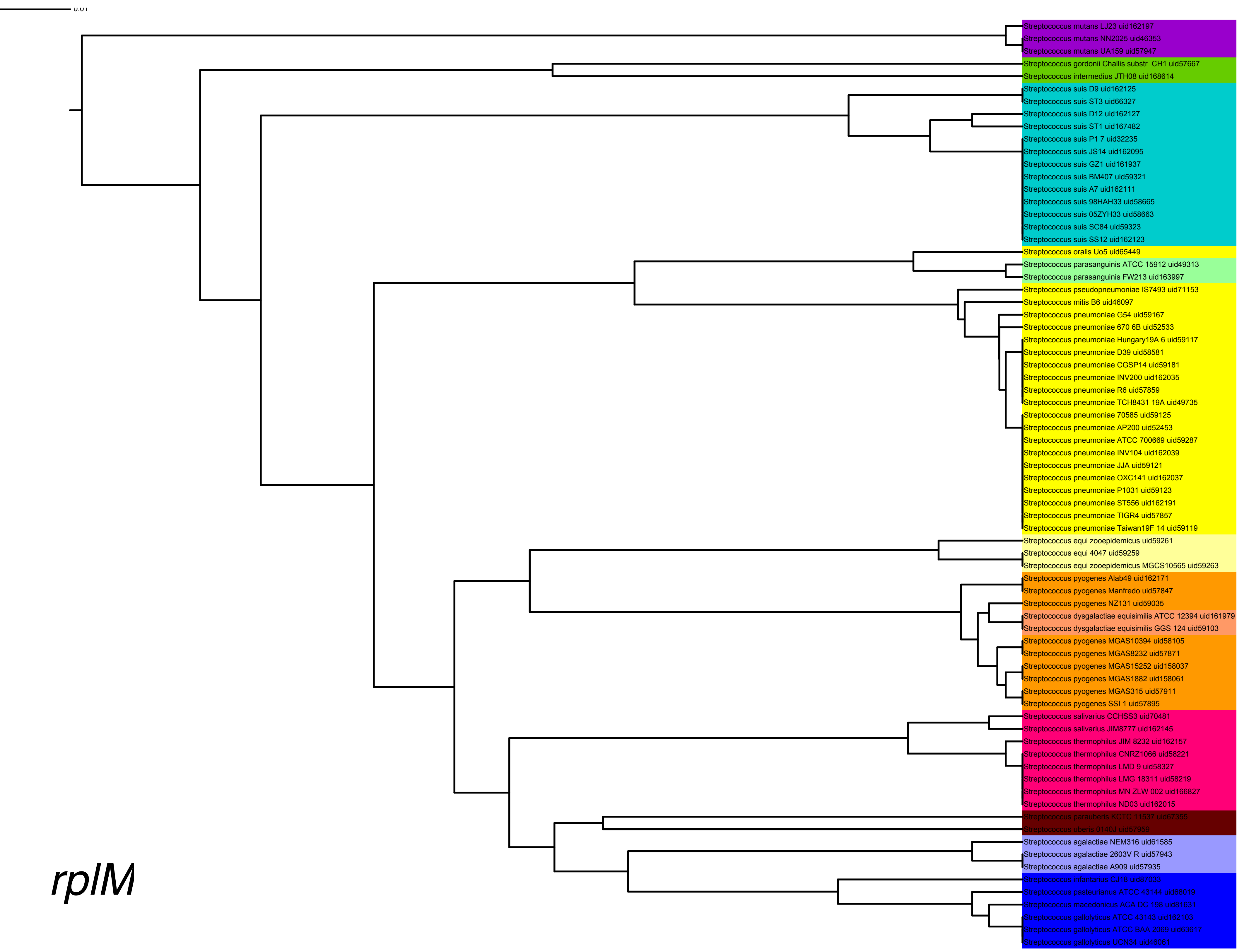
0.01
Streptococcus mutans LJ23 uid162197
Streptococcus mutans NN2025 uid46353
Streptococcus mutans UA159 uid57947
Streptococcus gordonii Challis substr CH1 uid57667
Streptococcus intermedius JTH08 uid168614
Streptococcus suis D9 uid162125
Streptococcus suis ST3 uid66327
Streptococcus suis D12 uid162127
Streptococcus suis ST1 uid167482
Streptococcus suis P1 7 uid32235
Streptococcus suis JS14 uid162095
Streptococcus suis GZ1 uid161937
Streptococcus suis BM407 uid59321
Streptococcus suis A7 uid162111
Streptococcus suis 98HAH33 uid58665
Streptococcus suis 05ZYH33 uid58663
Streptococcus suis SC84 uid59323
Streptococcus suis SS12 uid162123
Streptococcus oralis Uo5 uid65449
Streptococcus parasanguinis ATCC 15912 uid49313
Streptococcus parasanguinis FW213 uid163997
Streptococcus pseudopneumoniae IS7493 uid71153
Streptococcus mitis B6 uid46097
Streptococcus pneumoniae G54 uid59167
Streptococcus pneumoniae 670 6B uid52533
Streptococcus pneumoniae Hungary19A 6 uid59117
Streptococcus pneumoniae D39 uid58581
Streptococcus pneumoniae CGSP14 uid59181
Streptococcus pneumoniae INV200 uid162035
Streptococcus pneumoniae R6 uid57859
Streptococcus pneumoniae TCH8431 19A uid49735
Streptococcus pneumoniae 70585 uid59125
Streptococcus pneumoniae AP200 uid52453
Streptococcus pneumoniae ATCC 700669 uid59287
Streptococcus pneumoniae INV104 uid162039
Streptococcus pneumoniae JJA uid59121
Streptococcus pneumoniae OXC141 uid162037
Streptococcus pneumoniae P1031 uid59123
Streptococcus pneumoniae ST556 uid162191
Streptococcus pneumoniae TIGR4 uid57857
Streptococcus pneumoniae Taiwan19F 14 uid59119
Streptococcus equi zooepidemicus uid59261
Streptococcus equi 4047 uid59259
Streptococcus equi zooepidemicus MGCS10565 uid59263
Streptococcus pyogenes Alab49 uid162171
Streptococcus pyogenes Manfredo uid57847
Streptococcus pyogenes NZ131 uid59035
Streptococcus dysgalactiae equisimilis ATCC 12394 uid161979
Streptococcus dysgalactiae equisimilis GGS 124 uid59103
Streptococcus pyogenes MGAS10394 uid58105
Streptococcus pyogenes MGAS8232 uid57871
Streptococcus pyogenes MGAS15252 uid158037
Streptococcus pyogenes MGAS1882 uid158061
Streptococcus pyogenes MGAS315 uid57911
Streptococcus pyogenes SSI 1 uid57895
Streptococcus salivarius CCHSS3 uid70481
Streptococcus salivarius JIM8777 uid162145
Streptococcus thermophilus JIM 8232 uid162157
Streptococcus thermophilus CNRZ1066 uid58221
Streptococcus thermophilus LMD 9 uid58327
Streptococcus thermophilus LMG 18311 uid58219
Streptococcus thermophilus MN ZLW 002 uid166827
Streptococcus thermophilus ND03 uid162015
Streptococcus parauberis KCTC 11537 uid67355
Streptococcus uberis 0140J uid57959
Streptococcus agalactiae NEM316 uid61585
Streptococcus agalactiae 2603V R uid57943
Streptococcus agalactiae A909 uid57935
Streptococcus infantarius CJ18 uid87033
Streptococcus pasteurianus ATCC 43144 uid68019
Streptococcus macedonicus ACA DC 198 uid81631
Streptococcus gallolyticus ATCC 43143 uid162103
Streptococcus gallolyticus ATCC BAA 2069 uid63617
Streptococcus gallolyticus UCN34 uid46061
rplM

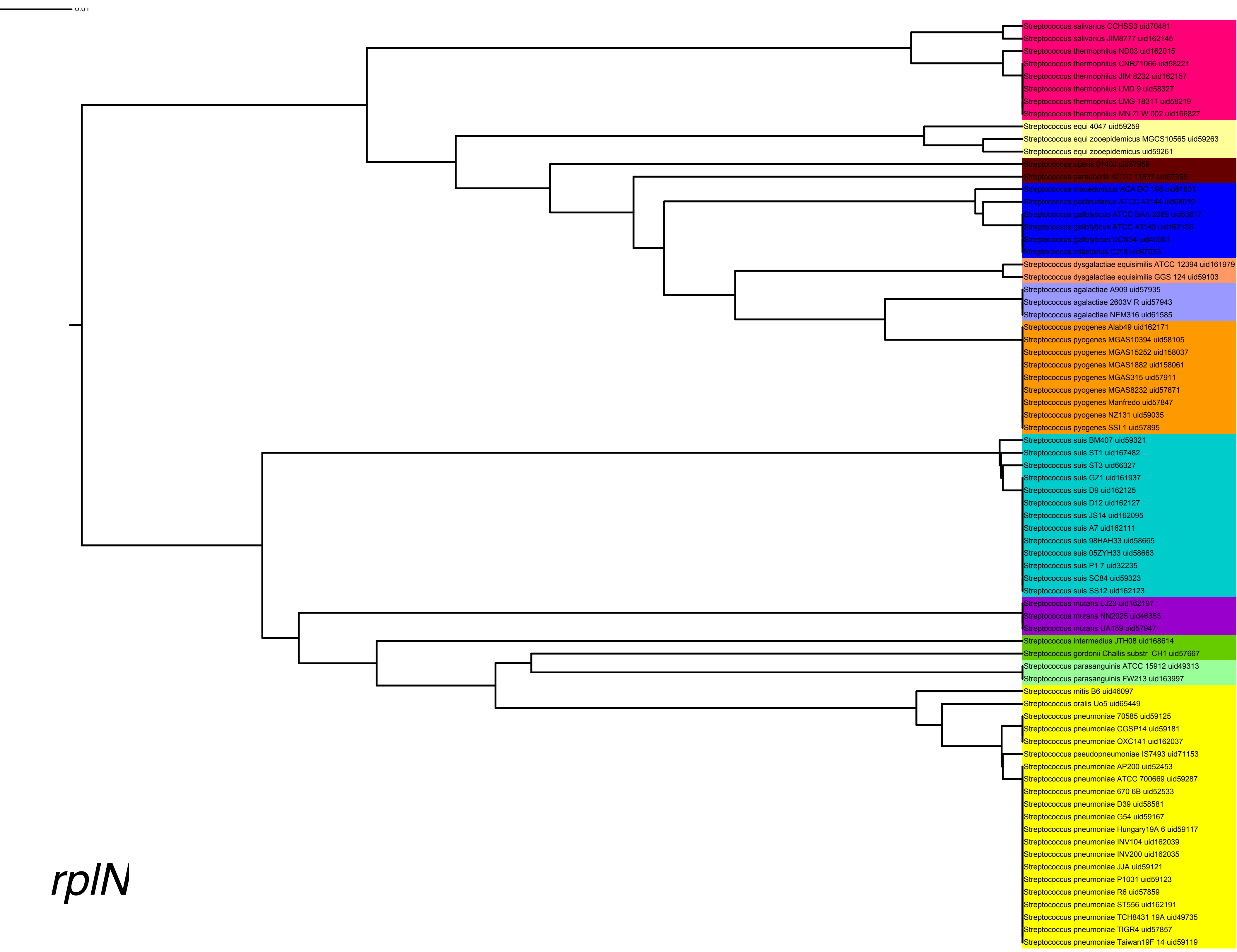
0.01
Streptococcus salivarius CCHSS3 uid70481
Streptococcus salivarius JIM8777 uid162145
Streptococcus thermophilus ND03 uid162015
Streptococcus thermophilus CNRZ1066 uid58221
Streptococcus thermophilus JIM 8232 uid162157
Streptococcus thermophilus LMD 9 uid58327
Streptococcus thermophilus LMG 18311 uid58219
Streptococcus thermophilus MN ZLW 002 uid166827
Streptococcus equi 4047 uid59259
Streptococcus equi zooepidemicus MGCS10565 uid59263
Streptococcus equi zooepidemicus uid59261
Streptococcus uberis 0140J uid57959
Streptococcus parauberis KCTC 11537 uid67355
Streptococcus macedonicus ACA DC 198 uid81631
Streptococcus pasteurianus ATCC 43144 uid68019
Streptococcus gallolyticus ATCC BAA 2069 uid63617
Streptococcus gallolyticus ATCC 43143 uid162103
Streptococcus gallolyticus UCN34 uid46061
Streptococcus infantarius CJ18 uid87033
Streptococcus dysgalactiae equisimilis ATCC 12394 uid161979
Streptococcus dysgalactiae equisimilis GGS 124 uid59103
Streptococcus agalactiae A909 uid57935
Streptococcus agalactiae 2603V R uid57943
Streptococcus agalactiae NEM316 uid61585
Streptococcus pyogenes Alab49 uid162171
Streptococcus pyogenes MGAS10394 uid58105
Streptococcus pyogenes MGAS15252 uid158037
Streptococcus pyogenes MGAS1882 uid158061
Streptococcus pyogenes MGAS315 uid57911
Streptococcus pyogenes MGAS8232 uid57871
Streptococcus pyogenes Manfredo uid57847
Streptococcus pyogenes NZ131 uid59035
Streptococcus pyogenes SSI 1 uid57895
Streptococcus suis BM407 uid59321
Streptococcus suis ST1 uid167482
Streptococcus suis ST3 uid66327
Streptococcus suis GZ1 uid161937
Streptococcus suis D9 uid162125
Streptococcus suis D12 uid162127
Streptococcus suis JS14 uid162095
Streptococcus suis A7 uid162111
Streptococcus suis 98HAH33 uid58665
Streptococcus suis 05ZYH33 uid58663
Streptococcus suis P1 7 uid32235
Streptococcus suis SC84 uid59323
Streptococcus suis SS12 uid162123
Streptococcus mutans LJ23 uid162197
Streptococcus mutans NN2025 uid46353
Streptococcus mutans UA159 uid57947
Streptococcus intermedius JTH08 uid168614
Streptococcus gordonii Challis substr CH1 uid57667
Streptococcus parasanguinis ATCC 15912 uid49313
Streptococcus parasanguinis FW213 uid163997
Streptococcus mitis B6 uid46097
Streptococcus oralis Uo5 uid65449
Streptococcus pneumoniae 70585 uid59125
Streptococcus pneumoniae CGSP14 uid59181
Streptococcus pneumoniae OXC141 uid162037
Streptococcus pseudopneumoniae IS7493 uid71153
Streptococcus pneumoniae AP200 uid52453
Streptococcus pneumoniae ATCC 700669 uid59287
Streptococcus pneumoniae 670 6B uid52533
Streptococcus pneumoniae D39 uid58581
Streptococcus pneumoniae G54 uid59167
Streptococcus pneumoniae Hungary19A 6 uid59117
Streptococcus pneumoniae INV104 uid162039
Streptococcus pneumoniae INV200 uid162035
Streptococcus pneumoniae JJA uid59121
Streptococcus pneumoniae P1031 uid59123
Streptococcus pneumoniae R6 uid57859
Streptococcus pneumoniae ST556 uid162191
Streptococcus pneumoniae TCH8431 19A uid49735
Streptococcus pneumoniae TIGR4 uid57857
Streptococcus pneumoniae Taiwan19F 14 uid59119
rplN

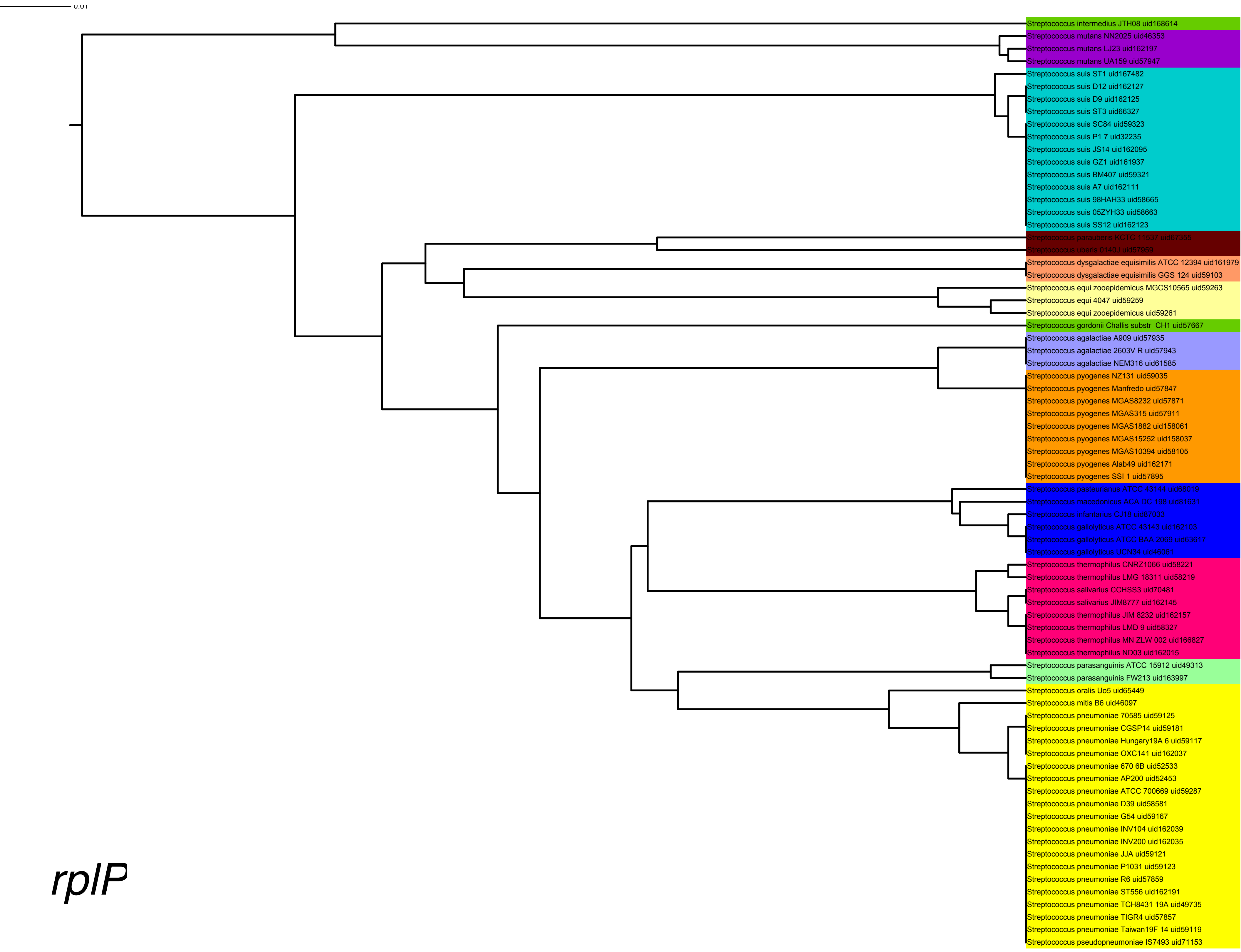

0.01
Streptococcus intermedius JTH08 uid168614
Streptococcus mutans NN2025 uid46353
Streptococcus mutans LJ23 uid162197
Streptococcus mutans UA159 uid57947
Streptococcus suis ST1 uid167482
Streptococcus suis D12 uid162127
Streptococcus suis D9 uid162125
Streptococcus suis ST3 uid66327
Streptococcus suis SC84 uid59323
Streptococcus suis P1 7 uid32235
Streptococcus suis JS14 uid162095
Streptococcus suis GZ1 uid161937
Streptococcus suis BM407 uid59321
Streptococcus suis A7 uid162111
Streptococcus suis 98HAH33 uid58665
Streptococcus suis 05ZYH33 uid58663
Streptococcus suis SS12 uid162123
Streptococcus parauberis KCTC 11537 uid67355
Streptococcus uberis 0140J uid57959
Streptococcus dysgalactiae equisimilis ATCC 12394 uid161979
Streptococcus dysgalactiae equisimilis GGS 124 uid59103
Streptococcus equi zooepidemicus MGCS10565 uid59263
Streptococcus equi 4047 uid59259
Streptococcus equi zooepidemicus uid59261
Streptococcus gordonii Challis substr CH1 uid57667
Streptococcus agalactiae A909 uid57935
Streptococcus agalactiae 2603V R uid57943
Streptococcus agalactiae NEM316 uid61585
Streptococcus pyogenes NZ131 uid59035
Streptococcus pyogenes Manfredo uid57847
Streptococcus pyogenes MGAS8232 uid57871
Streptococcus pyogenes MGAS315 uid57911
Streptococcus pyogenes MGAS1882 uid158061
Streptococcus pyogenes MGAS15252 uid158037
Streptococcus pyogenes MGAS10394 uid58105
Streptococcus pyogenes Alab49 uid162171
Streptococcus pyogenes SSI 1 uid57895
Streptococcus pasteurianus ATCC 43144 uid68019
Streptococcus macedonicus ACA DC 198 uid81631
Streptococcus infantarius CJ18 uid87033
Streptococcus gallolyticus ATCC 43143 uid162103
Streptococcus gallolyticus ATCC BAA 2069 uid63617
Streptococcus gallolyticus UCN34 uid46061
Streptococcus thermophilus CNRZ1066 uid58221
Streptococcus thermophilus LMG 18311 uid58219
Streptococcus salivarius CCHSS3 uid70481
Streptococcus salivarius JIM8777 uid162145
Streptococcus thermophilus JIM 8232 uid162157
Streptococcus thermophilus LMD 9 uid58327
Streptococcus thermophilus MN ZLW 002 uid166827
Streptococcus thermophilus ND03 uid162015
Streptococcus parasanguinis ATCC 15912 uid49313
Streptococcus parasanguinis FW213 uid163997
Streptococcus oralis Uo5 uid65449
Streptococcus mitis B6 uid46097
Streptococcus pneumoniae 70585 uid59125
Streptococcus pneumoniae CGSP14 uid59181
Streptococcus pneumoniae Hungary19A 6 uid59117
Streptococcus pneumoniae OXC141 uid162037
Streptococcus pneumoniae 670 6B uid52533
Streptococcus pneumoniae AP200 uid52453
Streptococcus pneumoniae ATCC 700669 uid59287
Streptococcus pneumoniae D39 uid58581
Streptococcus pneumoniae G54 uid59167
Streptococcus pneumoniae INV104 uid162039
Streptococcus pneumoniae INV200 uid162035
Streptococcus pneumoniae JJA uid59121
Streptococcus pneumoniae P1031 uid59123
Streptococcus pneumoniae R6 uid57859
Streptococcus pneumoniae ST556 uid162191
Streptococcus pneumoniae TCH8431 19A uid49735
Streptococcus pneumoniae TIGR4 uid57857
Streptococcus pneumoniae Taiwan19F 14 uid59119
Streptococcus pseudopneumoniae IS7493 uid71153

rplP

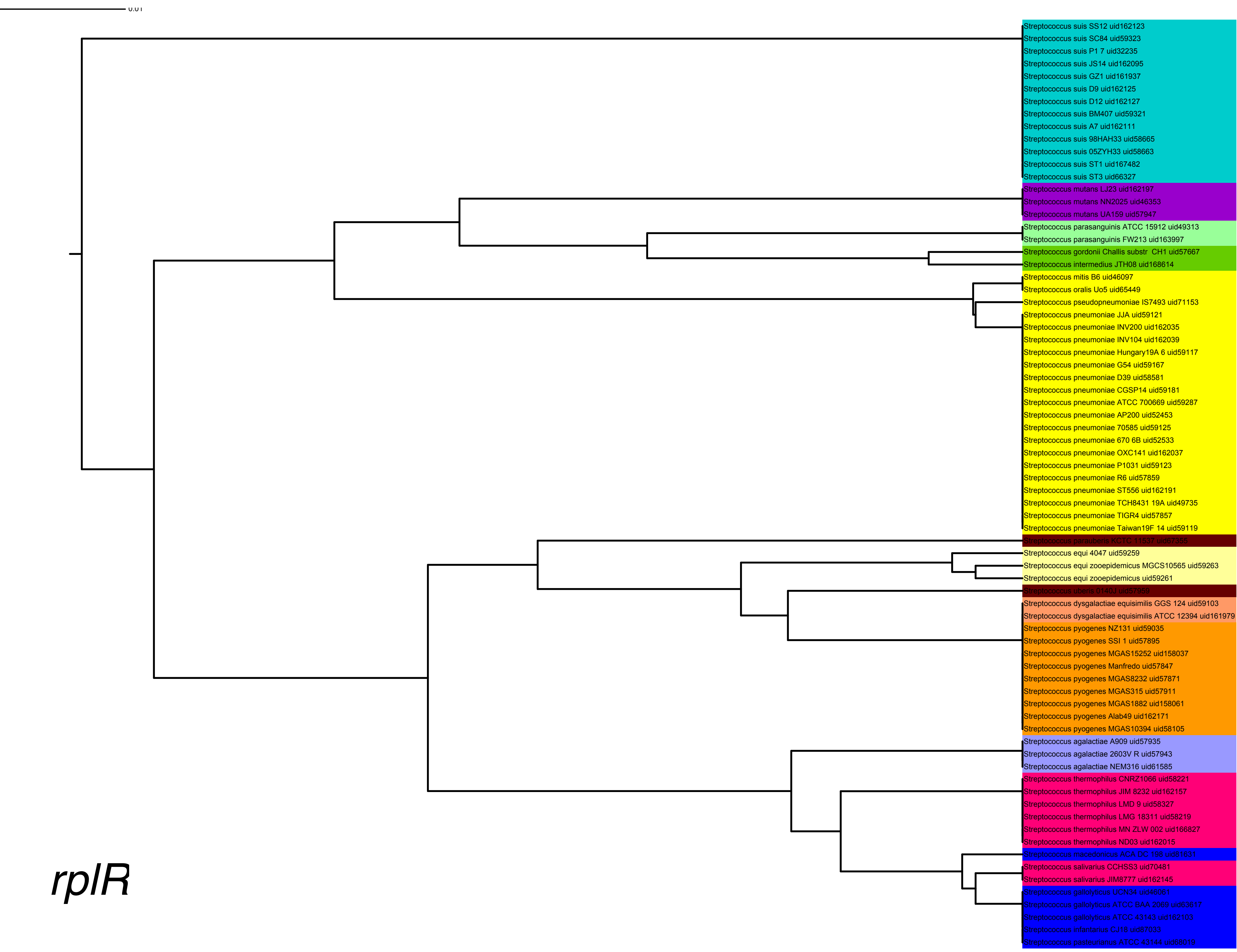
0.01
Streptococcus suis SS12 uid162123
Streptococcus suis SC84 uid59323
Streptococcus suis P1 7 uid32235
Streptococcus suis JS14 uid162095
Streptococcus suis GZ1 uid161937
Streptococcus suis D9 uid162125
Streptococcus suis D12 uid162127
Streptococcus suis BM407 uid59321
Streptococcus suis A7 uid162111
Streptococcus suis 98HAH33 uid58665
Streptococcus suis 05ZYH33 uid58663
Streptococcus suis ST1 uid167482
Streptococcus suis ST3 uid66327
Streptococcus mutans LJ23 uid162197
Streptococcus mutans NN2025 uid46353
Streptococcus mutans UA159 uid57947
Streptococcus parasanguinis ATCC 15912 uid49313
Streptococcus parasanguinis FW213 uid163997
Streptococcus gordonii Challis substr CH1 uid57667
Streptococcus intermedius JTH08 uid168614
Streptococcus mitis B6 uid46097
Streptococcus oralis Uo5 uid65449
Streptococcus pseudopneumoniae IS7493 uid71153
Streptococcus pneumoniae JJA uid59121
Streptococcus pneumoniae INV200 uid162035
Streptococcus pneumoniae INV104 uid162039
Streptococcus pneumoniae Hungary19A 6 uid59117
Streptococcus pneumoniae G54 uid59167
Streptococcus pneumoniae D39 uid58581
Streptococcus pneumoniae CGSP14 uid59181
Streptococcus pneumoniae ATCC 700669 uid59287
Streptococcus pneumoniae AP200 uid52453
Streptococcus pneumoniae 70585 uid59125
Streptococcus pneumoniae 670 6B uid52533
Streptococcus pneumoniae OXC141 uid162037
Streptococcus pneumoniae P1031 uid59123
Streptococcus pneumoniae R6 uid57859
Streptococcus pneumoniae ST556 uid162191
Streptococcus pneumoniae TCH8431 19A uid49735
Streptococcus pneumoniae TIGR4 uid57857
Streptococcus pneumoniae Taiwan19F 14 uid59119
Streptococcus parauberis KCTC 11537 uid67355
Streptococcus equi 4047 uid59259
Streptococcus equi zooepidemicus MGCS10565 uid59263
Streptococcus equi zooepidemicus uid59261
Streptococcus uberis 0140J uid57959
Streptococcus dysgalactiae equisimilis GGS 124 uid59103
Streptococcus dysgalactiae equisimilis ATCC 12394 uid161979
Streptococcus pyogenes NZ131 uid59035
Streptococcus pyogenes SSI 1 uid57895
Streptococcus pyogenes MGAS15252 uid158037
Streptococcus pyogenes Manfredo uid57847
Streptococcus pyogenes MGAS8232 uid57871
Streptococcus pyogenes MGAS315 uid57911
Streptococcus pyogenes MGAS1882 uid158061
Streptococcus pyogenes Alab49 uid162171
Streptococcus pyogenes MGAS10394 uid58105
Streptococcus agalactiae A909 uid57935
Streptococcus agalactiae 2603V R uid57943
Streptococcus agalactiae NEM316 uid61585
Streptococcus thermophilus CNRZ1066 uid58221
Streptococcus thermophilus JIM 8232 uid162157
Streptococcus thermophilus LMD 9 uid58327
Streptococcus thermophilus LMG 18311 uid58219
Streptococcus thermophilus MN ZLW 002 uid166827
Streptococcus thermophilus ND03 uid162015
Streptococcus macedonicus ACA DC 198 uid81631
Streptococcus salivarius CCHSS3 uid70481
Streptococcus salivarius JIM8777 uid162145
Streptococcus gallolyticus UCN34 uid46061
Streptococcus gallolyticus ATCC BAA 2069 uid63617
Streptococcus gallolyticus ATCC 43143 uid162103
Streptococcus infantarius CJ18 uid87033
Streptococcus pasteurianus ATCC 43144 uid68019
rplR

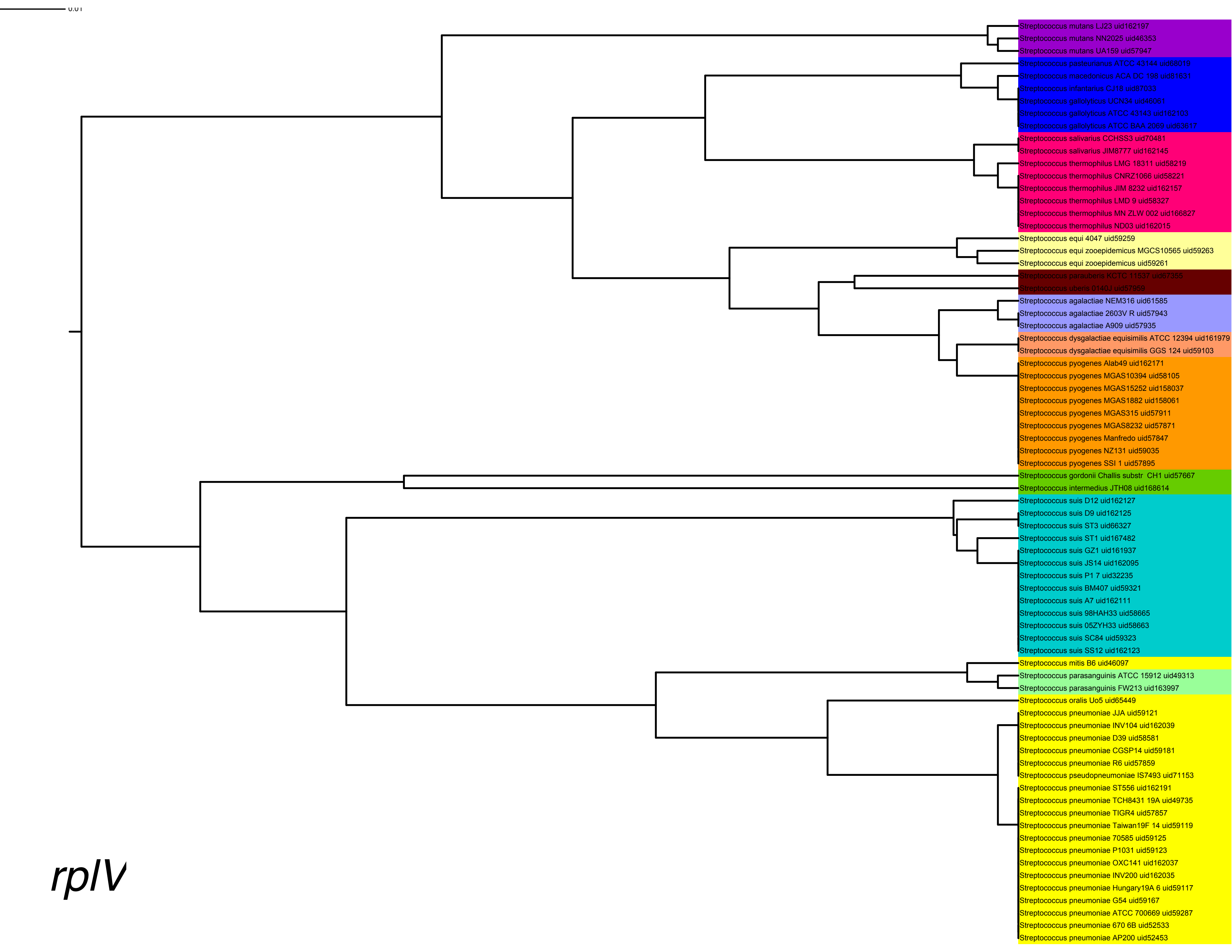
0.01
Streptococcus mutans LJ23 uid162197
Streptococcus mutans NN2025 uid46353
Streptococcus mutans UA159 uid57947
Streptococcus pasteurianus ATCC 43144 uid69019
Streptococcus macedonicus ACA DC 198 uid81631
Streptococcus infantarius CJ18 uid87033
Streptococcus gallolyticus UCN34 uid46061
Streptococcus gallolyticus ATCC 43143 uid162103
Streptococcus gallolyticus ATCC BAA 2069 uid63617
Streptococcus salivarius CCHSS3 uid70481
Streptococcus salivarius JIM8777 uid162145
Streptococcus thermophilus LMG 18311 uid58219
Streptococcus thermophilus CNRZ1066 uid58221
Streptococcus thermophilus JIM 8232 uid162157
Streptococcus thermophilus LMD 9 uid58327
Streptococcus thermophilus MN ZLW 002 uid166827
Streptococcus thermophilus ND03 uid162015
Streptococcus equi 4047 uid59259
Streptococcus equi zooepidemicus MGCS10565 uid59263
Streptococcus equi zooepidemicus uid59261
Streptococcus parauberis KCTC 11537 uid67355
Streptococcus uberis 0140J uid57959
Streptococcus agalactiae NEM316 uid61585
Streptococcus agalactiae 2603V R uid57943
Streptococcus agalactiae A909 uid57935
Streptococcus dysgalactiae equisimilis ATCC 12394 uid161979
Streptococcus dysgalactiae equisimilis GGS 124 uid59103
Streptococcus pyogenes Alab49 uid162171
Streptococcus pyogenes MGAS10394 uid58105
Streptococcus pyogenes MGAS15252 uid158037
Streptococcus pyogenes MGAS1882 uid158061
Streptococcus pyogenes MGAS315 uid57911
Streptococcus pyogenes MGAS8232 uid57871
Streptococcus pyogenes Manfredo uid57847
Streptococcus pyogenes NZ131 uid59035
Streptococcus pyogenes SSI 1 uid57895
Streptococcus gordonii Challis substr CH1 uid57667
Streptococcus intermedius JTH08 uid168614
Streptococcus suis D12 uid162127
Streptococcus suis D9 uid162125
Streptococcus suis ST3 uid66327
Streptococcus suis ST1 uid167482
Streptococcus suis GZ1 uid161937
Streptococcus suis JS14 uid162095
Streptococcus suis P1 7 uid32235
Streptococcus suis BM407 uid59321
Streptococcus suis A7 uid162111
Streptococcus suis 98HAH33 uid58665
Streptococcus suis 05ZYH33 uid58663
Streptococcus suis SC84 uid59323
Streptococcus suis SS12 uid162123
Streptococcus mitis B6 uid46097
Streptococcus parasanguinis ATCC 15912 uid49313
Streptococcus parasanguinis FW213 uid163997
Streptococcus oralis Uo5 uid65449
Streptococcus pneumoniae JJA uid59121
Streptococcus pneumoniae INV104 uid162039
Streptococcus pneumoniae D39 uid58581
Streptococcus pneumoniae CGSP14 uid59181
Streptococcus pneumoniae R6 uid57859
Streptococcus pseudopneumoniae IS7493 uid71153
Streptococcus pneumoniae ST556 uid162191
Streptococcus pneumoniae TCH8431 19A uid49735
Streptococcus pneumoniae TIGR4 uid57857
Streptococcus pneumoniae Taiwan19F 14 uid59119
Streptococcus pneumoniae 70585 uid59125
Streptococcus pneumoniae P1031 uid59123
Streptococcus pneumoniae OXC141 uid162037
Streptococcus pneumoniae INV200 uid162035
Streptococcus pneumoniae Hungary19A 6 uid59117
Streptococcus pneumoniae G54 uid59167
Streptococcus pneumoniae ATCC 700669 uid59287
Streptococcus pneumoniae 670 6B uid52533
Streptococcus pneumoniae AP200 uid52453
rplV

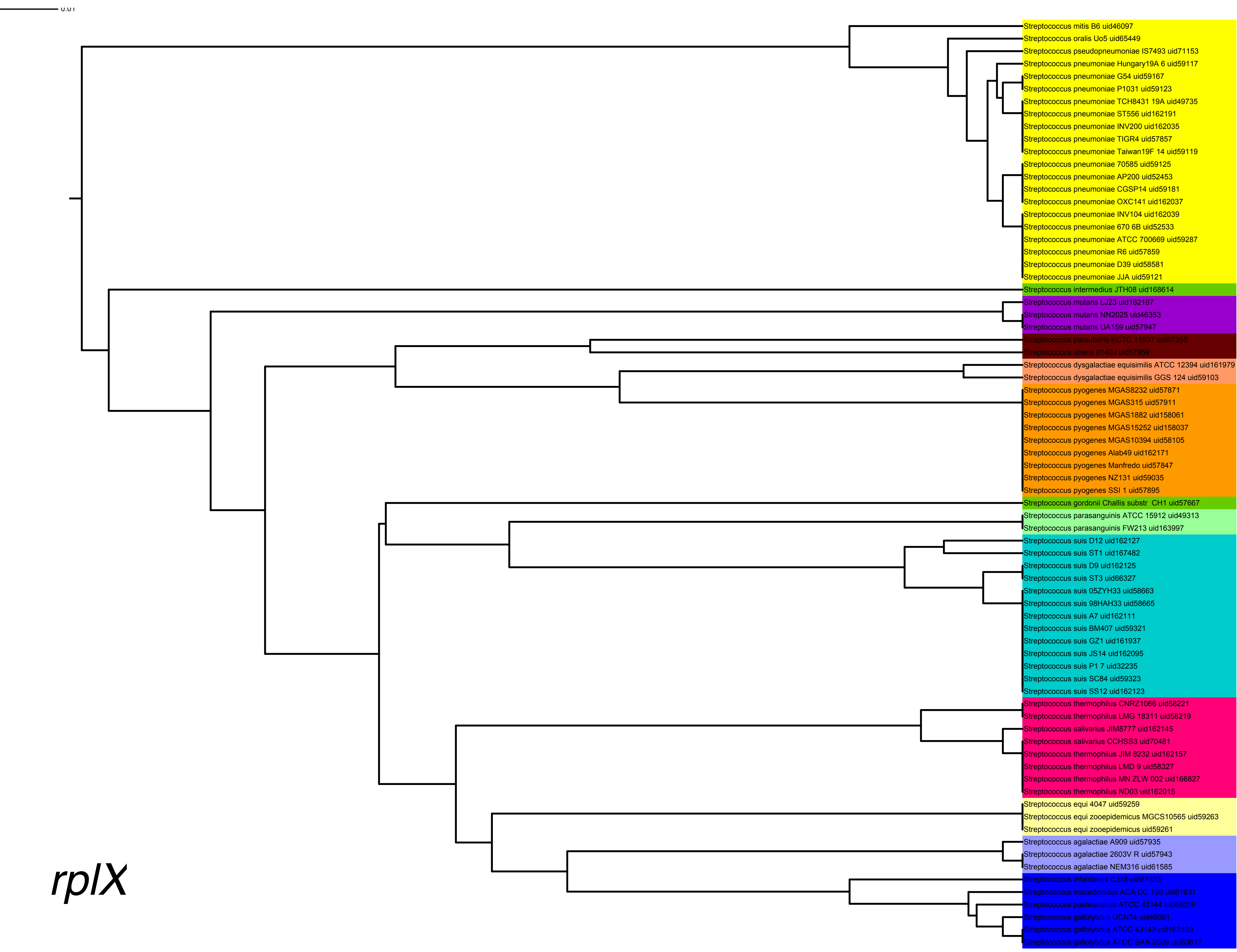
0.01
Streptococcus mitis B6 uid46097
Streptococcus oralis Uo5 uid65449
Streptococcus pseudopneumoniae IS7493 uid71153
Streptococcus pneumoniae Hungary19A 6 uid59117
Streptococcus pneumoniae G54 uid59167
Streptococcus pneumoniae P1031 uid59123
Streptococcus pneumoniae TCH8431 19A uid49735
Streptococcus pneumoniae ST556 uid162191
Streptococcus pneumoniae INV200 uid162035
Streptococcus pneumoniae TIGR4 uid57857
Streptococcus pneumoniae Taiwan19F 14 uid59119
Streptococcus pneumoniae 70585 uid59125
Streptococcus pneumoniae AP200 uid52453
Streptococcus pneumoniae CGSP14 uid59181
Streptococcus pneumoniae OXC141 uid162037
Streptococcus pneumoniae INV104 uid162039
Streptococcus pneumoniae 670 6B uid52533
Streptococcus pneumoniae ATCC 700669 uid59287
Streptococcus pneumoniae R6 uid57859
Streptococcus pneumoniae D39 uid58581
Streptococcus pneumoniae JJA uid59121
Streptococcus intermedius JTH08 uid168614
Streptococcus mutans LJ23 uid162197
Streptococcus mutans NN2025 uid46353
Streptococcus mutans UA159 uid57947
Streptococcus parauberis KCTC 11537 uid67355
Streptococcus uberis 0140J uid57959
Streptococcus dysgalactiae equisimilis ATCC 12394 uid161979
Streptococcus dysgalactiae equisimilis GGS 124 uid59103
Streptococcus pyogenes MGAS8232 uid57871
Streptococcus pyogenes MGAS315 uid57911
Streptococcus pyogenes MGAS1882 uid158061
Streptococcus pyogenes MGAS15252 uid158037
Streptococcus pyogenes MGAS10394 uid58105
Streptococcus pyogenes Alab49 uid162171
Streptococcus pyogenes Manfredo uid57847
Streptococcus pyogenes NZ131 uid59035
Streptococcus pyogenes SSI 1 uid57895
Streptococcus gordonii Challis substr CH1 uid57667
Streptococcus parasanguinis ATCC 15912 uid49313
Streptococcus parasanguinis FW213 uid163997
Streptococcus suis D12 uid162127
Streptococcus suis ST1 uid167482
Streptococcus suis D9 uid162125
Streptococcus suis ST3 uid66327
Streptococcus suis 05ZYH33 uid58663
Streptococcus suis 98HAH33 uid58665
Streptococcus suis A7 uid162111
Streptococcus suis BM407 uid59321
Streptococcus suis GZ1 uid161937
Streptococcus suis JS14 uid162095
Streptococcus suis P1 7 uid32235
Streptococcus suis SC84 uid59323
Streptococcus suis SS12 uid162123
Streptococcus thermophilus CNRZ1066 uid58221
Streptococcus thermophilus LMG 18311 uid58219
Streptococcus salivarius JIM8777 uid162145
Streptococcus salivarius CCHSS3 uid70481
Streptococcus thermophilus JIM 8232 uid162157
Streptococcus thermophilus LMD 9 uid58327
Streptococcus thermophilus MN ZLW 002 uid166827
Streptococcus thermophilus ND03 uid162015
Streptococcus equi 4047 uid59259
Streptococcus equi zooepidemicus MGCS10565 uid59263
Streptococcus equi zooepidemicus uid59261
Streptococcus agalactiae A909 uid57935
Streptococcus agalactiae 2603V R uid57943
Streptococcus agalactiae NEM316 uid61585
Streptococcus infantarius CJ18 uid87033
Streptococcus macedonicus ACA DC 198 uid81631
Streptococcus pasteurianus ATCC 43144 uid68019
Streptococcus gallolyticus UCN34 uid46061
Streptococcus gallolyticus ATCC 43143 uid162103
Streptococcus gallolyticus ATCC BAA 2069 uid63617
rplX

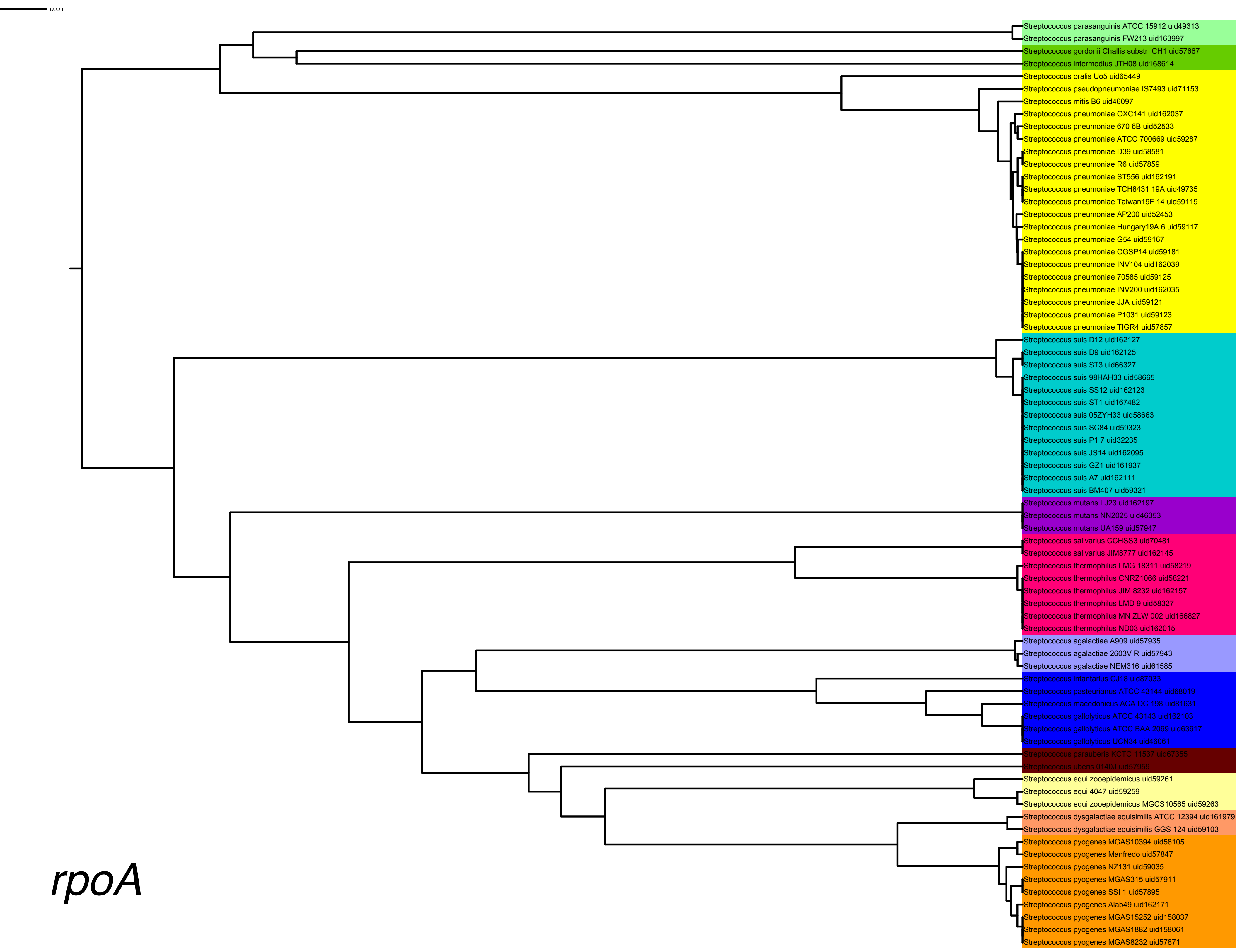
0.01
Streptococcus parasanguinis ATCC 15912 uid49313
Streptococcus parasanguinis FW213 uid163997
Streptococcus gordonii Challis substr CH1 uid57667
Streptococcus intermedius JTH08 uid168614
Streptococcus oralis Uo5 uid65449
Streptococcus pseudopneumoniae IS7493 uid71153
Streptococcus mitis B6 uid46097
Streptococcus pneumoniae OXC141 uid162037
Streptococcus pneumoniae 670 6B uid52533
Streptococcus pneumoniae ATCC 700669 uid59287
Streptococcus pneumoniae D39 uid58581
Streptococcus pneumoniae R6 uid57859
Streptococcus pneumoniae ST556 uid162191
Streptococcus pneumoniae TCH8431 19A uid49735
Streptococcus pneumoniae Taiwan19F 14 uid59119
Streptococcus pneumoniae AP200 uid52453
Streptococcus pneumoniae Hungary19A 6 uid59117
Streptococcus pneumoniae G54 uid59167
Streptococcus pneumoniae CGSP14 uid59181
Streptococcus pneumoniae INV104 uid162039
Streptococcus pneumoniae 70585 uid59125
Streptococcus pneumoniae INV200 uid162035
Streptococcus pneumoniae JJA uid59121
Streptococcus pneumoniae P1031 uid59123
Streptococcus pneumoniae TIGR4 uid57857
Streptococcus suis D12 uid162127
Streptococcus suis D9 uid162125
Streptococcus suis ST3 uid66327
Streptococcus suis 98HAH33 uid58665
Streptococcus suis SS12 uid162123
Streptococcus suis ST1 uid167482
Streptococcus suis 05ZYH33 uid58663
Streptococcus suis SC84 uid59323
Streptococcus suis P1 7 uid32235
Streptococcus suis JS14 uid162095
Streptococcus suis GZ1 uid161937
Streptococcus suis A7 uid162111
Streptococcus suis BM407 uid59321
Streptococcus mutans LJ23 uid162197
Streptococcus mutans NN2025 uid46353
Streptococcus mutans UA159 uid57947
Streptococcus salivarius CCHSS3 uid70481
Streptococcus salivarius JIM8777 uid162145
Streptococcus thermophilus LMG 18311 uid58219
Streptococcus thermophilus CNRZ1066 uid58221
Streptococcus thermophilus JIM 8232 uid162157
Streptococcus thermophilus LMD 9 uid58327
Streptococcus thermophilus MN ZLW 002 uid166827
Streptococcus thermophilus ND03 uid162015
Streptococcus agalactiae A909 uid57935
Streptococcus agalactiae 2603V R uid57943
Streptococcus agalactiae NEM316 uid61585
Streptococcus infantarius CJ18 uid87033
Streptococcus pasteurianus ATCC 43144 uid68019
Streptococcus macedonicus ACA DC 198 uid81631
Streptococcus gallolyticus ATCC 43143 uid162103
Streptococcus gallolyticus ATCC BAA 2069 uid63617
Streptococcus gallolyticus UCN34 uid46061
Streptococcus parauberis KCTC 11537 uid67355
Streptococcus uberis 0140J uid57959
Streptococcus equi zooepidemicus uid59261
Streptococcus equi 4047 uid59259
Streptococcus equi zooepidemicus MGCS10565 uid59263
Streptococcus dysgalactiae equisimilis ATCC 12394 uid161979
Streptococcus dysgalactiae equisimilis GGS 124 uid59103
Streptococcus pyogenes MGAS10394 uid58105
Streptococcus pyogenes Manfredo uid57847
Streptococcus pyogenes NZ131 uid59035
Streptococcus pyogenes MGAS315 uid57911
Streptococcus pyogenes SSI 1 uid57895
Streptococcus pyogenes Alab49 uid162171
Streptococcus pyogenes MGAS15252 uid158037
Streptococcus pyogenes MGAS1882 uid158061
Streptococcus pyogenes MGAS8232 uid57871
rpoA

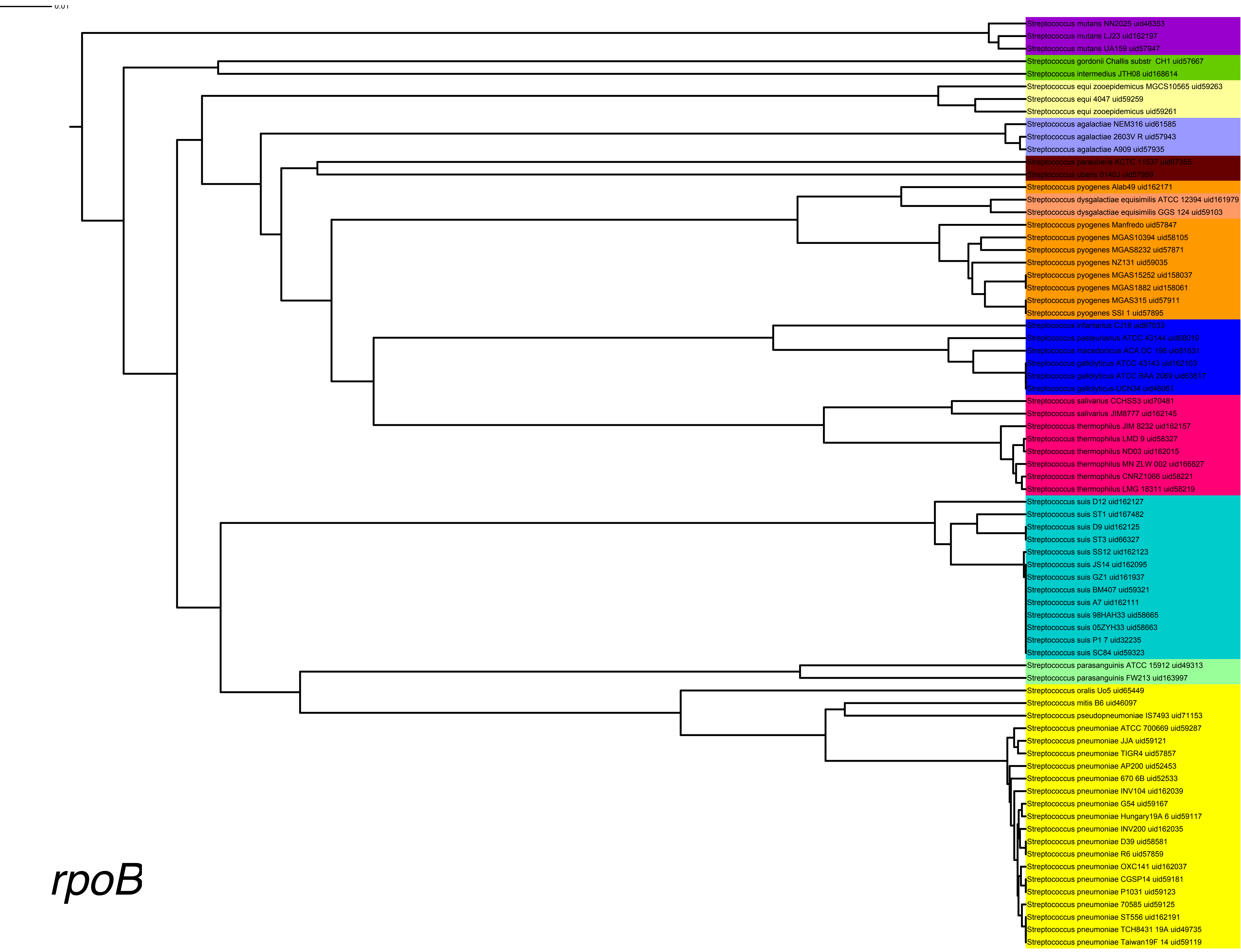
0.01
Streptococcus mutans NN2025 uid46353
Streptococcus mutans LJ23 uid162197
Streptococcus mutans UA159 uid57947
Streptococcus gordonii Challis substr CH1 uid57667
Streptococcus intermedius JTH08 uid168614
Streptococcus equi zooepidemicus MGCS10565 uid59263
Streptococcus equi 4047 uid59259
Streptococcus equi zooepidemicus uid59261
Streptococcus agalactiae NEM316 uid61585
Streptococcus agalactiae 2603V R uid57943
Streptococcus agalactiae A909 uid57935
Streptococcus parauberis KCTC 11537 uid67355
Streptococcus uberis 0140J uid57959
Streptococcus pyogenes Alab49 uid162171
Streptococcus dysgalactiae equisimilis ATCC 12394 uid161979
Streptococcus dysgalactiae equisimilis GGS 124 uid59103
Streptococcus pyogenes Manfredo uid57847
Streptococcus pyogenes MGAS10394 uid58105
Streptococcus pyogenes MGAS8232 uid57871
Streptococcus pyogenes NZ131 uid59035
Streptococcus pyogenes MGAS15252 uid158037
Streptococcus pyogenes MGAS1882 uid158061
Streptococcus pyogenes MGAS315 uid57911
Streptococcus pyogenes SSI 1 uid57895
Streptococcus infantarius CJ18 uid87033
Streptococcus pasteurianus ATCC 43144 uid68019
Streptococcus macedonicus ACA DC 198 uid81631
Streptococcus gallolyticus ATCC 43143 uid162103
Streptococcus gallolyticus ATCC BAA 2069 uid63617
Streptococcus gallolyticus UCN34 uid46061
Streptococcus salivarius CCHSS3 uid70481
Streptococcus salivarius JIM8777 uid162145
Streptococcus thermophilus JIM 8232 uid162157
Streptococcus thermophilus LMD 9 uid58327
Streptococcus thermophilus ND03 uid162015
Streptococcus thermophilus MN ZLW 002 uid166827
Streptococcus thermophilus CNRZ1066 uid58221
Streptococcus thermophilus LMG 18311 uid58219
Streptococcus suis D12 uid162127
Streptococcus suis ST1 uid167482
Streptococcus suis D9 uid162125
Streptococcus suis ST3 uid66327
Streptococcus suis SS12 uid162123
Streptococcus suis JS14 uid162095
Streptococcus suis GZ1 uid161937
Streptococcus suis BM407 uid59321
Streptococcus suis A7 uid162111
Streptococcus suis 98HAH33 uid58665
Streptococcus suis 05ZYH33 uid58663
Streptococcus suis P1 7 uid32235
Streptococcus suis SC84 uid59323
Streptococcus parasanguinis ATCC 15912 uid49313
Streptococcus parasanguinis FW213 uid163997
Streptococcus oralis Uo5 uid65449
Streptococcus mitis B6 uid46097
Streptococcus pseudopneumoniae IS7493 uid71153
Streptococcus pneumoniae ATCC 700669 uid59287
Streptococcus pneumoniae JJA uid59121
Streptococcus pneumoniae TIGR4 uid57857
Streptococcus pneumoniae AP200 uid52453
Streptococcus pneumoniae 670 6B uid52533
Streptococcus pneumoniae INV104 uid162039
Streptococcus pneumoniae G54 uid59167
Streptococcus pneumoniae Hungary19A 6 uid59117
Streptococcus pneumoniae INV200 uid162035
Streptococcus pneumoniae D39 uid58581
Streptococcus pneumoniae R6 uid57859
Streptococcus pneumoniae OXC141 uid162037
Streptococcus pneumoniae CGSP14 uid59181
Streptococcus pneumoniae P1031 uid59123
Streptococcus pneumoniae 70585 uid59125
Streptococcus pneumoniae ST556 uid162191
Streptococcus pneumoniae TCH8431 19A uid49735
Streptococcus pneumoniae Taiwan19F 14 uid59119
rpoB

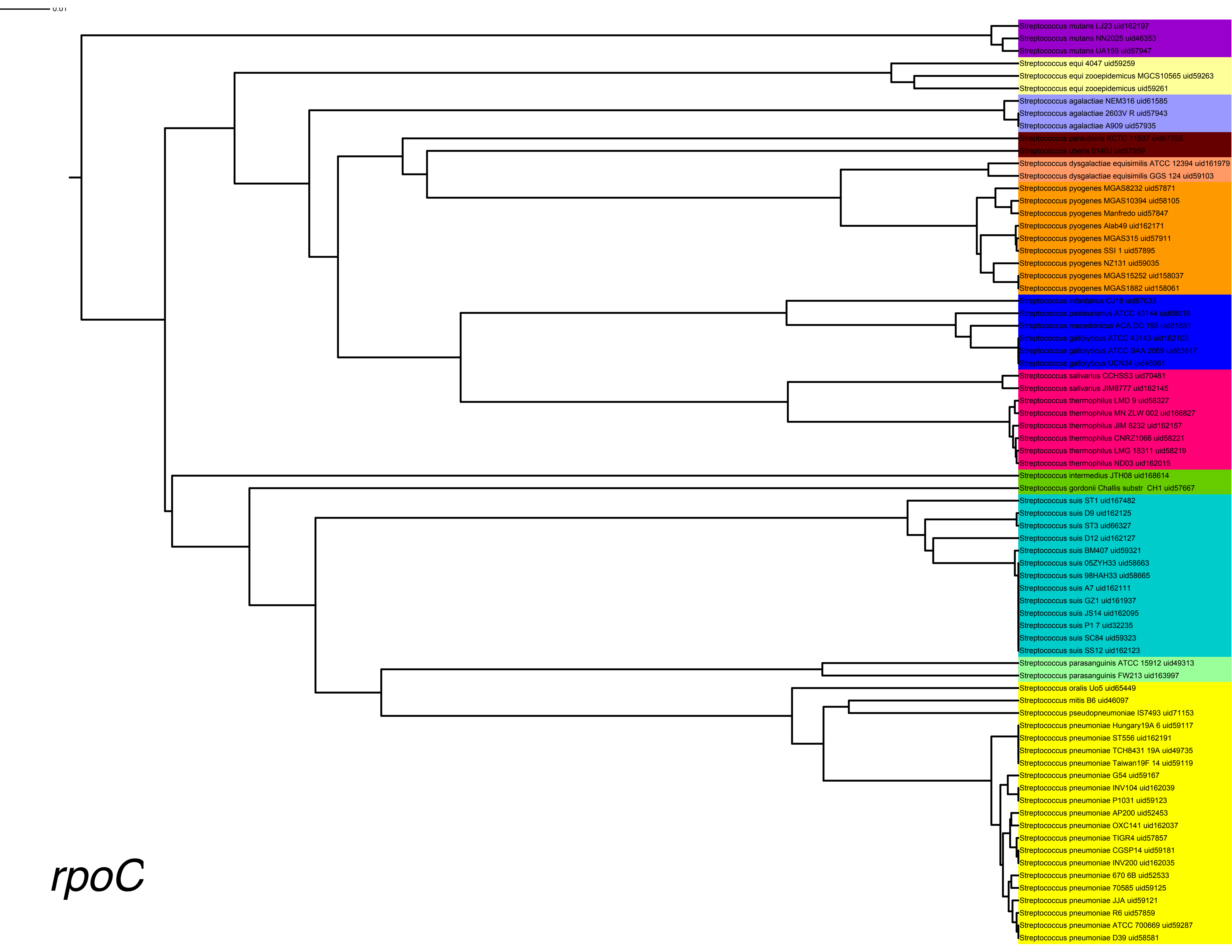
0.01
Streptococcus mutans LJ23 uid162197
Streptococcus mutans NN2025 uid46353
Streptococcus mutans UA159 uid57947
Streptococcus equi 4047 uid59259
Streptococcus equi zooepidemicus MGCS10565 uid59263
Streptococcus equi zooepidemicus uid59261
Streptococcus agalactiae NEM316 uid61585
Streptococcus agalactiae 2603V R uid57943
Streptococcus agalactiae A909 uid57935
Streptococcus parauberis KCTC 11537 uid67355
Streptococcus uberis 0140J uid57959
Streptococcus dysgalactiae equisimilis ATCC 12394 uid161979
Streptococcus dysgalactiae equisimilis GGS 124 uid59103
Streptococcus pyogenes MGAS8232 uid57871
Streptococcus pyogenes MGAS10394 uid58105
Streptococcus pyogenes Manfredo uid57847
Streptococcus pyogenes Alab49 uid162171
Streptococcus pyogenes MGAS315 uid57911
Streptococcus pyogenes SSI 1 uid57895
Streptococcus pyogenes NZ131 uid59035
Streptococcus pyogenes MGAS15252 uid158037
Streptococcus pyogenes MGAS1882 uid158061
Streptococcus infantarius CJ18 uid87033
Streptococcus pasteurianus ATCC 43144 uid68019
Streptococcus macedonicus ACA DC 198 uid81631
Streptococcus gallolyticus ATCC 43143 uid162103
Streptococcus gallolyticus ATCC BAA 2069 uid63617
Streptococcus gallolyticus UCN34 uid46061
Streptococcus salivarius CCHSS3 uid70481
Streptococcus salivarius JIM8777 uid162145
Streptococcus thermophilus LMD 9 uid58327
Streptococcus thermophilus MN ZLW 002 uid166827
Streptococcus thermophilus JIM 8232 uid162157
Streptococcus thermophilus CNRZ1066 uid58221
Streptococcus thermophilus LMG 18311 uid58219
Streptococcus thermophilus ND03 uid162015
Streptococcus intermedius JTH08 uid168614
Streptococcus gordonii Challis substr CH1 uid57667
Streptococcus suis ST1 uid167482
Streptococcus suis D9 uid162125
Streptococcus suis ST3 uid66327
Streptococcus suis D12 uid162127
Streptococcus suis BM407 uid59321
Streptococcus suis 05ZYH33 uid58663
Streptococcus suis 98HAH33 uid58665
Streptococcus suis A7 uid162111
Streptococcus suis GZ1 uid161937
Streptococcus suis JS14 uid162095
Streptococcus suis P1 7 uid32235
Streptococcus suis SC84 uid59323
Streptococcus suis SS12 uid162123
Streptococcus parasanguinis ATCC 15912 uid49313
Streptococcus parasanguinis FW213 uid163997
Streptococcus oralis Uo5 uid65449
Streptococcus mitis B6 uid46097
Streptococcus pseudopneumoniae IS7493 uid71153
Streptococcus pneumoniae Hungary19A 6 uid59117
Streptococcus pneumoniae ST556 uid162191
Streptococcus pneumoniae TCH8431 19A uid49735
Streptococcus pneumoniae Taiwan19F 14 uid59119
Streptococcus pneumoniae G54 uid59167
Streptococcus pneumoniae INV104 uid162039
Streptococcus pneumoniae P1031 uid59123
Streptococcus pneumoniae AP200 uid52453
Streptococcus pneumoniae OXC141 uid162037
Streptococcus pneumoniae TIGR4 uid57857
Streptococcus pneumoniae CGSP14 uid59181
Streptococcus pneumoniae INV200 uid162035
Streptococcus pneumoniae 670 6B uid52533
Streptococcus pneumoniae 70585 uid59125
Streptococcus pneumoniae JJA uid59121
Streptococcus pneumoniae R6 uid57859
Streptococcus pneumoniae ATCC 700669 uid59287
Streptococcus pneumoniae D39 uid58581
rpoC

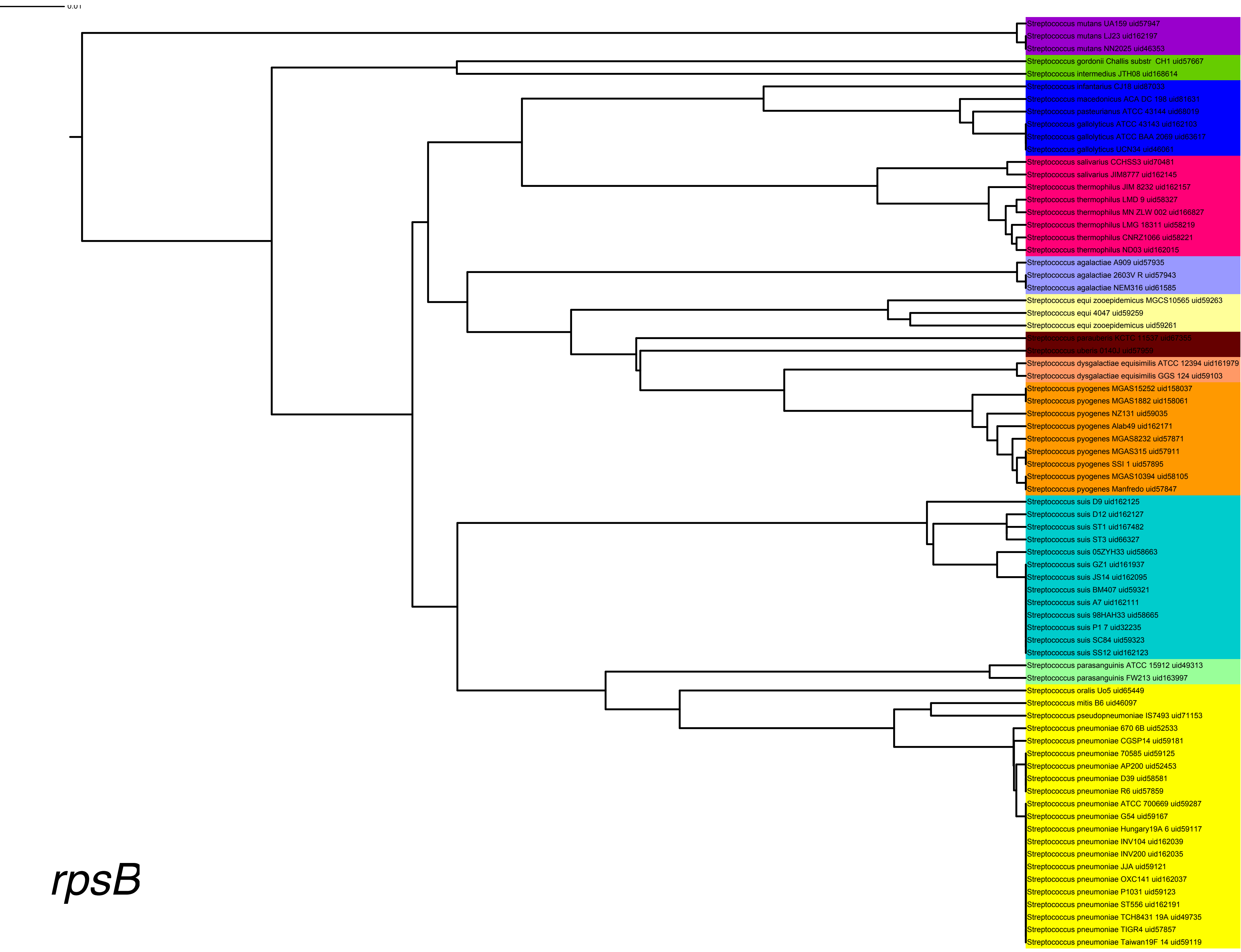
0.01
Streptococcus mutans UA159 uid57947
Streptococcus mutans LJ23 uid162197
Streptococcus mutans NN2025 uid46353
Streptococcus gordonii Challis substr CH1 uid57667
Streptococcus intermedius JTH08 uid168614
Streptococcus infantarius CJ18 uid87033
Streptococcus macedonicus ACA DC 198 uid81631
Streptococcus pasteurianus ATCC 43144 uid68019
Streptococcus gallolyticus ATCC 43143 uid162103
Streptococcus gallolyticus ATCC BAA 2069 uid63617
Streptococcus gallolyticus UCN34 uid46061
Streptococcus salivarius CCHSS3 uid70481
Streptococcus salivarius JIM8777 uid162145
Streptococcus thermophilus JIM 8232 uid162157
Streptococcus thermophilus LMD 9 uid58327
Streptococcus thermophilus MN ZLW 002 uid166827
Streptococcus thermophilus LMG 18311 uid58219
Streptococcus thermophilus CNRZ1066 uid58221
Streptococcus thermophilus ND03 uid162015
Streptococcus agalactiae A909 uid57935
Streptococcus agalactiae 2603V R uid57943
Streptococcus agalactiae NEM316 uid61585
Streptococcus equi zooepidemicus MGCS10565 uid59263
Streptococcus equi 4047 uid59259
Streptococcus equi zooepidemicus uid59261
Streptococcus parauberis KCTC 11537 uid67355
Streptococcus uberis 0140J uid57959
Streptococcus dysgalactiae equisimilis ATCC 12394 uid161979
Streptococcus dysgalactiae equisimilis GGS 124 uid59103
Streptococcus pyogenes MGAS15252 uid158037
Streptococcus pyogenes MGAS1882 uid158061
Streptococcus pyogenes NZ131 uid59035
Streptococcus pyogenes Alab49 uid162171
Streptococcus pyogenes MGAS8232 uid57871
Streptococcus pyogenes MGAS315 uid57911
Streptococcus pyogenes SSI 1 uid57895
Streptococcus pyogenes MGAS10394 uid58105
Streptococcus pyogenes Manfredo uid57847
Streptococcus suis D9 uid162125
Streptococcus suis D12 uid162127
Streptococcus suis ST1 uid167482
Streptococcus suis ST3 uid66327
Streptococcus suis 05ZYH33 uid58663
Streptococcus suis GZ1 uid161937
Streptococcus suis JS14 uid162095
Streptococcus suis BM407 uid59321
Streptococcus suis A7 uid162111
Streptococcus suis 98HAH33 uid58665
Streptococcus suis P1 7 uid32235
Streptococcus suis SC84 uid59323
Streptococcus suis SS12 uid162123
Streptococcus parasanguinis ATCC 15912 uid49313
Streptococcus parasanguinis FW213 uid163997
Streptococcus oralis Uo5 uid65449
Streptococcus mitis B6 uid46097
Streptococcus pseudopneumoniae IS7493 uid71153
Streptococcus pneumoniae 670 6B uid52533
Streptococcus pneumoniae CGSP14 uid59181
Streptococcus pneumoniae 70585 uid59125
Streptococcus pneumoniae AP200 uid52453
Streptococcus pneumoniae D39 uid58581
Streptococcus pneumoniae R6 uid57859
Streptococcus pneumoniae ATCC 700669 uid59287
Streptococcus pneumoniae G54 uid59167
Streptococcus pneumoniae Hungary19A 6 uid59117
Streptococcus pneumoniae INV104 uid162039
Streptococcus pneumoniae INV200 uid162035
Streptococcus pneumoniae JJA uid59121
Streptococcus pneumoniae OXC141 uid162037
Streptococcus pneumoniae P1031 uid59123
Streptococcus pneumoniae ST556 uid162191
Streptococcus pneumoniae TCH8431 19A uid49735
Streptococcus pneumoniae TIGR4 uid57857
Streptococcus pneumoniae Taiwan19F 14 uid59119
rpsB

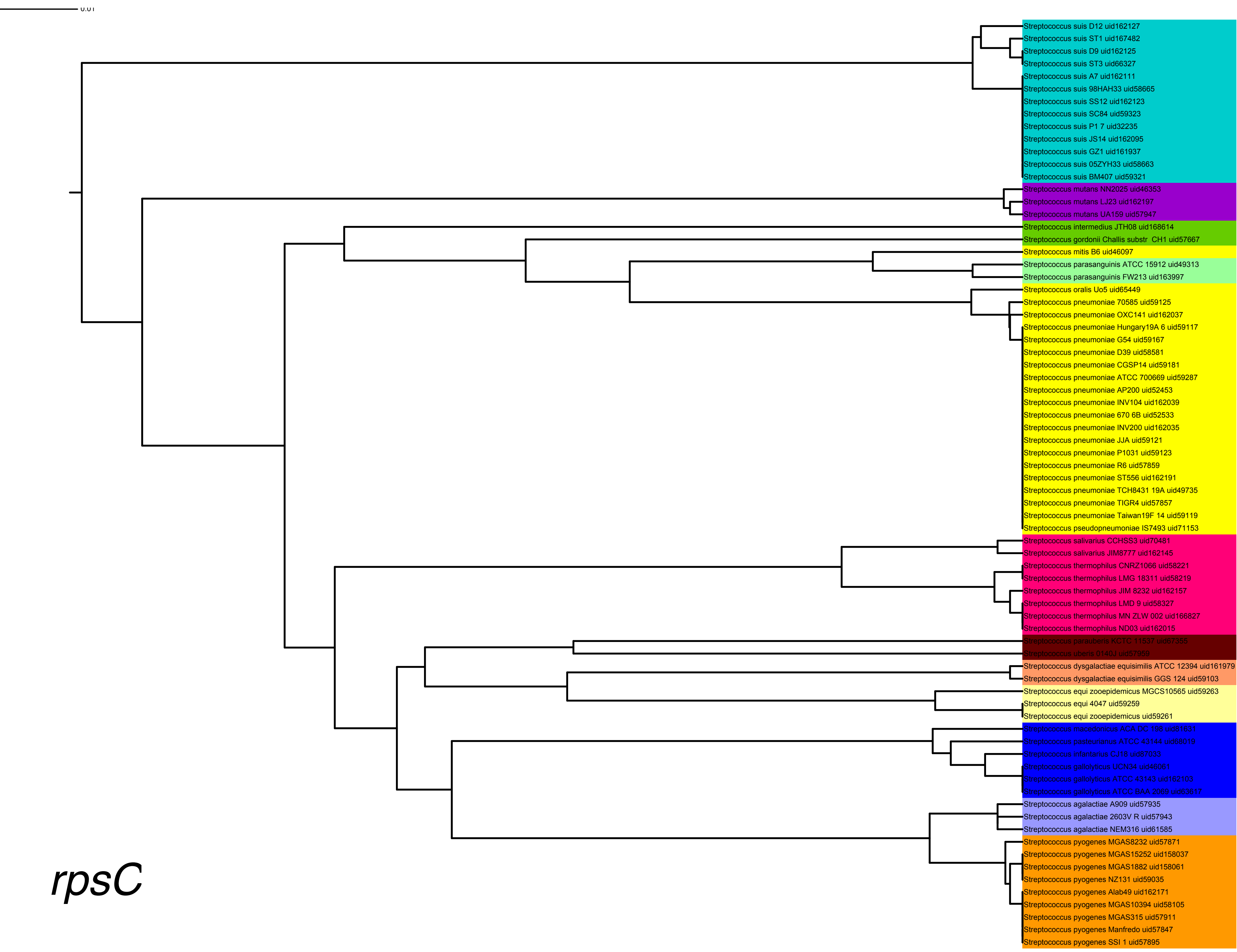
0.01
Streptococcus suis D12 uid162127
Streptococcus suis ST1 uid167482
Streptococcus suis D9 uid162125
Streptococcus suis ST3 uid66327
Streptococcus suis A7 uid162111
Streptococcus suis 98HAH33 uid58665
Streptococcus suis SS12 uid162123
Streptococcus suis SC84 uid59323
Streptococcus suis P1 7 uid32235
Streptococcus suis JS14 uid162095
Streptococcus suis GZ1 uid161937
Streptococcus suis 05ZYH33 uid58663
Streptococcus suis BM407 uid59321
Streptococcus mutans NN2025 uid46353
Streptococcus mutans LJ23 uid162197
Streptococcus mutans UA159 uid57947
Streptococcus intermedius JTH08 uid168614
Streptococcus gordonii Challis substr CH1 uid57667
Streptococcus mitis B6 uid46097
Streptococcus parasanguinis ATCC 15912 uid49313
Streptococcus parasanguinis FW213 uid163997
Streptococcus oralis Uo5 uid65449
Streptococcus pneumoniae 70585 uid59125
Streptococcus pneumoniae OXC141 uid162037
Streptococcus pneumoniae Hungary19A 6 uid59117
Streptococcus pneumoniae G54 uid59167
Streptococcus pneumoniae D39 uid58581
Streptococcus pneumoniae CGSP14 uid59181
Streptococcus pneumoniae ATCC 700669 uid59287
Streptococcus pneumoniae AP200 uid52453
Streptococcus pneumoniae INV104 uid162039
Streptococcus pneumoniae 670 6B uid52533
Streptococcus pneumoniae INV200 uid162035
Streptococcus pneumoniae JJA uid59121
Streptococcus pneumoniae P1031 uid59123
Streptococcus pneumoniae R6 uid57859
Streptococcus pneumoniae ST556 uid162191
Streptococcus pneumoniae TCH8431 19A uid49735
Streptococcus pneumoniae TIGR4 uid57857
Streptococcus pneumoniae Taiwan19F 14 uid59119
Streptococcus pseudopneumoniae IS7493 uid71153
Streptococcus salivarius CCHSS3 uid70481
Streptococcus salivarius JIM8777 uid162145
Streptococcus thermophilus CNRZ1066 uid58221
Streptococcus thermophilus LMG 18311 uid58219
Streptococcus thermophilus JIM 8232 uid162157
Streptococcus thermophilus LMD 9 uid58327
Streptococcus thermophilus MN ZLW 002 uid166827
Streptococcus thermophilus ND03 uid162015
Streptococcus parauberis KCTC 11537 uid67355
Streptococcus uberis 0140J uid57959
Streptococcus dysgalactiae equisimilis ATCC 12394 uid161979
Streptococcus dysgalactiae equisimilis GGS 124 uid59103
Streptococcus equi zooepidemicus MGCS10565 uid59263
Streptococcus equi 4047 uid59259
Streptococcus equi zooepidemicus uid59261
Streptococcus macedonicus ACA DC 198 uid81631
Streptococcus pasteurianus ATCC 43144 uid68019
Streptococcus infantarius CJ18 uid87033
Streptococcus gallolyticus UCN34 uid46061
Streptococcus gallolyticus ATCC 43143 uid162103
Streptococcus gallolyticus ATCC BAA 2069 uid63617
Streptococcus agalactiae A909 uid57935
Streptococcus agalactiae 2603V R uid57943
Streptococcus agalactiae NEM316 uid61585
Streptococcus pyogenes MGAS8232 uid57871
Streptococcus pyogenes MGAS15252 uid158037
Streptococcus pyogenes MGAS1882 uid158061
Streptococcus pyogenes NZ131 uid59035
Streptococcus pyogenes Alab49 uid162171
Streptococcus pyogenes MGAS10394 uid58105
Streptococcus pyogenes MGAS315 uid57911
Streptococcus pyogenes Manfredo uid57847
Streptococcus pyogenes SSI 1 uid57895
rpsC

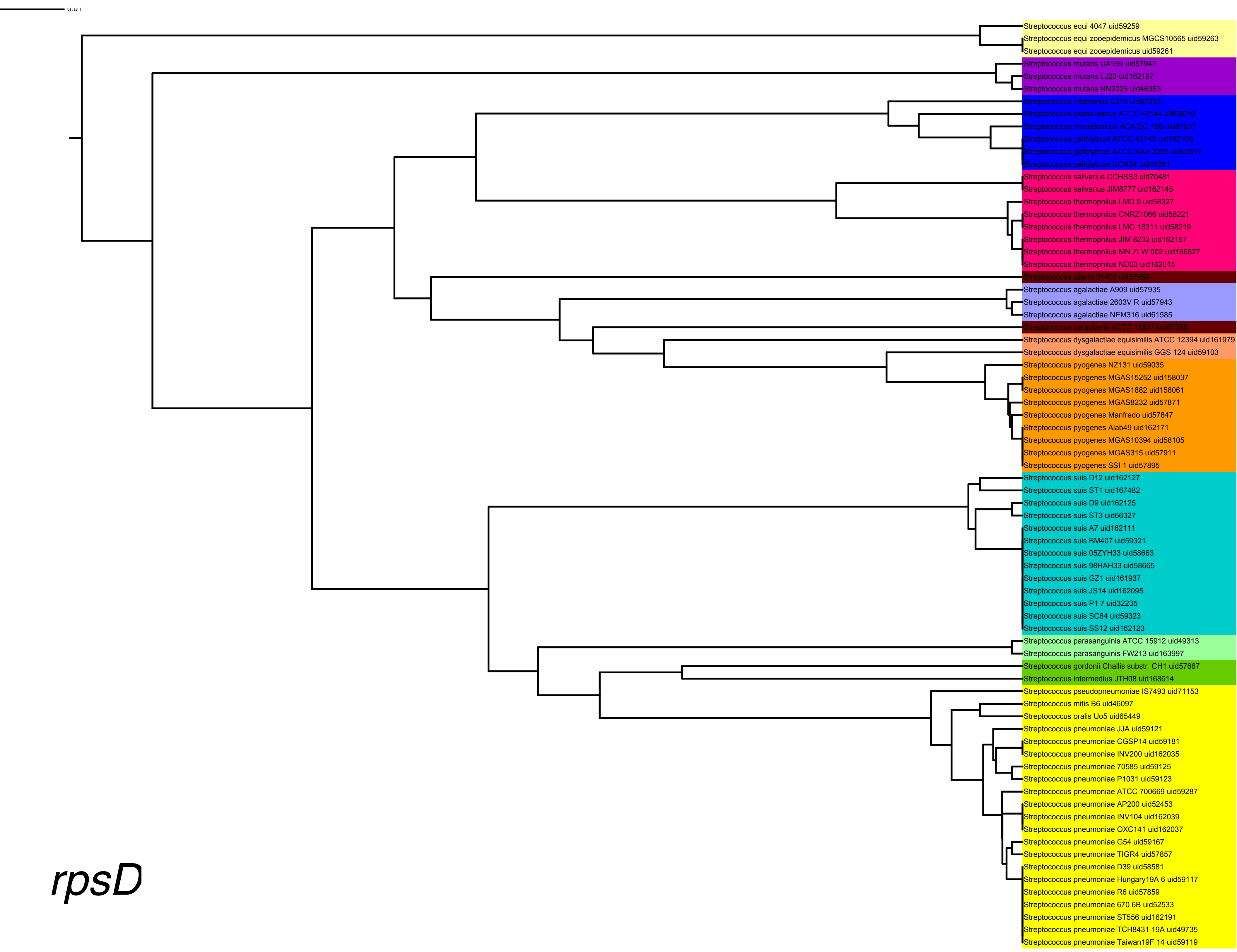
0.01
Streptococcus equi 4047 uid59259
Streptococcus equi zooepidemicus MGCS10565 uid59263
Streptococcus equi zooepidemicus uid59261
Streptococcus mutans UA159 uid57947
Streptococcus mutans LJ23 uid162197
Streptococcus mutans NN2025 uid46353
Streptococcus infantarius CJ18 uid87033
Streptococcus pasteurianus ATCC 43144 uid68019
Streptococcus macedonicus ACA DC 198 uid81631
Streptococcus gallolyticus ATCC 43143 uid162103
Streptococcus gallolyticus ATCC BAA 2069 uid63617
Streptococcus gallolyticus UCN34 uid46061
Streptococcus salivarius CCHSS3 uid70481
Streptococcus salivarius JIM8777 uid162145
Streptococcus thermophilus LMD 9 uid58327
Streptococcus thermophilus CNRZ1066 uid58221
Streptococcus thermophilus LMG 18311 uid58219
Streptococcus thermophilus JIM 8232 uid162157
Streptococcus thermophilus MN ZLW 002 uid166827
Streptococcus thermophilus ND03 uid162015
Streptococcus uberis 0140J uid57959
Streptococcus agalactiae A909 uid57935
Streptococcus agalactiae 2603V R uid57943
Streptococcus agalactiae NEM316 uid61585
Streptococcus parauberis KCTC 11537 uid67355
Streptococcus dysgalactiae equisimilis ATCC 12394 uid161979
Streptococcus dysgalactiae equisimilis GGS 124 uid59103
Streptococcus pyogenes NZ131 uid59035
Streptococcus pyogenes MGAS15252 uid158037
Streptococcus pyogenes MGAS1882 uid158061
Streptococcus pyogenes MGAS8232 uid57871
Streptococcus pyogenes Manfredo uid57847
Streptococcus pyogenes Alab49 uid162171
Streptococcus pyogenes MGAS10394 uid58105
Streptococcus pyogenes MGAS315 uid57911
Streptococcus pyogenes SSI 1 uid57895
Streptococcus suis D12 uid162127
Streptococcus suis ST1 uid167482
Streptococcus suis D9 uid162125
Streptococcus suis ST3 uid66327
Streptococcus suis A7 uid162111
Streptococcus suis BM407 uid59321
Streptococcus suis 05ZYH33 uid58663
Streptococcus suis 98HAH33 uid58665
Streptococcus suis GZ1 uid161937
Streptococcus suis JS14 uid162095
Streptococcus suis P1 7 uid32235
Streptococcus suis SC84 uid59323
Streptococcus suis SS12 uid162123
Streptococcus parasanguinis ATCC 15912 uid49313
Streptococcus parasanguinis FW213 uid163997
Streptococcus gordonii Challis substr CH1 uid57667
Streptococcus intermedius JTH08 uid168614
Streptococcus pseudopneumoniae IS7493 uid71153
Streptococcus mitis B6 uid46097
Streptococcus oralis Uo5 uid65449
Streptococcus pneumoniae JJA uid59121
Streptococcus pneumoniae CGSP14 uid59181
Streptococcus pneumoniae INV200 uid162035
Streptococcus pneumoniae 70585 uid59125
Streptococcus pneumoniae P1031 uid59123
Streptococcus pneumoniae ATCC 700669 uid59287
Streptococcus pneumoniae AP200 uid52453
Streptococcus pneumoniae INV104 uid162039
Streptococcus pneumoniae OXC141 uid162037
Streptococcus pneumoniae G54 uid59167
Streptococcus pneumoniae TIGR4 uid57857
Streptococcus pneumoniae D39 uid58581
Streptococcus pneumoniae Hungary19A 6 uid59117
Streptococcus pneumoniae R6 uid57859
Streptococcus pneumoniae 670 6B uid52533
Streptococcus pneumoniae ST556 uid162191
Streptococcus pneumoniae TCH8431 19A uid49735
Streptococcus pneumoniae Taiwan19F 14 uid59119
rpsD

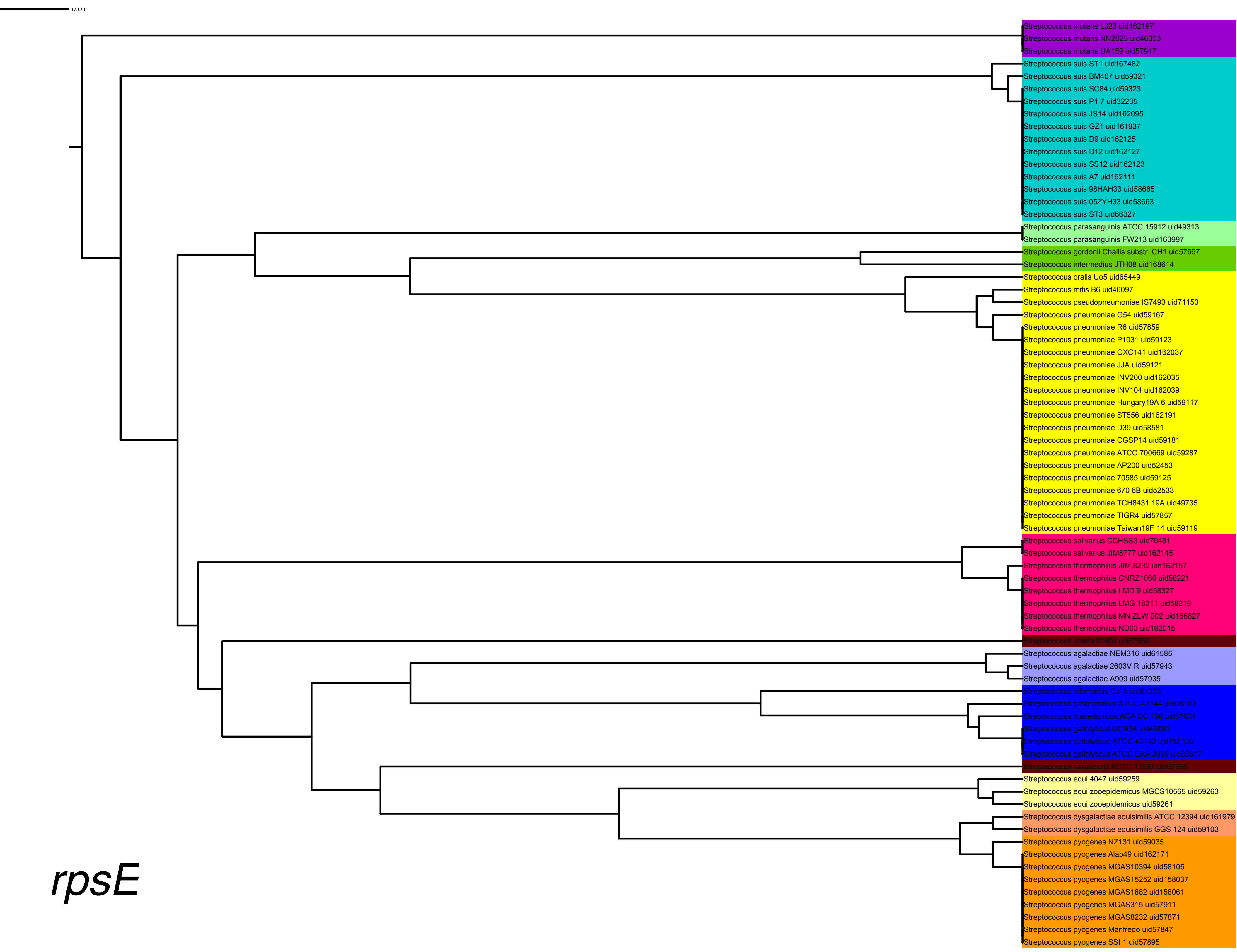
0.01
Streptococcus mutans LJ23 uid162197
Streptococcus mutans NN2025 uid46353
Streptococcus mutans UA159 uid57947
Streptococcus suis ST1 uid167482
Streptococcus suis BM407 uid59321
Streptococcus suis SC84 uid59323
Streptococcus suis P1 7 uid32235
Streptococcus suis JS14 uid162095
Streptococcus suis GZ1 uid161937
Streptococcus suis D9 uid162125
Streptococcus suis D12 uid162127
Streptococcus suis SS12 uid162123
Streptococcus suis A7 uid162111
Streptococcus suis 98HAH33 uid58665
Streptococcus suis 05ZYH33 uid58663
Streptococcus suis ST3 uid66327
Streptococcus parasanguinis ATCC 15912 uid49313
Streptococcus parasanguinis FW213 uid163997
Streptococcus gordonii Challis substr CH1 uid57667
Streptococcus intermedius JTH08 uid168614
Streptococcus oralis Uo5 uid65449
Streptococcus mitis B6 uid46097
Streptococcus pseudopneumoniae IS7493 uid71153
Streptococcus pneumoniae G54 uid59167
Streptococcus pneumoniae R6 uid57859
Streptococcus pneumoniae P1031 uid59123
Streptococcus pneumoniae OXC141 uid162037
Streptococcus pneumoniae JJA uid59121
Streptococcus pneumoniae INV200 uid162035
Streptococcus pneumoniae INV104 uid162039
Streptococcus pneumoniae Hungary19A 6 uid59117
Streptococcus pneumoniae ST556 uid162191
Streptococcus pneumoniae D39 uid58581
Streptococcus pneumoniae CGSP14 uid59181
Streptococcus pneumoniae ATCC 700669 uid59287
Streptococcus pneumoniae AP200 uid52453
Streptococcus pneumoniae 70585 uid59125
Streptococcus pneumoniae 670 6B uid52533
Streptococcus pneumoniae TCH8431 19A uid49735
Streptococcus pneumoniae TIGR4 uid57857
Streptococcus pneumoniae Taiwan19F 14 uid59119
Streptococcus salivarius CCHSS3 uid70481
Streptococcus salivarius JIM8777 uid162145
Streptococcus thermophilus JIM 8232 uid162157
Streptococcus thermophilus CNRZ1066 uid58221
Streptococcus thermophilus LMD 9 uid58327
Streptococcus thermophilus LMG 18311 uid58219
Streptococcus thermophilus MN ZLW 002 uid166827
Streptococcus thermophilus ND03 uid162015
Streptococcus uberis 0140J uid57959
Streptococcus agalactiae NEM316 uid61585
Streptococcus agalactiae 2603V R uid57943
Streptococcus agalactiae A909 uid57935
Streptococcus infantarius CJ18 uid87033
Streptococcus pasteurianus ATCC 43144 uid68019
Streptococcus macedonicus ACA DC 198 uid81631
Streptococcus gallolyticus UCN34 uid46061
Streptococcus gallolyticus ATCC 43143 uid162103
Streptococcus gallolyticus ATCC BAA 2069 uid63617
Streptococcus parauberis KCTC 11537 uid67355
Streptococcus equi 4047 uid59259
Streptococcus equi zooepidemicus MGCS10565 uid59263
Streptococcus equi zooepidemicus uid59261
Streptococcus dysgalactiae equisimilis ATCC 12394 uid161979
Streptococcus dysgalactiae equisimilis GGS 124 uid59103
Streptococcus pyogenes NZ131 uid59035
Streptococcus pyogenes Alab49 uid162171
Streptococcus pyogenes MGAS10394 uid58105
Streptococcus pyogenes MGAS15252 uid158037
Streptococcus pyogenes MGAS1882 uid158061
Streptococcus pyogenes MGAS315 uid57911
Streptococcus pyogenes MGAS8232 uid57871
Streptococcus pyogenes Manfredo uid57847
Streptococcus pyogenes SSI 1 uid57895
rpsE

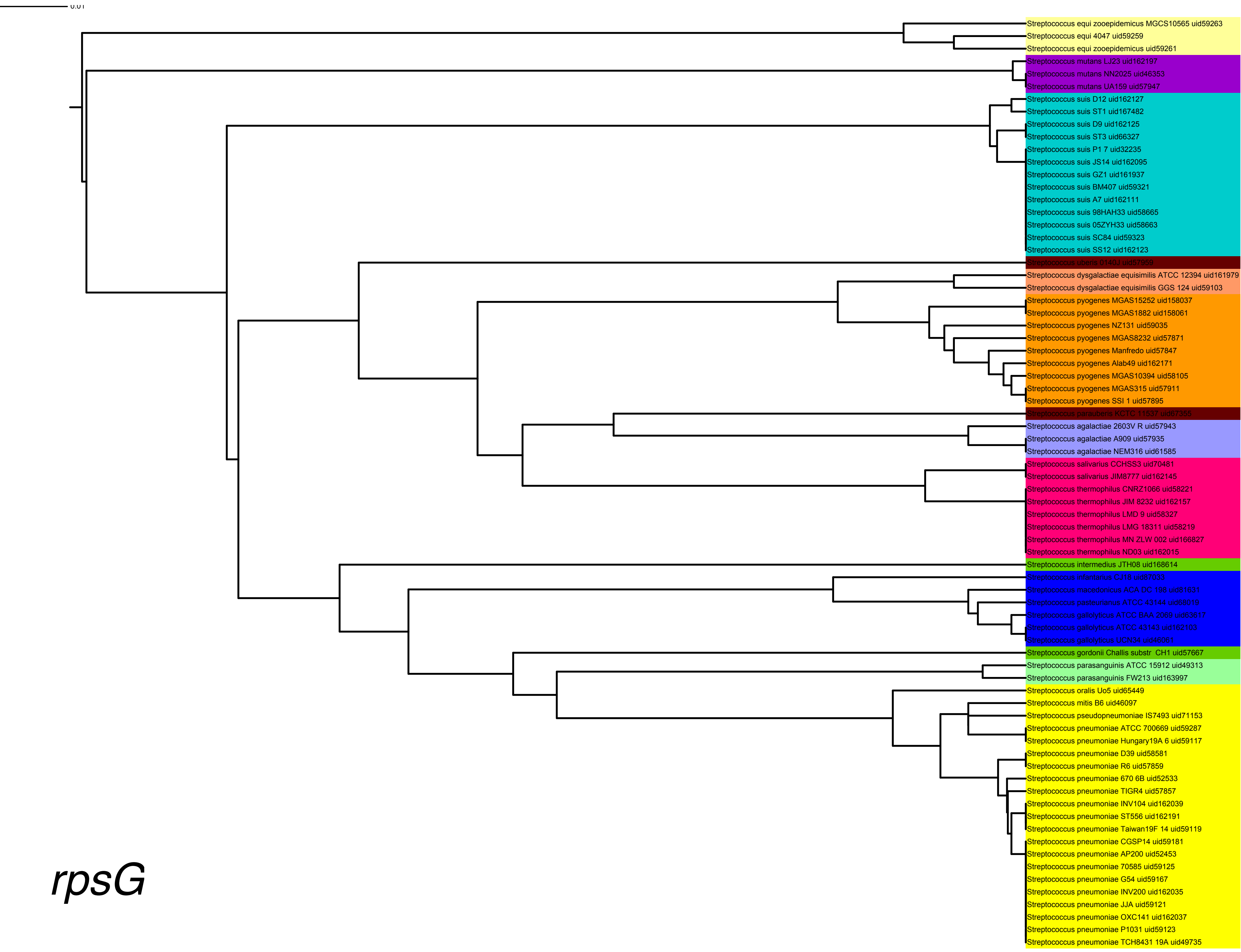
0.01
Streptococcus equi zooepidemicus MGCS10565 uid59263
Streptococcus equi 4047 uid59259
Streptococcus equi zooepidemicus uid59261
Streptococcus mutans LJ23 uid162197
Streptococcus mutans NN2025 uid46353
Streptococcus mutans UA159 uid57947
Streptococcus suis D12 uid162127
Streptococcus suis ST1 uid167482
Streptococcus suis D9 uid162125
Streptococcus suis ST3 uid66327
Streptococcus suis P1 7 uid32235
Streptococcus suis JS14 uid162095
Streptococcus suis GZ1 uid161937
Streptococcus suis BM407 uid59321
Streptococcus suis A7 uid162111
Streptococcus suis 98HAH33 uid58665
Streptococcus suis 05ZYH33 uid58663
Streptococcus suis SC84 uid59323
Streptococcus suis SS12 uid162123
Streptococcus uberis 0140J uid57959
Streptococcus dysgalactiae equisimilis ATCC 12394 uid161979
Streptococcus dysgalactiae equisimilis GGS 124 uid59103
Streptococcus pyogenes MGAS15252 uid158037
Streptococcus pyogenes MGAS1882 uid158061
Streptococcus pyogenes NZ131 uid59035
Streptococcus pyogenes MGAS8232 uid57871
Streptococcus pyogenes Manfredo uid57847
Streptococcus pyogenes Alab49 uid162171
Streptococcus pyogenes MGAS10394 uid58105
Streptococcus pyogenes MGAS315 uid57911
Streptococcus pyogenes SSI 1 uid57895
Streptococcus parauberis KCTC 11537 uid67355
Streptococcus agalactiae 2603V R uid57943
Streptococcus agalactiae A909 uid57935
Streptococcus agalactiae NEM316 uid61585
Streptococcus salivarius CCHSS3 uid70481
Streptococcus salivarius JIM8777 uid162145
Streptococcus thermophilus CNRZ1066 uid58221
Streptococcus thermophilus JIM 8232 uid162157
Streptococcus thermophilus LMD 9 uid58327
Streptococcus thermophilus LMG 18311 uid58219
Streptococcus thermophilus MN ZLW 002 uid166827
Streptococcus thermophilus ND03 uid162015
Streptococcus intermedius JTH08 uid168614
Streptococcus infantarius CJ18 uid87033
Streptococcus macedonicus ACA DC 198 uid81631
Streptococcus pasteurianus ATCC 43144 uid68019
Streptococcus gallolyticus ATCC BAA 2069 uid63617
Streptococcus gallolyticus ATCC 43143 uid162103
Streptococcus gallolyticus UCN34 uid46061
Streptococcus gordonii Challis substr CH1 uid57667
Streptococcus parasanguinis ATCC 15912 uid49313
Streptococcus parasanguinis FW213 uid163997
Streptococcus oralis Uo5 uid65449
Streptococcus mitis B6 uid46097
Streptococcus pseudopneumoniae IS7493 uid71153
Streptococcus pneumoniae ATCC 700669 uid59287
Streptococcus pneumoniae Hungary19A 6 uid59117
Streptococcus pneumoniae D39 uid58581
Streptococcus pneumoniae R6 uid57859
Streptococcus pneumoniae 670 6B uid52533
Streptococcus pneumoniae TIGR4 uid57857
Streptococcus pneumoniae INV104 uid162039
Streptococcus pneumoniae ST556 uid162191
Streptococcus pneumoniae Taiwan19F 14 uid59119
Streptococcus pneumoniae CGSP14 uid59181
Streptococcus pneumoniae AP200 uid52453
Streptococcus pneumoniae 70585 uid59125
Streptococcus pneumoniae G54 uid59167
Streptococcus pneumoniae INV200 uid162035
Streptococcus pneumoniae JJA uid59121
Streptococcus pneumoniae OXC141 uid162037
Streptococcus pneumoniae P1031 uid59123
Streptococcus pneumoniae TCH8431 19A uid49735
rpsG

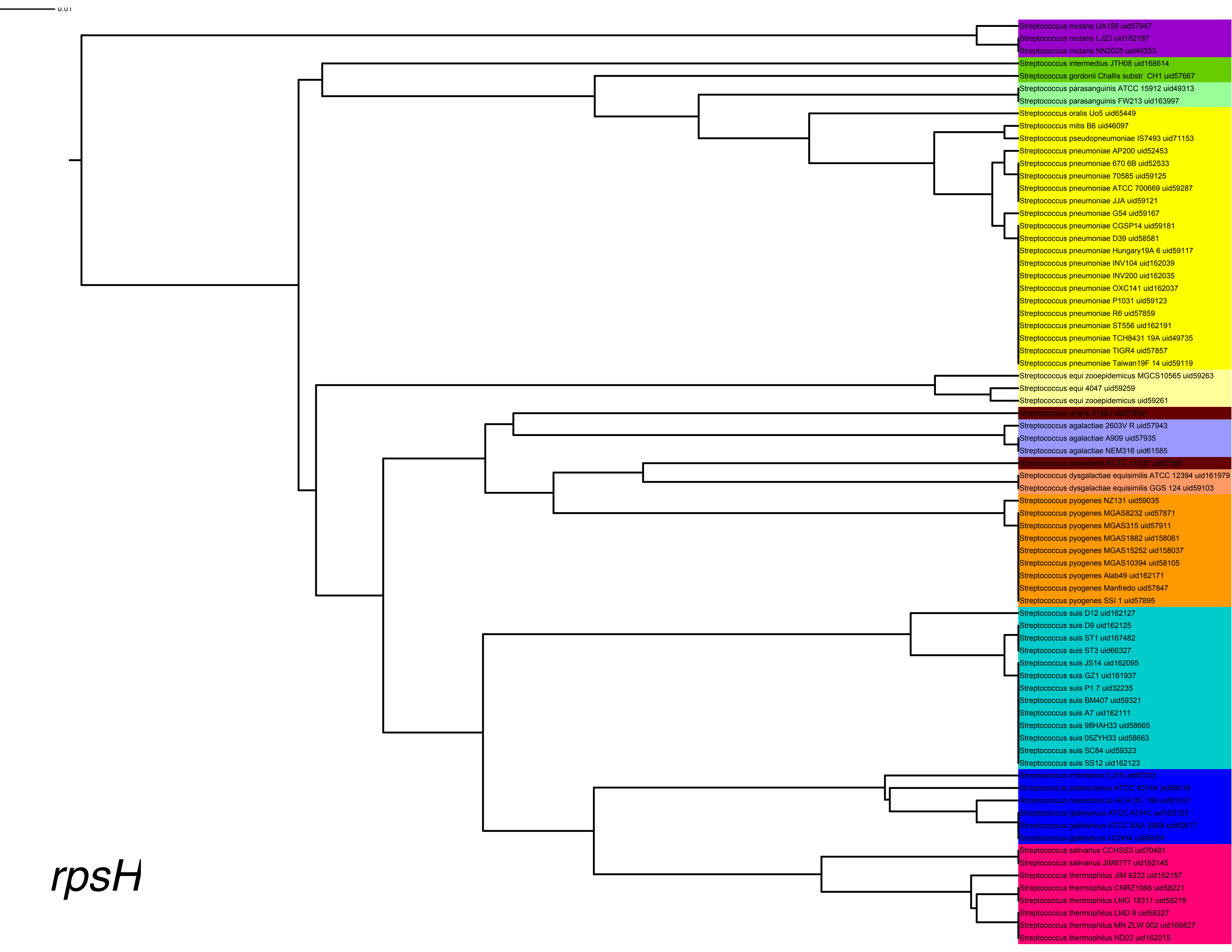
0.01
Streptococcus mutans UA159 uid57947
Streptococcus mutans LJ23 uid162197
Streptococcus mutans NN2025 uid46353
Streptococcus intermedius JTH08 uid168614
Streptococcus gordonii Challis substr CH1 uid57667
Streptococcus parasanguinis ATCC 15912 uid49313
Streptococcus parasanguinis FW213 uid163997
Streptococcus oralis Uo5 uid65449
Streptococcus mitis B6 uid46097
Streptococcus pseudopneumoniae IS7493 uid71153
Streptococcus pneumoniae AP200 uid52453
Streptococcus pneumoniae 670 6B uid52533
Streptococcus pneumoniae 70585 uid59125
Streptococcus pneumoniae ATCC 700669 uid59287
Streptococcus pneumoniae JJA uid59121
Streptococcus pneumoniae G54 uid59167
Streptococcus pneumoniae CGSP14 uid59181
Streptococcus pneumoniae D39 uid58581
Streptococcus pneumoniae Hungary19A 6 uid59117
Streptococcus pneumoniae INV104 uid162039
Streptococcus pneumoniae INV200 uid162035
Streptococcus pneumoniae OXC141 uid162037
Streptococcus pneumoniae P1031 uid59123
Streptococcus pneumoniae R6 uid57859
Streptococcus pneumoniae ST556 uid162191
Streptococcus pneumoniae TCH8431 19A uid49735
Streptococcus pneumoniae TIGR4 uid57857
Streptococcus pneumoniae Taiwan19F 14 uid59119
Streptococcus equi zooepidemicus MGCS10565 uid59263
Streptococcus equi 4047 uid59259
Streptococcus equi zooepidemicus uid59261
Streptococcus uberis 0140J uid57959
Streptococcus agalactiae 2603V R uid57943
Streptococcus agalactiae A909 uid57935
Streptococcus agalactiae NEM316 uid61585
Streptococcus parauberis KCTC 11537 uid67355
Streptococcus dysgalactiae equisimilis ATCC 12394 uid161979
Streptococcus dysgalactiae equisimilis GGS 124 uid59103
Streptococcus pyogenes NZ131 uid59035
Streptococcus pyogenes MGAS8232 uid57871
Streptococcus pyogenes MGAS315 uid57911
Streptococcus pyogenes MGAS1882 uid158061
Streptococcus pyogenes MGAS15252 uid158037
Streptococcus pyogenes MGAS10394 uid58105
Streptococcus pyogenes Alab49 uid162171
Streptococcus pyogenes Manfredo uid57847
Streptococcus pyogenes SSI 1 uid57895
Streptococcus suis D12 uid162127
Streptococcus suis D9 uid162125
Streptococcus suis ST1 uid167482
Streptococcus suis ST3 uid66327
Streptococcus suis JS14 uid162095
Streptococcus suis GZ1 uid161937
Streptococcus suis P1 7 uid32235
Streptococcus suis BM407 uid59321
Streptococcus suis A7 uid162111
Streptococcus suis 98HAH33 uid58665
Streptococcus suis 05ZYH33 uid58663
Streptococcus suis SC84 uid59323
Streptococcus suis SS12 uid162123
Streptococcus infantarius CJ18 uid87033
Streptococcus pasteurianus ATCC 43144 uid68019
Streptococcus macedonicus ACA DC 198 uid81631
Streptococcus gallolyticus ATCC 43143 uid162103
Streptococcus gallolyticus ATCC BAA 2069 uid63617
Streptococcus gallolyticus UCN34 uid46061
Streptococcus salivarius CCHSS3 uid70481
Streptococcus salivarius JIM8777 uid162145
Streptococcus thermophilus JIM 8232 uid162157
Streptococcus thermophilus CNRZ1066 uid58221
Streptococcus thermophilus LMG 18311 uid58219
Streptococcus thermophilus LMD 9 uid58327
Streptococcus thermophilus MN ZLW 002 uid166827
Streptococcus thermophilus ND03 uid162015
rpsH

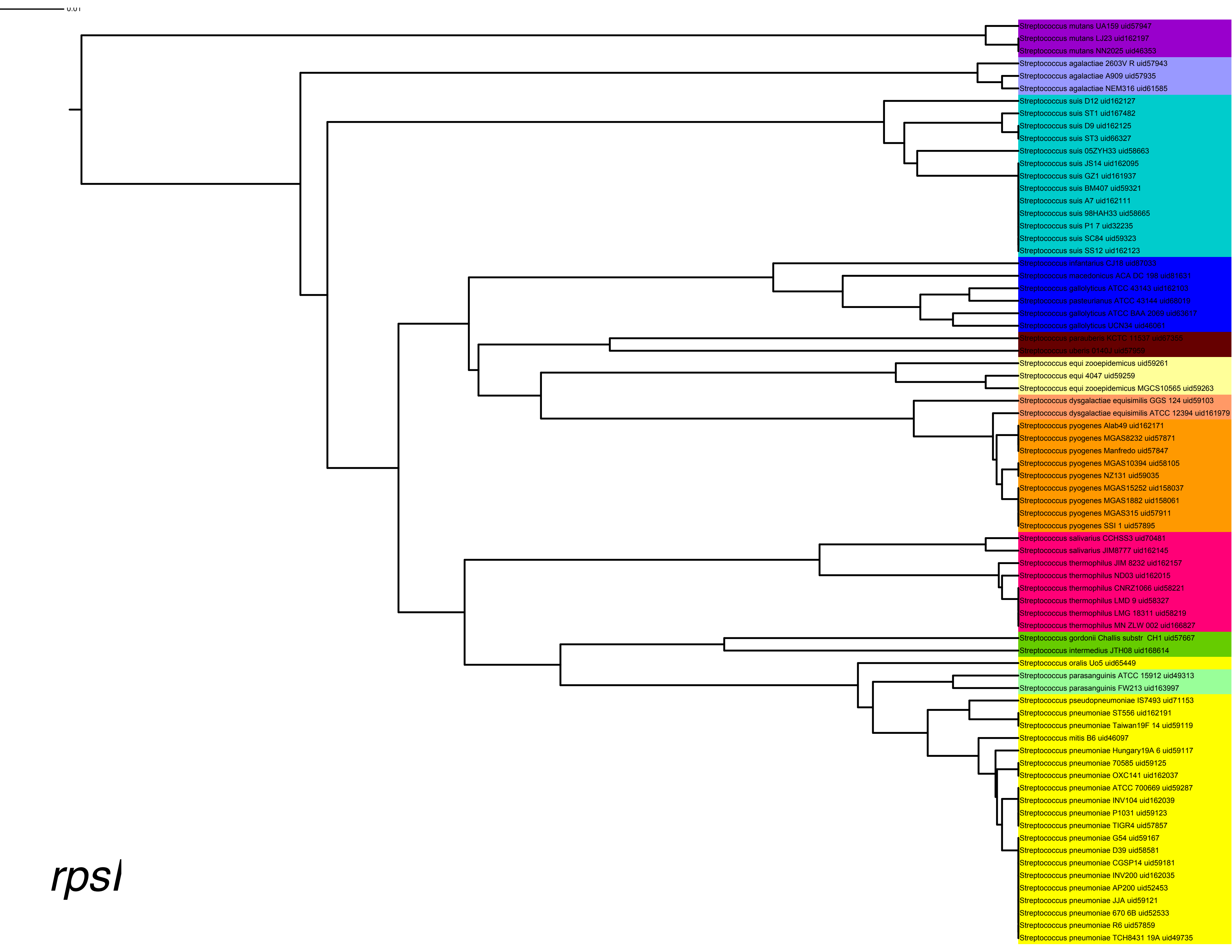
0.01
Streptococcus mutans UA159 uid57947
Streptococcus mutans LJ23 uid162197
Streptococcus mutans NN2025 uid46353
Streptococcus agalactiae 2603V R uid57943
Streptococcus agalactiae A909 uid57935
Streptococcus agalactiae NEM316 uid61585
Streptococcus suis D12 uid162127
Streptococcus suis ST1 uid167482
Streptococcus suis D9 uid162125
Streptococcus suis ST3 uid66327
Streptococcus suis 05ZYH33 uid58663
Streptococcus suis JS14 uid162095
Streptococcus suis GZ1 uid161937
Streptococcus suis BM407 uid59321
Streptococcus suis A7 uid162111
Streptococcus suis 98HAH33 uid58665
Streptococcus suis P1 7 uid32235
Streptococcus suis SC84 uid59323
Streptococcus suis SS12 uid162123
Streptococcus infantarius CJ18 uid87033
Streptococcus macedonicus ACA DC 198 uid81631
Streptococcus gallolyticus ATCC 43143 uid162103
Streptococcus pasteurianus ATCC 43144 uid68019
Streptococcus gallolyticus ATCC BAA 2069 uid63617
Streptococcus gallolyticus UCN34 uid46061
Streptococcus parauberis KCTC 11537 uid67355
Streptococcus uberis 0140J uid57959
Streptococcus equi zooepidemicus uid59261
Streptococcus equi 4047 uid59259
Streptococcus equi zooepidemicus MGCS10565 uid59263
Streptococcus dysgalactiae equisimilis GGS 124 uid59103
Streptococcus dysgalactiae equisimilis ATCC 12394 uid161979
Streptococcus pyogenes Alab49 uid162171
Streptococcus pyogenes MGAS8232 uid57871
Streptococcus pyogenes Manfredo uid57847
Streptococcus pyogenes MGAS10394 uid58105
Streptococcus pyogenes NZ131 uid59035
Streptococcus pyogenes MGAS15252 uid158037
Streptococcus pyogenes MGAS1882 uid158061
Streptococcus pyogenes MGAS315 uid57911
Streptococcus pyogenes SSI 1 uid57895
Streptococcus salivarius CCHSS3 uid70481
Streptococcus salivarius JIM8777 uid162145
Streptococcus thermophilus JIM 8232 uid162157
Streptococcus thermophilus ND03 uid162015
Streptococcus thermophilus CNRZ1066 uid58221
Streptococcus thermophilus LMD 9 uid58327
Streptococcus thermophilus LMG 18311 uid58219
Streptococcus thermophilus MN ZLW 002 uid166827
Streptococcus gordonii Challis substr CH1 uid57667
Streptococcus intermedius JTH08 uid168614
Streptococcus oralis Uo5 uid65449
Streptococcus parasanguinis ATCC 15912 uid49313
Streptococcus parasanguinis FW213 uid163997
Streptococcus pseudopneumoniae IS7493 uid71153
Streptococcus pneumoniae ST556 uid162191
Streptococcus pneumoniae Taiwan19F 14 uid59119
Streptococcus mitis B6 uid46097
Streptococcus pneumoniae Hungary19A 6 uid59117
Streptococcus pneumoniae 70585 uid59125
Streptococcus pneumoniae OXC141 uid162037
Streptococcus pneumoniae ATCC 700669 uid59287
Streptococcus pneumoniae INV104 uid162039
Streptococcus pneumoniae P1031 uid59123
Streptococcus pneumoniae TIGR4 uid57857
Streptococcus pneumoniae G54 uid59167
Streptococcus pneumoniae D39 uid58581
Streptococcus pneumoniae CGSP14 uid59181
Streptococcus pneumoniae INV200 uid162035
Streptococcus pneumoniae AP200 uid52453
Streptococcus pneumoniae JJA uid59121
Streptococcus pneumoniae 670 6B uid52533
Streptococcus pneumoniae R6 uid57859
Streptococcus pneumoniae TCH8431 19A uid49735
rpsI

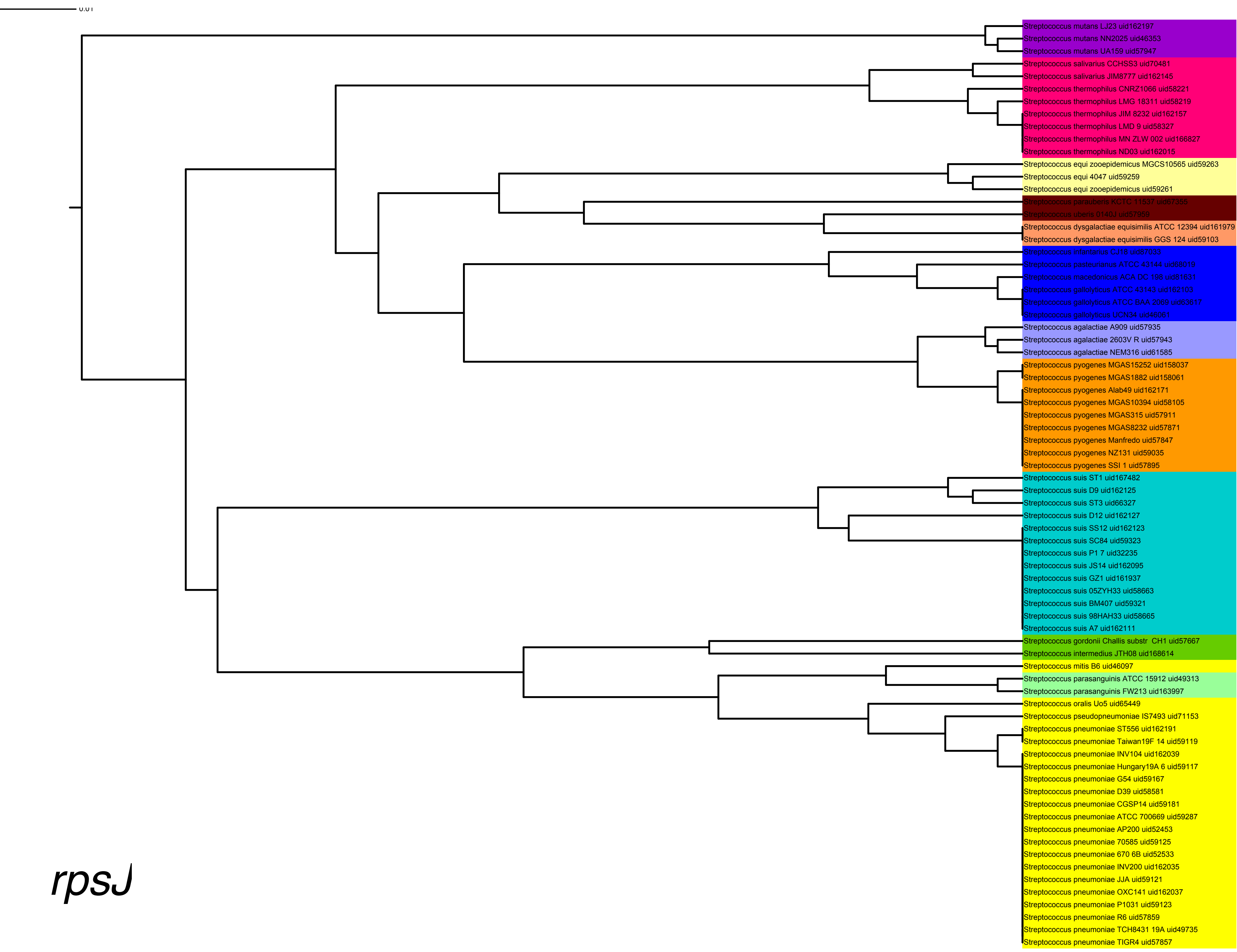
0.01
Streptococcus mutans LJ23 uid162197
Streptococcus mutans NN2025 uid46353
Streptococcus mutans UA159 uid57947
Streptococcus salivarius CCHSS3 uid70481
Streptococcus salivarius JIM8777 uid162145
Streptococcus thermophilus CNRZ1066 uid58221
Streptococcus thermophilus LMG 18311 uid58219
Streptococcus thermophilus JIM 8232 uid162157
Streptococcus thermophilus LMD 9 uid58327
Streptococcus thermophilus MN ZLW 002 uid166827
Streptococcus thermophilus ND03 uid162015
Streptococcus equi zooepidemicus MGCS10565 uid59263
Streptococcus equi 4047 uid59259
Streptococcus equi zooepidemicus uid59261
Streptococcus parauberis KCTC 11537 uid67355
Streptococcus uberis 0140J uid57959
Streptococcus dysgalactiae equisimilis ATCC 12394 uid161979
Streptococcus dysgalactiae equisimilis GGS 124 uid59103
Streptococcus infantarius CJ18 uid87033
Streptococcus pasteurianus ATCC 43144 uid68019
Streptococcus macedonicus ACA DC 198 uid81631
Streptococcus gallolyticus ATCC 43143 uid162103
Streptococcus gallolyticus ATCC BAA 2069 uid63617
Streptococcus gallolyticus UCN34 uid46061
Streptococcus agalactiae A909 uid57935
Streptococcus agalactiae 2603V R uid57943
Streptococcus agalactiae NEM316 uid61585
Streptococcus pyogenes MGAS15252 uid158037
Streptococcus pyogenes MGAS1882 uid158061
Streptococcus pyogenes Alab49 uid162171
Streptococcus pyogenes MGAS10394 uid58105
Streptococcus pyogenes MGAS315 uid57911
Streptococcus pyogenes MGAS8232 uid57871
Streptococcus pyogenes Manfredo uid57847
Streptococcus pyogenes NZ131 uid59035
Streptococcus pyogenes SSI 1 uid57895
Streptococcus suis ST1 uid167482
Streptococcus suis D9 uid162125
Streptococcus suis ST3 uid66327
Streptococcus suis D12 uid162127
Streptococcus suis SS12 uid162123
Streptococcus suis SC84 uid59323
Streptococcus suis P1 7 uid32235
Streptococcus suis JS14 uid162095
Streptococcus suis GZ1 uid161937
Streptococcus suis 05ZYH33 uid58663
Streptococcus suis BM407 uid59321
Streptococcus suis 98HAH33 uid58665
Streptococcus suis A7 uid162111
Streptococcus gordonii Challis substr CH1 uid57667
Streptococcus intermedius JTH08 uid168614
Streptococcus mitis B6 uid46097
Streptococcus parasanguinis ATCC 15912 uid49313
Streptococcus parasanguinis FW213 uid163997
Streptococcus oralis Uo5 uid65449
Streptococcus pseudopneumoniae IS7493 uid71153
Streptococcus pneumoniae ST556 uid162191
Streptococcus pneumoniae Taiwan19F 14 uid59119
Streptococcus pneumoniae INV104 uid162039
Streptococcus pneumoniae Hungary19A 6 uid59117
Streptococcus pneumoniae G54 uid59167
Streptococcus pneumoniae D39 uid58581
Streptococcus pneumoniae CGSP14 uid59181
Streptococcus pneumoniae ATCC 700669 uid59287
Streptococcus pneumoniae AP200 uid52453
Streptococcus pneumoniae 70585 uid59125
Streptococcus pneumoniae 670 6B uid52533
Streptococcus pneumoniae INV200 uid162035
Streptococcus pneumoniae JJA uid59121
Streptococcus pneumoniae OXC141 uid162037
Streptococcus pneumoniae P1031 uid59123
Streptococcus pneumoniae R6 uid57859
Streptococcus pneumoniae TCH8431 19A uid49735
Streptococcus pneumoniae TIGR4 uid57857
rpsJ

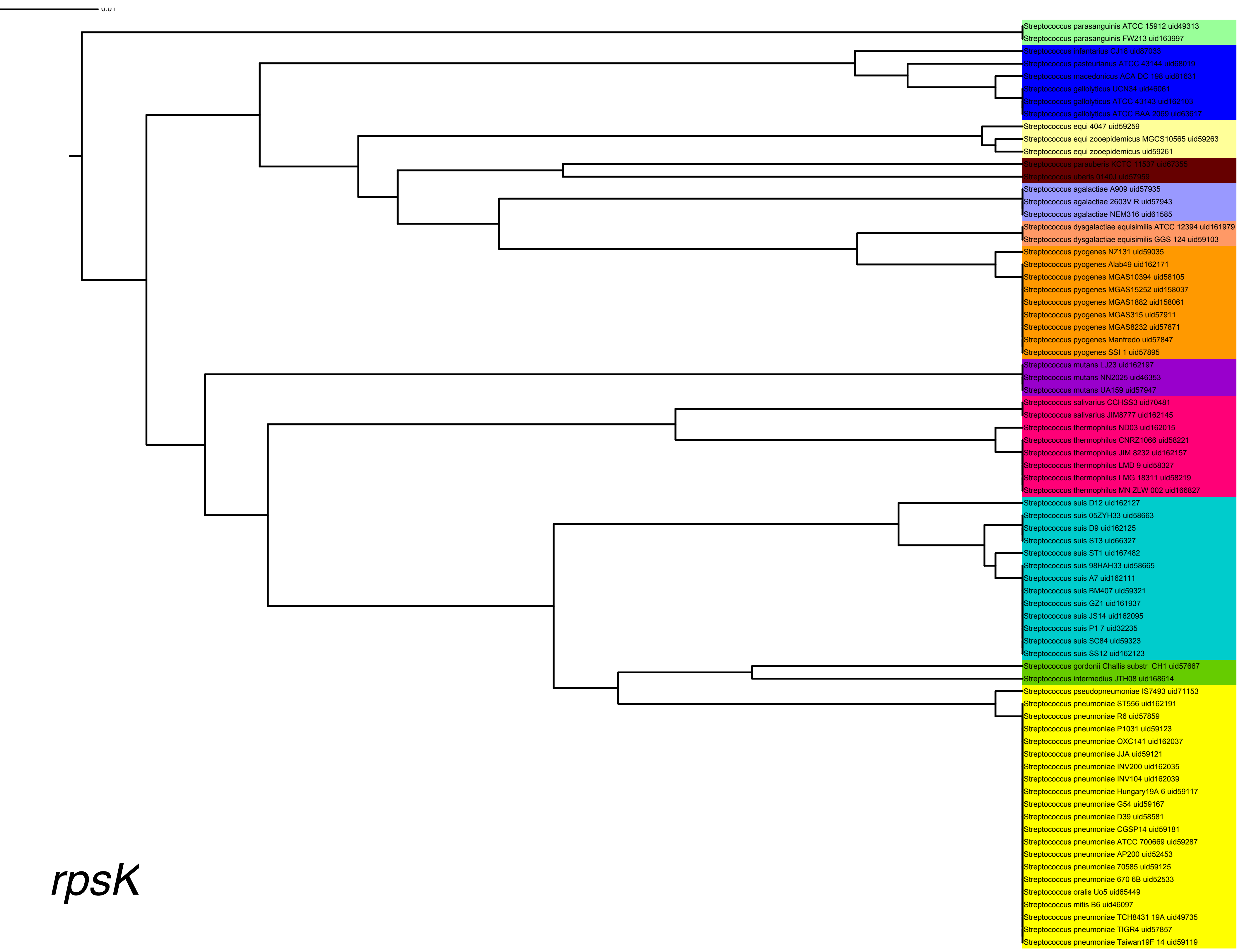

0.01
Streptococcus parasanguinis ATCC 15912 uid49313
Streptococcus parasanguinis FW213 uid163997
Streptococcus infantarius CJ18 uid87033
Streptococcus pasteurianus ATCC 43144 uid68019
Streptococcus macedonicus ACA DC 198 uid81631
Streptococcus gallolyticus UCN34 uid46061
Streptococcus gallolyticus ATCC 43143 uid162103
Streptococcus gallolyticus ATCC BAA 2069 uid63617
Streptococcus equi 4047 uid59259
Streptococcus equi zooepidemicus MGCS10565 uid59263
Streptococcus equi zooepidemicus uid59261
Streptococcus parauberis KCTC 11537 uid67355
Streptococcus uberis 0140J uid57959
Streptococcus agalactiae A909 uid57935
Streptococcus agalactiae 2603V R uid57943
Streptococcus agalactiae NEM316 uid61585
Streptococcus dysgalactiae equisimilis ATCC 12394 uid161979
Streptococcus dysgalactiae equisimilis GGS 124 uid59103
Streptococcus pyogenes NZ131 uid59035
Streptococcus pyogenes Alab49 uid162171
Streptococcus pyogenes MGAS10394 uid58105
Streptococcus pyogenes MGAS15252 uid158037
Streptococcus pyogenes MGAS1882 uid158061
Streptococcus pyogenes MGAS315 uid57911
Streptococcus pyogenes MGAS8232 uid57871
Streptococcus pyogenes Manfredo uid57847
Streptococcus pyogenes SSI 1 uid57895
Streptococcus mutans LJ23 uid162197
Streptococcus mutans NN2025 uid46353
Streptococcus mutans UA159 uid57947
Streptococcus salivarius CCHSS3 uid70481
Streptococcus salivarius JIM8777 uid162145
Streptococcus thermophilus ND03 uid162015
Streptococcus thermophilus CNRZ1066 uid58221
Streptococcus thermophilus JIM 8232 uid162157
Streptococcus thermophilus LMD 9 uid58327
Streptococcus thermophilus LMG 18311 uid58219
Streptococcus thermophilus MN ZLW 002 uid166827
Streptococcus suis D12 uid162127
Streptococcus suis 05ZYH33 uid58663
Streptococcus suis D9 uid162125
Streptococcus suis ST3 uid66327
Streptococcus suis ST1 uid167482
Streptococcus suis 98HAH33 uid58665
Streptococcus suis A7 uid162111
Streptococcus suis BM407 uid59321
Streptococcus suis GZ1 uid161937
Streptococcus suis JS14 uid162095
Streptococcus suis P1 7 uid32235
Streptococcus suis SC84 uid59323
Streptococcus suis SS12 uid162123
Streptococcus gordonii Challis substr CH1 uid57667
Streptococcus intermedius JTH08 uid168614
Streptococcus pseudopneumoniae IS7493 uid71153
Streptococcus pneumoniae ST556 uid162191
Streptococcus pneumoniae R6 uid57859
Streptococcus pneumoniae P1031 uid59123
Streptococcus pneumoniae OXC141 uid162037
Streptococcus pneumoniae JJA uid59121
Streptococcus pneumoniae INV200 uid162035
Streptococcus pneumoniae INV104 uid162039
Streptococcus pneumoniae Hungary19A 6 uid59117
Streptococcus pneumoniae G54 uid59167
Streptococcus pneumoniae D39 uid58581
Streptococcus pneumoniae CGSP14 uid59181
Streptococcus pneumoniae ATCC 700669 uid59287
Streptococcus pneumoniae AP200 uid52453
Streptococcus pneumoniae 70585 uid59125
Streptococcus pneumoniae 670 6B uid52533
Streptococcus oralis Uo5 uid65449
Streptococcus mitis B6 uid46097
Streptococcus pneumoniae TCH8431 19A uid49735
Streptococcus pneumoniae TIGR4 uid57857
Streptococcus pneumoniae Taiwan19F 14 uid59119
rpsK

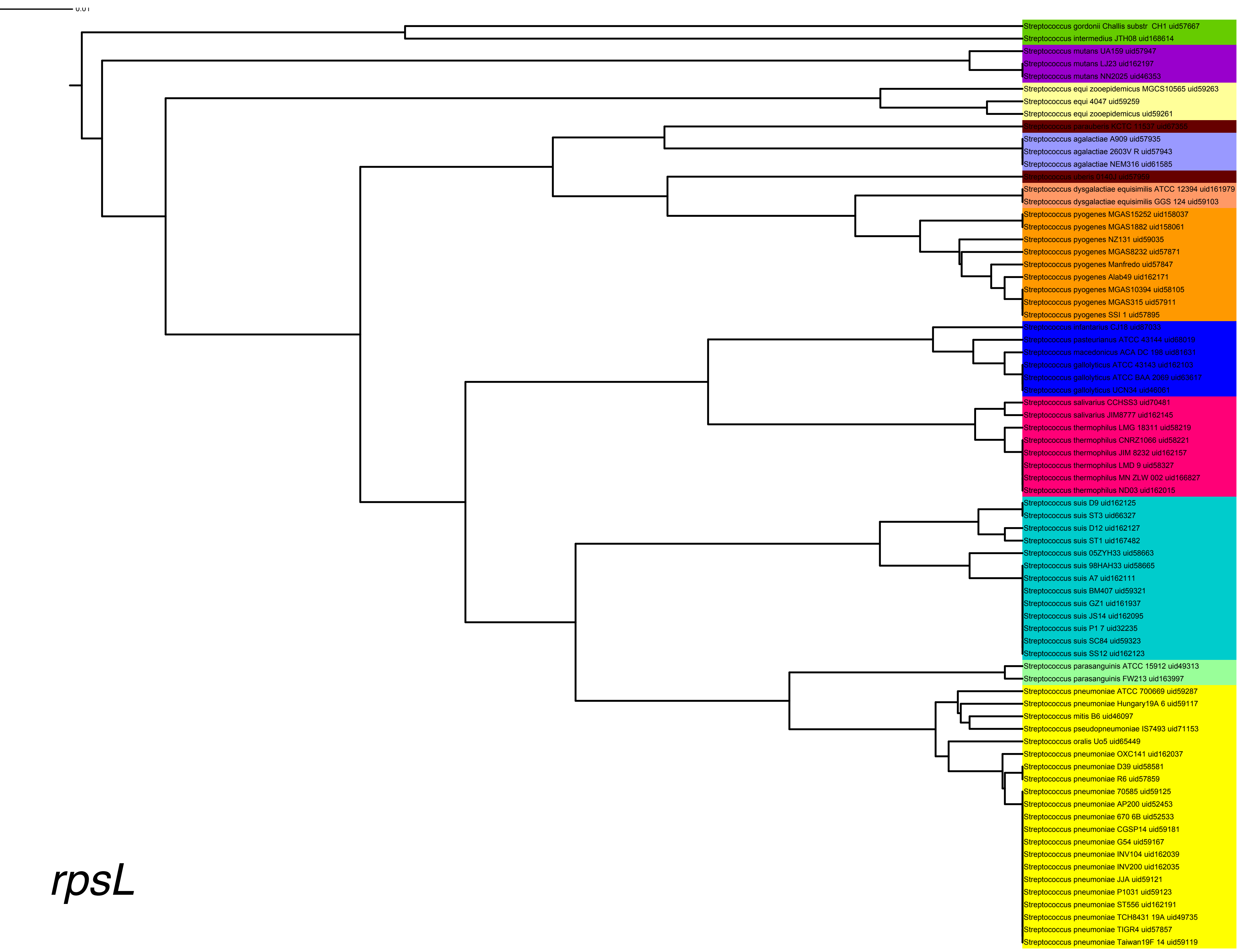
0.01
Streptococcus gordonii Challis substr CH1 uid57667
Streptococcus intermedius JTH08 uid168614
Streptococcus mutans UA159 uid57947
Streptococcus mutans LJ23 uid162197
Streptococcus mutans NN2025 uid46353
Streptococcus equi zooepidemicus MGCS10565 uid59263
Streptococcus equi 4047 uid59259
Streptococcus equi zooepidemicus uid59261
Streptococcus parauberis KCTC 11537 uid67355
Streptococcus agalactiae A909 uid57935
Streptococcus agalactiae 2603V R uid57943
Streptococcus agalactiae NEM316 uid61585
Streptococcus uberis 0140J uid57959
Streptococcus dysgalactiae equisimilis ATCC 12394 uid161979
Streptococcus dysgalactiae equisimilis GGS 124 uid59103
Streptococcus pyogenes MGAS15252 uid158037
Streptococcus pyogenes MGAS1882 uid158061
Streptococcus pyogenes NZ131 uid59035
Streptococcus pyogenes MGAS8232 uid57871
Streptococcus pyogenes Manfredo uid57847
Streptococcus pyogenes Alab49 uid162171
Streptococcus pyogenes MGAS10394 uid58105
Streptococcus pyogenes MGAS315 uid57911
Streptococcus pyogenes SSI 1 uid57895
Streptococcus infantarius CJ18 uid87033
Streptococcus pasteurianus ATCC 43144 uid68019
Streptococcus macedonicus ACA DC 198 uid81631
Streptococcus gallolyticus ATCC 43143 uid162103
Streptococcus gallolyticus ATCC BAA 2069 uid63617
Streptococcus gallolyticus UCN34 uid46061
Streptococcus salivarius CCHSS3 uid70481
Streptococcus salivarius JIM8777 uid162145
Streptococcus thermophilus LMG 18311 uid58219
Streptococcus thermophilus CNRZ1066 uid58221
Streptococcus thermophilus JIM 8232 uid162157
Streptococcus thermophilus LMD 9 uid58327
Streptococcus thermophilus MN ZLW 002 uid166827
Streptococcus thermophilus ND03 uid162015
Streptococcus suis D9 uid162125
Streptococcus suis ST3 uid66327
Streptococcus suis D12 uid162127
Streptococcus suis ST1 uid167482
Streptococcus suis 05ZYH33 uid58663
Streptococcus suis 98HAH33 uid58665
Streptococcus suis A7 uid162111
Streptococcus suis BM407 uid59321
Streptococcus suis GZ1 uid161937
Streptococcus suis JS14 uid162095
Streptococcus suis P1 7 uid32235
Streptococcus suis SC84 uid59323
Streptococcus suis SS12 uid162123
Streptococcus parasanguinis ATCC 15912 uid49313
Streptococcus parasanguinis FW213 uid163997
Streptococcus pneumoniae ATCC 700669 uid59287
Streptococcus pneumoniae Hungary19A 6 uid59117
Streptococcus mitis B6 uid46097
Streptococcus pseudopneumoniae IS7493 uid71153
Streptococcus oralis Uo5 uid65449
Streptococcus pneumoniae OXC141 uid162037
Streptococcus pneumoniae D39 uid58581
Streptococcus pneumoniae R6 uid57859
Streptococcus pneumoniae 70585 uid59125
Streptococcus pneumoniae AP200 uid52453
Streptococcus pneumoniae 670 6B uid52533
Streptococcus pneumoniae CGSP14 uid59181
Streptococcus pneumoniae G54 uid59167
Streptococcus pneumoniae INV104 uid162039
Streptococcus pneumoniae INV200 uid162035
Streptococcus pneumoniae JJA uid59121
Streptococcus pneumoniae P1031 uid59123
Streptococcus pneumoniae ST556 uid162191
Streptococcus pneumoniae TCH8431 19A uid49735
Streptococcus pneumoniae TIGR4 uid57857
Streptococcus pneumoniae Taiwan19F 14 uid59119
rpsL

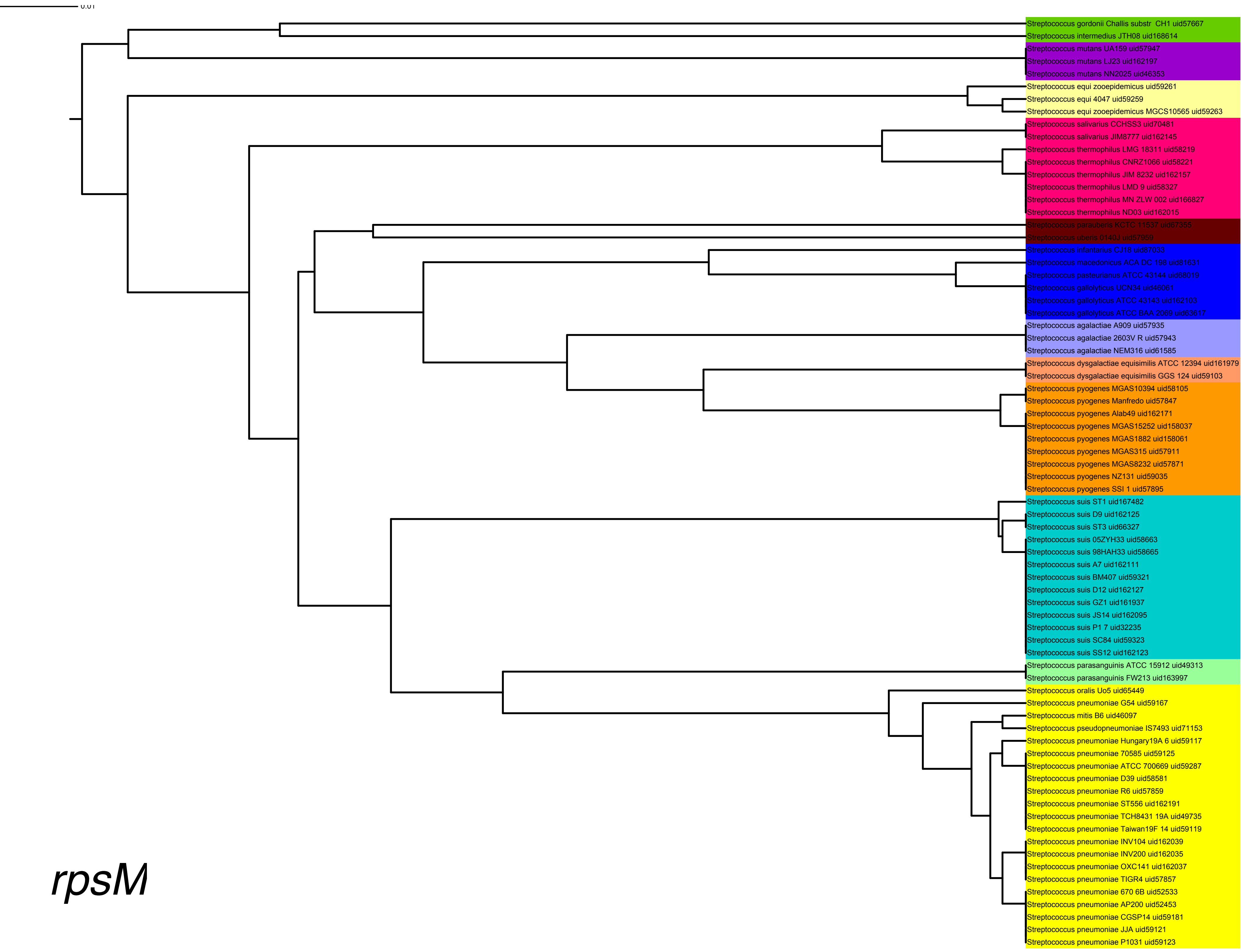
0.01
Streptococcus gordonii Challis substr CH1 uid57667
Streptococcus intermedius JTH08 uid168614
Streptococcus mutans UA159 uid57947
Streptococcus mutans LJ23 uid162197
Streptococcus mutans NN2025 uid46353
Streptococcus equi zooepidemicus uid59261
Streptococcus equi 4047 uid59259
Streptococcus equi zooepidemicus MGCS10565 uid59263
Streptococcus salivarius CCHSS3 uid70481
Streptococcus salivarius JIM8777 uid162145
Streptococcus thermophilus LMG 18311 uid58219
Streptococcus thermophilus CNRZ1066 uid58221
Streptococcus thermophilus JIM 8232 uid162157
Streptococcus thermophilus LMD 9 uid58327
Streptococcus thermophilus MN ZLW 002 uid166827
Streptococcus thermophilus ND03 uid162015
Streptococcus parauberis KCTC 11537 uid67355
Streptococcus uberis 0140J uid57959
Streptococcus infantarius CJ18 uid87033
Streptococcus macedonicus ACA DC 198 uid81631
Streptococcus pasteurianus ATCC 43144 uid68019
Streptococcus gallolyticus UCN34 uid46061
Streptococcus gallolyticus ATCC 43143 uid162103
Streptococcus gallolyticus ATCC BAA 2069 uid63617
Streptococcus agalactiae A909 uid57935
Streptococcus agalactiae 2603V R uid57943
Streptococcus agalactiae NEM316 uid61585
Streptococcus dysgalactiae equisimilis ATCC 12394 uid161979
Streptococcus dysgalactiae equisimilis GGS 124 uid59103
Streptococcus pyogenes MGAS10394 uid58105
Streptococcus pyogenes Manfredo uid57847
Streptococcus pyogenes Alab49 uid162171
Streptococcus pyogenes MGAS15252 uid158037
Streptococcus pyogenes MGAS1882 uid158061
Streptococcus pyogenes MGAS315 uid57911
Streptococcus pyogenes MGAS8232 uid57871
Streptococcus pyogenes NZ131 uid59035
Streptococcus pyogenes SSI 1 uid57895
Streptococcus suis ST1 uid167482
Streptococcus suis D9 uid162125
Streptococcus suis ST3 uid66327
Streptococcus suis 05ZYH33 uid58663
Streptococcus suis 98HAH33 uid58665
Streptococcus suis A7 uid162111
Streptococcus suis BM407 uid59321
Streptococcus suis D12 uid162127
Streptococcus suis GZ1 uid161937
Streptococcus suis JS14 uid162095
Streptococcus suis P1 7 uid32235
Streptococcus suis SC84 uid59323
Streptococcus suis SS12 uid162123
Streptococcus parasanguinis ATCC 15912 uid49313
Streptococcus parasanguinis FW213 uid163997
Streptococcus oralis Uo5 uid65449
Streptococcus pneumoniae G54 uid59167
Streptococcus mitis B6 uid46097
Streptococcus pseudopneumoniae IS7493 uid71153
Streptococcus pneumoniae Hungary19A 6 uid59117
Streptococcus pneumoniae 70585 uid59125
Streptococcus pneumoniae ATCC 700669 uid59287
Streptococcus pneumoniae D39 uid58581
Streptococcus pneumoniae R6 uid57859
Streptococcus pneumoniae ST556 uid162191
Streptococcus pneumoniae TCH8431 19A uid49735
Streptococcus pneumoniae Taiwan19F 14 uid59119
Streptococcus pneumoniae INV104 uid162039
Streptococcus pneumoniae INV200 uid162035
Streptococcus pneumoniae OXC141 uid162037
Streptococcus pneumoniae TIGR4 uid57857
Streptococcus pneumoniae 670 6B uid52533
Streptococcus pneumoniae AP200 uid52453
Streptococcus pneumoniae CGSP14 uid59181
Streptococcus pneumoniae JJA uid59121
Streptococcus pneumoniae P1031 uid59123
rpsM

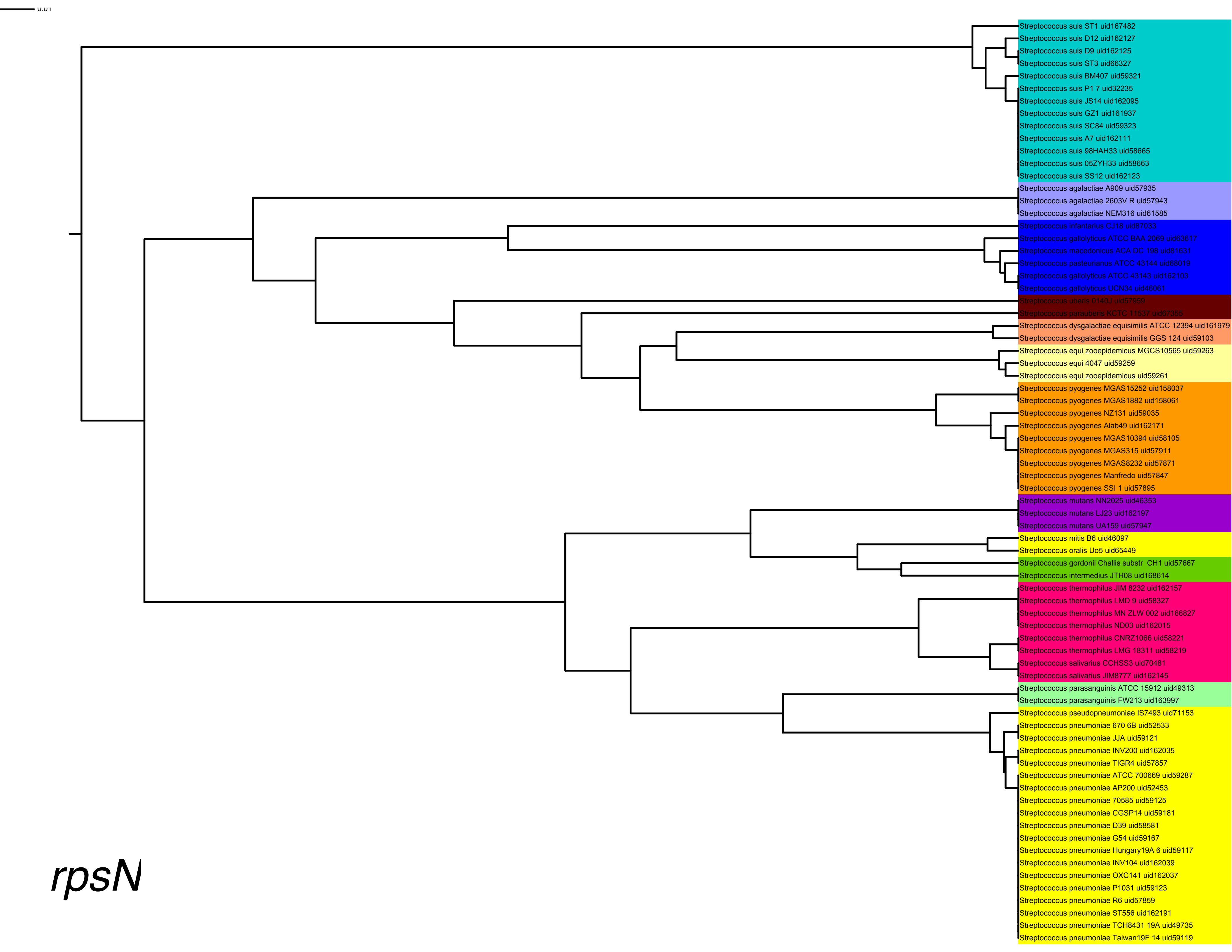
0.01
Streptococcus suis ST1 uid167482
Streptococcus suis D12 uid162127
Streptococcus suis D9 uid162125
Streptococcus suis ST3 uid66327
Streptococcus suis BM407 uid59321
Streptococcus suis P1 7 uid32235
Streptococcus suis JS14 uid162095
Streptococcus suis GZ1 uid161937
Streptococcus suis SC84 uid59323
Streptococcus suis A7 uid162111
Streptococcus suis 98HAH33 uid58665
Streptococcus suis 05ZYH33 uid58663
Streptococcus suis SS12 uid162123
Streptococcus agalactiae A909 uid57935
Streptococcus agalactiae 2603V R uid57943
Streptococcus agalactiae NEM316 uid61585
Streptococcus infantarius CJ18 uid87033
Streptococcus gallolyticus ATCC BAA 2069 uid63617
Streptococcus macedonicus ACA DC 198 uid81631
Streptococcus pasteurianus ATCC 43144 uid68019
Streptococcus gallolyticus ATCC 43143 uid162103
Streptococcus gallolyticus UCN34 uid46061
Streptococcus uberis 0140J uid57959
Streptococcus parauberis KCTC 11537 uid67355
Streptococcus dysgalactiae equisimilis ATCC 12394 uid161979
Streptococcus dysgalactiae equisimilis GGS 124 uid59103
Streptococcus equi zooepidemicus MGCS10565 uid59263
Streptococcus equi 4047 uid59259
Streptococcus equi zooepidemicus uid59261
Streptococcus pyogenes MGAS15252 uid158037
Streptococcus pyogenes MGAS1882 uid158061
Streptococcus pyogenes NZ131 uid59035
Streptococcus pyogenes Alab49 uid162171
Streptococcus pyogenes MGAS10394 uid58105
Streptococcus pyogenes MGAS315 uid57911
Streptococcus pyogenes MGAS8232 uid57871
Streptococcus pyogenes Manfredo uid57847
Streptococcus pyogenes SSI 1 uid57895
Streptococcus mutans NN2025 uid46353
Streptococcus mutans LJ23 uid162197
Streptococcus mutans UA159 uid57947
Streptococcus mitis B6 uid46097
Streptococcus oralis Uo5 uid65449
Streptococcus gordonii Challis substr CH1 uid57667
Streptococcus intermedius JTH08 uid168614
Streptococcus thermophilus JIM 8232 uid162157
Streptococcus thermophilus LMD 9 uid58327
Streptococcus thermophilus MN ZLW 002 uid166827
Streptococcus thermophilus ND03 uid162015
Streptococcus thermophilus CNRZ1066 uid58221
Streptococcus thermophilus LMG 18311 uid58219
Streptococcus salivarius CCHSS3 uid70481
Streptococcus salivarius JIM8777 uid162145
Streptococcus parasanguinis ATCC 15912 uid49313
Streptococcus parasanguinis FW213 uid163997
Streptococcus pseudopneumoniae IS7493 uid71153
Streptococcus pneumoniae 670 6B uid52533
Streptococcus pneumoniae JJA uid59121
Streptococcus pneumoniae INV200 uid162035
Streptococcus pneumoniae TIGR4 uid57857
Streptococcus pneumoniae ATCC 700669 uid59287
Streptococcus pneumoniae AP200 uid52453
Streptococcus pneumoniae 70585 uid59125
Streptococcus pneumoniae CGSP14 uid59181
Streptococcus pneumoniae D39 uid58581
Streptococcus pneumoniae G54 uid59167
Streptococcus pneumoniae Hungary19A 6 uid59117
Streptococcus pneumoniae INV104 uid162039
Streptococcus pneumoniae OXC141 uid162037
Streptococcus pneumoniae P1031 uid59123
Streptococcus pneumoniae R6 uid57859
Streptococcus pneumoniae ST556 uid162191
Streptococcus pneumoniae TCH8431 19A uid49735
Streptococcus pneumoniae Taiwan19F 14 uid59119
rpsN

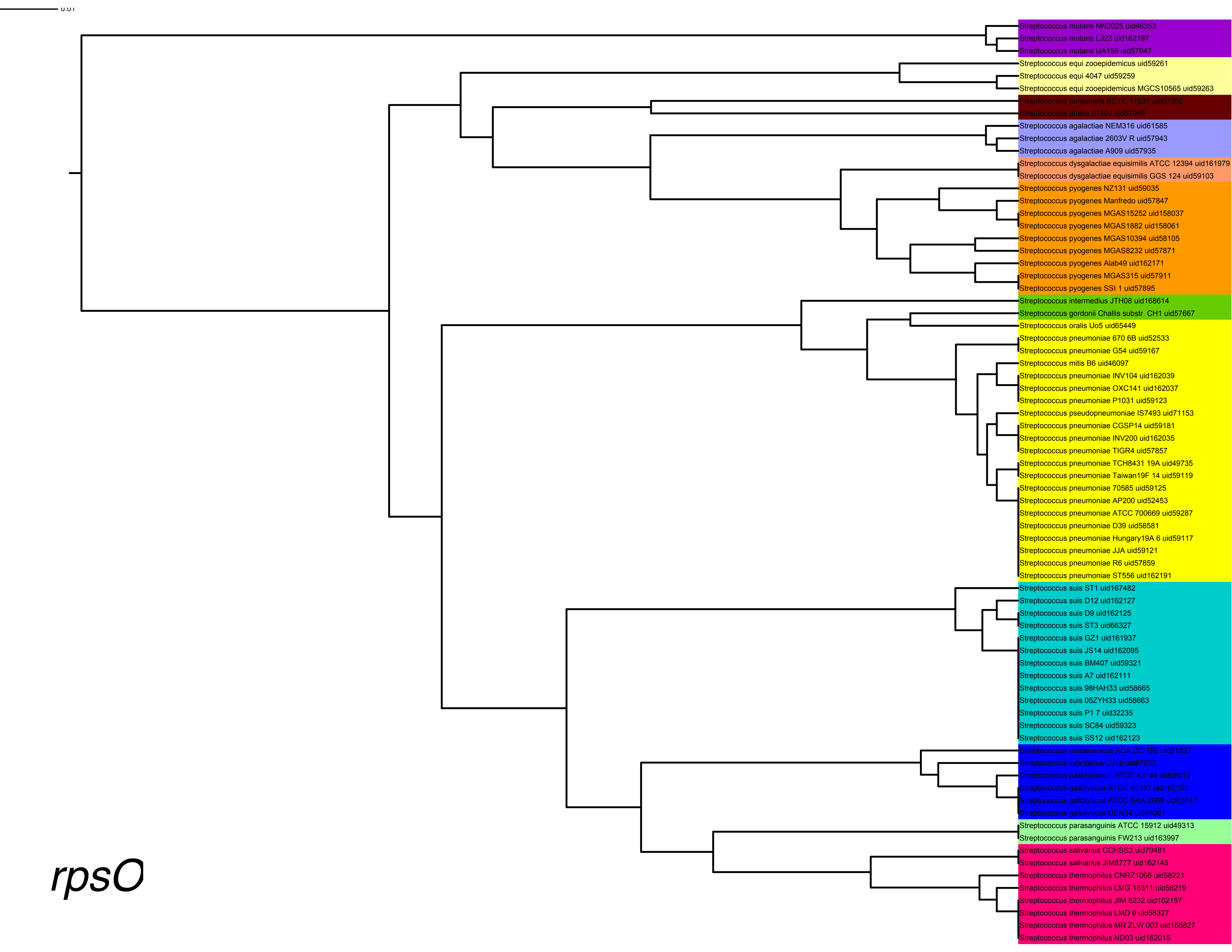
0.01
Streptococcus mutans NN2025 uid46353
Streptococcus mutans LJ23 uid162197
Streptococcus mutans UA159 uid57947
Streptococcus equi zooepidemicus uid59261
Streptococcus equi 4047 uid59259
Streptococcus equi zooepidemicus MGCS10565 uid59263
Streptococcus parauberis KCTC 11537 uid67355
Streptococcus uberis 0140J uid57959
Streptococcus agalactiae NEM316 uid61585
Streptococcus agalactiae 2603V R uid57943
Streptococcus agalactiae A909 uid57935
Streptococcus dysgalactiae equisimilis ATCC 12394 uid161979
Streptococcus dysgalactiae equisimilis GGS 124 uid59103
Streptococcus pyogenes NZ131 uid59035
Streptococcus pyogenes Manfredo uid57847
Streptococcus pyogenes MGAS15252 uid158037
Streptococcus pyogenes MGAS1882 uid158061
Streptococcus pyogenes MGAS10394 uid58105
Streptococcus pyogenes MGAS8232 uid57871
Streptococcus pyogenes Alab49 uid162171
Streptococcus pyogenes MGAS315 uid57911
Streptococcus pyogenes SSI 1 uid57895
Streptococcus intermedius JTH08 uid168614
Streptococcus gordonii Challis substr CH1 uid57667
Streptococcus oralis Uo5 uid65449
Streptococcus pneumoniae 670 6B uid52533
Streptococcus pneumoniae G54 uid59167
Streptococcus mitis B6 uid46097
Streptococcus pneumoniae INV104 uid162039
Streptococcus pneumoniae OXC141 uid162037
Streptococcus pneumoniae P1031 uid59123
Streptococcus pseudopneumoniae IS7493 uid71153
Streptococcus pneumoniae CGSP14 uid59181
Streptococcus pneumoniae INV200 uid162035
Streptococcus pneumoniae TIGR4 uid57857
Streptococcus pneumoniae TCH8431 19A uid49735
Streptococcus pneumoniae Taiwan19F 14 uid59119
Streptococcus pneumoniae 70585 uid59125
Streptococcus pneumoniae AP200 uid52453
Streptococcus pneumoniae ATCC 700669 uid59287
Streptococcus pneumoniae D39 uid58581
Streptococcus pneumoniae Hungary19A 6 uid59117
Streptococcus pneumoniae JJA uid59121
Streptococcus pneumoniae R6 uid57859
Streptococcus pneumoniae ST556 uid162191
Streptococcus suis ST1 uid167482
Streptococcus suis D12 uid162127
Streptococcus suis D9 uid162125
Streptococcus suis ST3 uid66327
Streptococcus suis GZ1 uid161937
Streptococcus suis JS14 uid162095
Streptococcus suis BM407 uid59321
Streptococcus suis A7 uid162111
Streptococcus suis 98HAH33 uid58665
Streptococcus suis 05ZYH33 uid58663
Streptococcus suis P1 7 uid32235
Streptococcus suis SC84 uid59323
Streptococcus suis SS12 uid162123
Streptococcus macedonicus ACA DC 198 uid81631
Streptococcus infantarius CJ18 uid87033
Streptococcus pasteurianus ATCC 43144 uid68019
Streptococcus gallolyticus ATCC 43143 uid162103
Streptococcus gallolyticus ATCC BAA 2069 uid63617
Streptococcus gallolyticus UCN34 uid46061
Streptococcus parasanguinis ATCC 15912 uid49313
Streptococcus parasanguinis FW213 uid163997
Streptococcus salivarius CCHSS3 uid70481
Streptococcus salivarius JIM8777 uid162145
Streptococcus thermophilus CNRZ1066 uid58221
Streptococcus thermophilus LMG 18311 uid58219
Streptococcus thermophilus JIM 8232 uid162157
Streptococcus thermophilus LMD 9 uid58327
Streptococcus thermophilus MN ZLW 002 uid166827
Streptococcus thermophilus ND03 uid162015
rpsO

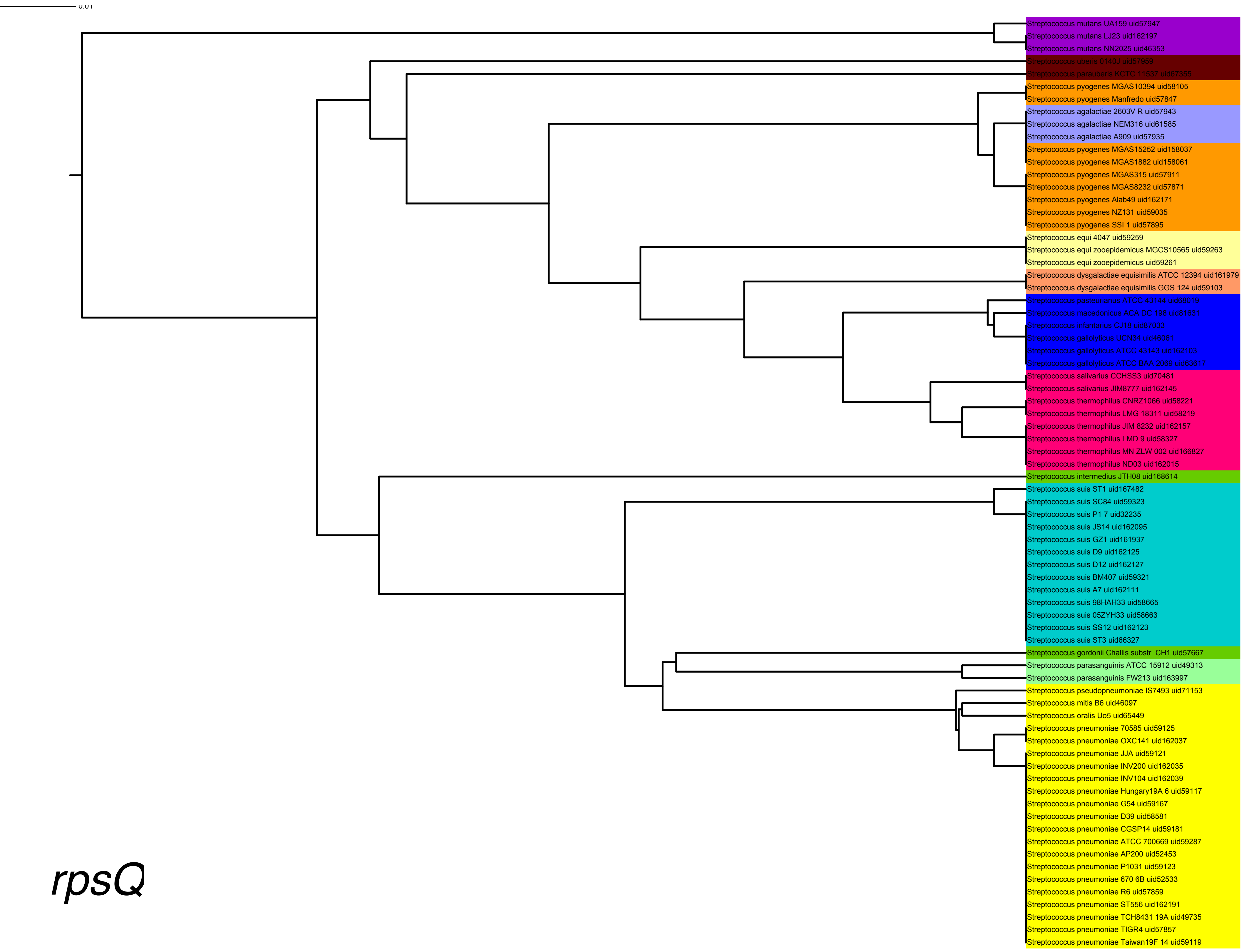
0.01
Streptococcus mutans UA159 uid57947
Streptococcus mutans LJ23 uid162197
Streptococcus mutans NN2025 uid46353
Streptococcus uberis 0140J uid57959
Streptococcus parauberis KCTC 11537 uid67355
Streptococcus pyogenes MGAS10394 uid58105
Streptococcus pyogenes Manfredo uid57847
Streptococcus agalactiae 2603V R uid57943
Streptococcus agalactiae NEM316 uid61585
Streptococcus agalactiae A909 uid57935
Streptococcus pyogenes MGAS15252 uid158037
Streptococcus pyogenes MGAS1882 uid158061
Streptococcus pyogenes MGAS315 uid57911
Streptococcus pyogenes MGAS8232 uid57871
Streptococcus pyogenes Alab49 uid162171
Streptococcus pyogenes NZ131 uid59035
Streptococcus pyogenes SSI 1 uid57895
Streptococcus equi 4047 uid59259
Streptococcus equi zooepidemicus MGCS10565 uid59263
Streptococcus equi zooepidemicus uid59261
Streptococcus dysgalactiae equisimilis ATCC 12394 uid161979
Streptococcus dysgalactiae equisimilis GGS 124 uid59103
Streptococcus pasteurianus ATCC 43144 uid68019
Streptococcus macedonicus ACA DC 198 uid81631
Streptococcus infantarius CJ18 uid87033
Streptococcus gallolyticus UCN34 uid46061
Streptococcus gallolyticus ATCC 43143 uid162103
Streptococcus gallolyticus ATCC BAA 2069 uid63617
Streptococcus salivarius CCHSS3 uid70481
Streptococcus salivarius JIM8777 uid162145
Streptococcus thermophilus CNRZ1066 uid58221
Streptococcus thermophilus LMG 18311 uid58219
Streptococcus thermophilus JIM 8232 uid162157
Streptococcus thermophilus LMD 9 uid58327
Streptococcus thermophilus MN ZLW 002 uid166827
Streptococcus thermophilus ND03 uid162015
Streptococcus intermedius JTH08 uid168614
Streptococcus suis ST1 uid167482
Streptococcus suis SC84 uid59323
Streptococcus suis P1 7 uid32235
Streptococcus suis JS14 uid162095
Streptococcus suis GZ1 uid161937
Streptococcus suis D9 uid162125
Streptococcus suis D12 uid162127
Streptococcus suis BM407 uid59321
Streptococcus suis A7 uid162111
Streptococcus suis 98HAH33 uid58665
Streptococcus suis 05ZYH33 uid58663
Streptococcus suis SS12 uid162123
Streptococcus suis ST3 uid66327
Streptococcus gordonii Challis substr CH1 uid57667
Streptococcus parasanguinis ATCC 15912 uid49313
Streptococcus parasanguinis FW213 uid163997
Streptococcus pseudopneumoniae IS7493 uid71153
Streptococcus mitis B6 uid46097
Streptococcus oralis Uo5 uid65449
Streptococcus pneumoniae 70585 uid59125
Streptococcus pneumoniae OXC141 uid162037
Streptococcus pneumoniae JJA uid59121
Streptococcus pneumoniae INV200 uid162035
Streptococcus pneumoniae INV104 uid162039
Streptococcus pneumoniae Hungary19A 6 uid59117
Streptococcus pneumoniae G54 uid59167
Streptococcus pneumoniae D39 uid58581
Streptococcus pneumoniae CGSP14 uid59181
Streptococcus pneumoniae ATCC 700669 uid59287
Streptococcus pneumoniae AP200 uid52453
Streptococcus pneumoniae P1031 uid59123
Streptococcus pneumoniae 670 6B uid52533
Streptococcus pneumoniae R6 uid57859
Streptococcus pneumoniae ST556 uid162191
Streptococcus pneumoniae TCH8431 19A uid49735
Streptococcus pneumoniae TIGR4 uid57857
Streptococcus pneumoniae Taiwan19F 14 uid59119
rpsQ

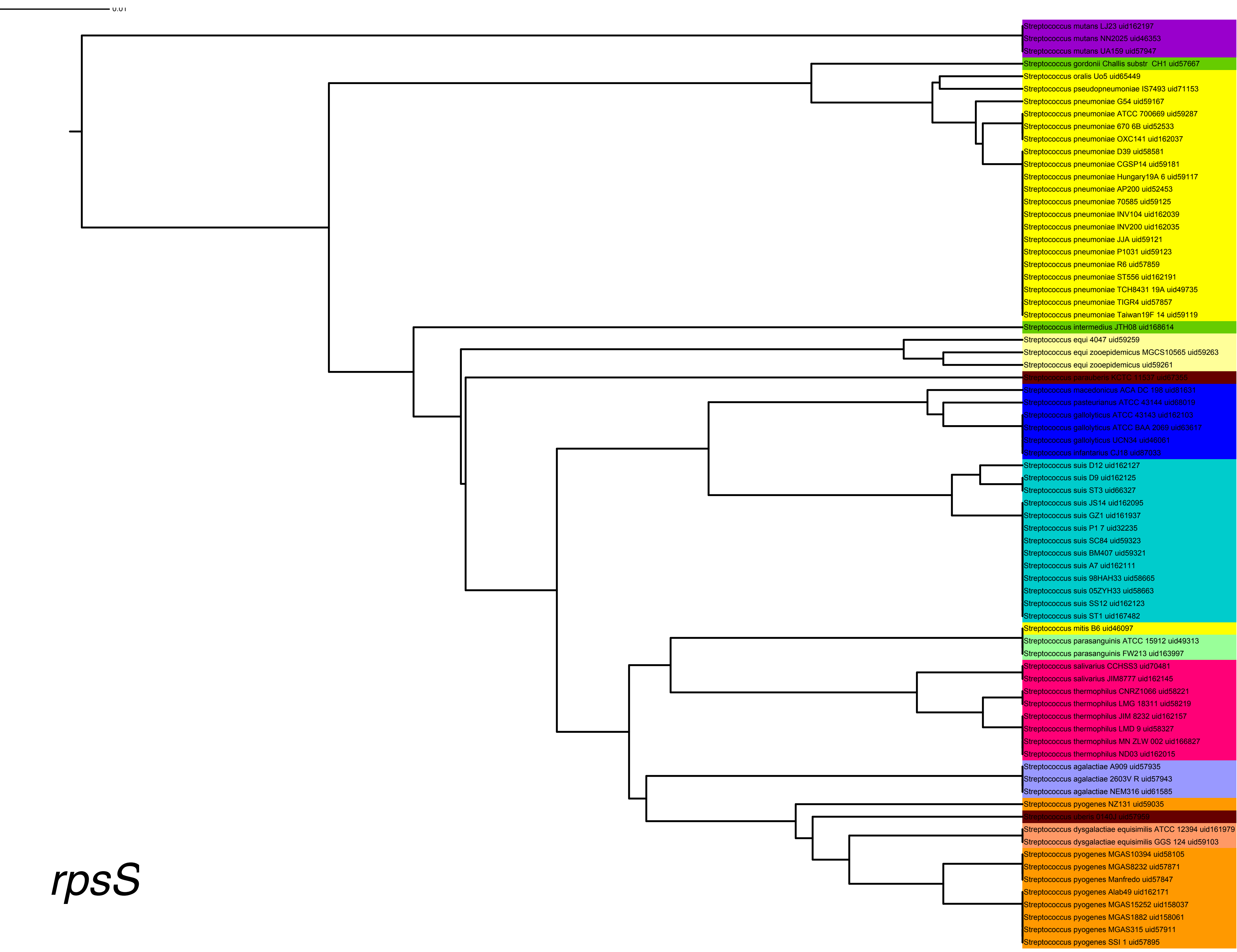

0.01
Streptococcus mutans LJ23 uid162197
Streptococcus mutans NN2025 uid46353
Streptococcus mutans UA159 uid57947
Streptococcus gordonii Challis substr CH1 uid57667
Streptococcus oralis Uo5 uid65449
Streptococcus pseudopneumoniae IS7493 uid71153
Streptococcus pneumoniae G54 uid59167
Streptococcus pneumoniae ATCC 700669 uid59287
Streptococcus pneumoniae 670 6B uid52533
Streptococcus pneumoniae OXC141 uid162037
Streptococcus pneumoniae D39 uid58581
Streptococcus pneumoniae CGSP14 uid59181
Streptococcus pneumoniae Hungary19A 6 uid59117
Streptococcus pneumoniae AP200 uid52453
Streptococcus pneumoniae 70585 uid59125
Streptococcus pneumoniae INV104 uid162039
Streptococcus pneumoniae INV200 uid162035
Streptococcus pneumoniae JJA uid59121
Streptococcus pneumoniae P1031 uid59123
Streptococcus pneumoniae R6 uid57859
Streptococcus pneumoniae ST556 uid162191
Streptococcus pneumoniae TCH8431 19A uid49735
Streptococcus pneumoniae TIGR4 uid57857
Streptococcus pneumoniae Taiwan19F 14 uid59119
Streptococcus intermedius JTH08 uid168614
Streptococcus equi 4047 uid59259
Streptococcus equi zooepidemicus MGCS10565 uid59263
Streptococcus equi zooepidemicus uid59261
Streptococcus parauberis KCTC 11537 uid67355
Streptococcus macedonicus ACA DC 198 uid81631
Streptococcus pasteurianus ATCC 43144 uid68019
Streptococcus gallolyticus ATCC 43143 uid162103
Streptococcus gallolyticus ATCC BAA 2069 uid63617
Streptococcus gallolyticus UCN34 uid46061
Streptococcus infantarius CJ18 uid87033
Streptococcus suis D12 uid162127
Streptococcus suis D9 uid162125
Streptococcus suis ST3 uid66327
Streptococcus suis JS14 uid162095
Streptococcus suis GZ1 uid161937
Streptococcus suis P1 7 uid32235
Streptococcus suis SC84 uid59323
Streptococcus suis BM407 uid59321
Streptococcus suis A7 uid162111
Streptococcus suis 98HAH33 uid58665
Streptococcus suis 05ZYH33 uid58663
Streptococcus suis SS12 uid162123
Streptococcus suis ST1 uid167482
Streptococcus mitis B6 uid46097
Streptococcus parasanguinis ATCC 15912 uid49313
Streptococcus parasanguinis FW213 uid163997
Streptococcus salivarius CCHSS3 uid70481
Streptococcus salivarius JIM8777 uid162145
Streptococcus thermophilus CNRZ1066 uid58221
Streptococcus thermophilus LMG 18311 uid58219
Streptococcus thermophilus JIM 8232 uid162157
Streptococcus thermophilus LMD 9 uid58327
Streptococcus thermophilus MN ZLW 002 uid166827
Streptococcus thermophilus ND03 uid162015
Streptococcus agalactiae A909 uid57935
Streptococcus agalactiae 2603V R uid57943
Streptococcus agalactiae NEM316 uid61585
Streptococcus pyogenes NZ131 uid59035
Streptococcus uberis 0140J uid57959
Streptococcus dysgalactiae equisimilis ATCC 12394 uid161979
Streptococcus dysgalactiae equisimilis GGS 124 uid59103
Streptococcus pyogenes MGAS10394 uid58105
Streptococcus pyogenes MGAS8232 uid57871
Streptococcus pyogenes Manfredo uid57847
Streptococcus pyogenes Alab49 uid162171
Streptococcus pyogenes MGAS15252 uid158037
Streptococcus pyogenes MGAS1882 uid158061
Streptococcus pyogenes MGAS315 uid57911
Streptococcus pyogenes SSI 1 uid57895
rpsS

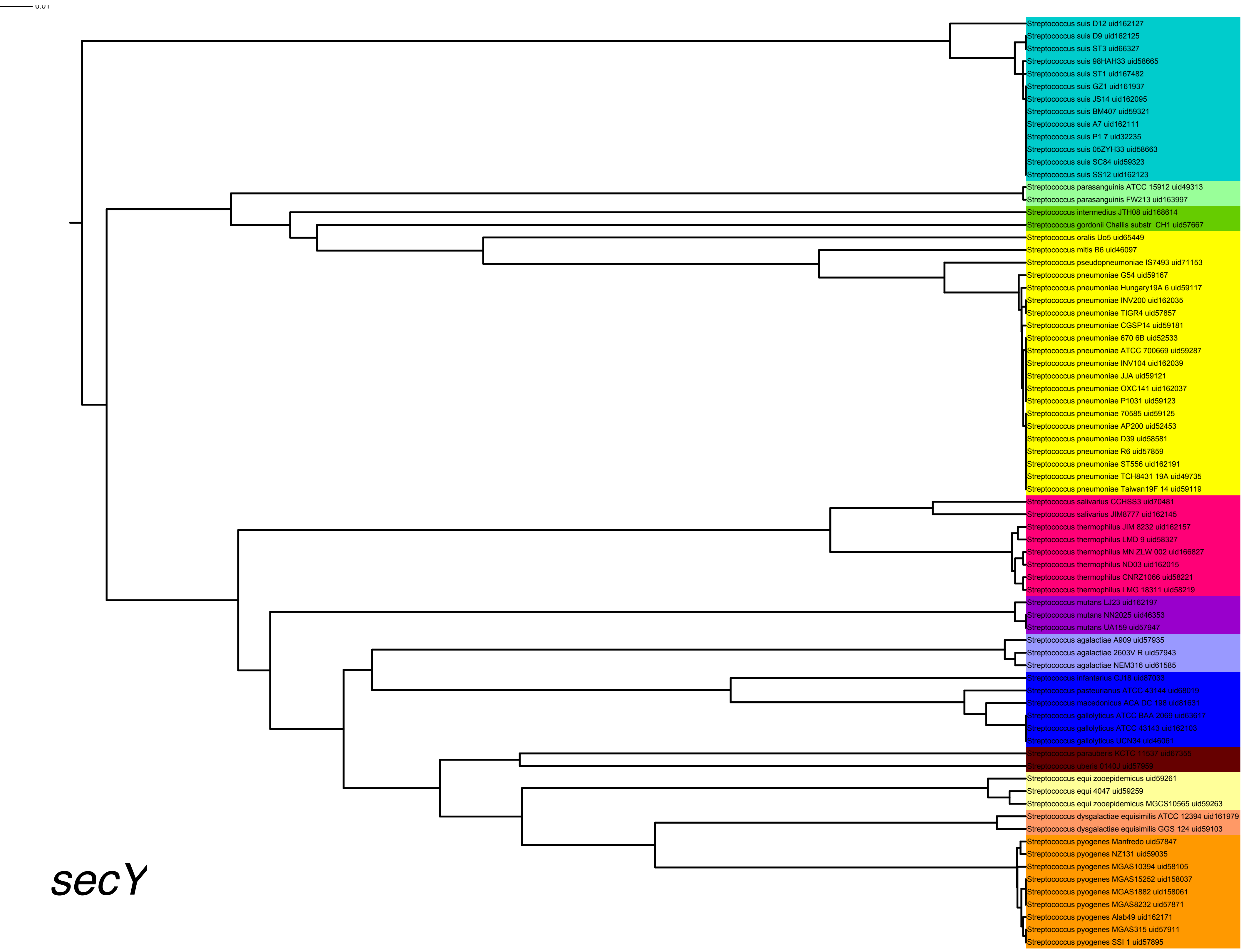
0.01
Streptococcus suis D12 uid162127
Streptococcus suis D9 uid162125
Streptococcus suis ST3 uid66327
Streptococcus suis 98HAH33 uid58665
Streptococcus suis ST1 uid167482
Streptococcus suis GZ1 uid161937
Streptococcus suis JS14 uid162095
Streptococcus suis BM407 uid59321
Streptococcus suis A7 uid162111
Streptococcus suis P1 7 uid32235
Streptococcus suis 05ZYH33 uid58663
Streptococcus suis SC84 uid59323
Streptococcus suis SS12 uid162123
Streptococcus parasanguinis ATCC 15912 uid49313
Streptococcus parasanguinis FW213 uid163997
Streptococcus intermedius JTH08 uid168614
Streptococcus gordonii Challis substr CH1 uid57667
Streptococcus oralis Uo5 uid65449
Streptococcus mitis B6 uid46097
Streptococcus pseudopneumoniae IS7493 uid71153
Streptococcus pneumoniae G54 uid59167
Streptococcus pneumoniae Hungary19A 6 uid59117
Streptococcus pneumoniae INV200 uid162035
Streptococcus pneumoniae TIGR4 uid57857
Streptococcus pneumoniae CGSP14 uid59181
Streptococcus pneumoniae 670 6B uid52533
Streptococcus pneumoniae ATCC 700669 uid59287
Streptococcus pneumoniae INV104 uid162039
Streptococcus pneumoniae JJA uid59121
Streptococcus pneumoniae OXC141 uid162037
Streptococcus pneumoniae P1031 uid59123
Streptococcus pneumoniae 70585 uid59125
Streptococcus pneumoniae AP200 uid52453
Streptococcus pneumoniae D39 uid58581
Streptococcus pneumoniae R6 uid57859
Streptococcus pneumoniae ST556 uid162191
Streptococcus pneumoniae TCH8431 19A uid49735
Streptococcus pneumoniae Taiwan19F 14 uid59119
Streptococcus salivarius CCHSS3 uid70481
Streptococcus salivarius JIM8777 uid162145
Streptococcus thermophilus JIM 8232 uid162157
Streptococcus thermophilus LMD 9 uid58327
Streptococcus thermophilus MN ZLW 002 uid166827
Streptococcus thermophilus ND03 uid162015
Streptococcus thermophilus CNRZ1066 uid58221
Streptococcus thermophilus LMG 18311 uid58219
Streptococcus mutans LJ23 uid162197
Streptococcus mutans NN2025 uid46353
Streptococcus mutans UA159 uid57947
Streptococcus agalactiae A909 uid57935
Streptococcus agalactiae 2603V R uid57943
Streptococcus agalactiae NEM316 uid61585
Streptococcus infantarius CJ18 uid87033
Streptococcus pasteurianus ATCC 43144 uid68019
Streptococcus macedonicus ACA DC 198 uid81631
Streptococcus gallolyticus ATCC BAA 2069 uid63617
Streptococcus gallolyticus ATCC 43143 uid162103
Streptococcus gallolyticus UCN34 uid46061
Streptococcus parauberis KCTC 11537 uid67355
Streptococcus uberis 0140J uid57959
Streptococcus equi zooepidemicus uid59261
Streptococcus equi 4047 uid59259
Streptococcus equi zooepidemicus MGCS10565 uid59263
Streptococcus dysgalactiae equisimilis ATCC 12394 uid161979
Streptococcus dysgalactiae equisimilis GGS 124 uid59103
Streptococcus pyogenes Manfredo uid57847
Streptococcus pyogenes NZ131 uid59035
Streptococcus pyogenes MGAS10394 uid58105
Streptococcus pyogenes MGAS15252 uid158037
Streptococcus pyogenes MGAS1882 uid158061
Streptococcus pyogenes MGAS8232 uid57871
Streptococcus pyogenes Alab49 uid162171
Streptococcus pyogenes MGAS315 uid57911
Streptococcus pyogenes SSI 1 uid57895
secY

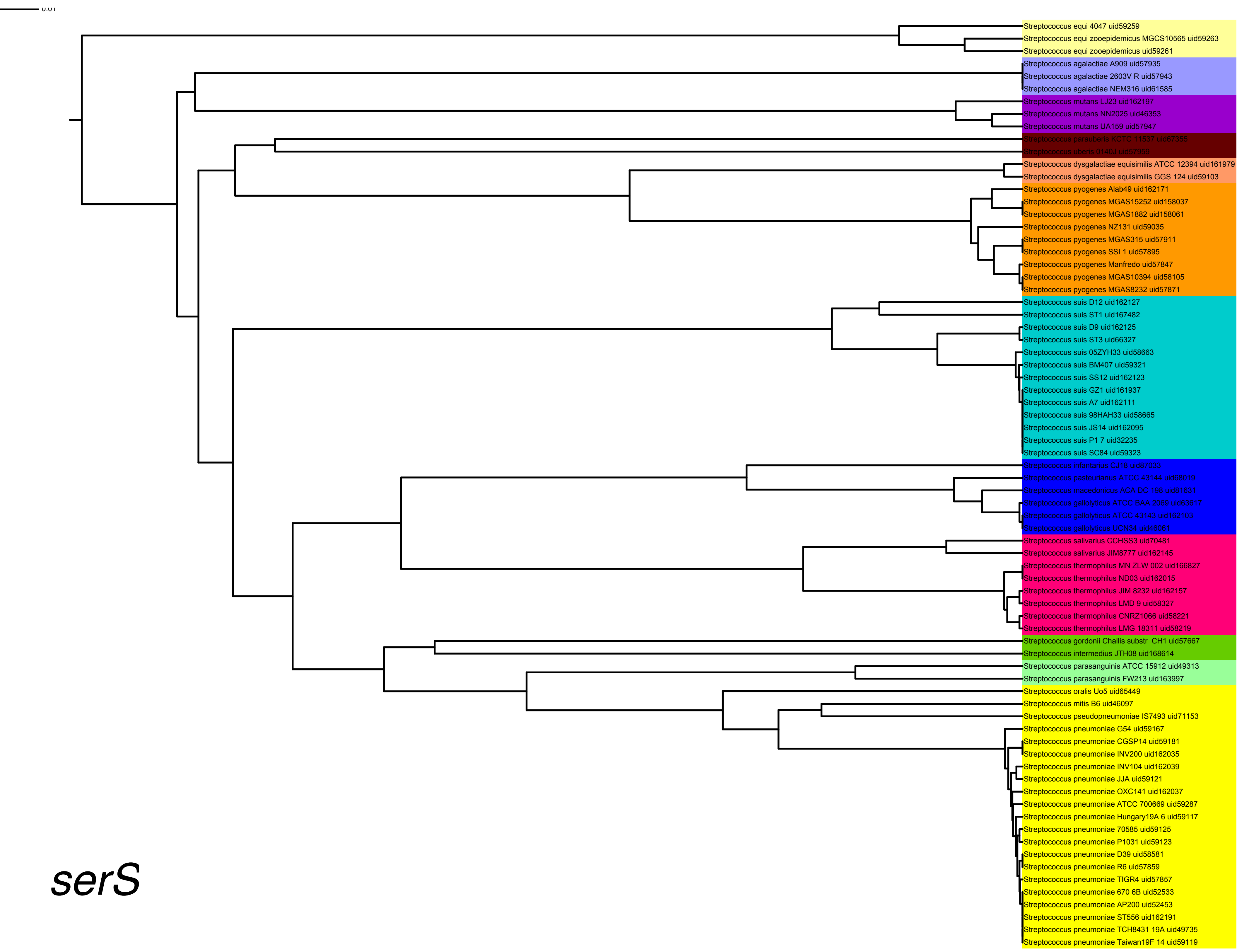
0.01
Streptococcus equi 4047 uid59259
Streptococcus equi zooepidemicus MGCS10565 uid59263
Streptococcus equi zooepidemicus uid59261
Streptococcus agalactiae A909 uid57935
Streptococcus agalactiae 2603V R uid57943
Streptococcus agalactiae NEM316 uid61585
Streptococcus mutans LJ23 uid162197
Streptococcus mutans NN2025 uid46353
Streptococcus mutans UA159 uid57947
Streptococcus parauberis KCTC 11537 uid67355
Streptococcus uberis 0140J uid57959
Streptococcus dysgalactiae equisimilis ATCC 12394 uid161979
Streptococcus dysgalactiae equisimilis GGS 124 uid59103
Streptococcus pyogenes Alab49 uid162171
Streptococcus pyogenes MGAS15252 uid158037
Streptococcus pyogenes MGAS1882 uid158061
Streptococcus pyogenes NZ131 uid59035
Streptococcus pyogenes MGAS315 uid57911
Streptococcus pyogenes SSI 1 uid57895
Streptococcus pyogenes Manfredo uid57847
Streptococcus pyogenes MGAS10394 uid58105
Streptococcus pyogenes MGAS8232 uid57871
Streptococcus suis D12 uid162127
Streptococcus suis ST1 uid167482
Streptococcus suis D9 uid162125
Streptococcus suis ST3 uid66327
Streptococcus suis 05ZYH33 uid58663
Streptococcus suis BM407 uid59321
Streptococcus suis SS12 uid162123
Streptococcus suis GZ1 uid161937
Streptococcus suis A7 uid162111
Streptococcus suis 98HAH33 uid58665
Streptococcus suis JS14 uid162095
Streptococcus suis P1 7 uid32235
Streptococcus suis SC84 uid59323
Streptococcus infantarius CJ18 uid87033
Streptococcus pasteurianus ATCC 43144 uid68019
Streptococcus macedonicus ACA DC 198 uid81631
Streptococcus gallolyticus ATCC BAA 2069 uid63617
Streptococcus gallolyticus ATCC 43143 uid162103
Streptococcus gallolyticus UCN34 uid46061
Streptococcus salivarius CCHSS3 uid70481
Streptococcus salivarius JIM8777 uid162145
Streptococcus thermophilus MN ZLW 002 uid166827
Streptococcus thermophilus ND03 uid162015
Streptococcus thermophilus JIM 8232 uid162157
Streptococcus thermophilus LMD 9 uid58327
Streptococcus thermophilus CNRZ1066 uid58221
Streptococcus thermophilus LMG 18311 uid58219
Streptococcus gordonii Challis substr CH1 uid57667
Streptococcus intermedius JTH08 uid168614
Streptococcus parasanguinis ATCC 15912 uid49313
Streptococcus parasanguinis FW213 uid163997
Streptococcus oralis Uo5 uid65449
Streptococcus mitis B6 uid46097
Streptococcus pseudopneumoniae IS7493 uid71153
Streptococcus pneumoniae G54 uid59167
Streptococcus pneumoniae CGSP14 uid59181
Streptococcus pneumoniae INV200 uid162035
Streptococcus pneumoniae INV104 uid162039
Streptococcus pneumoniae JJA uid59121
Streptococcus pneumoniae OXC141 uid162037
Streptococcus pneumoniae ATCC 700669 uid59287
Streptococcus pneumoniae Hungary19A 6 uid59117
Streptococcus pneumoniae 70585 uid59125
Streptococcus pneumoniae P1031 uid59123
Streptococcus pneumoniae D39 uid58581
Streptococcus pneumoniae R6 uid57859
Streptococcus pneumoniae TIGR4 uid57857
Streptococcus pneumoniae 670 6B uid52533
Streptococcus pneumoniae AP200 uid52453
Streptococcus pneumoniae ST556 uid162191
Streptococcus pneumoniae TCH8431 19A uid49735
Streptococcus pneumoniae Taiwan19F 14 uid59119
serS

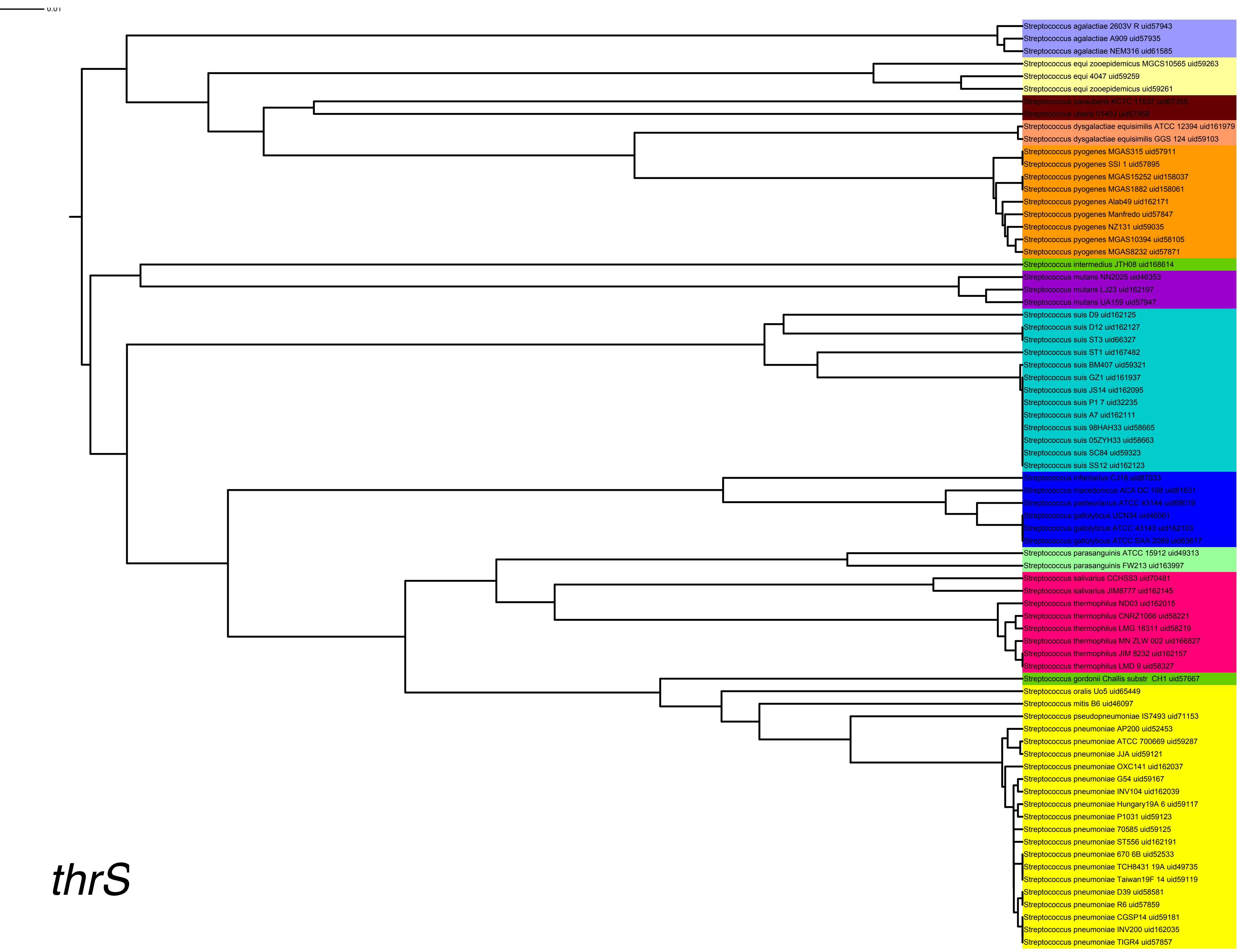
0.01
Streptococcus agalactiae 2603V R uid57943
Streptococcus agalactiae A909 uid57935
Streptococcus agalactiae NEM316 uid61585
Streptococcus equi zooepidemicus MGCS10565 uid59263
Streptococcus equi 4047 uid59259
Streptococcus equi zooepidemicus uid59261
Streptococcus parauberis KCTC 11537 uid67355
Streptococcus uberis 0140J uid57959
Streptococcus dysgalactiae equisimilis ATCC 12394 uid161979
Streptococcus dysgalactiae equisimilis GGS 124 uid59103
Streptococcus pyogenes MGAS315 uid57911
Streptococcus pyogenes SSI 1 uid57895
Streptococcus pyogenes MGAS15252 uid158037
Streptococcus pyogenes MGAS1882 uid158061
Streptococcus pyogenes Alab49 uid162171
Streptococcus pyogenes Manfredo uid57847
Streptococcus pyogenes NZ131 uid59035
Streptococcus pyogenes MGAS10394 uid58105
Streptococcus pyogenes MGAS8232 uid57871
Streptococcus intermedius JTH08 uid168614
Streptococcus mutans NN2025 uid46353
Streptococcus mutans LJ23 uid162197
Streptococcus mutans UA159 uid57947
Streptococcus suis D9 uid162125
Streptococcus suis D12 uid162127
Streptococcus suis ST3 uid66327
Streptococcus suis ST1 uid167482
Streptococcus suis BM407 uid59321
Streptococcus suis GZ1 uid161937
Streptococcus suis JS14 uid162095
Streptococcus suis P1 7 uid32235
Streptococcus suis A7 uid162111
Streptococcus suis 98HAH33 uid58665
Streptococcus suis 05ZYH33 uid58663
Streptococcus suis SC84 uid59323
Streptococcus suis SS12 uid162123
Streptococcus infantarius CJ18 uid87033
Streptococcus macedonicus ACA DC 198 uid81631
Streptococcus pasteurianus ATCC 43144 uid68019
Streptococcus gallolyticus UCN34 uid46061
Streptococcus gallolyticus ATCC 43143 uid162103
Streptococcus gallolyticus ATCC BAA 2069 uid63617
Streptococcus parasanguinis ATCC 15912 uid49313
Streptococcus parasanguinis FW213 uid163997
Streptococcus salivarius CCHSS3 uid70481
Streptococcus salivarius JIM8777 uid162145
Streptococcus thermophilus ND03 uid162015
Streptococcus thermophilus CNRZ1066 uid58221
Streptococcus thermophilus LMG 18311 uid58219
Streptococcus thermophilus MN ZLW 002 uid166827
Streptococcus thermophilus JIM 8232 uid162157
Streptococcus thermophilus LMD 9 uid58327
Streptococcus gordonii Challis substr CH1 uid57667
Streptococcus oralis Uo5 uid65449
Streptococcus mitis B6 uid46097
Streptococcus pseudopneumoniae IS7493 uid71153
Streptococcus pneumoniae AP200 uid52453
Streptococcus pneumoniae ATCC 700669 uid59287
Streptococcus pneumoniae JJA uid59121
Streptococcus pneumoniae OXC141 uid162037
Streptococcus pneumoniae G54 uid59167
Streptococcus pneumoniae INV104 uid162039
Streptococcus pneumoniae Hungary19A 6 uid59117
Streptococcus pneumoniae P1031 uid59123
Streptococcus pneumoniae 70585 uid59125
Streptococcus pneumoniae ST556 uid162191
Streptococcus pneumoniae 670 6B uid52533
Streptococcus pneumoniae TCH8431 19A uid49735
Streptococcus pneumoniae Taiwan19F 14 uid59119
Streptococcus pneumoniae D39 uid58581
Streptococcus pneumoniae R6 uid57859
Streptococcus pneumoniae CGSP14 uid59181
Streptococcus pneumoniae INV200 uid162035
Streptococcus pneumoniae TIGR4 uid57857
thrS

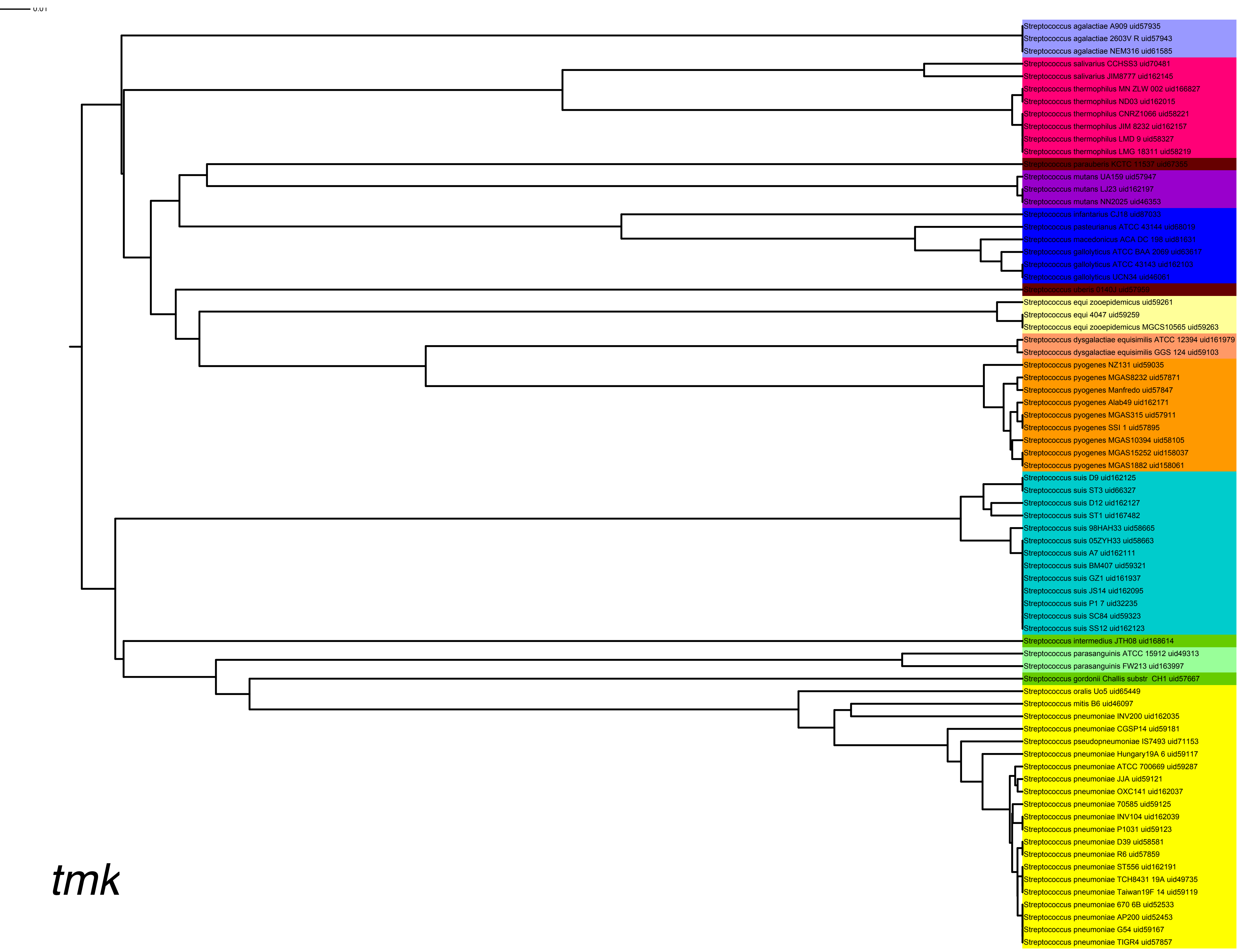
0.01
Streptococcus agalactiae A909 uid57935
Streptococcus agalactiae 2603V R uid57943
Streptococcus agalactiae NEM316 uid61585
Streptococcus salivarius CCHSS3 uid70481
Streptococcus salivarius JIM8777 uid162145
Streptococcus thermophilus MN ZLW 002 uid166827
Streptococcus thermophilus ND03 uid162015
Streptococcus thermophilus CNRZ1066 uid58221
Streptococcus thermophilus JIM 8232 uid162157
Streptococcus thermophilus LMD 9 uid58327
Streptococcus thermophilus LMG 18311 uid58219
Streptococcus parauberis KCTC 11537 uid67355
Streptococcus mutans UA159 uid57947
Streptococcus mutans LJ23 uid162197
Streptococcus mutans NN2025 uid46353
Streptococcus infantarius CJ18 uid87033
Streptococcus pasteurianus ATCC 43144 uid68019
Streptococcus macedonicus ACA DC 198 uid81631
Streptococcus gallolyticus ATCC BAA 2069 uid63617
Streptococcus gallolyticus ATCC 43143 uid162103
Streptococcus gallolyticus UCN34 uid46061
Streptococcus uberis 0140J uid57959
Streptococcus equi zooepidemicus uid59261
Streptococcus equi 4047 uid59259
Streptococcus equi zooepidemicus MGCS10565 uid59263
Streptococcus dysgalactiae equisimilis ATCC 12394 uid161979
Streptococcus dysgalactiae equisimilis GGS 124 uid59103
Streptococcus pyogenes NZ131 uid59035
Streptococcus pyogenes MGAS8232 uid57871
Streptococcus pyogenes Manfredo uid57847
Streptococcus pyogenes Alab49 uid162171
Streptococcus pyogenes MGAS315 uid57911
Streptococcus pyogenes SSI 1 uid57895
Streptococcus pyogenes MGAS10394 uid58105
Streptococcus pyogenes MGAS15252 uid158037
Streptococcus pyogenes MGAS1882 uid158061
Streptococcus suis D9 uid162125
Streptococcus suis ST3 uid66327
Streptococcus suis D12 uid162127
Streptococcus suis ST1 uid167482
Streptococcus suis 98HAH33 uid58665
Streptococcus suis 05ZYH33 uid58663
Streptococcus suis A7 uid162111
Streptococcus suis BM407 uid59321
Streptococcus suis GZ1 uid161937
Streptococcus suis JS14 uid162095
Streptococcus suis P1 7 uid32235
Streptococcus suis SC84 uid59323
Streptococcus suis SS12 uid162123
Streptococcus intermedius JTH08 uid168614
Streptococcus parasanguinis ATCC 15912 uid49313
Streptococcus parasanguinis FW213 uid163997
Streptococcus gordonii Challis substr CH1 uid57667
Streptococcus oralis Uo5 uid65449
Streptococcus mitis B6 uid46097
Streptococcus pneumoniae INV200 uid162035
Streptococcus pneumoniae CGSP14 uid59181
Streptococcus pseudopneumoniae IS7493 uid71153
Streptococcus pneumoniae Hungary19A 6 uid59117
Streptococcus pneumoniae ATCC 700669 uid59287
Streptococcus pneumoniae JJA uid59121
Streptococcus pneumoniae OXC141 uid162037
Streptococcus pneumoniae 70585 uid59125
Streptococcus pneumoniae INV104 uid162039
Streptococcus pneumoniae P1031 uid59123
Streptococcus pneumoniae D39 uid58581
Streptococcus pneumoniae R6 uid57859
Streptococcus pneumoniae ST556 uid162191
Streptococcus pneumoniae TCH8431 19A uid49735
Streptococcus pneumoniae Taiwan19F 14 uid59119
Streptococcus pneumoniae 670 6B uid52533
Streptococcus pneumoniae AP200 uid52453
Streptococcus pneumoniae G54 uid59167
Streptococcus pneumoniae TIGR4 uid57857
tmk

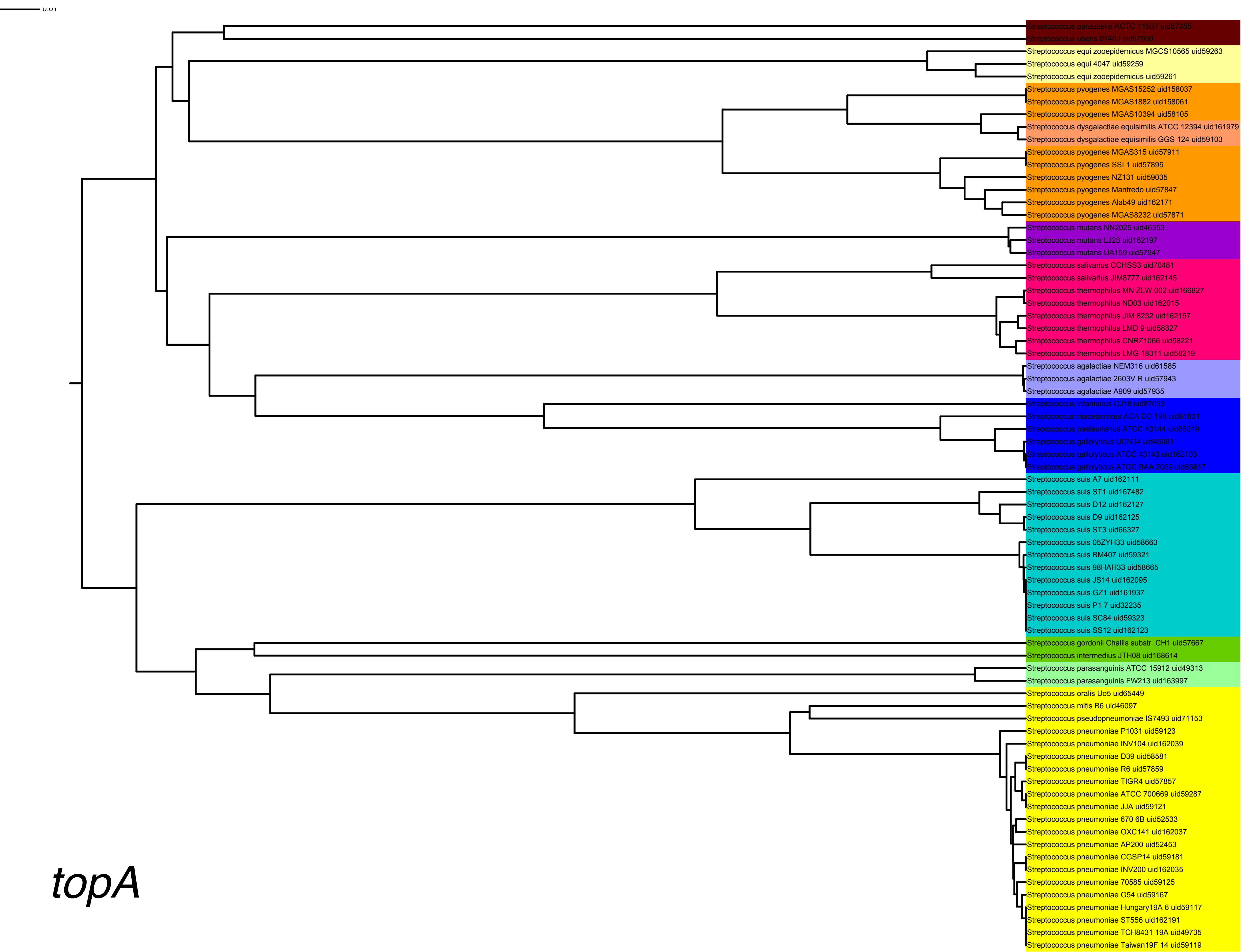
0.01
Streptococcus parauberis KCTC 11537 uid67355
Streptococcus uberis 0140J uid57959
Streptococcus equi zooepidemicus MGCS10565 uid59263
Streptococcus equi 4047 uid59259
Streptococcus equi zooepidemicus uid59261
Streptococcus pyogenes MGAS15252 uid158037
Streptococcus pyogenes MGAS1882 uid158061
Streptococcus pyogenes MGAS10394 uid58105
Streptococcus dysgalactiae equisimilis ATCC 12394 uid161979
Streptococcus dysgalactiae equisimilis GGS 124 uid59103
Streptococcus pyogenes MGAS315 uid57911
Streptococcus pyogenes SSI 1 uid57895
Streptococcus pyogenes NZ131 uid59035
Streptococcus pyogenes Manfredo uid57847
Streptococcus pyogenes Alab49 uid162171
Streptococcus pyogenes MGAS8232 uid57871
Streptococcus mutans NN2025 uid46353
Streptococcus mutans LJ23 uid162197
Streptococcus mutans UA159 uid57947
Streptococcus salivarius CCHSS3 uid70481
Streptococcus salivarius JIM8777 uid162145
Streptococcus thermophilus MN ZLW 002 uid166827
Streptococcus thermophilus ND03 uid162015
Streptococcus thermophilus JIM 8232 uid162157
Streptococcus thermophilus LMD 9 uid58327
Streptococcus thermophilus CNRZ1066 uid58221
Streptococcus thermophilus LMG 18311 uid58219
Streptococcus agalactiae NEM316 uid61585
Streptococcus agalactiae 2603V R uid57943
Streptococcus agalactiae A909 uid57935
Streptococcus infantarius CJ18 uid87033
Streptococcus macedonicus ACA DC 198 uid81631
Streptococcus pasteurianus ATCC 43144 uid68019
Streptococcus gallolyticus UCN34 uid46061
Streptococcus gallolyticus ATCC 43143 uid162103
Streptococcus gallolyticus ATCC BAA 2069 uid63617
Streptococcus suis A7 uid162111
Streptococcus suis ST1 uid167482
Streptococcus suis D12 uid162127
Streptococcus suis D9 uid162125
Streptococcus suis ST3 uid66327
Streptococcus suis 05ZYH33 uid58663
Streptococcus suis BM407 uid59321
Streptococcus suis 98HAH33 uid58665
Streptococcus suis JS14 uid162095
Streptococcus suis GZ1 uid161937
Streptococcus suis P1 7 uid32235
Streptococcus suis SC84 uid59323
Streptococcus suis SS12 uid162123
Streptococcus gordonii Challis substr CH1 uid57667
Streptococcus intermedius JTH08 uid168614
Streptococcus parasanguinis ATCC 15912 uid49313
Streptococcus parasanguinis FW213 uid163997
Streptococcus oralis Uo5 uid65449
Streptococcus mitis B6 uid46097
Streptococcus pseudopneumoniae IS7493 uid71153
Streptococcus pneumoniae P1031 uid59123
Streptococcus pneumoniae INV104 uid162039
Streptococcus pneumoniae D39 uid58581
Streptococcus pneumoniae R6 uid57859
Streptococcus pneumoniae TIGR4 uid57857
Streptococcus pneumoniae ATCC 700669 uid59287
Streptococcus pneumoniae JJA uid59121
Streptococcus pneumoniae 670 6B uid52533
Streptococcus pneumoniae OXC141 uid162037
Streptococcus pneumoniae AP200 uid52453
Streptococcus pneumoniae CGSP14 uid59181
Streptococcus pneumoniae INV200 uid162035
Streptococcus pneumoniae 70585 uid59125
Streptococcus pneumoniae G54 uid59167
Streptococcus pneumoniae Hungary19A 6 uid59117
Streptococcus pneumoniae ST556 uid162191
Streptococcus pneumoniae TCH8431 19A uid49735
Streptococcus pneumoniae Taiwan19F 14 uid59119
topA

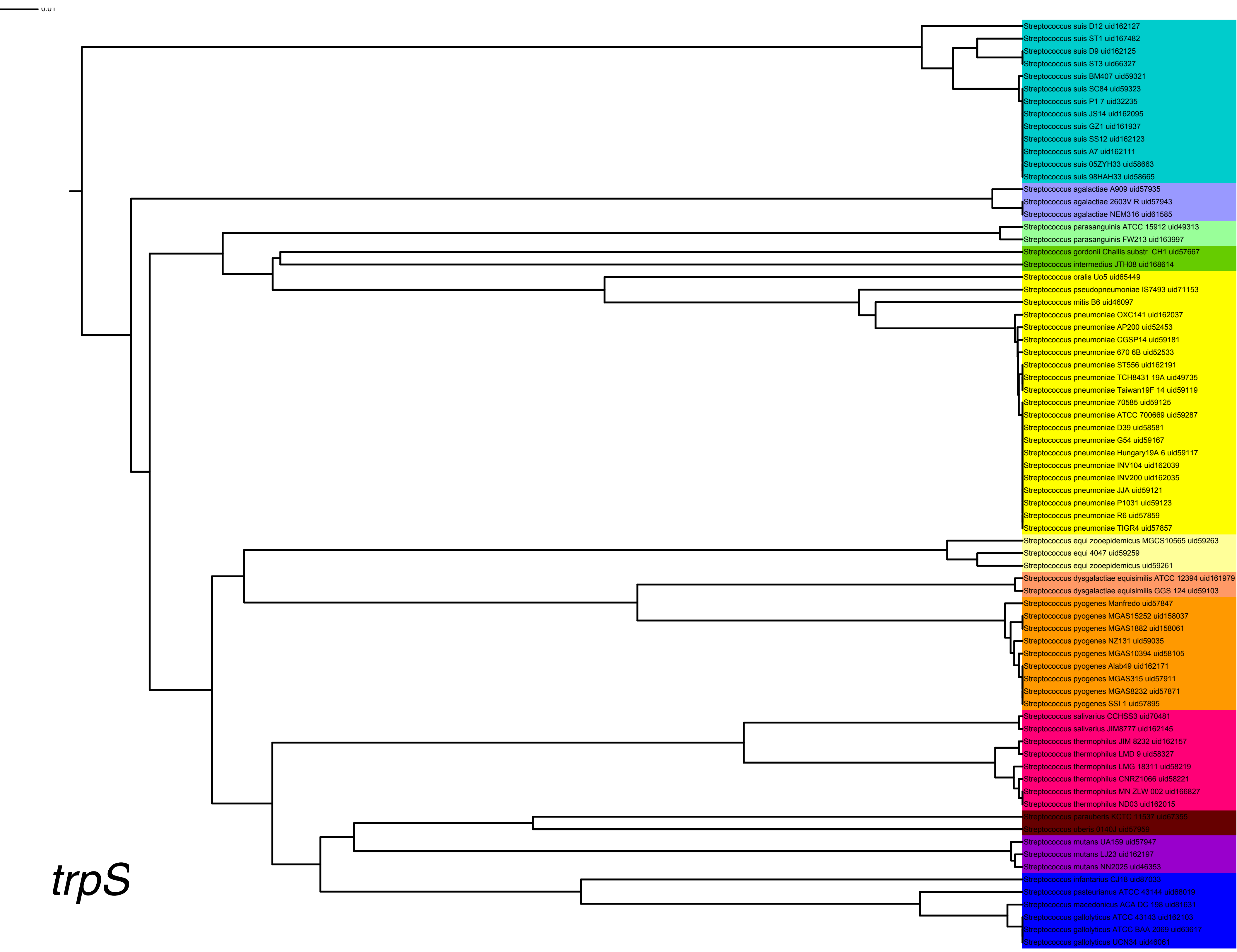
0.01
Streptococcus suis D12 uid162127
Streptococcus suis ST1 uid167482
Streptococcus suis D9 uid162125
Streptococcus suis ST3 uid66327
Streptococcus suis BM407 uid59321
Streptococcus suis SC84 uid59323
Streptococcus suis P1 7 uid32235
Streptococcus suis JS14 uid162095
Streptococcus suis GZ1 uid161937
Streptococcus suis SS12 uid162123
Streptococcus suis A7 uid162111
Streptococcus suis 05ZYH33 uid58663
Streptococcus suis 98HAH33 uid58665
Streptococcus agalactiae A909 uid57935
Streptococcus agalactiae 2603V R uid57943
Streptococcus agalactiae NEM316 uid61585
Streptococcus parasanguinis ATCC 15912 uid49313
Streptococcus parasanguinis FW213 uid163997
Streptococcus gordonii Challis substr CH1 uid57667
Streptococcus intermedius JTH08 uid168614
Streptococcus oralis Uo5 uid65449
Streptococcus pseudopneumoniae IS7493 uid71153
Streptococcus mitis B6 uid46097
Streptococcus pneumoniae OXC141 uid162037
Streptococcus pneumoniae AP200 uid52453
Streptococcus pneumoniae CGSP14 uid59181
Streptococcus pneumoniae 670 6B uid52533
Streptococcus pneumoniae ST556 uid162191
Streptococcus pneumoniae TCH8431 19A uid49735
Streptococcus pneumoniae Taiwan19F 14 uid59119
Streptococcus pneumoniae 70585 uid59125
Streptococcus pneumoniae ATCC 700669 uid59287
Streptococcus pneumoniae D39 uid58581
Streptococcus pneumoniae G54 uid59167
Streptococcus pneumoniae Hungary19A 6 uid59117
Streptococcus pneumoniae INV104 uid162039
Streptococcus pneumoniae INV200 uid162035
Streptococcus pneumoniae JJA uid59121
Streptococcus pneumoniae P1031 uid59123
Streptococcus pneumoniae R6 uid57859
Streptococcus pneumoniae TIGR4 uid57857
Streptococcus equi zooepidemicus MGCS10565 uid59263
Streptococcus equi 4047 uid59259
Streptococcus equi zooepidemicus uid59261
Streptococcus dysgalactiae equisimilis ATCC 12394 uid161979
Streptococcus dysgalactiae equisimilis GGS 124 uid59103
Streptococcus pyogenes Manfredo uid57847
Streptococcus pyogenes MGAS15252 uid158037
Streptococcus pyogenes MGAS1882 uid158061
Streptococcus pyogenes NZ131 uid59035
Streptococcus pyogenes MGAS10394 uid58105
Streptococcus pyogenes Alab49 uid162171
Streptococcus pyogenes MGAS315 uid57911
Streptococcus pyogenes MGAS8232 uid57871
Streptococcus pyogenes SSI 1 uid57895
Streptococcus salivarius CCHSS3 uid70481
Streptococcus salivarius JIM8777 uid162145
Streptococcus thermophilus JIM 8232 uid162157
Streptococcus thermophilus LMD 9 uid58327
Streptococcus thermophilus LMG 18311 uid58219
Streptococcus thermophilus CNRZ1066 uid58221
Streptococcus thermophilus MN ZLW 002 uid166827
Streptococcus thermophilus ND03 uid162015
Streptococcus parauberis KCTC 11537 uid67355
Streptococcus uberis 0140J uid57959
Streptococcus mutans UA159 uid57947
Streptococcus mutans LJ23 uid162197
Streptococcus mutans NN2025 uid46353
Streptococcus infantarius CJ18 uid87033
Streptococcus pasteurianus ATCC 43144 uid68019
Streptococcus macedonicus ACA DC 198 uid81631
Streptococcus gallolyticus ATCC 43143 uid162103
Streptococcus gallolyticus ATCC BAA 2069 uid63617
Streptococcus gallolyticus UCN34 uid46061
trpS

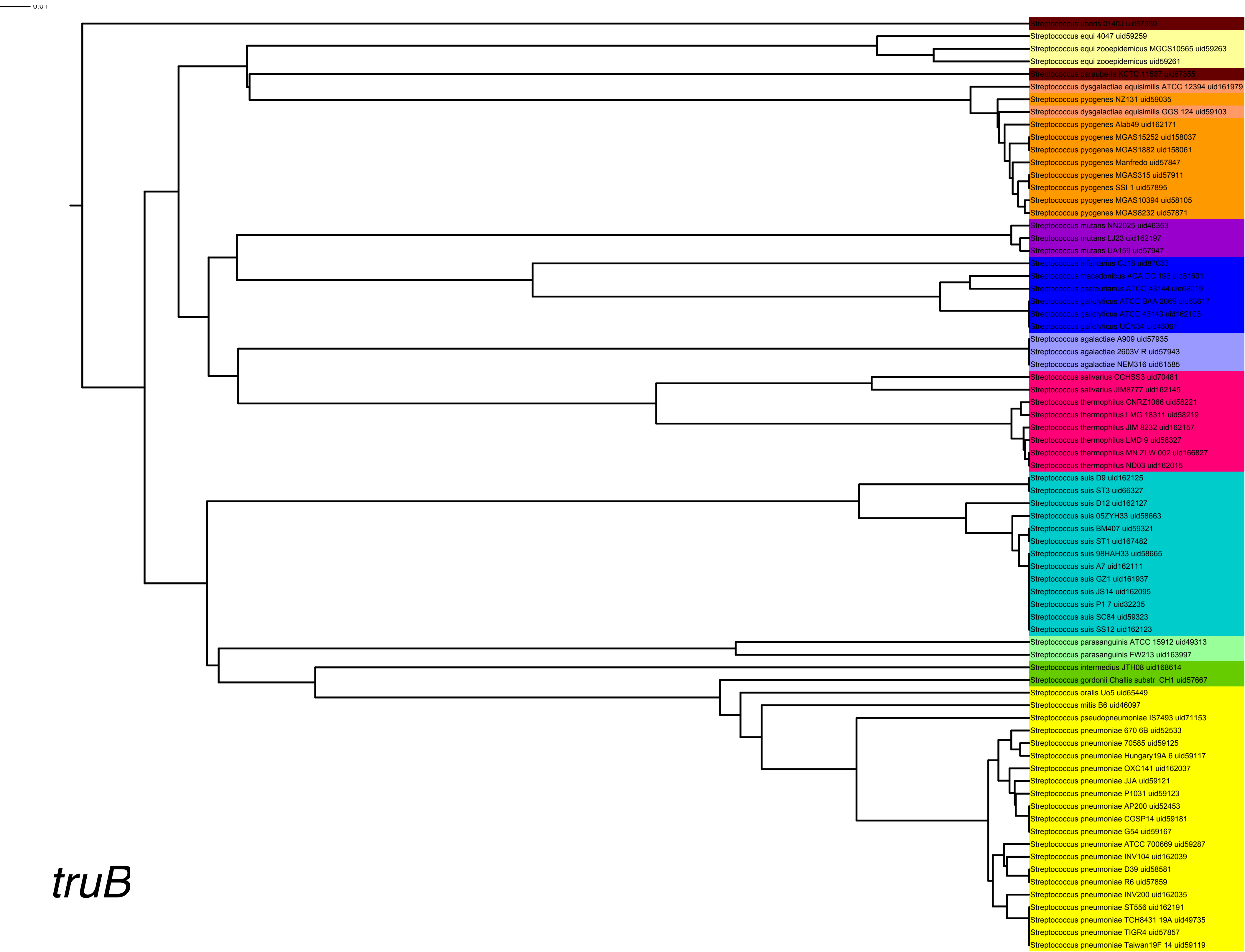

0.01
Streptococcus uberis 0140J uid57959
Streptococcus equi 4047 uid59259
Streptococcus equi zooepidemicus MGCS10565 uid59263
Streptococcus equi zooepidemicus uid59261
Streptococcus parauberis KCTC 11537 uid67355
Streptococcus dysgalactiae equisimilis ATCC 12394 uid161979
Streptococcus pyogenes NZ131 uid59035
Streptococcus dysgalactiae equisimilis GGS 124 uid59103
Streptococcus pyogenes Alab49 uid162171
Streptococcus pyogenes MGAS15252 uid158037
Streptococcus pyogenes MGAS1882 uid158061
Streptococcus pyogenes Manfredo uid57847
Streptococcus pyogenes MGAS315 uid57911
Streptococcus pyogenes SSI 1 uid57895
Streptococcus pyogenes MGAS10394 uid58105
Streptococcus pyogenes MGAS8232 uid57871
Streptococcus mutans NN2025 uid46353
Streptococcus mutans LJ23 uid162197
Streptococcus mutans UA159 uid57947
Streptococcus infantarius CJ18 uid87033
Streptococcus macedonicus ACA DC 198 uid81631
Streptococcus pasteurianus ATCC 43144 uid68019
Streptococcus gallolyticus ATCC BAA 2069 uid63617
Streptococcus gallolyticus ATCC 43143 uid162103
Streptococcus gallolyticus UCN34 uid46061
Streptococcus agalactiae A909 uid57935
Streptococcus agalactiae 2603V R uid57943
Streptococcus agalactiae NEM316 uid61585
Streptococcus salivarius CCHSS3 uid70481
Streptococcus salivarius JIM8777 uid162145
Streptococcus thermophilus CNRZ1066 uid58221
Streptococcus thermophilus LMG 18311 uid58219
Streptococcus thermophilus JIM 8232 uid162157
Streptococcus thermophilus LMD 9 uid58327
Streptococcus thermophilus MN ZLW 002 uid166827
Streptococcus thermophilus ND03 uid162015
Streptococcus suis D9 uid162125
Streptococcus suis ST3 uid66327
Streptococcus suis D12 uid162127
Streptococcus suis 05ZYH33 uid58663
Streptococcus suis BM407 uid59321
Streptococcus suis ST1 uid167482
Streptococcus suis 98HAH33 uid58665
Streptococcus suis A7 uid162111
Streptococcus suis GZ1 uid161937
Streptococcus suis JS14 uid162095
Streptococcus suis P1 7 uid32235
Streptococcus suis SC84 uid59323
Streptococcus suis SS12 uid162123
Streptococcus parasanguinis ATCC 15912 uid49313
Streptococcus parasanguinis FW213 uid163997
Streptococcus intermedius JTH08 uid168614
Streptococcus gordonii Challis substr CH1 uid57667
Streptococcus oralis Uo5 uid65449
Streptococcus mitis B6 uid46097
Streptococcus pseudopneumoniae IS7493 uid71153
Streptococcus pneumoniae 670 6B uid52533
Streptococcus pneumoniae 70585 uid59125
Streptococcus pneumoniae Hungary19A 6 uid59117
Streptococcus pneumoniae OXC141 uid162037
Streptococcus pneumoniae JJA uid59121
Streptococcus pneumoniae P1031 uid59123
Streptococcus pneumoniae AP200 uid52453
Streptococcus pneumoniae CGSP14 uid59181
Streptococcus pneumoniae G54 uid59167
Streptococcus pneumoniae ATCC 700669 uid59287
Streptococcus pneumoniae INV104 uid162039
Streptococcus pneumoniae D39 uid58581
Streptococcus pneumoniae R6 uid57859
Streptococcus pneumoniae INV200 uid162035
Streptococcus pneumoniae ST556 uid162191
Streptococcus pneumoniae TCH8431 19A uid49735
Streptococcus pneumoniae TIGR4 uid57857
Streptococcus pneumoniae Taiwan19F 14 uid59119

truB

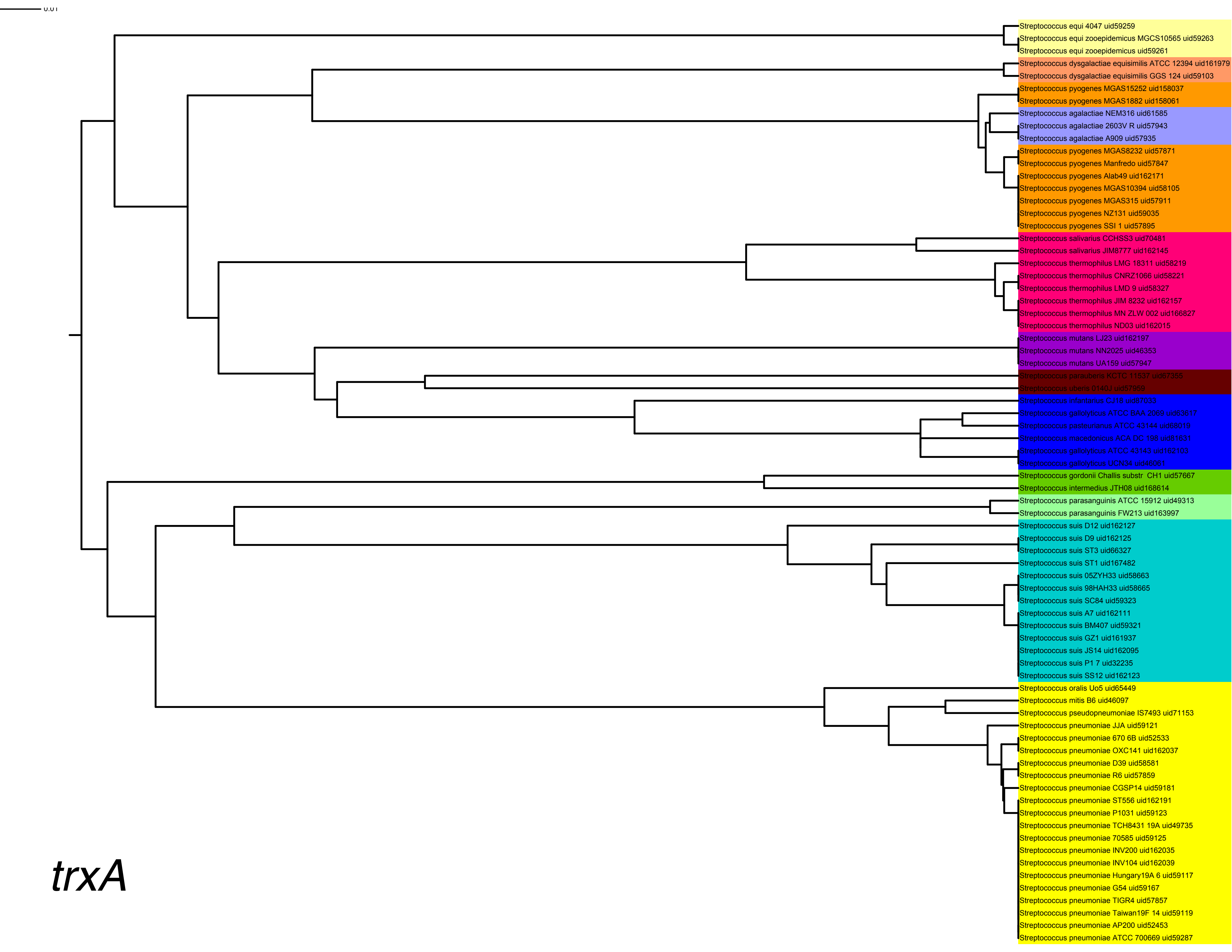
0.01
Streptococcus equi 4047 uid59259
Streptococcus equi zooepidemicus MGCS10565 uid59263
Streptococcus equi zooepidemicus uid59261
Streptococcus dysgalactiae equisimilis ATCC 12394 uid161979
Streptococcus dysgalactiae equisimilis GGS 124 uid59103
Streptococcus pyogenes MGAS15252 uid158037
Streptococcus pyogenes MGAS1882 uid158061
Streptococcus agalactiae NEM316 uid61585
Streptococcus agalactiae 2603V R uid57943
Streptococcus agalactiae A909 uid57935
Streptococcus pyogenes MGAS8232 uid57871
Streptococcus pyogenes Manfredo uid57847
Streptococcus pyogenes Alab49 uid162171
Streptococcus pyogenes MGAS10394 uid58105
Streptococcus pyogenes MGAS315 uid57911
Streptococcus pyogenes NZ131 uid59035
Streptococcus pyogenes SSI 1 uid57895
Streptococcus salivarius CCHSS3 uid70481
Streptococcus salivarius JIM8777 uid162145
Streptococcus thermophilus LMG 18311 uid58219
Streptococcus thermophilus CNRZ1066 uid58221
Streptococcus thermophilus LMD 9 uid58327
Streptococcus thermophilus JIM 8232 uid162157
Streptococcus thermophilus MN ZLW 002 uid166827
Streptococcus thermophilus ND03 uid162015
Streptococcus mutans LJ23 uid162197
Streptococcus mutans NN2025 uid46353
Streptococcus mutans UA159 uid57947
Streptococcus parauberis KCTC 11537 uid67355
Streptococcus uberis 0140J uid57959
Streptococcus infantarius CJ18 uid87033
Streptococcus gallolyticus ATCC BAA 2069 uid63617
Streptococcus pasteurianus ATCC 43144 uid68019
Streptococcus macedonicus ACA DC 198 uid81631
Streptococcus gallolyticus ATCC 43143 uid162103
Streptococcus gallolyticus UCN34 uid46061
Streptococcus gordonii Challis substr CH1 uid57667
Streptococcus intermedius JTH08 uid168614
Streptococcus parasanguinis ATCC 15912 uid49313
Streptococcus parasanguinis FW213 uid163997
Streptococcus suis D12 uid162127
Streptococcus suis D9 uid162125
Streptococcus suis ST3 uid66327
Streptococcus suis ST1 uid167482
Streptococcus suis 05ZYH33 uid58663
Streptococcus suis 98HAH33 uid58665
Streptococcus suis SC84 uid59323
Streptococcus suis A7 uid162111
Streptococcus suis BM407 uid59321
Streptococcus suis GZ1 uid161937
Streptococcus suis JS14 uid162095
Streptococcus suis P1 7 uid32235
Streptococcus suis SS12 uid162123
Streptococcus oralis Uo5 uid65449
Streptococcus mitis B6 uid46097
Streptococcus pseudopneumoniae IS7493 uid71153
Streptococcus pneumoniae JJA uid59121
Streptococcus pneumoniae 670 6B uid52533
Streptococcus pneumoniae OXC141 uid162037
Streptococcus pneumoniae D39 uid58581
Streptococcus pneumoniae R6 uid57859
Streptococcus pneumoniae CGSP14 uid59181
Streptococcus pneumoniae ST556 uid162191
Streptococcus pneumoniae P1031 uid59123
Streptococcus pneumoniae TCH8431 19A uid49735
Streptococcus pneumoniae 70585 uid59125
Streptococcus pneumoniae INV200 uid162035
Streptococcus pneumoniae INV104 uid162039
Streptococcus pneumoniae Hungary19A 6 uid59117
Streptococcus pneumoniae G54 uid59167
Streptococcus pneumoniae TIGR4 uid57857
Streptococcus pneumoniae Taiwan19F 14 uid59119
Streptococcus pneumoniae AP200 uid52453
Streptococcus pneumoniae ATCC 700669 uid59287
trxA

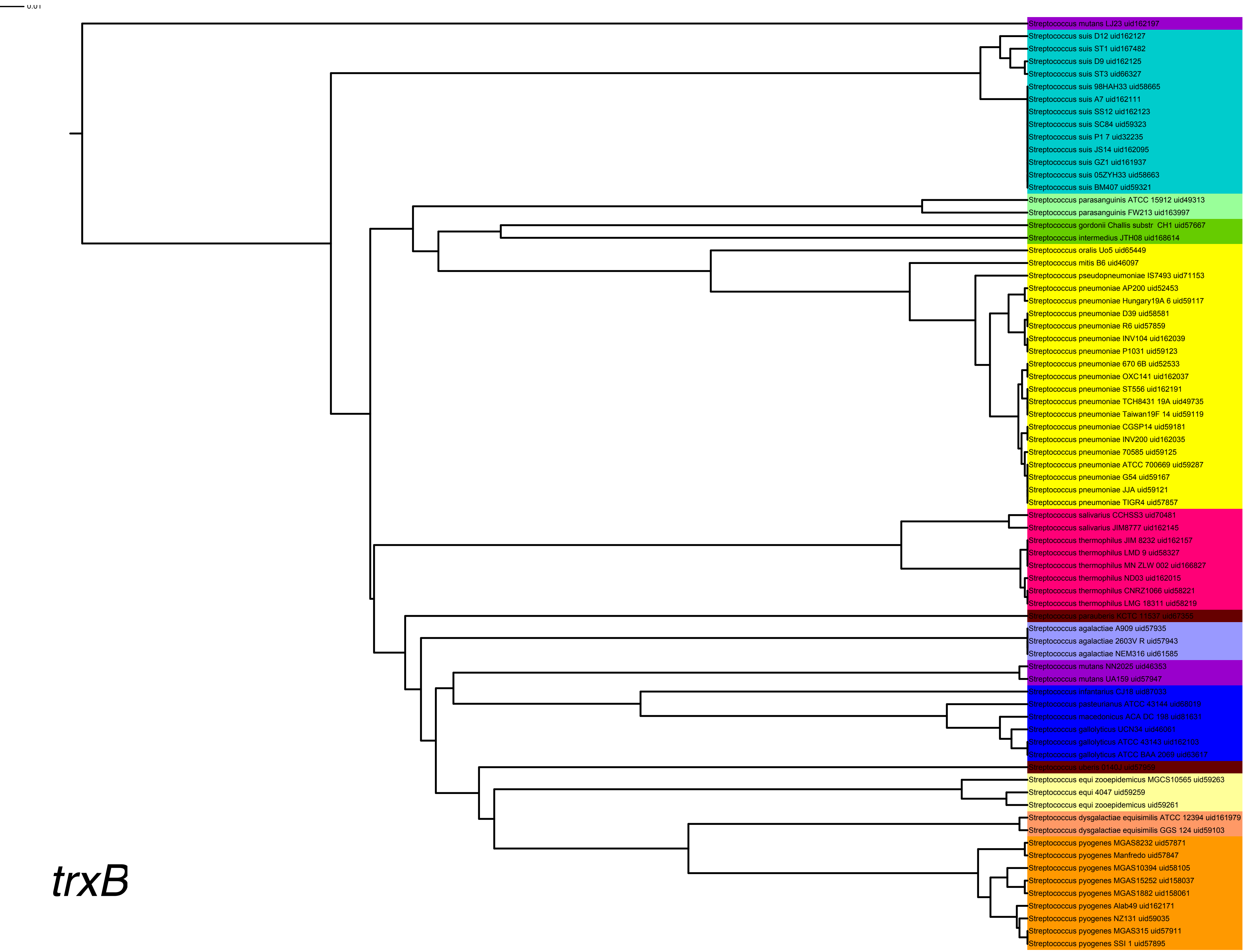
0.01
Streptococcus mutans LJ23 uid162197
Streptococcus suis D12 uid162127
Streptococcus suis ST1 uid167482
Streptococcus suis D9 uid162125
Streptococcus suis ST3 uid66327
Streptococcus suis 98HAH33 uid58665
Streptococcus suis A7 uid162111
Streptococcus suis SS12 uid162123
Streptococcus suis SC84 uid59323
Streptococcus suis P1 7 uid32235
Streptococcus suis JS14 uid162095
Streptococcus suis GZ1 uid161937
Streptococcus suis 05ZYH33 uid58663
Streptococcus suis BM407 uid59321
Streptococcus parasanguinis ATCC 15912 uid49313
Streptococcus parasanguinis FW213 uid163997
Streptococcus gordonii Challis substr CH1 uid57667
Streptococcus intermedius JTH08 uid168614
Streptococcus oralis Uo5 uid65449
Streptococcus mitis B6 uid46097
Streptococcus pseudopneumoniae IS7493 uid71153
Streptococcus pneumoniae AP200 uid52453
Streptococcus pneumoniae Hungary19A 6 uid59117
Streptococcus pneumoniae D39 uid58581
Streptococcus pneumoniae R6 uid57859
Streptococcus pneumoniae INV104 uid162039
Streptococcus pneumoniae P1031 uid59123
Streptococcus pneumoniae 670 6B uid52533
Streptococcus pneumoniae OXC141 uid162037
Streptococcus pneumoniae ST556 uid162191
Streptococcus pneumoniae TCH8431 19A uid49735
Streptococcus pneumoniae Taiwan19F 14 uid59119
Streptococcus pneumoniae CGSP14 uid59181
Streptococcus pneumoniae INV200 uid162035
Streptococcus pneumoniae 70585 uid59125
Streptococcus pneumoniae ATCC 700669 uid59287
Streptococcus pneumoniae G54 uid59167
Streptococcus pneumoniae JJA uid59121
Streptococcus pneumoniae TIGR4 uid57857
Streptococcus salivarius CCHSS3 uid70481
Streptococcus salivarius JIM8777 uid162145
Streptococcus thermophilus JIM 8232 uid162157
Streptococcus thermophilus LMD 9 uid58327
Streptococcus thermophilus MN ZLW 002 uid166827
Streptococcus thermophilus ND03 uid162015
Streptococcus thermophilus CNRZ1066 uid58221
Streptococcus thermophilus LMG 18311 uid58219
Streptococcus parauberis KCTC 11537 uid67355
Streptococcus agalactiae A909 uid57935
Streptococcus agalactiae 2603V R uid57943
Streptococcus agalactiae NEM316 uid61585
Streptococcus mutans NN2025 uid46353
Streptococcus mutans UA159 uid57947
Streptococcus infantarius CJ18 uid87033
Streptococcus pasteurianus ATCC 43144 uid68019
Streptococcus macedonicus ACA DC 198 uid81631
Streptococcus gallolyticus UCN34 uid46061
Streptococcus gallolyticus ATCC 43143 uid162103
Streptococcus gallolyticus ATCC BAA 2069 uid63617
Streptococcus uberis 0140J uid57959
Streptococcus equi zooepidemicus MGCS10565 uid59263
Streptococcus equi 4047 uid59259
Streptococcus equi zooepidemicus uid59261
Streptococcus dysgalactiae equisimilis ATCC 12394 uid161979
Streptococcus dysgalactiae equisimilis GGS 124 uid59103
Streptococcus pyogenes MGAS8232 uid57871
Streptococcus pyogenes Manfredo uid57847
Streptococcus pyogenes MGAS10394 uid58105
Streptococcus pyogenes MGAS15252 uid158037
Streptococcus pyogenes MGAS1882 uid158061
Streptococcus pyogenes Alab49 uid162171
Streptococcus pyogenes NZ131 uid59035
Streptococcus pyogenes MGAS315 uid57911
Streptococcus pyogenes SSI 1 uid57895
trxB

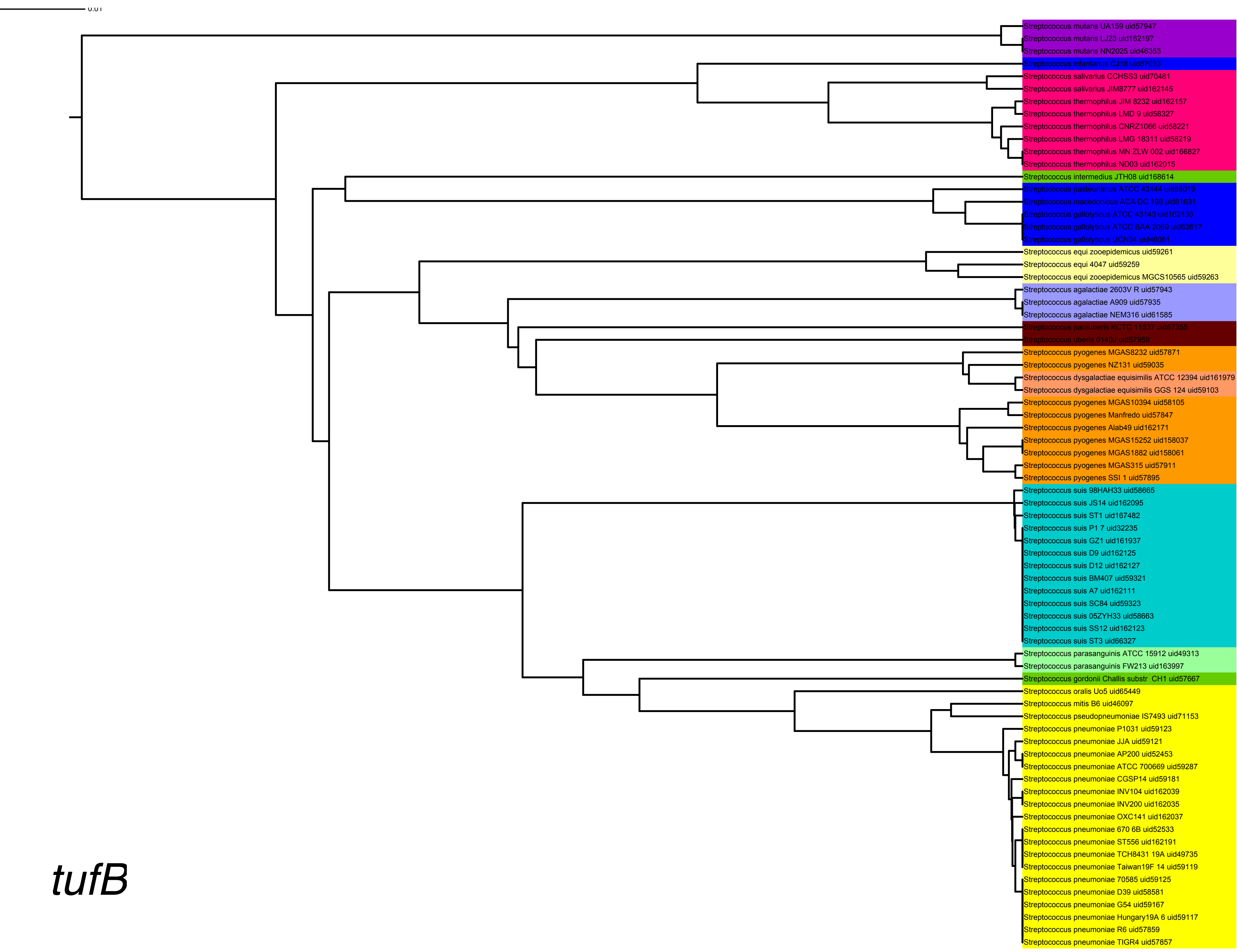
0.01
Streptococcus mutans UA159 uid57947
Streptococcus mutans LJ23 uid162197
Streptococcus mutans NN2025 uid46353
Streptococcus infantarius CJ18 uid87033
Streptococcus salivarius CCHSS3 uid70481
Streptococcus salivarius JIM8777 uid162145
Streptococcus thermophilus JIM 8232 uid162157
Streptococcus thermophilus LMD 9 uid58327
Streptococcus thermophilus CNRZ1066 uid58221
Streptococcus thermophilus LMG 18311 uid58219
Streptococcus thermophilus MN ZLW 002 uid166827
Streptococcus thermophilus ND03 uid162015
Streptococcus intermedius JTH08 uid168614
Streptococcus pasteurianus ATCC 43144 uid68019
Streptococcus macedonicus ACA DC 198 uid81631
Streptococcus gallolyticus ATCC 43143 uid162103
Streptococcus gallolyticus ATCC BAA 2069 uid63617
Streptococcus gallolyticus UCN34 uid46061
Streptococcus equi zooepidemicus uid59261
Streptococcus equi 4047 uid59259
Streptococcus equi zooepidemicus MGCS10565 uid59263
Streptococcus agalactiae 2603V R uid57943
Streptococcus agalactiae A909 uid57935
Streptococcus agalactiae NEM316 uid61585
Streptococcus parauberis KCTC 11537 uid67355
Streptococcus uberis 0140J uid57959
Streptococcus pyogenes MGAS8232 uid57871
Streptococcus pyogenes NZ131 uid59035
Streptococcus dysgalactiae equisimilis ATCC 12394 uid161979
Streptococcus dysgalactiae equisimilis GGS 124 uid59103
Streptococcus pyogenes MGAS10394 uid58105
Streptococcus pyogenes Manfredo uid57847
Streptococcus pyogenes Alab49 uid162171
Streptococcus pyogenes MGAS15252 uid158037
Streptococcus pyogenes MGAS1882 uid158061
Streptococcus pyogenes MGAS315 uid57911
Streptococcus pyogenes SSI 1 uid57895
Streptococcus suis 98HAH33 uid58665
Streptococcus suis JS14 uid162095
Streptococcus suis ST1 uid167482
Streptococcus suis P1 7 uid32235
Streptococcus suis GZ1 uid161937
Streptococcus suis D9 uid162125
Streptococcus suis D12 uid162127
Streptococcus suis BM407 uid59321
Streptococcus suis A7 uid162111
Streptococcus suis SC84 uid59323
Streptococcus suis 05ZYH33 uid58663
Streptococcus suis SS12 uid162123
Streptococcus suis ST3 uid66327
Streptococcus parasanguinis ATCC 15912 uid49313
Streptococcus parasanguinis FW213 uid163997
Streptococcus gordonii Challis substr CH1 uid57667
Streptococcus oralis Uo5 uid65449
Streptococcus mitis B6 uid46097
Streptococcus pseudopneumoniae IS7493 uid71153
Streptococcus pneumoniae P1031 uid59123
Streptococcus pneumoniae JJA uid59121
Streptococcus pneumoniae AP200 uid52453
Streptococcus pneumoniae ATCC 700669 uid59287
Streptococcus pneumoniae CGSP14 uid59181
Streptococcus pneumoniae INV104 uid162039
Streptococcus pneumoniae INV200 uid162035
Streptococcus pneumoniae OXC141 uid162037
Streptococcus pneumoniae 670 6B uid52533
Streptococcus pneumoniae ST556 uid162191
Streptococcus pneumoniae TCH8431 19A uid49735
Streptococcus pneumoniae Taiwan19F 14 uid59119
Streptococcus pneumoniae 70585 uid59125
Streptococcus pneumoniae D39 uid58581
Streptococcus pneumoniae G54 uid59167
Streptococcus pneumoniae Hungary19A 6 uid59117
Streptococcus pneumoniae R6 uid57859
Streptococcus pneumoniae TIGR4 uid57857
tufB

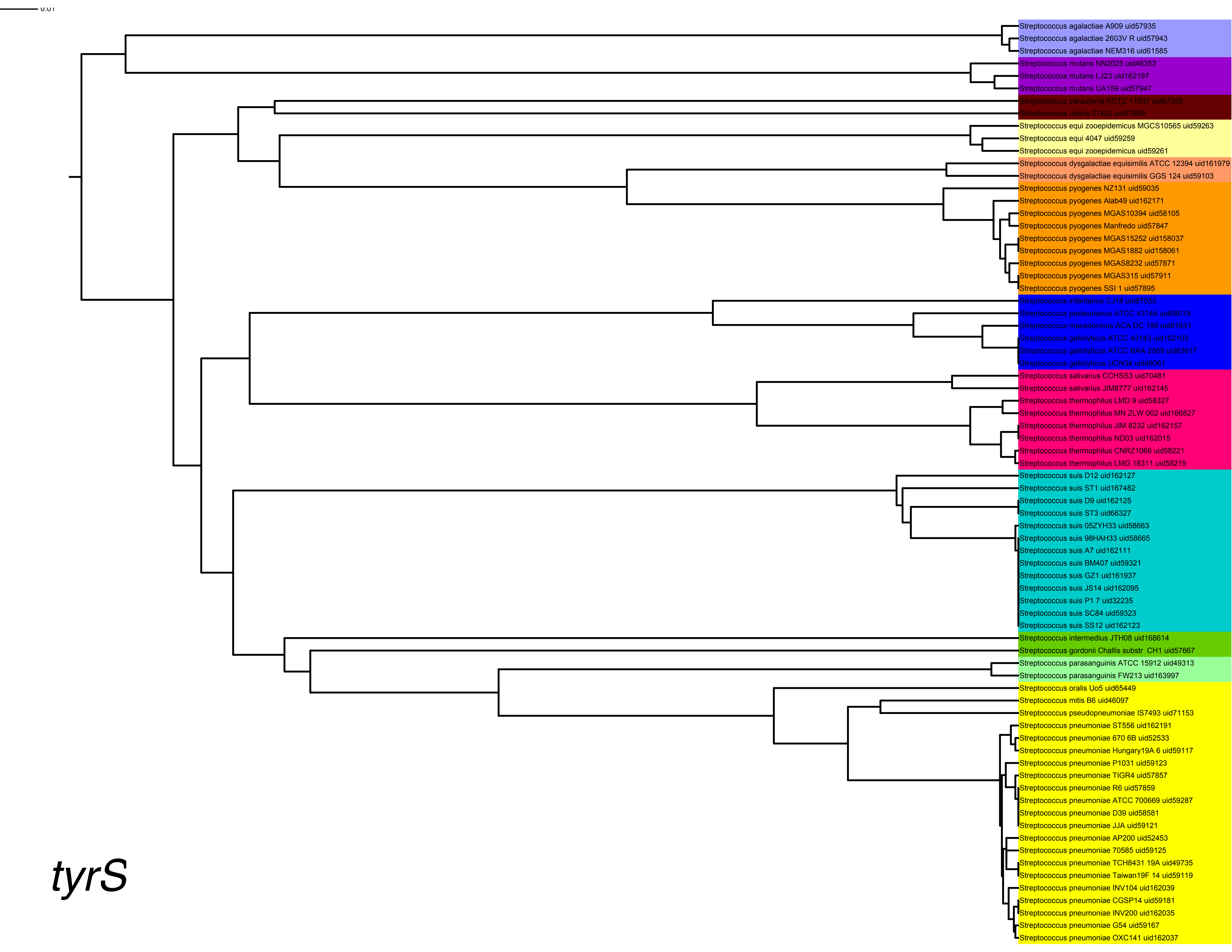

0.01
Streptococcus agalactiae A909 uid57935
Streptococcus agalactiae 2603V R uid57943
Streptococcus agalactiae NEM316 uid61585
Streptococcus mutans NN2025 uid46353
Streptococcus mutans LJ23 uid162197
Streptococcus mutans UA159 uid57947
Streptococcus parauberis KCTC 11537 uid67355
Streptococcus uberis 0140J uid57959
Streptococcus equi zooepidemicus MGCS10565 uid59263
Streptococcus equi 4047 uid59259
Streptococcus equi zooepidemicus uid59261
Streptococcus dysgalactiae equisimilis ATCC 12394 uid161979
Streptococcus dysgalactiae equisimilis GGS 124 uid59103
Streptococcus pyogenes NZ131 uid59035
Streptococcus pyogenes Alab49 uid162171
Streptococcus pyogenes MGAS10394 uid58105
Streptococcus pyogenes Manfredo uid57847
Streptococcus pyogenes MGAS15252 uid158037
Streptococcus pyogenes MGAS1882 uid158061
Streptococcus pyogenes MGAS8232 uid57871
Streptococcus pyogenes MGAS315 uid57911
Streptococcus pyogenes SSI 1 uid57895
Streptococcus infantarius CJ18 uid87033
Streptococcus pasteurianus ATCC 43144 uid68019
Streptococcus macedonicus ACA DC 198 uid81631
Streptococcus gallolyticus ATCC 43143 uid162103
Streptococcus gallolyticus ATCC BAA 2069 uid63617
Streptococcus gallolyticus UCN34 uid46061
Streptococcus salivarius CCHSS3 uid70481
Streptococcus salivarius JIM8777 uid162145
Streptococcus thermophilus LMD 9 uid58327
Streptococcus thermophilus MN ZLW 002 uid166827
Streptococcus thermophilus JIM 8232 uid162157
Streptococcus thermophilus ND03 uid162015
Streptococcus thermophilus CNRZ1066 uid58221
Streptococcus thermophilus LMG 18311 uid58219
Streptococcus suis D12 uid162127
Streptococcus suis ST1 uid167482
Streptococcus suis D9 uid162125
Streptococcus suis ST3 uid66327
Streptococcus suis 05ZYH33 uid58663
Streptococcus suis 98HAH33 uid58665
Streptococcus suis A7 uid162111
Streptococcus suis BM407 uid59321
Streptococcus suis GZ1 uid161937
Streptococcus suis JS14 uid162095
Streptococcus suis P1 7 uid32235
Streptococcus suis SC84 uid59323
Streptococcus suis SS12 uid162123
Streptococcus intermedius JTH08 uid168614
Streptococcus gordonii Challis substr CH1 uid57667
Streptococcus parasanguinis ATCC 15912 uid49313
Streptococcus parasanguinis FW213 uid163997
Streptococcus oralis Uo5 uid65449
Streptococcus mitis B6 uid46097
Streptococcus pseudopneumoniae IS7493 uid71153
Streptococcus pneumoniae ST556 uid162191
Streptococcus pneumoniae 670 6B uid52533
Streptococcus pneumoniae Hungary19A 6 uid59117
Streptococcus pneumoniae P1031 uid59123
Streptococcus pneumoniae TIGR4 uid57857
Streptococcus pneumoniae R6 uid57859
Streptococcus pneumoniae ATCC 700669 uid59287
Streptococcus pneumoniae D39 uid58581
Streptococcus pneumoniae JJA uid59121
Streptococcus pneumoniae AP200 uid52453
Streptococcus pneumoniae 70585 uid59125
Streptococcus pneumoniae TCH8431 19A uid49735
Streptococcus pneumoniae Taiwan19F 14 uid59119
Streptococcus pneumoniae INV104 uid162039
Streptococcus pneumoniae CGSP14 uid59181
Streptococcus pneumoniae INV200 uid162035
Streptococcus pneumoniae G54 uid59167
Streptococcus pneumoniae OXC141 uid162037
tyrS

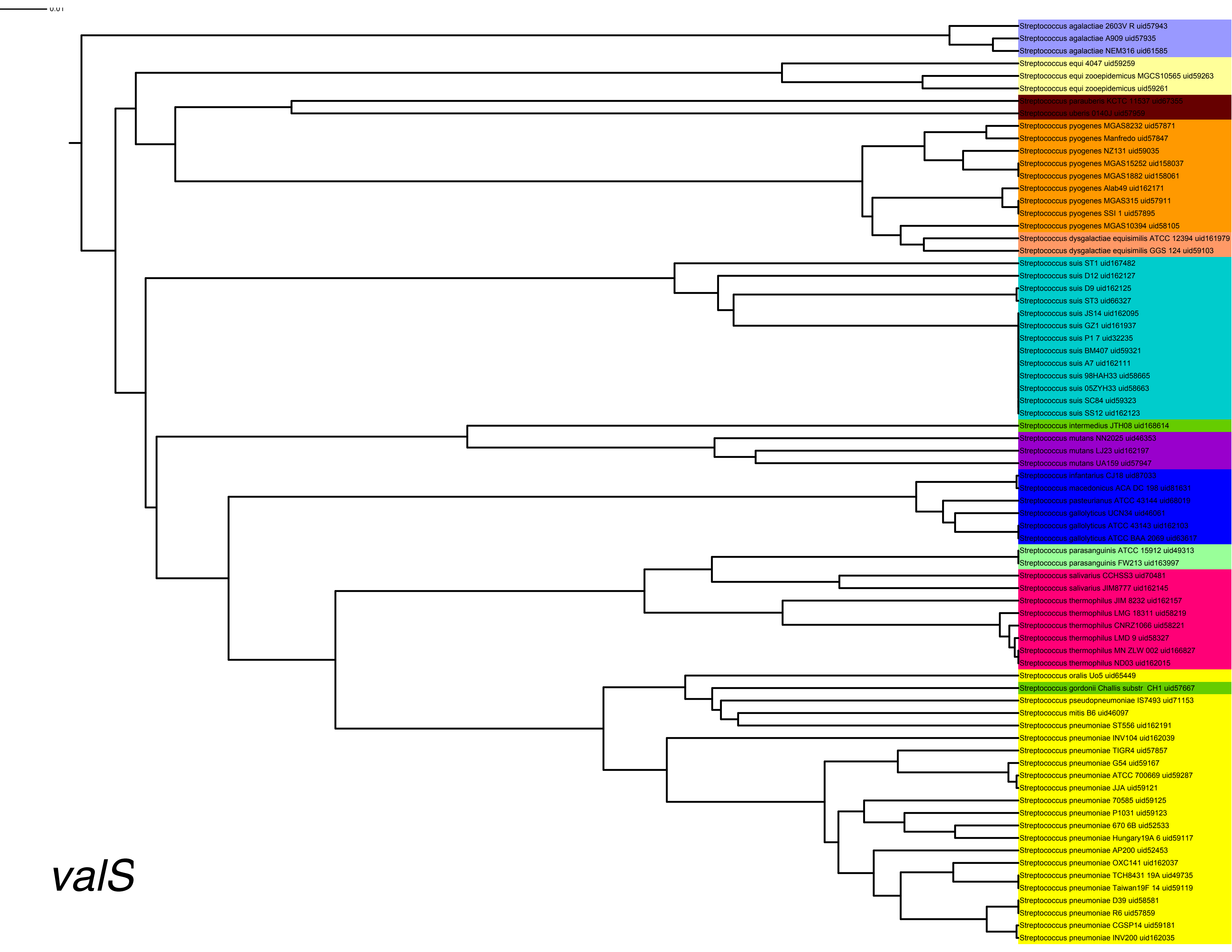
0.01
Streptococcus agalactiae 2603V R uid57943
Streptococcus agalactiae A909 uid57935
Streptococcus agalactiae NEM316 uid61585
Streptococcus equi 4047 uid59259
Streptococcus equi zooepidemicus MGCS10565 uid59263
Streptococcus equi zooepidemicus uid59261
Streptococcus parauberis KCTC 11537 uid67355
Streptococcus uberis 0140J uid57959
Streptococcus pyogenes MGAS8232 uid57871
Streptococcus pyogenes Manfredo uid57847
Streptococcus pyogenes NZ131 uid59035
Streptococcus pyogenes MGAS15252 uid158037
Streptococcus pyogenes MGAS1882 uid158061
Streptococcus pyogenes Alab49 uid162171
Streptococcus pyogenes MGAS315 uid57911
Streptococcus pyogenes SSI 1 uid57895
Streptococcus pyogenes MGAS10394 uid58105
Streptococcus dysgalactiae equisimilis ATCC 12394 uid161979
Streptococcus dysgalactiae equisimilis GGS 124 uid59103
Streptococcus suis ST1 uid167482
Streptococcus suis D12 uid162127
Streptococcus suis D9 uid162125
Streptococcus suis ST3 uid66327
Streptococcus suis JS14 uid162095
Streptococcus suis GZ1 uid161937
Streptococcus suis P1 7 uid32235
Streptococcus suis BM407 uid59321
Streptococcus suis A7 uid162111
Streptococcus suis 98HAH33 uid58665
Streptococcus suis 05ZYH33 uid58663
Streptococcus suis SC84 uid59323
Streptococcus suis SS12 uid162123
Streptococcus intermedius JTH08 uid168614
Streptococcus mutans NN2025 uid46353
Streptococcus mutans LJ23 uid162197
Streptococcus mutans UA159 uid57947
Streptococcus infantarius CJ18 uid87033
Streptococcus macedonicus ACA DC 198 uid81631
Streptococcus pasteurianus ATCC 43144 uid68019
Streptococcus gallolyticus UCN34 uid46061
Streptococcus gallolyticus ATCC 43143 uid162103
Streptococcus gallolyticus ATCC BAA 2069 uid63617
Streptococcus parasanguinis ATCC 15912 uid49313
Streptococcus parasanguinis FW213 uid163997
Streptococcus salivarius CCHSS3 uid70481
Streptococcus salivarius JIM8777 uid162145
Streptococcus thermophilus JIM 8232 uid162157
Streptococcus thermophilus LMG 18311 uid58219
Streptococcus thermophilus CNRZ1066 uid58221
Streptococcus thermophilus LMD 9 uid58327
Streptococcus thermophilus MN ZLW 002 uid166827
Streptococcus thermophilus ND03 uid162015
Streptococcus oralis Uo5 uid65449
Streptococcus gordonii Challis substr CH1 uid57667
Streptococcus pseudopneumoniae IS7493 uid71153
Streptococcus mitis B6 uid46097
Streptococcus pneumoniae ST556 uid162191
Streptococcus pneumoniae INV104 uid162039
Streptococcus pneumoniae TIGR4 uid57857
Streptococcus pneumoniae G54 uid59167
Streptococcus pneumoniae ATCC 700669 uid59287
Streptococcus pneumoniae JJA uid59121
Streptococcus pneumoniae 70585 uid59125
Streptococcus pneumoniae P1031 uid59123
Streptococcus pneumoniae 670 6B uid52533
Streptococcus pneumoniae Hungary19A 6 uid59117
Streptococcus pneumoniae AP200 uid52453
Streptococcus pneumoniae OXC141 uid162037
Streptococcus pneumoniae TCH8431 19A uid49735
Streptococcus pneumoniae Taiwan19F 14 uid59119
Streptococcus pneumoniae D39 uid58581
Streptococcus pneumoniae R6 uid57859
Streptococcus pneumoniae CGSP14 uid59181
Streptococcus pneumoniae INV200 uid162035
valS
