# Supplementary material for: Marker genes that are less conserved in their sequences are useful for predicting genome-wide similarity levels between closely related prokaryotic strains
Source: Microbiome. 2016 May 3;4:18. doi: 10.1186/s40168-016-0162-5 (PMC4853863; doi:10.1186/s40168-016-0162-5)
Supplement: Additional file 1: Figure S1. — The number of genomes in which each marker gene is identified. Out of the 79 potential marker genes, 73 are present in at least 90 % of the genomes. Figure S2. Spearman’s correlation between each marker gene and the average AAI for all complete genomes. Genes are ordered in the same way as in Fig. 3. Figure S3. Trees generated based on AAI and on percent identities of each marker gene (including 16s rRNA), for the Escherichia/Shigella clade. Figure S4. Trees generated based on AAI and on percent identities of each marker gene (including 16s rRNA), for the Streptococcus clade. Figure S5. Trees generated based on AAI and on percent identities of each marker gene (including 16s rRNA), for the Bacillus clade. Table S1. List of 79 potential marker genes surveyed, out of which 73 were found to be present in at least 90 % of the genomes. Table S2. Alternative names of 79 potential marker genes surveyed. Table S3. Split distances between UPGMA tree generated using AAI and that generated using the percent identities of each marker gene, shown in correspondence with the average percent identity ranks of the marker genes. Table S4. Designed primers for each of the 10 genes that were least conserved in their sequences in the Escherichia/Shigella lineage. (ZIP 3355 kb) [file 40168_2016_162_MOESM1_ESM.zip › FigureS5.pdf]

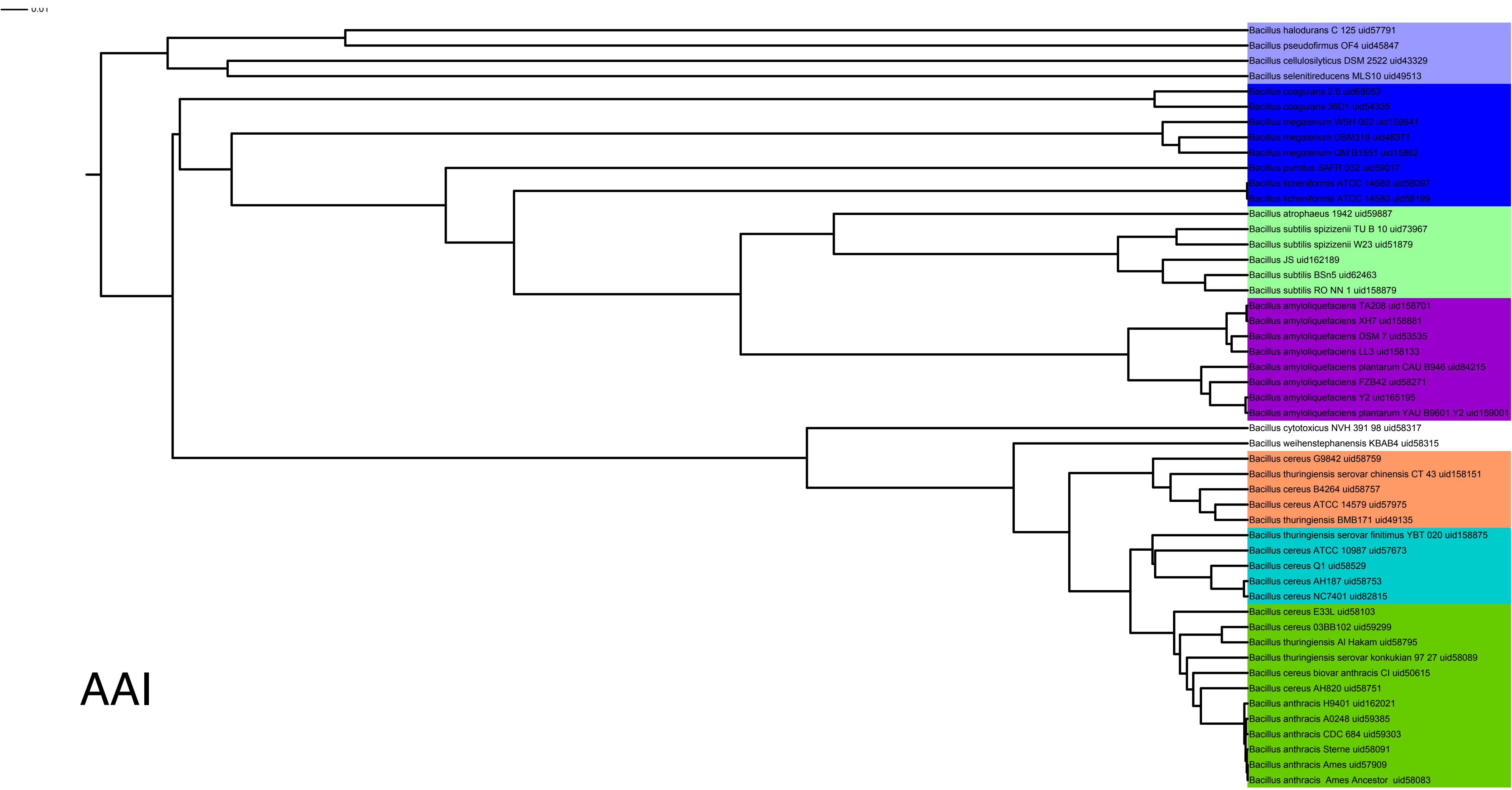
0.01
Bacillus halodurans C 125 uid57791
Bacillus pseudofirmus OF4 uid45847
Bacillus cellulosilyticus DSM 2522 uid43329
Bacillus selenitireducens MLS10 uid49513
Bacillus coagulans 2 6 uid68053
Bacillus coagulans 36D1 uid54335
Bacillus megaterium WSH 002 uid159841
Bacillus megaterium DSM319 uid48371
Bacillus megaterium QM B1551 uid15862
Bacillus pumilus SAFR 032 uid59017
Bacillus licheniformis ATCC 14580 uid58097
Bacillus licheniformis ATCC 14580 uid58199
Bacillus atrophaeus 1942 uid59887
Bacillus subtilis spizizenii TU B 10 uid73967
Bacillus subtilis spizizenii W23 uid51879
Bacillus JS uid162189
Bacillus subtilis BSn5 uid62463
Bacillus subtilis RO NN 1 uid158879
Bacillus amyloliquefaciens TA208 uid158701
Bacillus amyloliquefaciens XH7 uid158881
Bacillus amyloliquefaciens DSM 7 uid53535
Bacillus amyloliquefaciens LL3 uid158133
Bacillus amyloliquefaciens plantarum CAU B946 uid84215
Bacillus amyloliquefaciens FZB42 uid58271
Bacillus amyloliquefaciens Y2 uid165195
Bacillus amyloliquefaciens plantarum YAU B9601 Y2 uid159001
Bacillus cytotoxicus NVH 391 98 uid58317
Bacillus weihenstephanensis KBAB4 uid58315
Bacillus cereus G9842 uid58759
Bacillus thuringiensis serovar chinensis CT 43 uid158151
Bacillus cereus B4264 uid58757
Bacillus cereus ATCC 14579 uid57975
Bacillus thuringiensis BMB171 uid49135
Bacillus thuringiensis serovar finitimus YBT 020 uid158875
Bacillus cereus ATCC 10987 uid57673
Bacillus cereus Q1 uid58529
Bacillus cereus AH187 uid58753
Bacillus cereus NC7401 uid82815
Bacillus cereus E33L uid58103
Bacillus cereus 03BB102 uid59299
Bacillus thuringiensis Al Hakam uid58795
Bacillus thuringiensis serovar konkukian 97 27 uid58089
Bacillus cereus biovar anthracis CI uid50615
Bacillus cereus AH820 uid58751
Bacillus anthracis H9401 uid162021
Bacillus anthracis A0248 uid59385
Bacillus anthracis CDC 684 uid59303
Bacillus anthracis Sterne uid58091
Bacillus anthracis Ames uid57909
Bacillus anthracis Ames Ancestor uid58083
AAI

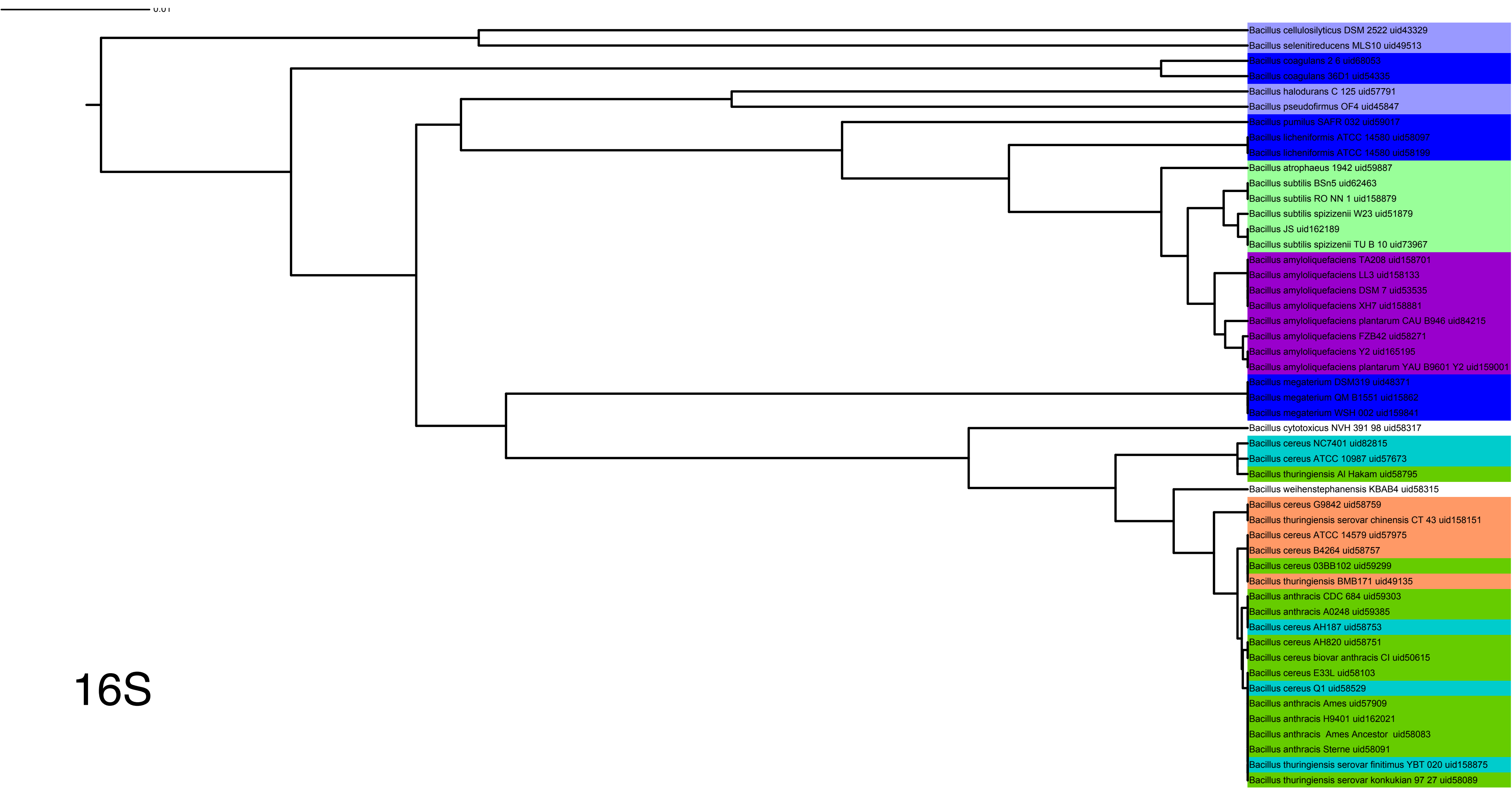
0.01
Bacillus cellulosilyticus DSM 2522 uid43329
Bacillus selenitireducens MLS10 uid49513
Bacillus coagulans 2 6 uid68053
Bacillus coagulans 36D1 uid54335
Bacillus halodurans C 125 uid57791
Bacillus pseudofirmus OF4 uid45847
Bacillus pumilus SAFR 032 uid59017
Bacillus licheniformis ATCC 14580 uid58097
Bacillus licheniformis ATCC 14580 uid58199
Bacillus atrophaeus 1942 uid59887
Bacillus subtilis BSn5 uid62463
Bacillus subtilis RO NN 1 uid158879
Bacillus subtilis spizizenii W23 uid51879
Bacillus JS uid162189
Bacillus subtilis spizizenii TU B 10 uid73967
Bacillus amyloliquefaciens TA208 uid158701
Bacillus amyloliquefaciens LL3 uid158133
Bacillus amyloliquefaciens DSM 7 uid53535
Bacillus amyloliquefaciens XH7 uid158881
Bacillus amyloliquefaciens plantarum CAU B946 uid84215
Bacillus amyloliquefaciens FZB42 uid58271
Bacillus amyloliquefaciens Y2 uid165195
Bacillus amyloliquefaciens plantarum YAU B9601 Y2 uid159001
Bacillus megaterium DSM319 uid48371
Bacillus megaterium QM B1551 uid15862
Bacillus megaterium WSH 002 uid159841
Bacillus cytotoxicus NVH 391 98 uid58317
Bacillus cereus NC7401 uid82815
Bacillus cereus ATCC 10987 uid57673
Bacillus thuringiensis Al Hakam uid58795
Bacillus weihenstephanensis KBAB4 uid58315
Bacillus cereus G9842 uid58759
Bacillus thuringiensis serovar chinensis CT 43 uid158151
Bacillus cereus ATCC 14579 uid57975
Bacillus cereus B4264 uid58757
Bacillus cereus 03BB102 uid59299
Bacillus thuringiensis BMB171 uid49135
Bacillus anthracis CDC 684 uid59303
Bacillus anthracis A0248 uid59385
Bacillus cereus AH187 uid58753
Bacillus cereus AH820 uid58751
Bacillus cereus biovar anthracis CI uid50615
Bacillus cereus E33L uid58103
Bacillus cereus Q1 uid58529
Bacillus anthracis Ames uid57909
Bacillus anthracis H9401 uid162021
Bacillus anthracis  Ames Ancestor  uid58083
Bacillus anthracis Sterne uid58091
Bacillus thuringiensis serovar finitimus YBT 020 uid158875
Bacillus thuringiensis serovar konkukian 97 27 uid58089
16S

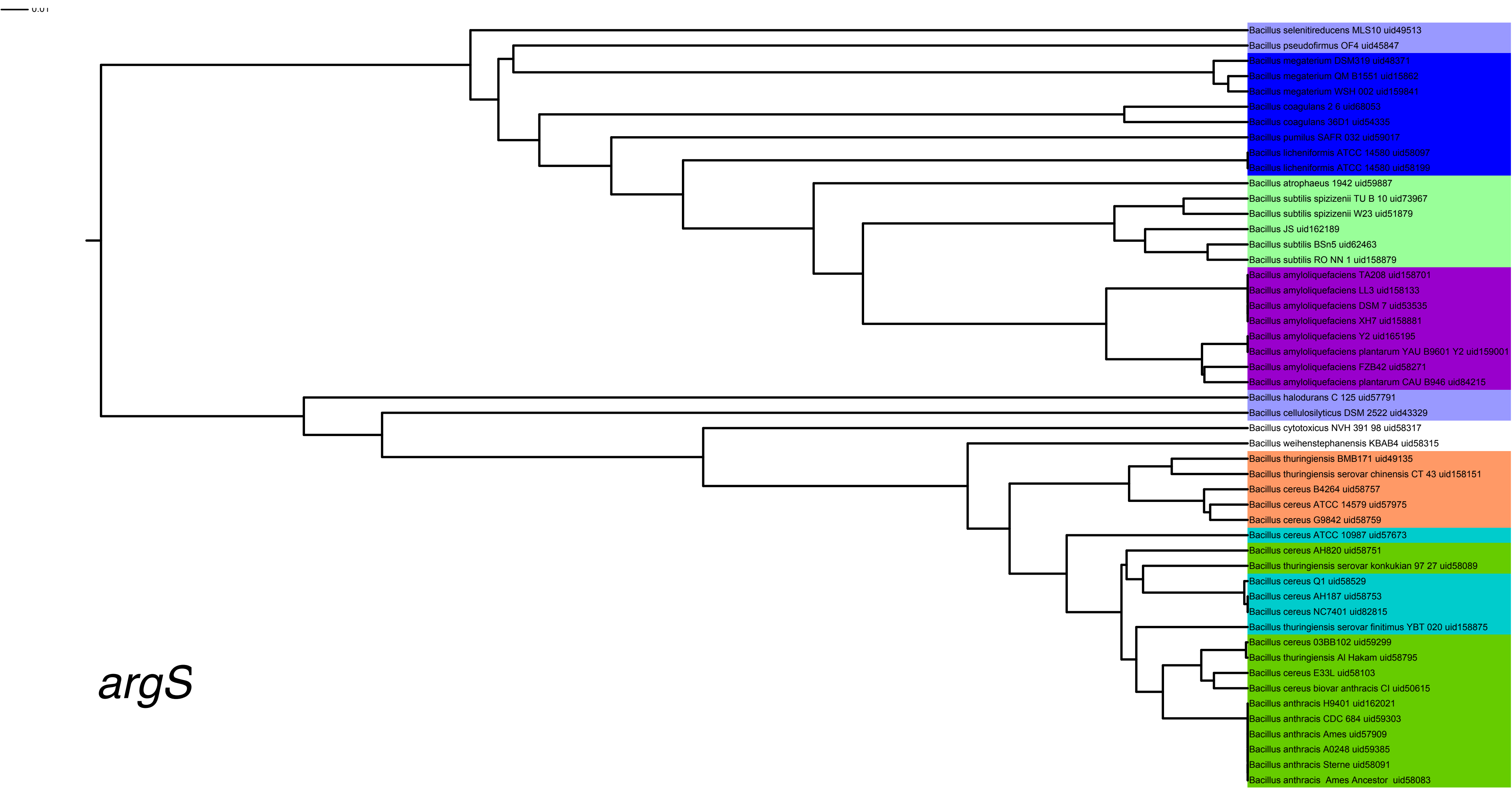
0.01
Bacillus selenitireducens MLS10 uid49513
Bacillus pseudofirmus OF4 uid45847
Bacillus megaterium DSM319 uid48371
Bacillus megaterium QM B1551 uid15862
Bacillus megaterium WSH 002 uid159841
Bacillus coagulans 2 6 uid68053
Bacillus coagulans 36D1 uid54335
Bacillus pumilus SAFR 032 uid59017
Bacillus licheniformis ATCC 14580 uid58097
Bacillus licheniformis ATCC 14580 uid58199
Bacillus atrophaeus 1942 uid59887
Bacillus subtilis spizizenii TU B 10 uid73967
Bacillus subtilis spizizenii W23 uid51879
Bacillus JS uid162189
Bacillus subtilis BSn5 uid62463
Bacillus subtilis RO NN 1 uid158879
Bacillus amyloliquefaciens TA208 uid158701
Bacillus amyloliquefaciens LL3 uid158133
Bacillus amyloliquefaciens DSM 7 uid53535
Bacillus amyloliquefaciens XH7 uid158881
Bacillus amyloliquefaciens Y2 uid165195
Bacillus amyloliquefaciens plantarum YAU B9601 Y2 uid159001
Bacillus amyloliquefaciens FZB42 uid58271
Bacillus amyloliquefaciens plantarum CAU B946 uid84215
Bacillus halodurans C 125 uid57791
Bacillus cellulosilyticus DSM 2522 uid43329
Bacillus cytotoxicus NVH 391 98 uid58317
Bacillus weihenstephanensis KBAB4 uid58315
Bacillus thuringiensis BMB171 uid49135
Bacillus thuringiensis serovar chinensis CT 43 uid158151
Bacillus cereus B4264 uid58757
Bacillus cereus ATCC 14579 uid57975
Bacillus cereus G9842 uid58759
Bacillus cereus ATCC 10987 uid57673
Bacillus cereus AH820 uid58751
Bacillus thuringiensis serovar konkukian 97 27 uid58089
Bacillus cereus Q1 uid58529
Bacillus cereus AH187 uid58753
Bacillus cereus NC7401 uid82815
Bacillus thuringiensis serovar finitimus YBT 020 uid158875
Bacillus cereus 03BB102 uid59299
Bacillus thuringiensis Al Hakam uid58795
Bacillus cereus E33L uid58103
Bacillus cereus biovar anthracis CI uid50615
Bacillus anthracis H9401 uid162021
Bacillus anthracis CDC 684 uid59303
Bacillus anthracis Ames uid57909
Bacillus anthracis A0248 uid59385
Bacillus anthracis Sterne uid58091
Bacillus anthracis  Ames Ancestor  uid58083
argS

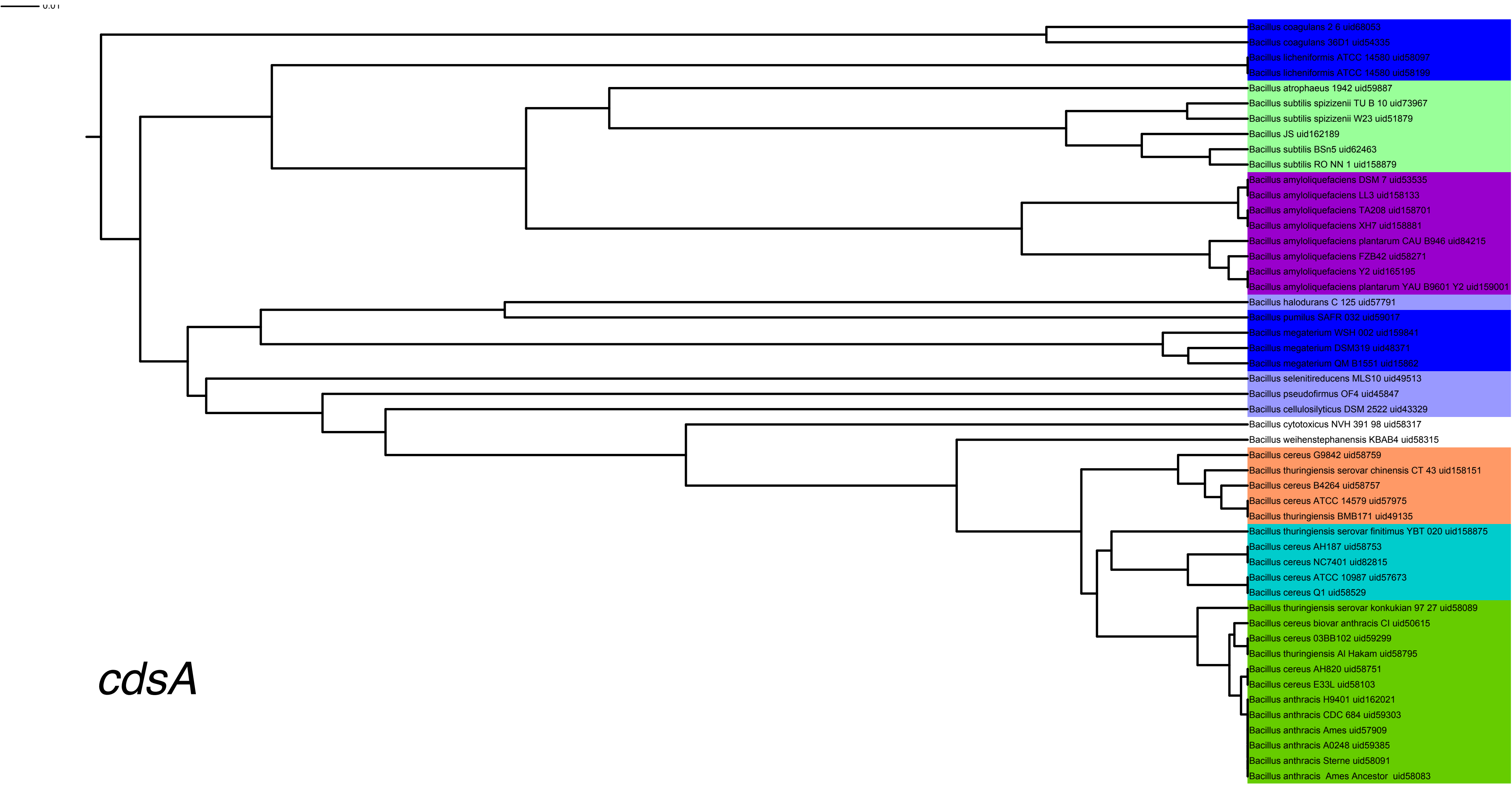
0.01
Bacillus coagulans 2 6 uid68053
Bacillus coagulans 36D1 uid54335
Bacillus licheniformis ATCC 14580 uid58097
Bacillus licheniformis ATCC 14580 uid58199
Bacillus atrophaeus 1942 uid59887
Bacillus subtilis spizizenii TU B 10 uid73967
Bacillus subtilis spizizenii W23 uid51879
Bacillus JS uid162189
Bacillus subtilis BSn5 uid62463
Bacillus subtilis RO NN 1 uid158879
Bacillus amyloliquefaciens DSM 7 uid53535
Bacillus amyloliquefaciens LL3 uid158133
Bacillus amyloliquefaciens TA208 uid158701
Bacillus amyloliquefaciens XH7 uid158881
Bacillus amyloliquefaciens plantarum CAU B946 uid84215
Bacillus amyloliquefaciens FZB42 uid58271
Bacillus amyloliquefaciens Y2 uid165195
Bacillus amyloliquefaciens plantarum YAU B9601 Y2 uid159001
Bacillus halodurans C 125 uid57791
Bacillus pumilus SAFR 032 uid59017
Bacillus megaterium WSH 002 uid159841
Bacillus megaterium DSM319 uid48371
Bacillus megaterium QM B1551 uid15862
Bacillus selenitireducens MLS10 uid49513
Bacillus pseudofirmus OF4 uid45847
Bacillus cellulosilyticus DSM 2522 uid43329
Bacillus cytotoxicus NVH 391 98 uid58317
Bacillus weihenstephanensis KBAB4 uid58315
Bacillus cereus G9842 uid58759
Bacillus thuringiensis serovar chinensis CT 43 uid158151
Bacillus cereus B4264 uid58757
Bacillus cereus ATCC 14579 uid57975
Bacillus thuringiensis BMB171 uid49135
Bacillus thuringiensis serovar finitimus YBT 020 uid158875
Bacillus cereus AH187 uid58753
Bacillus cereus NC7401 uid82815
Bacillus cereus ATCC 10987 uid57673
Bacillus cereus Q1 uid58529
Bacillus thuringiensis serovar konkukian 97 27 uid58089
Bacillus cereus biovar anthracis CI uid50615
Bacillus cereus 03BB102 uid59299
Bacillus thuringiensis Al Hakam uid58795
Bacillus cereus AH820 uid58751
Bacillus cereus E33L uid58103
Bacillus anthracis H9401 uid162021
Bacillus anthracis CDC 684 uid59303
Bacillus anthracis Ames uid57909
Bacillus anthracis A0248 uid59385
Bacillus anthracis Sterne uid58091
Bacillus anthracis Ames Ancestor uid58083
cdsA

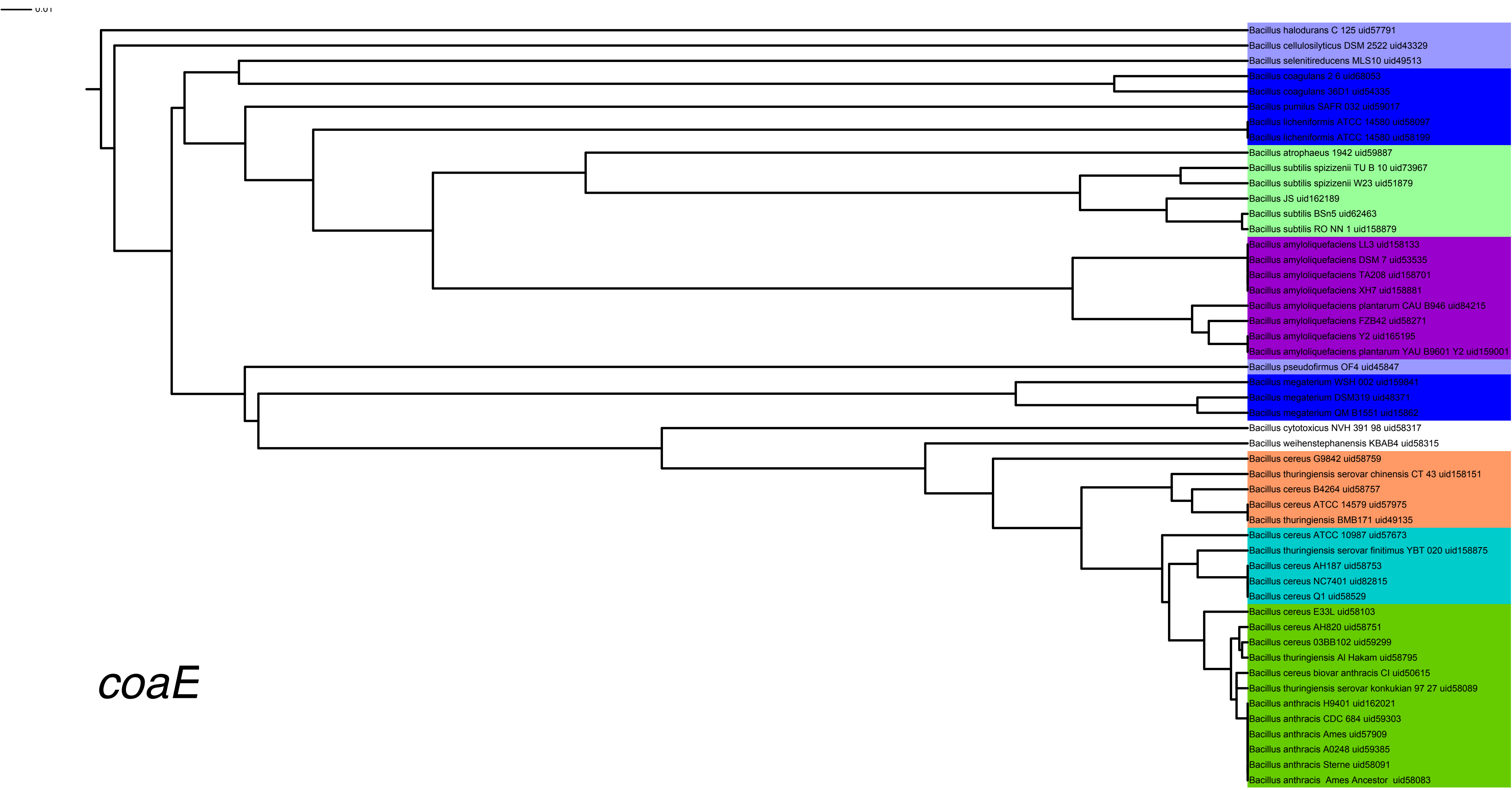
0.01
Bacillus halodurans C 125 uid57791
Bacillus cellulosilyticus DSM 2522 uid43329
Bacillus selenitireducens MLS10 uid49513
Bacillus coagulans 2 6 uid68053
Bacillus coagulans 36D1 uid54335
Bacillus pumilus SAFR 032 uid59017
Bacillus licheniformis ATCC 14580 uid58097
Bacillus licheniformis ATCC 14580 uid58199
Bacillus atrophaeus 1942 uid59887
Bacillus subtilis spizizenii TU B 10 uid73967
Bacillus subtilis spizizenii W23 uid51879
Bacillus JS uid162189
Bacillus subtilis BSn5 uid62463
Bacillus subtilis RO NN 1 uid158879
Bacillus amyloliquefaciens LL3 uid158133
Bacillus amyloliquefaciens DSM 7 uid53535
Bacillus amyloliquefaciens TA208 uid158701
Bacillus amyloliquefaciens XH7 uid158881
Bacillus amyloliquefaciens plantarum CAU B946 uid84215
Bacillus amyloliquefaciens FZB42 uid58271
Bacillus amyloliquefaciens Y2 uid165195
Bacillus amyloliquefaciens plantarum YAU B9601 Y2 uid159001
Bacillus pseudofirmus OF4 uid45847
Bacillus megaterium WSH 002 uid159841
Bacillus megaterium DSM319 uid48371
Bacillus megaterium QM B1551 uid15862
Bacillus cytotoxicus NVH 391 98 uid58317
Bacillus weihenstephanensis KBAB4 uid58315
Bacillus cereus G9842 uid58759
Bacillus thuringiensis serovar chinensis CT 43 uid158151
Bacillus cereus B4264 uid58757
Bacillus cereus ATCC 14579 uid57975
Bacillus thuringiensis BMB171 uid49135
Bacillus cereus ATCC 10987 uid57673
Bacillus thuringiensis serovar finitimus YBT 020 uid158875
Bacillus cereus AH187 uid58753
Bacillus cereus NC7401 uid82815
Bacillus cereus Q1 uid58529
Bacillus cereus E33L uid58103
Bacillus cereus AH820 uid58751
Bacillus cereus 03BB102 uid59299
Bacillus thuringiensis Al Hakam uid58795
Bacillus cereus biovar anthracis CI uid50615
Bacillus thuringiensis serovar konkukian 97 27 uid58089
Bacillus anthracis H9401 uid162021
Bacillus anthracis CDC 684 uid59303
Bacillus anthracis Ames uid57909
Bacillus anthracis A0248 uid59385
Bacillus anthracis Sterne uid58091
Bacillus anthracis  Ames Ancestor  uid58083
coaE

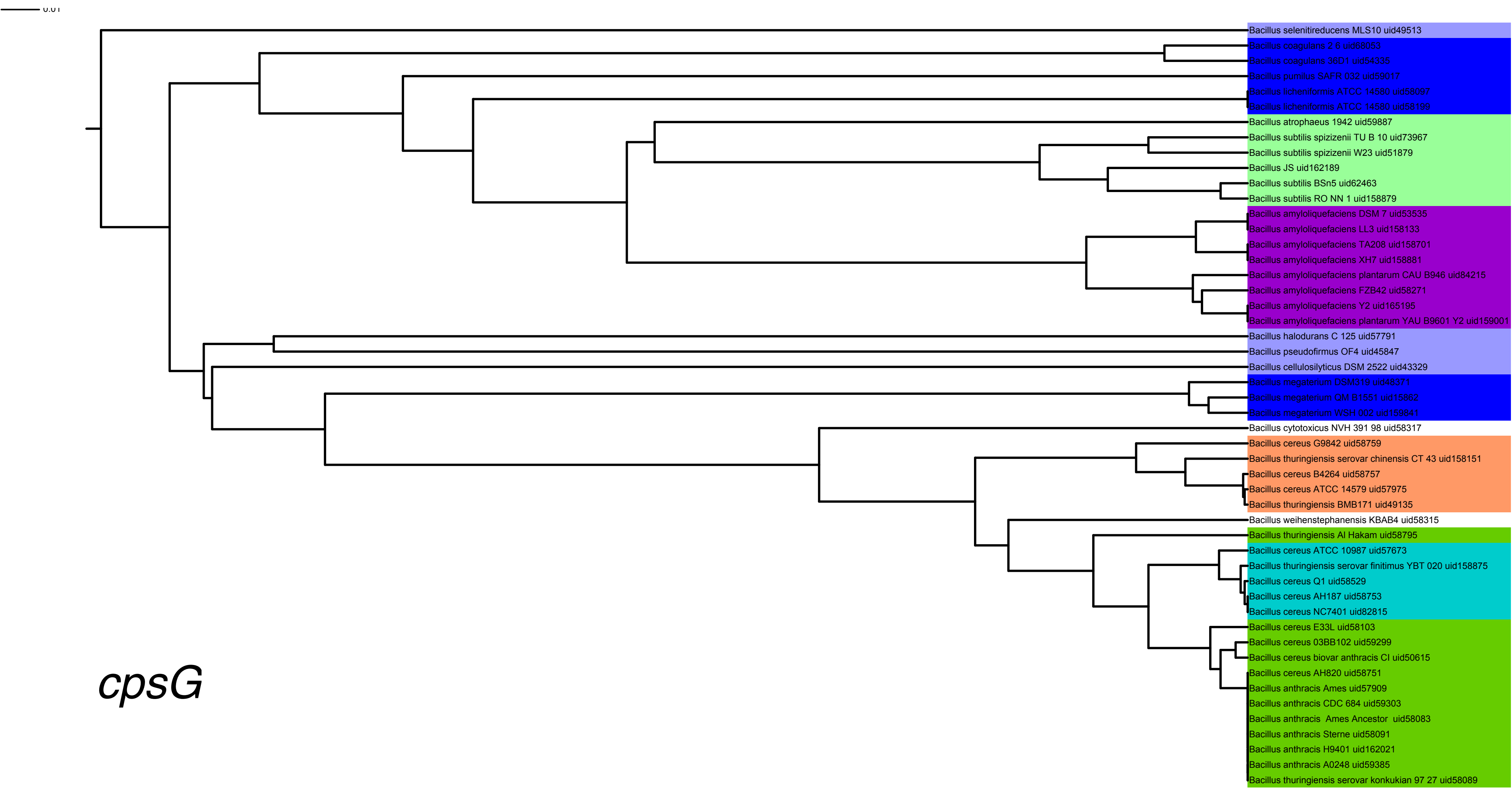
0.01
Bacillus selenitireducens MLS10 uid49513
Bacillus coagulans 2 6 uid68053
Bacillus coagulans 36D1 uid54335
Bacillus pumilus SAFR 032 uid59017
Bacillus licheniformis ATCC 14580 uid58097
Bacillus licheniformis ATCC 14580 uid58199
Bacillus atrophaeus 1942 uid59887
Bacillus subtilis spizizenii TU B 10 uid73967
Bacillus subtilis spizizenii W23 uid51879
Bacillus JS uid162189
Bacillus subtilis BSn5 uid62463
Bacillus subtilis RO NN 1 uid158879
Bacillus amyloliquefaciens DSM 7 uid53535
Bacillus amyloliquefaciens LL3 uid158133
Bacillus amyloliquefaciens TA208 uid158701
Bacillus amyloliquefaciens XH7 uid158881
Bacillus amyloliquefaciens plantarum CAU B946 uid84215
Bacillus amyloliquefaciens FZB42 uid58271
Bacillus amyloliquefaciens Y2 uid165195
Bacillus amyloliquefaciens plantarum YAU B9601 Y2 uid159001
Bacillus halodurans C 125 uid57791
Bacillus pseudofirmus OF4 uid45847
Bacillus cellulosilyticus DSM 2522 uid43329
Bacillus megaterium DSM319 uid48371
Bacillus megaterium QM B1551 uid15862
Bacillus megaterium WSH 002 uid159841
Bacillus cytotoxicus NVH 391 98 uid58317
Bacillus cereus G9842 uid58759
Bacillus thuringiensis serovar chinensis CT 43 uid158151
Bacillus cereus B4264 uid58757
Bacillus cereus ATCC 14579 uid57975
Bacillus thuringiensis BMB171 uid49135
Bacillus weihenstephanensis KBAB4 uid58315
Bacillus thuringiensis Al Hakam uid58795
Bacillus cereus ATCC 10987 uid57673
Bacillus thuringiensis serovar finitimus YBT 020 uid158875
Bacillus cereus Q1 uid58529
Bacillus cereus AH187 uid58753
Bacillus cereus NC7401 uid82815
Bacillus cereus E33L uid58103
Bacillus cereus 03BB102 uid59299
Bacillus cereus biovar anthracis CI uid50615
Bacillus cereus AH820 uid58751
Bacillus anthracis Ames uid57909
Bacillus anthracis CDC 684 uid59303
Bacillus anthracis  Ames Ancestor  uid58083
Bacillus anthracis Sterne uid58091
Bacillus anthracis H9401 uid162021
Bacillus anthracis A0248 uid59385
Bacillus thuringiensis serovar konkukian 97 27 uid58089
cpsG

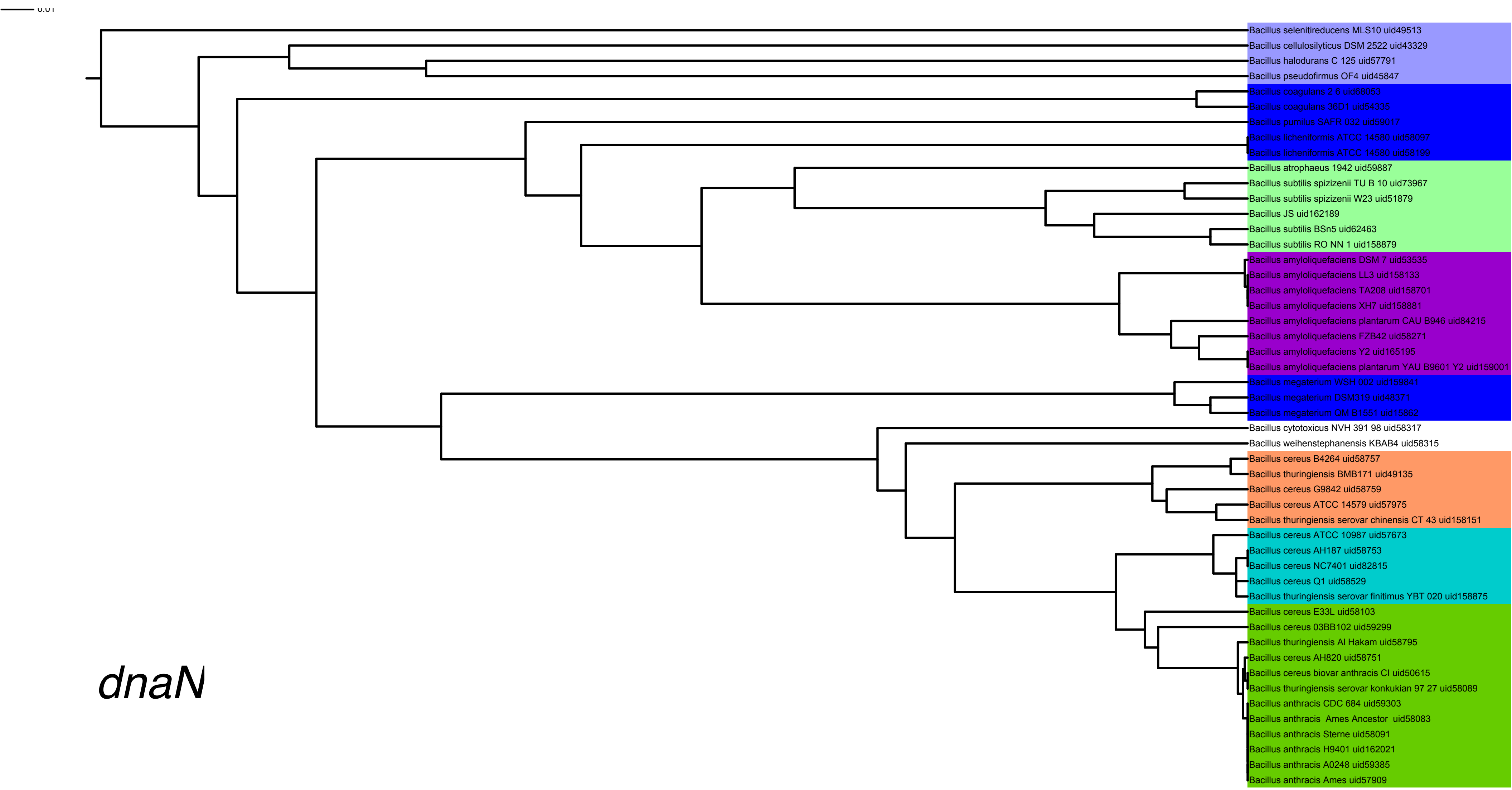
0.01
Bacillus selenitireducens MLS10 uid49513
Bacillus cellulosilyticus DSM 2522 uid43329
Bacillus halodurans C 125 uid57791
Bacillus pseudofirmus OF4 uid45847
Bacillus coagulans 2 6 uid68053
Bacillus coagulans 36D1 uid54335
Bacillus pumilus SAFR 032 uid59017
Bacillus licheniformis ATCC 14580 uid58097
Bacillus licheniformis ATCC 14580 uid58199
Bacillus atrophaeus 1942 uid59887
Bacillus subtilis spizizenii TU B 10 uid73967
Bacillus subtilis spizizenii W23 uid51879
Bacillus JS uid162189
Bacillus subtilis BSn5 uid62463
Bacillus subtilis RO NN 1 uid158879
Bacillus amyloliquefaciens DSM 7 uid53535
Bacillus amyloliquefaciens LL3 uid158133
Bacillus amyloliquefaciens TA208 uid158701
Bacillus amyloliquefaciens XH7 uid158881
Bacillus amyloliquefaciens plantarum CAU B946 uid84215
Bacillus amyloliquefaciens FZB42 uid58271
Bacillus amyloliquefaciens Y2 uid165195
Bacillus amyloliquefaciens plantarum YAU B9601 Y2 uid159001
Bacillus megaterium WSH 002 uid159841
Bacillus megaterium DSM319 uid48371
Bacillus megaterium QM B1551 uid15862
Bacillus cytotoxicus NVH 391 98 uid58317
Bacillus weihenstephanensis KBAB4 uid58315
Bacillus cereus B4264 uid58757
Bacillus thuringiensis BMB171 uid49135
Bacillus cereus G9842 uid58759
Bacillus cereus ATCC 14579 uid57975
Bacillus thuringiensis serovar chinensis CT 43 uid158151
Bacillus cereus ATCC 10987 uid57673
Bacillus cereus AH187 uid58753
Bacillus cereus NC7401 uid82815
Bacillus cereus Q1 uid58529
Bacillus thuringiensis serovar finitimus YBT 020 uid158875
Bacillus cereus E33L uid58103
Bacillus cereus 03BB102 uid59299
Bacillus thuringiensis Al Hakam uid58795
Bacillus cereus AH820 uid58751
Bacillus cereus biovar anthracis CI uid50615
Bacillus thuringiensis serovar konkukian 97 27 uid58089
Bacillus anthracis CDC 684 uid59303
Bacillus anthracis  Ames Ancestor  uid58083
Bacillus anthracis Sterne uid58091
Bacillus anthracis H9401 uid162021
Bacillus anthracis A0248 uid59385
Bacillus anthracis Ames uid57909
dnaN

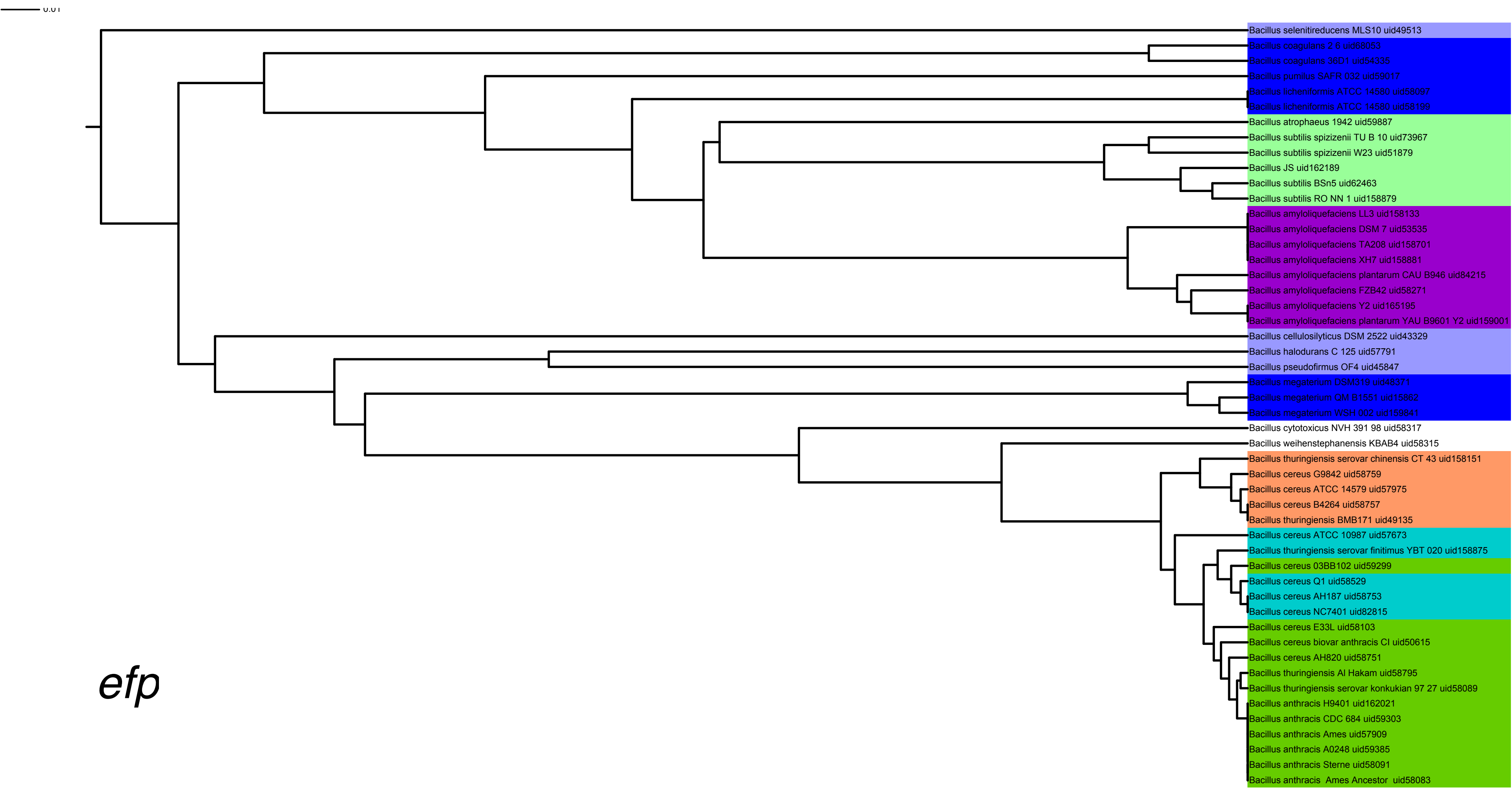
0.01
Bacillus selenitireducens MLS10 uid49513
Bacillus coagulans 2 6 uid68053
Bacillus coagulans 36D1 uid54335
Bacillus pumilus SAFR 032 uid59017
Bacillus licheniformis ATCC 14580 uid58097
Bacillus licheniformis ATCC 14580 uid58199
Bacillus atrophaeus 1942 uid59887
Bacillus subtilis spizizenii TU B 10 uid73967
Bacillus subtilis spizizenii W23 uid51879
Bacillus JS uid162189
Bacillus subtilis BSn5 uid62463
Bacillus subtilis RO NN 1 uid158879
Bacillus amyloliquefaciens LL3 uid158133
Bacillus amyloliquefaciens DSM 7 uid53535
Bacillus amyloliquefaciens TA208 uid158701
Bacillus amyloliquefaciens XH7 uid158881
Bacillus amyloliquefaciens plantarum CAU B946 uid84215
Bacillus amyloliquefaciens FZB42 uid58271
Bacillus amyloliquefaciens Y2 uid165195
Bacillus amyloliquefaciens plantarum YAU B9601 Y2 uid159001
Bacillus cellulosilyticus DSM 2522 uid43329
Bacillus halodurans C 125 uid57791
Bacillus pseudofirmus OF4 uid45847
Bacillus megaterium DSM319 uid48371
Bacillus megaterium QM B1551 uid15862
Bacillus megaterium WSH 002 uid159841
Bacillus cytotoxicus NVH 391 98 uid58317
Bacillus weihenstephanensis KBAB4 uid58315
Bacillus thuringiensis serovar chinensis CT 43 uid158151
Bacillus cereus G9842 uid58759
Bacillus cereus ATCC 14579 uid57975
Bacillus cereus B4264 uid58757
Bacillus thuringiensis BMB171 uid49135
Bacillus cereus ATCC 10987 uid57673
Bacillus thuringiensis serovar finitimus YBT 020 uid158875
Bacillus cereus 03BB102 uid59299
Bacillus cereus Q1 uid58529
Bacillus cereus AH187 uid58753
Bacillus cereus NC7401 uid82815
Bacillus cereus E33L uid58103
Bacillus cereus biovar anthracis CI uid50615
Bacillus cereus AH820 uid58751
Bacillus thuringiensis Al Hakam uid58795
Bacillus thuringiensis serovar konkukian 97 27 uid58089
Bacillus anthracis H9401 uid162021
Bacillus anthracis CDC 684 uid59303
Bacillus anthracis Ames uid57909
Bacillus anthracis A0248 uid59385
Bacillus anthracis Sterne uid58091
Bacillus anthracis  Ames Ancestor  uid58083
efp

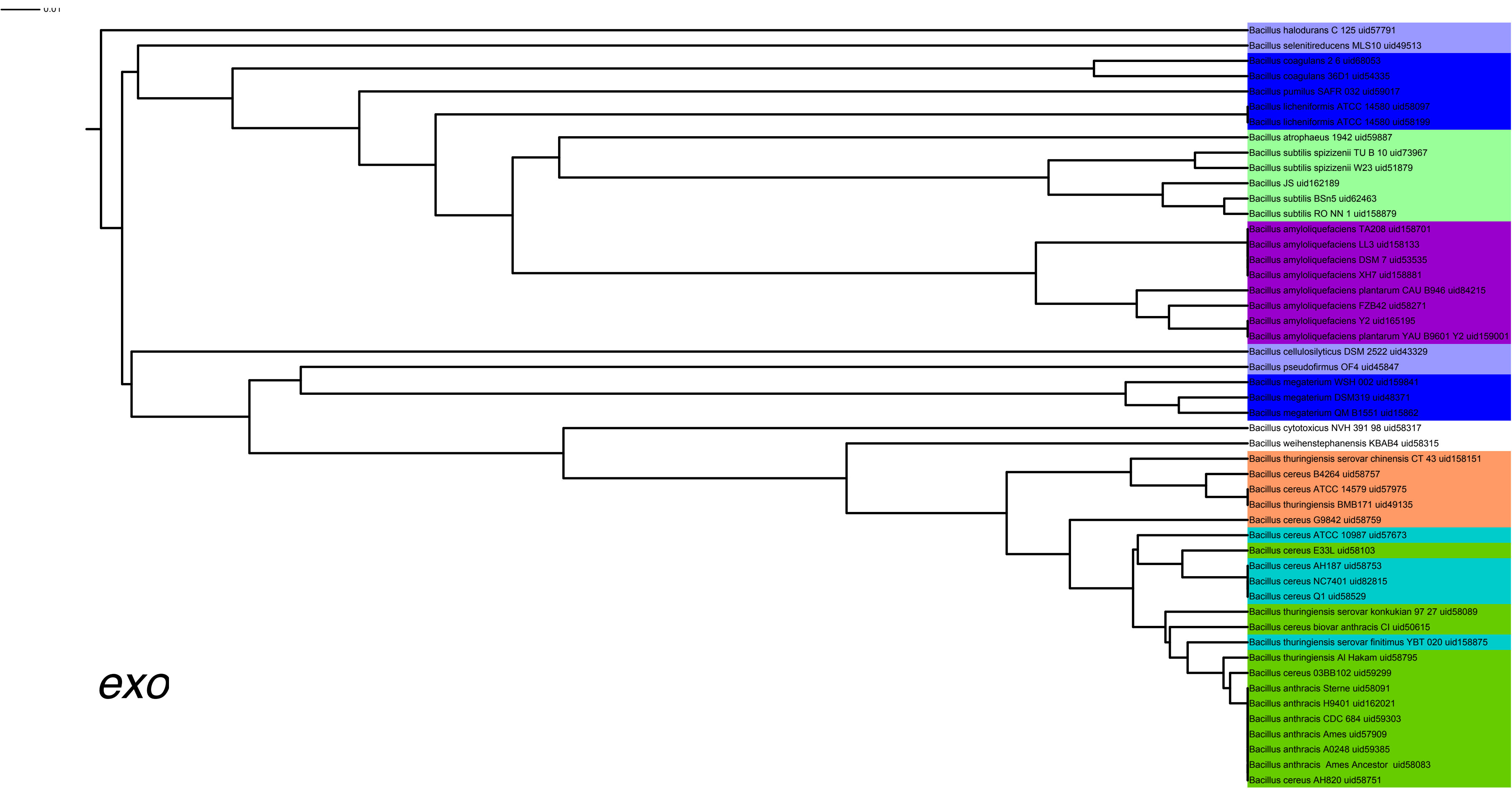

0.01
Bacillus halodurans C 125 uid57791
Bacillus selenitireducens MLS10 uid49513
Bacillus coagulans 2 6 uid68053
Bacillus coagulans 36D1 uid54335
Bacillus pumilus SAFR 032 uid59017
Bacillus licheniformis ATCC 14580 uid58097
Bacillus licheniformis ATCC 14580 uid58199
Bacillus atrophaeus 1942 uid59887
Bacillus subtilis spizizenii TU B 10 uid73967
Bacillus subtilis spizizenii W23 uid51879
Bacillus JS uid162189
Bacillus subtilis BSn5 uid62463
Bacillus subtilis RO NN 1 uid158879
Bacillus amyloliquefaciens TA208 uid158701
Bacillus amyloliquefaciens LL3 uid158133
Bacillus amyloliquefaciens DSM 7 uid53535
Bacillus amyloliquefaciens XH7 uid158881
Bacillus amyloliquefaciens plantarum CAU B946 uid84215
Bacillus amyloliquefaciens FZB42 uid58271
Bacillus amyloliquefaciens Y2 uid165195
Bacillus amyloliquefaciens plantarum YAU B9601 Y2 uid159001
Bacillus cellulosilyticus DSM 2522 uid43329
Bacillus pseudofirmus OF4 uid45847
Bacillus megaterium WSH 002 uid159841
Bacillus megaterium DSM319 uid48371
Bacillus megaterium QM B1551 uid1586[illegible]
Bacillus cytotoxicus NVH 391 98 uid58317
Bacillus weihenstephanensis KBAB4 uid58315
Bacillus thuringiensis serovar chinensis CT 43 uid158151
Bacillus cereus B4264 uid58757
Bacillus cereus ATCC 14579 uid57975
Bacillus thuringiensis BMB171 uid49135
Bacillus cereus G9842 uid58759
Bacillus cereus ATCC 10987 uid57673
Bacillus cereus E33L uid58103
Bacillus cereus AH187 uid58753
Bacillus cereus NC7401 uid82815
Bacillus cereus Q1 uid58529
Bacillus thuringiensis serovar konkukian 97 27 uid58089
Bacillus cereus biovar anthracis CI uid50615
Bacillus thuringiensis serovar finitimus YBT 020 uid158875
Bacillus thuringiensis Al Hakam uid58795
Bacillus cereus 03BB102 uid59299
Bacillus anthracis Sterne uid58091
Bacillus anthracis H9401 uid162021
Bacillus anthracis CDC 684 uid59303
Bacillus anthracis Ames uid57909
Bacillus anthracis A0248 uid59385
Bacillus anthracis Ames Ancestor uid58083
Bacillus cereus AH820 uid58751
exo

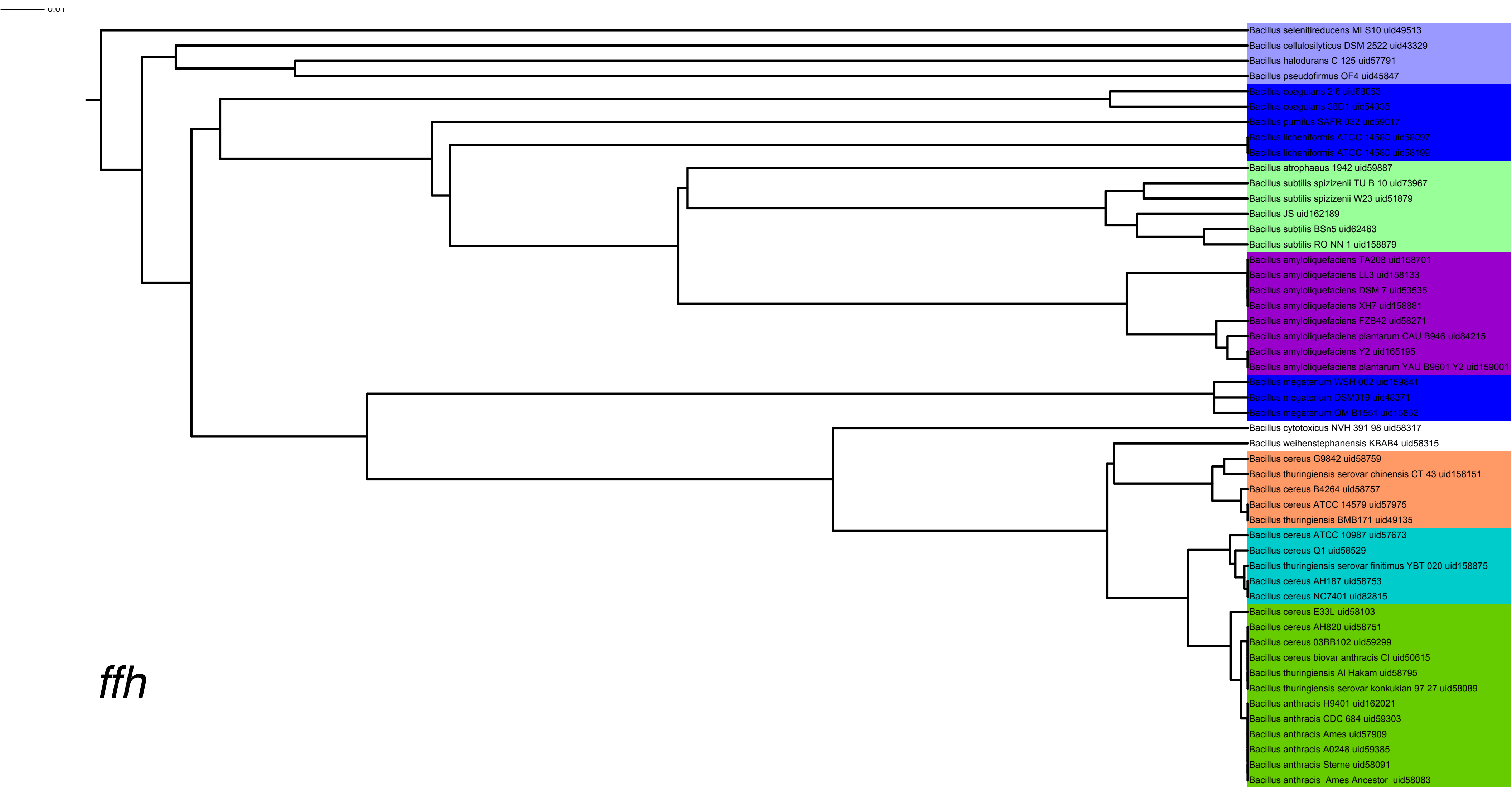
0.01
Bacillus selenitireducens MLS10 uid49513
Bacillus cellulosilyticus DSM 2522 uid43329
Bacillus halodurans C 125 uid57791
Bacillus pseudofirmus OF4 uid45847
Bacillus coagulans 2 6 uid68053
Bacillus coagulans 36D1 uid54335
Bacillus pumilus SAFR 032 uid59017
Bacillus licheniformis ATCC 14580 uid58097
Bacillus licheniformis ATCC 14580 uid58199
Bacillus atrophaeus 1942 uid59887
Bacillus subtilis spizizenii TU B 10 uid73967
Bacillus subtilis spizizenii W23 uid51879
Bacillus JS uid162189
Bacillus subtilis BSn5 uid62463
Bacillus subtilis RO NN 1 uid158879
Bacillus amyloliquefaciens TA208 uid158701
Bacillus amyloliquefaciens LL3 uid158133
Bacillus amyloliquefaciens DSM 7 uid53535
Bacillus amyloliquefaciens XH7 uid158881
Bacillus amyloliquefaciens FZB42 uid58271
Bacillus amyloliquefaciens plantarum CAU B946 uid84215
Bacillus amyloliquefaciens Y2 uid165195
Bacillus amyloliquefaciens plantarum YAU B9601 Y2 uid159001
Bacillus megaterium WSH 002 uid159841
Bacillus megaterium DSM319 uid48371
Bacillus megaterium QM B1551 uid158862
Bacillus cytotoxicus NVH 391 98 uid58317
Bacillus weihenstephanensis KBAB4 uid58315
Bacillus cereus G9842 uid58759
Bacillus thuringiensis serovar chinensis CT 43 uid158151
Bacillus cereus B4264 uid58757
Bacillus cereus ATCC 14579 uid57975
Bacillus thuringiensis BMB171 uid49135
Bacillus cereus ATCC 10987 uid57673
Bacillus cereus Q1 uid58529
Bacillus thuringiensis serovar finitimus YBT 020 uid158875
Bacillus cereus AH187 uid58753
Bacillus cereus NC7401 uid82815
Bacillus cereus E33L uid58103
Bacillus cereus AH820 uid58751
Bacillus cereus 03BB102 uid59299
Bacillus cereus biovar anthracis CI uid50615
Bacillus thuringiensis Al Hakam uid58795
Bacillus thuringiensis serovar konkukian 97 27 uid58089
Bacillus anthracis H9401 uid162021
Bacillus anthracis CDC 684 uid59303
Bacillus anthracis Ames uid57909
Bacillus anthracis A0248 uid59385
Bacillus anthracis Sterne uid58091
Bacillus anthracis Ames Ancestor uid58083
ffh

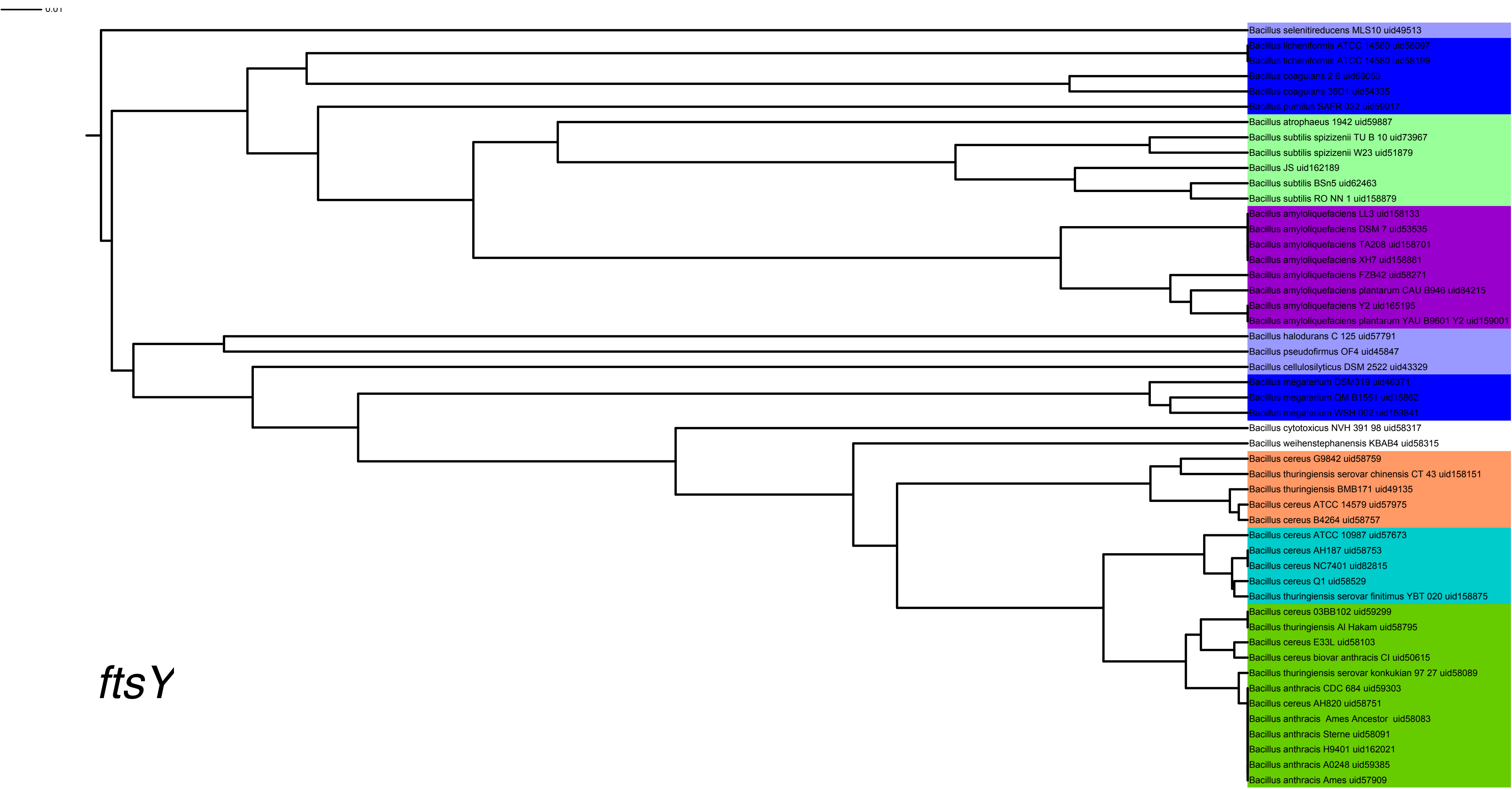
0.01
Bacillus selenitireducens MLS10 uid49513
Bacillus licheniformis ATCC 14580 uid58097
Bacillus licheniformis ATCC 14580 uid58199
Bacillus coagulans 2 6 uid68053
Bacillus coagulans 36D1 uid54335
Bacillus pumilus SAFR 032 uid59017
Bacillus atrophaeus 1942 uid59887
Bacillus subtilis spizizenii TU B 10 uid73967
Bacillus subtilis spizizenii W23 uid51879
Bacillus JS uid162189
Bacillus subtilis BSn5 uid62463
Bacillus subtilis RO NN 1 uid158879
Bacillus amyloliquefaciens LL3 uid158133
Bacillus amyloliquefaciens DSM 7 uid53535
Bacillus amyloliquefaciens TA208 uid158701
Bacillus amyloliquefaciens XH7 uid158881
Bacillus amyloliquefaciens FZB42 uid58271
Bacillus amyloliquefaciens plantarum CAU B946 uid84215
Bacillus amyloliquefaciens Y2 uid165195
Bacillus amyloliquefaciens plantarum YAU B9601 Y2 uid159001
Bacillus halodurans C 125 uid57791
Bacillus pseudofirmus OF4 uid45847
Bacillus cellulosilyticus DSM 2522 uid43329
Bacillus megaterium DSM319 uid48371
Bacillus megaterium QM B1551 uid15862
Bacillus megaterium WSH 002 uid159841
Bacillus cytotoxicus NVH 391 98 uid58317
Bacillus weihenstephanensis KBAB4 uid58315
Bacillus cereus G9842 uid58759
Bacillus thuringiensis serovar chinensis CT 43 uid158151
Bacillus thuringiensis BMB171 uid49135
Bacillus cereus ATCC 14579 uid57975
Bacillus cereus B4264 uid58757
Bacillus cereus ATCC 10987 uid57673
Bacillus cereus AH187 uid58753
Bacillus cereus NC7401 uid82815
Bacillus cereus Q1 uid58529
Bacillus thuringiensis serovar finitimus YBT 020 uid158875
Bacillus cereus 03BB102 uid59299
Bacillus thuringiensis Al Hakam uid58795
Bacillus cereus E33L uid58103
Bacillus cereus biovar anthracis CI uid50615
Bacillus thuringiensis serovar konkukian 97 27 uid58089
Bacillus anthracis CDC 684 uid59303
Bacillus cereus AH820 uid58751
Bacillus anthracis Ames Ancestor uid58083
Bacillus anthracis Sterne uid58091
Bacillus anthracis H9401 uid162021
Bacillus anthracis A0248 uid59385
Bacillus anthracis Ames uid57909
ftsY

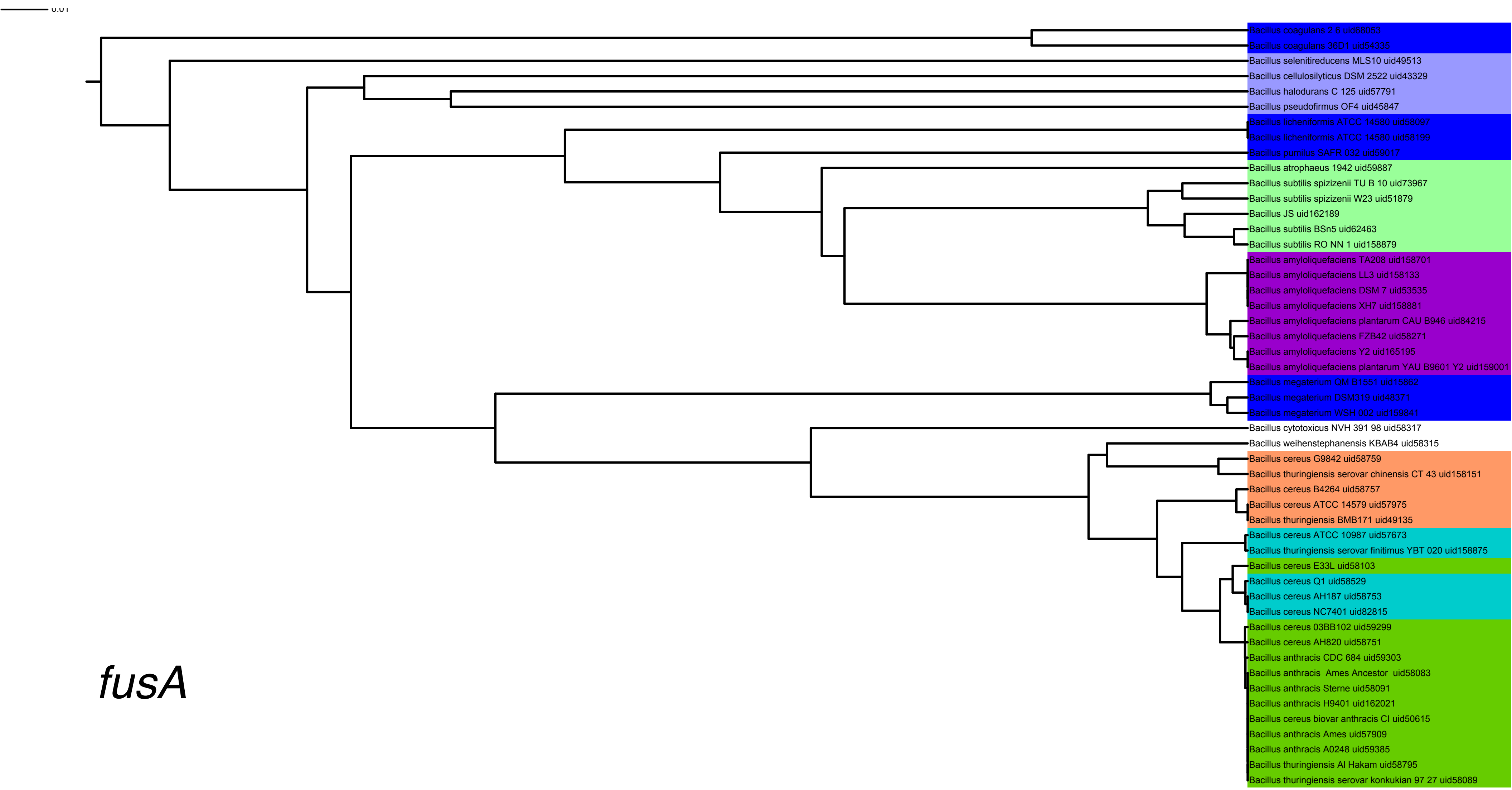
0.01
Bacillus coagulans 2 6 uid68053
Bacillus coagulans 36D1 uid54335
Bacillus selenitireducens MLS10 uid49513
Bacillus cellulosilyticus DSM 2522 uid43329
Bacillus halodurans C 125 uid57791
Bacillus pseudofirmus OF4 uid45847
Bacillus licheniformis ATCC 14580 uid58097
Bacillus licheniformis ATCC 14580 uid58199
Bacillus pumilus SAFR 032 uid59017
Bacillus atrophaeus 1942 uid59887
Bacillus subtilis spizizenii TU B 10 uid73967
Bacillus subtilis spizizenii W23 uid51879
Bacillus JS uid162189
Bacillus subtilis BSn5 uid62463
Bacillus subtilis RO NN 1 uid158879
Bacillus amyloliquefaciens TA208 uid158701
Bacillus amyloliquefaciens LL3 uid158133
Bacillus amyloliquefaciens DSM 7 uid53535
Bacillus amyloliquefaciens XH7 uid158881
Bacillus amyloliquefaciens plantarum CAU B946 uid84215
Bacillus amyloliquefaciens FZB42 uid58271
Bacillus amyloliquefaciens Y2 uid165195
Bacillus amyloliquefaciens plantarum YAU B9601 Y2 uid159001
Bacillus megaterium QM B1551 uid15862
Bacillus megaterium DSM319 uid48371
Bacillus megaterium WSH 002 uid159841
Bacillus cytotoxicus NVH 391 98 uid58317
Bacillus weihenstephanensis KBAB4 uid58315
Bacillus cereus G9842 uid58759
Bacillus thuringiensis serovar chinensis CT 43 uid158151
Bacillus cereus B4264 uid58757
Bacillus cereus ATCC 14579 uid57975
Bacillus thuringiensis BMB171 uid49135
Bacillus cereus ATCC 10987 uid57673
Bacillus thuringiensis serovar finitimus YBT 020 uid158875
Bacillus cereus E33L uid58103
Bacillus cereus Q1 uid58529
Bacillus cereus AH187 uid58753
Bacillus cereus NC7401 uid82815
Bacillus cereus 03BB102 uid59299
Bacillus cereus AH820 uid58751
Bacillus anthracis CDC 684 uid59303
Bacillus anthracis Ames Ancestor uid58083
Bacillus anthracis Sterne uid58091
Bacillus anthracis H9401 uid162021
Bacillus cereus biovar anthracis CI uid50615
Bacillus anthracis Ames uid57909
Bacillus anthracis A0248 uid59385
Bacillus thuringiensis Al Hakam uid58795
Bacillus thuringiensis serovar konkukian 97 27 uid58089
fusA

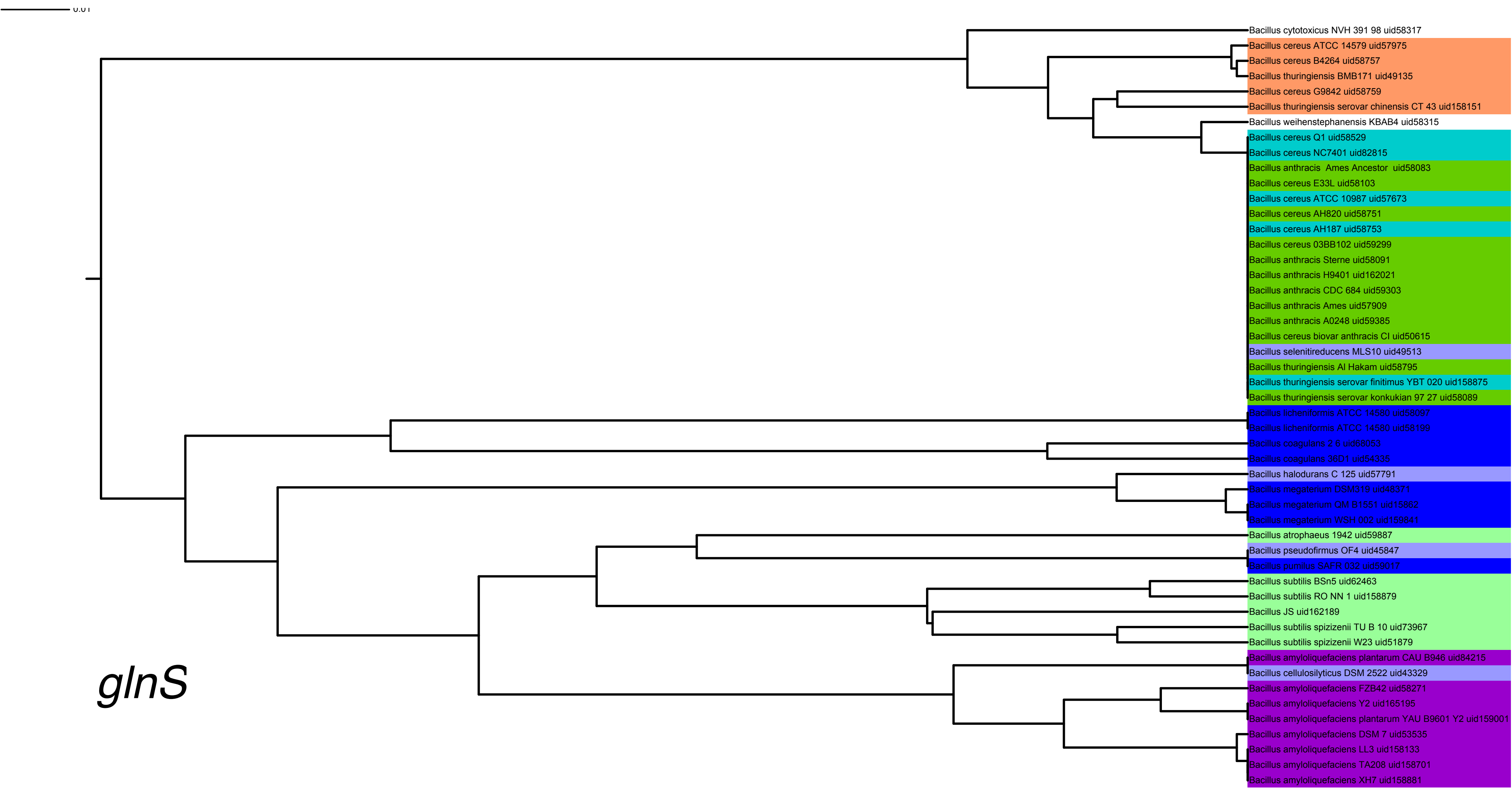
0.01
Bacillus cytotoxicus NVH 391 98 uid58317
Bacillus cereus ATCC 14579 uid57975
Bacillus cereus B4264 uid58757
Bacillus thuringiensis BMB171 uid49135
Bacillus cereus G9842 uid58759
Bacillus thuringiensis serovar chinensis CT 43 uid158151
Bacillus weihenstephanensis KBAB4 uid58315
Bacillus cereus Q1 uid58529
Bacillus cereus NC7401 uid82815
Bacillus anthracis Ames Ancestor uid58083
Bacillus cereus E33L uid58103
Bacillus cereus ATCC 10987 uid57673
Bacillus cereus AH820 uid58751
Bacillus cereus AH187 uid58753
Bacillus cereus 03BB102 uid59299
Bacillus anthracis Sterne uid58091
Bacillus anthracis H9401 uid162021
Bacillus anthracis CDC 684 uid59303
Bacillus anthracis Ames uid57909
Bacillus anthracis A0248 uid59385
Bacillus cereus biovar anthracis CI uid50615
Bacillus selenitireducens MLS10 uid49513
Bacillus thuringiensis Al Hakam uid58795
Bacillus thuringiensis serovar finitimus YBT 020 uid158875
Bacillus thuringiensis serovar konkukian 97 27 uid58089
Bacillus licheniformis ATCC 14580 uid58097
Bacillus licheniformis ATCC 14580 uid58199
Bacillus coagulans 2 6 uid68053
Bacillus coagulans 36D1 uid54335
Bacillus halodurans C 125 uid57791
Bacillus megaterium DSM319 uid48371
Bacillus megaterium QM B1551 uid16862
Bacillus megaterium WSH 002 uid159841
Bacillus atrophaeus 1942 uid59887
Bacillus pseudofirmus OF4 uid45847
Bacillus pumilus SAFR 032 uid59017
Bacillus subtilis BSn5 uid62463
Bacillus subtilis RO NN 1 uid158879
Bacillus JS uid162189
Bacillus subtilis spizizenii TU B 10 uid73967
Bacillus subtilis spizizenii W23 uid51879
Bacillus amyloliquefaciens plantarum CAU B946 uid84215
Bacillus cellulosilyticus DSM 2522 uid43329
Bacillus amyloliquefaciens FZB42 uid58271
Bacillus amyloliquefaciens Y2 uid165195
Bacillus amyloliquefaciens plantarum YAU B9601 Y2 uid159001
Bacillus amyloliquefaciens DSM 7 uid53535
Bacillus amyloliquefaciens LL3 uid158133
Bacillus amyloliquefaciens TA208 uid158701
Bacillus amyloliquefaciens XH7 uid158881
glnS

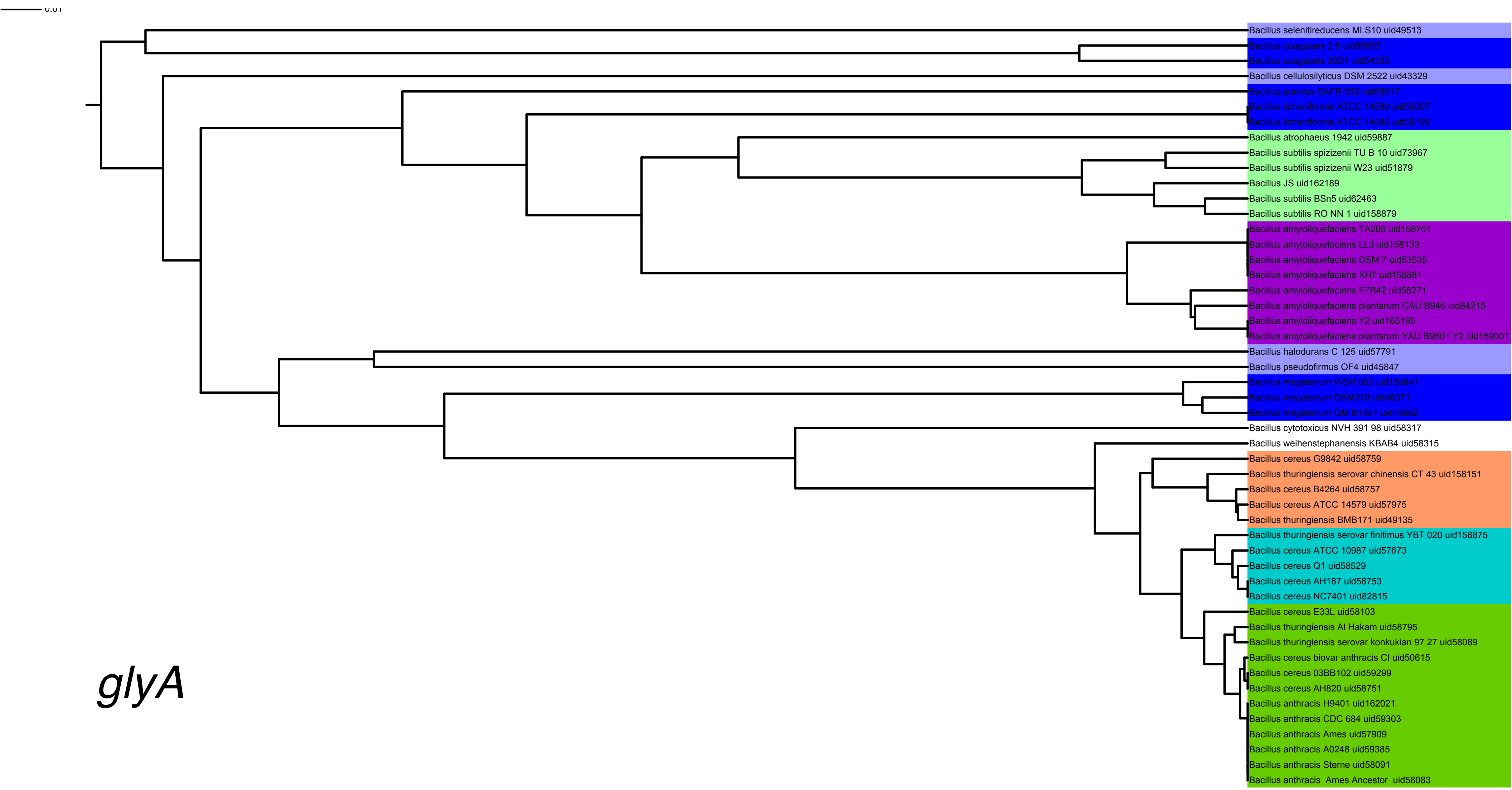
0.01
Bacillus selenitireducens MLS10 uid49513
Bacillus coagulans 2 6 uid68053
Bacillus coagulans 36D1 uid54335
Bacillus cellulosilyticus DSM 2522 uid43329
Bacillus pumilus SAFR 032 uid59017
Bacillus licheniformis ATCC 14580 uid58097
Bacillus licheniformis ATCC 14580 uid58199
Bacillus atrophaeus 1942 uid59887
Bacillus subtilis spizizenii TU B 10 uid73967
Bacillus subtilis spizizenii W23 uid51879
Bacillus JS uid162189
Bacillus subtilis BSn5 uid62463
Bacillus subtilis RO NN 1 uid158879
Bacillus amyloliquefaciens TA208 uid158701
Bacillus amyloliquefaciens LL3 uid158133
Bacillus amyloliquefaciens DSM 7 uid53535
Bacillus amyloliquefaciens XH7 uid158881
Bacillus amyloliquefaciens FZB42 uid58271
Bacillus amyloliquefaciens plantarum CAU B946 uid84215
Bacillus amyloliquefaciens Y2 uid165195
Bacillus amyloliquefaciens plantarum YAU B9601 Y2 uid159001
Bacillus halodurans C 125 uid57791
Bacillus pseudofirmus OF4 uid45847
Bacillus megaterium WSH 002 uid159841
Bacillus megaterium DSM319 uid48371
Bacillus megaterium QM B1551 uid15862
Bacillus cytotoxicus NVH 391 98 uid58317
Bacillus weihenstephanensis KBAB4 uid58315
Bacillus cereus G9842 uid58759
Bacillus thuringiensis serovar chinensis CT 43 uid158151
Bacillus cereus B4264 uid58757
Bacillus cereus ATCC 14579 uid57975
Bacillus thuringiensis BMB171 uid49135
Bacillus thuringiensis serovar finitimus YBT 020 uid158875
Bacillus cereus ATCC 10987 uid57673
Bacillus cereus Q1 uid58529
Bacillus cereus AH187 uid58753
Bacillus cereus NC7401 uid82815
Bacillus cereus E33L uid58103
Bacillus thuringiensis Al Hakam uid58795
Bacillus thuringiensis serovar konkukian 97 27 uid58089
Bacillus cereus biovar anthracis CI uid50615
Bacillus cereus 03BB102 uid59299
Bacillus cereus AH820 uid58751
Bacillus anthracis H9401 uid162021
Bacillus anthracis CDC 684 uid59303
Bacillus anthracis Ames uid57909
Bacillus anthracis A0248 uid59385
Bacillus anthracis Sterne uid58091
Bacillus anthracis Ames Ancestor uid58083
glyA

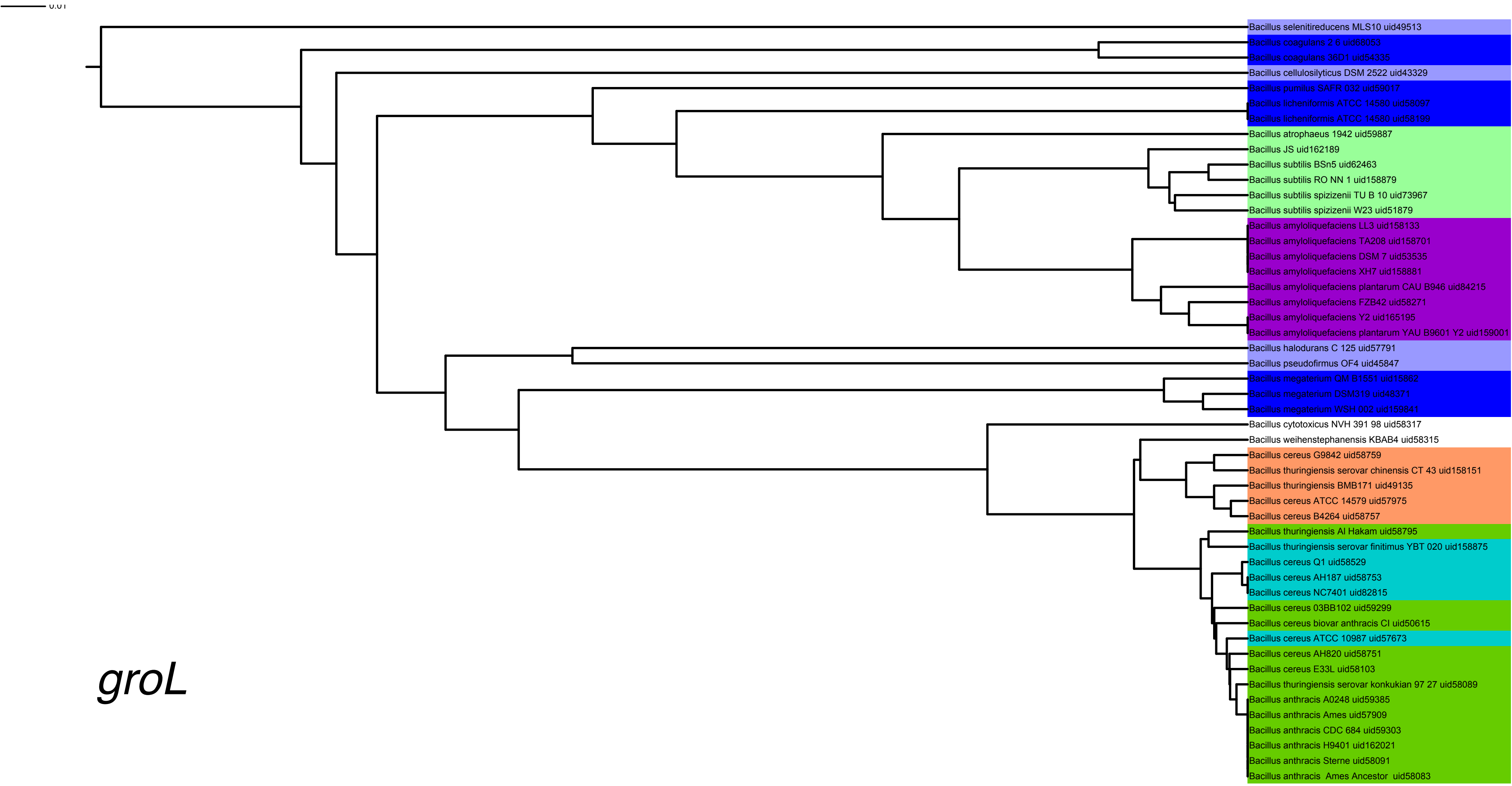
0.01
Bacillus selenitireducens MLS10 uid49513
Bacillus coagulans 2 6 uid68053
Bacillus coagulans 36D1 uid54335
Bacillus cellulosilyticus DSM 2522 uid43329
Bacillus pumilus SAFR 032 uid59017
Bacillus licheniformis ATCC 14580 uid58097
Bacillus licheniformis ATCC 14580 uid58199
Bacillus atrophaeus 1942 uid59887
Bacillus JS uid162189
Bacillus subtilis BSn5 uid62463
Bacillus subtilis RO NN 1 uid158879
Bacillus subtilis spizizenii TU B 10 uid73967
Bacillus subtilis spizizenii W23 uid51879
Bacillus amyloliquefaciens LL3 uid158133
Bacillus amyloliquefaciens TA208 uid158701
Bacillus amyloliquefaciens DSM 7 uid53535
Bacillus amyloliquefaciens XH7 uid158881
Bacillus amyloliquefaciens plantarum CAU B946 uid84215
Bacillus amyloliquefaciens FZB42 uid58271
Bacillus amyloliquefaciens Y2 uid165195
Bacillus amyloliquefaciens plantarum YAU B9601 Y2 uid159001
Bacillus halodurans C 125 uid57791
Bacillus pseudofirmus OF4 uid45847
Bacillus megaterium QM B1551 uid15862
Bacillus megaterium DSM319 uid48371
Bacillus megaterium WSH 002 uid159841
Bacillus cytotoxicus NVH 391 98 uid58317
Bacillus weihenstephanensis KBAB4 uid58315
Bacillus cereus G9842 uid58759
Bacillus thuringiensis serovar chinensis CT 43 uid158151
Bacillus thuringiensis BMB171 uid49135
Bacillus cereus ATCC 14579 uid57975
Bacillus cereus B4264 uid58757
Bacillus thuringiensis Al Hakam uid58795
Bacillus thuringiensis serovar finitimus YBT 020 uid158875
Bacillus cereus Q1 uid58529
Bacillus cereus AH187 uid58753
Bacillus cereus NC7401 uid82815
Bacillus cereus 03BB102 uid59299
Bacillus cereus biovar anthracis CI uid50615
Bacillus cereus ATCC 10987 uid57673
Bacillus cereus AH820 uid58751
Bacillus cereus E33L uid58103
Bacillus thuringiensis serovar konkukian 97 27 uid58089
Bacillus anthracis A0248 uid59385
Bacillus anthracis Ames uid57909
Bacillus anthracis CDC 684 uid59303
Bacillus anthracis H9401 uid162021
Bacillus anthracis Sterne uid58091
Bacillus anthracis Ames Ancestor uid58083
groL

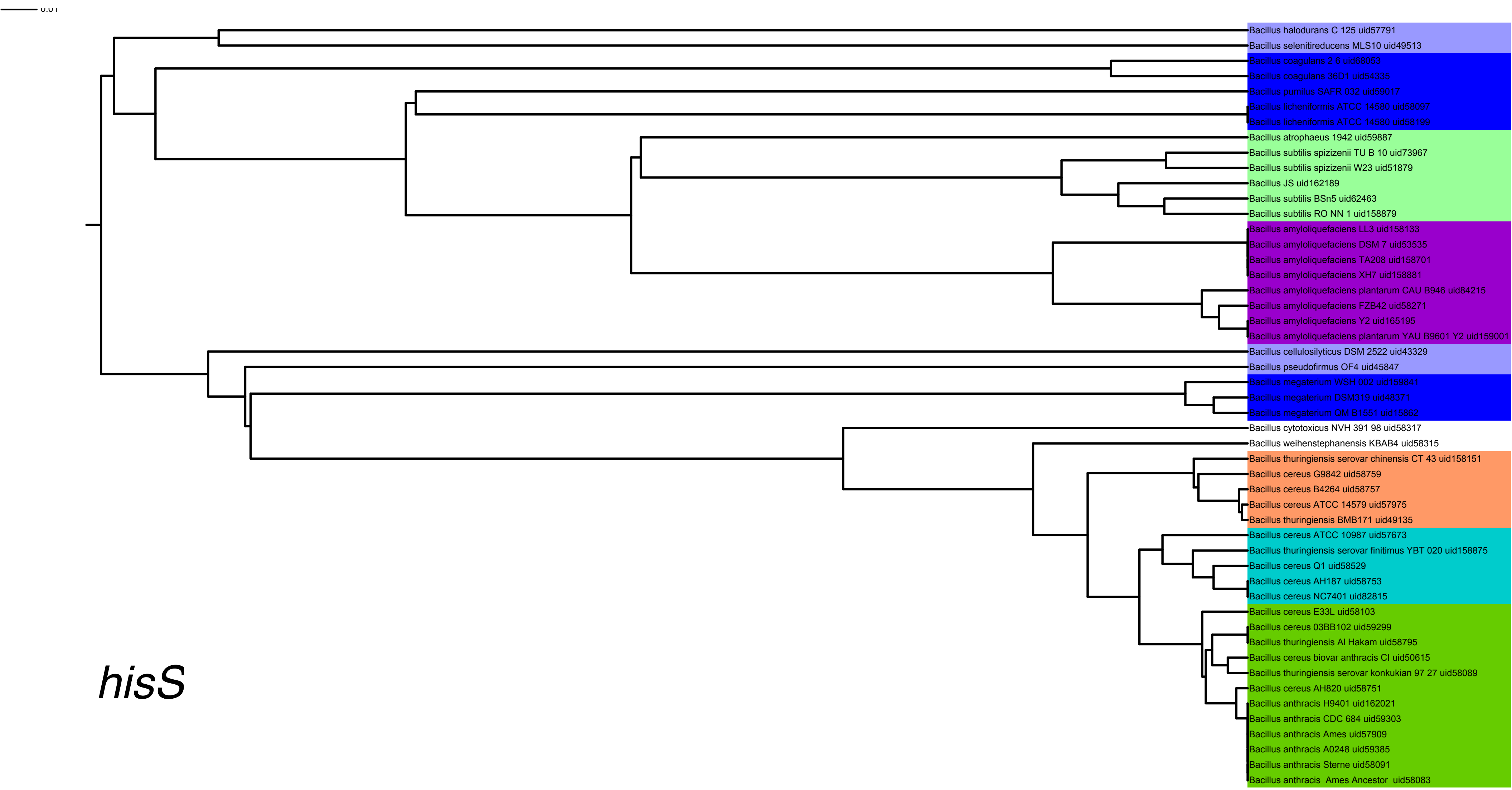

0.01
Bacillus halodurans C 125 uid57791
Bacillus selenitireducens MLS10 uid49513
Bacillus coagulans 2 6 uid68053
Bacillus coagulans 36D1 uid54335
Bacillus pumilus SAFR 032 uid59017
Bacillus licheniformis ATCC 14580 uid58097
Bacillus licheniformis ATCC 14580 uid58199
Bacillus atrophaeus 1942 uid59887
Bacillus subtilis spizizenii TU B 10 uid73967
Bacillus subtilis spizizenii W23 uid51879
Bacillus JS uid162189
Bacillus subtilis BSn5 uid62463
Bacillus subtilis RO NN 1 uid158879
Bacillus amyloliquefaciens LL3 uid158133
Bacillus amyloliquefaciens DSM 7 uid53535
Bacillus amyloliquefaciens TA208 uid158701
Bacillus amyloliquefaciens XH7 uid158881
Bacillus amyloliquefaciens plantarum CAU B946 uid84215
Bacillus amyloliquefaciens FZB42 uid58271
Bacillus amyloliquefaciens Y2 uid165195
Bacillus amyloliquefaciens plantarum YAU B9601 Y2 uid159001
Bacillus cellulosilyticus DSM 2522 uid43329
Bacillus pseudofirmus OF4 uid45847
Bacillus megaterium WSH 002 uid159841
Bacillus megaterium DSM319 uid48371
Bacillus megaterium QM B1551 uid15862
Bacillus cytotoxicus NVH 391 98 uid58317
Bacillus weihenstephanensis KBAB4 uid58315
Bacillus thuringiensis serovar chinensis CT 43 uid158151
Bacillus cereus G9842 uid58759
Bacillus cereus B4264 uid58757
Bacillus cereus ATCC 14579 uid57975
Bacillus thuringiensis BMB171 uid49135
Bacillus cereus ATCC 10987 uid57673
Bacillus thuringiensis serovar finitimus YBT 020 uid158875
Bacillus cereus Q1 uid58529
Bacillus cereus AH187 uid58753
Bacillus cereus NC7401 uid82815
Bacillus cereus E33L uid58103
Bacillus cereus 03BB102 uid59299
Bacillus thuringiensis Al Hakam uid58795
Bacillus cereus biovar anthracis CI uid50615
Bacillus thuringiensis serovar konkukian 97 27 uid58089
Bacillus cereus AH820 uid58751
Bacillus anthracis H9401 uid162021
Bacillus anthracis CDC 684 uid59303
Bacillus anthracis Ames uid57909
Bacillus anthracis A0248 uid59385
Bacillus anthracis Sterne uid58091
Bacillus anthracis Ames Ancestor uid58083
hisS

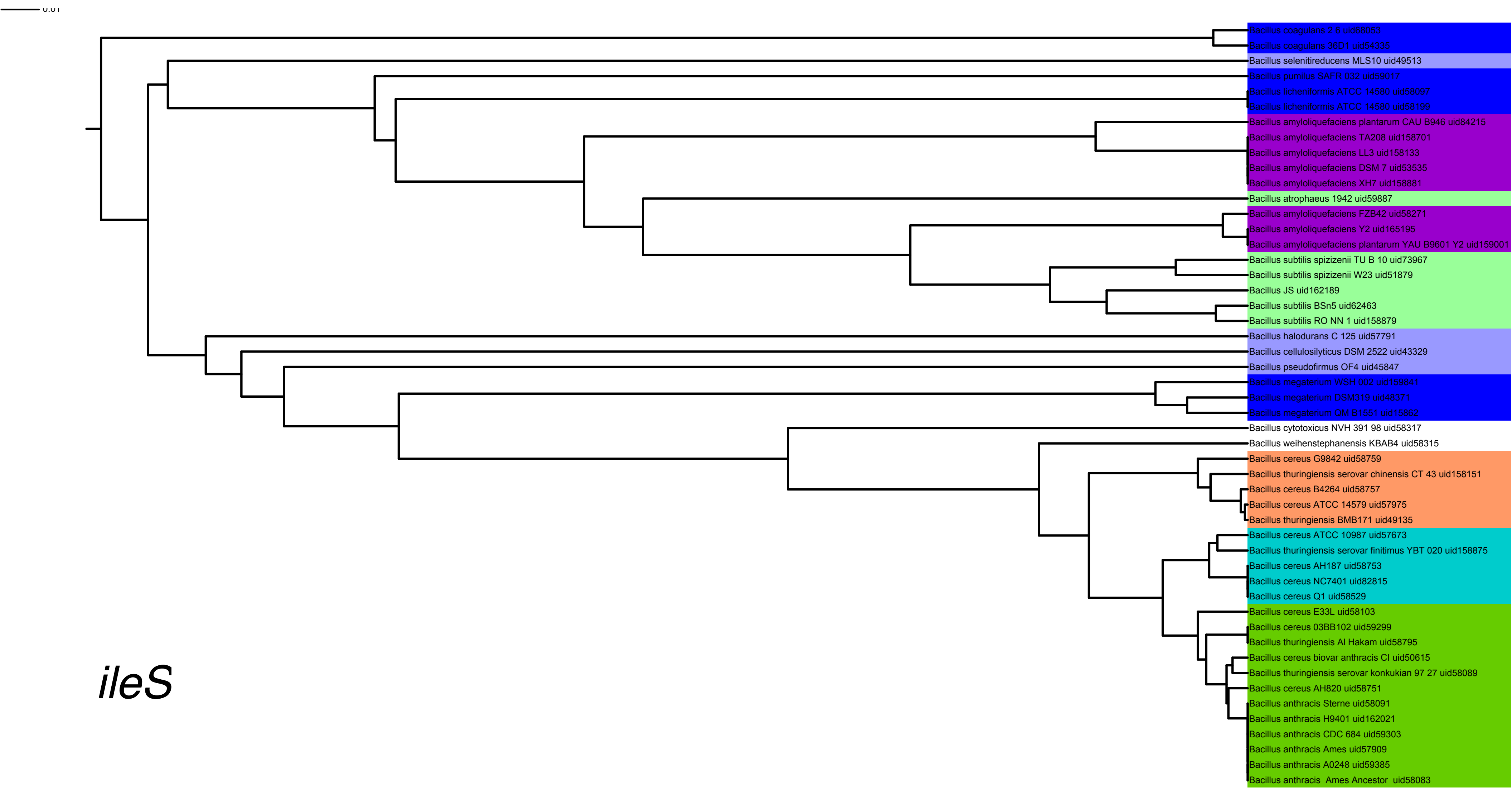
0.01
Bacillus coagulans 2 6 uid68053
Bacillus coagulans 36D1 uid54335
Bacillus selenitireducens MLS10 uid49513
Bacillus pumilus SAFR 032 uid59017
Bacillus licheniformis ATCC 14580 uid58097
Bacillus licheniformis ATCC 14580 uid58199
Bacillus amyloliquefaciens plantarum CAU B946 uid84215
Bacillus amyloliquefaciens TA208 uid158701
Bacillus amyloliquefaciens LL3 uid158133
Bacillus amyloliquefaciens DSM 7 uid53535
Bacillus amyloliquefaciens XH7 uid158881
Bacillus atrophaeus 1942 uid59887
Bacillus amyloliquefaciens FZB42 uid58271
Bacillus amyloliquefaciens Y2 uid165195
Bacillus amyloliquefaciens plantarum YAU B9601 Y2 uid159001
Bacillus subtilis spizizenii TU B 10 uid73967
Bacillus subtilis spizizenii W23 uid51879
Bacillus JS uid162189
Bacillus subtilis BSn5 uid62463
Bacillus subtilis RO NN 1 uid158879
Bacillus halodurans C 125 uid57791
Bacillus cellulosilyticus DSM 2522 uid43329
Bacillus pseudofirmus OF4 uid45847
Bacillus megaterium WSH 002 uid159841
Bacillus megaterium DSM319 uid48371
Bacillus megaterium QM B1551 uid15862
Bacillus cytotoxicus NVH 391 98 uid58317
Bacillus weihenstephanensis KBAB4 uid58315
Bacillus cereus G9842 uid58759
Bacillus thuringiensis serovar chinensis CT 43 uid158151
Bacillus cereus B4264 uid58757
Bacillus cereus ATCC 14579 uid57975
Bacillus thuringiensis BMB171 uid49135
Bacillus cereus ATCC 10987 uid57673
Bacillus thuringiensis serovar finitimus YBT 020 uid158875
Bacillus cereus AH187 uid58753
Bacillus cereus NC7401 uid82815
Bacillus cereus Q1 uid58529
Bacillus cereus E33L uid58103
Bacillus cereus 03BB102 uid59299
Bacillus thuringiensis Al Hakam uid58795
Bacillus cereus biovar anthracis CI uid50615
Bacillus thuringiensis serovar konkukian 97 27 uid58089
Bacillus cereus AH820 uid58751
Bacillus anthracis Sterne uid58091
Bacillus anthracis H9401 uid162021
Bacillus anthracis CDC 684 uid59303
Bacillus anthracis Ames uid57909
Bacillus anthracis A0248 uid59385
Bacillus anthracis  Ames Ancestor  uid58083
ileS

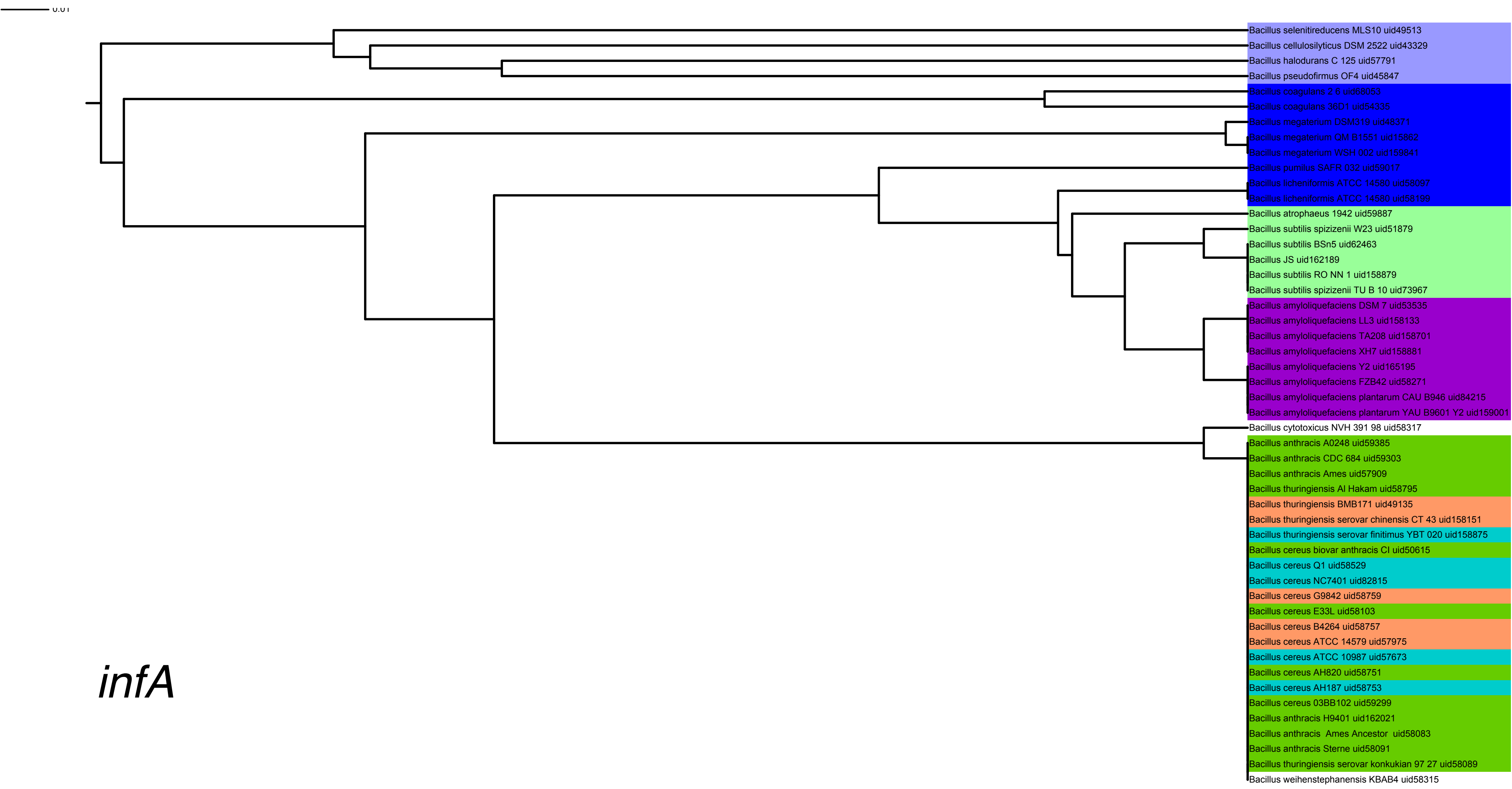
0.01
Bacillus selenitireducens MLS10 uid49513
Bacillus cellulosilyticus DSM 2522 uid43329
Bacillus halodurans C 125 uid57791
Bacillus pseudofirmus OF4 uid45847
Bacillus coagulans 2 6 uid68053
Bacillus coagulans 36D1 uid54335
Bacillus megaterium DSM319 uid48371
Bacillus megaterium QM B1551 uid15862
Bacillus megaterium WSH 002 uid159841
Bacillus pumilus SAFR 032 uid59017
Bacillus licheniformis ATCC 14580 uid58097
Bacillus licheniformis ATCC 14580 uid58199
Bacillus atrophaeus 1942 uid59887
Bacillus subtilis spizizenii W23 uid51879
Bacillus subtilis BSn5 uid62463
Bacillus JS uid162189
Bacillus subtilis RO NN 1 uid158879
Bacillus subtilis spizizenii TU B 10 uid73967
Bacillus amyloliquefaciens DSM 7 uid53535
Bacillus amyloliquefaciens LL3 uid158133
Bacillus amyloliquefaciens TA208 uid158701
Bacillus amyloliquefaciens XH7 uid158881
Bacillus amyloliquefaciens Y2 uid165195
Bacillus amyloliquefaciens FZB42 uid58271
Bacillus amyloliquefaciens plantarum CAU B946 uid84215
Bacillus amyloliquefaciens plantarum YAU B9601 Y2 uid159001
Bacillus cytotoxicus NVH 391 98 uid58317
Bacillus anthracis A0248 uid59385
Bacillus anthracis CDC 684 uid59303
Bacillus anthracis Ames uid57909
Bacillus thuringiensis Al Hakam uid58795
Bacillus thuringiensis BMB171 uid49135
Bacillus thuringiensis serovar chinensis CT 43 uid158151
Bacillus thuringiensis serovar finitimus YBT 020 uid158875
Bacillus cereus biovar anthracis CI uid50615
Bacillus cereus Q1 uid58529
Bacillus cereus NC7401 uid82815
Bacillus cereus G9842 uid58759
Bacillus cereus E33L uid58103
Bacillus cereus B4264 uid58757
Bacillus cereus ATCC 14579 uid57975
Bacillus cereus ATCC 10987 uid57673
Bacillus cereus AH820 uid58751
Bacillus cereus AH187 uid58753
Bacillus cereus 03BB102 uid59299
Bacillus anthracis H9401 uid162021
Bacillus anthracis Ames Ancestor uid58083
Bacillus anthracis Sterne uid58091
Bacillus thuringiensis serovar konkukian 97 27 uid58089
Bacillus weihenstephanensis KBAB4 uid58315
infA

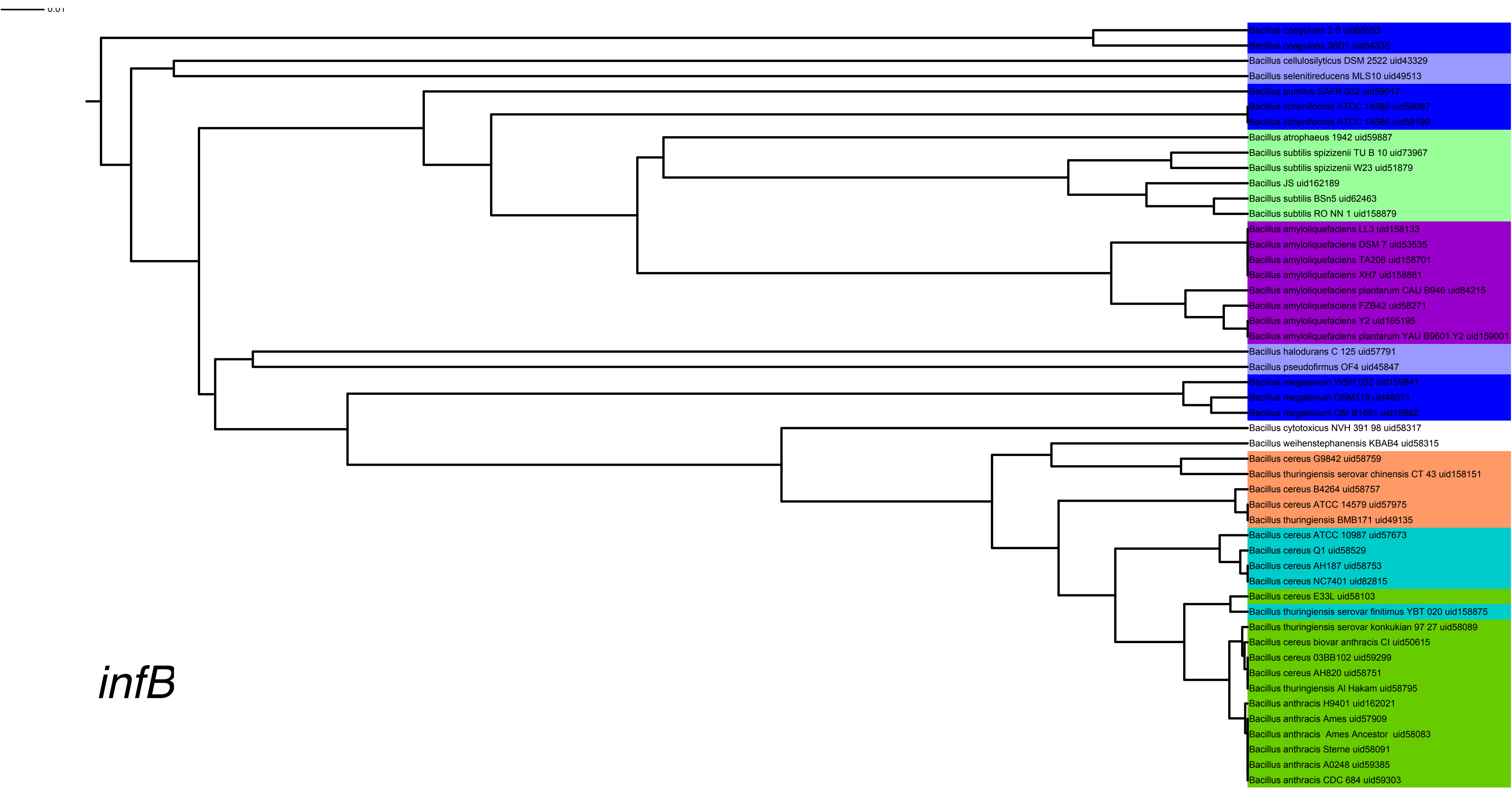
Bacillus coagulans 2 6 uid68053
Bacillus coagulans 36D1 uid54335
Bacillus cellulosilyticus DSM 2522 uid43329
Bacillus selenitireducens MLS10 uid49513
Bacillus pumilus SAFR 032 uid59017
Bacillus licheniformis ATCC 14580 uid58097
Bacillus licheniformis ATCC 14580 uid58199
Bacillus atrophaeus 1942 uid59887
Bacillus subtilis spizizenii TU B 10 uid73967
Bacillus subtilis spizizenii W23 uid51879
Bacillus JS uid162189
Bacillus subtilis BSn5 uid62463
Bacillus subtilis RO NN 1 uid158879
Bacillus amyloliquefaciens LL3 uid158133
Bacillus amyloliquefaciens DSM 7 uid53535
Bacillus amyloliquefaciens TA208 uid158701
Bacillus amyloliquefaciens XH7 uid158881
Bacillus amyloliquefaciens plantarum CAU B946 uid84215
Bacillus amyloliquefaciens FZB42 uid58271
Bacillus amyloliquefaciens Y2 uid165195
Bacillus amyloliquefaciens plantarum YAU B9601 Y2 uid159001
Bacillus halodurans C 125 uid57791
Bacillus pseudofirmus OF4 uid45847
Bacillus megaterium WSH 002 uid159841
Bacillus megaterium DSM319 uid48371
Bacillus megaterium QM B1551 uid15862
Bacillus cytotoxicus NVH 391 98 uid58317
Bacillus weihenstephanensis KBAB4 uid58315
Bacillus cereus G9842 uid58759
Bacillus thuringiensis serovar chinensis CT 43 uid158151
Bacillus cereus B4264 uid58757
Bacillus cereus ATCC 14579 uid57975
Bacillus thuringiensis BMB171 uid49135
Bacillus cereus ATCC 10987 uid57673
Bacillus cereus Q1 uid58529
Bacillus cereus AH187 uid58753
Bacillus cereus NC7401 uid82815
Bacillus cereus E33L uid58103
Bacillus thuringiensis serovar finitimus YBT 020 uid158875
Bacillus thuringiensis serovar konkukian 97 27 uid58089
Bacillus cereus biovar anthracis CI uid50615
Bacillus cereus 03BB102 uid59299
Bacillus cereus AH820 uid58751
Bacillus thuringiensis Al Hakam uid58795
Bacillus anthracis H9401 uid162021
Bacillus anthracis Ames uid57909
Bacillus anthracis  Ames Ancestor  uid58083
Bacillus anthracis Sterne uid58091
Bacillus anthracis A0248 uid59385
Bacillus anthracis CDC 684 uid59303
*infB*

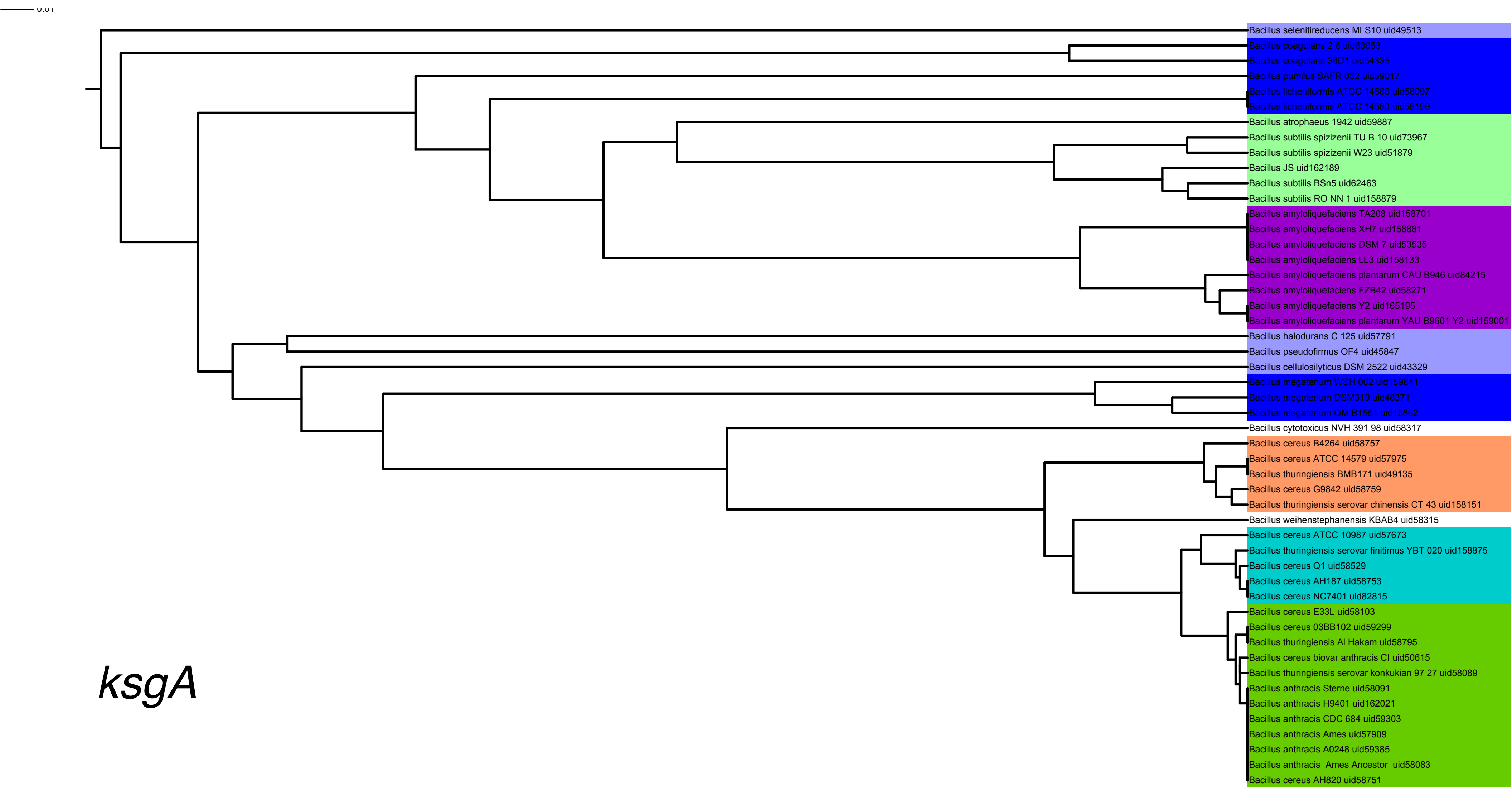

Bacillus selenitireducens MLS10 uid49513
Bacillus coagulans 2 6 uid68053
Bacillus coagulans 36D1 uid54335
Bacillus pumilus SAFR 032 uid59017
Bacillus licheniformis ATCC 14580 uid58097
Bacillus licheniformis ATCC 14580 uid58199
Bacillus atrophaeus 1942 uid59887
Bacillus subtilis spizizenii TU B 10 uid73967
Bacillus subtilis spizizenii W23 uid51879
Bacillus JS uid162189
Bacillus subtilis BSn5 uid62463
Bacillus subtilis RO NN 1 uid158879
Bacillus amyloliquefaciens TA208 uid158701
Bacillus amyloliquefaciens XH7 uid158881
Bacillus amyloliquefaciens DSM 7 uid53535
Bacillus amyloliquefaciens LL3 uid158133
Bacillus amyloliquefaciens plantarum CAU B946 uid84215
Bacillus amyloliquefaciens FZB42 uid58271
Bacillus amyloliquefaciens Y2 uid165195
Bacillus amyloliquefaciens plantarum YAU B9601 Y2 uid159001
Bacillus halodurans C 125 uid57791
Bacillus pseudofirmus OF4 uid45847
Bacillus cellulosilyticus DSM 2522 uid43329
Bacillus megaterium WSH 002 uid159841
Bacillus megaterium DSM319 uid48371
Bacillus megaterium QM B1551 uid15862
Bacillus cytotoxicus NVH 391 98 uid58317
Bacillus cereus B4264 uid58757
Bacillus cereus ATCC 14579 uid57975
Bacillus thuringiensis BMB171 uid49135
Bacillus cereus G9842 uid58759
Bacillus thuringiensis serovar chinensis CT 43 uid158151
Bacillus weihenstephanensis KBAB4 uid58315
Bacillus cereus ATCC 10987 uid57673
Bacillus thuringiensis serovar finitimus YBT 020 uid158875
Bacillus cereus Q1 uid58529
Bacillus cereus AH187 uid58753
Bacillus cereus NC7401 uid82815
Bacillus cereus E33L uid58103
Bacillus cereus 03BB102 uid59299
Bacillus thuringiensis Al Hakam uid58795
Bacillus cereus biovar anthracis CI uid50615
Bacillus thuringiensis serovar konkukian 97 27 uid58089
Bacillus anthracis Sterne uid58091
Bacillus anthracis H9401 uid162021
Bacillus anthracis CDC 684 uid59303
Bacillus anthracis Ames uid57909
Bacillus anthracis A0248 uid59385
Bacillus anthracis Ames Ancestor uid58083
Bacillus cereus AH820 uid58751
ksgA

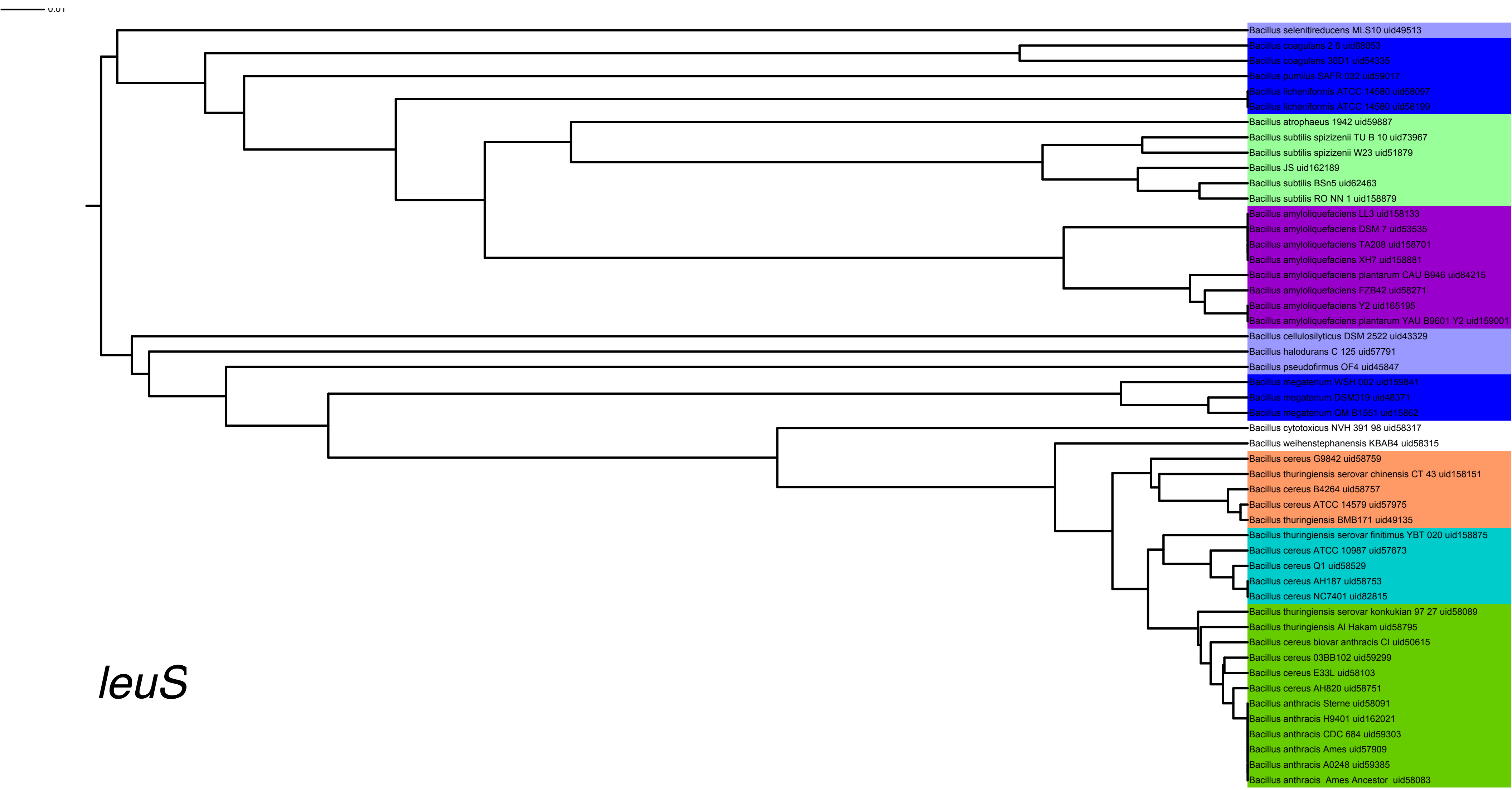

0.01
Bacillus selenitireducens MLS10 uid49513
Bacillus coagulans 2 6 uid68053
Bacillus coagulans 36D1 uid54335
Bacillus pumilus SAFR 032 uid59017
Bacillus licheniformis ATCC 14580 uid58097
Bacillus licheniformis ATCC 14580 uid58199
Bacillus atrophaeus 1942 uid59887
Bacillus subtilis spizizenii TU B 10 uid73967
Bacillus subtilis spizizenii W23 uid51879
Bacillus JS uid162189
Bacillus subtilis BSn5 uid62463
Bacillus subtilis RO NN 1 uid158879
Bacillus amyloliquefaciens LL3 uid158133
Bacillus amyloliquefaciens DSM 7 uid53535
Bacillus amyloliquefaciens TA208 uid158701
Bacillus amyloliquefaciens XH7 uid158881
Bacillus amyloliquefaciens plantarum CAU B946 uid84215
Bacillus amyloliquefaciens FZB42 uid58271
Bacillus amyloliquefaciens Y2 uid165195
Bacillus amyloliquefaciens plantarum YAU B9601 Y2 uid159001
Bacillus cellulosilyticus DSM 2522 uid43329
Bacillus halodurans C 125 uid57791
Bacillus pseudofirmus OF4 uid45847
Bacillus megaterium WSH 002 uid159841
Bacillus megaterium DSM319 uid48371
Bacillus megaterium QM B1551 uid15862
Bacillus cytotoxicus NVH 391 98 uid58317
Bacillus weihenstephanensis KBAB4 uid58315
Bacillus cereus G9842 uid58759
Bacillus thuringiensis serovar chinensis CT 43 uid158151
Bacillus cereus B4264 uid58757
Bacillus cereus ATCC 14579 uid57975
Bacillus thuringiensis BMB171 uid49135
Bacillus thuringiensis serovar finitimus YBT 020 uid158875
Bacillus cereus ATCC 10987 uid57673
Bacillus cereus Q1 uid58529
Bacillus cereus AH187 uid58753
Bacillus cereus NC7401 uid82815
Bacillus thuringiensis serovar konkukian 97 27 uid58089
Bacillus thuringiensis Al Hakam uid58795
Bacillus cereus biovar anthracis CI uid50615
Bacillus cereus 03BB102 uid59299
Bacillus cereus E33L uid58103
Bacillus cereus AH820 uid58751
Bacillus anthracis Sterne uid58091
Bacillus anthracis H9401 uid162021
Bacillus anthracis CDC 684 uid59303
Bacillus anthracis Ames uid57909
Bacillus anthracis A0248 uid59385
Bacillus anthracis Ames Ancestor uid58083
leuS

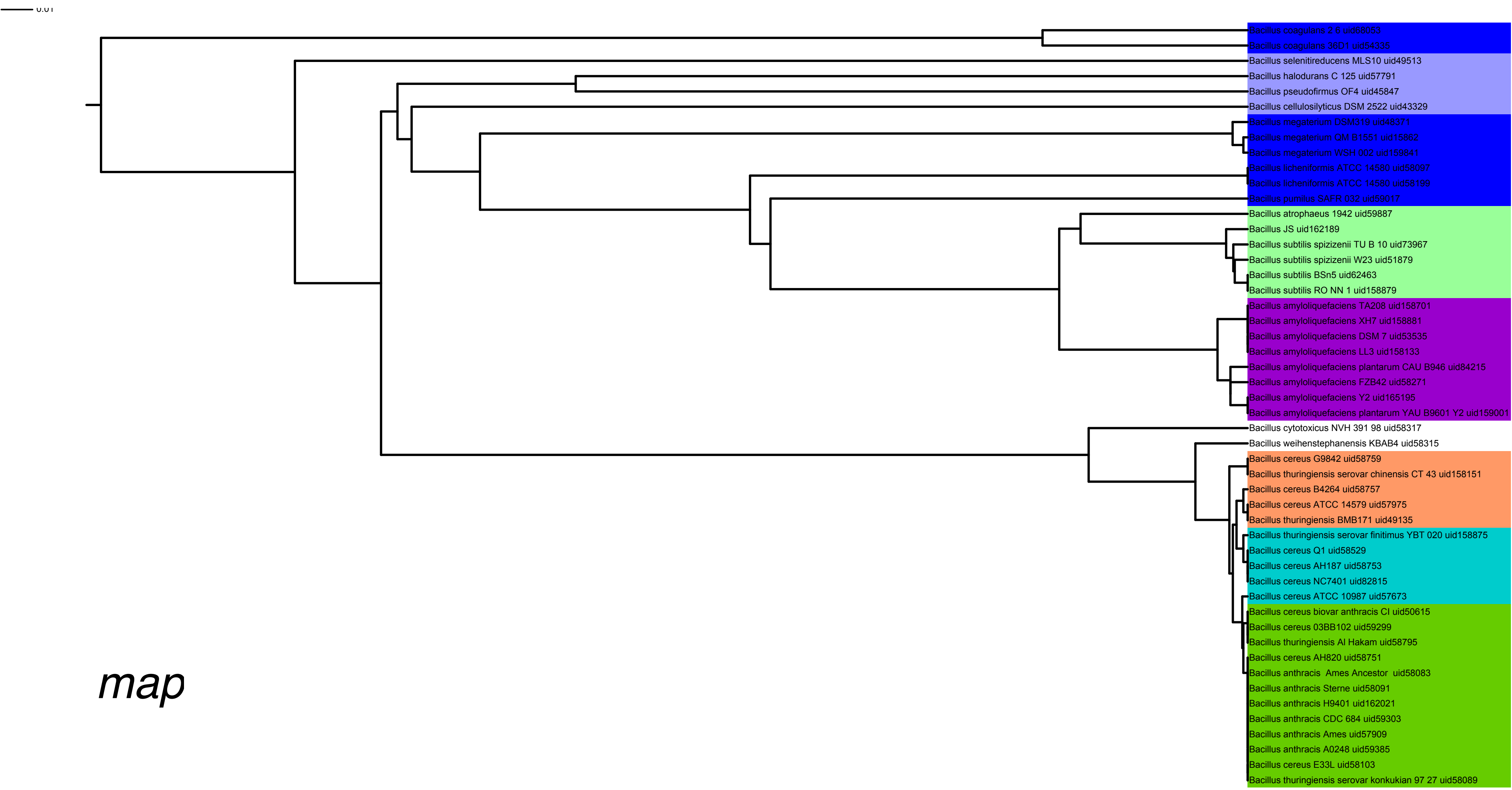

0.01
Bacillus coagulans 2 6 uid68053
Bacillus coagulans 36D1 uid54335
Bacillus selenitireducens MLS10 uid49513
Bacillus halodurans C 125 uid57791
Bacillus pseudofirmus OF4 uid45847
Bacillus cellulosilyticus DSM 2522 uid43329
Bacillus megaterium DSM319 uid48371
Bacillus megaterium QM B1551 uid15862
Bacillus megaterium WSH 002 uid159841
Bacillus licheniformis ATCC 14580 uid58097
Bacillus licheniformis ATCC 14580 uid58199
Bacillus pumilus SAFR 032 uid59017
Bacillus atrophaeus 1942 uid59887
Bacillus JS uid162189
Bacillus subtilis spizizenii TU B 10 uid73967
Bacillus subtilis spizizenii W23 uid51879
Bacillus subtilis BSn5 uid62463
Bacillus subtilis RO NN 1 uid158879
Bacillus amyloliquefaciens TA208 uid158701
Bacillus amyloliquefaciens XH7 uid158881
Bacillus amyloliquefaciens DSM 7 uid53535
Bacillus amyloliquefaciens LL3 uid158133
Bacillus amyloliquefaciens plantarum CAU B946 uid84215
Bacillus amyloliquefaciens FZB42 uid58271
Bacillus amyloliquefaciens Y2 uid165195
Bacillus amyloliquefaciens plantarum YAU B9601 Y2 uid159001
Bacillus cytotoxicus NVH 391 98 uid58317
Bacillus weihenstephanensis KBAB4 uid58315
Bacillus cereus G9842 uid58759
Bacillus thuringiensis serovar chinensis CT 43 uid158151
Bacillus cereus B4264 uid58757
Bacillus cereus ATCC 14579 uid57975
Bacillus thuringiensis BMB171 uid49135
Bacillus thuringiensis serovar finitimus YBT 020 uid158875
Bacillus cereus Q1 uid58529
Bacillus cereus AH187 uid58753
Bacillus cereus NC7401 uid82815
Bacillus cereus ATCC 10987 uid57673
Bacillus cereus biovar anthracis CI uid50615
Bacillus cereus 03BB102 uid59299
Bacillus thuringiensis Al Hakam uid58795
Bacillus cereus AH820 uid58751
Bacillus anthracis Ames Ancestor uid58083
Bacillus anthracis Sterne uid58091
Bacillus anthracis H9401 uid162021
Bacillus anthracis CDC 684 uid59303
Bacillus anthracis Ames uid57909
Bacillus anthracis A0248 uid59385
Bacillus cereus E33L uid58103
Bacillus thuringiensis serovar konkukian 97 27 uid58089
map

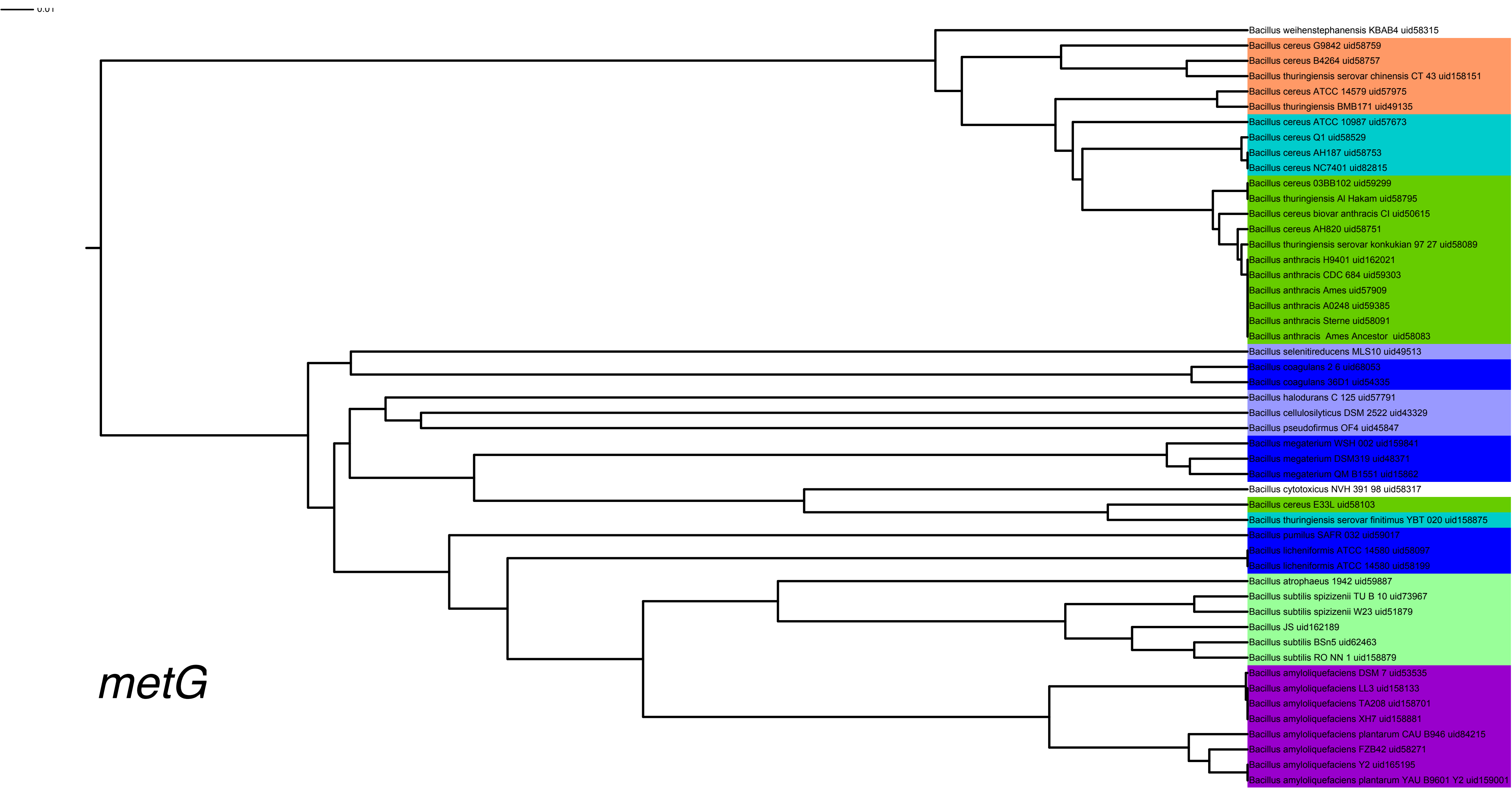
0.01
Bacillus weihenstephanensis KBAB4 uid58315
Bacillus cereus G9842 uid58759
Bacillus cereus B4264 uid58757
Bacillus thuringiensis serovar chinensis CT 43 uid158151
Bacillus cereus ATCC 14579 uid57975
Bacillus thuringiensis BMB171 uid49135
Bacillus cereus ATCC 10987 uid57673
Bacillus cereus Q1 uid58529
Bacillus cereus AH187 uid58753
Bacillus cereus NC7401 uid82815
Bacillus cereus 03BB102 uid59299
Bacillus thuringiensis Al Hakam uid58795
Bacillus cereus biovar anthracis CI uid50615
Bacillus cereus AH820 uid58751
Bacillus thuringiensis serovar konkukian 97 27 uid58089
Bacillus anthracis H9401 uid162021
Bacillus anthracis CDC 684 uid59303
Bacillus anthracis Ames uid57909
Bacillus anthracis A0248 uid59385
Bacillus anthracis Sterne uid58091
Bacillus anthracis  Ames Ancestor  uid58083
Bacillus selenitireducens MLS10 uid49513
Bacillus coagulans 2 6 uid68053
Bacillus coagulans 36D1 uid54335
Bacillus halodurans C 125 uid57791
Bacillus cellulosilyticus DSM 2522 uid43329
Bacillus pseudofirmus OF4 uid45847
Bacillus megaterium WSH 002 uid159841
Bacillus megaterium DSM319 uid48371
Bacillus megaterium QM B1551 uid15862
Bacillus cytotoxicus NVH 391 98 uid58317
Bacillus cereus E33L uid58103
Bacillus thuringiensis serovar finitimus YBT 020 uid158875
Bacillus pumilus SAFR 032 uid59017
Bacillus licheniformis ATCC 14580 uid58097
Bacillus licheniformis ATCC 14580 uid58199
Bacillus atrophaeus 1942 uid59887
Bacillus subtilis spizizenii TU B 10 uid73967
Bacillus subtilis spizizenii W23 uid51879
Bacillus JS uid162189
Bacillus subtilis BSn5 uid62463
Bacillus subtilis RO NN 1 uid158879
Bacillus amyloliquefaciens DSM 7 uid53535
Bacillus amyloliquefaciens LL3 uid158133
Bacillus amyloliquefaciens TA208 uid158701
Bacillus amyloliquefaciens XH7 uid158881
Bacillus amyloliquefaciens plantarum CAU B946 uid84215
Bacillus amyloliquefaciens FZB42 uid58271
Bacillus amyloliquefaciens Y2 uid165195
Bacillus amyloliquefaciens plantarum YAU B9601 Y2 uid159001
metG

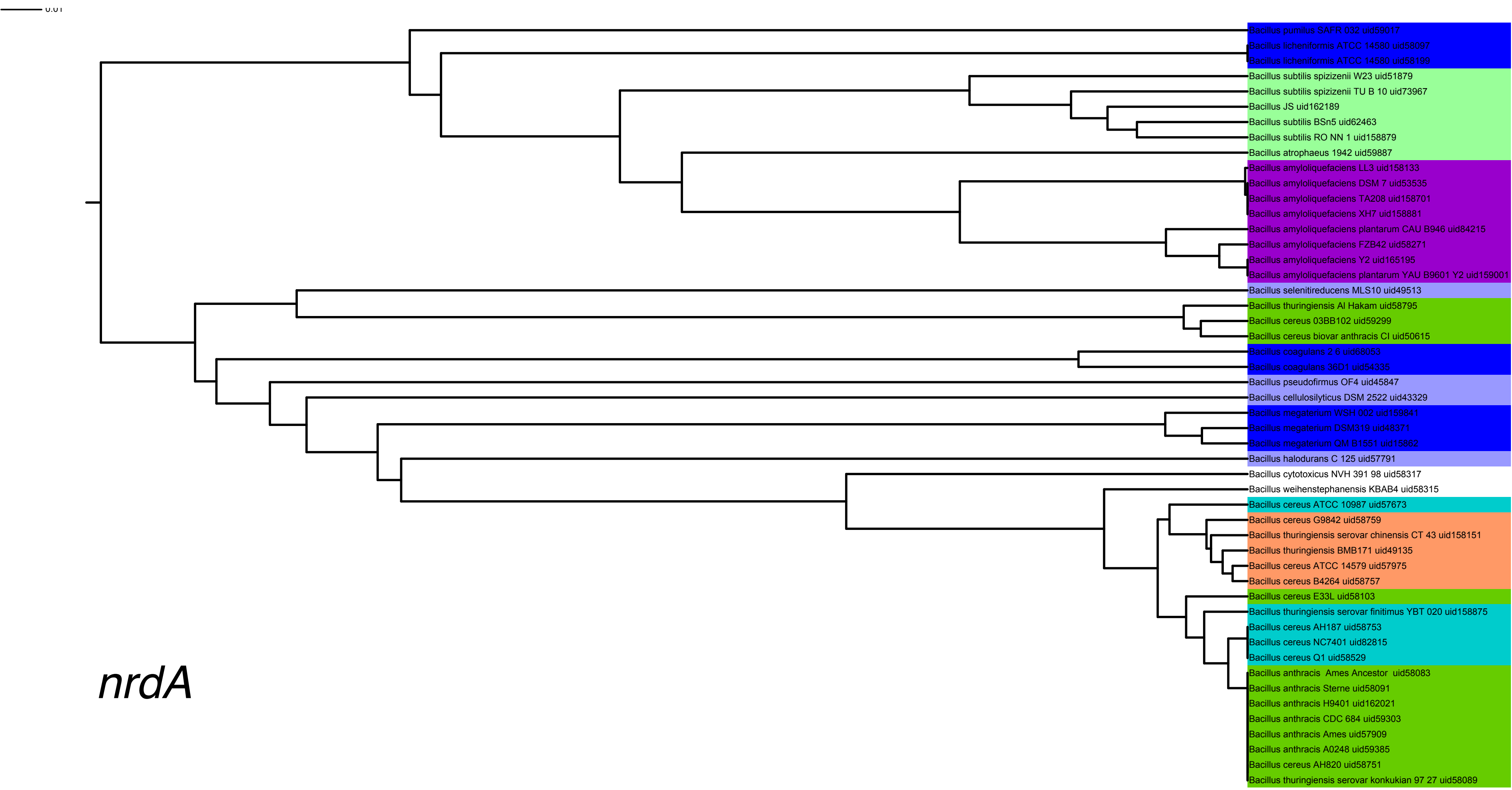
0.01
Bacillus pumilus SAFR 032 uid59017
Bacillus licheniformis ATCC 14580 uid58097
Bacillus licheniformis ATCC 14580 uid58199
Bacillus subtilis spizizenii W23 uid51879
Bacillus subtilis spizizenii TU B 10 uid73967
Bacillus JS uid162189
Bacillus subtilis BSn5 uid62463
Bacillus subtilis RO NN 1 uid158879
Bacillus atrophaeus 1942 uid59887
Bacillus amyloliquefaciens LL3 uid158133
Bacillus amyloliquefaciens DSM 7 uid53535
Bacillus amyloliquefaciens TA208 uid158701
Bacillus amyloliquefaciens XH7 uid158881
Bacillus amyloliquefaciens plantarum CAU B946 uid84215
Bacillus amyloliquefaciens FZB42 uid58271
Bacillus amyloliquefaciens Y2 uid165195
Bacillus amyloliquefaciens plantarum YAU B9601 Y2 uid159001
Bacillus selenitireducens MLS10 uid49513
Bacillus thuringiensis Al Hakam uid58795
Bacillus cereus 03BB102 uid59299
Bacillus cereus biovar anthracis CI uid50615
Bacillus coagulans 2 6 uid68053
Bacillus coagulans 36D1 uid54335
Bacillus pseudofirmus OF4 uid45847
Bacillus cellulosilyticus DSM 2522 uid43329
Bacillus megaterium WSH 002 uid159841
Bacillus megaterium DSM319 uid48371
Bacillus megaterium QM B1551 uid15862
Bacillus halodurans C 125 uid57791
Bacillus cytotoxicus NVH 391 98 uid58317
Bacillus weihenstephanensis KBAB4 uid58315
Bacillus cereus ATCC 10987 uid57673
Bacillus cereus G9842 uid58759
Bacillus thuringiensis serovar chinensis CT 43 uid158151
Bacillus thuringiensis BMB171 uid49135
Bacillus cereus ATCC 14579 uid57975
Bacillus cereus B4264 uid58757
Bacillus cereus E33L uid58103
Bacillus thuringiensis serovar finitimus YBT 020 uid158875
Bacillus cereus AH187 uid58753
Bacillus cereus NC7401 uid82815
Bacillus cereus Q1 uid58529
Bacillus anthracis Ames Ancestor uid58083
Bacillus anthracis Sterne uid58091
Bacillus anthracis H9401 uid162021
Bacillus anthracis CDC 684 uid59303
Bacillus anthracis Ames uid57909
Bacillus anthracis A0248 uid59385
Bacillus cereus AH820 uid58751
Bacillus thuringiensis serovar konkukian 97 27 uid58089
nrdA

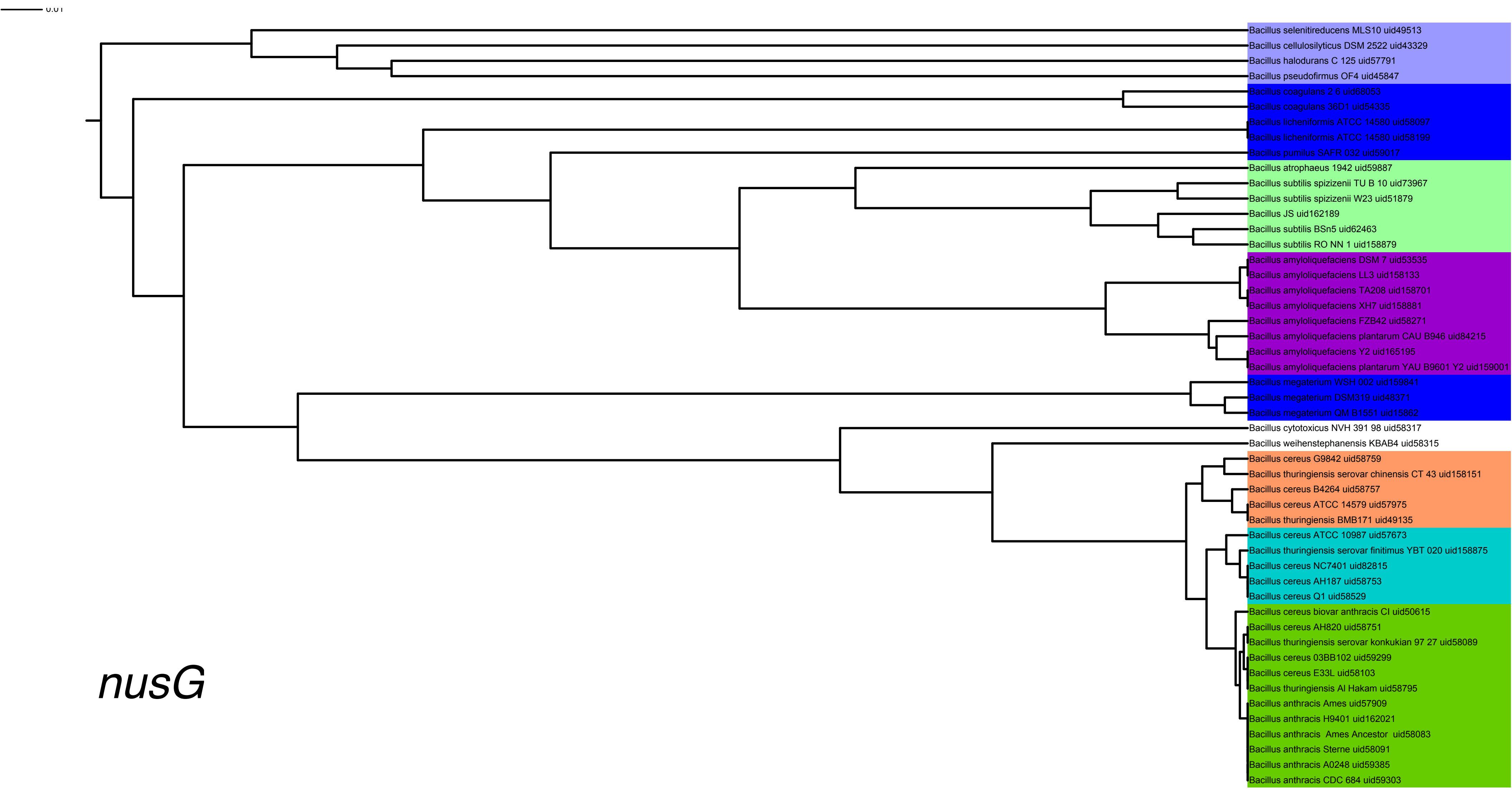
0.01
Bacillus selenitireducens MLS10 uid49513
Bacillus cellulosilyticus DSM 2522 uid43329
Bacillus halodurans C 125 uid57791
Bacillus pseudofirmus OF4 uid45847
Bacillus coagulans 2 6 uid68053
Bacillus coagulans 36D1 uid54335
Bacillus licheniformis ATCC 14580 uid58097
Bacillus licheniformis ATCC 14580 uid58199
Bacillus pumilus SAFR 032 uid59017
Bacillus atrophaeus 1942 uid59887
Bacillus subtilis spizizenii TU B 10 uid73967
Bacillus subtilis spizizenii W23 uid51879
Bacillus JS uid162189
Bacillus subtilis BSn5 uid62463
Bacillus subtilis RO NN 1 uid158879
Bacillus amyloliquefaciens DSM 7 uid53535
Bacillus amyloliquefaciens LL3 uid158133
Bacillus amyloliquefaciens TA208 uid158701
Bacillus amyloliquefaciens XH7 uid158881
Bacillus amyloliquefaciens FZB42 uid58271
Bacillus amyloliquefaciens plantarum CAU B946 uid84215
Bacillus amyloliquefaciens Y2 uid165195
Bacillus amyloliquefaciens plantarum YAU B9601 Y2 uid159001
Bacillus megaterium WSH 002 uid159841
Bacillus megaterium DSM319 uid48371
Bacillus megaterium QM B1551 uid15862
Bacillus cytotoxicus NVH 391 98 uid58317
Bacillus weihenstephanensis KBAB4 uid58315
Bacillus cereus G9842 uid58759
Bacillus thuringiensis serovar chinensis CT 43 uid158151
Bacillus cereus B4264 uid58757
Bacillus cereus ATCC 14579 uid57975
Bacillus thuringiensis BMB171 uid49135
Bacillus cereus ATCC 10987 uid57673
Bacillus thuringiensis serovar finitimus YBT 020 uid158875
Bacillus cereus NC7401 uid82815
Bacillus cereus AH187 uid58753
Bacillus cereus Q1 uid58529
Bacillus cereus biovar anthracis CI uid50615
Bacillus cereus AH820 uid58751
Bacillus thuringiensis serovar konkukian 97 27 uid58089
Bacillus cereus 03BB102 uid59299
Bacillus cereus E33L uid58103
Bacillus thuringiensis Al Hakam uid58795
Bacillus anthracis Ames uid57909
Bacillus anthracis H9401 uid162021
Bacillus anthracis Ames Ancestor uid58083
Bacillus anthracis Sterne uid58091
Bacillus anthracis A0248 uid59385
Bacillus anthracis CDC 684 uid59303
nusG

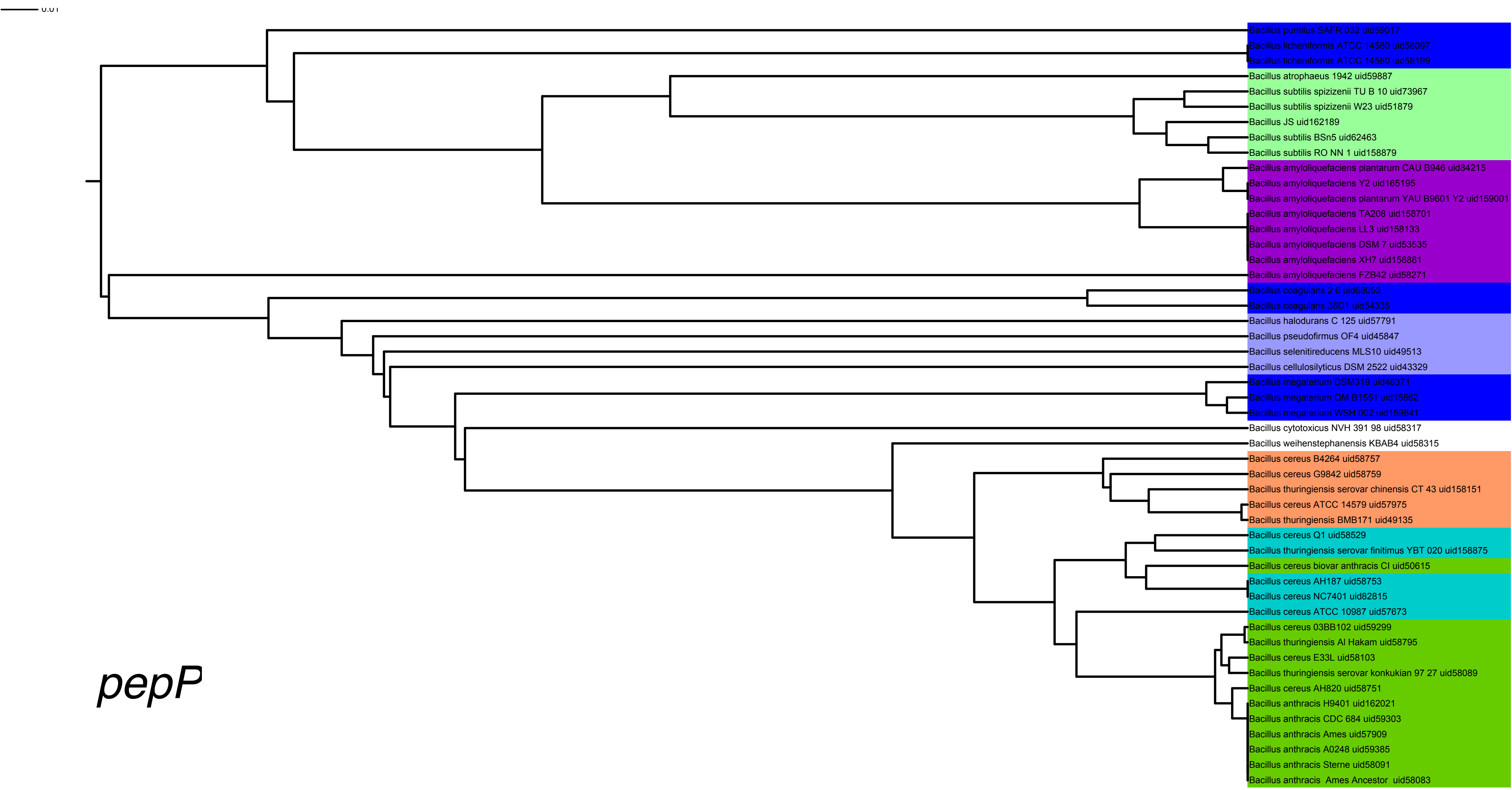
0.01
Bacillus pumilus SAFR 032 uid59017
Bacillus licheniformis ATCC 14580 uid58097
Bacillus licheniformis ATCC 14580 uid58199
Bacillus atrophaeus 1942 uid59887
Bacillus subtilis spizizenii TU B 10 uid73967
Bacillus subtilis spizizenii W23 uid51879
Bacillus JS uid162189
Bacillus subtilis BSn5 uid62463
Bacillus subtilis RO NN 1 uid158879
Bacillus amyloliquefaciens plantarum CAU B946 uid84215
Bacillus amyloliquefaciens Y2 uid165195
Bacillus amyloliquefaciens plantarum YAU B9601 Y2 uid159001
Bacillus amyloliquefaciens TA208 uid158701
Bacillus amyloliquefaciens LL3 uid158133
Bacillus amyloliquefaciens DSM 7 uid53535
Bacillus amyloliquefaciens XH7 uid158881
Bacillus amyloliquefaciens FZB42 uid58271
Bacillus coagulans 2 6 uid68053
Bacillus coagulans 36D1 uid54335
Bacillus halodurans C 125 uid57791
Bacillus pseudofirmus OF4 uid45847
Bacillus selenitireducens MLS10 uid49513
Bacillus cellulosilyticus DSM 2522 uid43329
Bacillus megaterium DSM319 uid48371
Bacillus megaterium QM B1551 uid15862
Bacillus megaterium WSH 002 uid159841
Bacillus cytotoxicus NVH 391 98 uid58317
Bacillus weihenstephanensis KBAB4 uid58315
Bacillus cereus B4264 uid58757
Bacillus cereus G9842 uid58759
Bacillus thuringiensis serovar chinensis CT 43 uid158151
Bacillus cereus ATCC 14579 uid57975
Bacillus thuringiensis BMB171 uid49135
Bacillus cereus Q1 uid58529
Bacillus thuringiensis serovar finitimus YBT 020 uid158875
Bacillus cereus biovar anthracis CI uid50615
Bacillus cereus AH187 uid58753
Bacillus cereus NC7401 uid82815
Bacillus cereus ATCC 10987 uid57673
Bacillus cereus 03BB102 uid59299
Bacillus thuringiensis Al Hakam uid58795
Bacillus cereus E33L uid58103
Bacillus thuringiensis serovar konkukian 97 27 uid58089
Bacillus cereus AH820 uid58751
Bacillus anthracis H9401 uid162021
Bacillus anthracis CDC 684 uid59303
Bacillus anthracis Ames uid57909
Bacillus anthracis A0248 uid59385
Bacillus anthracis Sterne uid58091
Bacillus anthracis  Ames Ancestor  uid58083
pepP

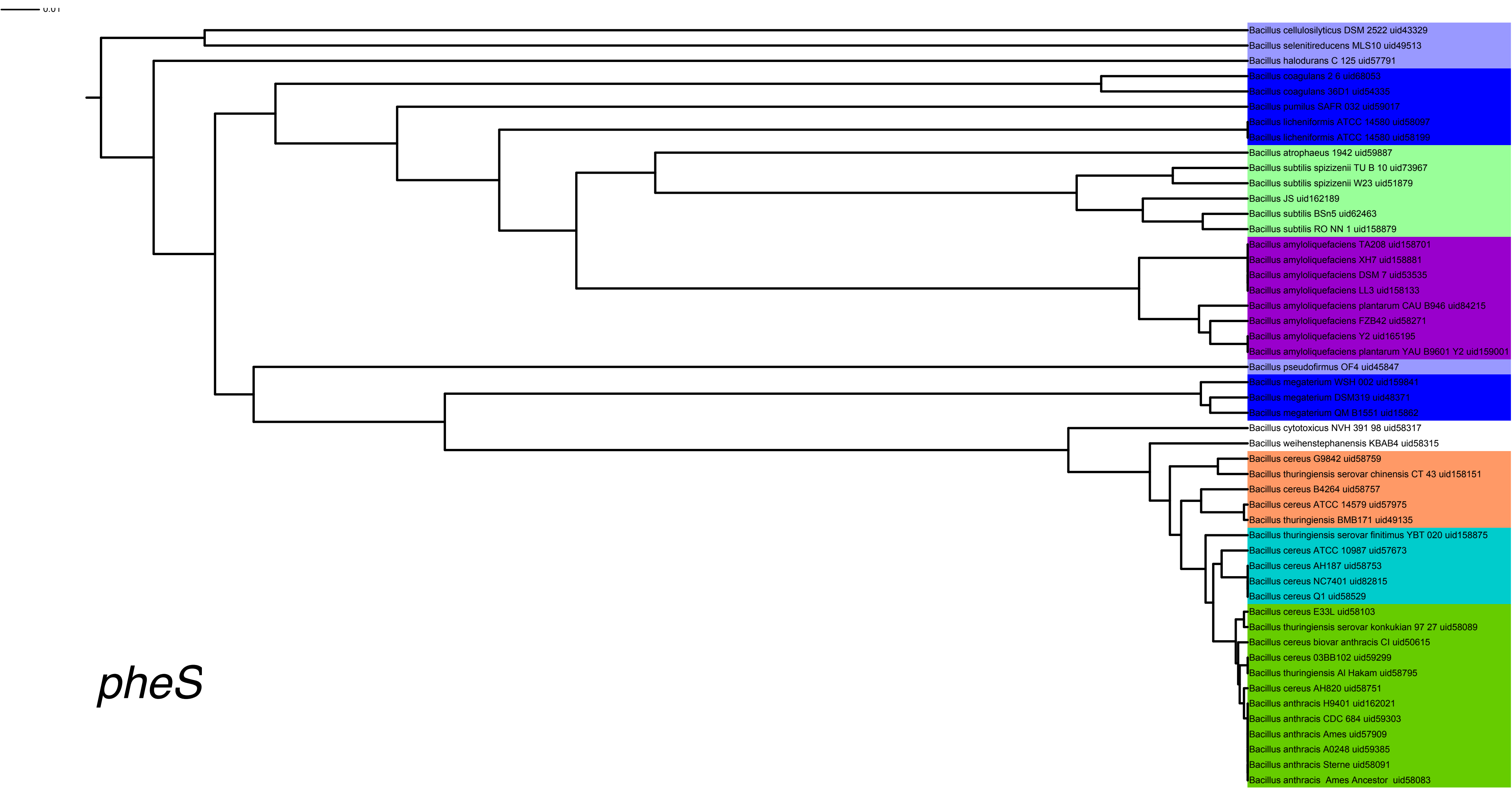
0.01
Bacillus cellulosilyticus DSM 2522 uid43329
Bacillus selenitireducens MLS10 uid49513
Bacillus halodurans C 125 uid57791
Bacillus coagulans 2 6 uid68053
Bacillus coagulans 36D1 uid54335
Bacillus pumilus SAFR 032 uid59017
Bacillus licheniformis ATCC 14580 uid58097
Bacillus licheniformis ATCC 14580 uid58199
Bacillus atrophaeus 1942 uid59887
Bacillus subtilis spizizenii TU B 10 uid73967
Bacillus subtilis spizizenii W23 uid51879
Bacillus JS uid162189
Bacillus subtilis BSn5 uid62463
Bacillus subtilis RO NN 1 uid158879
Bacillus amyloliquefaciens TA208 uid158701
Bacillus amyloliquefaciens XH7 uid158881
Bacillus amyloliquefaciens DSM 7 uid53535
Bacillus amyloliquefaciens LL3 uid158133
Bacillus amyloliquefaciens plantarum CAU B946 uid84215
Bacillus amyloliquefaciens FZB42 uid58271
Bacillus amyloliquefaciens Y2 uid165195
Bacillus amyloliquefaciens plantarum YAU B9601 Y2 uid159001
Bacillus pseudofirmus OF4 uid45847
Bacillus megaterium WSH 002 uid159841
Bacillus megaterium DSM319 uid48371
Bacillus megaterium QM B1551 uid15862
Bacillus cytotoxicus NVH 391 98 uid58317
Bacillus weihenstephanensis KBAB4 uid58315
Bacillus cereus G9842 uid58759
Bacillus thuringiensis serovar chinensis CT 43 uid158151
Bacillus cereus B4264 uid58757
Bacillus cereus ATCC 14579 uid57975
Bacillus thuringiensis BMB171 uid49135
Bacillus thuringiensis serovar finitimus YBT 020 uid158875
Bacillus cereus ATCC 10987 uid57673
Bacillus cereus AH187 uid58753
Bacillus cereus NC7401 uid82815
Bacillus cereus Q1 uid58529
Bacillus cereus E33L uid58103
Bacillus thuringiensis serovar konkukian 97 27 uid58089
Bacillus cereus biovar anthracis CI uid50615
Bacillus cereus 03BB102 uid59299
Bacillus thuringiensis Al Hakam uid58795
Bacillus cereus AH820 uid58751
Bacillus anthracis H9401 uid162021
Bacillus anthracis CDC 684 uid59303
Bacillus anthracis Ames uid57909
Bacillus anthracis A0248 uid59385
Bacillus anthracis Sterne uid58091
Bacillus anthracis  Ames Ancestor  uid58083
pheS

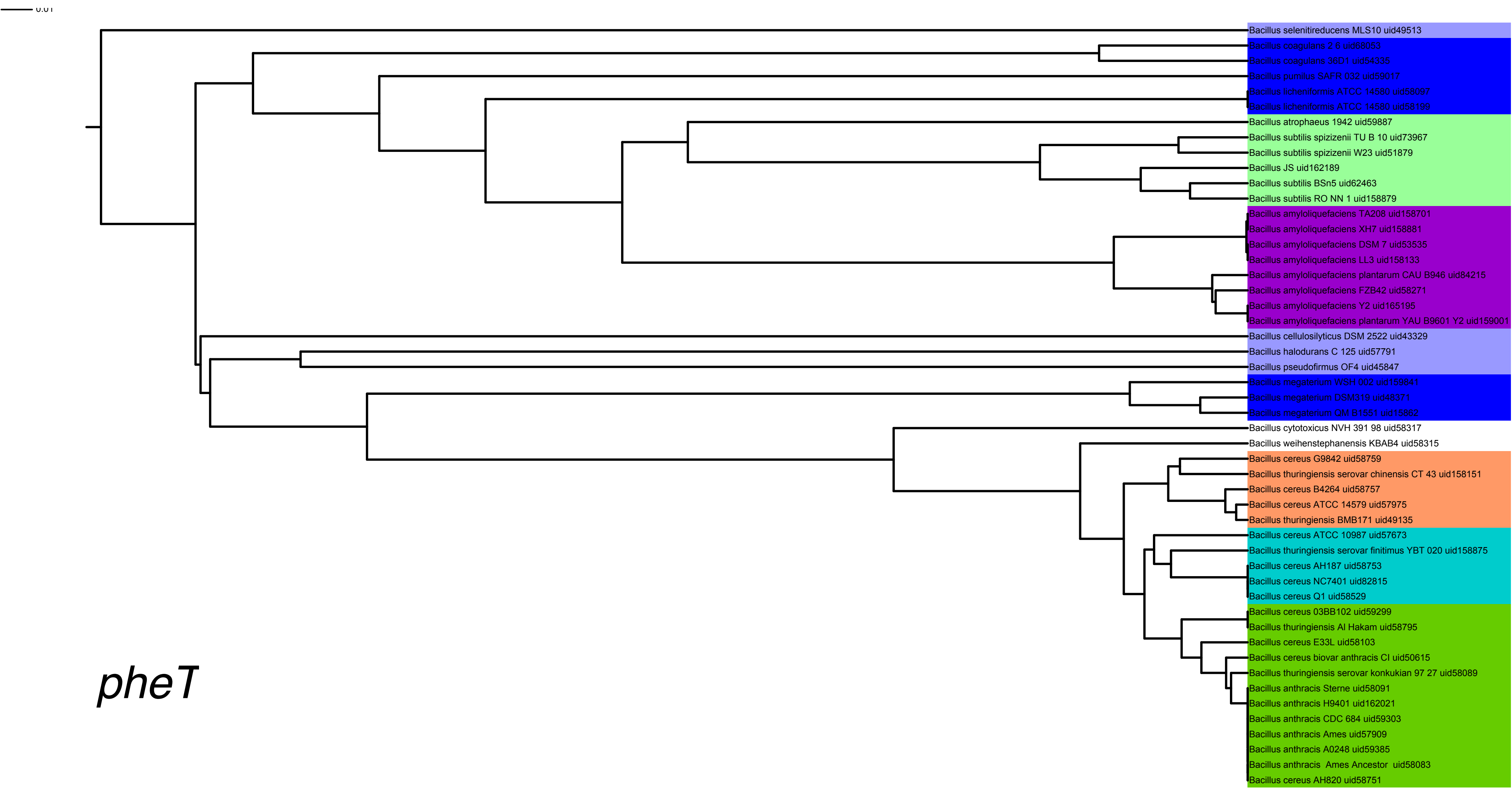
0.01
Bacillus selenitireducens MLS10 uid49513
Bacillus coagulans 2 6 uid68053
Bacillus coagulans 36D1 uid54335
Bacillus pumilus SAFR 032 uid59017
Bacillus licheniformis ATCC 14580 uid58097
Bacillus licheniformis ATCC 14580 uid58199
Bacillus atrophaeus 1942 uid59887
Bacillus subtilis spizizenii TU B 10 uid73967
Bacillus subtilis spizizenii W23 uid51879
Bacillus JS uid162189
Bacillus subtilis BSn5 uid62463
Bacillus subtilis RO NN 1 uid158879
Bacillus amyloliquefaciens TA208 uid158701
Bacillus amyloliquefaciens XH7 uid158881
Bacillus amyloliquefaciens DSM 7 uid53535
Bacillus amyloliquefaciens LL3 uid158133
Bacillus amyloliquefaciens plantarum CAU B946 uid84215
Bacillus amyloliquefaciens FZB42 uid58271
Bacillus amyloliquefaciens Y2 uid165195
Bacillus amyloliquefaciens plantarum YAU B9601 Y2 uid159001
Bacillus cellulosilyticus DSM 2522 uid43329
Bacillus halodurans C 125 uid57791
Bacillus pseudofirmus OF4 uid45847
Bacillus megaterium WSH 002 uid159841
Bacillus megaterium DSM319 uid48371
Bacillus megaterium QM B1551 uid15862
Bacillus cytotoxicus NVH 391 98 uid58317
Bacillus weihenstephanensis KBAB4 uid58315
Bacillus cereus G9842 uid58759
Bacillus thuringiensis serovar chinensis CT 43 uid158151
Bacillus cereus B4264 uid58757
Bacillus cereus ATCC 14579 uid57975
Bacillus thuringiensis BMB171 uid49135
Bacillus cereus ATCC 10987 uid57673
Bacillus thuringiensis serovar finitimus YBT 020 uid158875
Bacillus cereus AH187 uid58753
Bacillus cereus NC7401 uid82815
Bacillus cereus Q1 uid58529
Bacillus cereus 03BB102 uid59299
Bacillus thuringiensis Al Hakam uid58795
Bacillus cereus E33L uid58103
Bacillus cereus biovar anthracis CI uid50615
Bacillus thuringiensis serovar konkukian 97 27 uid58089
Bacillus anthracis Sterne uid58091
Bacillus anthracis H9401 uid162021
Bacillus anthracis CDC 684 uid59303
Bacillus anthracis Ames uid57909
Bacillus anthracis A0248 uid59385
Bacillus anthracis  Ames Ancestor  uid58083
Bacillus cereus AH820 uid58751
pheT

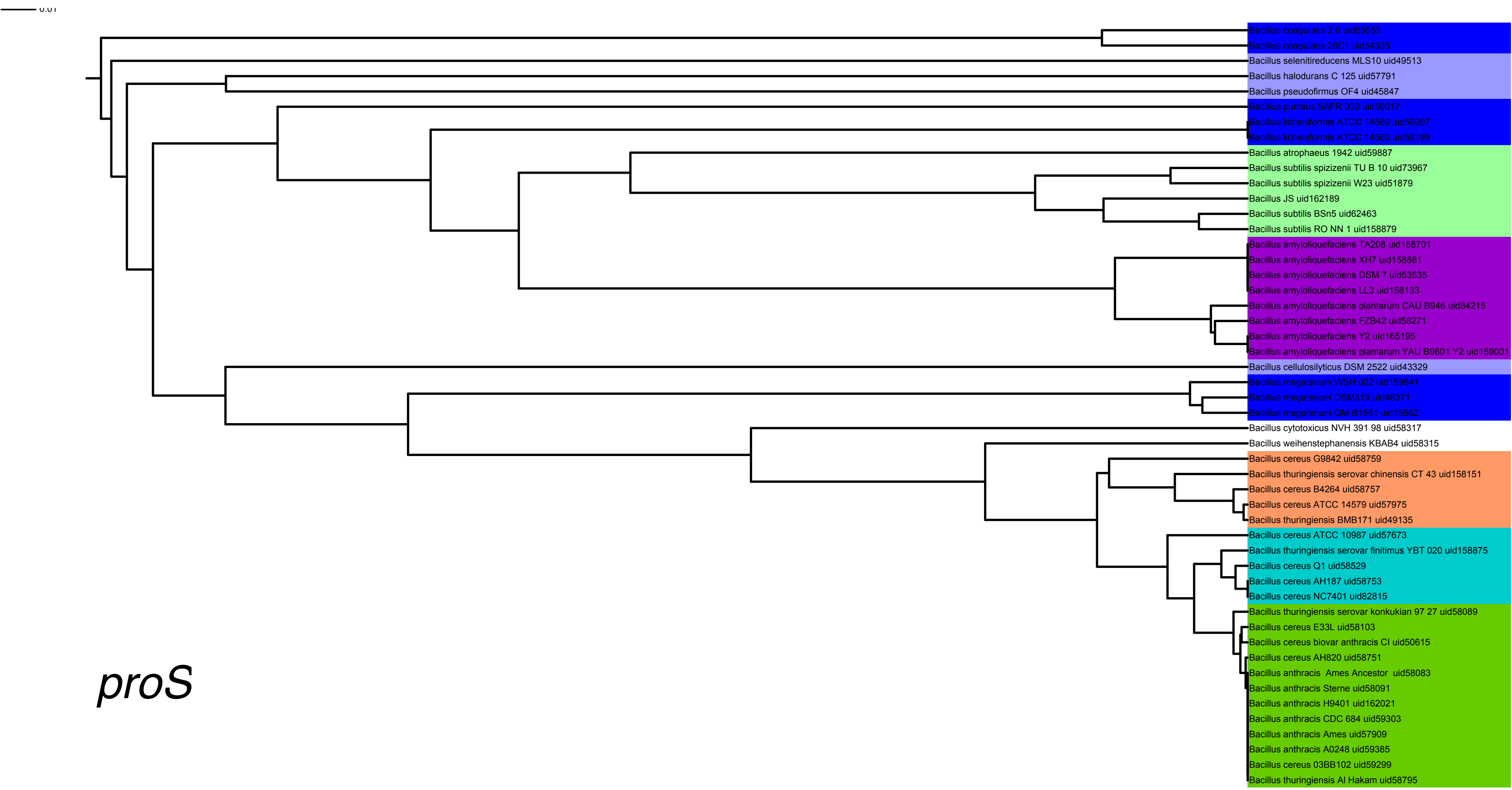

0.01
Bacillus coagulans 2 6 uid68053
Bacillus coagulans 36D1 uid54335
Bacillus selenitireducens MLS10 uid49513
Bacillus halodurans C 125 uid57791
Bacillus pseudofirmus OF4 uid45847
Bacillus pumilus SAFR 032 uid59017
Bacillus licheniformis ATCC 14580 uid58097
Bacillus licheniformis ATCC 14580 uid58199
Bacillus atrophaeus 1942 uid59887
Bacillus subtilis spizizenii TU B 10 uid73967
Bacillus subtilis spizizenii W23 uid51879
Bacillus JS uid162189
Bacillus subtilis BSn5 uid62463
Bacillus subtilis RO NN 1 uid158879
Bacillus amyloliquefaciens TA208 uid158701
Bacillus amyloliquefaciens XH7 uid158881
Bacillus amyloliquefaciens DSM 7 uid53535
Bacillus amyloliquefaciens LL3 uid158133
Bacillus amyloliquefaciens plantarum CAU B946 uid84215
Bacillus amyloliquefaciens FZB42 uid58271
Bacillus amyloliquefaciens Y2 uid165195
Bacillus amyloliquefaciens plantarum YAU B9601 Y2 uid159001
Bacillus cellulosilyticus DSM 2522 uid43329
Bacillus megaterium WSH 002 uid159841
Bacillus megaterium DSM319 uid48371
Bacillus megaterium QM B1551 uid15862
Bacillus cytotoxicus NVH 391 98 uid58317
Bacillus weihenstephanensis KBAB4 uid58315
Bacillus cereus G9842 uid58759
Bacillus thuringiensis serovar chinensis CT 43 uid158151
Bacillus cereus B4264 uid58757
Bacillus cereus ATCC 14579 uid57975
Bacillus thuringiensis BMB171 uid49135
Bacillus cereus ATCC 10987 uid57673
Bacillus thuringiensis serovar finitimus YBT 020 uid158875
Bacillus cereus Q1 uid58529
Bacillus cereus AH187 uid58753
Bacillus cereus NC7401 uid82815
Bacillus thuringiensis serovar konkukian 97 27 uid58089
Bacillus cereus E33L uid58103
Bacillus cereus biovar anthracis CI uid50615
Bacillus cereus AH820 uid58751
Bacillus anthracis Ames Ancestor uid58083
Bacillus anthracis Sterne uid58091
Bacillus anthracis H9401 uid162021
Bacillus anthracis CDC 684 uid59303
Bacillus anthracis Ames uid57909
Bacillus anthracis A0248 uid59385
Bacillus cereus 03BB102 uid59299
Bacillus thuringiensis Al Hakam uid58795
proS

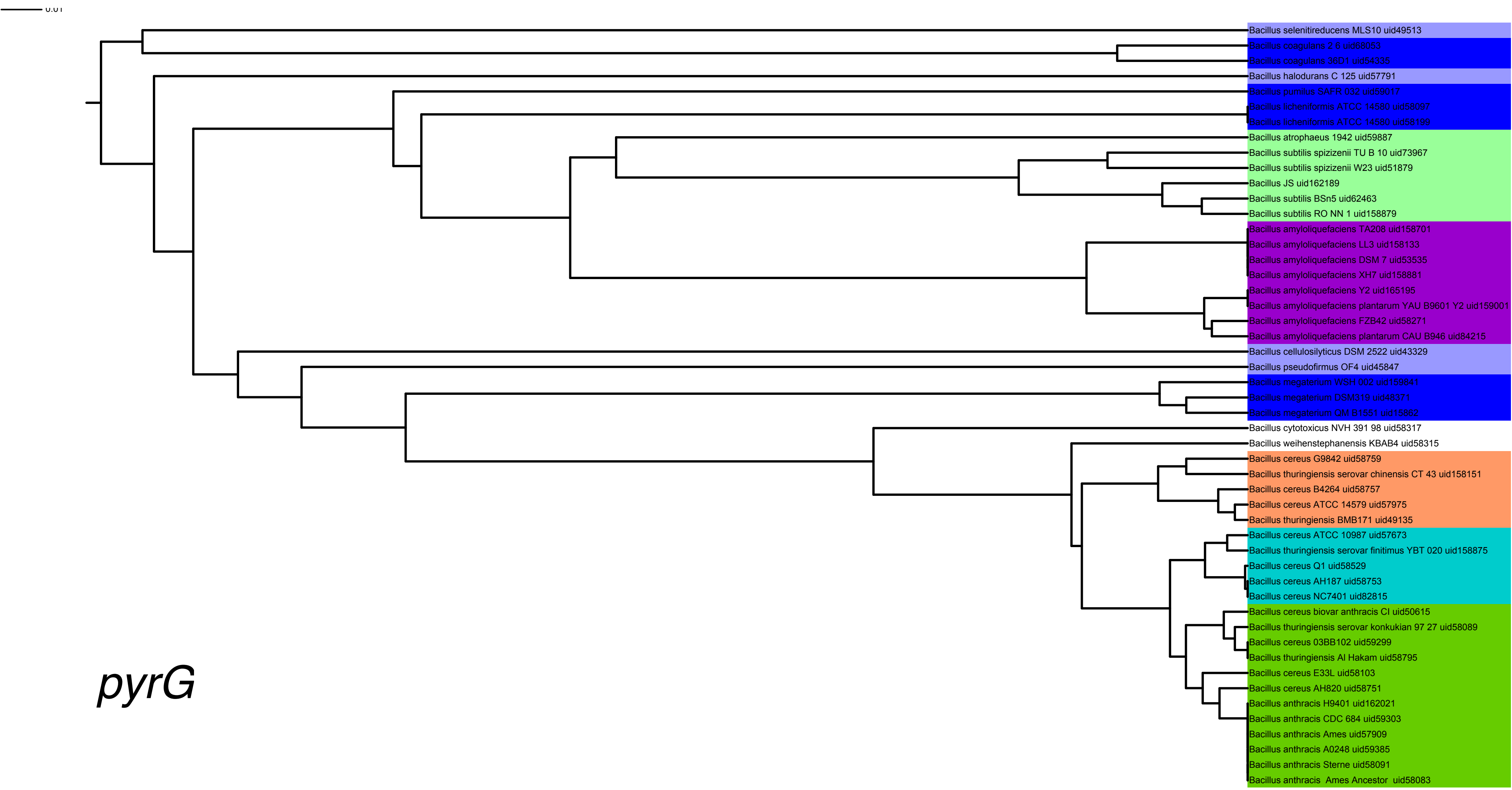
0.01
Bacillus selenitireducens MLS10 uid49513
Bacillus coagulans 2 6 uid68053
Bacillus coagulans 36D1 uid54335
Bacillus halodurans C 125 uid57791
Bacillus pumilus SAFR 032 uid59017
Bacillus licheniformis ATCC 14580 uid58097
Bacillus licheniformis ATCC 14580 uid58199
Bacillus atrophaeus 1942 uid59887
Bacillus subtilis spizizenii TU B 10 uid73967
Bacillus subtilis spizizenii W23 uid51879
Bacillus JS uid162189
Bacillus subtilis BSn5 uid62463
Bacillus subtilis RO NN 1 uid158879
Bacillus amyloliquefaciens TA208 uid158701
Bacillus amyloliquefaciens LL3 uid158133
Bacillus amyloliquefaciens DSM 7 uid53535
Bacillus amyloliquefaciens XH7 uid158881
Bacillus amyloliquefaciens Y2 uid165195
Bacillus amyloliquefaciens plantarum YAU B9601 Y2 uid159001
Bacillus amyloliquefaciens FZB42 uid58271
Bacillus amyloliquefaciens plantarum CAU B946 uid84215
Bacillus cellulosilyticus DSM 2522 uid43329
Bacillus pseudofirmus OF4 uid45847
Bacillus megaterium WSH 002 uid159841
Bacillus megaterium DSM319 uid48371
Bacillus megaterium QM B1551 uid15862
Bacillus cytotoxicus NVH 391 98 uid58317
Bacillus weihenstephanensis KBAB4 uid58315
Bacillus cereus G9842 uid58759
Bacillus thuringiensis serovar chinensis CT 43 uid158151
Bacillus cereus B4264 uid58757
Bacillus cereus ATCC 14579 uid57975
Bacillus thuringiensis BMB171 uid49135
Bacillus cereus ATCC 10987 uid57673
Bacillus thuringiensis serovar finitimus YBT 020 uid158875
Bacillus cereus Q1 uid58529
Bacillus cereus AH187 uid58753
Bacillus cereus NC7401 uid82815
Bacillus cereus biovar anthracis CI uid50615
Bacillus thuringiensis serovar konkukian 97 27 uid58089
Bacillus cereus 03BB102 uid59299
Bacillus thuringiensis Al Hakam uid58795
Bacillus cereus E33L uid58103
Bacillus cereus AH820 uid58751
Bacillus anthracis H9401 uid162021
Bacillus anthracis CDC 684 uid59303
Bacillus anthracis Ames uid57909
Bacillus anthracis A0248 uid59385
Bacillus anthracis Sterne uid58091
Bacillus anthracis Ames Ancestor uid58083
pyrG

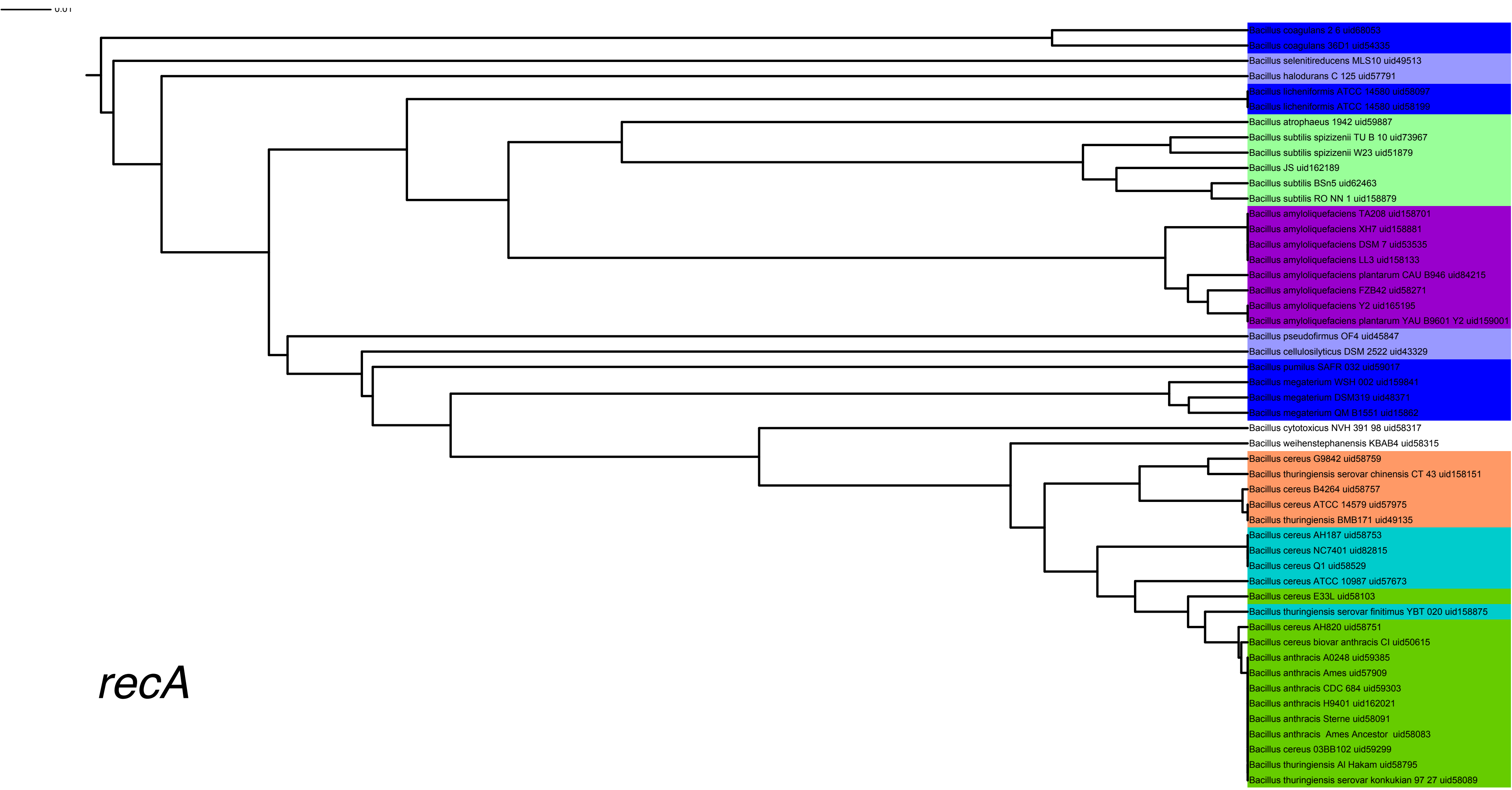
0.01
Bacillus coagulans 2 6 uid68053
Bacillus coagulans 36D1 uid54335
Bacillus selenitireducens MLS10 uid49513
Bacillus halodurans C 125 uid57791
Bacillus licheniformis ATCC 14580 uid58097
Bacillus licheniformis ATCC 14580 uid58199
Bacillus atrophaeus 1942 uid59887
Bacillus subtilis spizizenii TU B 10 uid73967
Bacillus subtilis spizizenii W23 uid51879
Bacillus JS uid162189
Bacillus subtilis BSn5 uid62463
Bacillus subtilis RO NN 1 uid158879
Bacillus amyloliquefaciens TA208 uid158701
Bacillus amyloliquefaciens XH7 uid158881
Bacillus amyloliquefaciens DSM 7 uid53535
Bacillus amyloliquefaciens LL3 uid158133
Bacillus amyloliquefaciens plantarum CAU B946 uid84215
Bacillus amyloliquefaciens FZB42 uid58271
Bacillus amyloliquefaciens Y2 uid165195
Bacillus amyloliquefaciens plantarum YAU B9601 Y2 uid159001
Bacillus pseudofirmus OF4 uid45847
Bacillus cellulosilyticus DSM 2522 uid43329
Bacillus pumilus SAFR 032 uid59017
Bacillus megaterium WSH 002 uid159841
Bacillus megaterium DSM319 uid48371
Bacillus megaterium QM B1551 uid15862
Bacillus cytotoxicus NVH 391 98 uid58317
Bacillus weihenstephanensis KBAB4 uid58315
Bacillus cereus G9842 uid58759
Bacillus thuringiensis serovar chinensis CT 43 uid158151
Bacillus cereus B4264 uid58757
Bacillus cereus ATCC 14579 uid57975
Bacillus thuringiensis BMB171 uid49135
Bacillus cereus AH187 uid58753
Bacillus cereus NC7401 uid82815
Bacillus cereus Q1 uid58529
Bacillus cereus ATCC 10987 uid57673
Bacillus cereus E33L uid58103
Bacillus thuringiensis serovar finitimus YBT 020 uid158875
Bacillus cereus AH820 uid58751
Bacillus cereus biovar anthracis CI uid50615
Bacillus anthracis A0248 uid59385
Bacillus anthracis Ames uid57909
Bacillus anthracis CDC 684 uid59303
Bacillus anthracis H9401 uid162021
Bacillus anthracis Sterne uid58091
Bacillus anthracis Ames Ancestor uid58083
Bacillus cereus 03BB102 uid59299
Bacillus thuringiensis Al Hakam uid58795
Bacillus thuringiensis serovar konkukian 97 27 uid58089
recA

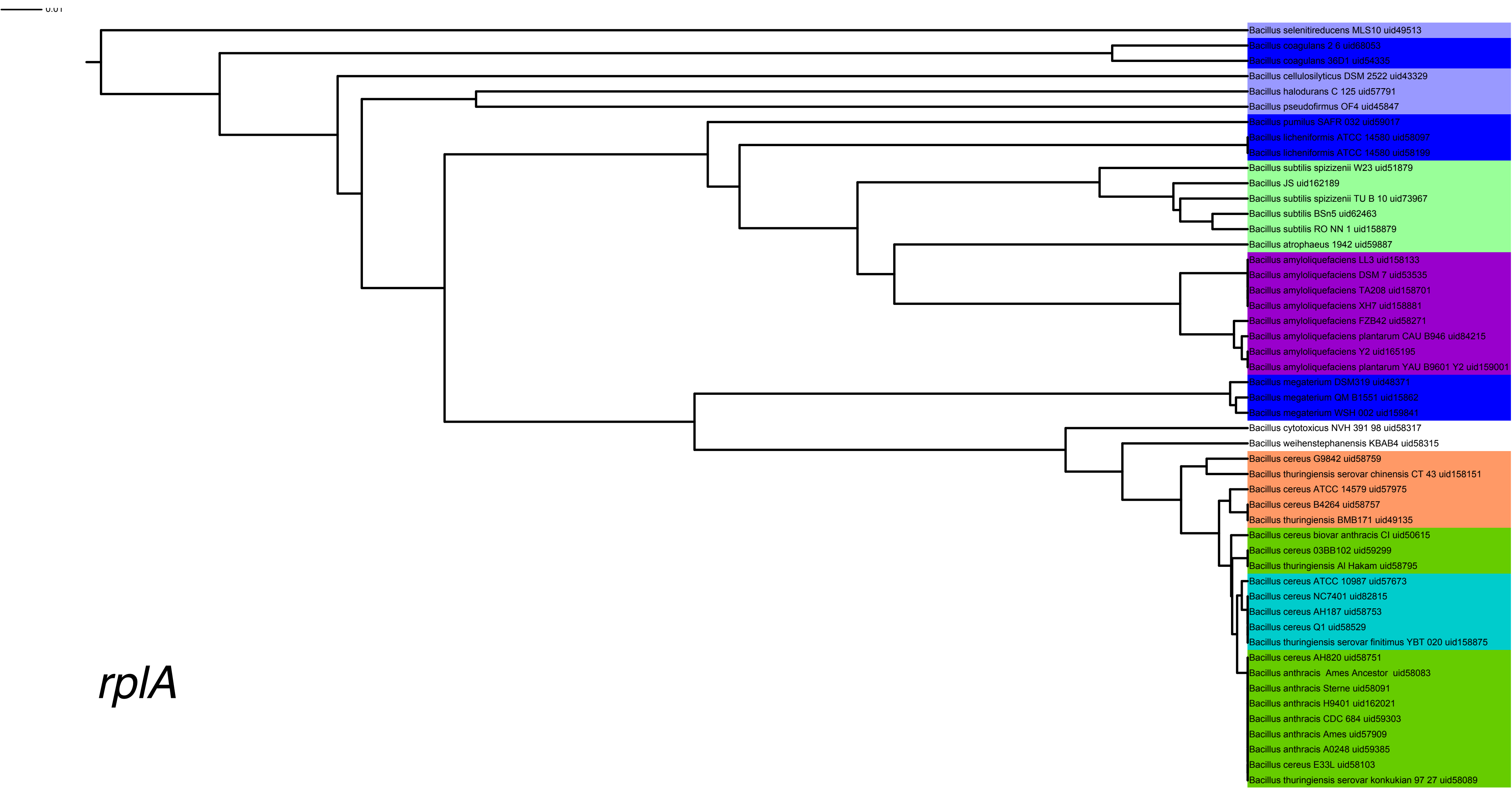
0.01
Bacillus selenitireducens MLS10 uid49513
Bacillus coagulans 2 6 uid68053
Bacillus coagulans 36D1 uid54335
Bacillus cellulosilyticus DSM 2522 uid43329
Bacillus halodurans C 125 uid57791
Bacillus pseudofirmus OF4 uid45847
Bacillus pumilus SAFR 032 uid59017
Bacillus licheniformis ATCC 14580 uid58097
Bacillus licheniformis ATCC 14580 uid58199
Bacillus subtilis spizizenii W23 uid51879
Bacillus JS uid162189
Bacillus subtilis spizizenii TU B 10 uid73967
Bacillus subtilis BSn5 uid62463
Bacillus subtilis RO NN 1 uid158879
Bacillus atrophaeus 1942 uid59887
Bacillus amyloliquefaciens LL3 uid158133
Bacillus amyloliquefaciens DSM 7 uid53535
Bacillus amyloliquefaciens TA208 uid158701
Bacillus amyloliquefaciens XH7 uid158881
Bacillus amyloliquefaciens FZB42 uid58271
Bacillus amyloliquefaciens plantarum CAU B946 uid84215
Bacillus amyloliquefaciens Y2 uid165195
Bacillus amyloliquefaciens plantarum YAU B9601 Y2 uid159001
Bacillus megaterium DSM319 uid48371
Bacillus megaterium QM B1551 uid15862
Bacillus megaterium WSH 002 uid159841
Bacillus cytotoxicus NVH 391 98 uid58317
Bacillus weihenstephanensis KBAB4 uid58315
Bacillus cereus G9842 uid58759
Bacillus thuringiensis serovar chinensis CT 43 uid158151
Bacillus cereus ATCC 14579 uid57975
Bacillus cereus B4264 uid58757
Bacillus thuringiensis BMB171 uid49135
Bacillus cereus biovar anthracis CI uid50615
Bacillus cereus 03BB102 uid59299
Bacillus thuringiensis Al Hakam uid58795
Bacillus cereus ATCC 10987 uid57673
Bacillus cereus NC7401 uid82815
Bacillus cereus AH187 uid58753
Bacillus cereus Q1 uid58529
Bacillus thuringiensis serovar finitimus YBT 020 uid158875
Bacillus cereus AH820 uid58751
Bacillus anthracis Ames Ancestor uid58083
Bacillus anthracis Sterne uid58091
Bacillus anthracis H9401 uid162021
Bacillus anthracis CDC 684 uid59303
Bacillus anthracis Ames uid57909
Bacillus anthracis A0248 uid59385
Bacillus cereus E33L uid58103
Bacillus thuringiensis serovar konkukian 97 27 uid58089
rplA

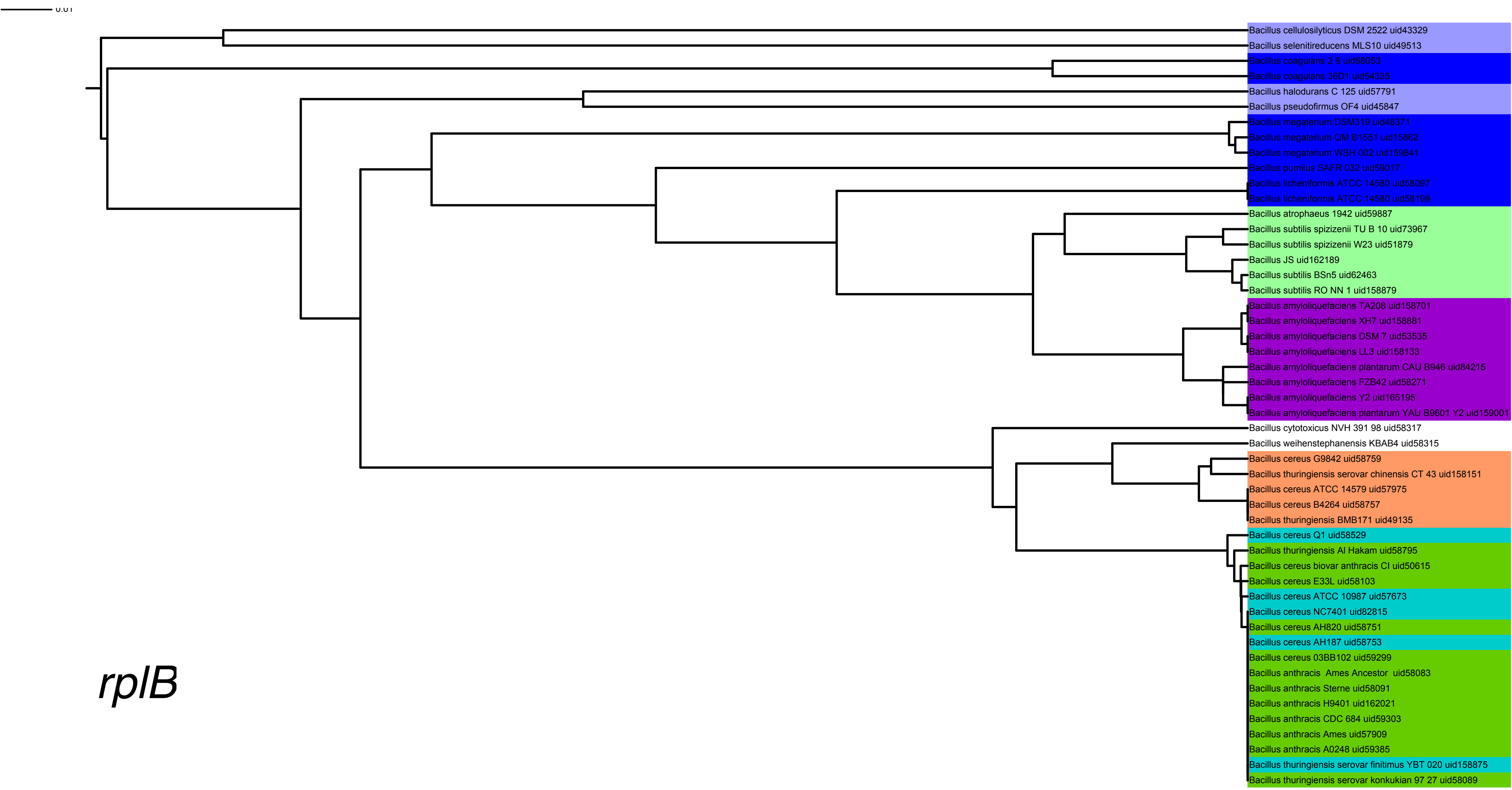
0.01
Bacillus cellulosilyticus DSM 2522 uid43329
Bacillus selenitireducens MLS10 uid49513
Bacillus coagulans 2 6 uid68053
Bacillus coagulans 36D1 uid54335
Bacillus halodurans C 125 uid57791
Bacillus pseudofirmus OF4 uid45847
Bacillus megaterium DSM319 uid48371
Bacillus megaterium QM B1551 uid15862
Bacillus megaterium WSH 002 uid159841
Bacillus pumilus SAFR 032 uid59017
Bacillus licheniformis ATCC 14580 uid58097
Bacillus licheniformis ATCC 14580 uid58199
Bacillus atrophaeus 1942 uid59887
Bacillus subtilis spizizenii TU B 10 uid73967
Bacillus subtilis spizizenii W23 uid51879
Bacillus JS uid162189
Bacillus subtilis BSn5 uid62463
Bacillus subtilis RO NN 1 uid158879
Bacillus amyloliquefaciens TA208 uid158701
Bacillus amyloliquefaciens XH7 uid158881
Bacillus amyloliquefaciens DSM 7 uid53535
Bacillus amyloliquefaciens LL3 uid158133
Bacillus amyloliquefaciens plantarum CAU B946 uid84215
Bacillus amyloliquefaciens FZB42 uid58271
Bacillus amyloliquefaciens Y2 uid165195
Bacillus amyloliquefaciens plantarum YAU B9601 Y2 uid159001
Bacillus cytotoxicus NVH 391 98 uid58317
Bacillus weihenstephanensis KBAB4 uid58315
Bacillus cereus G9842 uid58759
Bacillus thuringiensis serovar chinensis CT 43 uid158151
Bacillus cereus ATCC 14579 uid57975
Bacillus cereus B4264 uid58757
Bacillus thuringiensis BMB171 uid49135
Bacillus cereus Q1 uid58529
Bacillus thuringiensis Al Hakam uid58795
Bacillus cereus biovar anthracis CI uid50615
Bacillus cereus E33L uid58103
Bacillus cereus ATCC 10987 uid57673
Bacillus cereus NC7401 uid82815
Bacillus cereus AH820 uid58751
Bacillus cereus AH187 uid58753
Bacillus cereus 03BB102 uid59299
Bacillus anthracis Ames Ancestor uid58083
Bacillus anthracis Sterne uid58091
Bacillus anthracis H9401 uid162021
Bacillus anthracis CDC 684 uid59303
Bacillus anthracis Ames uid57909
Bacillus anthracis A0248 uid59385
Bacillus thuringiensis serovar finitimus YBT 020 uid158875
Bacillus thuringiensis serovar konkukian 97 27 uid58089
rplB

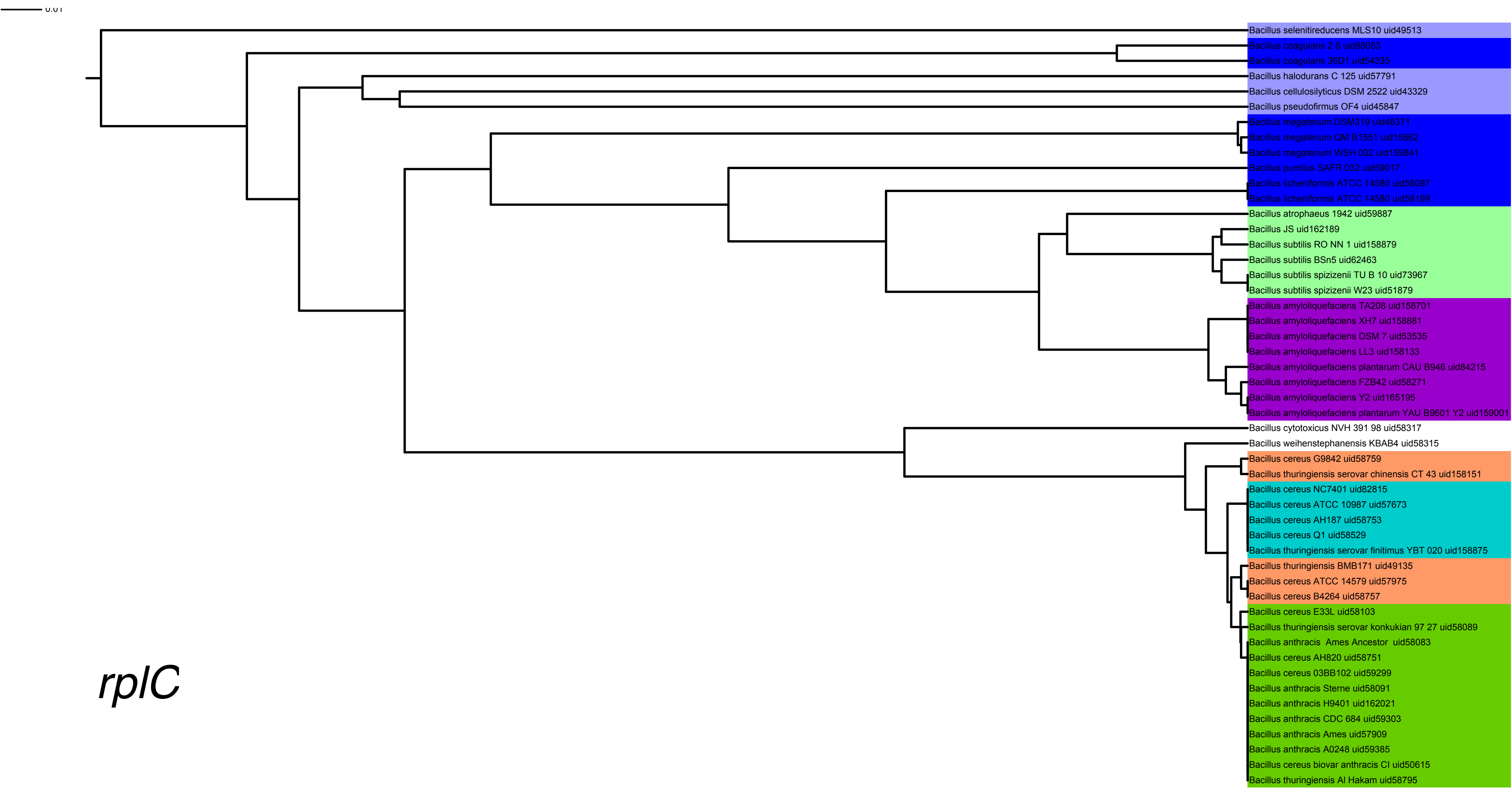
0.01
Bacillus selenitireducens MLS10 uid49513
Bacillus coagulans 2 6 uid68053
Bacillus coagulans 36D1 uid54335
Bacillus halodurans C 125 uid57791
Bacillus cellulosilyticus DSM 2522 uid43329
Bacillus pseudofirmus OF4 uid45847
Bacillus megaterium DSM319 uid48371
Bacillus megaterium QM B1551 uid15862
Bacillus megaterium WSH 002 uid159841
Bacillus pumilus SAFR 032 uid59017
Bacillus licheniformis ATCC 14580 uid58097
Bacillus licheniformis ATCC 14580 uid58199
Bacillus atrophaeus 1942 uid59887
Bacillus JS uid162189
Bacillus subtilis RO NN 1 uid158879
Bacillus subtilis BSn5 uid62463
Bacillus subtilis spizizenii TU B 10 uid73967
Bacillus subtilis spizizenii W23 uid51879
Bacillus amyloliquefaciens TA208 uid158701
Bacillus amyloliquefaciens XH7 uid158881
Bacillus amyloliquefaciens DSM 7 uid53535
Bacillus amyloliquefaciens LL3 uid158133
Bacillus amyloliquefaciens plantarum CAU B946 uid84215
Bacillus amyloliquefaciens FZB42 uid58271
Bacillus amyloliquefaciens Y2 uid165195
Bacillus amyloliquefaciens plantarum YAU B9601 Y2 uid159001
Bacillus cytotoxicus NVH 391 98 uid58317
Bacillus weihenstephanensis KBAB4 uid58315
Bacillus cereus G9842 uid58759
Bacillus thuringiensis serovar chinensis CT 43 uid158151
Bacillus cereus NC7401 uid82815
Bacillus cereus ATCC 10987 uid57673
Bacillus cereus AH187 uid58753
Bacillus cereus Q1 uid58529
Bacillus thuringiensis serovar finitimus YBT 020 uid158875
Bacillus thuringiensis BMB171 uid49135
Bacillus cereus ATCC 14579 uid57975
Bacillus cereus B4264 uid58757
Bacillus cereus E33L uid58103
Bacillus thuringiensis serovar konkukian 97 27 uid58089
Bacillus anthracis Ames Ancestor uid58083
Bacillus cereus AH820 uid58751
Bacillus cereus 03BB102 uid59299
Bacillus anthracis Sterne uid58091
Bacillus anthracis H9401 uid162021
Bacillus anthracis CDC 684 uid59303
Bacillus anthracis Ames uid57909
Bacillus anthracis A0248 uid59385
Bacillus cereus biovar anthracis CI uid50615
Bacillus thuringiensis Al Hakam uid58795
rplC

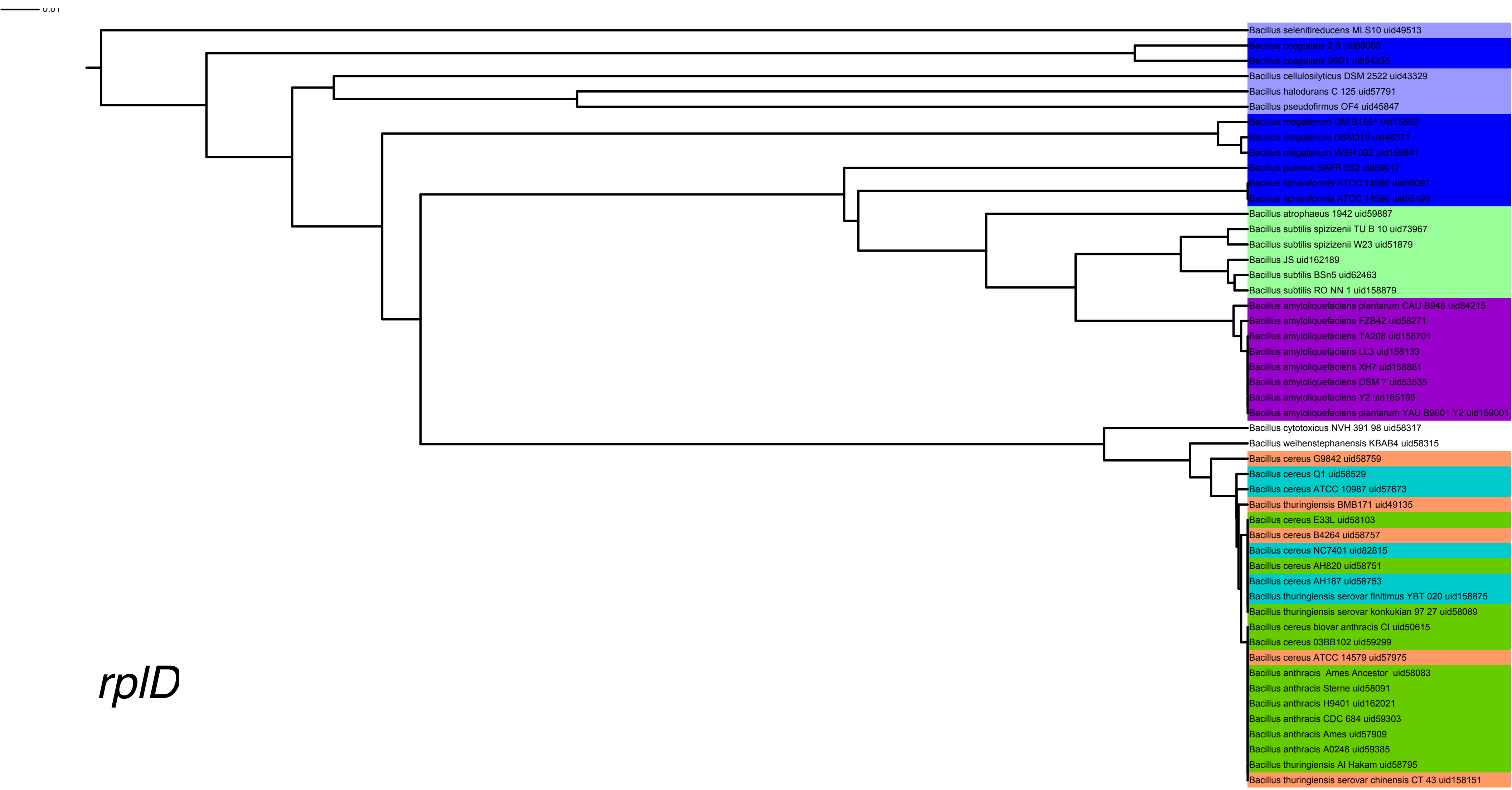
Bacillus selenitireducens MLS10 uid49513
Bacillus coagulans 2 6 uid68053
Bacillus coagulans 36D1 uid54335
Bacillus cellulosilyticus DSM 2522 uid43329
Bacillus halodurans C 125 uid57791
Bacillus pseudofirmus OF4 uid45847
Bacillus megaterium QM B1551 uid48363
Bacillus megaterium DSM319 uid48371
Bacillus megaterium WSH 002 uid159841
Bacillus pumilus SAFR 032 uid59017
Bacillus licheniformis ATCC 14580 uid58097
Bacillus licheniformis ATCC 14580 uid58199
Bacillus atrophaeus 1942 uid59887
Bacillus subtilis spizizenii TU B 10 uid73967
Bacillus subtilis spizizenii W23 uid51879
Bacillus JS uid162189
Bacillus subtilis BSn5 uid62463
Bacillus subtilis RO NN 1 uid158879
Bacillus amyloliquefaciens plantarum CAU B946 uid84215
Bacillus amyloliquefaciens FZB42 uid58271
Bacillus amyloliquefaciens TA208 uid158701
Bacillus amyloliquefaciens LL3 uid158133
Bacillus amyloliquefaciens XH7 uid158881
Bacillus amyloliquefaciens DSM 7 uid53535
Bacillus amyloliquefaciens Y2 uid165195
Bacillus amyloliquefaciens plantarum YAU B9601 Y2 uid159001
Bacillus cytotoxicus NVH 391 98 uid58317
Bacillus weihenstephanensis KBAB4 uid58315
Bacillus cereus G9842 uid58759
Bacillus cereus Q1 uid58529
Bacillus cereus ATCC 10987 uid57673
Bacillus thuringiensis BMB171 uid49135
Bacillus cereus E33L uid58103
Bacillus cereus B4264 uid58757
Bacillus cereus NC7401 uid82815
Bacillus cereus AH820 uid58751
Bacillus cereus AH187 uid58753
Bacillus thuringiensis serovar finitimus YBT 020 uid158875
Bacillus thuringiensis serovar konkukian 97 27 uid58089
Bacillus cereus biovar anthracis CI uid50615
Bacillus cereus 03BB102 uid59299
Bacillus cereus ATCC 14579 uid57975
Bacillus anthracis Ames Ancestor uid58083
Bacillus anthracis Sterne uid58091
Bacillus anthracis H9401 uid162021
Bacillus anthracis CDC 684 uid59303
Bacillus anthracis Ames uid57909
Bacillus anthracis A0248 uid59385
Bacillus thuringiensis Al Hakam uid58795
Bacillus thuringiensis serovar chinensis CT 43 uid158151
rplD

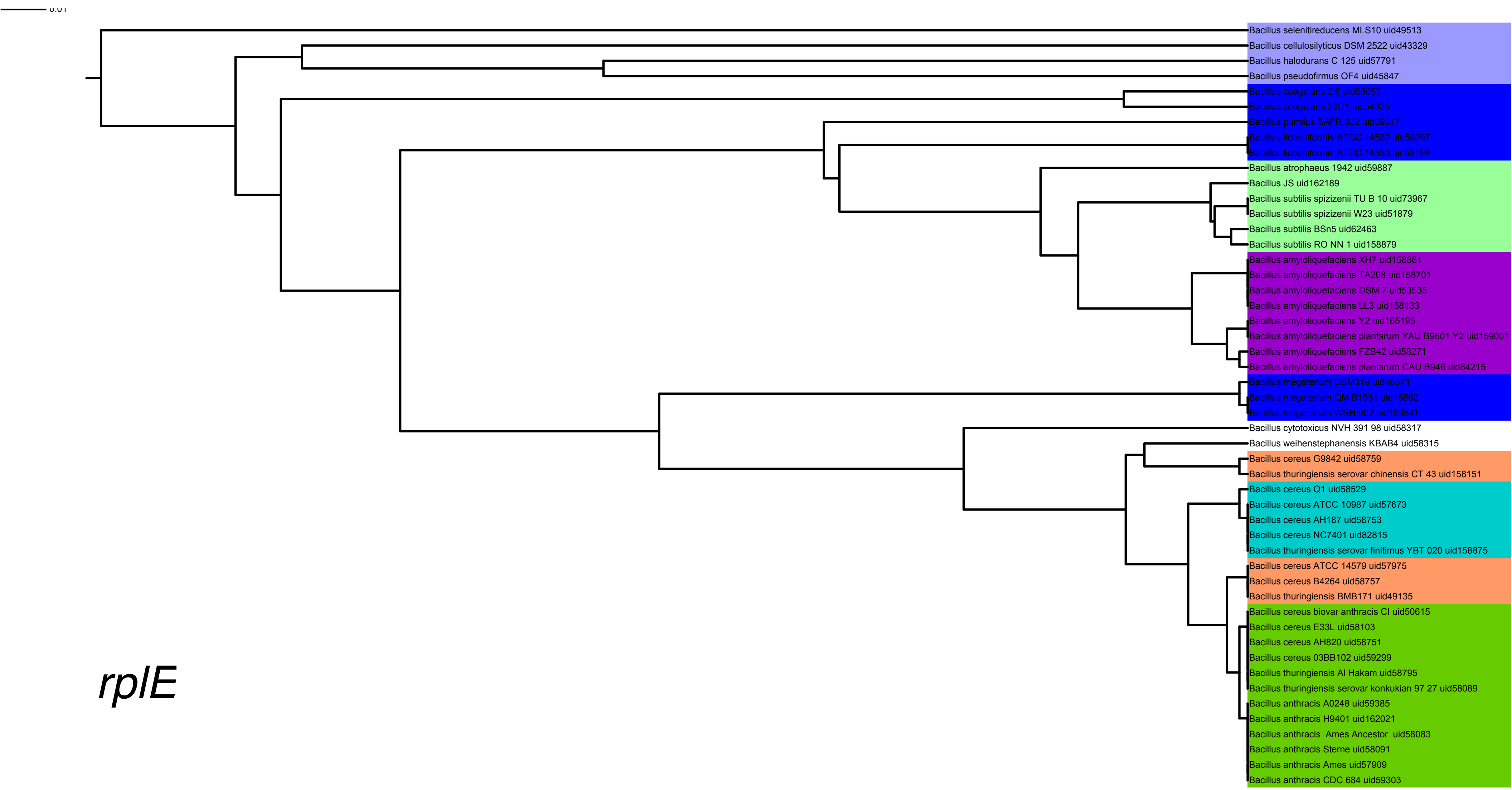
Bacillus selenitireducens MLS10 uid49513
Bacillus cellulosilyticus DSM 2522 uid43329
Bacillus halodurans C 125 uid57791
Bacillus pseudofirmus OF4 uid45847
Bacillus coagulans 2 6 uid68053
Bacillus coagulans 36D1 uid54335
Bacillus pumilus SAFR 032 uid59017
Bacillus licheniformis ATCC 14580 uid58097
Bacillus licheniformis ATCC 14580 uid58199
Bacillus atrophaeus 1942 uid59887
Bacillus JS uid162189
Bacillus subtilis spizizenii TU B 10 uid73967
Bacillus subtilis spizizenii W23 uid51879
Bacillus subtilis BSn5 uid62463
Bacillus subtilis RO NN 1 uid158879
Bacillus amyloliquefaciens XH7 uid158881
Bacillus amyloliquefaciens TA208 uid158701
Bacillus amyloliquefaciens DSM 7 uid53535
Bacillus amyloliquefaciens LL3 uid158133
Bacillus amyloliquefaciens Y2 uid165195
Bacillus amyloliquefaciens plantarum YAU B9601 Y2 uid159001
Bacillus amyloliquefaciens FZB42 uid58271
Bacillus amyloliquefaciens plantarum CAU B946 uid84215
Bacillus megaterium DSM319 uid48371
Bacillus megaterium QM B1551 uid15862
Bacillus megaterium WSH 002 uid159841
Bacillus cytotoxicus NVH 391 98 uid58317
Bacillus weihenstephanensis KBAB4 uid58315
Bacillus cereus G9842 uid58759
Bacillus thuringiensis serovar chinensis CT 43 uid158151
Bacillus cereus Q1 uid58529
Bacillus cereus ATCC 10987 uid57673
Bacillus cereus AH187 uid58753
Bacillus cereus NC7401 uid82815
Bacillus thuringiensis serovar finitimus YBT 020 uid158875
Bacillus cereus ATCC 14579 uid57975
Bacillus cereus B4264 uid58757
Bacillus thuringiensis BMB171 uid49135
Bacillus cereus biovar anthracis CI uid50615
Bacillus cereus E33L uid58103
Bacillus cereus AH820 uid58751
Bacillus cereus 03BB102 uid59299
Bacillus thuringiensis Al Hakam uid58795
Bacillus thuringiensis serovar konkukian 97 27 uid58089
Bacillus anthracis A0248 uid59385
Bacillus anthracis H9401 uid162021
Bacillus anthracis Ames Ancestor uid58083
Bacillus anthracis Sterne uid58091
Bacillus anthracis Ames uid57909
Bacillus anthracis CDC 684 uid59303
rplE

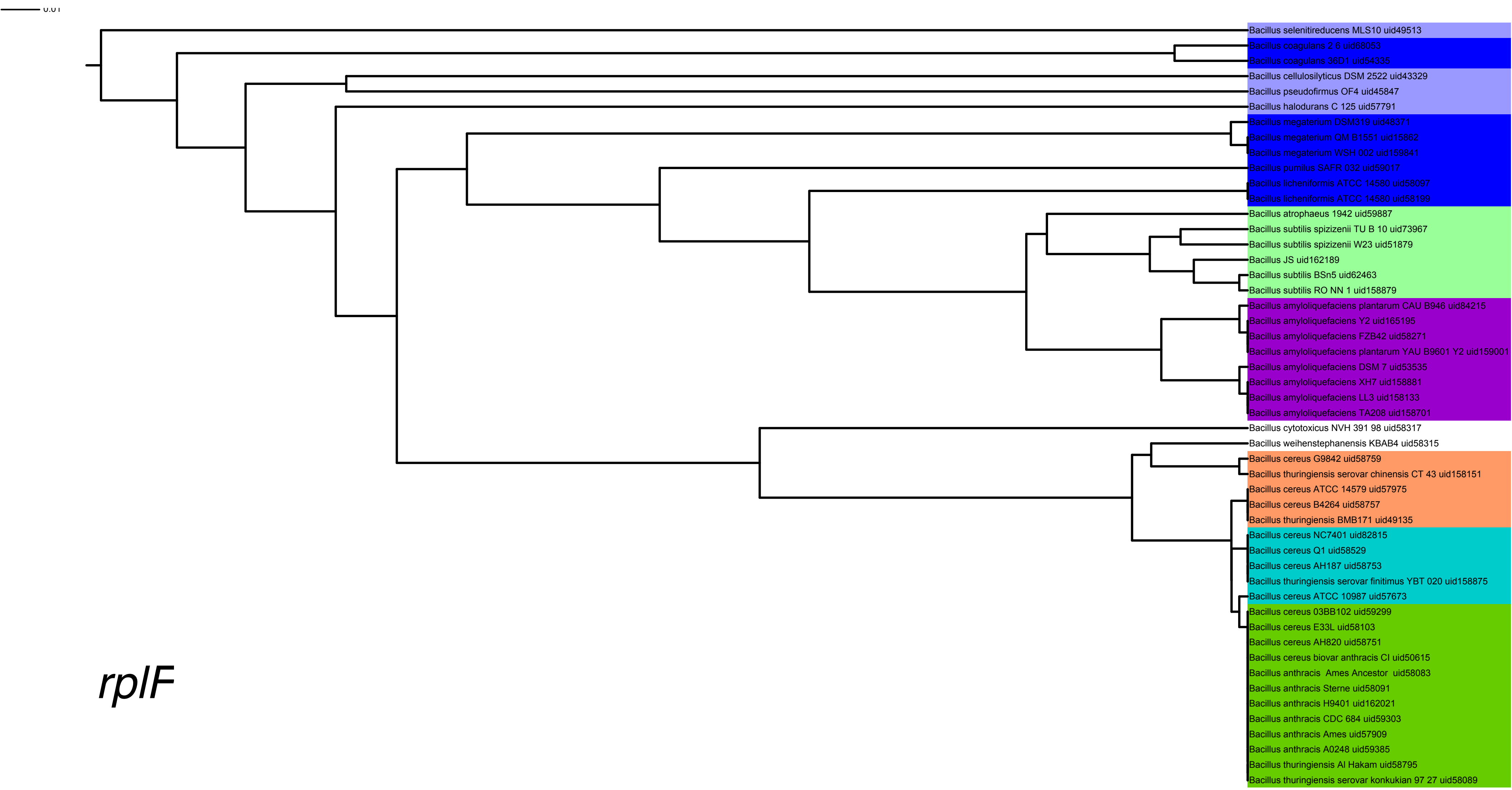
0.01
Bacillus selenitireducens MLS10 uid49513
Bacillus coagulans 2 6 uid68053
Bacillus coagulans 36D1 uid54335
Bacillus cellulosilyticus DSM 2522 uid43329
Bacillus pseudofirmus OF4 uid45847
Bacillus halodurans C 125 uid57791
Bacillus megaterium DSM319 uid48371
Bacillus megaterium QM B1551 uid15862
Bacillus megaterium WSH 002 uid159841
Bacillus pumilus SAFR 032 uid59017
Bacillus licheniformis ATCC 14580 uid58097
Bacillus licheniformis ATCC 14580 uid58199
Bacillus atrophaeus 1942 uid59887
Bacillus subtilis spizizenii TU B 10 uid73967
Bacillus subtilis spizizenii W23 uid51879
Bacillus JS uid162189
Bacillus subtilis BSn5 uid62463
Bacillus subtilis RO NN 1 uid158879
Bacillus amyloliquefaciens plantarum CAU B946 uid84215
Bacillus amyloliquefaciens Y2 uid165195
Bacillus amyloliquefaciens FZB42 uid58271
Bacillus amyloliquefaciens plantarum YAU B9601 Y2 uid159001
Bacillus amyloliquefaciens DSM 7 uid53535
Bacillus amyloliquefaciens XH7 uid158881
Bacillus amyloliquefaciens LL3 uid158133
Bacillus amyloliquefaciens TA208 uid158701
Bacillus cytotoxicus NVH 391 98 uid58317
Bacillus weihenstephanensis KBAB4 uid58315
Bacillus cereus G9842 uid58759
Bacillus thuringiensis serovar chinensis CT 43 uid158151
Bacillus cereus ATCC 14579 uid57975
Bacillus cereus B4264 uid58757
Bacillus thuringiensis BMB171 uid49135
Bacillus cereus NC7401 uid82815
Bacillus cereus Q1 uid58529
Bacillus cereus AH187 uid58753
Bacillus thuringiensis serovar finitimus YBT 020 uid158875
Bacillus cereus ATCC 10987 uid57673
Bacillus cereus 03BB102 uid59299
Bacillus cereus E33L uid58103
Bacillus cereus AH820 uid58751
Bacillus cereus biovar anthracis CI uid50615
Bacillus anthracis Ames Ancestor uid58083
Bacillus anthracis Sterne uid58091
Bacillus anthracis H9401 uid162021
Bacillus anthracis CDC 684 uid59303
Bacillus anthracis Ames uid57909
Bacillus anthracis A0248 uid59385
Bacillus thuringiensis Al Hakam uid58795
Bacillus thuringiensis serovar konkukian 97 27 uid58089
rplF

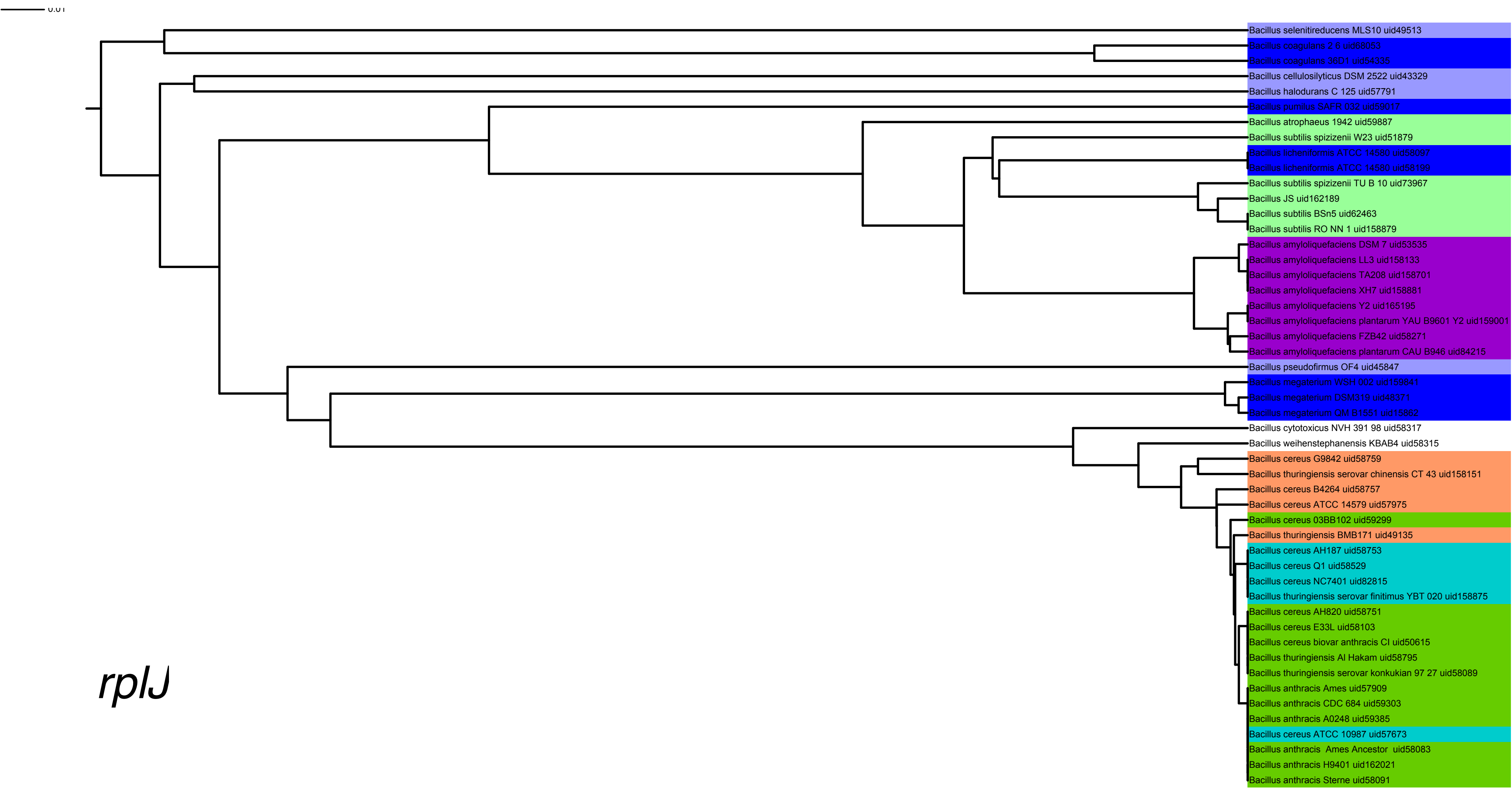

0.01
Bacillus selenitireducens MLS10 uid49513
Bacillus coagulans 2 6 uid68053
Bacillus coagulans 36D1 uid54335
Bacillus cellulosilyticus DSM 2522 uid43329
Bacillus halodurans C 125 uid57791
Bacillus pumilus SAFR 032 uid59017
Bacillus atrophaeus 1942 uid59887
Bacillus subtilis spizizenii W23 uid51879
Bacillus licheniformis ATCC 14580 uid58097
Bacillus licheniformis ATCC 14580 uid58199
Bacillus subtilis spizizenii TU B 10 uid73967
Bacillus JS uid162189
Bacillus subtilis BSn5 uid62463
Bacillus subtilis RO NN 1 uid158879
Bacillus amyloliquefaciens DSM 7 uid53535
Bacillus amyloliquefaciens LL3 uid158133
Bacillus amyloliquefaciens TA208 uid158701
Bacillus amyloliquefaciens XH7 uid158881
Bacillus amyloliquefaciens Y2 uid165195
Bacillus amyloliquefaciens plantarum YAU B9601 Y2 uid159001
Bacillus amyloliquefaciens FZB42 uid58271
Bacillus amyloliquefaciens plantarum CAU B946 uid84215
Bacillus pseudofirmus OF4 uid45847
Bacillus megaterium WSH 002 uid159841
Bacillus megaterium DSM319 uid48371
Bacillus megaterium QM B1551 uid15862
Bacillus cytotoxicus NVH 391 98 uid58317
Bacillus weihenstephanensis KBAB4 uid58315
Bacillus cereus G9842 uid58759
Bacillus thuringiensis serovar chinensis CT 43 uid158151
Bacillus cereus B4264 uid58757
Bacillus cereus ATCC 14579 uid57975
Bacillus cereus 03BB102 uid59299
Bacillus thuringiensis BMB171 uid49135
Bacillus cereus AH187 uid58753
Bacillus cereus Q1 uid58529
Bacillus cereus NC7401 uid82815
Bacillus thuringiensis serovar finitimus YBT 020 uid158875
Bacillus cereus AH820 uid58751
Bacillus cereus E33L uid58103
Bacillus cereus biovar anthracis CI uid50615
Bacillus thuringiensis Al Hakam uid58795
Bacillus thuringiensis serovar konkukian 97 27 uid58089
Bacillus anthracis Ames uid57909
Bacillus anthracis CDC 684 uid59303
Bacillus anthracis A0248 uid59385
Bacillus cereus ATCC 10987 uid57673
Bacillus anthracis Ames Ancestor uid58083
Bacillus anthracis H9401 uid162021
Bacillus anthracis Sterne uid58091
rplJ

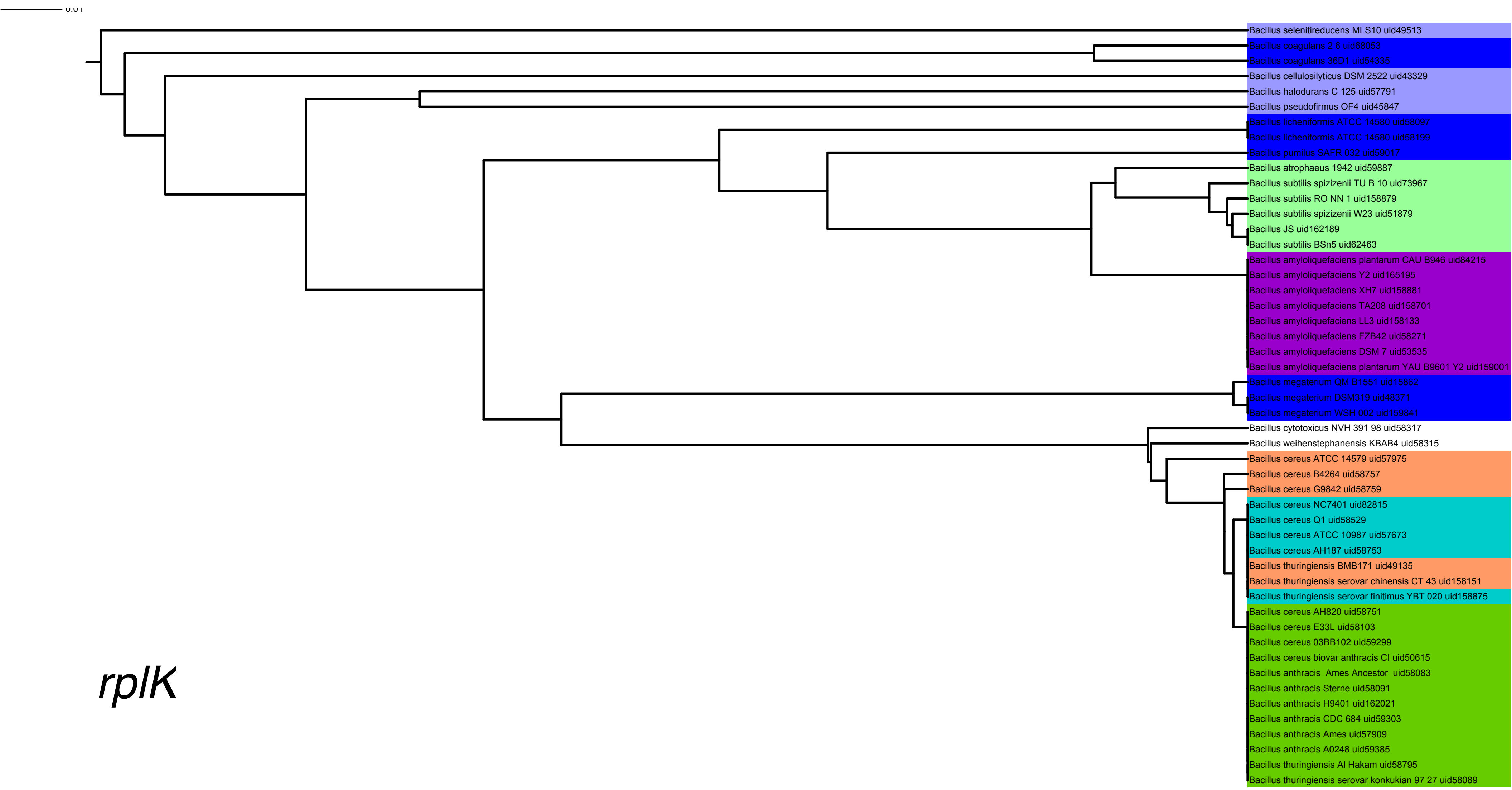

0.01
Bacillus selenitireducens MLS10 uid49513
Bacillus coagulans 2 6 uid68053
Bacillus coagulans 36D1 uid54335
Bacillus cellulosilyticus DSM 2522 uid43329
Bacillus halodurans C 125 uid57791
Bacillus pseudofirmus OF4 uid45847
Bacillus licheniformis ATCC 14580 uid58097
Bacillus licheniformis ATCC 14580 uid58199
Bacillus pumilus SAFR 032 uid59017
Bacillus atrophaeus 1942 uid59887
Bacillus subtilis spizizenii TU B 10 uid73967
Bacillus subtilis RO NN 1 uid158879
Bacillus subtilis spizizenii W23 uid51879
Bacillus JS uid162189
Bacillus subtilis BSn5 uid62463
Bacillus amyloliquefaciens plantarum CAU B946 uid84215
Bacillus amyloliquefaciens Y2 uid165195
Bacillus amyloliquefaciens XH7 uid158881
Bacillus amyloliquefaciens TA208 uid158701
Bacillus amyloliquefaciens LL3 uid158133
Bacillus amyloliquefaciens FZB42 uid58271
Bacillus amyloliquefaciens DSM 7 uid53535
Bacillus amyloliquefaciens plantarum YAU B9601 Y2 uid159001
Bacillus megaterium QM B1551 uid15862
Bacillus megaterium DSM319 uid48371
Bacillus megaterium WSH 002 uid159841
Bacillus cytotoxicus NVH 391 98 uid58317
Bacillus weihenstephanensis KBAB4 uid58315
Bacillus cereus ATCC 14579 uid57975
Bacillus cereus B4264 uid58757
Bacillus cereus G9842 uid58759
Bacillus cereus NC7401 uid82815
Bacillus cereus Q1 uid58529
Bacillus cereus ATCC 10987 uid57673
Bacillus cereus AH187 uid58753
Bacillus thuringiensis BMB171 uid49135
Bacillus thuringiensis serovar chinensis CT 43 uid158151
Bacillus thuringiensis serovar finitimus YBT 020 uid158875
Bacillus cereus AH820 uid58751
Bacillus cereus E33L uid58103
Bacillus cereus 03BB102 uid59299
Bacillus cereus biovar anthracis CI uid50615
Bacillus anthracis Ames Ancestor uid58083
Bacillus anthracis Sterne uid58091
Bacillus anthracis H9401 uid162021
Bacillus anthracis CDC 684 uid59303
Bacillus anthracis Ames uid57909
Bacillus anthracis A0248 uid59385
Bacillus thuringiensis Al Hakam uid58795
Bacillus thuringiensis serovar konkukian 97 27 uid58089
rplK

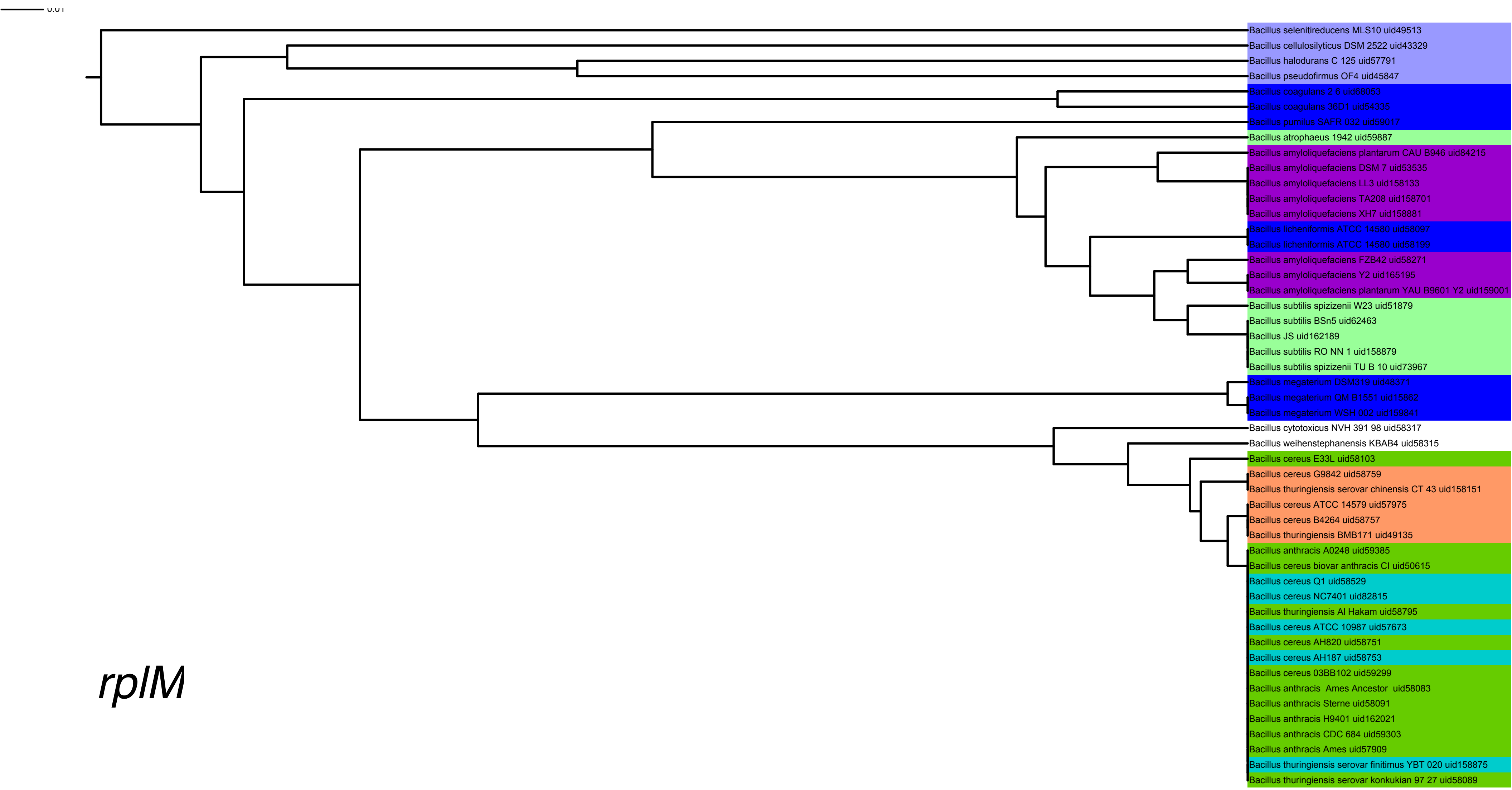
0.01
Bacillus selenitireducens MLS10 uid49513
Bacillus cellulosilyticus DSM 2522 uid43329
Bacillus halodurans C 125 uid57791
Bacillus pseudofirmus OF4 uid45847
Bacillus coagulans 2 6 uid68053
Bacillus coagulans 36D1 uid54335
Bacillus pumilus SAFR 032 uid59017
Bacillus atrophaeus 1942 uid59887
Bacillus amyloliquefaciens plantarum CAU B946 uid84215
Bacillus amyloliquefaciens DSM 7 uid53535
Bacillus amyloliquefaciens LL3 uid158133
Bacillus amyloliquefaciens TA208 uid158701
Bacillus amyloliquefaciens XH7 uid158881
Bacillus licheniformis ATCC 14580 uid58097
Bacillus licheniformis ATCC 14580 uid58199
Bacillus amyloliquefaciens FZB42 uid58271
Bacillus amyloliquefaciens Y2 uid165195
Bacillus amyloliquefaciens plantarum YAU B9601 Y2 uid159001
Bacillus subtilis spizizenii W23 uid51879
Bacillus subtilis BSn5 uid62463
Bacillus JS uid162189
Bacillus subtilis RO NN 1 uid158879
Bacillus subtilis spizizenii TU B 10 uid73967
Bacillus megaterium DSM319 uid48371
Bacillus megaterium QM B1551 uid15862
Bacillus megaterium WSH 002 uid159841
Bacillus cytotoxicus NVH 391 98 uid58317
Bacillus weihenstephanensis KBAB4 uid58315
Bacillus cereus E33L uid58103
Bacillus cereus G9842 uid58759
Bacillus thuringiensis serovar chinensis CT 43 uid158151
Bacillus cereus ATCC 14579 uid57975
Bacillus cereus B4264 uid58757
Bacillus thuringiensis BMB171 uid49135
Bacillus anthracis A0248 uid59385
Bacillus cereus biovar anthracis CI uid50615
Bacillus cereus Q1 uid58529
Bacillus cereus NC7401 uid82815
Bacillus thuringiensis Al Hakam uid58795
Bacillus cereus ATCC 10987 uid57673
Bacillus cereus AH820 uid58751
Bacillus cereus AH187 uid58753
Bacillus cereus 03BB102 uid59299
Bacillus anthracis Ames Ancestor uid58083
Bacillus anthracis Sterne uid58091
Bacillus anthracis H9401 uid162021
Bacillus anthracis CDC 684 uid59303
Bacillus anthracis Ames uid57909
Bacillus thuringiensis serovar finitimus YBT 020 uid158875
Bacillus thuringiensis serovar konkukian 97 27 uid58089
rplM

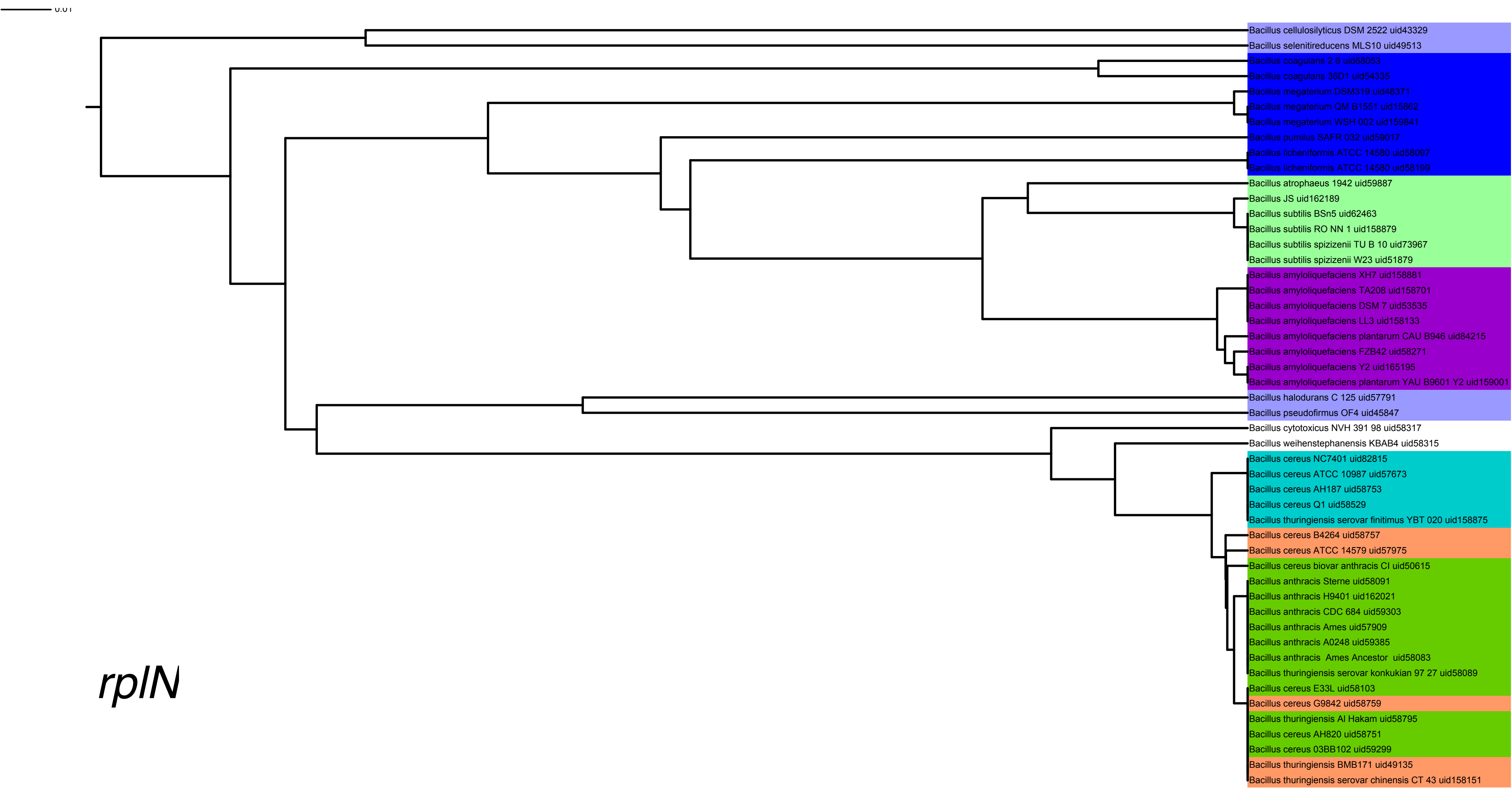
0.01
Bacillus cellulosilyticus DSM 2522 uid43329
Bacillus selenitireducens MLS10 uid49513
Bacillus coagulans 2 6 uid68053
Bacillus coagulans 36D1 uid54335
Bacillus megaterium DSM319 uid48371
Bacillus megaterium QM B1551 uid15862
Bacillus megaterium WSH 002 uid159841
Bacillus pumilus SAFR 032 uid59017
Bacillus licheniformis ATCC 14580 uid58097
Bacillus licheniformis ATCC 14580 uid58199
Bacillus atrophaeus 1942 uid59887
Bacillus JS uid162189
Bacillus subtilis BSn5 uid62463
Bacillus subtilis RO NN 1 uid158879
Bacillus subtilis spizizenii TU B 10 uid73967
Bacillus subtilis spizizenii W23 uid51879
Bacillus amyloliquefaciens XH7 uid158881
Bacillus amyloliquefaciens TA208 uid158701
Bacillus amyloliquefaciens DSM 7 uid53535
Bacillus amyloliquefaciens LL3 uid158133
Bacillus amyloliquefaciens plantarum CAU B946 uid84215
Bacillus amyloliquefaciens FZB42 uid58271
Bacillus amyloliquefaciens Y2 uid165195
Bacillus amyloliquefaciens plantarum YAU B9601 Y2 uid159001
Bacillus halodurans C 125 uid57791
Bacillus pseudofirmus OF4 uid45847
Bacillus cytotoxicus NVH 391 98 uid58317
Bacillus weihenstephanensis KBAB4 uid58315
Bacillus cereus NC7401 uid82815
Bacillus cereus ATCC 10987 uid57673
Bacillus cereus AH187 uid58753
Bacillus cereus Q1 uid58529
Bacillus thuringiensis serovar finitimus YBT 020 uid158875
Bacillus cereus B4264 uid58757
Bacillus cereus ATCC 14579 uid57975
Bacillus cereus biovar anthracis CI uid50615
Bacillus anthracis Sterne uid58091
Bacillus anthracis H9401 uid162021
Bacillus anthracis CDC 684 uid59303
Bacillus anthracis Ames uid57909
Bacillus anthracis A0248 uid59385
Bacillus anthracis Ames Ancestor uid58083
Bacillus thuringiensis serovar konkukian 97 27 uid58089
Bacillus cereus E33L uid58103
Bacillus cereus G9842 uid58759
Bacillus thuringiensis Al Hakam uid58795
Bacillus cereus AH820 uid58751
Bacillus cereus 03BB102 uid59299
Bacillus thuringiensis BMB171 uid49135
Bacillus thuringiensis serovar chinensis CT 43 uid158151

*rplN*

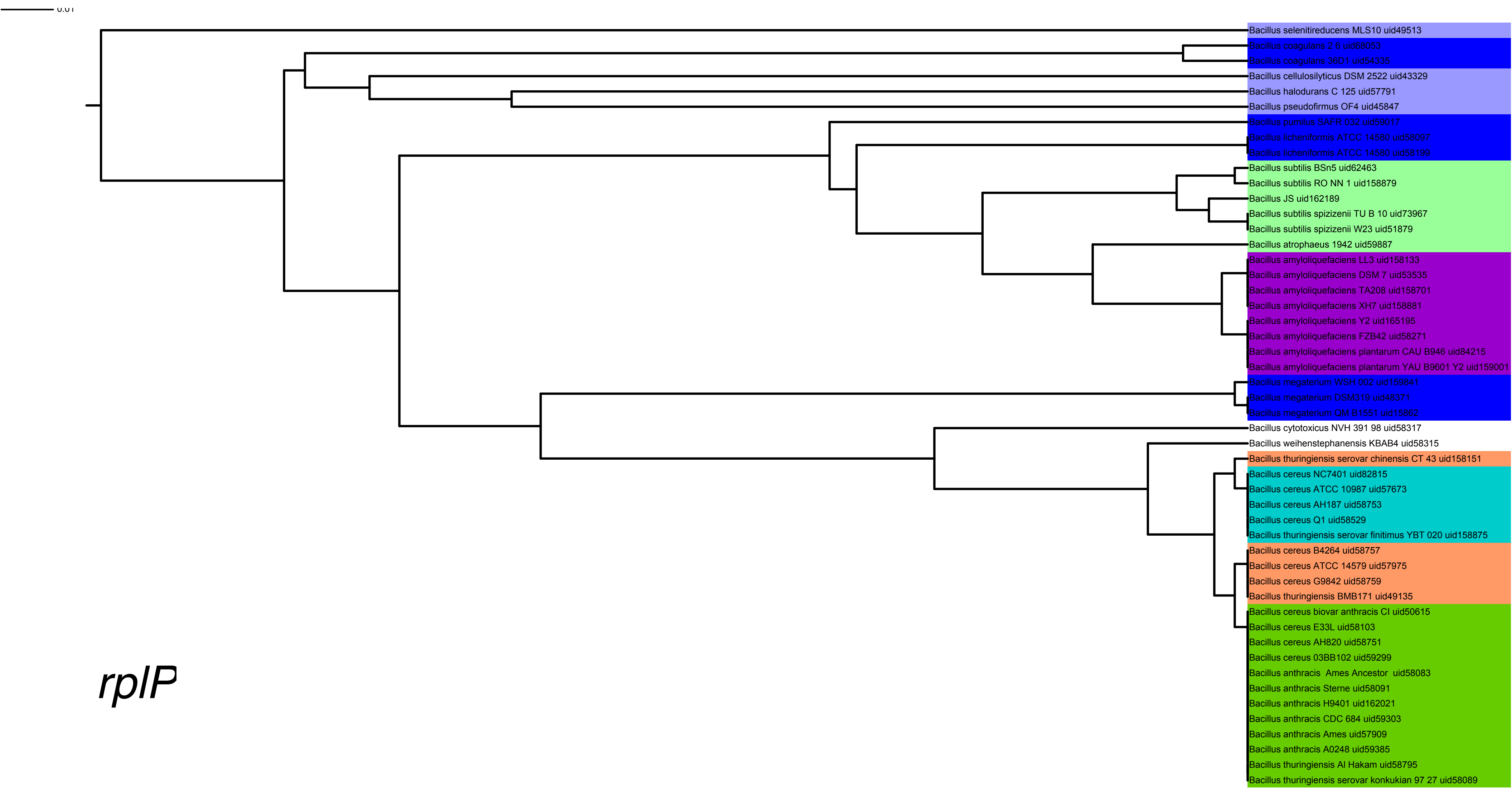
0.01
Bacillus selenitireducens MLS10 uid49513
Bacillus coagulans 2 6 uid68053
Bacillus coagulans 36D1 uid54335
Bacillus cellulosilyticus DSM 2522 uid43329
Bacillus halodurans C 125 uid57791
Bacillus pseudofirmus OF4 uid45847
Bacillus pumilus SAFR 032 uid59017
Bacillus licheniformis ATCC 14580 uid58097
Bacillus licheniformis ATCC 14580 uid58199
Bacillus subtilis BSn5 uid62463
Bacillus subtilis RO NN 1 uid158879
Bacillus JS uid162189
Bacillus subtilis spizizenii TU B 10 uid73967
Bacillus subtilis spizizenii W23 uid51879
Bacillus atrophaeus 1942 uid59887
Bacillus amyloliquefaciens LL3 uid158133
Bacillus amyloliquefaciens DSM 7 uid53535
Bacillus amyloliquefaciens TA208 uid158701
Bacillus amyloliquefaciens XH7 uid158881
Bacillus amyloliquefaciens Y2 uid165195
Bacillus amyloliquefaciens FZB42 uid58271
Bacillus amyloliquefaciens plantarum CAU B946 uid84215
Bacillus amyloliquefaciens plantarum YAU B9601 Y2 uid159001
Bacillus megaterium WSH 002 uid159841
Bacillus megaterium DSM319 uid48371
Bacillus megaterium QM B1551 uid15862
Bacillus cytotoxicus NVH 391 98 uid58317
Bacillus weihenstephanensis KBAB4 uid58315
Bacillus thuringiensis serovar chinensis CT 43 uid158151
Bacillus cereus NC7401 uid82815
Bacillus cereus ATCC 10987 uid57673
Bacillus cereus AH187 uid58753
Bacillus cereus Q1 uid58529
Bacillus thuringiensis serovar finitimus YBT 020 uid158875
Bacillus cereus B4264 uid58757
Bacillus cereus ATCC 14579 uid57975
Bacillus cereus G9842 uid58759
Bacillus thuringiensis BMB171 uid49135
Bacillus cereus biovar anthracis CI uid50615
Bacillus cereus E33L uid58103
Bacillus cereus AH820 uid58751
Bacillus cereus 03BB102 uid59299
Bacillus anthracis Ames Ancestor uid58083
Bacillus anthracis Sterne uid58091
Bacillus anthracis H9401 uid162021
Bacillus anthracis CDC 684 uid59303
Bacillus anthracis Ames uid57909
Bacillus anthracis A0248 uid59385
Bacillus thuringiensis Al Hakam uid58795
Bacillus thuringiensis serovar konkukian 97 27 uid58089
rplP

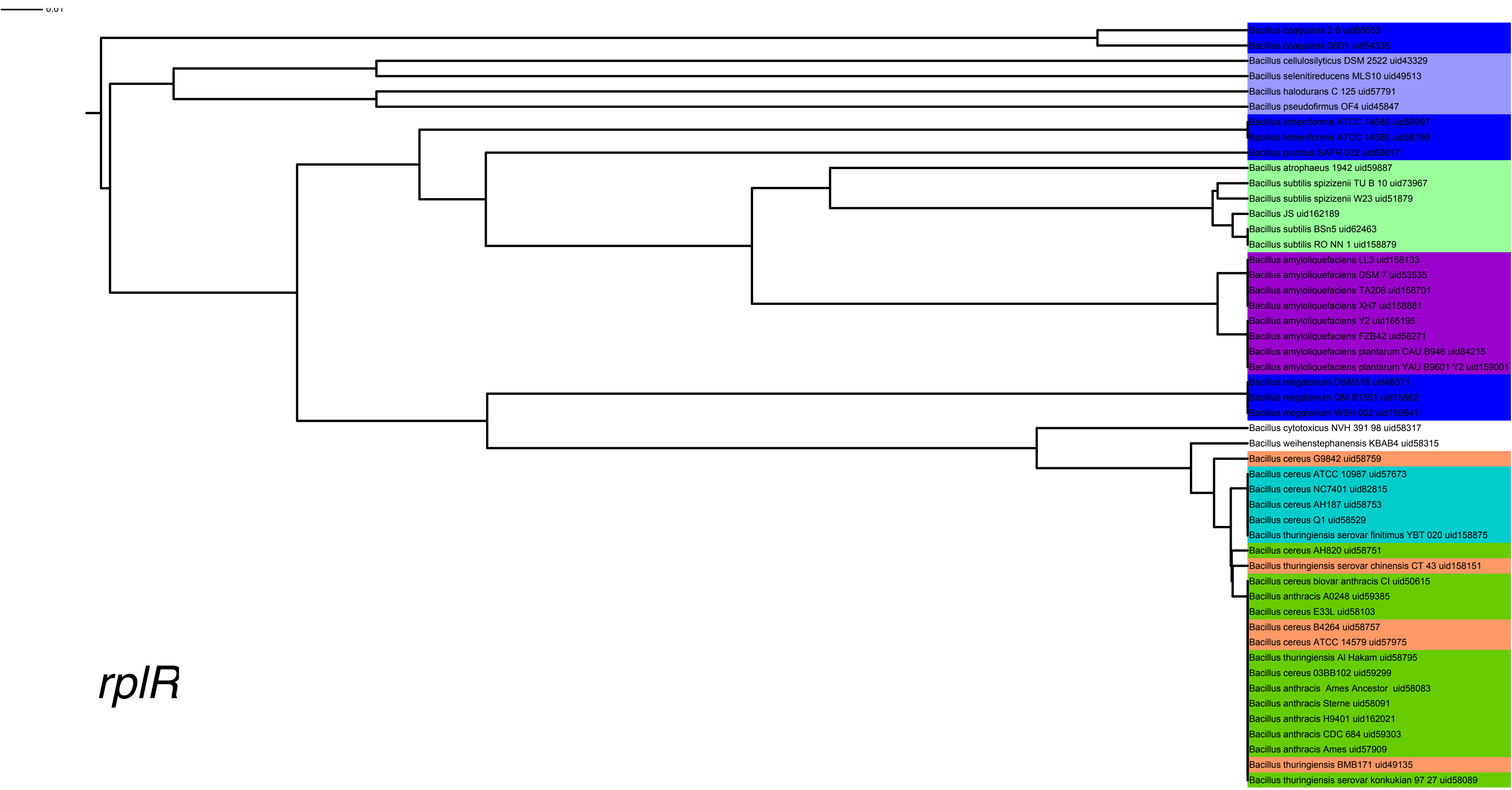
0.01
Bacillus coagulans 2 6 uid68053
Bacillus coagulans 36D1 uid54335
Bacillus cellulosilyticus DSM 2522 uid43329
Bacillus selenitireducens MLS10 uid49513
Bacillus halodurans C 125 uid57791
Bacillus pseudofirmus OF4 uid45847
Bacillus licheniformis ATCC 14580 uid58097
Bacillus licheniformis ATCC 14580 uid58199
Bacillus pumilus SAFR 032 uid59017
Bacillus atrophaeus 1942 uid59887
Bacillus subtilis spizizenii TU B 10 uid73967
Bacillus subtilis spizizenii W23 uid51879
Bacillus JS uid162189
Bacillus subtilis BSn5 uid62463
Bacillus subtilis RO NN 1 uid158879
Bacillus amyloliquefaciens LL3 uid158133
Bacillus amyloliquefaciens DSM 7 uid53535
Bacillus amyloliquefaciens TA208 uid158701
Bacillus amyloliquefaciens XH7 uid158881
Bacillus amyloliquefaciens Y2 uid165195
Bacillus amyloliquefaciens FZB42 uid58271
Bacillus amyloliquefaciens plantarum CAU B946 uid84215
Bacillus amyloliquefaciens plantarum YAU B9601 Y2 uid159001
Bacillus megaterium DSM319 uid48371
Bacillus megaterium QM B1551 uid15862
Bacillus megaterium WSH 002 uid159841
Bacillus cytotoxicus NVH 391 98 uid58317
Bacillus weihenstephanensis KBAB4 uid58315
Bacillus cereus G9842 uid58759
Bacillus cereus ATCC 10987 uid57673
Bacillus cereus NC7401 uid82815
Bacillus cereus AH187 uid58753
Bacillus cereus Q1 uid58529
Bacillus thuringiensis serovar finitimus YBT 020 uid158875
Bacillus cereus AH820 uid58751
Bacillus thuringiensis serovar chinensis CT 43 uid158151
Bacillus cereus biovar anthracis CI uid50615
Bacillus anthracis A0248 uid59385
Bacillus cereus E33L uid58103
Bacillus cereus B4264 uid58757
Bacillus cereus ATCC 14579 uid57975
Bacillus thuringiensis Al Hakam uid58795
Bacillus cereus 03BB102 uid59299
Bacillus anthracis  Ames Ancestor  uid58083
Bacillus anthracis Sterne uid58091
Bacillus anthracis H9401 uid162021
Bacillus anthracis CDC 684 uid59303
Bacillus anthracis Ames uid57909
Bacillus thuringiensis BMB171 uid49135
Bacillus thuringiensis serovar konkukian 97 27 uid58089

*rplR*

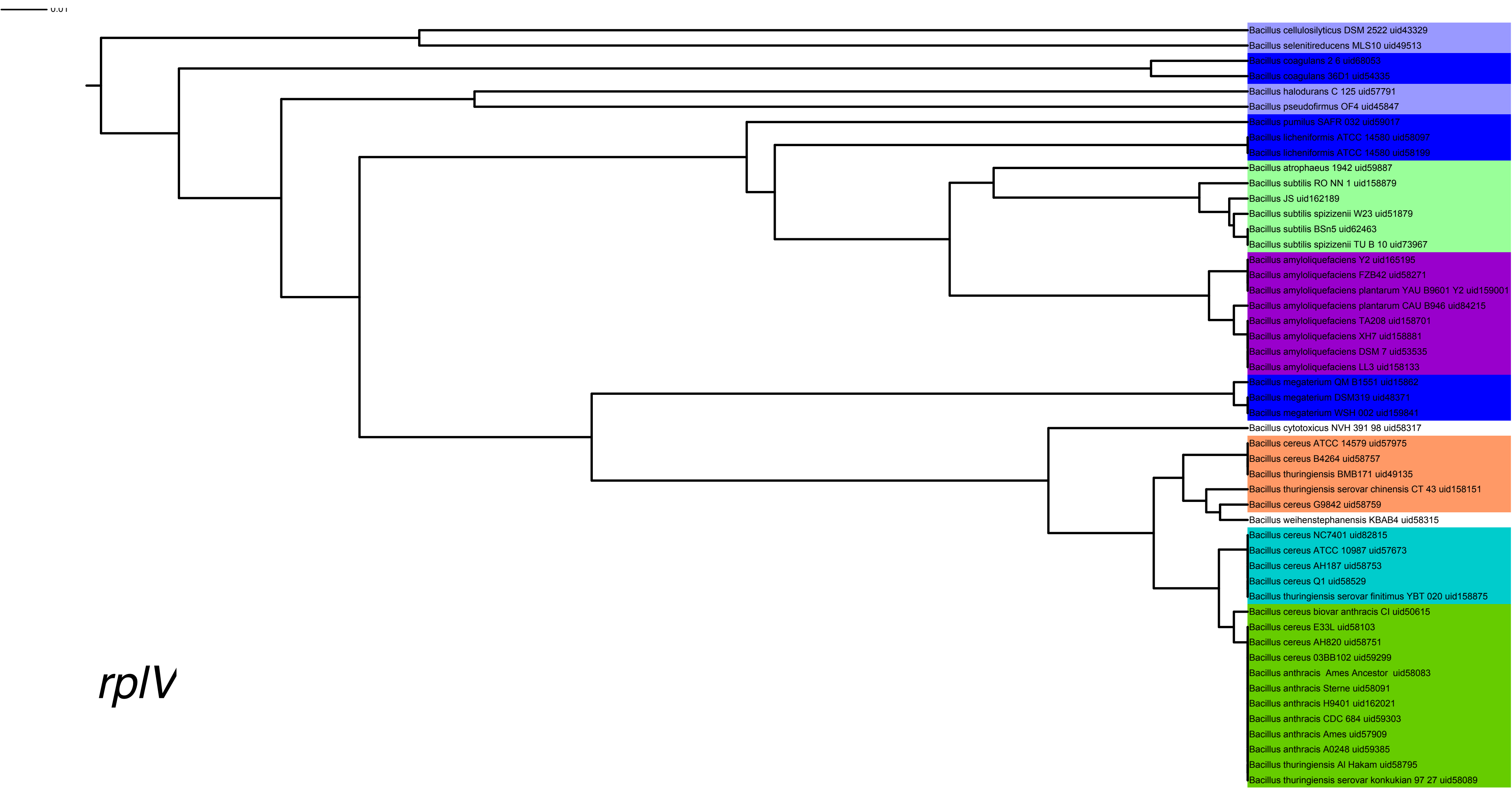
0.01
Bacillus cellulosilyticus DSM 2522 uid43329
Bacillus selenitireducens MLS10 uid49513
Bacillus coagulans 2 6 uid68053
Bacillus coagulans 36D1 uid54335
Bacillus halodurans C 125 uid57791
Bacillus pseudofirmus OF4 uid45847
Bacillus pumilus SAFR 032 uid59017
Bacillus licheniformis ATCC 14580 uid58097
Bacillus licheniformis ATCC 14580 uid58199
Bacillus atrophaeus 1942 uid59887
Bacillus subtilis RO NN 1 uid158879
Bacillus JS uid162189
Bacillus subtilis spizizenii W23 uid51879
Bacillus subtilis BSn5 uid62463
Bacillus subtilis spizizenii TU B 10 uid73967
Bacillus amyloliquefaciens Y2 uid165195
Bacillus amyloliquefaciens FZB42 uid58271
Bacillus amyloliquefaciens plantarum YAU B9601 Y2 uid159001
Bacillus amyloliquefaciens plantarum CAU B946 uid84215
Bacillus amyloliquefaciens TA208 uid158701
Bacillus amyloliquefaciens XH7 uid158881
Bacillus amyloliquefaciens DSM 7 uid53535
Bacillus amyloliquefaciens LL3 uid158133
Bacillus megaterium QM B1551 uid15862
Bacillus megaterium DSM319 uid48371
Bacillus megaterium WSH 002 uid159841
Bacillus cytotoxicus NVH 391 98 uid58317
Bacillus cereus ATCC 14579 uid57975
Bacillus cereus B4264 uid58757
Bacillus thuringiensis BMB171 uid49135
Bacillus thuringiensis serovar chinensis CT 43 uid158151
Bacillus cereus G9842 uid58759
Bacillus weihenstephanensis KBAB4 uid58315
Bacillus cereus NC7401 uid82815
Bacillus cereus ATCC 10987 uid57673
Bacillus cereus AH187 uid58753
Bacillus cereus Q1 uid58529
Bacillus thuringiensis serovar finitimus YBT 020 uid158875
Bacillus cereus biovar anthracis CI uid50615
Bacillus cereus E33L uid58103
Bacillus cereus AH820 uid58751
Bacillus cereus 03BB102 uid59299
Bacillus anthracis Ames Ancestor uid58083
Bacillus anthracis Sterne uid58091
Bacillus anthracis H9401 uid162021
Bacillus anthracis CDC 684 uid59303
Bacillus anthracis Ames uid57909
Bacillus anthracis A0248 uid59385
Bacillus thuringiensis Al Hakam uid58795
Bacillus thuringiensis serovar konkukian 97 27 uid58089
rplV

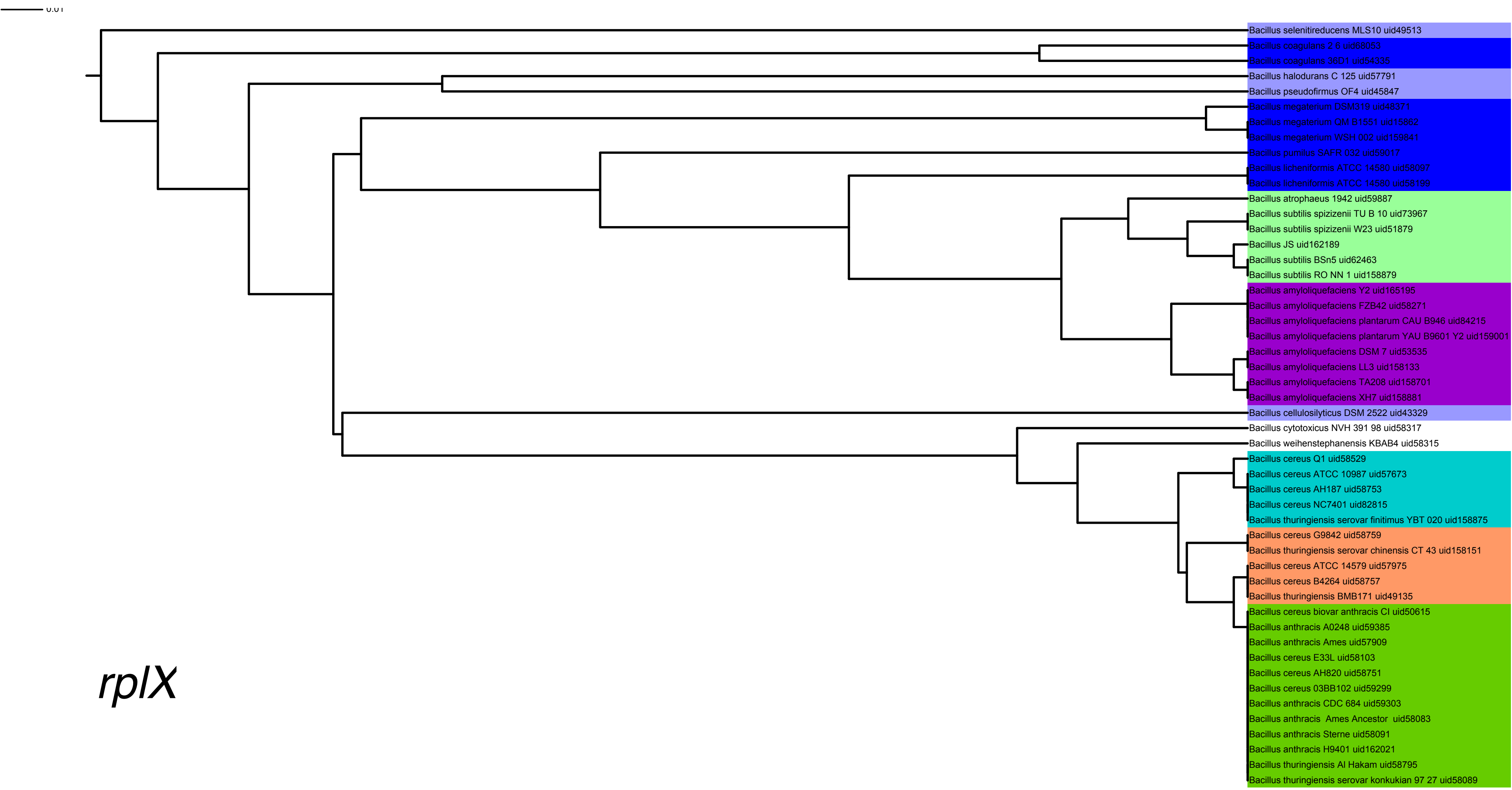
0.01
Bacillus selenitireducens MLS10 uid49513
Bacillus coagulans 2 6 uid68053
Bacillus coagulans 36D1 uid54335
Bacillus halodurans C 125 uid57791
Bacillus pseudofirmus OF4 uid45847
Bacillus megaterium DSM319 uid48371
Bacillus megaterium QM B1551 uid15862
Bacillus megaterium WSH 002 uid159841
Bacillus pumilus SAFR 032 uid59017
Bacillus licheniformis ATCC 14580 uid58097
Bacillus licheniformis ATCC 14580 uid58199
Bacillus atrophaeus 1942 uid59887
Bacillus subtilis spizizenii TU B 10 uid73967
Bacillus subtilis spizizenii W23 uid51879
Bacillus JS uid162189
Bacillus subtilis BSn5 uid62463
Bacillus subtilis RO NN 1 uid158879
Bacillus amyloliquefaciens Y2 uid165195
Bacillus amyloliquefaciens FZB42 uid58271
Bacillus amyloliquefaciens plantarum CAU B946 uid84215
Bacillus amyloliquefaciens plantarum YAU B9601 Y2 uid159001
Bacillus amyloliquefaciens DSM 7 uid53535
Bacillus amyloliquefaciens LL3 uid158133
Bacillus amyloliquefaciens TA208 uid158701
Bacillus amyloliquefaciens XH7 uid158881
Bacillus cellulosilyticus DSM 2522 uid43329
Bacillus cytotoxicus NVH 391 98 uid58317
Bacillus weihenstephanensis KBAB4 uid58315
Bacillus cereus Q1 uid58529
Bacillus cereus ATCC 10987 uid57673
Bacillus cereus AH187 uid58753
Bacillus cereus NC7401 uid82815
Bacillus thuringiensis serovar finitimus YBT 020 uid158875
Bacillus cereus G9842 uid58759
Bacillus thuringiensis serovar chinensis CT 43 uid158151
Bacillus cereus ATCC 14579 uid57975
Bacillus cereus B4264 uid58757
Bacillus thuringiensis BMB171 uid49135
Bacillus cereus biovar anthracis CI uid50615
Bacillus anthracis A0248 uid59385
Bacillus anthracis Ames uid57909
Bacillus cereus E33L uid58103
Bacillus cereus AH820 uid58751
Bacillus cereus 03BB102 uid59299
Bacillus anthracis CDC 684 uid59303
Bacillus anthracis Ames Ancestor uid58083
Bacillus anthracis Sterne uid58091
Bacillus anthracis H9401 uid162021
Bacillus thuringiensis Al Hakam uid58795
Bacillus thuringiensis serovar konkukian 97 27 uid58089
rplX

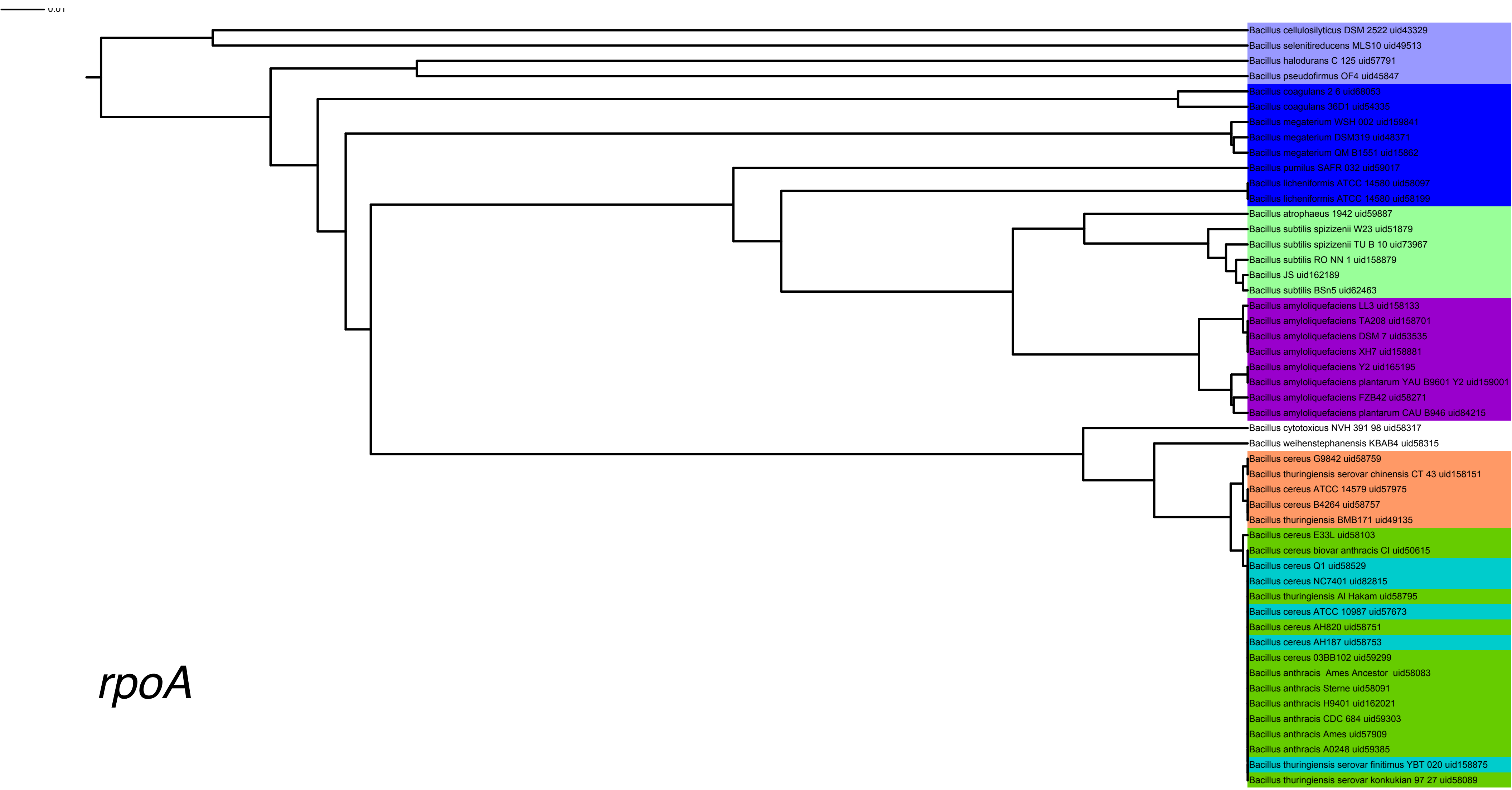
0.01
rpoA
Bacillus cellulosilyticus DSM 2522 uid43329
Bacillus selenitireducens MLS10 uid49513
Bacillus halodurans C 125 uid57791
Bacillus pseudofirmus OF4 uid45847
Bacillus coagulans 2 6 uid68053
Bacillus coagulans 36D1 uid54335
Bacillus megaterium WSH 002 uid159841
Bacillus megaterium DSM319 uid48371
Bacillus megaterium QM B1551 uid15862
Bacillus pumilus SAFR 032 uid59017
Bacillus licheniformis ATCC 14580 uid58097
Bacillus licheniformis ATCC 14580 uid58199
Bacillus atrophaeus 1942 uid59887
Bacillus subtilis spizizenii W23 uid51879
Bacillus subtilis spizizenii TU B 10 uid73967
Bacillus subtilis RO NN 1 uid158879
Bacillus JS uid162189
Bacillus subtilis BSn5 uid62463
Bacillus amyloliquefaciens LL3 uid158133
Bacillus amyloliquefaciens TA208 uid158701
Bacillus amyloliquefaciens DSM 7 uid53535
Bacillus amyloliquefaciens XH7 uid158881
Bacillus amyloliquefaciens Y2 uid165195
Bacillus amyloliquefaciens plantarum YAU B9601 Y2 uid159001
Bacillus amyloliquefaciens FZB42 uid58271
Bacillus amyloliquefaciens plantarum CAU B946 uid84215
Bacillus cytotoxicus NVH 391 98 uid58317
Bacillus weihenstephanensis KBAB4 uid58315
Bacillus cereus G9842 uid58759
Bacillus thuringiensis serovar chinensis CT 43 uid158151
Bacillus cereus ATCC 14579 uid57975
Bacillus cereus B4264 uid58757
Bacillus thuringiensis BMB171 uid49135
Bacillus cereus E33L uid58103
Bacillus cereus biovar anthracis CI uid50615
Bacillus cereus Q1 uid58529
Bacillus cereus NC7401 uid82815
Bacillus thuringiensis Al Hakam uid58795
Bacillus cereus ATCC 10987 uid57673
Bacillus cereus AH820 uid58751
Bacillus cereus AH187 uid58753
Bacillus cereus 03BB102 uid59299
Bacillus anthracis Ames Ancestor uid58083
Bacillus anthracis Sterne uid58091
Bacillus anthracis H9401 uid162021
Bacillus anthracis CDC 684 uid59303
Bacillus anthracis Ames uid57909
Bacillus anthracis A0248 uid59385
Bacillus thuringiensis serovar finitimus YBT 020 uid158875
Bacillus thuringiensis serovar konkukian 97 27 uid58089

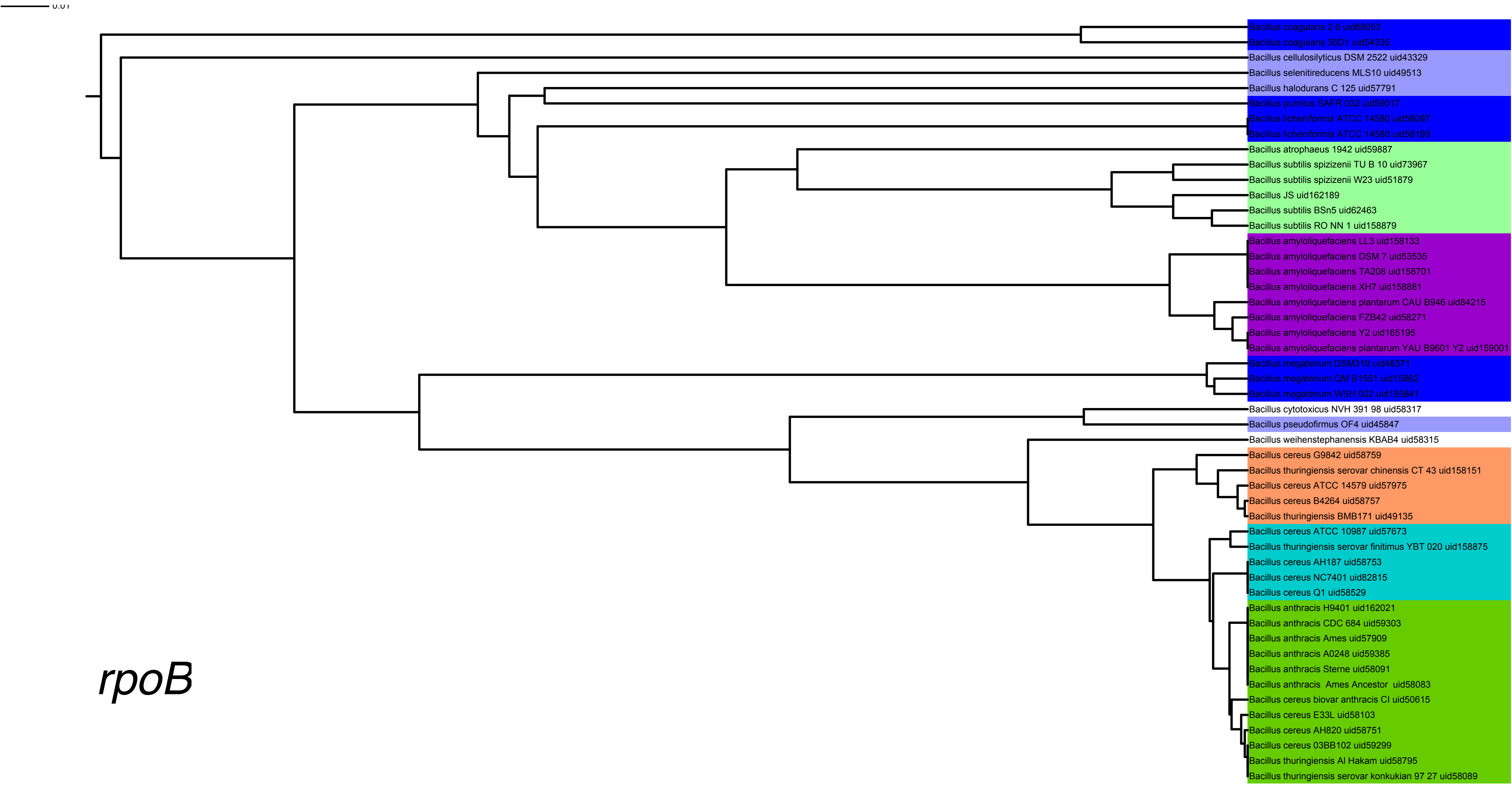
0.01
Bacillus coagulans 2 6 uid68053
Bacillus coagulans 36D1 uid54335
Bacillus cellulosilyticus DSM 2522 uid43329
Bacillus selenitireducens MLS10 uid49513
Bacillus halodurans C 125 uid57791
Bacillus pumilus SAFR 032 uid59017
Bacillus licheniformis ATCC 14580 uid58097
Bacillus licheniformis ATCC 14580 uid58199
Bacillus atrophaeus 1942 uid59887
Bacillus subtilis spizizenii TU B 10 uid73967
Bacillus subtilis spizizenii W23 uid51879
Bacillus JS uid162189
Bacillus subtilis BSn5 uid62463
Bacillus subtilis RO NN 1 uid158879
Bacillus amyloliquefaciens LL3 uid158133
Bacillus amyloliquefaciens DSM 7 uid53535
Bacillus amyloliquefaciens TA208 uid158701
Bacillus amyloliquefaciens XH7 uid158881
Bacillus amyloliquefaciens plantarum CAU B946 uid84215
Bacillus amyloliquefaciens FZB42 uid58271
Bacillus amyloliquefaciens Y2 uid165195
Bacillus amyloliquefaciens plantarum YAU B9601 Y2 uid159001
Bacillus megaterium DSM319 uid48371
Bacillus megaterium QM B1551 uid15862
Bacillus megaterium WSH 002 uid159841
Bacillus cytotoxicus NVH 391 98 uid58317
Bacillus pseudofirmus OF4 uid45847
Bacillus weihenstephanensis KBAB4 uid58315
Bacillus cereus G9842 uid58759
Bacillus thuringiensis serovar chinensis CT 43 uid158151
Bacillus cereus ATCC 14579 uid57975
Bacillus cereus B4264 uid58757
Bacillus thuringiensis BMB171 uid49135
Bacillus cereus ATCC 10987 uid57673
Bacillus thuringiensis serovar finitimus YBT 020 uid158875
Bacillus cereus AH187 uid58753
Bacillus cereus NC7401 uid82815
Bacillus cereus Q1 uid58529
Bacillus anthracis H9401 uid162021
Bacillus anthracis CDC 684 uid59303
Bacillus anthracis Ames uid57909
Bacillus anthracis A0248 uid59385
Bacillus anthracis Sterne uid58091
Bacillus anthracis Ames Ancestor uid58083
Bacillus cereus biovar anthracis CI uid50615
Bacillus cereus E33L uid58103
Bacillus cereus AH820 uid58751
Bacillus cereus 03BB102 uid59299
Bacillus thuringiensis Al Hakam uid58795
Bacillus thuringiensis serovar konkukian 97 27 uid58089
rpoB

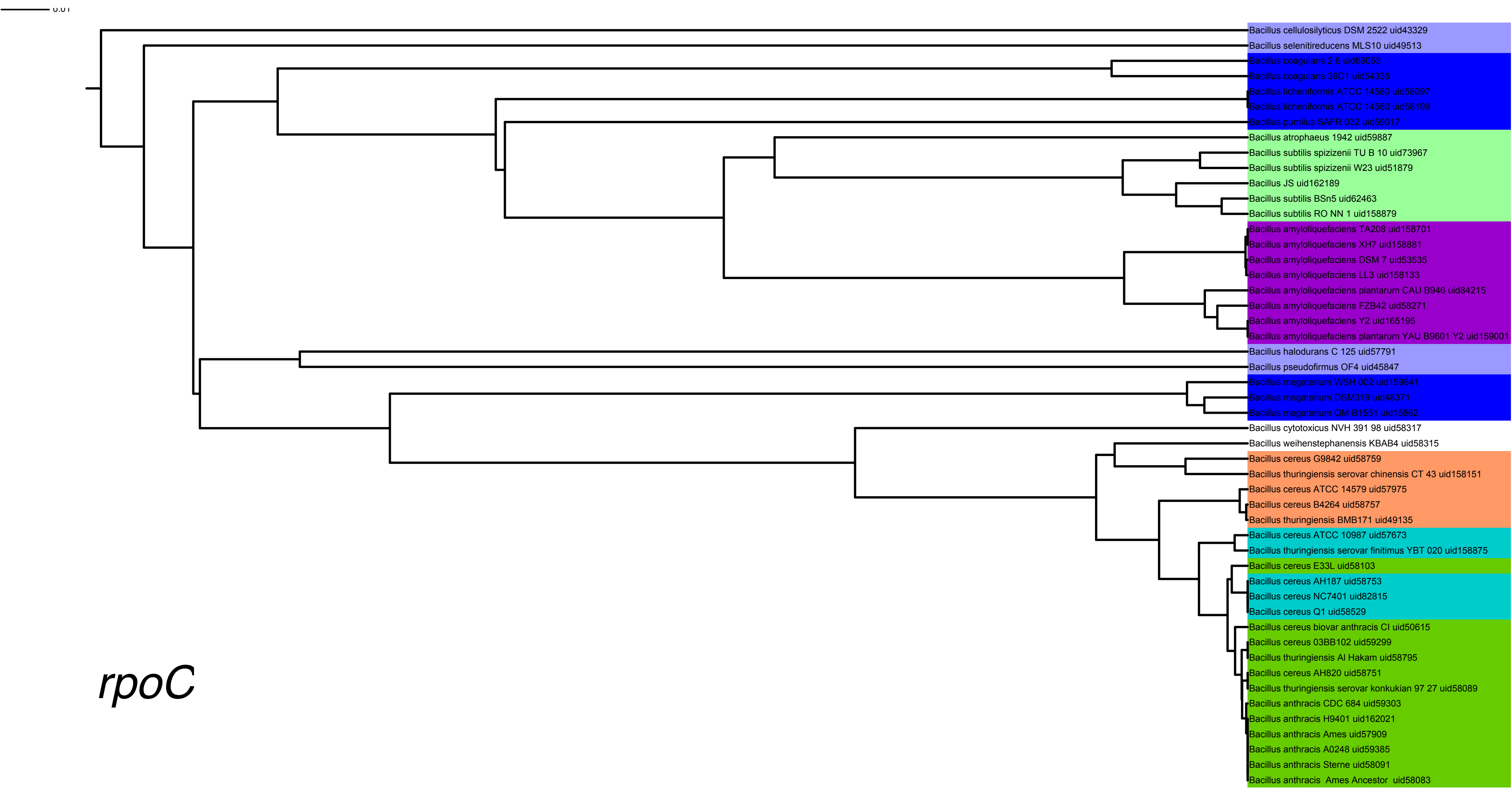
0.01
Bacillus cellulosilyticus DSM 2522 uid43329
Bacillus selenitireducens MLS10 uid49513
Bacillus coagulans 2 6 uid68053
Bacillus coagulans 36D1 uid54335
Bacillus licheniformis ATCC 14580 uid58097
Bacillus licheniformis ATCC 14580 uid58199
Bacillus pumilus SAFR 032 uid59017
Bacillus atrophaeus 1942 uid59887
Bacillus subtilis spizizenii TU B 10 uid73967
Bacillus subtilis spizizenii W23 uid51879
Bacillus JS uid162189
Bacillus subtilis BSn5 uid62463
Bacillus subtilis RO NN 1 uid158879
Bacillus amyloliquefaciens TA208 uid158701
Bacillus amyloliquefaciens XH7 uid158881
Bacillus amyloliquefaciens DSM 7 uid53535
Bacillus amyloliquefaciens LL3 uid158133
Bacillus amyloliquefaciens plantarum CAU B946 uid84215
Bacillus amyloliquefaciens FZB42 uid58271
Bacillus amyloliquefaciens Y2 uid165195
Bacillus amyloliquefaciens plantarum YAU B9601 Y2 uid159001
Bacillus halodurans C 125 uid57791
Bacillus pseudofirmus OF4 uid45847
Bacillus megaterium WSH 002 uid159841
Bacillus megaterium DSM319 uid48371
Bacillus megaterium QM B1551 uid15862
Bacillus cytotoxicus NVH 391 98 uid58317
Bacillus weihenstephanensis KBAB4 uid58315
Bacillus cereus G9842 uid58759
Bacillus thuringiensis serovar chinensis CT 43 uid158151
Bacillus cereus ATCC 14579 uid57975
Bacillus cereus B4264 uid58757
Bacillus thuringiensis BMB171 uid49135
Bacillus cereus ATCC 10987 uid57673
Bacillus thuringiensis serovar finitimus YBT 020 uid158875
Bacillus cereus E33L uid58103
Bacillus cereus AH187 uid58753
Bacillus cereus NC7401 uid82815
Bacillus cereus Q1 uid58529
Bacillus cereus biovar anthracis CI uid50615
Bacillus cereus 03BB102 uid59299
Bacillus thuringiensis Al Hakam uid58795
Bacillus cereus AH820 uid58751
Bacillus thuringiensis serovar konkukian 97 27 uid58089
Bacillus anthracis CDC 684 uid59303
Bacillus anthracis H9401 uid162021
Bacillus anthracis Ames uid57909
Bacillus anthracis A0248 uid59385
Bacillus anthracis Sterne uid58091
Bacillus anthracis  Ames Ancestor  uid58083
rpoC

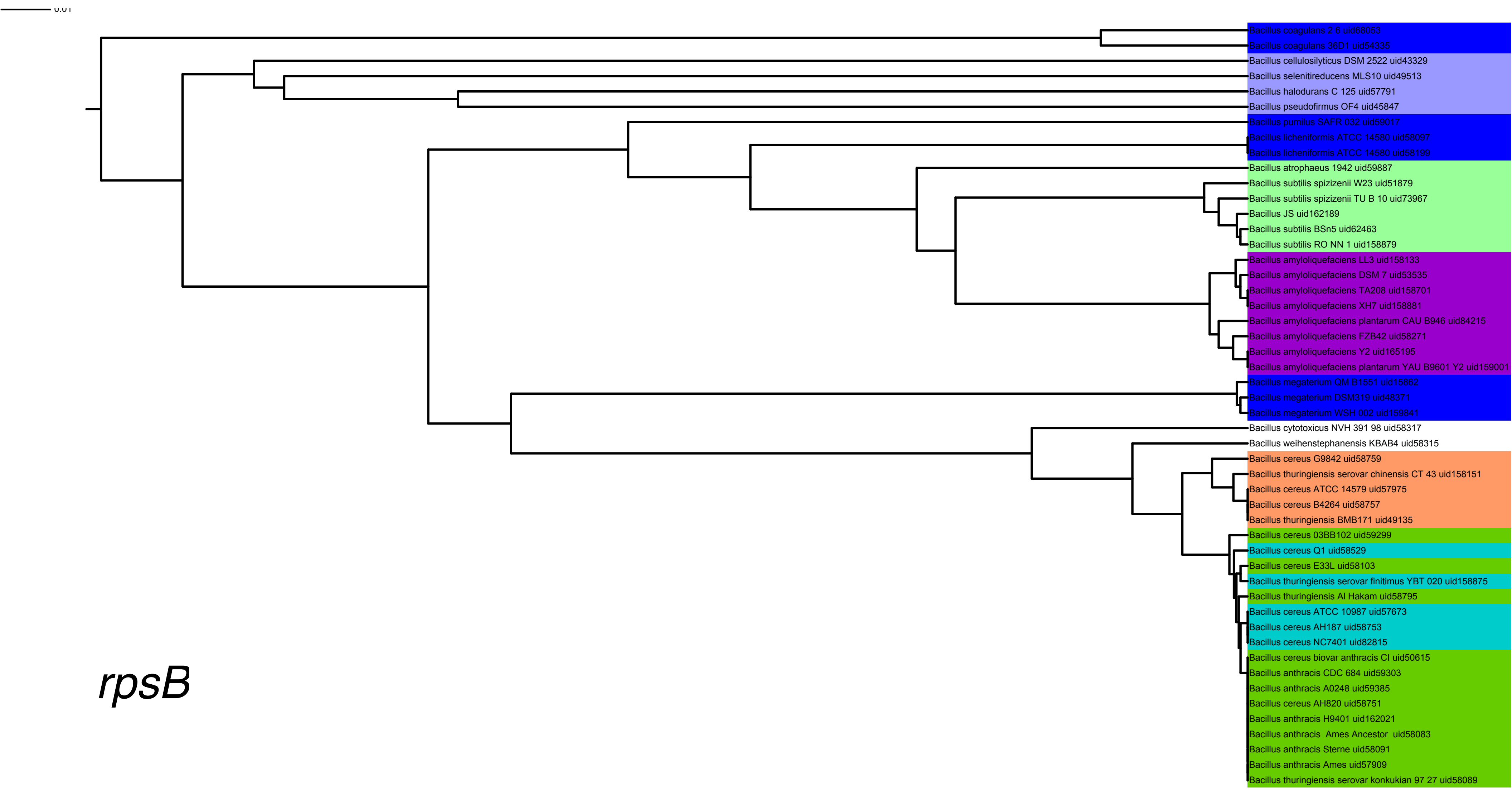
0.01
Bacillus coagulans 2 6 uid68053
Bacillus coagulans 36D1 uid54335
Bacillus cellulosilyticus DSM 2522 uid43329
Bacillus selenitireducens MLS10 uid49513
Bacillus halodurans C 125 uid57791
Bacillus pseudofirmus OF4 uid45847
Bacillus pumilus SAFR 032 uid59017
Bacillus licheniformis ATCC 14580 uid58097
Bacillus licheniformis ATCC 14580 uid58199
Bacillus atrophaeus 1942 uid59887
Bacillus subtilis spizizenii W23 uid51879
Bacillus subtilis spizizenii TU B 10 uid73967
Bacillus JS uid162189
Bacillus subtilis BSn5 uid62463
Bacillus subtilis RO NN 1 uid158879
Bacillus amyloliquefaciens LL3 uid158133
Bacillus amyloliquefaciens DSM 7 uid53535
Bacillus amyloliquefaciens TA208 uid158701
Bacillus amyloliquefaciens XH7 uid158881
Bacillus amyloliquefaciens plantarum CAU B946 uid84215
Bacillus amyloliquefaciens FZB42 uid58271
Bacillus amyloliquefaciens Y2 uid165195
Bacillus amyloliquefaciens plantarum YAU B9601 Y2 uid159001
Bacillus megaterium QM B1551 uid15862
Bacillus megaterium DSM319 uid48371
Bacillus megaterium WSH 002 uid159841
Bacillus cytotoxicus NVH 391 98 uid58317
Bacillus weihenstephanensis KBAB4 uid58315
Bacillus cereus G9842 uid58759
Bacillus thuringiensis serovar chinensis CT 43 uid158151
Bacillus cereus ATCC 14579 uid57975
Bacillus cereus B4264 uid58757
Bacillus thuringiensis BMB171 uid49135
Bacillus cereus 03BB102 uid59299
Bacillus cereus Q1 uid58529
Bacillus cereus E33L uid58103
Bacillus thuringiensis serovar finitimus YBT 020 uid158875
Bacillus thuringiensis Al Hakam uid58795
Bacillus cereus ATCC 10987 uid57673
Bacillus cereus AH187 uid58753
Bacillus cereus NC7401 uid82815
Bacillus cereus biovar anthracis CI uid50615
Bacillus anthracis CDC 684 uid59303
Bacillus anthracis A0248 uid59385
Bacillus cereus AH820 uid58751
Bacillus anthracis H9401 uid162021
Bacillus anthracis Ames Ancestor uid58083
Bacillus anthracis Sterne uid58091
Bacillus anthracis Ames uid57909
Bacillus thuringiensis serovar konkukian 97 27 uid58089
rpsB

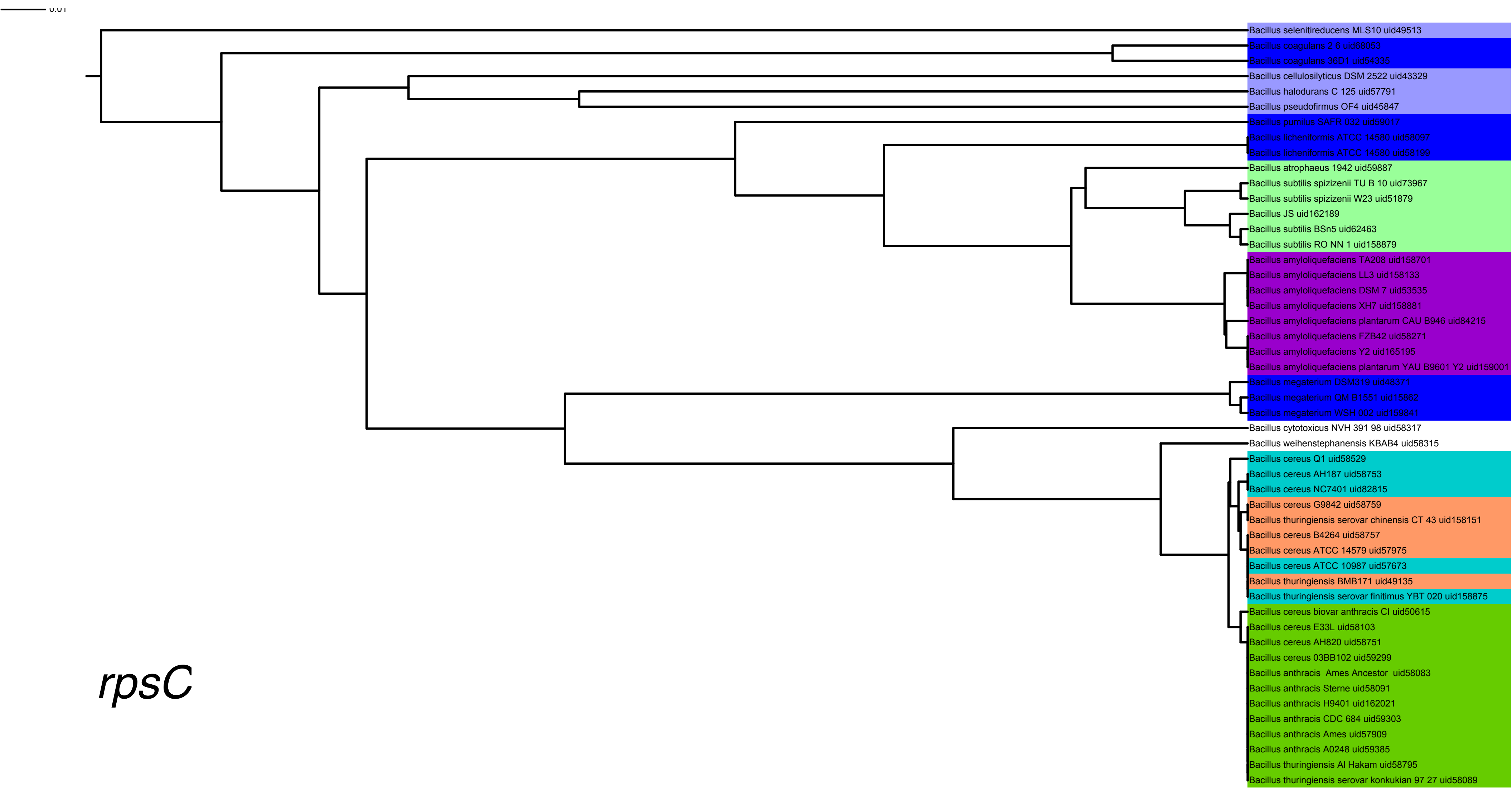
0.01
Bacillus selenitireducens MLS10 uid49513
Bacillus coagulans 2 6 uid68053
Bacillus coagulans 36D1 uid54335
Bacillus cellulosilyticus DSM 2522 uid43329
Bacillus halodurans C 125 uid57791
Bacillus pseudofirmus OF4 uid45847
Bacillus pumilus SAFR 032 uid59017
Bacillus licheniformis ATCC 14580 uid58097
Bacillus licheniformis ATCC 14580 uid58199
Bacillus atrophaeus 1942 uid59887
Bacillus subtilis spizizenii TU B 10 uid73967
Bacillus subtilis spizizenii W23 uid51879
Bacillus JS uid162189
Bacillus subtilis BSn5 uid62463
Bacillus subtilis RO NN 1 uid158879
Bacillus amyloliquefaciens TA208 uid158701
Bacillus amyloliquefaciens LL3 uid158133
Bacillus amyloliquefaciens DSM 7 uid53535
Bacillus amyloliquefaciens XH7 uid158881
Bacillus amyloliquefaciens plantarum CAU B946 uid84215
Bacillus amyloliquefaciens FZB42 uid58271
Bacillus amyloliquefaciens Y2 uid165195
Bacillus amyloliquefaciens plantarum YAU B9601 Y2 uid159001
Bacillus megaterium DSM319 uid48371
Bacillus megaterium QM B1551 uid15862
Bacillus megaterium WSH 002 uid159841
Bacillus cytotoxicus NVH 391 98 uid58317
Bacillus weihenstephanensis KBAB4 uid58315
Bacillus cereus Q1 uid58529
Bacillus cereus AH187 uid58753
Bacillus cereus NC7401 uid82815
Bacillus cereus G9842 uid58759
Bacillus thuringiensis serovar chinensis CT 43 uid158151
Bacillus cereus B4264 uid58757
Bacillus cereus ATCC 14579 uid57975
Bacillus cereus ATCC 10987 uid57673
Bacillus thuringiensis BMB171 uid49135
Bacillus thuringiensis serovar finitimus YBT 020 uid158875
Bacillus cereus biovar anthracis CI uid50615
Bacillus cereus E33L uid58103
Bacillus cereus AH820 uid58751
Bacillus cereus 03BB102 uid59299
Bacillus anthracis Ames Ancestor uid58083
Bacillus anthracis Sterne uid58091
Bacillus anthracis H9401 uid162021
Bacillus anthracis CDC 684 uid59303
Bacillus anthracis Ames uid57909
Bacillus anthracis A0248 uid59385
Bacillus thuringiensis Al Hakam uid58795
Bacillus thuringiensis serovar konkukian 97 27 uid58089
rpsC

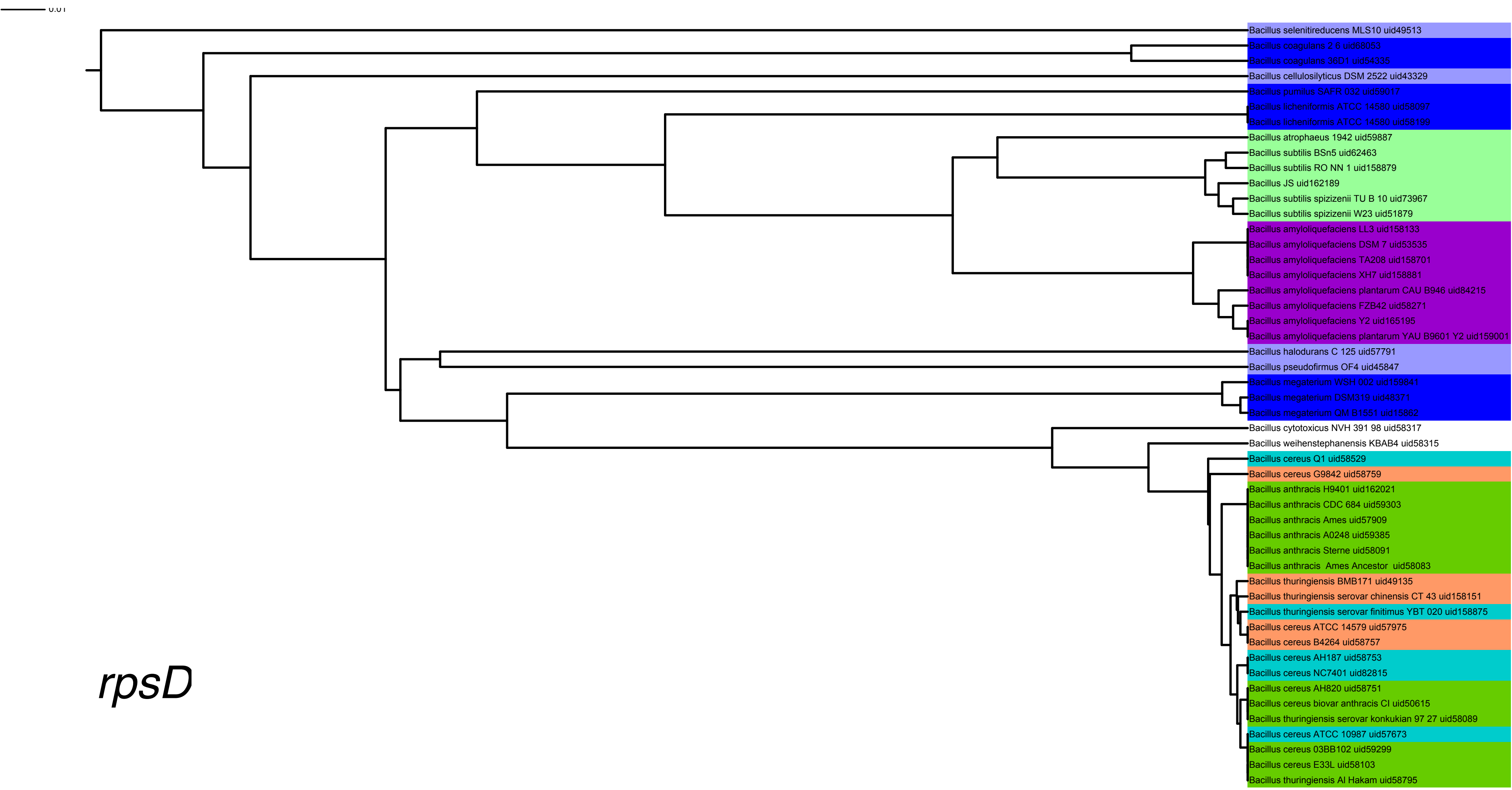
0.01
Bacillus selenitireducens MLS10 uid49513
Bacillus coagulans 2 6 uid68053
Bacillus coagulans 36D1 uid54335
Bacillus cellulosilyticus DSM 2522 uid43329
Bacillus pumilus SAFR 032 uid59017
Bacillus licheniformis ATCC 14580 uid58097
Bacillus licheniformis ATCC 14580 uid58199
Bacillus atrophaeus 1942 uid59887
Bacillus subtilis BSn5 uid62463
Bacillus subtilis RO NN 1 uid158879
Bacillus JS uid162189
Bacillus subtilis spizizenii TU B 10 uid73967
Bacillus subtilis spizizenii W23 uid51879
Bacillus amyloliquefaciens LL3 uid158133
Bacillus amyloliquefaciens DSM 7 uid53535
Bacillus amyloliquefaciens TA208 uid158701
Bacillus amyloliquefaciens XH7 uid158881
Bacillus amyloliquefaciens plantarum CAU B946 uid84215
Bacillus amyloliquefaciens FZB42 uid58271
Bacillus amyloliquefaciens Y2 uid165195
Bacillus amyloliquefaciens plantarum YAU B9601 Y2 uid159001
Bacillus halodurans C 125 uid57791
Bacillus pseudofirmus OF4 uid45847
Bacillus megaterium WSH 002 uid159841
Bacillus megaterium DSM319 uid48371
Bacillus megaterium QM B1551 uid15862
Bacillus cytotoxicus NVH 391 98 uid58317
Bacillus weihenstephanensis KBAB4 uid58315
Bacillus cereus Q1 uid58529
Bacillus cereus G9842 uid58759
Bacillus anthracis H9401 uid162021
Bacillus anthracis CDC 684 uid59303
Bacillus anthracis Ames uid57909
Bacillus anthracis A0248 uid59385
Bacillus anthracis Sterne uid58091
Bacillus anthracis Ames Ancestor uid58083
Bacillus thuringiensis BMB171 uid49135
Bacillus thuringiensis serovar chinensis CT 43 uid158151
Bacillus thuringiensis serovar finitimus YBT 020 uid158875
Bacillus cereus ATCC 14579 uid57975
Bacillus cereus B4264 uid58757
Bacillus cereus AH187 uid58753
Bacillus cereus NC7401 uid82815
Bacillus cereus AH820 uid58751
Bacillus cereus biovar anthracis CI uid50615
Bacillus thuringiensis serovar konkukian 97 27 uid58089
Bacillus cereus ATCC 10987 uid57673
Bacillus cereus 03BB102 uid59299
Bacillus cereus E33L uid58103
Bacillus thuringiensis Al Hakam uid58795
rpsD

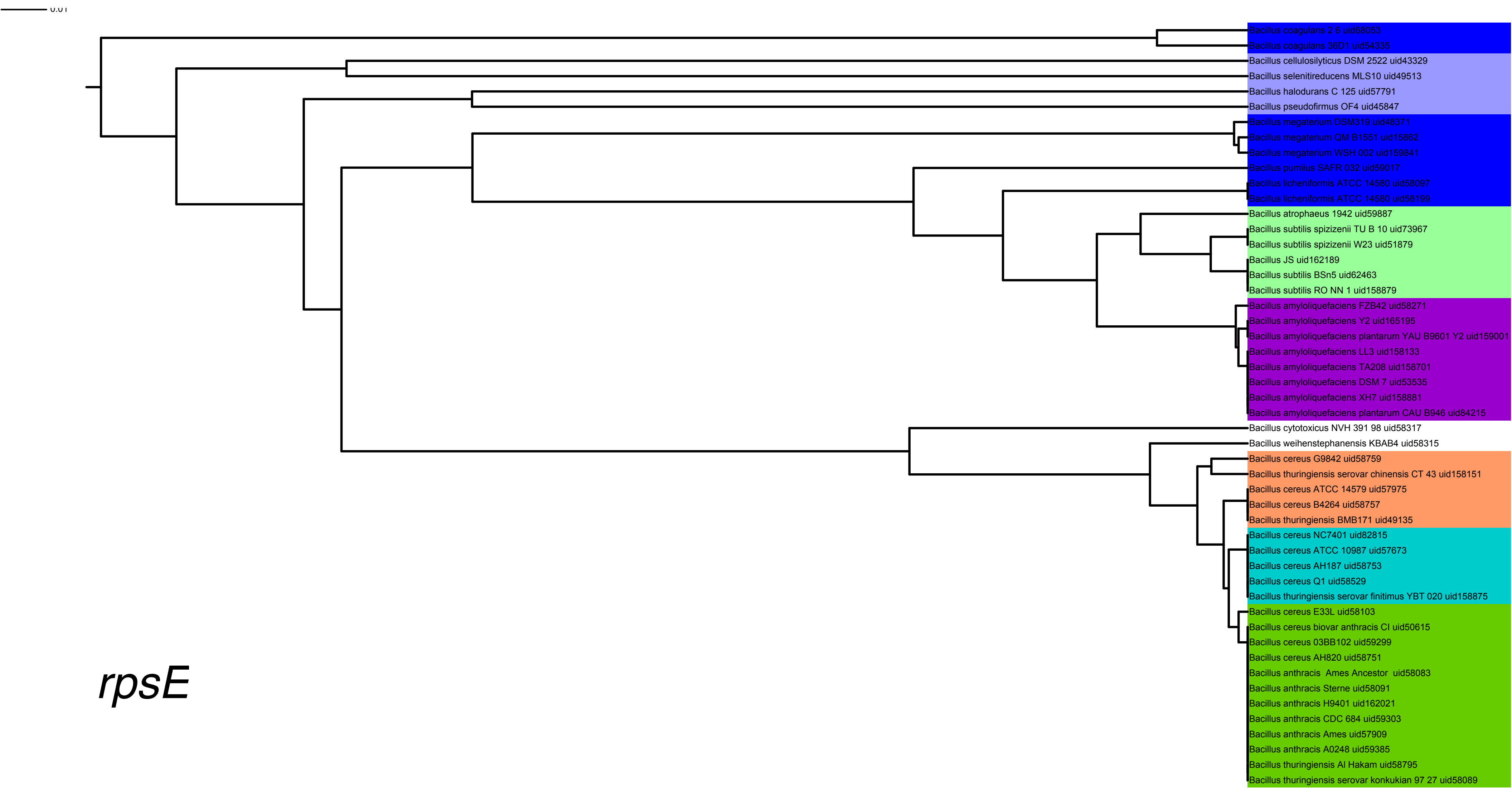
0.01
Bacillus coagulans 2 6 uid68053
Bacillus coagulans 36D1 uid54335
Bacillus cellulosilyticus DSM 2522 uid43329
Bacillus selenitireducens MLS10 uid49513
Bacillus halodurans C 125 uid57791
Bacillus pseudofirmus OF4 uid45847
Bacillus megaterium DSM319 uid48371
Bacillus megaterium QM B1551 uid15862
Bacillus megaterium WSH 002 uid159841
Bacillus pumilus SAFR 032 uid59017
Bacillus licheniformis ATCC 14580 uid58097
Bacillus licheniformis ATCC 14580 uid58199
Bacillus atrophaeus 1942 uid59887
Bacillus subtilis spizizenii TU B 10 uid73967
Bacillus subtilis spizizenii W23 uid51879
Bacillus JS uid162189
Bacillus subtilis BSn5 uid62463
Bacillus subtilis RO NN 1 uid158879
Bacillus amyloliquefaciens FZB42 uid58271
Bacillus amyloliquefaciens Y2 uid165195
Bacillus amyloliquefaciens plantarum YAU B9601 Y2 uid159001
Bacillus amyloliquefaciens LL3 uid158133
Bacillus amyloliquefaciens TA208 uid158701
Bacillus amyloliquefaciens DSM 7 uid53535
Bacillus amyloliquefaciens XH7 uid158881
Bacillus amyloliquefaciens plantarum CAU B946 uid84215
Bacillus cytotoxicus NVH 391 98 uid58317
Bacillus weihenstephanensis KBAB4 uid58315
Bacillus cereus G9842 uid58759
Bacillus thuringiensis serovar chinensis CT 43 uid158151
Bacillus cereus ATCC 14579 uid57975
Bacillus cereus B4264 uid58757
Bacillus thuringiensis BMB171 uid49135
Bacillus cereus NC7401 uid82815
Bacillus cereus ATCC 10987 uid57673
Bacillus cereus AH187 uid58753
Bacillus cereus Q1 uid58529
Bacillus thuringiensis serovar finitimus YBT 020 uid158875
Bacillus cereus E33L uid58103
Bacillus cereus biovar anthracis CI uid50615
Bacillus cereus 03BB102 uid59299
Bacillus cereus AH820 uid58751
Bacillus anthracis Ames Ancestor uid58083
Bacillus anthracis Sterne uid58091
Bacillus anthracis H9401 uid162021
Bacillus anthracis CDC 684 uid59303
Bacillus anthracis Ames uid57909
Bacillus anthracis A0248 uid59385
Bacillus thuringiensis Al Hakam uid58795
Bacillus thuringiensis serovar konkukian 97 27 uid58089
rpsE

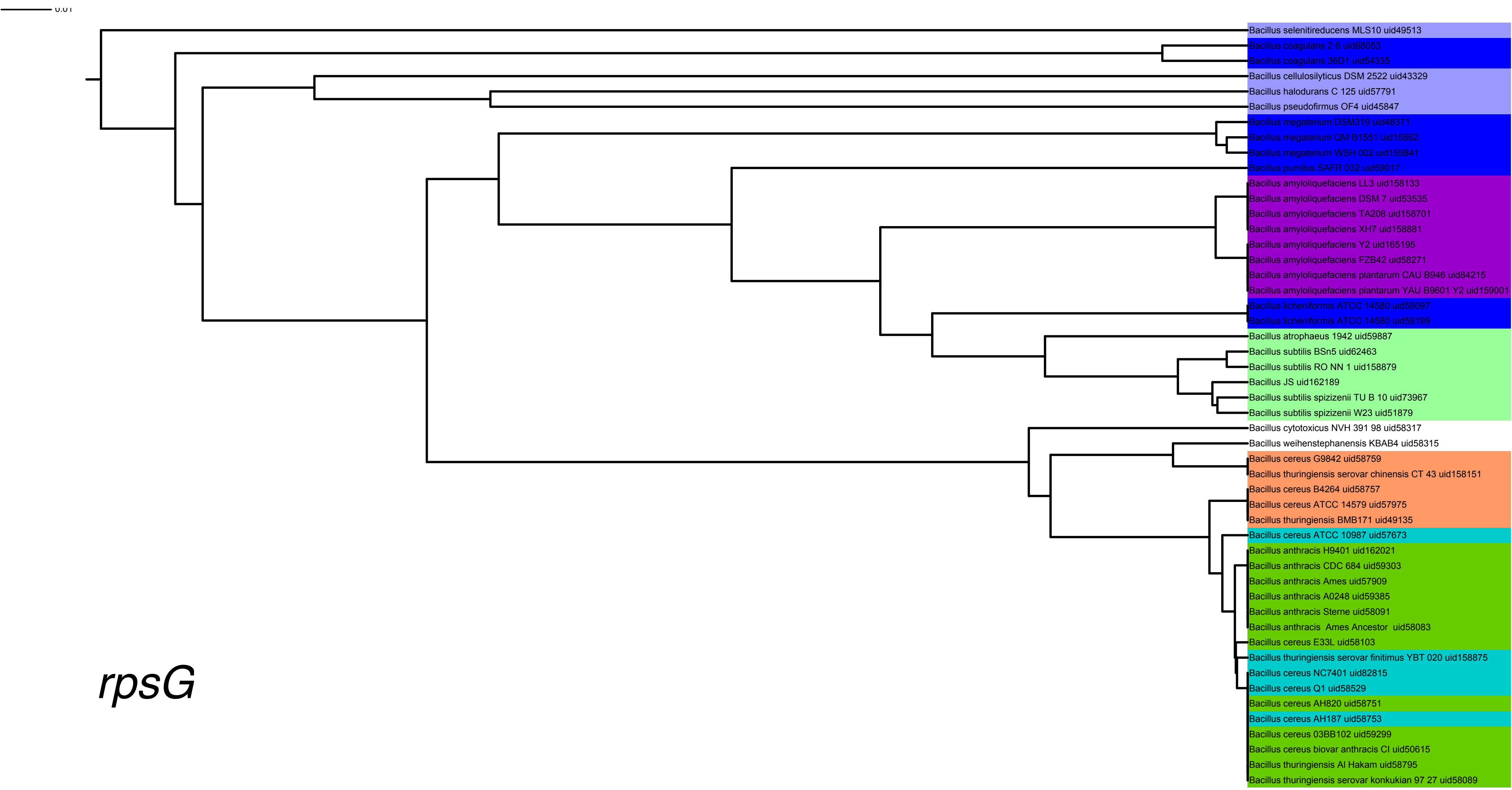
0.01
Bacillus selenitireducens MLS10 uid49513
Bacillus coagulans 2 6 uid68053
Bacillus coagulans 36D1 uid54335
Bacillus cellulosilyticus DSM 2522 uid43329
Bacillus halodurans C 125 uid57791
Bacillus pseudofirmus OF4 uid45847
Bacillus megaterium DSM319 uid48371
Bacillus megaterium QM B1551 uid15862
Bacillus megaterium WSH 002 uid159841
Bacillus pumilus SAFR 032 uid59017
Bacillus amyloliquefaciens LL3 uid158133
Bacillus amyloliquefaciens DSM 7 uid53535
Bacillus amyloliquefaciens TA208 uid158701
Bacillus amyloliquefaciens XH7 uid158881
Bacillus amyloliquefaciens Y2 uid165195
Bacillus amyloliquefaciens FZB42 uid58271
Bacillus amyloliquefaciens plantarum CAU B946 uid84215
Bacillus amyloliquefaciens plantarum YAU B9601 Y2 uid159001
Bacillus licheniformis ATCC 14580 uid58097
Bacillus licheniformis ATCC 14580 uid58199
Bacillus atrophaeus 1942 uid59887
Bacillus subtilis BSn5 uid62463
Bacillus subtilis RO NN 1 uid158879
Bacillus JS uid162189
Bacillus subtilis spizizenii TU B 10 uid73967
Bacillus subtilis spizizenii W23 uid51879
Bacillus cytotoxicus NVH 391 98 uid58317
Bacillus weihenstephanensis KBAB4 uid58315
Bacillus cereus G9842 uid58759
Bacillus thuringiensis serovar chinensis CT 43 uid158151
Bacillus cereus B4264 uid58757
Bacillus cereus ATCC 14579 uid57975
Bacillus thuringiensis BMB171 uid49135
Bacillus cereus ATCC 10987 uid57673
Bacillus anthracis H9401 uid162021
Bacillus anthracis CDC 684 uid59303
Bacillus anthracis Ames uid57909
Bacillus anthracis A0248 uid59385
Bacillus anthracis Sterne uid58091
Bacillus anthracis Ames Ancestor uid58083
Bacillus cereus E33L uid58103
Bacillus thuringiensis serovar finitimus YBT 020 uid158875
Bacillus cereus NC7401 uid82815
Bacillus cereus Q1 uid58529
Bacillus cereus AH820 uid58751
Bacillus cereus AH187 uid58753
Bacillus cereus 03BB102 uid59299
Bacillus cereus biovar anthracis CI uid50615
Bacillus thuringiensis Al Hakam uid58795
Bacillus thuringiensis serovar konkukian 97 27 uid58089
rpsG

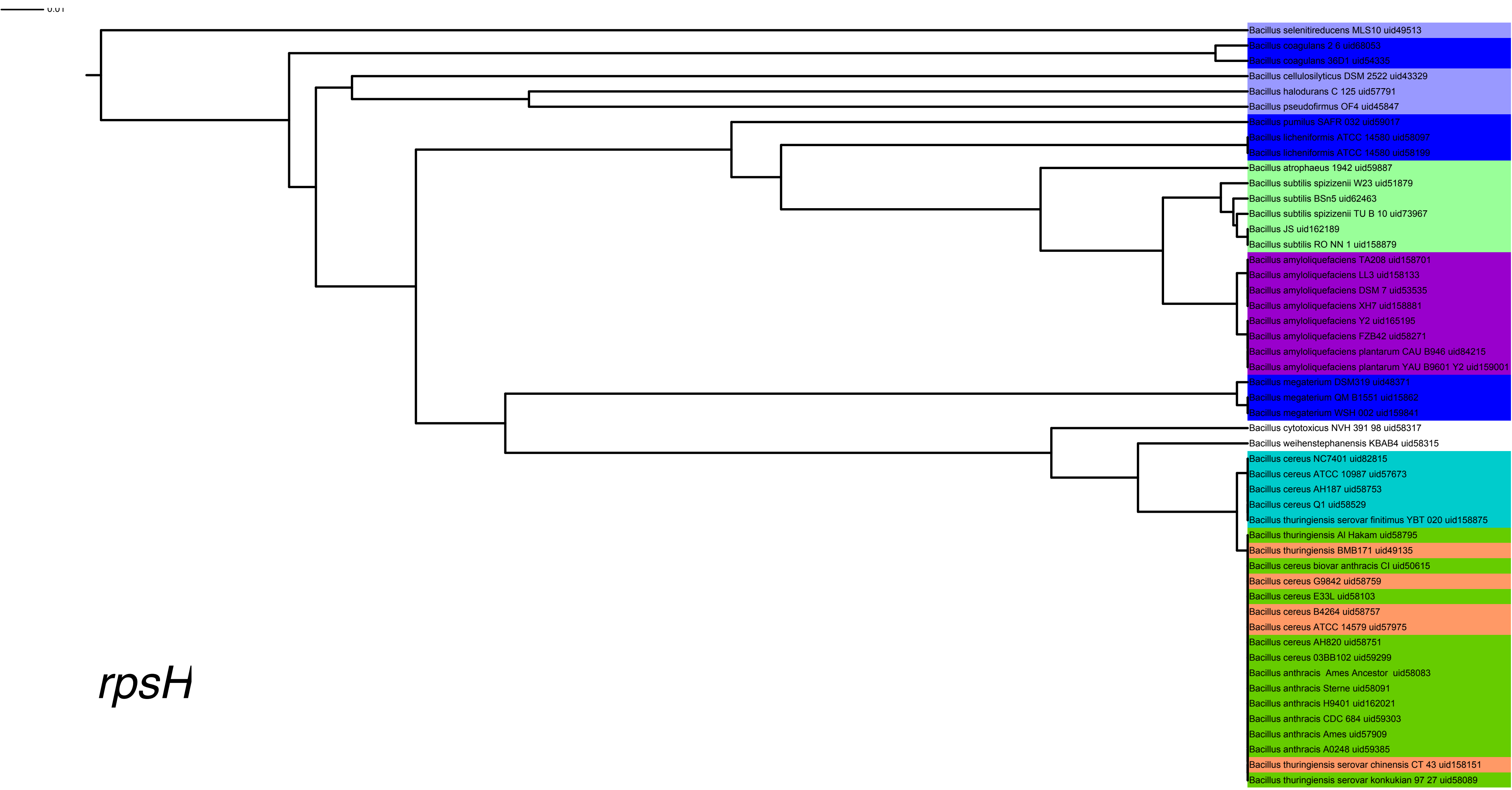
0.01
Bacillus selenitireducens MLS10 uid49513
Bacillus coagulans 2 6 uid68053
Bacillus coagulans 36D1 uid54335
Bacillus cellulosilyticus DSM 2522 uid43329
Bacillus halodurans C 125 uid57791
Bacillus pseudofirmus OF4 uid45847
Bacillus pumilus SAFR 032 uid59017
Bacillus licheniformis ATCC 14580 uid58097
Bacillus licheniformis ATCC 14580 uid58199
Bacillus atrophaeus 1942 uid59887
Bacillus subtilis spizizenii W23 uid51879
Bacillus subtilis BSn5 uid62463
Bacillus subtilis spizizenii TU B 10 uid73967
Bacillus JS uid162189
Bacillus subtilis RO NN 1 uid158879
Bacillus amyloliquefaciens TA208 uid158701
Bacillus amyloliquefaciens LL3 uid158133
Bacillus amyloliquefaciens DSM 7 uid53535
Bacillus amyloliquefaciens XH7 uid158881
Bacillus amyloliquefaciens Y2 uid165195
Bacillus amyloliquefaciens FZB42 uid58271
Bacillus amyloliquefaciens plantarum CAU B946 uid84215
Bacillus amyloliquefaciens plantarum YAU B9601 Y2 uid159001
Bacillus megaterium DSM319 uid48371
Bacillus megaterium QM B1551 uid15862
Bacillus megaterium WSH 002 uid159841
Bacillus cytotoxicus NVH 391 98 uid58317
Bacillus weihenstephanensis KBAB4 uid58315
Bacillus cereus NC7401 uid82815
Bacillus cereus ATCC 10987 uid57673
Bacillus cereus AH187 uid58753
Bacillus cereus Q1 uid58529
Bacillus thuringiensis serovar finitimus YBT 020 uid158875
Bacillus thuringiensis Al Hakam uid58795
Bacillus thuringiensis BMB171 uid49135
Bacillus cereus biovar anthracis CI uid50615
Bacillus cereus G9842 uid58759
Bacillus cereus E33L uid58103
Bacillus cereus B4264 uid58757
Bacillus cereus ATCC 14579 uid57975
Bacillus cereus AH820 uid58751
Bacillus cereus 03BB102 uid59299
Bacillus anthracis Ames Ancestor uid58083
Bacillus anthracis Sterne uid58091
Bacillus anthracis H9401 uid162021
Bacillus anthracis CDC 684 uid59303
Bacillus anthracis Ames uid57909
Bacillus anthracis A0248 uid59385
Bacillus thuringiensis serovar chinensis CT 43 uid158151
Bacillus thuringiensis serovar konkukian 97 27 uid58089

*rpsH*

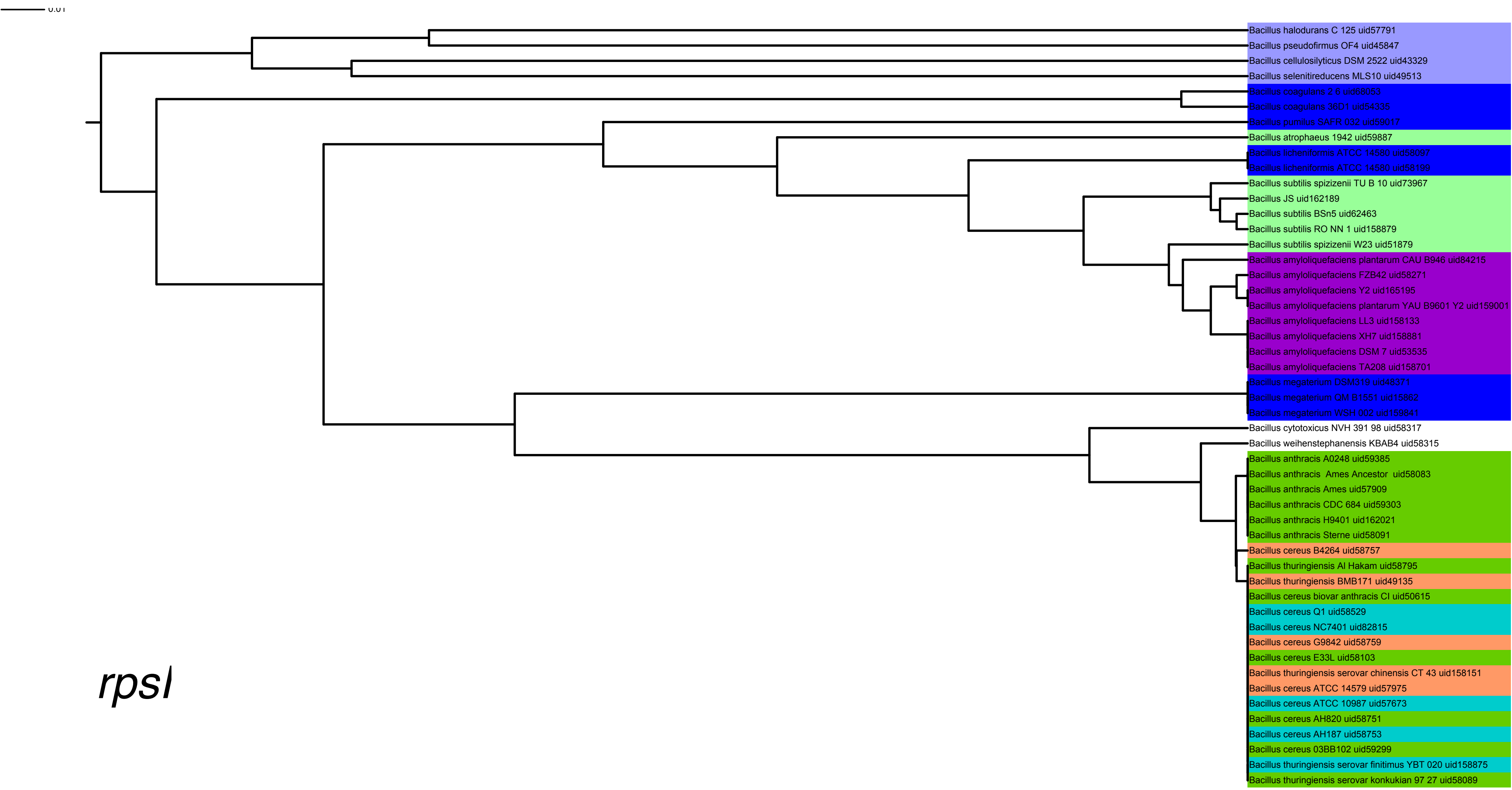
0.01
Bacillus halodurans C 125 uid57791
Bacillus pseudofirmus OF4 uid45847
Bacillus cellulosilyticus DSM 2522 uid43329
Bacillus selenitireducens MLS10 uid49513
Bacillus coagulans 2 6 uid68053
Bacillus coagulans 36D1 uid54335
Bacillus pumilus SAFR 032 uid59017
Bacillus atrophaeus 1942 uid59887
Bacillus licheniformis ATCC 14580 uid58097
Bacillus licheniformis ATCC 14580 uid58199
Bacillus subtilis spizizenii TU B 10 uid73967
Bacillus JS uid162189
Bacillus subtilis BSn5 uid62463
Bacillus subtilis RO NN 1 uid158879
Bacillus subtilis spizizenii W23 uid51879
Bacillus amyloliquefaciens plantarum CAU B946 uid84215
Bacillus amyloliquefaciens FZB42 uid58271
Bacillus amyloliquefaciens Y2 uid165195
Bacillus amyloliquefaciens plantarum YAU B9601 Y2 uid159001
Bacillus amyloliquefaciens LL3 uid158133
Bacillus amyloliquefaciens XH7 uid158881
Bacillus amyloliquefaciens DSM 7 uid53535
Bacillus amyloliquefaciens TA208 uid158701
Bacillus megaterium DSM319 uid48371
Bacillus megaterium QM B1551 uid15862
Bacillus megaterium WSH 002 uid159841
Bacillus cytotoxicus NVH 391 98 uid58317
Bacillus weihenstephanensis KBAB4 uid58315
Bacillus anthracis A0248 uid59385
Bacillus anthracis Ames Ancestor uid58083
Bacillus anthracis Ames uid57909
Bacillus anthracis CDC 684 uid59303
Bacillus anthracis H9401 uid162021
Bacillus anthracis Sterne uid58091
Bacillus cereus B4264 uid58757
Bacillus thuringiensis Al Hakam uid58795
Bacillus thuringiensis BMB171 uid49135
Bacillus cereus biovar anthracis CI uid50615
Bacillus cereus Q1 uid58529
Bacillus cereus NC7401 uid82815
Bacillus cereus G9842 uid58759
Bacillus cereus E33L uid58103
Bacillus thuringiensis serovar chinensis CT 43 uid158151
Bacillus cereus ATCC 14579 uid57975
Bacillus cereus ATCC 10987 uid57673
Bacillus cereus AH820 uid58751
Bacillus cereus AH187 uid58753
Bacillus cereus 03BB102 uid59299
Bacillus thuringiensis serovar finitimus YBT 020 uid158875
Bacillus thuringiensis serovar konkukian 97 27 uid58089
rpsI

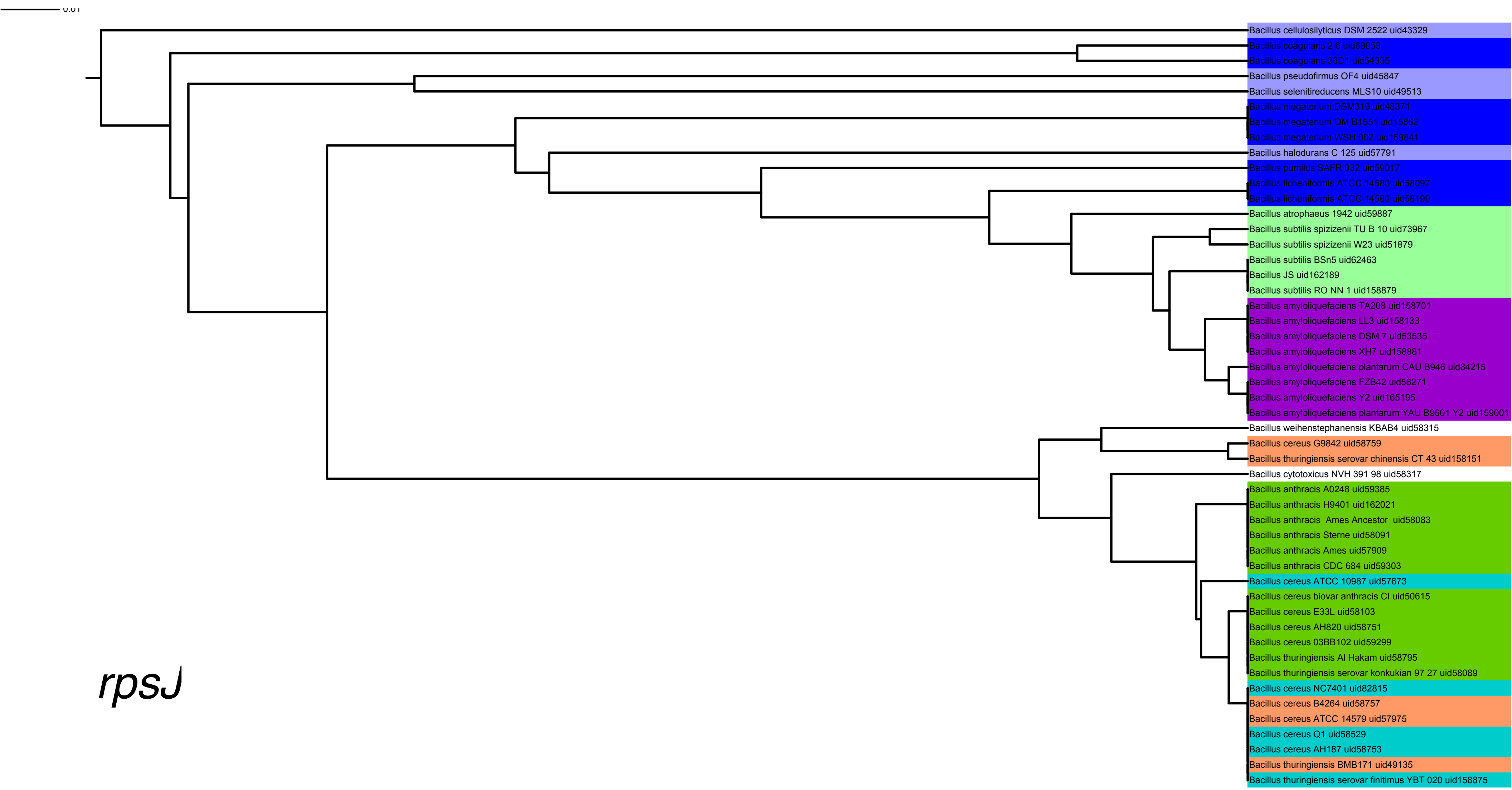
0.01
Bacillus cellulosilyticus DSM 2522 uid43329
Bacillus coagulans 2 6 uid68053
Bacillus coagulans 36D1 uid54335
Bacillus pseudofirmus OF4 uid45847
Bacillus selenitireducens MLS10 uid49513
Bacillus megaterium DSM319 uid48371
Bacillus megaterium QM B1551 uid15862
Bacillus megaterium WSH 002 uid159841
Bacillus halodurans C 125 uid57791
Bacillus pumilus SAFR 032 uid59017
Bacillus licheniformis ATCC 14580 uid58097
Bacillus licheniformis ATCC 14580 uid58199
Bacillus atrophaeus 1942 uid59887
Bacillus subtilis spizizenii TU B 10 uid73967
Bacillus subtilis spizizenii W23 uid51879
Bacillus subtilis BSn5 uid62463
Bacillus JS uid162189
Bacillus subtilis RO NN 1 uid158879
Bacillus amyloliquefaciens TA208 uid158701
Bacillus amyloliquefaciens LL3 uid158133
Bacillus amyloliquefaciens DSM 7 uid53535
Bacillus amyloliquefaciens XH7 uid158881
Bacillus amyloliquefaciens plantarum CAU B946 uid84215
Bacillus amyloliquefaciens FZB42 uid58271
Bacillus amyloliquefaciens Y2 uid165195
Bacillus amyloliquefaciens plantarum YAU B9601 Y2 uid159001
Bacillus weihenstephanensis KBAB4 uid58315
Bacillus cereus G9842 uid58759
Bacillus thuringiensis serovar chinensis CT 43 uid158151
Bacillus cytotoxicus NVH 391 98 uid58317
Bacillus anthracis A0248 uid59385
Bacillus anthracis H9401 uid162021
Bacillus anthracis Ames Ancestor uid58083
Bacillus anthracis Sterne uid58091
Bacillus anthracis Ames uid57909
Bacillus anthracis CDC 684 uid59303
Bacillus cereus ATCC 10987 uid57673
Bacillus cereus biovar anthracis CI uid50615
Bacillus cereus E33L uid58103
Bacillus cereus AH820 uid58751
Bacillus cereus 03BB102 uid59299
Bacillus thuringiensis Al Hakam uid58795
Bacillus thuringiensis serovar konkukian 97 27 uid58089
Bacillus cereus NC7401 uid82815
Bacillus cereus B4264 uid58757
Bacillus cereus ATCC 14579 uid57975
Bacillus cereus Q1 uid58529
Bacillus cereus AH187 uid58753
Bacillus thuringiensis BMB171 uid49135
Bacillus thuringiensis serovar finitimus YBT 020 uid158875
rpsJ

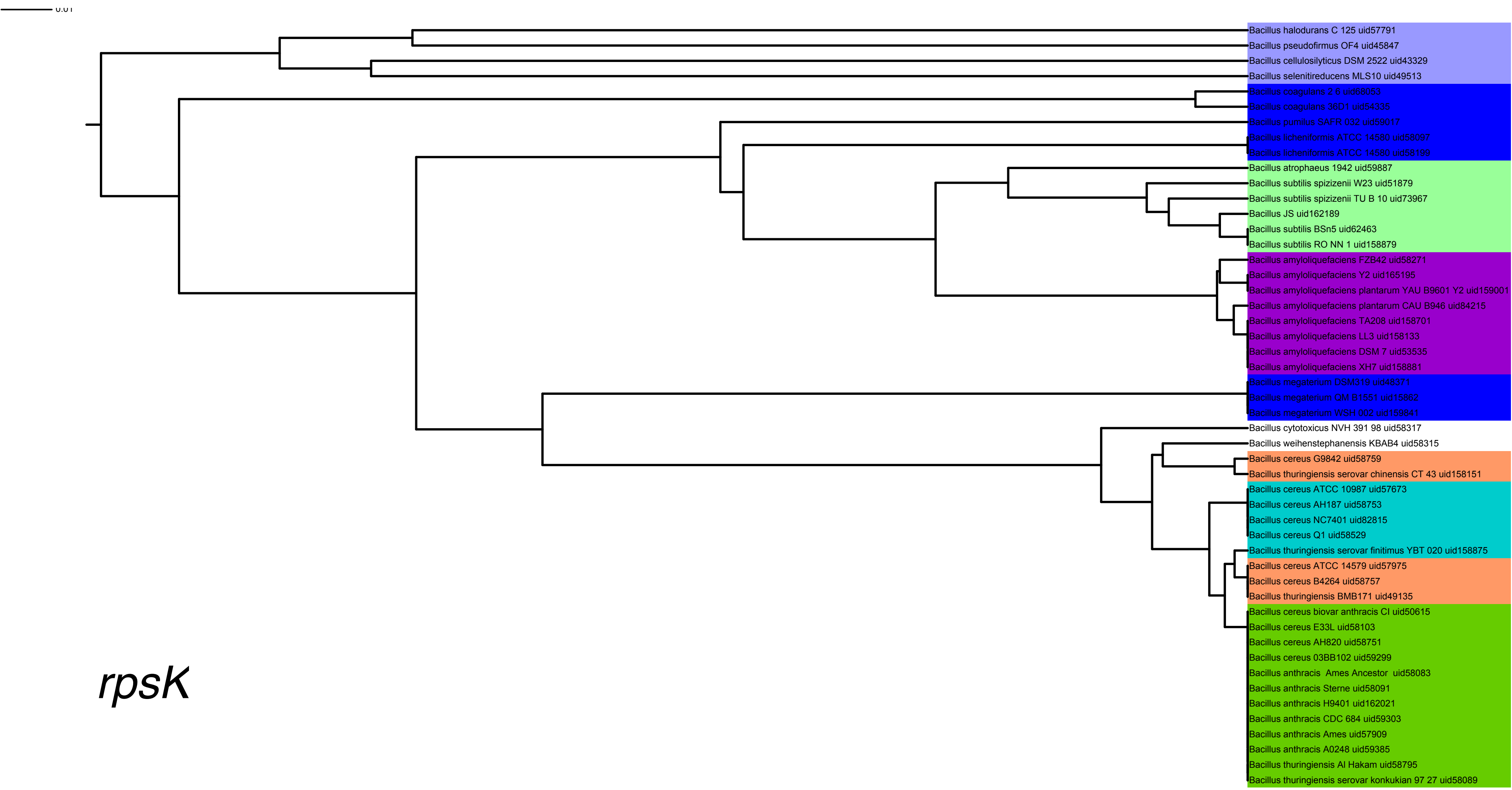
0.01
Bacillus halodurans C 125 uid57791
Bacillus pseudofirmus OF4 uid45847
Bacillus cellulosilyticus DSM 2522 uid43329
Bacillus selenitireducens MLS10 uid49513
Bacillus coagulans 2 6 uid68053
Bacillus coagulans 36D1 uid54335
Bacillus pumilus SAFR 032 uid59017
Bacillus licheniformis ATCC 14580 uid58097
Bacillus licheniformis ATCC 14580 uid58199
Bacillus atrophaeus 1942 uid59887
Bacillus subtilis spizizenii W23 uid51879
Bacillus subtilis spizizenii TU B 10 uid73967
Bacillus JS uid162189
Bacillus subtilis BSn5 uid62463
Bacillus subtilis RO NN 1 uid158879
Bacillus amyloliquefaciens FZB42 uid58271
Bacillus amyloliquefaciens Y2 uid165195
Bacillus amyloliquefaciens plantarum YAU B9601 Y2 uid159001
Bacillus amyloliquefaciens plantarum CAU B946 uid84215
Bacillus amyloliquefaciens TA208 uid158701
Bacillus amyloliquefaciens LL3 uid158133
Bacillus amyloliquefaciens DSM 7 uid53535
Bacillus amyloliquefaciens XH7 uid158881
Bacillus megaterium DSM319 uid48371
Bacillus megaterium QM B1551 uid15862
Bacillus megaterium WSH 002 uid159841
Bacillus cytotoxicus NVH 391 98 uid58317
Bacillus weihenstephanensis KBAB4 uid58315
Bacillus cereus G9842 uid58759
Bacillus thuringiensis serovar chinensis CT 43 uid158151
Bacillus cereus ATCC 10987 uid57673
Bacillus cereus AH187 uid58753
Bacillus cereus NC7401 uid82815
Bacillus cereus Q1 uid58529
Bacillus thuringiensis serovar finitimus YBT 020 uid158875
Bacillus cereus ATCC 14579 uid57975
Bacillus cereus B4264 uid58757
Bacillus thuringiensis BMB171 uid49135
Bacillus cereus biovar anthracis CI uid50615
Bacillus cereus E33L uid58103
Bacillus cereus AH820 uid58751
Bacillus cereus 03BB102 uid59299
Bacillus anthracis Ames Ancestor uid58083
Bacillus anthracis Sterne uid58091
Bacillus anthracis H9401 uid162021
Bacillus anthracis CDC 684 uid59303
Bacillus anthracis Ames uid57909
Bacillus anthracis A0248 uid59385
Bacillus thuringiensis Al Hakam uid58795
Bacillus thuringiensis serovar konkukian 97 27 uid58089
rpsK

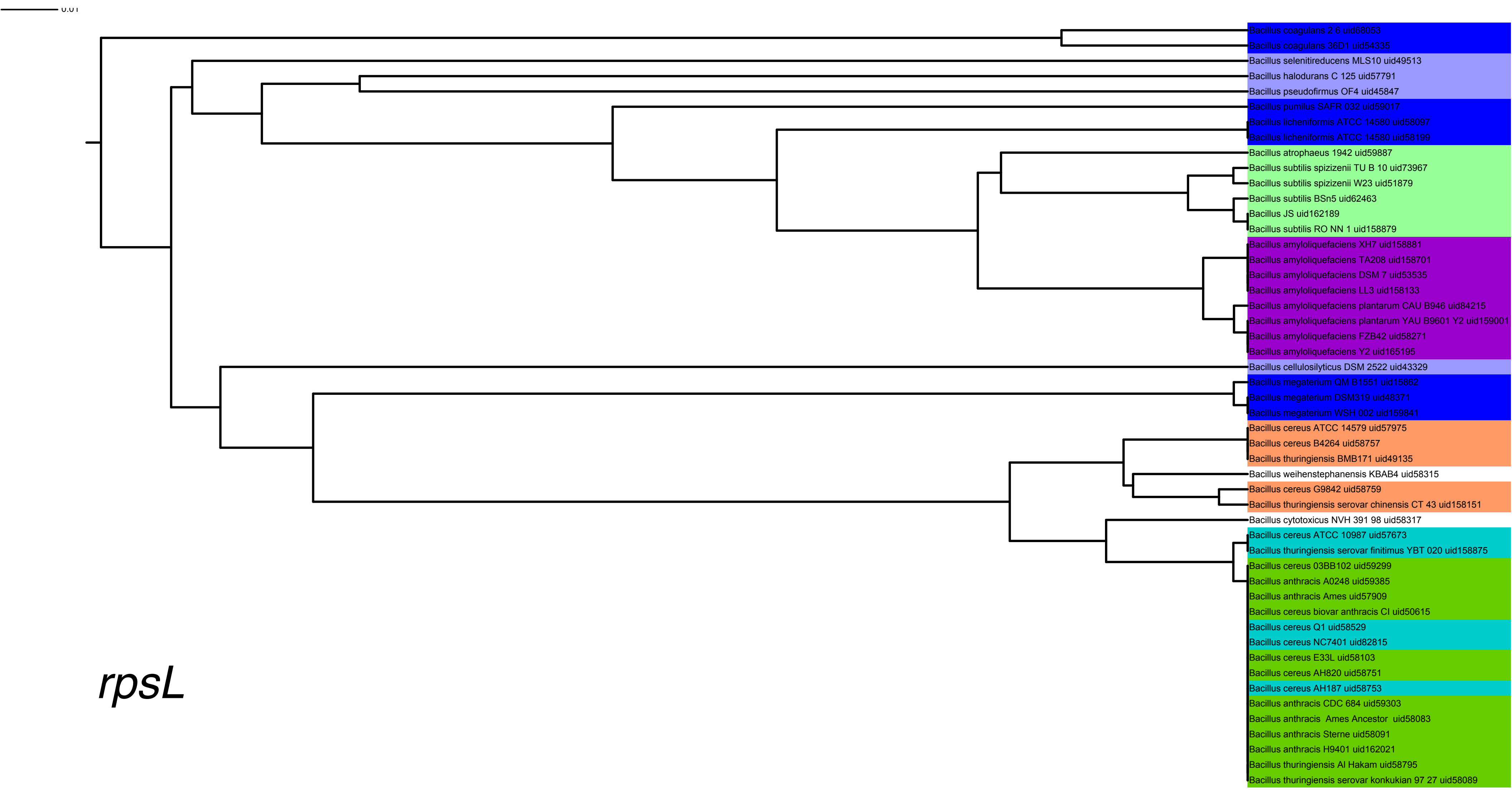
0.01
Bacillus coagulans 2 6 uid68053
Bacillus coagulans 36D1 uid54335
Bacillus selenitireducens MLS10 uid49513
Bacillus halodurans C 125 uid57791
Bacillus pseudofirmus OF4 uid45847
Bacillus pumilus SAFR 032 uid59017
Bacillus licheniformis ATCC 14580 uid58097
Bacillus licheniformis ATCC 14580 uid58199
Bacillus atrophaeus 1942 uid59887
Bacillus subtilis spizizenii TU B 10 uid73967
Bacillus subtilis spizizenii W23 uid51879
Bacillus subtilis BSn5 uid62463
Bacillus JS uid162189
Bacillus subtilis RO NN 1 uid158879
Bacillus amyloliquefaciens XH7 uid158881
Bacillus amyloliquefaciens TA208 uid158701
Bacillus amyloliquefaciens DSM 7 uid53535
Bacillus amyloliquefaciens LL3 uid158133
Bacillus amyloliquefaciens plantarum CAU B946 uid84215
Bacillus amyloliquefaciens plantarum YAU B9601 Y2 uid159001
Bacillus amyloliquefaciens FZB42 uid58271
Bacillus amyloliquefaciens Y2 uid165195
Bacillus cellulosilyticus DSM 2522 uid43329
Bacillus megaterium QM B1551 uid15862
Bacillus megaterium DSM319 uid48371
Bacillus megaterium WSH 002 uid159841
Bacillus cereus ATCC 14579 uid57975
Bacillus cereus B4264 uid58757
Bacillus thuringiensis BMB171 uid49135
Bacillus weihenstephanensis KBAB4 uid58315
Bacillus cereus G9842 uid58759
Bacillus thuringiensis serovar chinensis CT 43 uid158151
Bacillus cytotoxicus NVH 391 98 uid58317
Bacillus cereus ATCC 10987 uid57673
Bacillus thuringiensis serovar finitimus YBT 020 uid158875
Bacillus cereus 03BB102 uid59299
Bacillus anthracis A0248 uid59385
Bacillus anthracis Ames uid57909
Bacillus cereus biovar anthracis CI uid50615
Bacillus cereus Q1 uid58529
Bacillus cereus NC7401 uid82815
Bacillus cereus E33L uid58103
Bacillus cereus AH820 uid58751
Bacillus cereus AH187 uid58753
Bacillus anthracis CDC 684 uid59303
Bacillus anthracis  Ames Ancestor  uid58083
Bacillus anthracis Sterne uid58091
Bacillus anthracis H9401 uid162021
Bacillus thuringiensis Al Hakam uid58795
Bacillus thuringiensis serovar konkukian 97 27 uid58089
rpsL

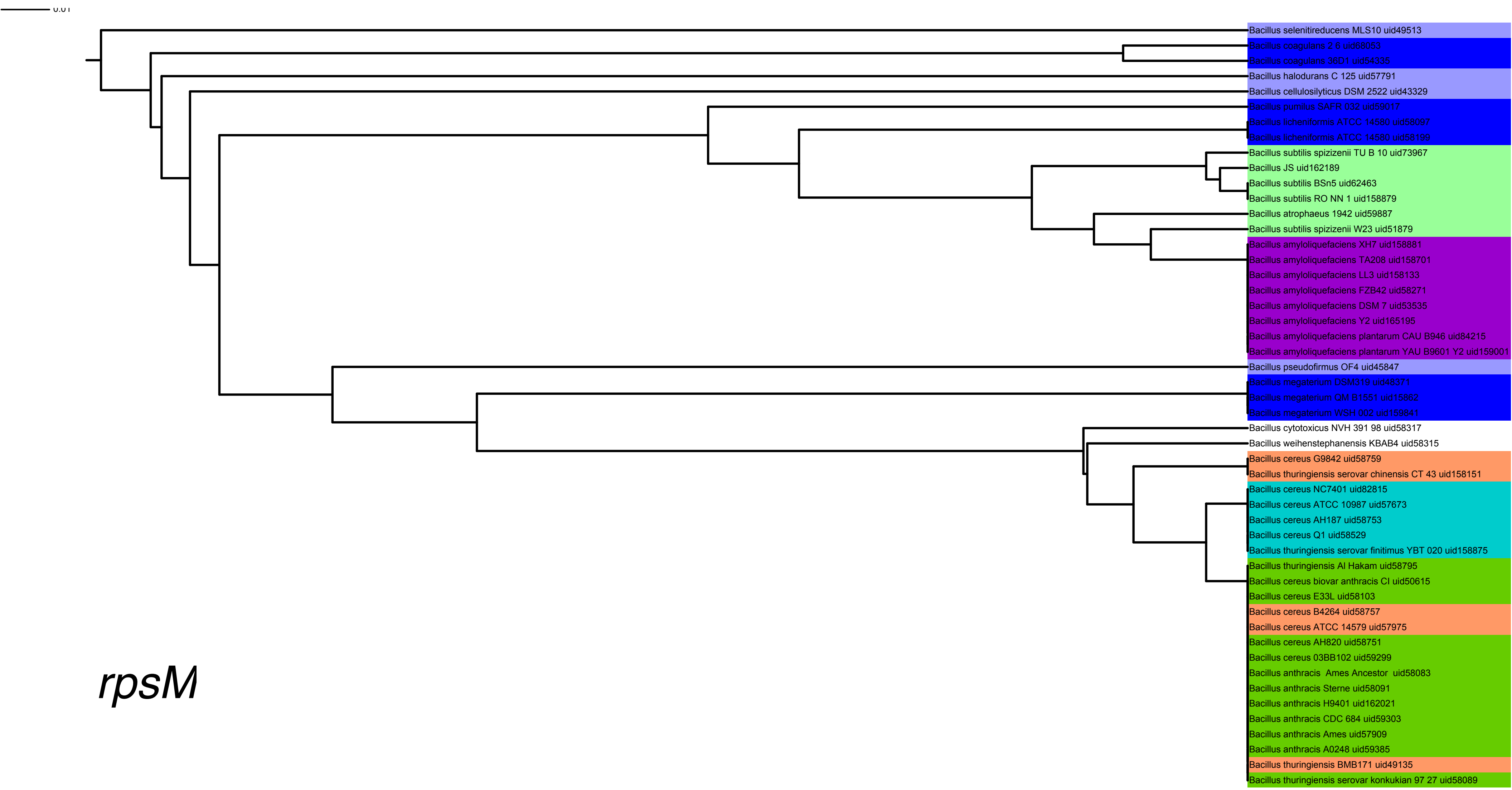
0.01
Bacillus selenitireducens MLS10 uid49513
Bacillus coagulans 2 6 uid68053
Bacillus coagulans 36D1 uid54335
Bacillus halodurans C 125 uid57791
Bacillus cellulosilyticus DSM 2522 uid43329
Bacillus pumilus SAFR 032 uid59017
Bacillus licheniformis ATCC 14580 uid58097
Bacillus licheniformis ATCC 14580 uid55199
Bacillus subtilis spizizenii TU B 10 uid73967
Bacillus JS uid162189
Bacillus subtilis BSn5 uid62463
Bacillus subtilis RO NN 1 uid158879
Bacillus atrophaeus 1942 uid59887
Bacillus subtilis spizizenii W23 uid51879
Bacillus amyloliquefaciens XH7 uid158881
Bacillus amyloliquefaciens TA208 uid158701
Bacillus amyloliquefaciens LL3 uid158133
Bacillus amyloliquefaciens FZB42 uid58271
Bacillus amyloliquefaciens DSM 7 uid53535
Bacillus amyloliquefaciens Y2 uid165195
Bacillus amyloliquefaciens plantarum CAU B946 uid84215
Bacillus amyloliquefaciens plantarum YAU B9601 Y2 uid159001
Bacillus pseudofirmus OF4 uid45847
Bacillus megaterium DSM319 uid48371
Bacillus megaterium QM B1551 uid15882
Bacillus megaterium WSH 002 uid159841
Bacillus cytotoxicus NVH 391 98 uid58317
Bacillus weihenstephanensis KBAB4 uid58315
Bacillus cereus G9842 uid58759
Bacillus thuringiensis serovar chinensis CT 43 uid158151
Bacillus cereus NC7401 uid82815
Bacillus cereus ATCC 10987 uid57673
Bacillus cereus AH187 uid58753
Bacillus cereus Q1 uid58529
Bacillus thuringiensis serovar finitimus YBT 020 uid158875
Bacillus thuringiensis Al Hakam uid58795
Bacillus cereus biovar anthracis CI uid50615
Bacillus cereus E33L uid58103
Bacillus cereus B4264 uid58757
Bacillus cereus ATCC 14579 uid57975
Bacillus cereus AH820 uid58751
Bacillus cereus 03BB102 uid59299
Bacillus anthracis Ames Ancestor uid58083
Bacillus anthracis Sterne uid58091
Bacillus anthracis H9401 uid162021
Bacillus anthracis CDC 684 uid59303
Bacillus anthracis Ames uid57909
Bacillus anthracis A0248 uid59385
Bacillus thuringiensis BMB171 uid49135
Bacillus thuringiensis serovar konkukian 97 27 uid58089
rpsM

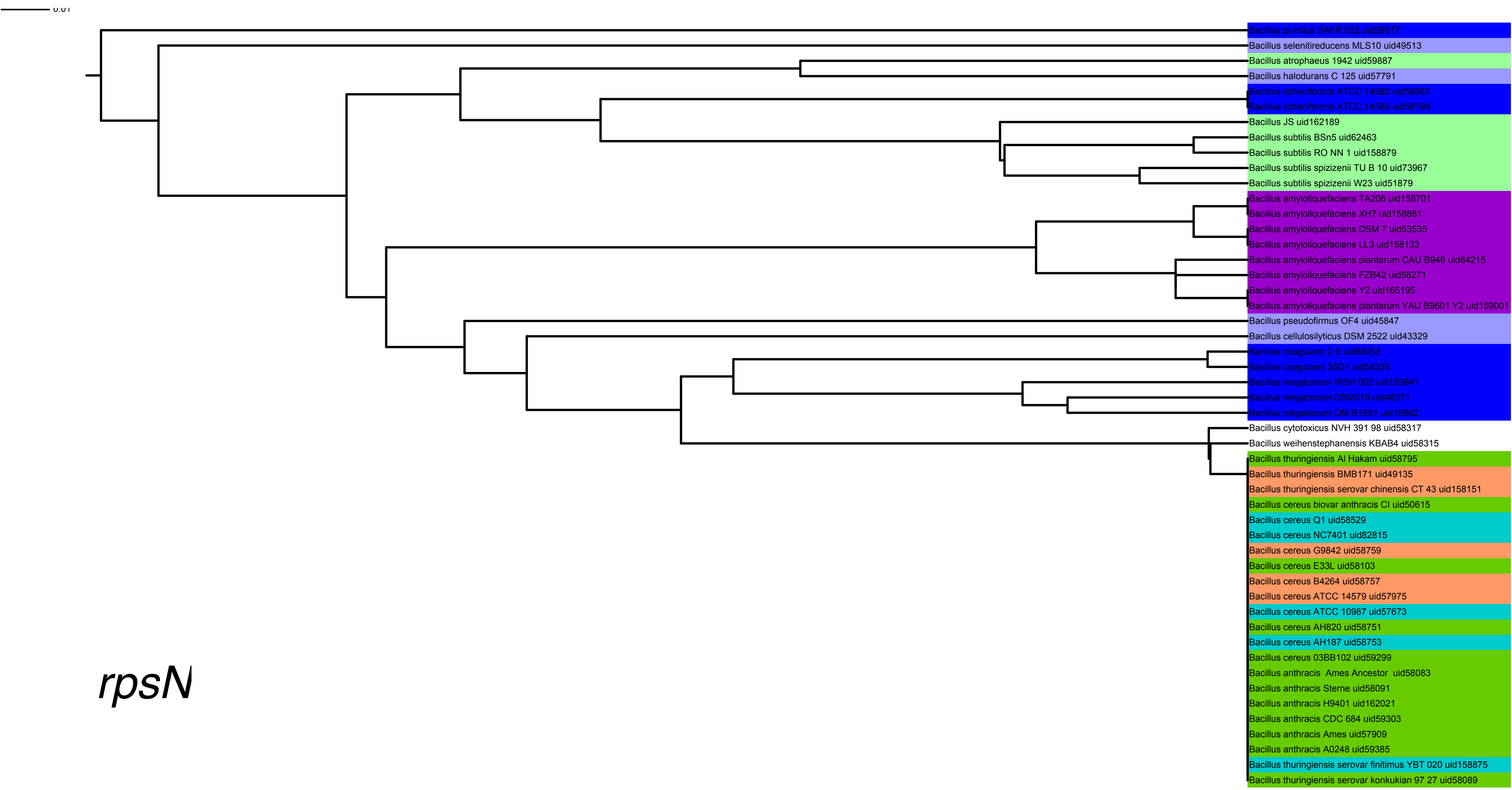
0.01
Bacillus pumilus SAFR 032 uid59017
Bacillus selenitireducens MLS10 uid49513
Bacillus atrophaeus 1942 uid59887
Bacillus halodurans C 125 uid57791
Bacillus licheniformis ATCC 14580 uid58097
Bacillus licheniformis ATCC 14580 uid58199
Bacillus JS uid162189
Bacillus subtilis BSn5 uid62463
Bacillus subtilis RO NN 1 uid158879
Bacillus subtilis spizizenii TU B 10 uid73967
Bacillus subtilis spizizenii W23 uid51879
Bacillus amyloliquefaciens TA208 uid158701
Bacillus amyloliquefaciens XH7 uid158881
Bacillus amyloliquefaciens DSM 7 uid53535
Bacillus amyloliquefaciens LL3 uid158133
Bacillus amyloliquefaciens plantarum CAU B946 uid84215
Bacillus amyloliquefaciens FZB42 uid58271
Bacillus amyloliquefaciens Y2 uid165195
Bacillus amyloliquefaciens plantarum YAU B9601 Y2 uid159001
Bacillus pseudofirmus OF4 uid45847
Bacillus cellulosilyticus DSM 2522 uid43329
Bacillus coagulans 2 6 uid68053
Bacillus coagulans 36D1 uid54335
Bacillus megaterium WSH 002 uid159841
Bacillus megaterium DSM319 uid48371
Bacillus megaterium QM B1551 uid15862
Bacillus cytotoxicus NVH 391 98 uid58317
Bacillus weihenstephanensis KBAB4 uid58315
Bacillus thuringiensis Al Hakam uid58795
Bacillus thuringiensis BMB171 uid49135
Bacillus thuringiensis serovar chinensis CT 43 uid158151
Bacillus cereus biovar anthracis CI uid50615
Bacillus cereus Q1 uid58529
Bacillus cereus NC7401 uid82815
Bacillus cereus G9842 uid58759
Bacillus cereus E33L uid58103
Bacillus cereus B4264 uid58757
Bacillus cereus ATCC 14579 uid57975
Bacillus cereus ATCC 10987 uid57673
Bacillus cereus AH820 uid58751
Bacillus cereus AH187 uid58753
Bacillus cereus 03BB102 uid59299
Bacillus anthracis Ames Ancestor uid58083
Bacillus anthracis Sterne uid58091
Bacillus anthracis H9401 uid162021
Bacillus anthracis CDC 684 uid59303
Bacillus anthracis Ames uid57909
Bacillus anthracis A0248 uid59385
Bacillus thuringiensis serovar finitimus YBT 020 uid158875
Bacillus thuringiensis serovar konkukian 97 27 uid58089
rpsN

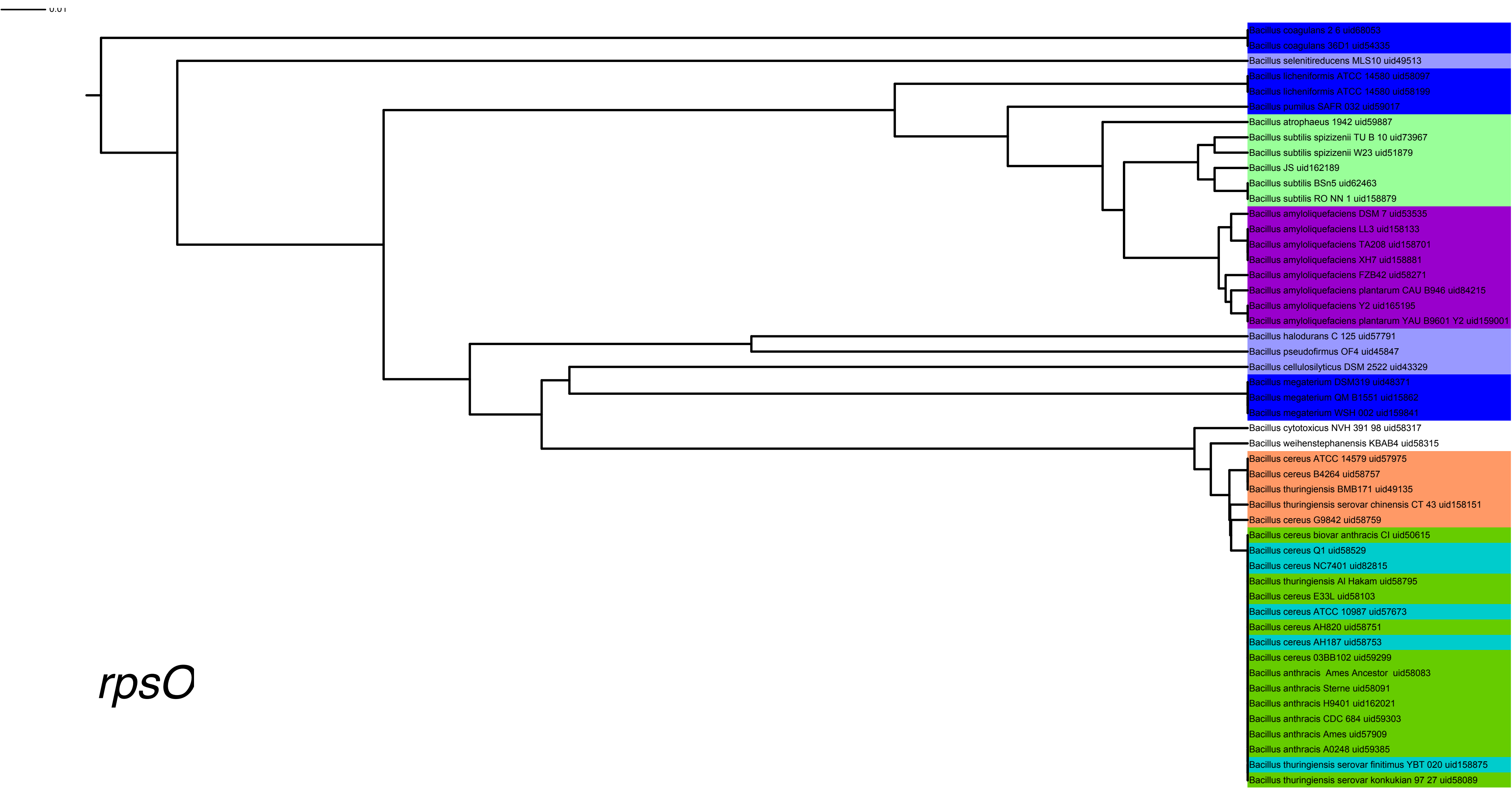
0.01
Bacillus coagulans 2 6 uid68053
Bacillus coagulans 36D1 uid54335
Bacillus selenitireducens MLS10 uid49513
Bacillus licheniformis ATCC 14580 uid58097
Bacillus licheniformis ATCC 14580 uid58199
Bacillus pumilus SAFR 032 uid59017
Bacillus atrophaeus 1942 uid59887
Bacillus subtilis spizizenii TU B 10 uid73967
Bacillus subtilis spizizenii W23 uid51879
Bacillus JS uid162189
Bacillus subtilis BSn5 uid62463
Bacillus subtilis RO NN 1 uid158879
Bacillus amyloliquefaciens DSM 7 uid53535
Bacillus amyloliquefaciens LL3 uid158133
Bacillus amyloliquefaciens TA208 uid158701
Bacillus amyloliquefaciens XH7 uid158881
Bacillus amyloliquefaciens FZB42 uid58271
Bacillus amyloliquefaciens plantarum CAU B946 uid84215
Bacillus amyloliquefaciens Y2 uid165195
Bacillus amyloliquefaciens plantarum YAU B9601 Y2 uid159001
Bacillus halodurans C 125 uid57791
Bacillus pseudofirmus OF4 uid45847
Bacillus cellulosilyticus DSM 2522 uid43329
Bacillus megaterium DSM319 uid48371
Bacillus megaterium QM B1551 uid15862
Bacillus megaterium WSH 002 uid159841
Bacillus cytotoxicus NVH 391 98 uid58317
Bacillus weihenstephanensis KBAB4 uid58315
Bacillus cereus ATCC 14579 uid57975
Bacillus cereus B4264 uid58757
Bacillus thuringiensis BMB171 uid49135
Bacillus thuringiensis serovar chinensis CT 43 uid158151
Bacillus cereus G9842 uid58759
Bacillus cereus biovar anthracis CI uid50615
Bacillus cereus Q1 uid58529
Bacillus cereus NC7401 uid82815
Bacillus thuringiensis Al Hakam uid58795
Bacillus cereus E33L uid58103
Bacillus cereus ATCC 10987 uid57673
Bacillus cereus AH820 uid58751
Bacillus cereus AH187 uid58753
Bacillus cereus 03BB102 uid59299
Bacillus anthracis  Ames Ancestor  uid58083
Bacillus anthracis Sterne uid58091
Bacillus anthracis H9401 uid162021
Bacillus anthracis CDC 684 uid59303
Bacillus anthracis Ames uid57909
Bacillus anthracis A0248 uid59385
Bacillus thuringiensis serovar finitimus YBT 020 uid158875
Bacillus thuringiensis serovar konkukian 97 27 uid58089
rpsO

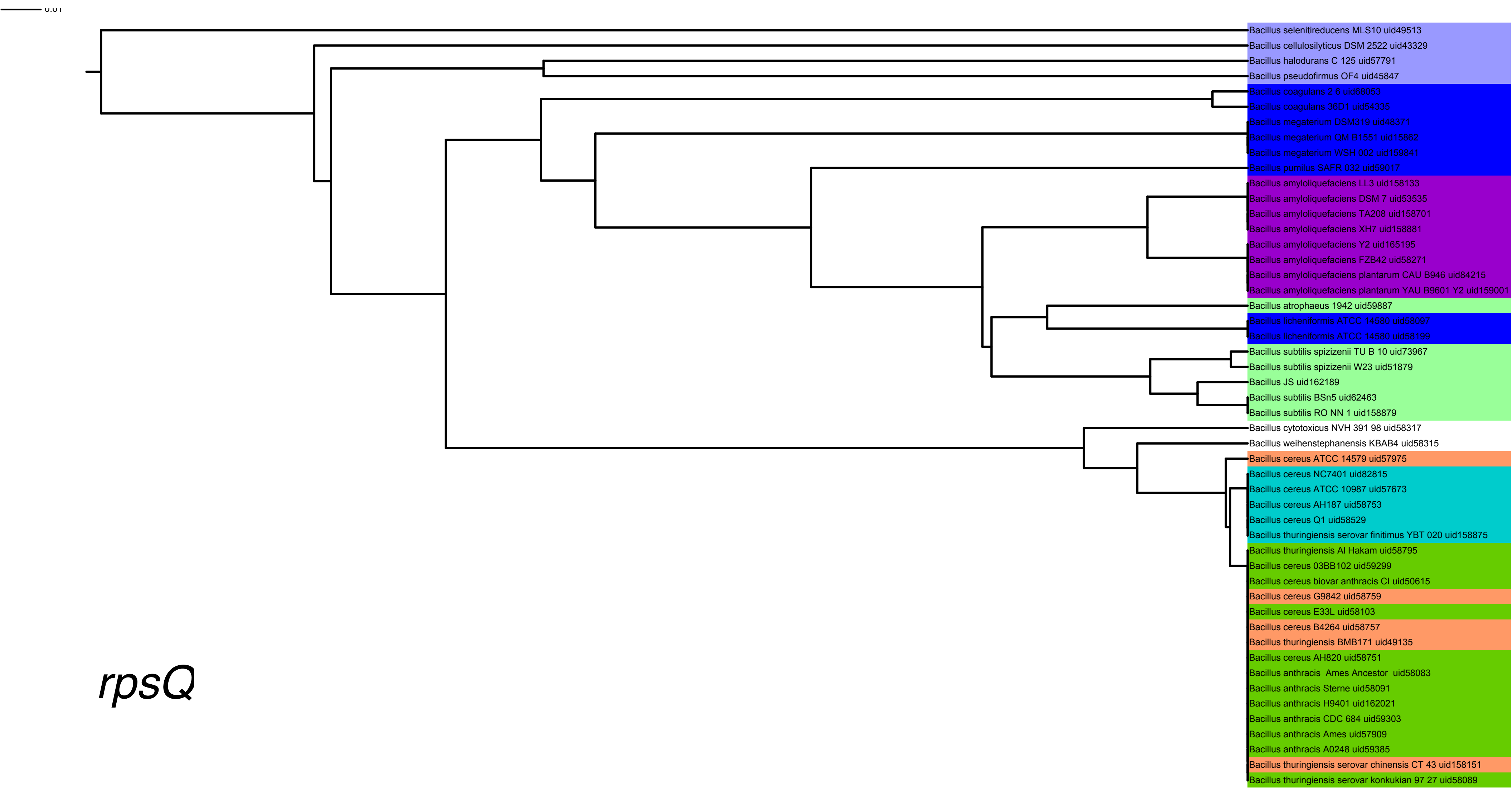
0.01
Bacillus selenitireducens MLS10 uid49513
Bacillus cellulosilyticus DSM 2522 uid43329
Bacillus halodurans C 125 uid57791
Bacillus pseudofirmus OF4 uid45847
Bacillus coagulans 2 6 uid68053
Bacillus coagulans 36D1 uid54335
Bacillus megaterium DSM319 uid48371
Bacillus megaterium QM B1551 uid16862
Bacillus megaterium WSH 002 uid159841
Bacillus pumilus SAFR 032 uid59017
Bacillus amyloliquefaciens LL3 uid158133
Bacillus amyloliquefaciens DSM 7 uid53535
Bacillus amyloliquefaciens TA208 uid158701
Bacillus amyloliquefaciens XH7 uid158881
Bacillus amyloliquefaciens Y2 uid165195
Bacillus amyloliquefaciens FZB42 uid58271
Bacillus amyloliquefaciens plantarum CAU B946 uid84215
Bacillus amyloliquefaciens plantarum YAU B9601 Y2 uid159001
Bacillus atrophaeus 1942 uid59887
Bacillus licheniformis ATCC 14580 uid58097
Bacillus licheniformis ATCC 14580 uid58199
Bacillus subtilis spizizenii TU B 10 uid73967
Bacillus subtilis spizizenii W23 uid51879
Bacillus JS uid162189
Bacillus subtilis BSn5 uid62463
Bacillus subtilis RO NN 1 uid158879
Bacillus cytotoxicus NVH 391 98 uid58317
Bacillus weihenstephanensis KBAB4 uid58315
Bacillus cereus ATCC 14579 uid57975
Bacillus cereus NC7401 uid82815
Bacillus cereus ATCC 10987 uid57673
Bacillus cereus AH187 uid58753
Bacillus cereus Q1 uid58529
Bacillus thuringiensis serovar finitimus YBT 020 uid158875
Bacillus thuringiensis Al Hakam uid58795
Bacillus cereus 03BB102 uid59299
Bacillus cereus biovar anthracis CI uid50615
Bacillus cereus G9842 uid58759
Bacillus cereus E33L uid58103
Bacillus cereus B4264 uid58757
Bacillus thuringiensis BMB171 uid49135
Bacillus cereus AH820 uid58751
Bacillus anthracis Ames Ancestor uid58083
Bacillus anthracis Sterne uid58091
Bacillus anthracis H9401 uid162021
Bacillus anthracis CDC 684 uid59303
Bacillus anthracis Ames uid57909
Bacillus anthracis A0248 uid59385
Bacillus thuringiensis serovar chinensis CT 43 uid158151
Bacillus thuringiensis serovar konkukian 97 27 uid58089
rpsQ

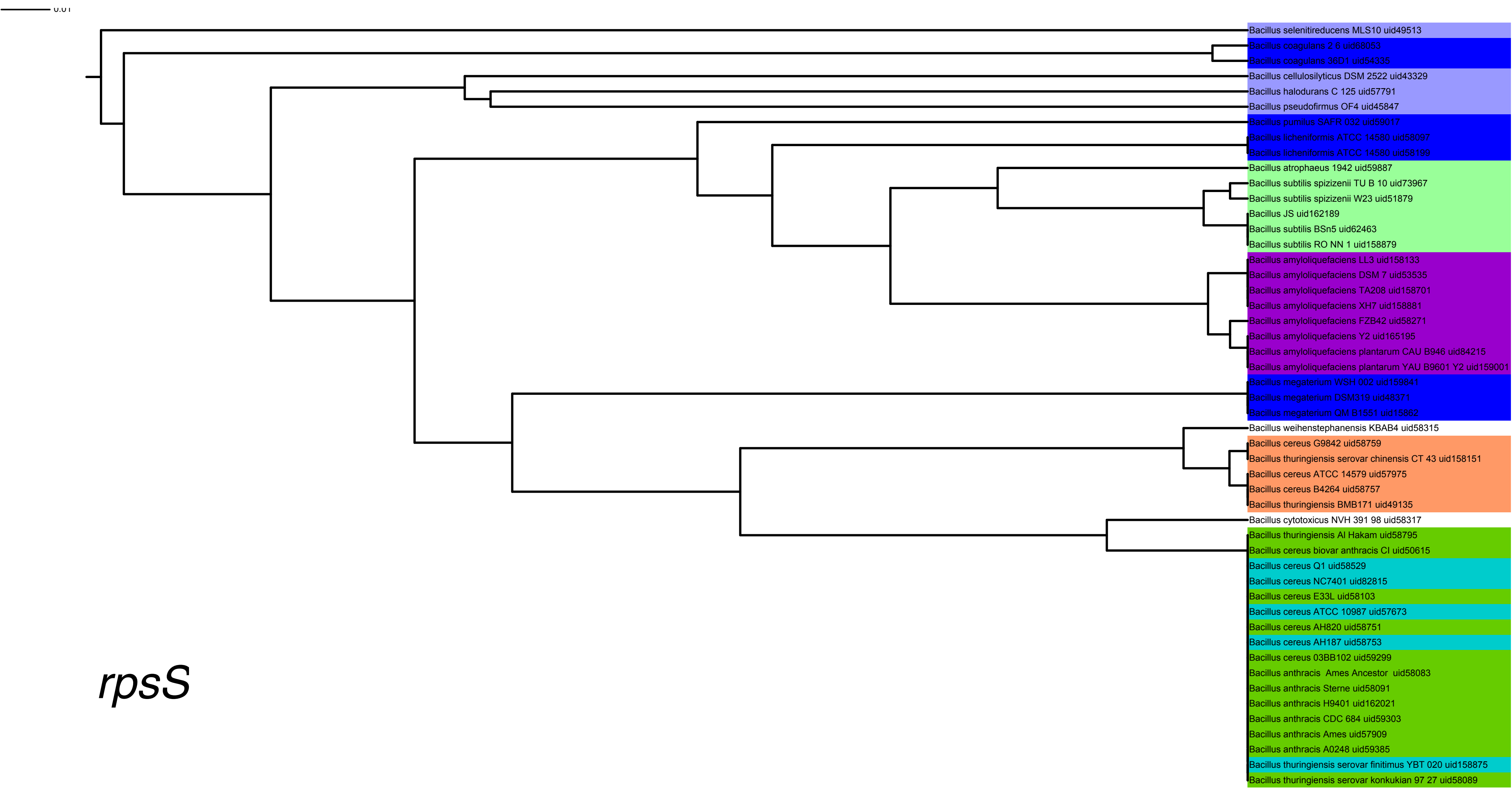
0.01
Bacillus selenitireducens MLS10 uid49513
Bacillus coagulans 2 6 uid68053
Bacillus coagulans 36D1 uid54335
Bacillus cellulosilyticus DSM 2522 uid43329
Bacillus halodurans C 125 uid57791
Bacillus pseudofirmus OF4 uid45847
Bacillus pumilus SAFR 032 uid59017
Bacillus licheniformis ATCC 14580 uid58097
Bacillus licheniformis ATCC 14580 uid58199
Bacillus atrophaeus 1942 uid59887
Bacillus subtilis spizizenii TU B 10 uid73967
Bacillus subtilis spizizenii W23 uid51879
Bacillus JS uid162189
Bacillus subtilis BSn5 uid62463
Bacillus subtilis RO NN 1 uid158879
Bacillus amyloliquefaciens LL3 uid158133
Bacillus amyloliquefaciens DSM 7 uid53535
Bacillus amyloliquefaciens TA208 uid158701
Bacillus amyloliquefaciens XH7 uid158881
Bacillus amyloliquefaciens FZB42 uid58271
Bacillus amyloliquefaciens Y2 uid165195
Bacillus amyloliquefaciens plantarum CAU B946 uid84215
Bacillus amyloliquefaciens plantarum YAU B9601 Y2 uid159001
Bacillus megaterium WSH 002 uid159841
Bacillus megaterium DSM319 uid48371
Bacillus megaterium QM B1551 uid15862
Bacillus weihenstephanensis KBAB4 uid58315
Bacillus cereus G9842 uid58759
Bacillus thuringiensis serovar chinensis CT 43 uid158151
Bacillus cereus ATCC 14579 uid57975
Bacillus cereus B4264 uid58757
Bacillus thuringiensis BMB171 uid49135
Bacillus cytotoxicus NVH 391 98 uid58317
Bacillus thuringiensis Al Hakam uid58795
Bacillus cereus biovar anthracis CI uid50615
Bacillus cereus Q1 uid58529
Bacillus cereus NC7401 uid82815
Bacillus cereus E33L uid58103
Bacillus cereus ATCC 10987 uid57673
Bacillus cereus AH820 uid58751
Bacillus cereus AH187 uid58753
Bacillus cereus 03BB102 uid59299
Bacillus anthracis Ames Ancestor uid58083
Bacillus anthracis Sterne uid58091
Bacillus anthracis H9401 uid162021
Bacillus anthracis CDC 684 uid59303
Bacillus anthracis Ames uid57909
Bacillus anthracis A0248 uid59385
Bacillus thuringiensis serovar finitimus YBT 020 uid158875
Bacillus thuringiensis serovar konkukian 97 27 uid58089
rpsS

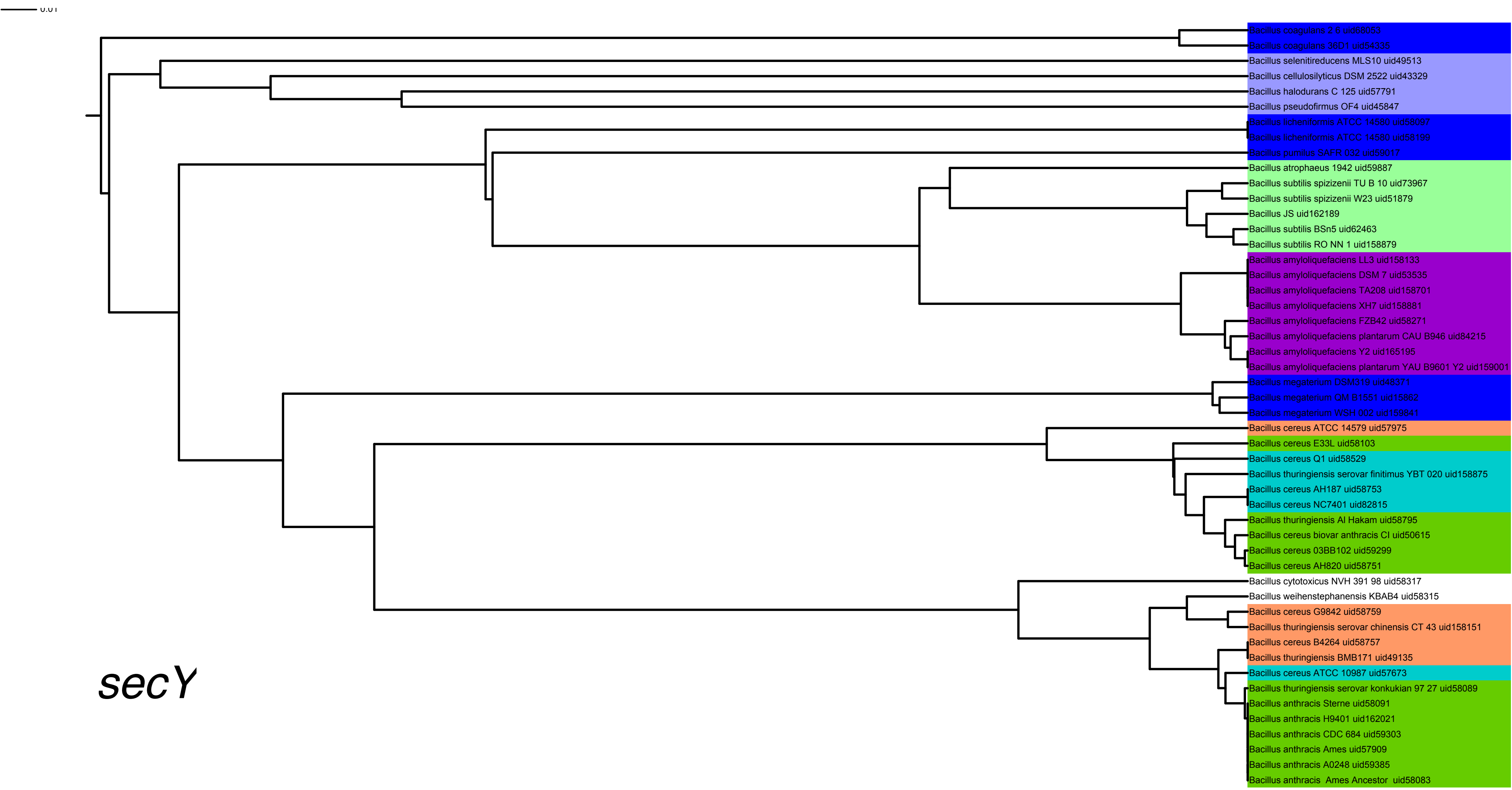
Bacillus coagulans 2 6 uid68053
Bacillus coagulans 36D1 uid54335
Bacillus selenitireducens MLS10 uid49513
Bacillus cellulosilyticus DSM 2522 uid43329
Bacillus halodurans C 125 uid57791
Bacillus pseudofirmus OF4 uid45847
Bacillus licheniformis ATCC 14580 uid58097
Bacillus licheniformis ATCC 14580 uid58199
Bacillus pumilus SAFR 032 uid59017
Bacillus atrophaeus 1942 uid59887
Bacillus subtilis spizizenii TU B 10 uid73967
Bacillus subtilis spizizenii W23 uid51879
Bacillus JS uid162189
Bacillus subtilis BSn5 uid62463
Bacillus subtilis RO NN 1 uid158879
Bacillus amyloliquefaciens LL3 uid158133
Bacillus amyloliquefaciens DSM 7 uid53535
Bacillus amyloliquefaciens TA208 uid158701
Bacillus amyloliquefaciens XH7 uid158881
Bacillus amyloliquefaciens FZB42 uid58271
Bacillus amyloliquefaciens plantarum CAU B946 uid84215
Bacillus amyloliquefaciens Y2 uid165195
Bacillus amyloliquefaciens plantarum YAU B9601 Y2 uid159001
Bacillus megaterium DSM319 uid48371
Bacillus megaterium QM B1551 uid15862
Bacillus megaterium WSH 002 uid159841
Bacillus cereus ATCC 14579 uid57975
Bacillus cereus E33L uid58103
Bacillus cereus Q1 uid58529
Bacillus thuringiensis serovar finitimus YBT 020 uid158875
Bacillus cereus AH187 uid58753
Bacillus cereus NC7401 uid82815
Bacillus thuringiensis Al Hakam uid58795
Bacillus cereus biovar anthracis CI uid50615
Bacillus cereus 03BB102 uid59299
Bacillus cereus AH820 uid58751
Bacillus cytotoxicus NVH 391 98 uid58317
Bacillus weihenstephanensis KBAB4 uid58315
Bacillus cereus G9842 uid58759
Bacillus thuringiensis serovar chinensis CT 43 uid158151
Bacillus cereus B4264 uid58757
Bacillus thuringiensis BMB171 uid49135
Bacillus cereus ATCC 10987 uid57673
Bacillus thuringiensis serovar konkukian 97 27 uid58089
Bacillus anthracis Sterne uid58091
Bacillus anthracis H9401 uid162021
Bacillus anthracis CDC 684 uid59303
Bacillus anthracis Ames uid57909
Bacillus anthracis A0248 uid59385
Bacillus anthracis Ames Ancestor uid58083
secY

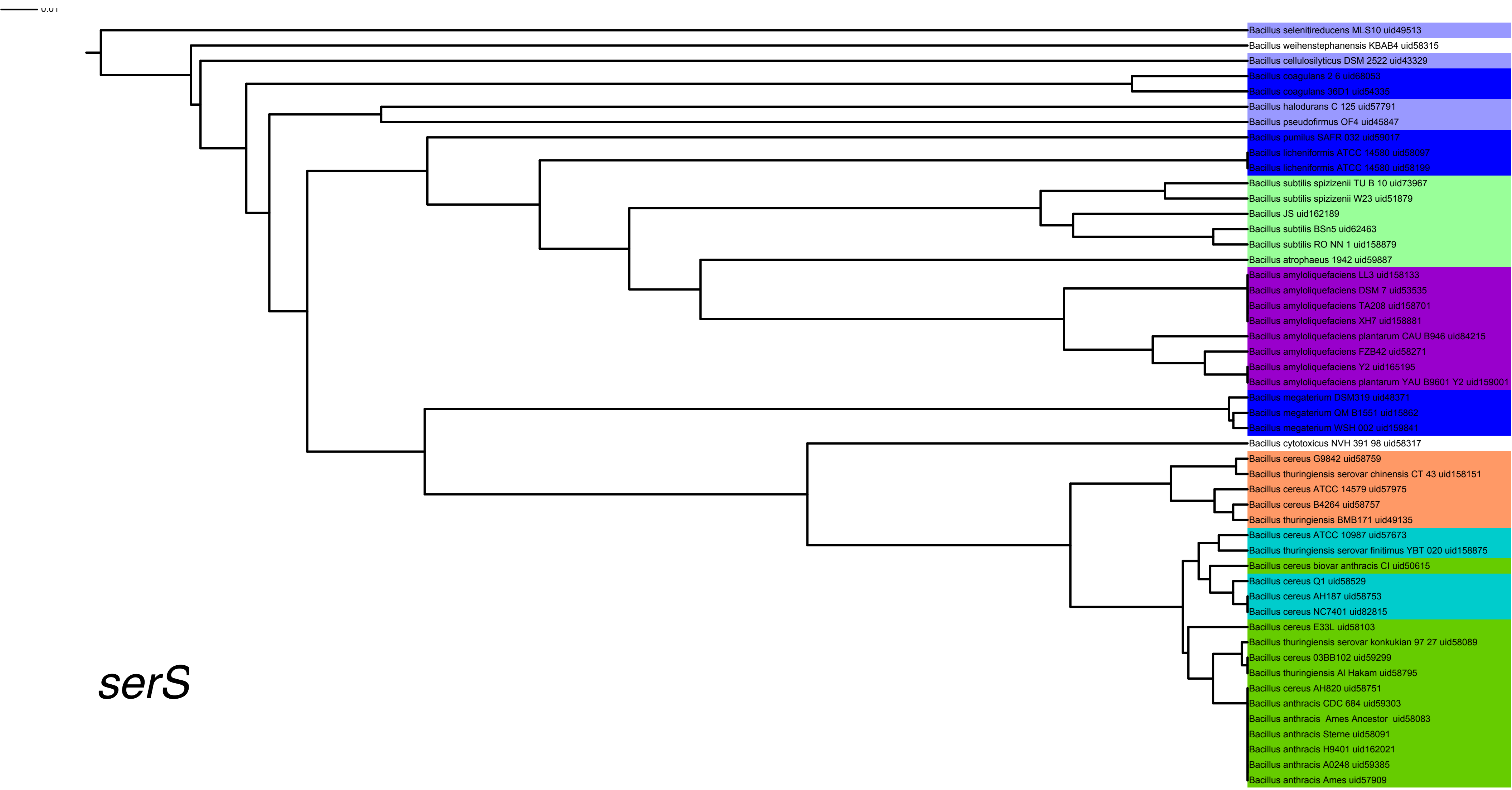
Bacillus selenitireducens MLS10 uid49513
Bacillus weihenstephanensis KBAB4 uid58315
Bacillus cellulosilyticus DSM 2522 uid43329
Bacillus coagulans 2 6 uid68053
Bacillus coagulans 36D1 uid54335
Bacillus halodurans C 125 uid57791
Bacillus pseudofirmus OF4 uid45847
Bacillus pumilus SAFR 032 uid59017
Bacillus licheniformis ATCC 14580 uid58097
Bacillus licheniformis ATCC 14580 uid58199
Bacillus subtilis spizizenii TU B 10 uid73967
Bacillus subtilis spizizenii W23 uid51879
Bacillus JS uid162189
Bacillus subtilis BSn5 uid62463
Bacillus subtilis RO NN 1 uid158879
Bacillus atrophaeus 1942 uid59887
Bacillus amyloliquefaciens LL3 uid158133
Bacillus amyloliquefaciens DSM 7 uid53535
Bacillus amyloliquefaciens TA208 uid158701
Bacillus amyloliquefaciens XH7 uid158881
Bacillus amyloliquefaciens plantarum CAU B946 uid84215
Bacillus amyloliquefaciens FZB42 uid58271
Bacillus amyloliquefaciens Y2 uid165195
Bacillus amyloliquefaciens plantarum YAU B9601 Y2 uid159001
Bacillus megaterium DSM319 uid48371
Bacillus megaterium QM B1551 uid15862
Bacillus megaterium WSH 002 uid159841
Bacillus cytotoxicus NVH 391 98 uid58317
Bacillus cereus G9842 uid58759
Bacillus thuringiensis serovar chinensis CT 43 uid158151
Bacillus cereus ATCC 14579 uid57975
Bacillus cereus B4264 uid58757
Bacillus thuringiensis BMB171 uid49135
Bacillus cereus ATCC 10987 uid57673
Bacillus thuringiensis serovar finitimus YBT 020 uid158875
Bacillus cereus biovar anthracis CI uid50615
Bacillus cereus Q1 uid58529
Bacillus cereus AH187 uid58753
Bacillus cereus NC7401 uid82815
Bacillus cereus E33L uid58103
Bacillus thuringiensis serovar konkukian 97 27 uid58089
Bacillus cereus 03BB102 uid59299
Bacillus thuringiensis Al Hakam uid58795
Bacillus cereus AH820 uid58751
Bacillus anthracis CDC 684 uid59303
Bacillus anthracis  Ames Ancestor  uid58083
Bacillus anthracis Sterne uid58091
Bacillus anthracis H9401 uid162021
Bacillus anthracis A0248 uid59385
Bacillus anthracis Ames uid57909
serS

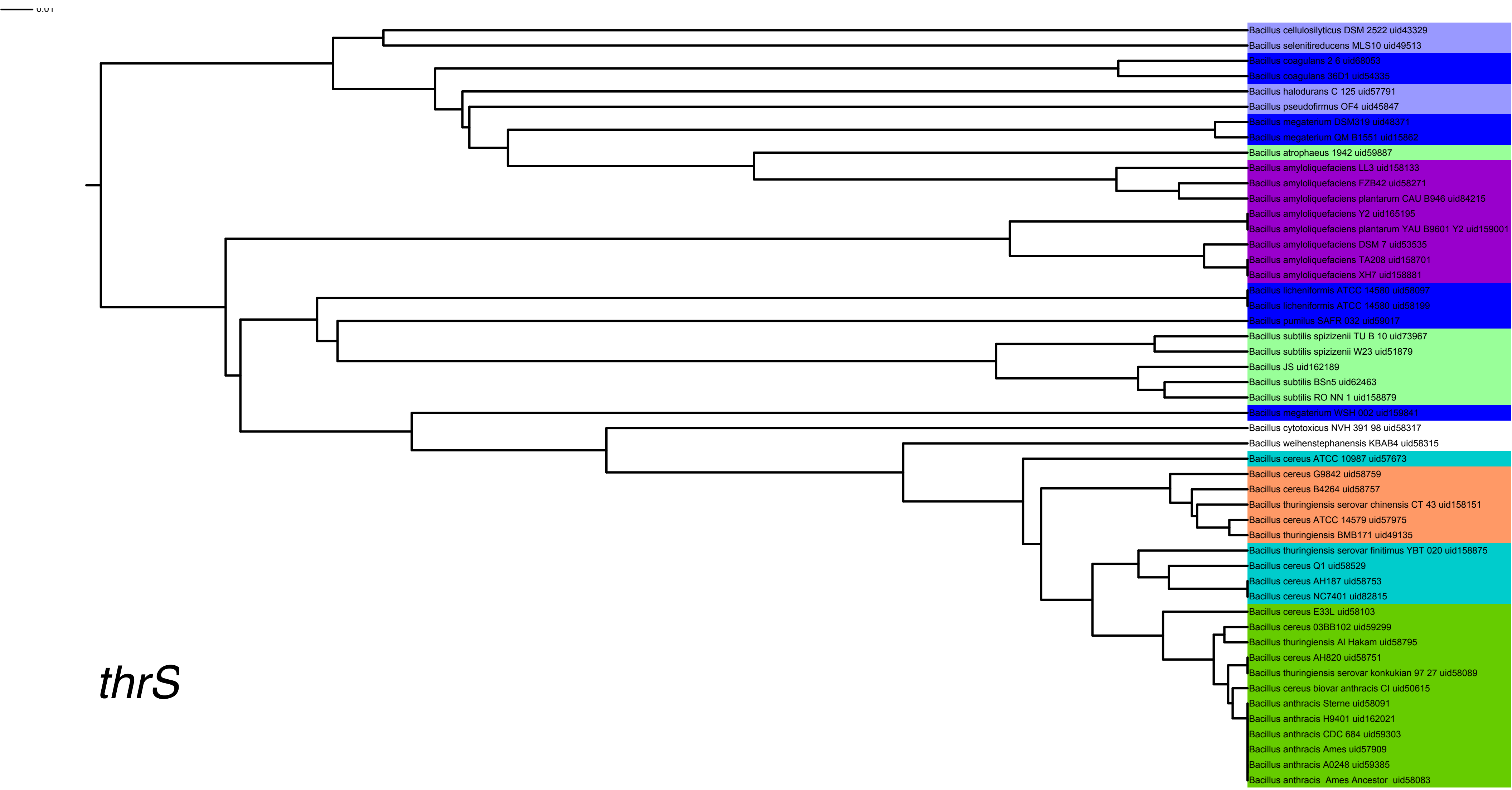
Bacillus cellulosilyticus DSM 2522 uid43329
Bacillus selenitireducens MLS10 uid49513
Bacillus coagulans 2 6 uid68053
Bacillus coagulans 36D1 uid54335
Bacillus halodurans C 125 uid57791
Bacillus pseudofirmus OF4 uid45847
Bacillus megaterium DSM319 uid48371
Bacillus megaterium QM B1551 uid15862
Bacillus atrophaeus 1942 uid59887
Bacillus amyloliquefaciens LL3 uid158133
Bacillus amyloliquefaciens FZB42 uid58271
Bacillus amyloliquefaciens plantarum CAU B946 uid84215
Bacillus amyloliquefaciens Y2 uid165195
Bacillus amyloliquefaciens plantarum YAU B9601 Y2 uid159001
Bacillus amyloliquefaciens DSM 7 uid53535
Bacillus amyloliquefaciens TA208 uid158701
Bacillus amyloliquefaciens XH7 uid158881
Bacillus licheniformis ATCC 14580 uid58097
Bacillus licheniformis ATCC 14580 uid58199
Bacillus pumilus SAFR 032 uid59017
Bacillus subtilis spizizenii TU B 10 uid73967
Bacillus subtilis spizizenii W23 uid51879
Bacillus JS uid162189
Bacillus subtilis BSn5 uid62463
Bacillus subtilis RO NN 1 uid158879
Bacillus megaterium WSH 002 uid159841
Bacillus cytotoxicus NVH 391 98 uid58317
Bacillus weihenstephanensis KBAB4 uid58315
Bacillus cereus ATCC 10987 uid57673
Bacillus cereus G9842 uid58759
Bacillus cereus B4264 uid58757
Bacillus thuringiensis serovar chinensis CT 43 uid158151
Bacillus cereus ATCC 14579 uid57975
Bacillus thuringiensis BMB171 uid49135
Bacillus thuringiensis serovar finitimus YBT 020 uid158875
Bacillus cereus Q1 uid58529
Bacillus cereus AH187 uid58753
Bacillus cereus NC7401 uid82815
Bacillus cereus E33L uid58103
Bacillus cereus 03BB102 uid59299
Bacillus thuringiensis Al Hakam uid58795
Bacillus cereus AH820 uid58751
Bacillus thuringiensis serovar konkukian 97 27 uid58089
Bacillus cereus biovar anthracis CI uid50615
Bacillus anthracis Sterne uid58091
Bacillus anthracis H9401 uid162021
Bacillus anthracis CDC 684 uid59303
Bacillus anthracis Ames uid57909
Bacillus anthracis A0248 uid59385
Bacillus anthracis  Ames Ancestor  uid58083
thrS

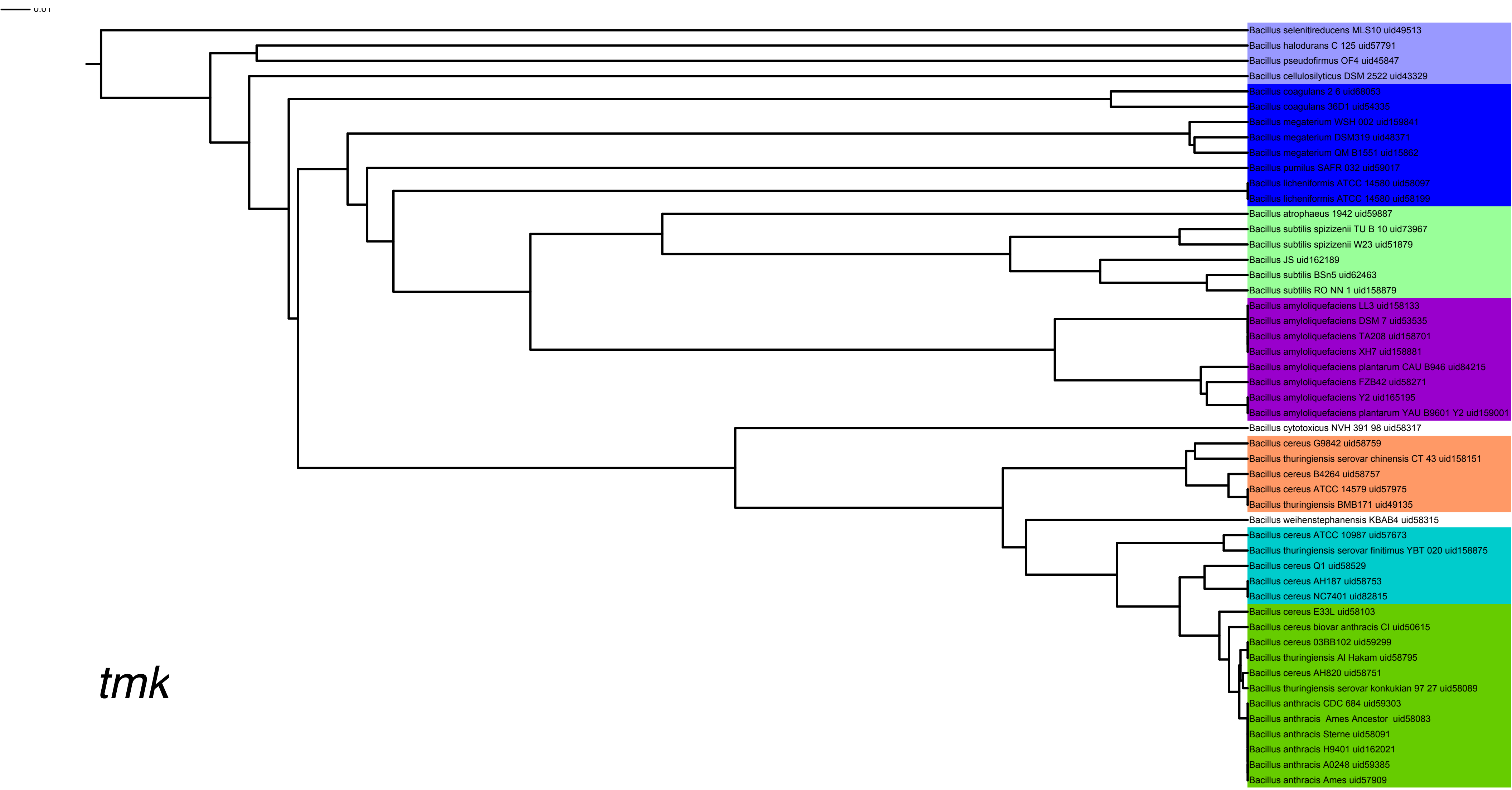

0.01
Bacillus selenitireducens MLS10 uid49513
Bacillus halodurans C 125 uid57791
Bacillus pseudofirmus OF4 uid45847
Bacillus cellulosilyticus DSM 2522 uid43329
Bacillus coagulans 2 6 uid68053
Bacillus coagulans 36D1 uid54335
Bacillus megaterium WSH 002 uid159841
Bacillus megaterium DSM319 uid48371
Bacillus megaterium QM B1551 uid15862
Bacillus pumilus SAFR 032 uid59017
Bacillus licheniformis ATCC 14580 uid58097
Bacillus licheniformis ATCC 14580 uid58199
Bacillus atrophaeus 1942 uid59887
Bacillus subtilis spizizenii TU B 10 uid73967
Bacillus subtilis spizizenii W23 uid51879
Bacillus JS uid162189
Bacillus subtilis BSn5 uid62463
Bacillus subtilis RO NN 1 uid158879
Bacillus amyloliquefaciens LL3 uid158133
Bacillus amyloliquefaciens DSM 7 uid53535
Bacillus amyloliquefaciens TA208 uid158701
Bacillus amyloliquefaciens XH7 uid158881
Bacillus amyloliquefaciens plantarum CAU B946 uid84215
Bacillus amyloliquefaciens FZB42 uid58271
Bacillus amyloliquefaciens Y2 uid165195
Bacillus amyloliquefaciens plantarum YAU B9601 Y2 uid159001
Bacillus cytotoxicus NVH 391 98 uid58317
Bacillus cereus G9842 uid58759
Bacillus thuringiensis serovar chinensis CT 43 uid158151
Bacillus cereus B4264 uid58757
Bacillus cereus ATCC 14579 uid57975
Bacillus thuringiensis BMB171 uid49135
Bacillus weihenstephanensis KBAB4 uid58315
Bacillus cereus ATCC 10987 uid57673
Bacillus thuringiensis serovar finitimus YBT 020 uid158875
Bacillus cereus Q1 uid58529
Bacillus cereus AH187 uid58753
Bacillus cereus NC7401 uid82815
Bacillus cereus E33L uid58103
Bacillus cereus biovar anthracis CI uid50615
Bacillus cereus 03BB102 uid59299
Bacillus thuringiensis Al Hakam uid58795
Bacillus cereus AH820 uid58751
Bacillus thuringiensis serovar konkukian 97 27 uid58089
Bacillus anthracis CDC 684 uid59303
Bacillus anthracis Ames Ancestor uid58083
Bacillus anthracis Sterne uid58091
Bacillus anthracis H9401 uid162021
Bacillus anthracis A0248 uid59385
Bacillus anthracis Ames uid57909
tmk

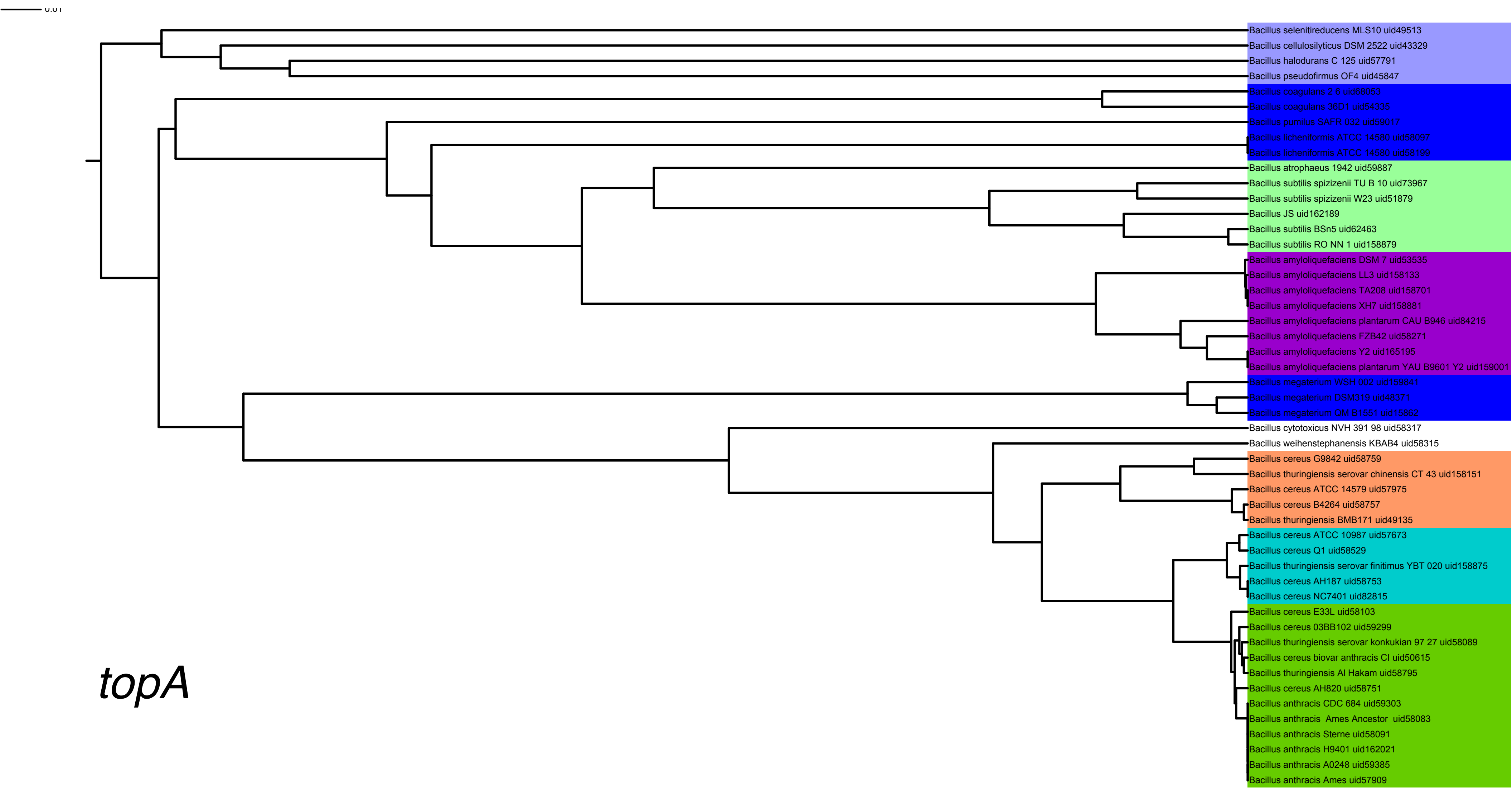
0.01
Bacillus selenitireducens MLS10 uid49513
Bacillus cellulosilyticus DSM 2522 uid43329
Bacillus halodurans C 125 uid57791
Bacillus pseudofirmus OF4 uid45847
Bacillus coagulans 2 6 uid68053
Bacillus coagulans 36D1 uid54335
Bacillus pumilus SAFR 032 uid59017
Bacillus licheniformis ATCC 14580 uid58097
Bacillus licheniformis ATCC 14580 uid58199
Bacillus atrophaeus 1942 uid59887
Bacillus subtilis spizizenii TU B 10 uid73967
Bacillus subtilis spizizenii W23 uid51879
Bacillus JS uid162189
Bacillus subtilis BSn5 uid62463
Bacillus subtilis RO NN 1 uid158879
Bacillus amyloliquefaciens DSM 7 uid53535
Bacillus amyloliquefaciens LL3 uid158133
Bacillus amyloliquefaciens TA208 uid158701
Bacillus amyloliquefaciens XH7 uid158881
Bacillus amyloliquefaciens plantarum CAU B946 uid84215
Bacillus amyloliquefaciens FZB42 uid58271
Bacillus amyloliquefaciens Y2 uid165195
Bacillus amyloliquefaciens plantarum YAU B9601 Y2 uid159001
Bacillus megaterium WSH 002 uid159841
Bacillus megaterium DSM319 uid48371
Bacillus megaterium QM B1551 uid15862
Bacillus cytotoxicus NVH 391 98 uid58317
Bacillus weihenstephanensis KBAB4 uid58315
Bacillus cereus G9842 uid58759
Bacillus thuringiensis serovar chinensis CT 43 uid158151
Bacillus cereus ATCC 14579 uid57975
Bacillus cereus B4264 uid58757
Bacillus thuringiensis BMB171 uid49135
Bacillus cereus ATCC 10987 uid57673
Bacillus cereus Q1 uid58529
Bacillus thuringiensis serovar finitimus YBT 020 uid158875
Bacillus cereus AH187 uid58753
Bacillus cereus NC7401 uid82815
Bacillus cereus E33L uid58103
Bacillus cereus 03BB102 uid59299
Bacillus thuringiensis serovar konkukian 97 27 uid58089
Bacillus cereus biovar anthracis CI uid50615
Bacillus thuringiensis Al Hakam uid58795
Bacillus cereus AH820 uid58751
Bacillus anthracis CDC 684 uid59303
Bacillus anthracis Ames Ancestor uid58083
Bacillus anthracis Sterne uid58091
Bacillus anthracis H9401 uid162021
Bacillus anthracis A0248 uid59385
Bacillus anthracis Ames uid57909
topA

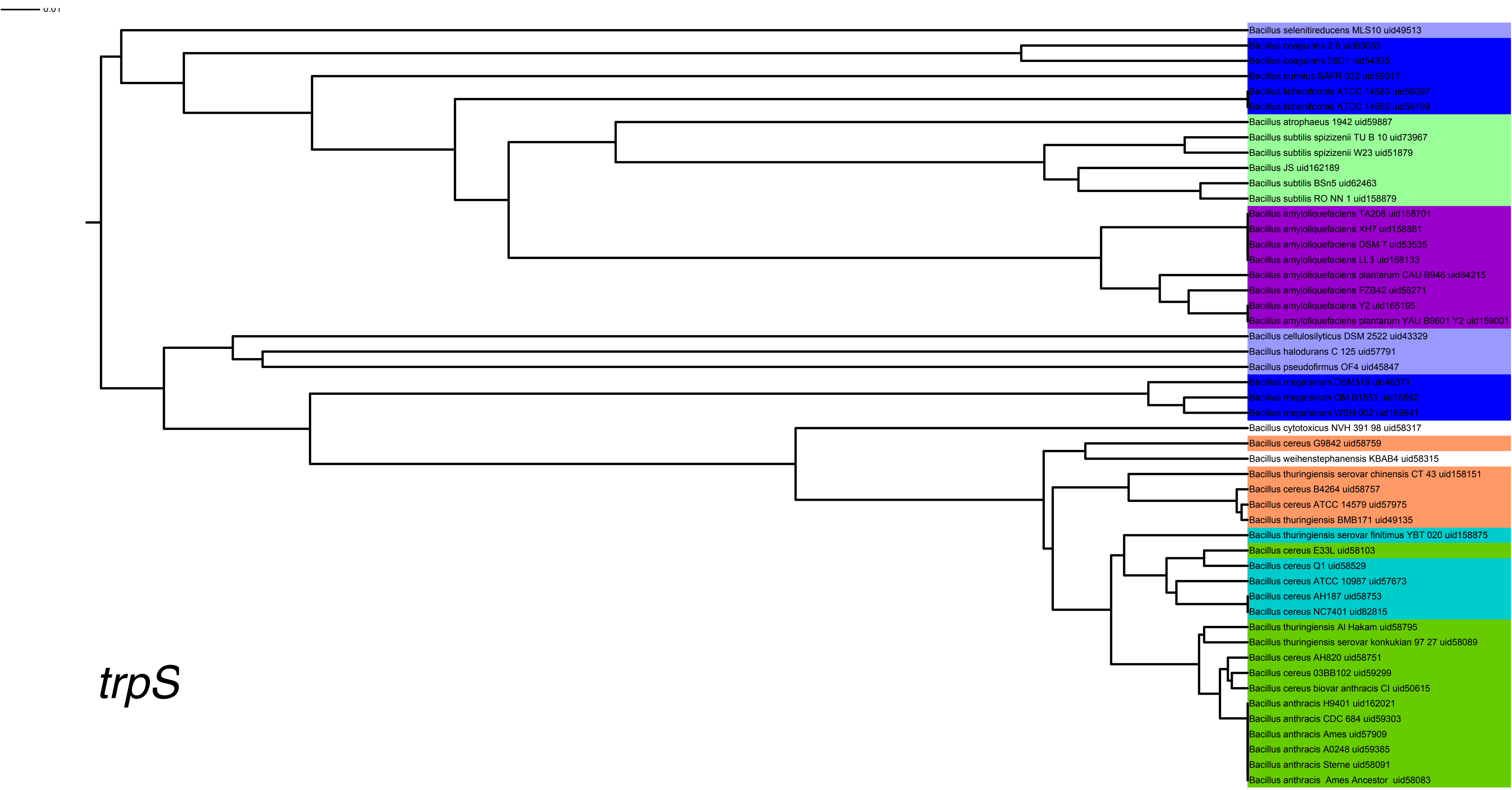
0.01
Bacillus selenitireducens MLS10 uid49513
Bacillus coagulans 2 6 uid68053
Bacillus coagulans 36D1 uid54335
Bacillus pumilus SAFR 032 uid59017
Bacillus licheniformis ATCC 14580 uid58097
Bacillus licheniformis ATCC 14580 uid58199
Bacillus atrophaeus 1942 uid59887
Bacillus subtilis spizizenii TU B 10 uid73967
Bacillus subtilis spizizenii W23 uid51879
Bacillus JS uid162189
Bacillus subtilis BSn5 uid62463
Bacillus subtilis RO NN 1 uid158879
Bacillus amyloliquefaciens TA208 uid158701
Bacillus amyloliquefaciens XH7 uid158881
Bacillus amyloliquefaciens DSM 7 uid53535
Bacillus amyloliquefaciens LL3 uid158133
Bacillus amyloliquefaciens plantarum CAU B946 uid84215
Bacillus amyloliquefaciens FZB42 uid58271
Bacillus amyloliquefaciens Y2 uid165195
Bacillus amyloliquefaciens plantarum YAU B9601 Y2 uid159001
Bacillus cellulosilyticus DSM 2522 uid43329
Bacillus halodurans C 125 uid57791
Bacillus pseudofirmus OF4 uid45847
Bacillus megaterium DSM319 uid48371
Bacillus megaterium QM B1551 uid15862
Bacillus megaterium WSH 002 uid159841
Bacillus cytotoxicus NVH 391 98 uid58317
Bacillus cereus G9842 uid58759
Bacillus weihenstephanensis KBAB4 uid58315
Bacillus thuringiensis serovar chinensis CT 43 uid158151
Bacillus cereus B4264 uid58757
Bacillus cereus ATCC 14579 uid57975
Bacillus thuringiensis BMB171 uid49135
Bacillus thuringiensis serovar finitimus YBT 020 uid158875
Bacillus cereus E33L uid58103
Bacillus cereus Q1 uid58529
Bacillus cereus ATCC 10987 uid57673
Bacillus cereus AH187 uid58753
Bacillus cereus NC7401 uid82815
Bacillus thuringiensis Al Hakam uid58795
Bacillus thuringiensis serovar konkukian 97 27 uid58089
Bacillus cereus AH820 uid58751
Bacillus cereus 03BB102 uid59299
Bacillus cereus biovar anthracis CI uid50615
Bacillus anthracis H9401 uid162021
Bacillus anthracis CDC 684 uid59303
Bacillus anthracis Ames uid57909
Bacillus anthracis A0248 uid59385
Bacillus anthracis Sterne uid58091
Bacillus anthracis  Ames Ancestor  uid58083
trpS

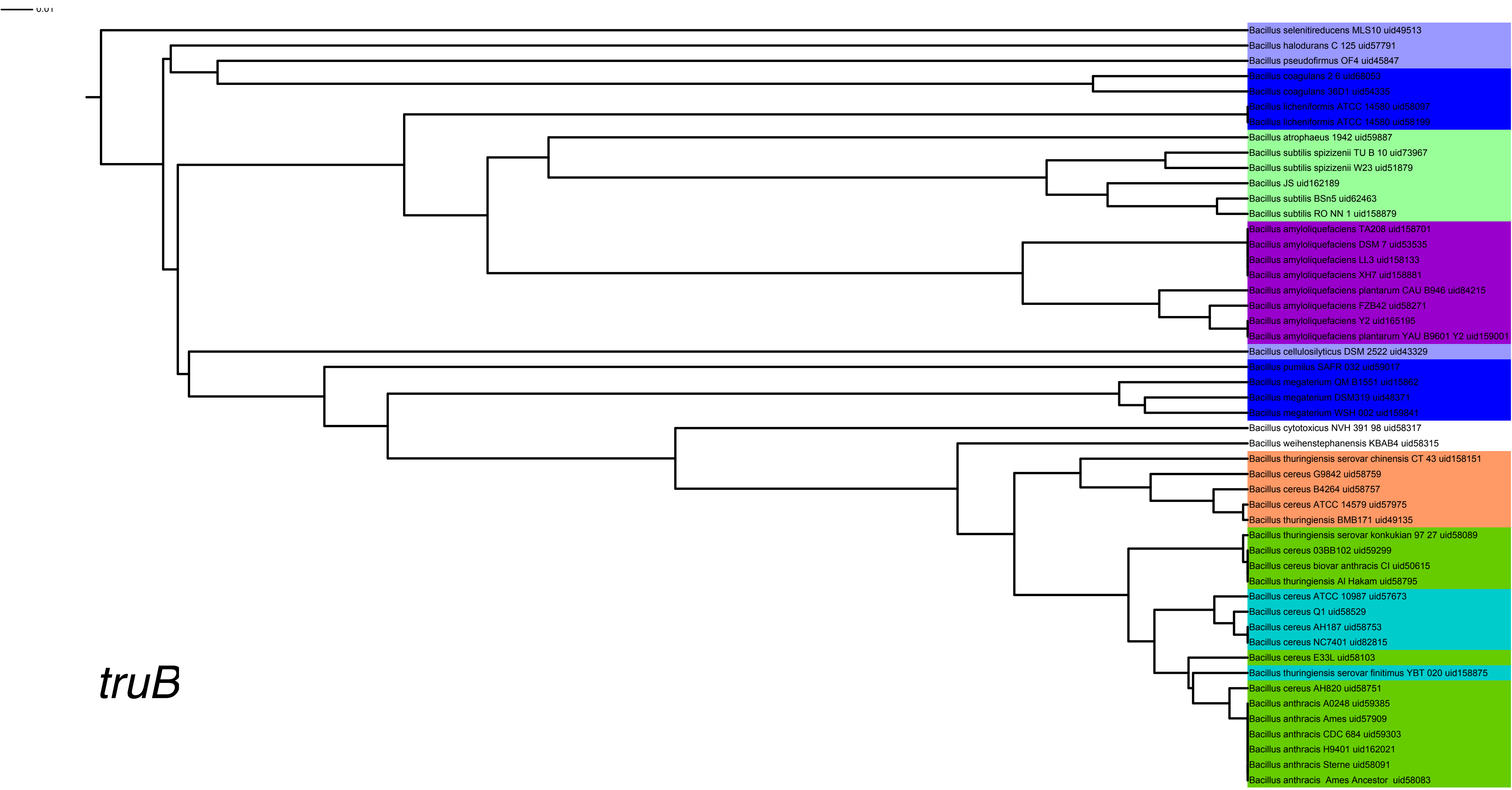
0.01
Bacillus selenitireducens MLS10 uid49513
Bacillus halodurans C 125 uid57791
Bacillus pseudofirmus OF4 uid45847
Bacillus coagulans 2 6 uid68053
Bacillus coagulans 36D1 uid54335
Bacillus licheniformis ATCC 14580 uid58097
Bacillus licheniformis ATCC 14580 uid58199
Bacillus atrophaeus 1942 uid59887
Bacillus subtilis spizizenii TU B 10 uid73967
Bacillus subtilis spizizenii W23 uid51879
Bacillus JS uid162189
Bacillus subtilis BSn5 uid62463
Bacillus subtilis RO NN 1 uid158879
Bacillus amyloliquefaciens TA208 uid158701
Bacillus amyloliquefaciens DSM 7 uid53535
Bacillus amyloliquefaciens LL3 uid158133
Bacillus amyloliquefaciens XH7 uid158881
Bacillus amyloliquefaciens plantarum CAU B946 uid84215
Bacillus amyloliquefaciens FZB42 uid58271
Bacillus amyloliquefaciens Y2 uid165195
Bacillus amyloliquefaciens plantarum YAU B9601 Y2 uid159001
Bacillus cellulosilyticus DSM 2522 uid43329
Bacillus pumilus SAFR 032 uid59017
Bacillus megaterium QM B1551 uid15862
Bacillus megaterium DSM319 uid48371
Bacillus megaterium WSH 002 uid159841
Bacillus cytotoxicus NVH 391 98 uid58317
Bacillus weihenstephanensis KBAB4 uid58315
Bacillus thuringiensis serovar chinensis CT 43 uid158151
Bacillus cereus G9842 uid58759
Bacillus cereus B4264 uid58757
Bacillus cereus ATCC 14579 uid57975
Bacillus thuringiensis BMB171 uid49135
Bacillus thuringiensis serovar konkukian 97 27 uid58089
Bacillus cereus 03BB102 uid59299
Bacillus cereus biovar anthracis CI uid50615
Bacillus thuringiensis Al Hakam uid58795
Bacillus cereus ATCC 10987 uid57673
Bacillus cereus Q1 uid58529
Bacillus cereus AH187 uid58753
Bacillus cereus NC7401 uid82815
Bacillus cereus E33L uid58103
Bacillus thuringiensis serovar finitimus YBT 020 uid158875
Bacillus cereus AH820 uid58751
Bacillus anthracis A0248 uid59385
Bacillus anthracis Ames uid57909
Bacillus anthracis CDC 684 uid59303
Bacillus anthracis H9401 uid162021
Bacillus anthracis Sterne uid58091
Bacillus anthracis Ames Ancestor uid58083
truB

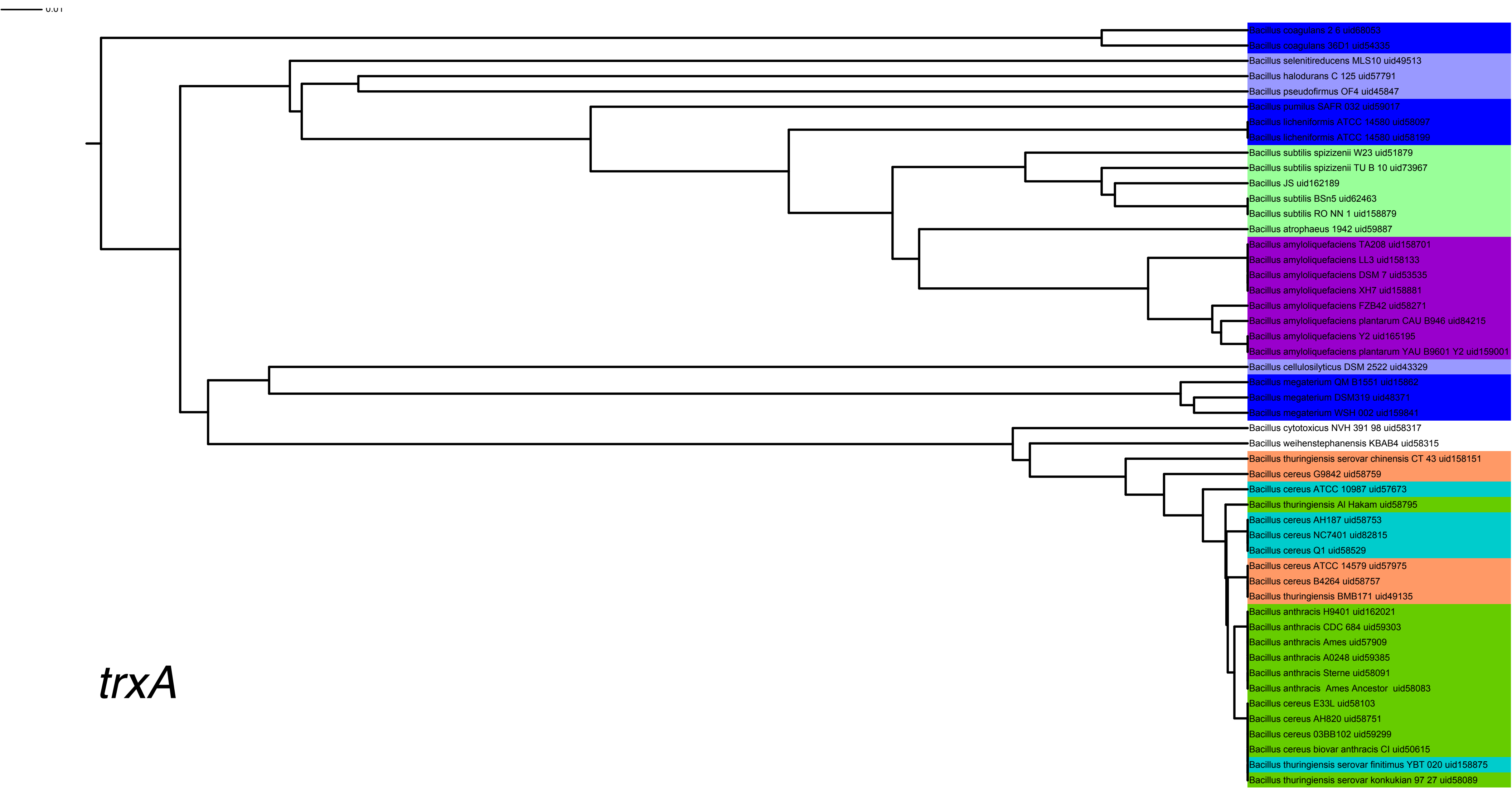

0.01
Bacillus coagulans 2 6 uid68053
Bacillus coagulans 36D1 uid54335
Bacillus selenitireducens MLS10 uid49513
Bacillus halodurans C 125 uid57791
Bacillus pseudofirmus OF4 uid45847
Bacillus pumilus SAFR 032 uid59017
Bacillus licheniformis ATCC 14580 uid58097
Bacillus licheniformis ATCC 14580 uid58199
Bacillus subtilis spizizenii W23 uid51879
Bacillus subtilis spizizenii TU B 10 uid73967
Bacillus JS uid162189
Bacillus subtilis BSn5 uid62463
Bacillus subtilis RO NN 1 uid158879
Bacillus atrophaeus 1942 uid59887
Bacillus amyloliquefaciens TA208 uid158701
Bacillus amyloliquefaciens LL3 uid158133
Bacillus amyloliquefaciens DSM 7 uid53535
Bacillus amyloliquefaciens XH7 uid158881
Bacillus amyloliquefaciens FZB42 uid58271
Bacillus amyloliquefaciens plantarum CAU B946 uid84215
Bacillus amyloliquefaciens Y2 uid165195
Bacillus amyloliquefaciens plantarum YAU B9601 Y2 uid159001
Bacillus cellulosilyticus DSM 2522 uid43329
Bacillus megaterium QM B1551 uid15862
Bacillus megaterium DSM319 uid48371
Bacillus megaterium WSH 002 uid159841
Bacillus cytotoxicus NVH 391 98 uid58317
Bacillus weihenstephanensis KBAB4 uid58315
Bacillus thuringiensis serovar chinensis CT 43 uid158151
Bacillus cereus G9842 uid58759
Bacillus cereus ATCC 10987 uid57673
Bacillus thuringiensis Al Hakam uid58795
Bacillus cereus AH187 uid58753
Bacillus cereus NC7401 uid82815
Bacillus cereus Q1 uid58529
Bacillus cereus ATCC 14579 uid57975
Bacillus cereus B4264 uid58757
Bacillus thuringiensis BMB171 uid49135
Bacillus anthracis H9401 uid162021
Bacillus anthracis CDC 684 uid59303
Bacillus anthracis Ames uid57909
Bacillus anthracis A0248 uid59385
Bacillus anthracis Sterne uid58091
Bacillus anthracis Ames Ancestor uid58083
Bacillus cereus E33L uid58103
Bacillus cereus AH820 uid58751
Bacillus cereus 03BB102 uid59299
Bacillus cereus biovar anthracis CI uid50615
Bacillus thuringiensis serovar finitimus YBT 020 uid158875
Bacillus thuringiensis serovar konkukian 97 27 uid58089
trxA

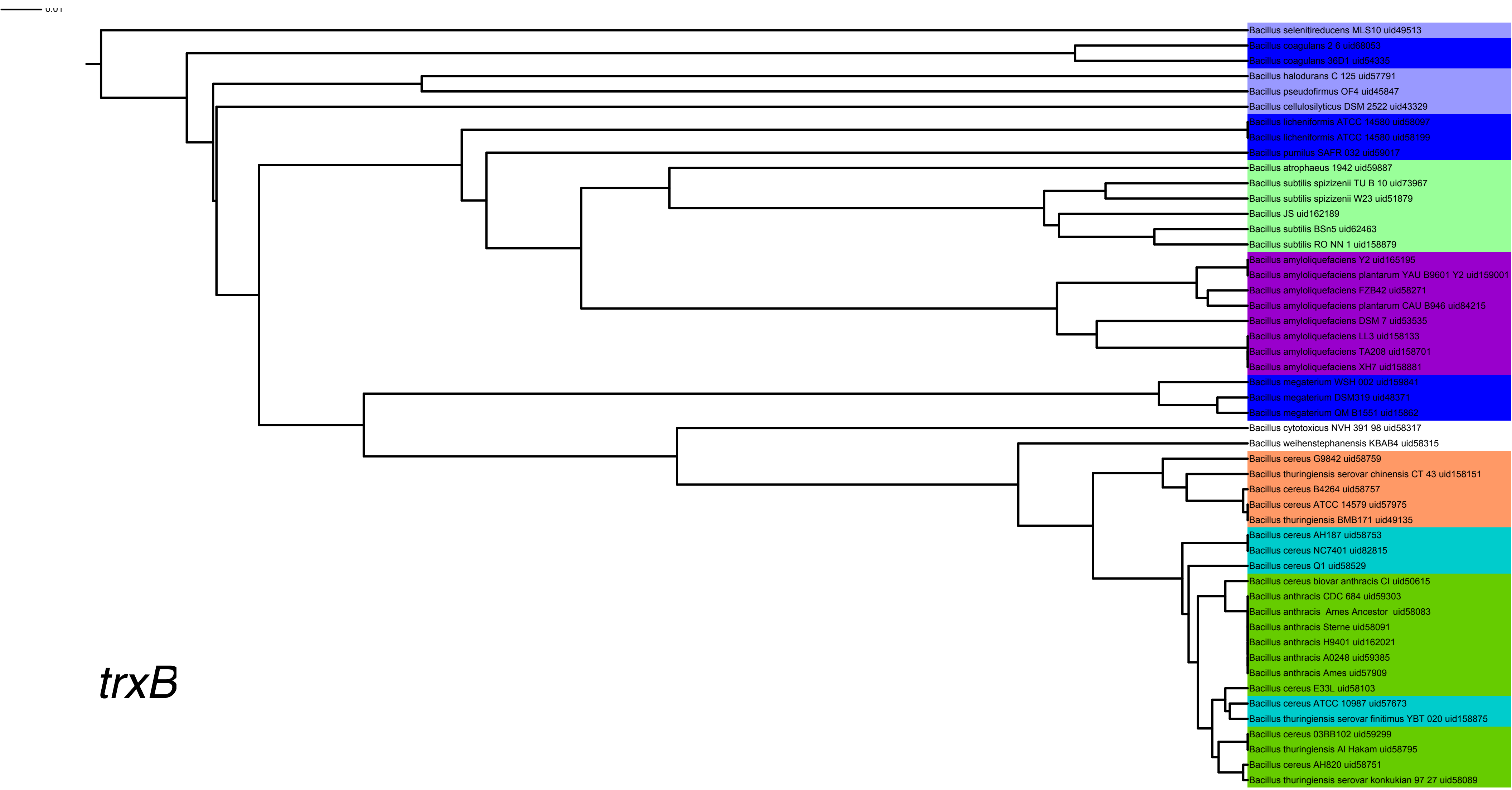
0.01
Bacillus selenitireducens MLS10 uid49513
Bacillus coagulans 2 6 uid68053
Bacillus coagulans 36D1 uid54335
Bacillus halodurans C 125 uid57791
Bacillus pseudofirmus OF4 uid45847
Bacillus cellulosilyticus DSM 2522 uid43329
Bacillus licheniformis ATCC 14580 uid58097
Bacillus licheniformis ATCC 14580 uid58199
Bacillus pumilus SAFR 032 uid59017
Bacillus atrophaeus 1942 uid59887
Bacillus subtilis spizizenii TU B 10 uid73967
Bacillus subtilis spizizenii W23 uid51879
Bacillus JS uid162189
Bacillus subtilis BSn5 uid62463
Bacillus subtilis RO NN 1 uid158879
Bacillus amyloliquefaciens Y2 uid165195
Bacillus amyloliquefaciens plantarum YAU B9601 Y2 uid159001
Bacillus amyloliquefaciens FZB42 uid58271
Bacillus amyloliquefaciens plantarum CAU B946 uid84215
Bacillus amyloliquefaciens DSM 7 uid53535
Bacillus amyloliquefaciens LL3 uid158133
Bacillus amyloliquefaciens TA208 uid158701
Bacillus amyloliquefaciens XH7 uid158881
Bacillus megaterium WSH 002 uid159841
Bacillus megaterium DSM319 uid48371
Bacillus megaterium QM B1551 uid15862
Bacillus cytotoxicus NVH 391 98 uid58317
Bacillus weihenstephanensis KBAB4 uid58315
Bacillus cereus G9842 uid58759
Bacillus thuringiensis serovar chinensis CT 43 uid158151
Bacillus cereus B4264 uid58757
Bacillus cereus ATCC 14579 uid57975
Bacillus thuringiensis BMB171 uid49135
Bacillus cereus AH187 uid58753
Bacillus cereus NC7401 uid82815
Bacillus cereus Q1 uid58529
Bacillus cereus biovar anthracis CI uid50615
Bacillus anthracis CDC 684 uid59303
Bacillus anthracis Ames Ancestor uid58083
Bacillus anthracis Sterne uid58091
Bacillus anthracis H9401 uid162021
Bacillus anthracis A0248 uid59385
Bacillus anthracis Ames uid57909
Bacillus cereus E33L uid58103
Bacillus cereus ATCC 10987 uid57673
Bacillus thuringiensis serovar finitimus YBT 020 uid158875
Bacillus cereus 03BB102 uid59299
Bacillus thuringiensis Al Hakam uid58795
Bacillus cereus AH820 uid58751
Bacillus thuringiensis serovar konkukian 97 27 uid58089
trxB

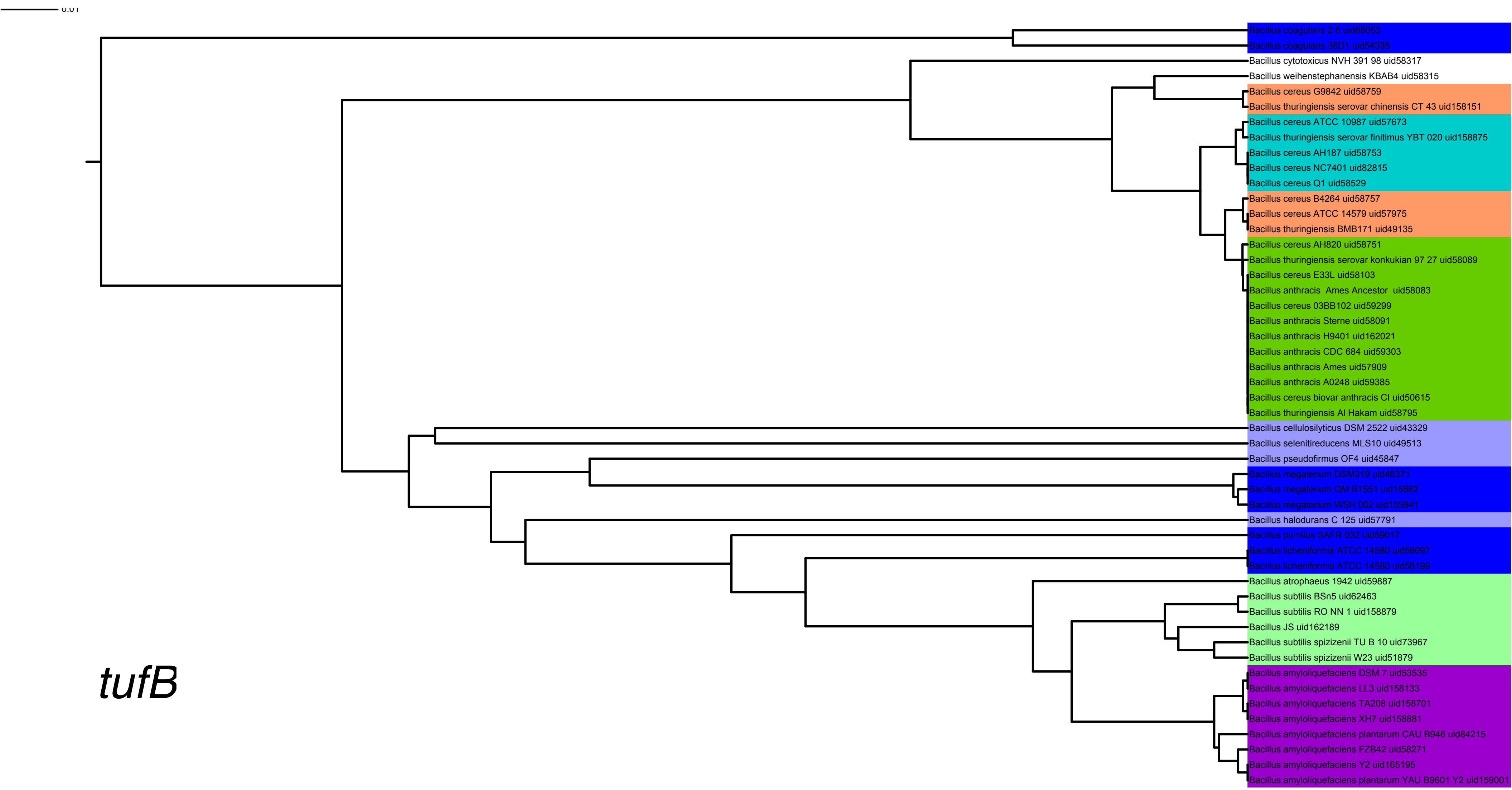
0.01
Bacillus coagulans 2 6 uid68053
Bacillus coagulans 36D1 uid54335
Bacillus cytotoxicus NVH 391 98 uid58317
Bacillus weihenstephanensis KBAB4 uid58315
Bacillus cereus G9842 uid58759
Bacillus thuringiensis serovar chinensis CT 43 uid158151
Bacillus cereus ATCC 10987 uid57673
Bacillus thuringiensis serovar finitimus YBT 020 uid158875
Bacillus cereus AH187 uid58753
Bacillus cereus NC7401 uid82815
Bacillus cereus Q1 uid58529
Bacillus cereus B4264 uid58757
Bacillus cereus ATCC 14579 uid57975
Bacillus thuringiensis BMB171 uid49135
Bacillus cereus AH820 uid58751
Bacillus thuringiensis serovar konkukian 97 27 uid58089
Bacillus cereus E33L uid58103
Bacillus anthracis Ames Ancestor uid58083
Bacillus cereus 03BB102 uid59299
Bacillus anthracis Sterne uid58091
Bacillus anthracis H9401 uid162021
Bacillus anthracis CDC 684 uid59303
Bacillus anthracis Ames uid57909
Bacillus anthracis A0248 uid59385
Bacillus cereus biovar anthracis CI uid50615
Bacillus thuringiensis Al Hakam uid58795
Bacillus cellulosilyticus DSM 2522 uid43329
Bacillus selenitireducens MLS10 uid49513
Bacillus pseudofirmus OF4 uid45847
Bacillus megaterium DSM319 uid48371
Bacillus megaterium QM B1551 uid15862
Bacillus megaterium WSH 002 uid159841
Bacillus halodurans C 125 uid57791
Bacillus pumilus SAFR 032 uid59017
Bacillus licheniformis ATCC 14580 uid58097
Bacillus licheniformis ATCC 14580 uid58199
Bacillus atrophaeus 1942 uid59887
Bacillus subtilis BSn5 uid62463
Bacillus subtilis RO NN 1 uid158879
Bacillus JS uid162189
Bacillus subtilis spizizenii TU B 10 uid73967
Bacillus subtilis spizizenii W23 uid51879
Bacillus amyloliquefaciens DSM 7 uid53535
Bacillus amyloliquefaciens LL3 uid158133
Bacillus amyloliquefaciens TA208 uid158701
Bacillus amyloliquefaciens XH7 uid158881
Bacillus amyloliquefaciens plantarum CAU B946 uid84215
Bacillus amyloliquefaciens FZB42 uid58271
Bacillus amyloliquefaciens Y2 uid165195
Bacillus amyloliquefaciens plantarum YAU B9601 Y2 uid159001
tufB

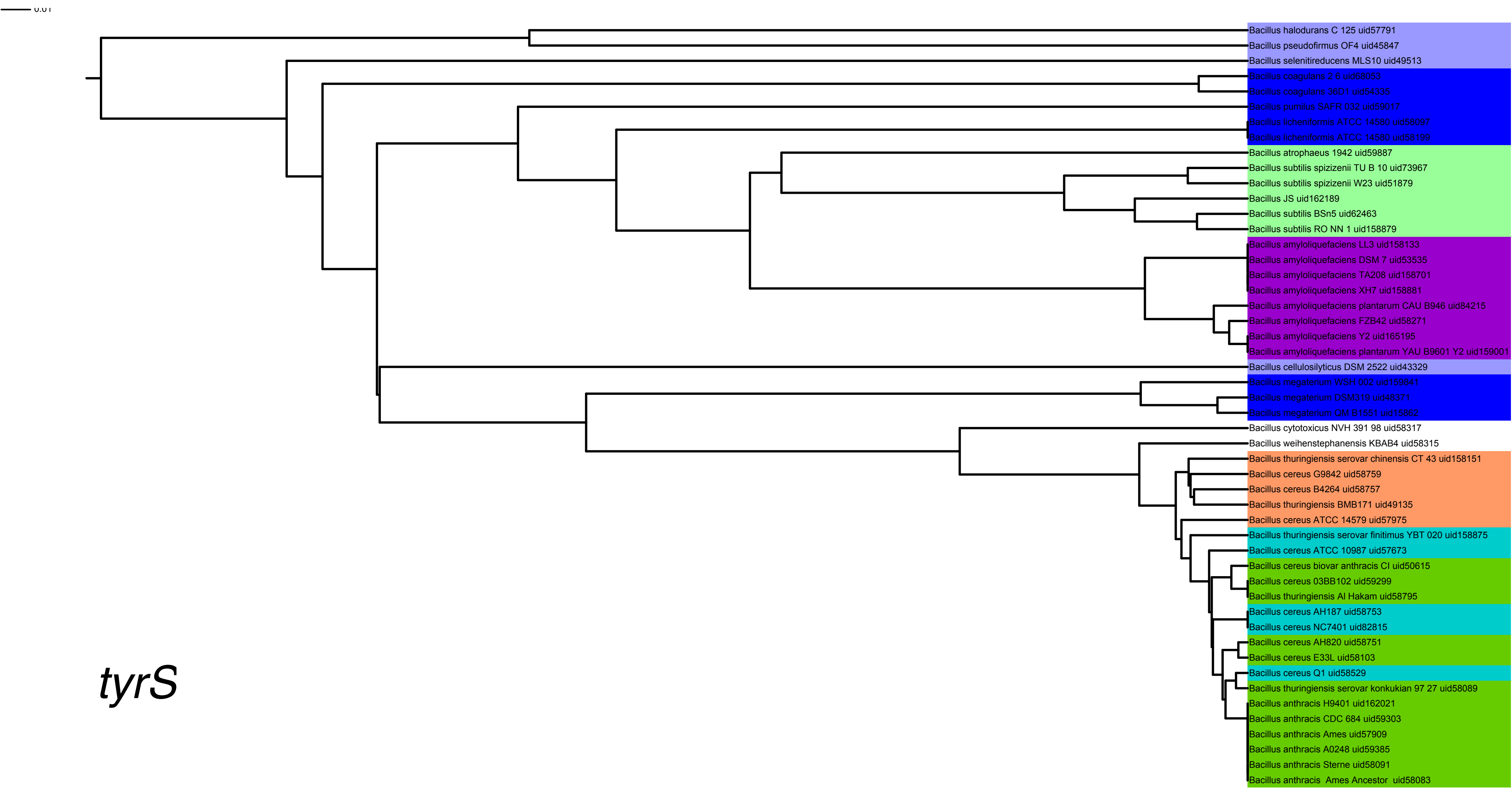
0.01
Bacillus halodurans C 125 uid57791
Bacillus pseudofirmus OF4 uid45847
Bacillus selenitireducens MLS10 uid49513
Bacillus coagulans 2 6 uid68053
Bacillus coagulans 36D1 uid54335
Bacillus pumilus SAFR 032 uid59017
Bacillus licheniformis ATCC 14580 uid58097
Bacillus licheniformis ATCC 14580 uid58199
Bacillus atrophaeus 1942 uid59887
Bacillus subtilis spizizenii TU B 10 uid73967
Bacillus subtilis spizizenii W23 uid51879
Bacillus JS uid162189
Bacillus subtilis BSn5 uid62463
Bacillus subtilis RO NN 1 uid158879
Bacillus amyloliquefaciens LL3 uid158133
Bacillus amyloliquefaciens DSM 7 uid53535
Bacillus amyloliquefaciens TA208 uid158701
Bacillus amyloliquefaciens XH7 uid158881
Bacillus amyloliquefaciens plantarum CAU B946 uid84215
Bacillus amyloliquefaciens FZB42 uid58271
Bacillus amyloliquefaciens Y2 uid165195
Bacillus amyloliquefaciens plantarum YAU B9601 Y2 uid159001
Bacillus cellulosilyticus DSM 2522 uid43329
Bacillus megaterium WSH 002 uid159841
Bacillus megaterium DSM319 uid48371
Bacillus megaterium QM B1551 uid15862
Bacillus cytotoxicus NVH 391 98 uid58317
Bacillus weihenstephanensis KBAB4 uid58315
Bacillus thuringiensis serovar chinensis CT 43 uid158151
Bacillus cereus G9842 uid58759
Bacillus cereus B4264 uid58757
Bacillus thuringiensis BMB171 uid49135
Bacillus cereus ATCC 14579 uid57975
Bacillus thuringiensis serovar finitimus YBT 020 uid158875
Bacillus cereus ATCC 10987 uid57673
Bacillus cereus biovar anthracis CI uid50615
Bacillus cereus 03BB102 uid59299
Bacillus thuringiensis Al Hakam uid58795
Bacillus cereus AH187 uid58753
Bacillus cereus NC7401 uid82815
Bacillus cereus AH820 uid58751
Bacillus cereus E33L uid58103
Bacillus cereus Q1 uid58529
Bacillus thuringiensis serovar konkukian 97 27 uid58089
Bacillus anthracis H9401 uid162021
Bacillus anthracis CDC 684 uid59303
Bacillus anthracis Ames uid57909
Bacillus anthracis A0248 uid59385
Bacillus anthracis Sterne uid58091
Bacillus anthracis  Ames Ancestor  uid58083
tyrS

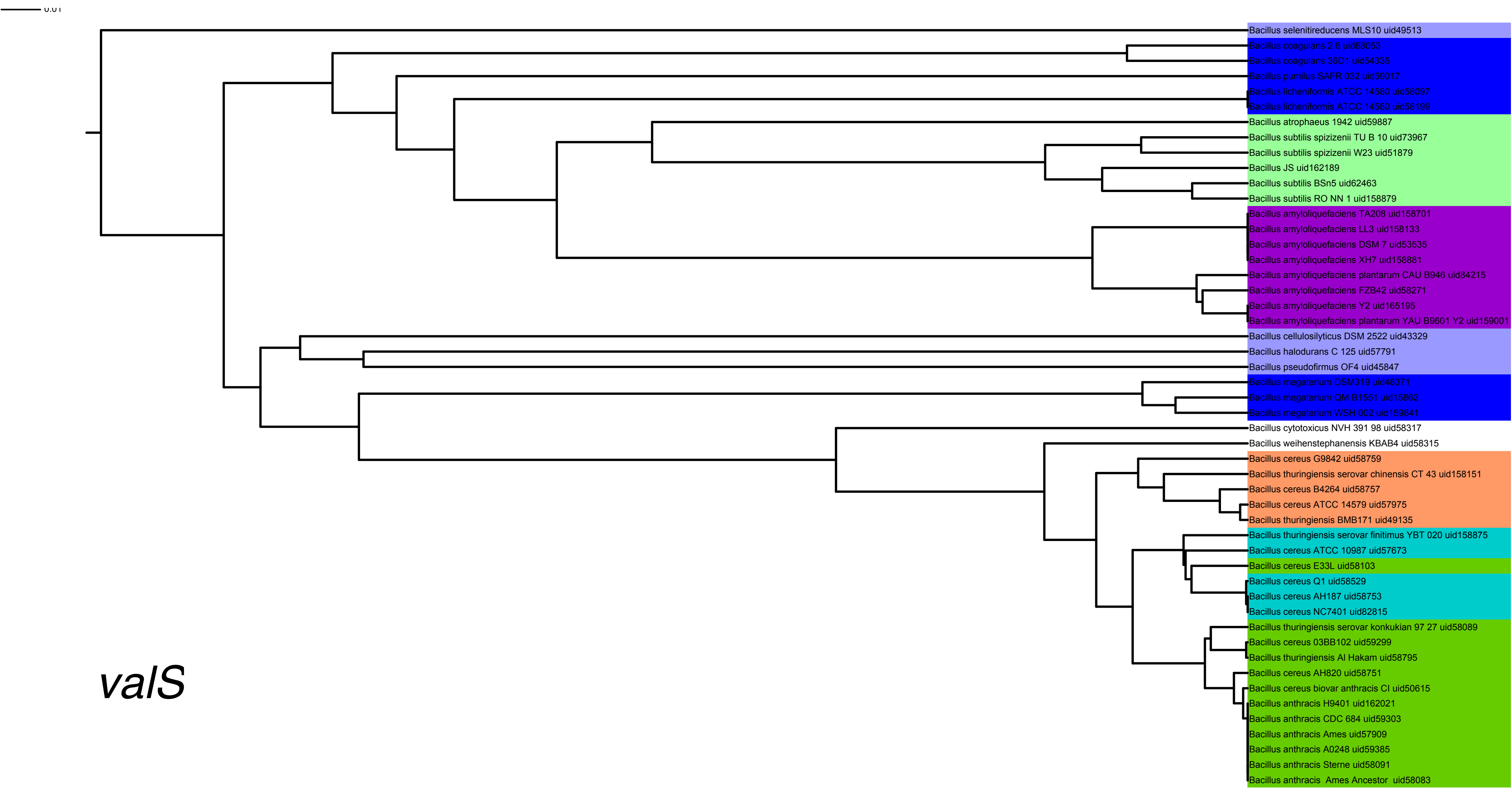
0.01
Bacillus selenitireducens MLS10 uid49513
Bacillus coagulans 2 6 uid68053
Bacillus coagulans 36D1 uid54335
Bacillus pumilus SAFR 032 uid59017
Bacillus licheniformis ATCC 14580 uid58097
Bacillus licheniformis ATCC 14580 uid58199
Bacillus atrophaeus 1942 uid59887
Bacillus subtilis spizizenii TU B 10 uid73967
Bacillus subtilis spizizenii W23 uid51879
Bacillus JS uid162189
Bacillus subtilis BSn5 uid62463
Bacillus subtilis RO NN 1 uid158879
Bacillus amyloliquefaciens TA208 uid158701
Bacillus amyloliquefaciens LL3 uid158133
Bacillus amyloliquefaciens DSM 7 uid53535
Bacillus amyloliquefaciens XH7 uid158881
Bacillus amyloliquefaciens plantarum CAU B946 uid84215
Bacillus amyloliquefaciens FZB42 uid58271
Bacillus amyloliquefaciens Y2 uid165195
Bacillus amyloliquefaciens plantarum YAU B9601 Y2 uid159001
Bacillus cellulosilyticus DSM 2522 uid43329
Bacillus halodurans C 125 uid57791
Bacillus pseudofirmus OF4 uid45847
Bacillus megaterium DSM319 uid48371
Bacillus megaterium QM B1551 uid15862
Bacillus megaterium WSH 002 uid159841
Bacillus cytotoxicus NVH 391 98 uid58317
Bacillus weihenstephanensis KBAB4 uid58315
Bacillus cereus G9842 uid58759
Bacillus thuringiensis serovar chinensis CT 43 uid158151
Bacillus cereus B4264 uid58757
Bacillus cereus ATCC 14579 uid57975
Bacillus thuringiensis BMB171 uid49135
Bacillus thuringiensis serovar finitimus YBT 020 uid158875
Bacillus cereus ATCC 10987 uid57673
Bacillus cereus E33L uid58103
Bacillus cereus Q1 uid58529
Bacillus cereus AH187 uid58753
Bacillus cereus NC7401 uid82815
Bacillus thuringiensis serovar konkukian 97 27 uid58089
Bacillus cereus 03BB102 uid59299
Bacillus thuringiensis Al Hakam uid58795
Bacillus cereus AH820 uid58751
Bacillus cereus biovar anthracis CI uid50615
Bacillus anthracis H9401 uid162021
Bacillus anthracis CDC 684 uid59303
Bacillus anthracis Ames uid57909
Bacillus anthracis A0248 uid59385
Bacillus anthracis Sterne uid58091
Bacillus anthracis  Ames Ancestor  uid58083
valS
