# Supplementary material for: Marker genes that are less conserved in their sequences are useful for predicting genome-wide similarity levels between closely related prokaryotic strains
Source: Microbiome. 2016 May 3;4:18. doi: 10.1186/s40168-016-0162-5 (PMC4853863; doi:10.1186/s40168-016-0162-5)
Supplement: Additional file 1: Figure S1. — The number of genomes in which each marker gene is identified. Out of the 79 potential marker genes, 73 are present in at least 90 % of the genomes. Figure S2. Spearman’s correlation between each marker gene and the average AAI for all complete genomes. Genes are ordered in the same way as in Fig. 3. Figure S3. Trees generated based on AAI and on percent identities of each marker gene (including 16s rRNA), for the Escherichia/Shigella clade. Figure S4. Trees generated based on AAI and on percent identities of each marker gene (including 16s rRNA), for the Streptococcus clade. Figure S5. Trees generated based on AAI and on percent identities of each marker gene (including 16s rRNA), for the Bacillus clade. Table S1. List of 79 potential marker genes surveyed, out of which 73 were found to be present in at least 90 % of the genomes. Table S2. Alternative names of 79 potential marker genes surveyed. Table S3. Split distances between UPGMA tree generated using AAI and that generated using the percent identities of each marker gene, shown in correspondence with the average percent identity ranks of the marker genes. Table S4. Designed primers for each of the 10 genes that were least conserved in their sequences in the Escherichia/Shigella lineage. (ZIP 3355 kb) [file 40168_2016_162_MOESM1_ESM.zip › SupportingInformation.pdf]

Supporting Information

Table S1. List of 79 potential marker genes surveyed, out of which 73 were found to be present in at least 90% of the genomes.

*Genes present in less than 90% of the genomes and therefore removed in analysis.

Gene Symbol	COG ID	Description
argS	COG0018	Arginyl-tRNA synthetase
*atpE**	COG0636	F0F1-type ATP synthase c subunit/Archaeal/vacuolar-type H+-ATPase subunit K
cdsA	COG0575	CDP-diglyceride synthetase
coaE	COG0237	Dephospho-CoA kinase
cpsG	COG1109	Phosphomannomutase
dnaN	COG0592	DNA polymerase sliding clamp subunit (PCNA homolog)
efp	COG0231	Translation elongation factor P/translation initiation factor eIF-5A
exo	COG0258	5-3 exonuclease (including N-terminal domain of PolI)
ffh	COG0541	Signal recognition particle GTPase
ftsY	COG0552	Signal recognition particle GTPase
fusA	COG0480	Translation elongation and release factors (GTPases)
glnS	COG0008	Glutamyl- and glutaminyl-tRNA synthetases
glyA	COG0112	Glycine hydroxymethyltransferase
groL	COG0459	Chaperonin GroEL (HSP60 family)
hisS	COG0124	Histidyl-tRNA synthetase
ileS	COG0060	Isoleucyl-tRNA synthetase
infA	COG0361	Translation initiation factor IF-1
infB	COG0532	Translation initiation factor 2 (GTPase)
ksgA	COG0030	Dimethyladenosine transferase (rRNA methylation)
leuS	COG0495	Leucyl-tRNA synthetase
map	COG0024	Methionine aminopeptidase
metG	COG0143	Methionyl-tRNA synthetase
nrdA	COG0209	Ribonucleotide reductase alpha subunit
nusG	COG0250	Transcription antiterminator
pepP	COG0006	Xaa-Pro aminopeptidase
pheS	COG0016	Phenylalanyl-tRNA synthetase alpha subunit
pheT	COG0072	Phenylalanyl-tRNA synthetase beta subunit
*proP**	COG0477	Permeases of the major facilitator superfamily
proS	COG0442	Prolyl-tRNA synthetase
pyrG	COG0504	CTP synthase (UTP-ammonia lyase)
recA	COG0468	RecA/RadA recombinase
rplA	COG0081	Ribosomal protein L1
rplB	COG0090	Ribosomal protein L2
rplC	COG0087	Ribosomal protein L3
rplD	COG0088	Ribosomal protein L4
rplE	COG0094	Ribosomal protein L5
rplF	COG0097	Ribosomal protein L6

rplJ	COG0244	Ribosomal protein L10
rplK	COG0080	Ribosomal protein L11
rplM	COG0102	Ribosomal protein L13
rplN	COG0093	Ribosomal protein L14
*rplO**	COG0200	Ribosomal protein L15
rplP	COG0197	Ribosomal protein L16/L10E
rplR	COG0256	Ribosomal protein L18
rplV	COG0091	Ribosomal protein L22
*rplW**	COG0089	Ribosomal protein L23
*rpmC**	COG0255	Ribosomal protein L29
*smtA**	COG0500	SAM-dependent methyltransferases
rplX	COG0198	Ribosomal protein L24
rpoA	COG0202	DNA-directed RNA polymerase alpha subunit/40 kD subunit
rpoB	COG0085	DNA-directed RNA polymerase beta subunit/140 kD subunit
rpoC	COG0086	DNA-directed RNA polymerase beta subunit/160 kD subunit
rpsB	COG0052	Ribosomal protein S2
rpsC	COG0092	Ribosomal protein S3
rpsD	COG0522	Ribosomal protein S4 and related proteins
rpsE	COG0098	Ribosomal protein S5
rpsG	COG0049	Ribosomal protein S7
rpsH	COG0096	Ribosomal protein S8
rpsI	COG0103	Ribosomal protein S9
rpsJ	COG0051	Ribosomal protein S10
rpsK	COG0100	Ribosomal protein S11
rpsL	COG0048	Ribosomal protein S12
rpsM	COG0099	Ribosomal protein S13
rpsN	COG0199	Ribosomal protein S14
rpsO	COG0184	Ribosomal protein S15P/S13E
rpsQ	COG0186	Ribosomal protein S17
rpsS	COG0185	Ribosomal protein S19
secY	COG0201	Preprotein translocase subunit SecY
serS	COG0172	Seryl-tRNA synthetase
thrS	COG0441	Threonyl-tRNA synthetase
tmk	COG0125	Thymidylate kinase
topA	COG0550	Topoisomerase IA
trpS	COG0180	Tryptophanyl-tRNA synthetase
truB	COG0130	Pseudouridine synthase
trxA	COG0526	Thiol-disulfide isomerase and thioredoxins
trxB	COG0492	Thioredoxin reductase
tufB	COG0050	GTPases - translation elongation factors
tyrS	COG0162	Tyrosyl-tRNA synthetase
valS	COG0525	Valyl-tRNA synthetase

Table S2. Alternative names of 79 potential marker genes surveyed.

Gene Symbol	Alternative Gene Names
argS	*argRS*
*atpE**	*papH, uncE*
cdsA	*ygdJ*
coaE	*yacE*
cpsG	*cspI*
dnaN	-
efp	-
exo	*ygdG, xni*
ffh	-
ftsY	-
fusA	*far, fus*
glnS	-
glyA	-
groL	*groEL, cpn60, mopA*
hisS	-
ileS	*ilvS*
infA	-
infB	*ssyG*
ksgA	*rsmA*
leuS	-
map	-
metG	-
nrdA	*dnaF*
nusG	-
pepP	-
pheS	-
pheT	-
*proP**	-
proS	*drpA*
pyrG	-
recA	*lexB, recH, rnmB, tif, umuB, zab*
rplA	-
rplB	-
rplC	-
rplD	*eryA*
rplE	-
rplF	-
rplJ	-
rplK	*relC*
rplM	-

rplN	-
*rplO**	-
rplP	-
rplR	-
rplV	*eryB*
*rplW**	-
*rpmC**	-
*smtA**	*ycbD*
rplX	-
rpoA	*pez, phs, sez*
rpoB	*groN, nitB, rif, ron, stl, stv, tabD*
rpoC	*tabB*
rpsB	-
rpsC	-
rpsD	*ramA*
rpsE	*spc*
rpsG	-
rpsH	-
rpsI	-
rpsJ	*nusE*
rpsK	-
rpsL	*strA*
rpsM	-
rpsN	-
rpsO	*secC*
rpsQ	*neaA*
rpsS	-
secY	*prlA*
serS	-
thrS	-
tmk	*ycfG*
topA	*supX*
trpS	-
truB	*yhbA*
trxA	*fipA, tsnC*
trxB	-
tufB	-
tyrS	-
valS	-

Table S3. Split distance between UPGMA trees generated using AAI and that generated using the percent identities of each marker gene, shown in correspondence with the average percent identity ranks of the marker genes.

	**Between *Escherichia/Shigella* genomes**		**Between *Streptococcus* genomes**		**Between *Baciilus* genomes**	
**Marker gene**	**Ave. rank**	**Split distance to AAI tree**	**Ave. rank**	**Split distance to AAI tree**	**Ave. rank**	**Split distance to AAI tree**
16S	22.4	0.923	4.0	0.592	3.6	0.638
argS	55.9	0.558	58.1	0.620	67.0	0.489
cdsA	40.5	0.731	61.6	0.620	55.7	0.298
coaE	38.5	0.731	70.3	0.606	67.2	0.340
cpsG	70.6	0.673	58.9	0.521	53.7	0.383
dnaN	61.5	0.750	58.7	0.507	59.2	0.340
efp	38.8	0.788	35.6	0.606	48.1	0.404
exo	45.9	0.846	54.0	0.563	56.8	0.447
ffh	58.7	0.635	48.9	0.549	39.1	0.362
ftsY	55.9	0.750	55.5	0.535	54.2	0.447
fusA	28.6	0.615	19.6	0.521	23.9	0.511
glnS	56.7	0.577	40.1	0.535	19.7	0.766
glyA	52.8	0.654	59.9	0.577	45.0	0.298
groL	47.2	0.769	42.4	0.549	25.2	0.511
hisS	47.4	0.712	60.9	0.606	61.3	0.298
ileS	63.8	0.577	52.2	0.634	54.4	0.362
infA	1.8	0.981	21.7	0.732	12.5	0.681
infB	47.9	0.596	39.2	0.606	43.2	0.319
ksgA	46.4	0.750	57.5	0.563	62.6	0.319
leuS	52.3	0.673	41.6	0.620	46.0	0.298
map	46.8	0.750	49.6	0.592	43.4	0.468
metG	65.8	0.731	54.4	0.535	65.2	0.447
nrdA	57.4	0.654	59.0	0.479	45.9	0.574
nusG	24.6	0.846	46.2	0.577	43.7	0.340
pepP	65.1	0.712	63.4	0.535	56.0	0.532
pheS	47.1	0.731	49.6	0.535	45.7	0.319
pheT	59.4	0.673	63.3	0.577	64.5	0.298
proS	55.8	0.635	54.6	0.521	59.9	0.340
pyrG	41.5	0.692	43.7	0.521	46.3	0.340
recA	42.8	0.654	38.1	0.535	27.8	0.447
rplA	36.7	0.788	25.1	0.634	23.9	0.553
rplB	13.1	0.923	20.9	0.620	18.4	0.447
rplC	18.3	0.923	13.5	0.634	26.4	0.511
rplD	11.3	0.923	22.8	0.662	30.7	0.660
rplE	11.2	0.942	21.0	0.606	20.5	0.532
rplF	16.8	0.923	33.6	0.549	33.8	0.553

rplJ	8.7	0.923	35.2	0.676	33.0	0.660
rplK	25.5	0.885	12.0	0.634	5.2	0.723
rplM	9.8	0.923	15.5	0.648	25.9	0.660
rplN	4.3	0.962	19.1	0.775	19.1	0.532
rplP	15.1	0.942	11.7	0.676	10.3	0.574
rplR	5.1	0.981	4.3	0.775	32.9	0.553
rplV	9.1	0.962	20.6	0.606	21.3	0.574
rplX	25.3	0.904	28.2	0.620	31.4	0.489
rpoA	11.3	0.942	36.5	0.535	22.5	0.511
rpoB	40.1	0.673	33.6	0.606	26.5	0.447
rpoC	41.3	0.654	34.5	0.563	31.2	0.404
rpsB	23.5	0.865	16.1	0.606	15.9	0.447
rpsC	13.3	0.962	15.5	0.690	19.4	0.532
rpsD	7.5	0.962	19.6	0.592	23.2	0.489
rpsE	32.6	0.788	22.0	0.662	20.9	0.489
rpsG	9.0	0.981	22.1	0.606	15.8	0.532
rpsH	3.7	0.981	24.7	0.634	18.1	0.681
rpsI	20.1	0.942	18.4	0.620	26.6	0.617
rpsJ	10.6	0.962	17.0	0.620	11.7	0.681
rpsK	21.1	0.904	8.4	0.648	14.4	0.511
rpsL	17.6	0.923	13.4	0.620	17.1	0.596
rpsM	4.8	0.962	15.7	0.634	22.9	0.723
rpsN	4.2	0.981	45.5	0.648	17.1	0.638
rpsO	13.2	0.942	19.7	0.648	16.0	0.574
rpsQ	4.2	0.942	10.2	0.718	20.8	0.532
rpsS	16.7	0.962	5.1	0.676	18.0	0.553
secY	31.3	0.827	60.1	0.535	58.4	0.574
serS	46.7	0.788	46.3	0.493	50.1	0.447
thrS	43.2	0.673	43.7	0.577	66.5	0.511
tmk	59.1	0.635	66.9	0.563	66.0	0.298
topA	53.0	0.615	54.7	0.535	52.1	0.319
trpS	57.0	0.769	51.6	0.620	57.4	0.404
truB	60.4	0.769	65.6	0.563	66.9	0.426
trxA	21.1	0.942	48.6	0.620	40.9	0.553
trxB	55.9	0.692	63.8	0.577	43.7	0.404
tufB	31.7	0.865	10.9	0.662	11.2	0.489
tyrS	44.8	0.769	50.9	0.521	56.4	0.426
valS	65.7	0.577	41.4	0.620	46.6	0.319

Table S4. Designed primers for each of the ten least conserved genes in the *Escherichia/Shigella* clade.

Gene	Primer	Melting temperature	Primer lengths	Strains aligned out of 55	Product length	Gene length	Amplified length %	A/T's among last 8 bp of 3'-end	Ambiguities
argS	Fw 5'-GCAGGYGCGCCTGCGGATTGCG<br>Rv 5'-TCAAACTGCAGCAGGCGRGCTGCCAG	74.9 / 72.2	22 / 26	55 / 55	1466	1731	85%	3 / 2	1 / 1
ffh	Fw 5'-TGTCGCGCACGCTRCGCAATATCAGYGGCCG<br>Rv 5'-TCTTCATCATGCGCTGCATGTCGTCGAACTG	75.2 / 70.0	31 / 31	55 / 55	1260	1359	93%	2 / 4	2 / 0
fusA	Fw 5'-CCGGTTCCGCTGCAGCTGGCGATTGGTGCTGAAG<br>Rv 5'-CGACCTTTGGTCAGAGAACGCAGCTGAGTTGC	74.6 / 70.3	34 / 32	55 / 55	1535	2112	73%	4 / 4	0 / 0
glnS	Fw 5'-TCAGATCATYGATGAAGATCTGGCCAGTGGTAAGC<br>Rv 5'-TCGCCCAGGTATCRCGCAGSCCAACGG	68.2 / 70.9	35 / 27	55 / 55	1616	1662	97%	4 / 2	1 / 2
ileS	Fw 5'-AGACTTTATCCGYCTGGGCGTGCTGGGCG<br>Rv 5'-GAGCAGAAGCGCATCAGACGCTGTACCACTTC	70.9 / 70.6	29 / 32	55 / 55	1777	2814	63%	1 / 4	1 / 0
infB	Fw 5'-CGCACCTTTGTGAAACGCGATCCGCAAGAGGCTG<br>Rv 5'-TGCTGGGTAATGCCGCCCGCTTCGCCAGAGGC	72.7 / 75.0	34 / 32	54 / 55	1016	2670	38%	3 / 2	0 / 0
proS	Fw 5'-CTGGCCTCCGGGTTATATACCTGGCTGCCGACCGG<br>Rv 5'-TCCATATCRGCAAACATCACGCCCGG	72.2 / 66.3	35 / 26	55 / 55	1469	1716	86%	1 / 1	0 / 1
tmk	Fw 5'-ATCGTCATTGARGGGCTGGAAGGC<br>Rv 5'-CCWGATARCGGGCGCGGGTGCG	66.1 / 72.7	24 / 22	55 / 55	511	639	80%	3 / 1	1 / 2
topA	Fw 5'-CCGCGAAGGGGAAGCCATTGCMTGGCACCTGCGGG<br>Rv 5'-AAACGCCCCATTTTCAGGTGCATTTCAG	75.0 / 64.9	35 / 28	55 / 55	1836	2595	71%	1 / 5	1 / 0
valS	Fw 5'-TGCATCATGATCCCGCCGCCGAACGTCACCGGCAG<br>Rv 5'-TCTTTGATGAAGTGCATGGTCATCATGATCATGCG	74.5 / 67.2	35 / 35	55 / 55	1481	2853	52%	2 / 4	0 / 0

Figure S1. The number of genomes in which each marker gene is identified. Out of the 79 potential marker genes, 73 are present in at least 90% of the genomes.

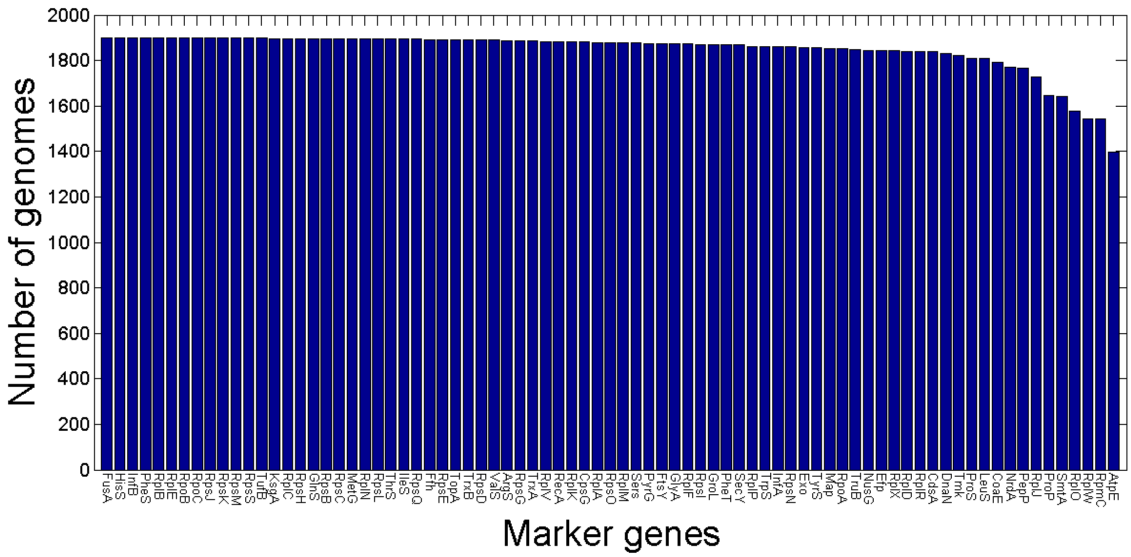

Figure S2. Spearman's correlation between each marker gene and the average AAI for all complete genomes. Genes are ordered in the same way as in Figure 3.

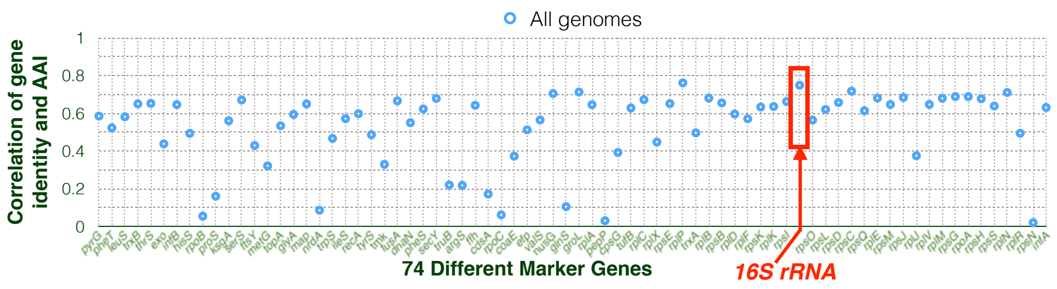

Figure S3. Trees generated based on AAI and on percent identities of each marker gene (including 16S rRNA), for the *Escherichia/Shigella* clade.

Figure S4. Trees generated based on AAI and on percent identities of each marker gene (including 16S rRNA), for the *Streptococcus* clade.

Figure S5. Trees generated based on AAI and on percent identities of each marker gene (including 16S rRNA), for the *Bacillus* clade.
